# Association between dietary antioxidant quality score and severity of coronavirus infection: a case–control study

**DOI:** 10.3389/fnut.2023.1174113

**Published:** 2023-07-06

**Authors:** Mobina Aghajani, Alexei Wong, Mehdi Azimi, Shadmehr Mirdar Harijani

**Affiliations:** ^1^Department of Exercise Physiology, Faculty of Sport Sciences, University of Mazandaran, Babolsar, Iran; ^2^Department of Health and Human Performance, Marymount University, Arlington, TX, United States; ^3^Department of Internal Medicine, School of Medicine, Firoozgar General Hospital, Iran University of Medical Sciences, Tehran, Iran; ^4^Department of Exercise Physiology, Faculty of Sport Sciences, University of Mazandaran, Babolsar, Iran; ^5^Athletic Performance and Health Research Center, University of Mazandaran, Babolsar, Iran

**Keywords:** DAQS, COVID-19, antioxidant, diet, ICU

## Abstract

The purpose of this study was to examine the association between the dietary antioxidant quality score (DAQS) and the severity of Coronavirus disease 2019 (COVID-19). The present case–control study was carried out on 295 patients diagnosed with COVID-19 (≥18 years old), including 104 critical patients (Intensive care unit [ICU] admission) and 191 COVID-19 patients without severe complications (Non-intensive care unit [Non-ICU] patients) as cases. Dietary intake was assessed by a 147-item, semi-quantitative food frequency questionnaire (FFQ). Logistic regression was performed to calculate the odds ratio (OR) and 95% confidence interval (CI) for the considered risk factors. Our outcomes (after multivariate adjustment) suggested that higher adherence to DAQS was significantly associated with a decreased risk of COVID-19 infection severity (OR = 0.12; 95% CI: 0.04–0.29, *p* < 0.001). Similar results were seen when analyzed by sex [men (OR = 0.02; 95% CI: 0.002–0.15, *p* < 0.001) and women (OR = 0.21; 95% CI: 0.06–0.68, *p* = 0.012)]. A significant association between vitamin D3 intake and decreased risk of COVID-19 severity (OR = 0.91; 95% CI: 0.89–0.94, *p* < 0.001) was also observed. Moreover, multivariate results revealed that there were no significant associations between vitamin C (OR = 1.00; 95% CI: 0.99–1.00, *p* = 0.067), vitamin E (OR = 0.98; 95% CI: 0.86–1.11, *p* = 0.798), zinc (OR = 1.02; 95% CI: 0.86–1.20, *p* = 0.805), and selenium (OR = 0.99; 95% CI: 0.99–1.00, *p* = 0.077) intakes with the risk of COVID-19 severity. However, subgroup analyses by sex suggested a significant association between vitamin C intake and the risk of COVID-19 infection severity in women (OR = 1.00; 95% CI: 1.00–1.00, *p* = 0.028). Our findings showed a negative association between DAQS adherence and the risk of COVID-19 infection severity. Our results may be used to develop potential dietary therapies to decrease COVID-19 severity.

## Introduction

Severe acute respiratory syndrome coronavirus 2 (SARS-CoV-2) appeared a little over 3 years ago, and since then, it has triggered a worldwide pandemic of acute respiratory disease known as the coronavirus disease of 2019 (COVID-19) (1). COVID-19 induces oxidative stress, which in severe cases can lead to the cytokine storm (2), a life-threatening systemic inflammatory syndrome characterized by an uncontrolled expression of immune cells and circulating cytokines (2, 3). The major risk factors for increased COVID-19 severity include male gender, obesity, smoking, older age, cancer, diabetes, cardiovascular disease (CVD), hypertension, chronic obstructive pulmonary disease (COPD), and acute kidney injury (4), which alone or together can lead to elevated oxidative stress levels and inflammation (3, 5–7). Hence, controlling or reducing oxidative stress and inflammation may be considered as the therapeutic target to reverse cytokine storm-associated outcomes (8).

Antioxidants are compounds with important scavenging power that can stop chain reactions, decomposition of hydrogen peroxide, metal-chelating, and antioxidant enzyme induction and thus can prevent or reduce oxidative damage (9). Indeed, previous studies have found that increases in total oxidative stress occur concurrently with declines in antioxidant enzymes (10, 11). Several studies reported that the new antioxidant discoveries for COVID-19 therapy are useful and highlighted the role of dietary antioxidants in COVID-19 infection severity, such as Aminothiadiazole antioxidants (12), Teriflunomide (13), polyphenolic compounds (14, 15), adenosine analogue cordycepin (16), novel 5-Substituted-2-(3,4,5-trihydroxyphenyl)-1,3,4-oxadiazoles compound (17), and novel 5-Substituted-1,3,4-thiadiazole-2-thiols compound (18). In addition, trace elements such as Zinc (Zn), Selenium (Se), Manganese (Mn), Copper (Cu), and vitamins including vitamin C and E act as cofactors of antioxidant enzymes. This is important to point out as plasma concentrations of vitamin A, vitamin C, vitamin E, Se, Zn, Mg, and Cu have been shown to be lower in COVID-19 patients compared to controls (10, 11). Although a relationship between antioxidant compounds and COVID-19 severity is not completely clear, recent literature suggested that supplementary antioxidant compounds could decrease COVID-19 severity (19). For example, previous studies documented the beneficial effects of Zn supplementation in improving gustatory sensitivity (20) and decreasing the duration of anosmia/hyposmia in COVID-19 patients (21). Moreover, selenium parenteral therapy improved respiratory mechanics in adults with acute respiratory distress syndrome (ARDS) (22). Furthermore, COVID-19 patients that underwent parenteral ascorbic acid therapy had higher survival rates (23), a lower incidence of thrombosis (24), and improvements in oxygenation (25) compared to controls. Interestingly, Bousquet et al. (26) pointed out that besides many factors such as trained immunity of the population and public hygiene, diet had an important role in the extension and severity of COVID-19. In fact, Mohajeri et al. (27) demonstrated that dietary intake of antioxidants such as vitamin D, vitamin C, vitamin E, Zn, Se, and some functional foods such as onion, garlic, and oat was significantly higher in healthy cohorts than in COVID-19 patients. Functional foods maintain gut health via the modulation of the gut microbiota, which has enormous beneficial effects (28), while antioxidant supplements may help to boost the immune system, reduce oxidative stress and inflammation, and prevent the progression of COVID-19 disease severity (27, 29). Ebrahimzade et al. (30) indicated that, compared with unhealthy or traditional dietary patterns, healthy dietary patterns can decrease the C-reactive protein (CRP) and erythrocyte sedimentation rate (ESR) levels, as well as negative COVID-19 outcomes. Prior research has also revealed that adherence to the Mediterranean diet decreases COVID-19 severity indicators such as duration of hospitalization and circulating inflammatory biomarkers (31). Additionally, a healthy plant-based diet was associated with a lower risk of severe COVID-19 (32). Despite these encouraging findings, no prior research has focused on overall dietary antioxidant intake and its possible benefits for COVID-19. Focusing on this topic is a necessary step to expand on current knowledge, as people eat a variety of foods with complex substances that can act as antioxidants (33).

The dietary antioxidant quality score (DAQS), used to calculate antioxidant-nutrient intake (vitamin A, vitamin C, vitamin E, Zn, and Se), is derived from the validated food frequency questionnaire (34) and has previously been recommended for the assessment of the overall effects of antioxidants on health-related outcomes. Indeed, some studies evaluated the association between DAQS and cardiorespiratory fitness (34), the risk of mortality among diabetic adults (35), and metabolic syndrome outcomes (36). However, no current research is available evaluating DAQS in COVID-19 hospitalized patients. Therefore, the purpose of the present cross-sectional study was to examine the association between DAQS and COVID-19 severity.

## Materials and methods

### Participants

The present case–control study was conducted at Firosgar Hospital, located in Tehran, Iran, from April to August 2022. The sample consisted of 295 patients diagnosed with COVID-19 (≥ 18 years old), including 104 critical patients (Intensive Care Unit [ICU] admission) and 191 COVID-19 patients without severe complications (Non-intensive Care Unit [Non-ICU] patients) as cases. Anthropometric measurements and biochemical parameter evaluations were performed within 24 h after hospital admission. Additionally, information on demographic characteristics, socioeconomic status, physical activity levels, and dietary intake was compiled in the same timeframe using validated questionnaires. All participants signed an informed consent form prior to participating in this research. The original ethical participants’ consent form is provided in Supplementary Material 1. This work was approved by the local ethics committee of Mazandaran University (Ethic ID: IR.UMZ.REC.1401.001) and conducted according to the Declaration of Helsinki. The original ethical approval documents are provided in Supplementary Material 2. The participants were registered based on the following inclusion criteria: adults (≥18 years old), positive SARS-CoV-2 detected using the polymerase chain reaction (PCR) method, no previous history of chronic diseases, no adherence to special diets or consumption of antioxidant supplements in the last 3 months. Participants were excluded if they were less than 18 years of age, pregnant or lactating, smokers, or had recent dietary changes. Moreover, those who did not complete questionnaires, did not respond to more than 35 food items available in the food frequency questionnaire, and those who under- or over-reported (less than 800 and more than 4,200 kcal) total daily energy intake were excluded from the analysis.

### Demographics, socioeconomic status and physical activity levels

Demographic parameters, including age, sex, education, smoking, marital status, and patients’ admission information, were gathered via a general questionnaire. Socioeconomic levels were evaluated using the Socioeconomic Survey (SES) questionnaire, consisting of five indexes, namely income, occupation, education, wealth, and place of living. Based on this score, the participants were classified into three levels of SES: low (<12), middle (≥12 to <16), and high (≥16) (37). In addition, physical activity levels were evaluated by the International Physical Activity Questionnaire (IPAQ) short form, which is an instrument designed primarily for population surveillance activity among adults (38). The validity and reliability of this questionnaire have been previously established (39).

### Biochemical parameters

Within 24 h after hospital admission, venous blood samples were collected between 8 and 10 A.M. and subsequently analyzed. Biochemical parameter levels were then incorporated into the patients’ medical history. Relevant biochemical parameter levels were extracted from the patients’ medical history, including high-sensitive C-reactive protein (hs-CRP), hydrogen potential (pH), partial carbon dioxide pressure (pCO2), base excess (BE), bicarbonate (HCO3), partial oxygen pressure (pO2), lactic dehydrogenase (LDH), creatine phosphokinase (CPK), and creatine kinase MB (CK-MB).

### Anthropometrics

The weight of participants was measured and recorded using a Seca scale with an accuracy of 100 grams. The participants’ height was measured using a commercial stadiometer. Body mass index (BMI) was calculated by dividing weight (kilograms) by the square of height (square meters).

### Dietary assessment

Dietary intake was evaluated using a valid and reliable (40), 147-item semi-quantitative food frequency questionnaire (FFQ) to determine the usual dietary intake during the year preceding the assessment. Energy and nutrient intake were estimated using the Nutritionist IV software. The DAQS was evaluated based on the daily dietary intake of vitamin E, vitamin A, vitamin C, selenium, and zinc compared to the daily recommended intake (DRI) (41). This comparison was performed for each of the five mentioned nutrients, and the following values were assigned: 0 if the intake was <2/3 of the DRI and 1 if the intake was >2/3 of the DRI. The total DAQS score was calculated by adding the scores of the five antioxidant nutrients, with a range of 0 (very poor quality) to 5 (high quality) (41). To examine the association between DAQS and COVID-19 severity, the DAQS score was divided into three tertiles as follows: Tertile 1: 0 < DAQS score < 3, Tertile 2: DAQS score = 3, and Tertile 3: DAQS score > 3.

### Statistical analysis

Baseline characteristics were summarized using mean and standard deviation for continuous variables, and frequencies (%) were utilized for categorical variables. One-way analysis of variance and chi-square tests were used to compare continuous and categorical variables between the groups. Logistic regression was performed to calculate the odds ratio (OR) and 95% confidence interval (CI) for the identified risk factors. Variables with a significance level of *p* < 0.2 were included in the multivariable model for further analysis. Bivariable regression models, referred to as model 1, were initially presented without adjustment for participant characteristics. This was followed by multivariable regression models, specifically model 2, which accounted for age (years). Additional adjustments were made in model 3, which included age (years), saturated fats (g/d), linoleic fat (g/d), iron (mg/d), biotin (mg/d), dietary fiber (g/d), β-carotene (mg/d), BMI (kg/m2), and magnesium (mg/d). To assess the association between DAQS tertiles, dietary antioxidants, and COVID-19 severity, ORs and 95% CI were estimated. These associations were adjusted for confounding factors. Separate models were developed for sex to examine potential sex-specific effects. Statistical significance was set at *p* < 0.05. All analyses were performed using STATA Version 16 (Stata Corp).

## Results

### Participant’s characteristics

Participant’s anthropometrics and biochemical parameters are reported in Table 1. Our results showed that the age (mean ± SD) of ICU patients (62.4 ± 15.3 years, *p* < 0.001) was significantly higher than non-ICU patients (56.5 ± 14.8 years). Compared to Non-ICU, ICU patients had a higher hs-CRP concentration (16.0 ± 20.1 vs. 9.1 ± 17.8 mg/dL, *p* < 0.001), D-Dimer (1.39 ± 1.58 vs. 0.98 ± 1.12 mg/L, *p* = 0.01), LDK (712.6 ± 276.6 vs. 559.0 ± 219.2 U/L, *p* < 0.001), CPK (213.1 ± 267.1 vs. 104.8 ± 243.7, *p* < 0.001) and CK-MB (32.8 ± 50.1 vs. 19.5 ± 37.2 U/L, *p* < 0.001). However, weight (*p* = 0.415), BMI (*p* = 0.101), PH (*p* = 0.402), pCO2 (*p* = 0.461), BB (*p* = 0.533), HCO3 (*p* = 0.176) and pO2 (*p* = 0.787) did not significantly differ between groups. In addition, non-ICU patients had higher levels of ferritin compared to their ICU counterparts (376.2 ± 166.9 vs. 289.5 ± 163.2 mg/L, *p* < 0.001).

**Table 1:** Participant’s anthropometrics and biochemical parameters.

Variables	Non-ICU patients (*N* = 191)	ICU patients (*N* = 104)	*p*-value
Age(years)	56.54 ± 14.8	62.44 ± 15.26	<0.001
Weight (kg)	76.22 ± 12.8	77.5 ± 12.7	0.415
BMI (kg/m2)	27.45 ± 4.1	28.31 ± 4.73	0.101
hs-CRP (mg/dL)	9.07 ± 17.8	16.0 ± 20.1	<0.001
PH	7.37 ± 0.15	7.35 ± 0.21	0.402
pCO_2_ (mm Hg)	41.5 ± 22.8	39.56 ± 12.8	0.461
BB (mmol/L)	44.79 ± 9.2	44.17 ± 6.2	0.533
HCO_3_(mEq/L)	22.4 ± 4.58	21.5 ± 5.9	0.176
pO_2_ (mm Hg)	38.5 ± 18.4	39.1 ± 17.9	0.787
Ferritin (μg/l)	376.17 ± 166.9	289.47 ± 163.2	<0.001
D-Dimer (mg/L)	0.98 ± 1.12	1.39 ± 1.58	0.011
LDH (U/L)	559.02 ± 219.2	712.58 ± 276.6	<0.001
CPK(U/L)	104.8 ± 243.7	213.12 ± 267.1	<0.001
CK-MB (U/L)	19.48 ± 37.2	32.79 ± 50.09	0.010

BMI, Body mass index. hs-CRP, High-sensitive C-reactive protein. PH, Hydrogen potential. pCO2, Partial dioxide carbon pressure. BE, Base excess. HCO3, Bicarbonate. pO2, Partial oxygen pressure. LDH, Lactic dehydrogenase. CPK, Preatine phosphokinase. CK-MB, creatine kinase MB.

Statistical significance was set at *p* < 0.05.

Participant’s characteristics, physical activity levels and nutrient intake by DAQS tertiles are illustrated in Table 2. Our outcomes indicated that there were fewer critical status patients in highest tertile of DAQS compare to lowest tertile (T3 vs. T1) (*p* < 0.001). Most dietary intake items differed significantly between DAQS tertiles, such as intake of energy (*p* < 0.001), protein (*p* < 0.001), total fat (*p* < 0.001), saturated fats (*p* = 0.040), polyunsaturated fats (*p* < 0.001), linoleic fats (*p* < 0.001), calcium (*p* < 0.001), iron (*p* < 0.001), magnesium (*p* < 0.001), zinc (*p* < 0.001), manganese (*p* < 0.001), fluoride (*p* = 0.020), vitamin E (*p* < 0.001), vitamin B1 (*p* < 0.001), B6 (*p* < 0.001), vitamin C (*p* < 0.001), vitamin A (*p* < 0.001), soluble fiber (*p* = 0.007), crude fiber (*p* = 0.001), glucose (*p* < 0.001), fructose (*p* < 0.001), lactose (*p* < 0.001), oleic fat (*p* < 0.001), sodium (*p* = 0.08), phosphorus (*p* < 0.001), copper (*p* < 0.001), selenium (*p* = 0.001), B2 (*p* < 0.001), B6 (*p* = 0.001), B12 (*p* < 0.001), biotin (*p* < 0.001), vitamin D (*p* < 0.001), dietary fiber (*p* < 0.001), insoluble fiber (*p* < 0.001), sugar (*p* < 0.001), galactose (*p* = 0.0008), sucrose (*p* = 0.0012), α-Carotene (*p* < 0.001) and β-Carotene (*p* < 0.001). There were no significant differences in other factors, including age (*p* = 0.647), weight (*p* = 0.392), BMI (*p* = 0.143), sex (*p* = 0.236), education (*p* = 0.717), socioeconomic status (p = 0.23), physical activity levels (*p* = 0.441), smoking (*p* = 0.173), marital status (*p* = 0.754) as well as EPA-Omega 3 (*p* = 0.125), sodium (*p* = 0.084) and maltose (*p* = 0.440) intakes.

**Table 2:** Participant’s characteristics, socioeconomic status, physical activity levels, and nutrient intake by dietary antioxidant quality score tertiles.

Variables	Tertile 1 0 < DAQS score < 3 (*N* = 85)	Tertile 2 DAQS score = 3 (*N* = 114)	Tertile 3 DAQS score > 3 (*N* = 96)	*p*-value
Age(years)	59.3 ± 16.2	57.57 ± 14.13	59.2 ± 15.2	0.647
Weight (kg)	77 ± 12.7	78 ± 12.5	75.3 ± 13.2	0.392
BMI (kg/m2)	27.6 ± 4.1	28.3 ± 4.4	27.1 ± 4.2	0.143
Sex				
Men	46(%54.12)	52(%45.61)	40(%41.67)	0.236
Women	39(%45.88)	62(%54.39)	56(%58.33)	
Patients’ status				
General	41(48.24)	71(62.28)	79(82.29)	<0.001
Intensive care unit	44(51.76)	43(37.72)	17(17.71)	
Education				
Under-graduate	42 (49.41)	51(44.73)	45(46.87)	0.717
Graduate	43(50.59)	63(55.27)	51(53.13)	
Social-economical status				
Low	31(36.47)	28(24.56)	26(37.50)	0.237
Moderate	28(32.94)	52(45.61)	30(31.25)	
High	26(30.58)	34(29.82)	35(36.45)	
Physical activity levels (MET)	1254.8 ± 135.2	1164.9 ± 693.4	1286.02 ± 807.5	0.441
Smoking (yes)	12 (14.11)	8(0.07)	7(0.07)	0.173
Marital status (married)	67 (78.82)	90(78.94)	72(0.75)	0.754
Energy (kcal/day)	1723.4 ± 796.1	1526.3 ± 135.2	2183.3 ± 764.6	<0.001
Protein (g/day)	95.4 ± 74.6	138.5 ± 98.6	107.6 ± 70.7	<0.001
Fat (g/day)	88.29 ± 67.89	131.89 ± 100.07	99.36 ± 77.7	<0.001
Saturated fats (g/day)	22.42 ± 10.5	25.31 ± 10.6	26.24 ± 10.8	0.046
Polyunsaturated fats (g/day)	20.8 ± 7.4	22.2 ± 10.9	27.5 ± 9.5	<0.001
Linoleic fat (g/day)	9.19 ± 4.7	9.62 ± 8.1	13.37 ± 7.5	<0.001
EPA-Omega 3 (g/day)	0.028 ± 0.04	0.044 ± 0.06	0.040 ± 0.05	0.125
Calcium (g/day)	841.7 ± 397.5	773.2 ± 579.4	1,263 ± 629.1	<0.001
Iron (mg/day)	18.44 ± 12.7	17.91 ± 15.3	34.9 ± 33.5	<0.001
Magnesium (mg/day)	322.3 ± 131.1	287.7 ± 204.3	477.15 ± 189.7	<0.001
Zinc (mg/day)	8.81 ± 3.3	8.71 ± 4.7	11.87 ± 4.3	<0.001
Manganese (mg/day)	18.02 ± 31.38	37.18 ± 47.7	20.3 ± 34.4	<0.001
Fluoride (mg/day)	2237.9 ± 1428.4	1727.4 ± 1584.3	2162.7 ± 1221.4	0.025
Vitamin A (IU/day)	996.2 ± 182.4	1928.7 ± 275.2	1458.5 ± 211.5	0.018
Vitamin E (mg/day)	8.93 ± 2.30	10.10 ± 3.92	13.57±	<0.001
B1(mg/day)	1.72 ± 0.45	1.84 ± 0.48	2.08 ± 0.51	<0.001
B6(Ug/day)	53.4 ± 153.9	157.6 ± 240.4	52.02 ± 168.4	<0.001
Vitamin C (mg/day)	224.9 ± 362.6	468.38 ± 556.8	295.7 ± 346.3	<0.001
Vitamin K (mg/day)	326.6 ± 387.4	598.36 ± 543.5	608.7 ± 509.9	<0.001
Soluble fiber (g/day)	0.54 ± 0.5	0.92 ± 0.8	0.75 ± 0.9	0.007
Crude fiber (g/day)	19.7 ± 31.7	37.7 ± 44.3	22.8 ± 31.0	0.001
Glucose (g/day)	12.1 ± 6.2	11.89 ± 8.4	18.2 ± 7.8	<0.001
Fructose (g/day)	16.8 ± 6.4	19.7 ± 7.6	23.3 ± 9.6	<0.001
Lactose (g/day)	9.14 ± 6.3	9.2 ± 9.7	12.9 ± 9.4	0.003
Oleic fat (g/day)	17.5 ± 6.9	19.5 ± 10.3	24.8 ± 9.1	<0.001
Sodium (mg/day)	3602.7 ± 1059.9	3712.4 ± 1006.6	3923.7 ± 944.0	0.084
Phosphorus (mg/day)	1334.8 ± 282.8	1438.6 ± 338.2	1669.9 ± 336.6	<0.001
Copper (mg/day)	2.19 ± 2.1	3.71 ± 3.5	2.7 ± 2.6	<0.001
Selenium (mg/day)	292.2 ± 600.1	789.6 ± 1170.04	411.19 ± 1042.06	0.001
B 2 (mg/day)	3.74 ± 6.3	8.17 ± 9.8	4.17 ± 6.14	<0.001
B 6 (mg/day)	53.4 ± 15.3	157.09 ± 24	52.02 ± 16.8	0.001
B12 (Ug/day)	5.2 ± 7.2	10.3 ± 11.3	5.77 ± 9.02	<0.001
Biotin (mg/day)	26.6 ± 10.1	22.03 ± 14.9	36.6 ± 16.1	<0.001
Vitamin D (*μg/d*)	24.9 ± 36.2	31.3 ± 55.6	27.7 ± 34.6	<0.001
Dietary fiber (g/day)	32.3 ± 17.7	26.3 ± 20.3	43.16 ± 19	<0.001
Insoluble fiber (mg/day)	2.84 ± 2.9	4.84 ± 4.3	3.99 ± 2.9	<0.001
Sugar (g/day)	83.9 ± 35.2	80.28 ± 51.2	119.7 ± 45.7	<0.001
Galactose (g/day)	4.31 ± 6.7	7.4 ± 8.7	3.90 ± 5.6	<0.001
Sucrose (g/day)	17.42 ± 8.7	17.07 ± 8.9	21.29 ± 9.2	0.001
Maltose (g/day)	1.49 ± 0.7	1.55 ± 0.8	1.65 ± 0.7	0.440
α-Carotene (mg/day)	917.9 ± 991.0	1427.9 ± 1499.0	1868.1 ± 1137.0	<0.001
β-Carotene (mg/day)	3036.19 ± 1758.4	3196.1 ± 2273.2	7229.01 ± 4391.1	<0.001

MET, Metabolic equivalent of task; EPA, Eicosapentaenoic acid.

Statistical significance was set at *p* < 0.05.

Table 3 shows the results of the multivariable model for the association between dietary antioxidants and risk of COVID-19 severity. After multivariate adjustment, we observed a significant association between vitamin D intake and decreased risk of severity of COVID-19 infection (OR = 0.91; 95% CI: 0.89–0.94, *p* < 0.001). Sex subgroup analyses showed similar results among men (OR = 0.87; 95% CI: 0.82–0.92, *p* < 0.001) and women (OR = 0.94; 95% CI: 0.91–0.97, *p* < 0.001). Moreover, multivariate results revealed that there was no significant association between vitamin C (OR = 1.00; 95% CI: 0.99–1.00, *p* = 0.067), vitamin E (OR = 0.98; 95% CI: 0.86–1.11, *p* = 0.798), Zn (OR = 1.02; 95% CI: 0.86–1.20, *p* = 805), and Se (OR = 0.99; 95% CI: 0.99–1.00, *p* = 0.077) intake and risk of COVID-19 infection severity. However, sex subgroup analyses suggested a significant positive association between vitamin C intake and enhanced risk of COVID-19 infection severity in women (OR = 1.00; 95% CI: 1.00–1.00, *p* = 0.028).

**Table 3:** Odds ratios (ORs) and 95% confidence intervals (CIs) for the association between dietary antioxidants and risk of COVID-19 severity multivariable model.

	All		Men		Women	
Variables	OR (%95 CI)	*p*-value	OR (%95 CI)	*p*-value	OR (%95 CI)	*p*-value
Vitamin C						
Model 1	1.00 (0.99–1.00)	0.251	0.99(0.99–1.00)	0.715	1.00(1.00–1.00)	0.059
Model 2	1.00 (0.99–1.00)	0.161	0.99(0.99–1.00)	0.944	1.00 (0.99–1.00)	0.051
Model 3	1.00 (0.99–1.00)	0.067	0.99(0.99–1.00)	0.751	1.00(1.00–1.00)	0.028
Vitamin E						
Model 1	0.96 (0.90–1.02)	0.267	0.91 (0.83–1.00)	0.077	1.010 (0.92–1.10)	0.819
Model 2	0.95 (0.89–1.02)	0.180	0.90 (0.81–0.99)	0.047	1.00 (0.92–1.10)	0.883
Model 3	0.98 (0.86–1.11)	0.798	0.94 (0.78–1.15)	0.598	1.08 (0.89–1.31)	0.385
Vitamin D						
Model 1	0.92(0.89–0.94)	<0.001	0.89(0.85–0.92)	*p* < 0.001	0.94 (0.91–0.97)	<0.001
Model 2	0.92(0.89–0.94)	<0.001	0.87(0.83–0.92)	*p* < 0.001	0.94 (0.91–0.97)	<0.001
Model 3	0.91(0.89–0.94)	<0.001	0.87(0.82–0.92)	*p* < 0.001	0.94 (0.91–0.97)	<0.001
Zinc						
Model 1	0.98 (0.92–1.03)	0.458	0.98(0.91–1.06)	0.748	1.97(0.90–1.04)	0.434
Model 2	0.96 (0.91–1.01)	0.181	0.94(0.87–1.03)	0.226	0.96 (0.89–1.04)	0.373
Model 3	1.02 (0.86–1.20)	0.805	0.94 (0.72–1.22)	0.647	1.05 (0.83–1.35)	0.643
Selenium						
Model 1	1.00(0.99–1.00)	0.783	1.00(0.99–1.00)	0.855	1.00(0.99–1.00)	0.746
Model 2	1.00(0.99–1.00)	0.620	1.00(0.99–1.00)	0.643	1.00(0.99–1.00)	0.710
Model 3	0.99 (0.99–1.00)	0.077	1.00(0.99–1.00)	0.847	0.99(0.99–1.00)	0.073

Model 1: Crude. Model 2: Adjusted for age. Model 3: Adjusted for age, BMI, Saturated fats, Linoleic fat, Iron, Biotin, Dietary fiber, β-Carotene, and Magnesium.

Statistical significance was set at *p* < 0.05.

Outcomes from the logistic regression univariable model for the factors associated with COVID-19 severity are reported in Table 4. Our results suggested that age (as a factor) is positively associated with severity of COVID-19 infection (OR = 1.02; 95% CI: 1.01–1.04, *p* = 0.002). Moreover, vitamin D (OR = 0.92; 95% CI: 0.89–0.94, *p* < 0.001) and dietary fiber (OR = 0.98; 95% CI: 0.97–0.99, *p* = 0.034) intakes are associated with COVID-19 infection severity. The associations are not significant for other potential factors (*p* > 0.05).

**Table 4:** Odds ratios (ORs) and 95% confidence intervals (CIs) for the factors associated with severity of COVID-19: results from logistic regression univariable model.

Variables	OR (%95 CI)	*p*-value
Age(years)Total	1.02 (1.01–1.04)	0.002
Weight (kg) Total	1.00 (0.98–1.02)	0.412
BMI (kg/m2) Total	1.04 (0.99–1.10)	0.107
Gender (Women)	1.17(0.72–1.89)	0.517
Energy (kcal/day)	0.99 (0.99–1.00)	0.230
Protein (g/day)	1.00 (0.99–1.00)	0.597
Fat (g/day)	1.00 (0.99–1.00)	0.563
Saturated fats (g/day)	1.01 (0.99–1.03)	0.187
Polyunsaturated fats (g/day)	0.99 (0.97–1.01)	0.642
Linoleic fat (g/day)	0.97 (0.93–1.00)	0.092
EPA-Omega 3 (g/day)	0.13 (0.001–13.31)	0.389
Calcium (g/day)	0.99 (0.99–1.00)	0.251
Iron (mg/day)	0.99 (0.97–1.00)	0.105
Magnesium (mg/day)	0.99 (0.99–1.00)	0.168
Zinc (mg/day)	0.98 (0.92–1.03)	0.458
Manganese (mg/day)	1.00 (0.99–1.00)	0.459
Fluoride (mg/day)	0.99 (0.99–1.00)	0.951
Vitamin A (IU/day)	1.00 (0.99–1.00)	0.532
Vitamin E (mg/day)	0.96 (0.90–1.02)	0.267
B1(mg/day)	0.81 (0.50–1.32)	0.412
B6(Ug/day)	1.00 (0.99–1.00)	0.569
Vitamin C (mg/day)	1.00 (0.99–1.00)	0.251
Vitamin K (mg/day)	0.99 (0.99–1.00)	0.624
Soluble fiber (g/day)	0.98 (0.74–1.31)	0.930
Crude fiber (g/day)	1.00 (0.99–1.00)	0.765
Glucose (g/day)	0.99 (0.96–1.02)	0.629
Fructose (g/day)	0.99 (0.97–1.02)	0.917
Lactose (g/day)	1.00 (0.97–1.03)	0.701
Oleic fat (g/day)	0.99 (0.96–1.01)	0.542
Sodium (mg/day)	0.99 (0.99–1.00)	0.603
Phosphorus (mg/day)	0.99 (0.99–1.00)	0.910
Copper (mg/day)	1.03 (0.95–1.11)	0.398
Selenium (mg/day)	1.00 (0.99–1.00)	0.783
B2 (mg/day)	1.00 (0.98–1.03)	0.522
B12 (Ug/day)	1.00 (0.98–1.03)	0.571
Biotin (mg/day)	0.98 (0.97–1.00)	0.173
Vitamin D (*μg/d*)	0.92 (0.89–0.94)	<0.001
Dietary fiber (g/day)	0.98 (0.97–0.99)	0.034
Insoluble fiber (mg/day)	1.01 (0.95–1.08)	0.604
Sugar (g/day)	0.99 (0.99–1.00)	0.425
Galactose (g/day)	1.01 (0.98–1.04)	0.337
Sucrose (g/day)	0.99 (0.96–1.02)	0.755
Maltose (g/day)	1.07 (0.77–1.50)	0.649
α-Carotene (mg/day)	1.00 (0.99–1.00)	0.895
β-Carotene (mg/day)	0.99 (0.99–1.00)	0.065

Statistical significance was set at *p* < 0.05.

Results of the logistic regression multivariable model (T1 as reference group) analyses evaluating the association between DAQS and severity of COVID-19 infection are reported in Table 5. Our crude model outcomes suggest that increases in DAQS as a continuous variable are related to a significant decrease in the risk of COVID-19 infection severity (OR = 0.20; 95% CI: 0.10–0.38, *p* < 0.001). Sex subgroup analyses show consistent results in men (OR = 0.12; 95% CI: 0.03–0.36, *p* < 0.001) and women (OR = 0.28; 95% CI: 0.11–0.70, *p* = 0.005). In addition, the relationship remained significant even after multivariate adjustment (OR = 0.12; 95% CI: 0.04–0.29, *p* < 0.001). Moreover, the same results are observed in men (OR = 0.02; 95% CI: 0.002–0.15, *p* < 0.001) and women (OR = 0.21; 95% CI: 0.06–0.68, *p* = 0.012). Supplementary Material 3 provides detailed statistical calculations for all the reported results. Additionally, Supplementary Material 4 includes the original statistical analytical documents supporting the findings of this study.

**Table 5:** Odds ratios (ORs) and 95% confidence intervals (CIs) for the association between Dietary Antioxidant Quality Score tertiles and COVID-19 severity.

DAQS	All		Men		Women	
	OR(%95 CI)	*P* for trend	OR (%95 CI)	*P* for trend	OR (%95 CI)	*P* for trend
Model 1						
Tertile 1	1	<0.001	1	<0.001	1	0.005
Tertile 2	0.56 (0.31–0.99)		0.37(0.16–0.85)		0.81(0.36–1.81)	
Tertile 3	0.20 (0.10–0.39)		0.12(0.03–0.36)		0.28(0.11–0.70)	
Model 2						
Tertile 1	1	<0.001	1	<0.001	1	0.005
Tertile 2	0.58(0.32–1.03)		0.36(0.15–0.88)		0.83 (0.37–1.86)	
Tertile 3	0.18(0.09–0.37)		0.09(0.03–0.31)		0.28 (0.11–0.70)	
Model 3						
Tertile 1	1	<0.001	1	<0.001	1	0.012
Tertile 2	0.47 (0.25–0.89)		0.26 (0.09–0.72)		0.71 (0.30–1.68)	
Tertile 3	0.12(0.04–0.29)		0.02 (0.005–0.15)		0.21 (0.06–0.68)	

Results from logistic regression multivariable model (T1 as reference group).

Model 1: Crude. Model 2: Adjusted for Age. Model 3: Adjusted for Age, Saturated fats, Linoleic fat, Iron, Biotin, Dietary fiber, β-Carotene, BMI, and Magnesium. Statistical significance was set at *p* < 0.05.

## Discussion

The aim of the present case–control study was to examine the association between DAQS and COVID-19 infection severity. Our results indicate a negative relationship between DAQS and the risk of COVID-19 infection severity. This relationship remained significant after adjusting for possible confounders, as well as in the subgroups of men and women. To our knowledge, this is the first study to examine the association between DAQS and COVID-19 outcomes.

Several investigations have reported an association between selected dietary antioxidants and COVID-19-related outcomes (29, 42–46). Vitamins A, B group, C, and D, as well as trace elements such as Zn, Se, Cu, and Iron, are listed by the European Food Safety Authority as micronutrients that can maintain immune system function (47). Furthermore, previous studies have highlighted the role of vitamin E, vitamin K, and Mg in the management of infectious respiratory disease (48–52). COVID-19, like other viral infections, can induce oxidative stress by enhancing reactive oxygen species (ROS) production and weakening defense mechanisms. Neutrophils and mononuclear phagocytic cells are also responsible for the widespread release of ROS into lung tissue (53). Additionally, the massive release of tumor necrosis factor-alpha (TNF-α) during the cytokine storm could exacerbate ROS production and, consequently, the severity of the COVID-19 infection (54, 55). The excessive formation of ROS could be controlled by appropriate antioxidant intake (43) as these play the role of free radical scavengers that can lower or terminate chain reactions (56). Therefore, due to the bold role of antioxidants in the pathogenesis of COVID-19, these compounds have been proposed as an adjuvant therapy in the management of this condition (43).

Our results revealed that vitamin D intake was negatively associated with the severity of COVID-19 infection. Similar outcomes were found in the analyses of the male and female subgroups. In agreement with our findings, a previous study in Iranian patients showed that vitamin D intake lower than 25 ng/ml was associated with COVID-19 infection severity (57). Dror et al. (58), in a case–control study, reported that levels of vitamin D in severe or critical disease were lower than those in patients with mild or moderate disease. Additionally, Sulli et al. (59) reported that the risk of death and severe COVID-19 infection was higher among elderly patients with insufficient 25-OH-vitamin D serum levels compared to patients with sufficient levels. While the previously mentioned studies were performed in adult cohorts, the association between serum vitamin D3 levels and clinical COVID-19 outcomes in children has been investigated in several studies (60, 61). For instance, Alpcan et al. (60) reported that there was a significant relationship between vitamin D levels and COVID-19 infection severity in children. Similarly, Yilmaz et al. (61) suggested that pediatric patients with COVID-19 had significantly lower vitamin D levels than control groups, along with a negative association between symptomatology and vitamin D serum levels. Vitamin D supplementation also appears to benefit COVID-19 outcomes in hospitalized patients. For example, Annweiler et al. (62) found that early administration of a single oral high dose of vitamin D3 (400,000 IU) versus the standard dose (50,000 IU) can improve overall mortality rates among at-risk older patients with COVID-19. Furthermore, Zurita-Cruz et al. (63) reported that vitamin D3 supplementation in COVID-19 pediatric hospitalized patients decreased disease severity and death. The inverse association between vitamin D and COVID-19 infection severity highlighted in this study, among many others, may be related to its role as a major immunologic mediator. Indeed, uncontrolled inflammation is the main factor in the severity of clinical COVID-19 outcomes such as ARDS, myocarditis, microvascular thrombosis, and/or the cytokine storm (64). Vitamin D supplementation can increase T regulatory lymphocyte (Tregs) levels, the principal defense mechanism against uncontrolled inflammation (64, 65). Additionally, vitamin D3 can control the cytokine storm by downregulating the release of inflammatory cytokines, such as TNF-alpha and Interleukin 6 (66). Some studies showed that higher levels of vitamin D were associated with lower levels of D-dimer and thrombotic complications (67, 68). Hence, vitamin D may regulate thrombotic pathways (69).

**Figure 1:**
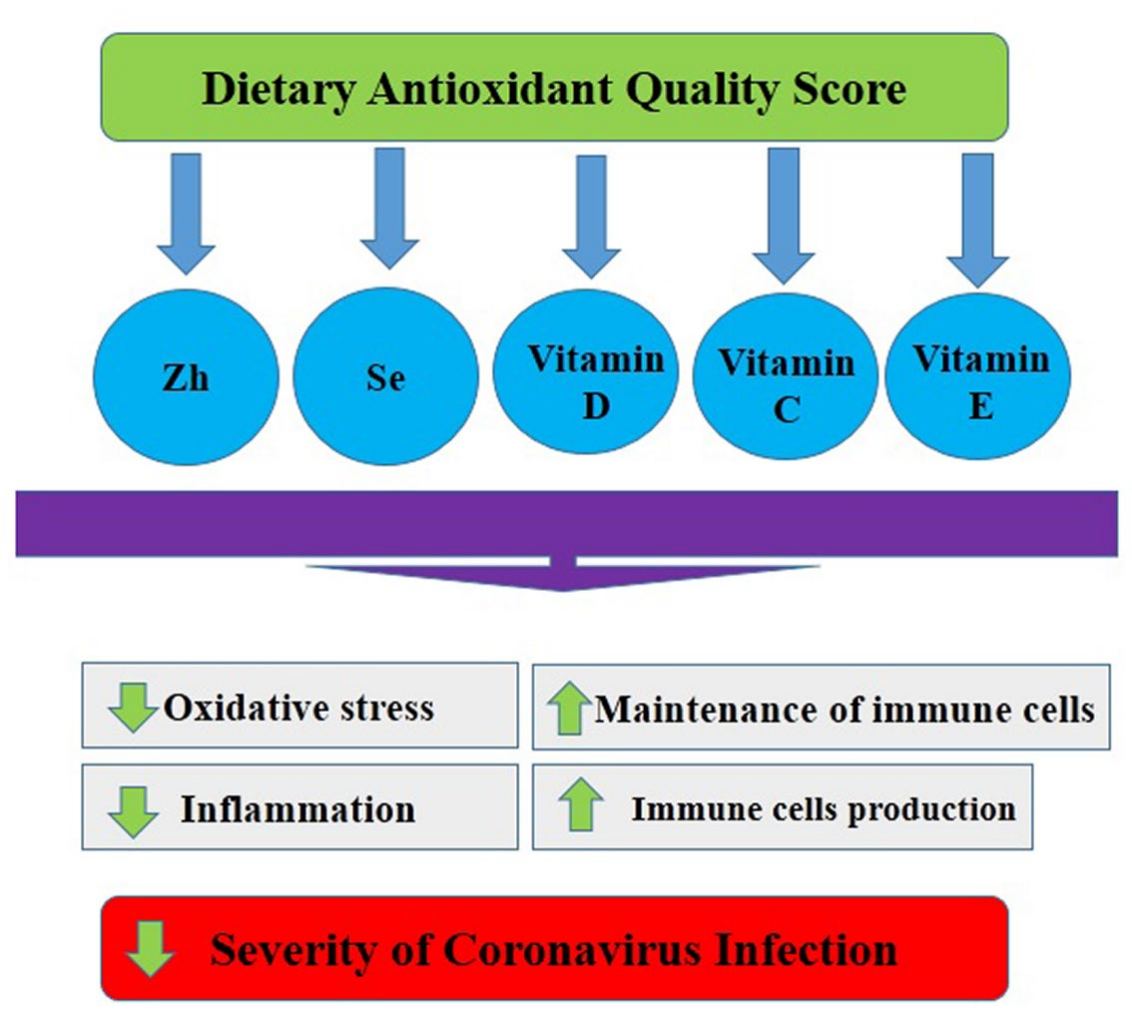
Possible mechanisms of the relationship between dietary antioxidant quality score and Covid-19.

Another important outcome of this research is that low vitamin C intake was associated with an increased risk of COVID-19 infection severity in women, but no significant association was found in males. Previous studies have investigated the relationship between vitamin C and COVID-19 infection severity, but research findings are contradictory and controversial (70–72). Tomasa-Irriguible et al. (71) indicated that up to 82% of ICU patients had low plasma vitamin C values. Similarly, Chiscano-Camon et al. (70) reported that the plasma vitamin C levels in individuals with acute respiratory distress syndrome were very low. Additionally, Shahbaz et al. (72) reported that high doses of intravenous ascorbic acid may be beneficial for critically ill and older COVID-19-infected patients, by reducing inflammation and improving oxygenation status, leading to a decrease in mortality. However, a recent randomized controlled trial showed that treatment of severe COVID-19 with high-dose intravenous vitamin C did not improve outcomes in infected individuals compared to controls (73). The discrepant results on the effectiveness of ascorbic acid for the treatment of COVID-19 severity may be related to several factors such as dosage utilized, age, genetic background, and characteristics of infected participants. Nevertheless, vitamin C is well-known to play a major role in immune functions (72). It is a cofactor of enzymes such as dioxygenases and ketoglutarate, which can neutralize ROS and regenerate vitamin E. Moreover, vitamin C can deplete or block Neutrophil Extracellular Trap (NETosis) formation and control the cytokine storm in the alveolar region, which is relevant in COVID-19 (72). Additionally, vitamin C can block Interleukin-1 and TNF-mediated suppression of kappaBalpha (NFκB) activation. NFκB is a nuclear transcription factor and plays a central role in altered gene expression during inflammation (74) (Figure 1).

We also found that the age of ICU patients was significantly higher than that of non-ICU patients, and age (as a factor) was positively associated with the severity of COVID-19 infection. In line with our results, previous studies found that the risk of severe COVID-19 infection was higher in elderly patients compared to young and middle-aged cohorts (75–78). These studies documented that DNA damage, telomere dysfunction, epigenetic disruption, mitogenic signaling, and oxidative stress increase with aging (75–78). Furthermore, inflammation, immunosenescence, cellular senescence, and immune cell dysfunction contribute to chronic inflammation with age, providing another possible link between aging and the severity of COVID-19 infection (78). Furthermore, declines in sex steroids and growth hormone levels during the aging process may impact immune regulation and, consequently, disease severity (77). Moreover, comorbidities such as hypertension, diabetes, coronary artery disease, chronic kidney disease, and hyperlipidemia are common in the elderly, and previous studies have shown that these comorbidities were predictors of the severity of COVID-19 infection (75). Thus, advanced age may be considered an important risk factor for the severity of COVID-19 infection.

Another outcome of the current research is that dietary fiber intake was inversely associated with the severity of COVID-19 infection. The dietary fiber is divided into two groups: water-soluble fiber and non-water-soluble fiber (79). Water-soluble fibers can be fermented by intestinal microorganisms and modulate intestinal microbiota (79). Moreover, many soluble fibers are prebiotics, and they can feed the bacteria that are beneficial for the digestive system (80). Prior findings by Shahramian et al. (81) supported the use of galactan and fructan as prebiotics in COVID-19 infant formula, as these reduced upper-respiratory infection. Additionally, Olaimat et al. (82) reported that COVID-19 patients have an imbalanced gut microbiota and that the non-digestible fibers in vegetables have an anti-inflammatory and antioxidant effect that can support microbiota-mediated antiviral immunity (83). Importantly, dietary fibers have a prebiotic effect on immunity by the growth and function of probiotics. For instance, fermentable probiotics including fructan, glucan, and arabinoxylan produce short-chain fatty acids (SCFAs). These metabolites can increase host immunity through improvement in specific receptor intensity, such as G protein-coupled receptor activation (84). Also, metabolites such as butyric acid and propionic acid have beneficial effects on T cells, macrophages, and dendritic cell function (85). In this study, levels of biomarkers such as hs-CRP, D-Dimer, LDH, CPK, and CKMB were higher in the ICU compared to non-ICU patients. In line with our results, Xu et al. (86) reported that D-Dimer levels in COVID-19 patients were associated with inflammatory factors and organ function. A body of knowledge also reported that CRP concentration (87), CK-MB (88), CK (89), and LDH (90) levels were higher in COVID-19 patients who died compared to survivors and that these biomarkers were predictors of COVID-19 infection severity.

The present study showed no significant association between Zn and Se intake and the severity of COVID-19 infection. Zinc, an essential trace metal, plays a crucial role in the development and maintenance of immune cells. Insufficient zinc levels may lead to impaired humoral function and cell-mediated immunity (91). Several studies have reported that zinc status can impact the outcome of COVID-19 infection, including disease duration and the risk of persistent respiratory infections with severe symptoms (92–94). Selenium, another vital trace element, is essential for mammalian redox biology (91). In the absence of adequate dietary selenium and in the presence of increased oxidative stress in the host, a viral genome can transform from a mildly pathogenic virus into a highly virulent agent. This phenomenon was observed with the Coxsackie 3B virus in Keshan disease in individuals deficient in selenium (91, 95). Moreover, it has been suggested that selenium deficiency may substantially contribute to the genesis of SARS-CoV (91, 96). Given the roles and importance of zinc and selenium in immune system function, their maximum impact is likely observed in patients with deficiencies in these minerals. Considering the lack of association between these elements and the severity of COVID-19 infection, the most plausible explanation could be that the patients had sufficient levels of these elements in their bodies.

Our findings also revealed no significant association between vitamin E intake and the severity of COVID-19 infection. However, there is limited information regarding the effects of vitamin E or selenium supplementation in humans with COVID-19 infection. Previous studies have suggested that vitamin E may impact respiratory tract infection severities through several possible mechanisms (97–99). Vitamin E acts through antioxidant mechanisms to initiate T-cell activation signals, increase the count of T cells, enhance mitogenic lymphocyte activities, enhance IL-2 cytokine secretion, enhance NK cell responses, and reduce the risk of infection (100). Despite these beneficial roles in immunity, a prior clinical trial reported that supplementation with 200 mg of vitamin E did not have a favorable effect on the incidence and severity of acute respiratory tract infections in well-nourished noninstitutionalized elderly individuals (97). Another study conducted by Meydani et al. (101) revealed that supplementation with 200 IU per day of vitamin E did not have a statistically significant effect on lower respiratory tract infections in elderly nursing home residents. Similarly, Hakamifard et al. (102) demonstrated that the combination of oral vitamins C (1,000 mg daily) and E (400 IU daily) supplementation had no beneficial effect in COVID-19 patients. However, it is worth noting that while vitamin E supplementation did not exhibit sharp efficacy in COVID-19 infection management, it was reported that administering 60 or 200 mg of vitamin E daily resulted in a higher humoral immune response to poliovirus and fewer mutant viral strains (101). Therefore, it is suggested that vitamin E supplementation may improve the efficacy of the COVID-19 vaccination (103), although it does not directly impact infection management.

### Strengths and limitations

Our study has some strengths. To our knowledge, this was the first study to investigate the association between DAQS and COVID-19 severity. Indeed, this was also the first study in this research area assessing numerous factors and biomarkers. Nevertheless, this work is not without limitations. First, even though valid and widely used self-reported questionnaires were utilized for the assessment of dietary intake (40), this method might lead to the misclassification of food intake. Second, other confounding factors such as race/ethnicity or genetic background were not evaluated in this study. Lastly, we did not evaluate any mechanisms that may explain the relationship between DAQS and the severity of COVID-19. Therefore, to explain the possible mechanisms, future studies must assess biological biomarkers and factors associated with the severity of COVID-19.

### Implications for practice and research

The outcomes of this cross-sectional study have important implications for both practice and future research. The study demonstrated a significant and negative relationship between DAQS and the risk of COVID-19 infection severity among adults. However, to establish a causal relationship between dietary DAQS score and COVID-19 infection severity, further investigation is required. Additionally, the use of antioxidant therapy shows promising potential as an adjuvant therapy in the prevention and management of infection severity in adults with COVID-19. However, more research is needed to validate these findings and determine the specific antioxidants that are most effective in this context. Antioxidants such as vitamin E, vitamin A, vitamin C, selenium, and zinc should be specifically investigated in future longitudinal studies and/or clinical trials to better understand their relationship with the risk of COVID-19 severity associated with DAQS score.

## Conclusion

In summary, this study suggests that a lower age and higher intake of DAQS, vitamin D, and dietary fibers are associated with a lower risk of COVID-19 severity. Although we found no relationship between vitamin C intake and the risk of severe COVID-19 infection, subgroup analysis by sex showed a negative association in women. Future gender-specific studies should be conducted to provide detailed recommendations for each gender. Furthermore, clinical trial studies with larger sample sizes are needed to support the veracity of our findings.

## Data availability statement

The original contributions presented in the study are included in the article/Supplementary material, further inquiries can be directed to the corresponding author.

## Ethics statement

All participants signed an informed consent prior to partaking in this research. This work was approved by the local ethics committee of Mazandaran University (Ethic ID: IR.UMZ.REC.1401.001) and performed according to the Declaration of Helsinki. The patients/participants provided their written informed consent to participate in this study.

## Author contributions

MoA and SH: concept and design, acquisition, analysis, interpretation of data, and statistical analysis. MoA, AW, MeA, and SH: drafting of the manuscript. SH: supervision. All authors contributed to critical revision of the manuscript for important intellectual content, and read and approved the final manuscript.

## Conflict of interest

The authors declare that the research was conducted in the absence of any commercial or financial relationships that could be construed as a potential conflict of interest.

## Publisher’s note

All claims expressed in this article are solely those of the authors and do not necessarily represent those of their affiliated organizations, or those of the publisher, the editors and the reviewers. Any product that may be evaluated in this article, or claim that may be made by its manufacturer, is not guaranteed or endorsed by the publisher.
