# Supplementary material for: Association between dietary antioxidant quality score and severity of coronavirus infection: a case–control study
Source: Front Nutr. 2023 Jul 6;10:1174113. doi: 10.3389/fnut.2023.1174113 (PMC10358364; doi:10.3389/fnut.2023.1174113)
Supplement: Supplementary file 1 [file Data_Sheet_1.PDF]

consent form

first name and last name…………………

I hereby agree that as a person studied in the research "Effect of respiratory muscle training, spirometry".

Encouraging and complementary help of acetylcysteine on hematological profile, blood gases, some inflammatory markers and structure and Pulmonary function of hospitalized patients with corona virus infection" under the supervision of Mrs. Mobina Aghajani..

It was explained to me that in case of any problem arising from respiratory muscle exercises and spirometry or taking supplements If it is acetylcysteine, all medical support will be provided to me.

It also gave me the opportunity to discuss and ask questions about this research and about the difficulty of the work and possible risks He informed me.

Therefore, knowing that the medical information and the results of this research will be used by the officials of the project and the personal information of the individuals will be kept confidential and the names of the subjects will not be mentioned. I have declared my readiness to participate in this research and I consider it my natural right that at every stage ofResearch, without mentioning any reason, I will be able to refuse to continue cooperation

Therefore, I agree to carry out this research.

Signature:

Date:

Address and phone:
