# Supplementary material for: Association between dietary antioxidant quality score and severity of coronavirus infection: a case–control study
Source: Front Nutr. 2023 Jul 6;10:1174113. doi: 10.3389/fnut.2023.1174113 (PMC10358364; doi:10.3389/fnut.2023.1174113)
Supplement: Supplementary file 2 [file Data_Sheet_2.PDF]

University of Mazandaran

# Research Ethics Committees Certificate

| | | | |
|---|---|---|---|
| Approval ID: | IR.UMZ.REC.1401.001 | Approval Date: | 2022-01-26 |
| Evaluated by: | Research Ethics Committees of University of Mazandaran | | |
| Status: | Approved | | |
| Approval Statement: | The project was found to be in accordance to the ethical principles and the national norms and standards for conducting Medical Research in Iran.<br>Notice:<br>1. Although the proposal has been approved by the Biomedical Research Ethics Committee, meeting the professional and legal requirements is the sole responsibility of the PI and other project collaborators.<br>2. This certificate is reliant on the proposal/documents received by this committee on 2022-01-26. The committee must be notified by the PI as soon as the proposal/documents are modified. | | |
| Thesis Title: | Effect of Respiratory Muscle Training, Incentive Spirometry and N-Acetylcysteine Supplement on Metabolic status, Mood status, Sleep quality, Inflammatory Biomarkers and Pulmonary Scan of Hospitalized Coronavirus Infection Patients | | |
| Supervisor: | Name: Shadmehr mirdar harijani<br>Email: s.mirdar@umz.ac.ir | | |
| Student: | Name: Mobina aghajani<br>Email: Aghajani.mobina@gmail.com | | |

Dr. Heshmatalah Alinezhad
Committee Director
University of Mazandaran

Dr. Jamal Ghasemi
Committee Secretary
University of Mazandaran
