# Supplementary material for: Association between dietary antioxidant quality score and severity of coronavirus infection: a case–control study
Source: Front Nutr. 2023 Jul 6;10:1174113. doi: 10.3389/fnut.2023.1174113 (PMC10358364; doi:10.3389/fnut.2023.1174113)
Supplement: Supplementary file 3 [file Data_Sheet_3.PDF]

Code	Age	Sex	High	Wieght	BMI	Bastary_Sa	Education
226.00	20.00	1	170.00	75.00	25.95	1.00	1.00
41.00	20.00	1	190.00	110.00	30.47	1.00	1.00
280.00	21.00	1	193.00	67.00	17.99	1.00	0.00
93.00	25.00	2	160.00	70.00	27.34	1.00	1.00
229.00	26.00	2	168.00	58.00	25.55	1.00	0.00
294.00	28.00	1	167.00	76.00	27.25	1.00	1.00
143.00	28.00	2	157.00	65.00	26.37	2.00	0.00
193.00	30.00	1	168.00	60.00	21.26	2.00	1.00
187.00	30.00	1	175.00	80.00	26.12	2.00	1.00
228.00	31.00	1	182.00	73.00	26.04	1.00	0.00
157.00	31.00	2	163.00	80.00	30.11	2.00	1.00
243.00	32.00	1	180.00	90.00	27.78	1.00	1.00
185.00	32.00	1	172.00	80.00	27.04	2.00	1.00
88.00	33.00	2	163.00	76.00	28.60	1.00	1.00
136.00	33.00	2	165.00	65.00	23.88	2.00	0.00
3.00	34.00	1	188.00	96.00	27.16	1.00	1.00
270.00	34.00	2	160.00	65.00	25.39	1.00	0.00
235.00	34.00	2	154.00	47.00	19.82	1.00	1.00
159.00	34.00	2	167.00	74.00	26.53	2.00	1.00
38.00	35.00	1	185.00	98.00	26.63	1.00	0.00
39.00	35.00	1	163.00	93.00	35.00	1.00	0.00
50.00	35.00	1	180.00	70.00	21.60	1.00	1.00
287.00	35.00	2	153.00	40.00	17.09	1.00	1.00
19.00	36.00	2	170.00	52.00	17.99	1.00	1.00
277.00	36.00	2	164.00	64.00	23.80	1.00	1.00
148.00	36.00	2	165.00	56.00	20.57	2.00	1.00
126.00	36.00	2	165.00	86.00	31.59	2.00	0.00
34.00	37.00	1	182.00	85.00	25.66	1.00	1.00
215.00	37.00	2	153.00	69.00	29.48	1.00	0.00
104.00	37.00	2	189.00	91.00	25.48	1.00	0.00
111.00	37.00	2	158.00	56.00	22.43	2.00	1.00
254.00	38.00	1	180.00	98.00	30.25	1.00	1.00
242.00	38.00	1	167.00	100.00	35.86	1.00	1.00
265.00	38.00	1	167.00	87.00	31.20	1.00	1.00
14.00	38.00	1	175.00	82.00	26.78	1.00	1.00
27.00	38.00	1	175.00	85.00	27.76	1.00	1.00
221.00	38.00	2	174.00	74.00	24.44	1.00	0.00
174.00	38.00	2	155.00	75.00	31.22	2.00	0.00
70.00	39.00	1	175.00	108.00	25.27	1.00	0.00
1.00	39.00	1	152.00	56.00	22.24	1.00	0.00
28.00	39.00	1	163.00	70.00	26.35	1.00	1.00
216.00	39.00	1	163.00	70.00	26.35	1.00	1.00
231.00	39.00	2	169.00	68.00	23.81	1.00	0.00
217.00	40.00	1	160.00	78.00	30.47	1.00	0.00
220.00	40.00	1	177.00	90.00	28.73	1.00	1.00
176.00	40.00	1	170.00	88.00	30.45	2.00	1.00
289.00	40.00	2	159.00	55.00	21.76	1.00	1.00
134.00	40.00	2	160.00	80.00	29.25	2.00	1.00
113.00	40.00	2	153.00	73.00	31.18	2.00	0.00
91.00	41.00	1	178.00	80.00	25.25	1.00	1.00
227.00	41.00	2	170.00	75.00	25.95	1.00	1.00
59.00	41.00	2	165.00	70.00	25.71	1.00	1.00

76.00	42.00	1	174.00	85.00	28.08	1.00	0.00
232.00	42.00	2	164.00	70.00	26.03	1.00	0.00
102.00	42.00	2	176.00	90.00	29.05	1.00	0.00
241.00	42.00	2	165.00	83.00	30.49	1.00	1.00
84.00	43.00	2	171.00	80.00	27.36	1.00	1.00
8.00	44.00	1	172.00	78.00	26.37	1.00	1.00
292.00	45.00	1	172.00	82.00	27.72	1.00	1.00
271.00	45.00	2	165.00	65.00	23.88	1.00	0.00
160.00	45.00	2	168.00	65.00	23.03	2.00	1.00
214.00	46.00	1	172.00	80.00	27.04	1.00	1.00
236.00	46.00	1	170.00	80.00	27.68	1.00	1.00
77.00	47.00	1	178.00	68.00	21.46	1.00	1.00
237.00	47.00	2	161.00	78.00	30.09	1.00	0.00
213.00	47.00	2	170.00	68.00	23.53	1.00	1.00
29.00	48.00	2	160.00	71.00	27.73	1.00	0.00
100.00	48.00	2	166.00	82.00	29.76	1.00	1.00
274.00	48.00	2	170.00	82.00	28.37	1.00	1.00
124.00	48.00	2	167.00	75.00	26.89	2.00	0.00
249.00	49.00	1	167.00	68.00	24.38	1.00	0.00
266.00	49.00	1	175.00	96.00	31.35	1.00	1.00
283.00	49.00	1	176.00	80.00	25.83	1.00	1.00
259.00	49.00	1	185.00	80.00	23.37	1.00	1.00
47.00	49.00	2	165.00	75.00	27.55	1.00	0.00
279.00	49.00	2	160.00	59.00	23.05	1.00	1.00
21.00	49.00	2	158.00	81.00	32.45	1.00	1.00
51.00	50.00	1	180.00	100.00	30.86	1.00	1.00
246.00	50.00	1	177.00	77.00	24.58	1.00	1.00
200.00	50.00	1	168.00	77.00	27.28	2.00	0.00
167.00	50.00	1	183.00	83.00	24.78	2.00	1.00
57.00	51.00	1	170.00	70.00	24.22	1.00	1.00
52.00	51.00	1	170.00	82.00	28.37	1.00	1.00
245.00	51.00	2	156.00	65.00	26.71	1.00	0.00
282.00	51.00	2	153.00	63.00	26.91	1.00	1.00
263.00	51.00	2	170.00	86.00	29.76	1.00	1.00
20.00	51.00	2	159.00	84.00	33.23	1.00	1.00
195.00	51.00	2	160.00	85.00	33.20	2.00	1.00
268.00	52.00	1	174.00	85.00	27.08	1.00	0.00
4.00	52.00	1	160.00	75.00	29.30	1.00	1.00
2.00	52.00	2	180.00	70.00	24.60	1.00	1.00
166.00	52.00	2	160.00	105.00	41.02	2.00	1.00
202.00	52.00	2	170.00	80.00	27.68	2.00	1.00
161.00	52.00	2	158.00	83.00	33.25	2.00	1.00
71.00	53.00	1	165.00	90.00	33.06	1.00	1.00
208.00	53.00	1	185.00	90.00	36.30	2.00	1.00
152.00	53.00	2	150.00	70.00	31.11	2.00	0.00
181.00	53.00	2	160.00	75.00	29.30	2.00	0.00
291.00	54.00	1	154.00	66.00	27.83	1.00	0.00
67.00	54.00	1	175.00	73.00	23.84	1.00	1.00
180.00	54.00	1	175.00	113.00	36.90	2.00	0.00
5.00	54.00	2	165.00	97.00	35.63	1.00	1.00
12.00	54.00	2	157.00	75.00	30.43	1.00	1.00
17.00	54.00	2	157.00	94.00	38.14	1.00	1.00
219.00	54.00	2	164.00	84.00	31.23	1.00	1.00

23.00	54.00	2	162.00	83.00	31.63	1.00	1.00
129.00	54.00	2	165.00	80.00	29.38	2.00	1.00
290.00	55.00	1	155.00	68.00	28.30	1.00	0.00
248.00	55.00	1	178.00	89.00	28.09	1.00	1.00
53.00	55.00	1	182.00	74.00	22.34	1.00	1.00
11.00	55.00	1	157.00	80.00	27.68	1.00	1.00
244.00	55.00	2	150.00	55.00	24.44	1.00	0.00
286.00	55.00	2	158.00	88.00	35.25	1.00	0.00
62.00	55.00	2	158.00	55.00	24.03	1.00	1.00
116.00	55.00	2	158.00	72.00	28.84	2.00	0.00
288.00	56.00	1	171.00	99.00	33.86	1.00	0.00
222.00	56.00	1	184.00	97.00	28.65	1.00	0.00
240.00	56.00	1	175.00	88.00	28.73	1.00	1.00
247.00	56.00	1	183.00	82.00	24.49	1.00	1.00
142.00	56.00	1	170.00	85.00	29.41	2.00	1.00
199.00	56.00	1	165.00	58.00	21.30	2.00	0.00
64.00	56.00	2	160.00	65.00	25.39	1.00	0.00
273.00	56.00	2	167.00	78.00	27.97	1.00	1.00
36.00	56.00	2	170.00	78.00	26.99	1.00	1.00
101.00	56.00	2	165.00	80.00	29.38	1.00	1.00
204.00	56.00	2	165.00	110.00	40.40	2.00	0.00
122.00	56.00	2	160.00	85.00	33.20	2.00	0.00
285.00	57.00	1	172.00	86.00	29.07	1.00	1.00
178.00	57.00	1	180.00	100.00	30.86	2.00	1.00
224.00	57.00	2	165.00	72.00	26.45	1.00	0.00
212.00	57.00	2	164.00	77.00	28.63	1.00	1.00
210.00	57.00	2	160.00	70.00	27.34	1.00	1.00
196.00	57.00	2	160.00	90.00	35.16	2.00	0.00
130.00	57.00	2	157.00	75.00	30.43	2.00	0.00
260.00	58.00	1	175.00	65.00	21.22	1.00	0.00
150.00	58.00	1	168.00	79.00	27.99	2.00	0.00
164.00	58.00	1	174.00	82.00	27.08	2.00	1.00
63.00	58.00	2	157.00	70.00	28.40	1.00	0.00
239.00	58.00	2	152.00	70.00	30.30	1.00	1.00
89.00	58.00	2	170.00	89.00	30.80	1.00	1.00
261.00	59.00	1	168.00	70.00	24.80	1.00	1.00
140.00	59.00	1	185.00	93.00	27.17	2.00	0.00
82.00	59.00	2	170.00	100.00	34.60	1.00	1.00
223.00	59.00	2	160.00	88.00	34.38	1.00	1.00
293.00	59.00	2	154.00	70.00	29.52	1.00	1.00
6.00	59.00	2	157.00	79.00	32.05	1.00	1.00
154.00	59.00	2	160.00	68.00	26.56	2.00	1.00
205.00	59.00	2	177.00	90.00	28.73	2.00	0.00
264.00	60.00	1	167.00	77.00	27.61	1.00	1.00
295.00	60.00	1	176.00	73.00	23.57	1.00	1.00
26.00	60.00	1	188.00	88.00	24.90	1.00	1.00
31.00	60.00	1	185.00	81.00	23.67	1.00	1.00
45.00	60.00	2	155.00	90.00	37.46	1.00	1.00
156.00	61.00	1	175.00	100.00	32.65	2.00	0.00
120.00	61.00	1	175.00	75.00	24.49	2.00	0.00
80.00	61.00	2	155.00	65.00	27.06	1.00	0.00
238.00	61.00	2	158.00	70.00	28.04	1.00	1.00
255.00	61.00	2	166.00	69.00	25.04	1.00	1.00

175.00	61.00	2	160.00	75.00	29.30	2.00	1.00
110.00	61.00	2	162.00	75.00	28.58	2.00	0.00
87.00	62.00	1	170.00	78.00	26.99	1.00	1.00
37.00	62.00	1	172.00	80.00	27.04	1.00	1.00
127.00	62.00	1	175.00	90.00	29.39	2.00	0.00
144.00	62.00	1	1720.00	70.00	25.24	2.00	0.00
250.00	62.00	2	150.00	52.00	23.11	1.00	0.00
146.00	62.00	2	165.00	73.00	26.81	2.00	1.00
189.00	62.00	2	160.00	68.00	26.56	2.00	1.00
252.00	63.00	1	168.00	75.00	26.57	1.00	1.00
191.00	63.00	1	170.00	80.00	27.68	2.00	0.00
131.00	63.00	1	165.00	80.00	29.38	2.00	1.00
137.00	63.00	1	169.00	84.00	29.41	2.00	1.00
74.00	63.00	2	165.00	95.00	34.89	1.00	1.00
182.00	63.00	2	157.00	67.00	27.18	2.00	1.00
108.00	63.00	2	160.00	87.00	33.98	2.00	0.00
207.00	63.00	2	165.00	51.00	18.73	2.00	0.00
117.00	63.00	2	165.00	82.00	30.12	2.00	1.00
68.00	64.00	1	170.00	86.00	29.76	1.00	1.00
32.00	64.00	1	167.00	75.00	26.89	1.00	1.00
211.00	64.00	1	160.00	88.00	34.38	1.00	1.00
203.00	64.00	1	180.00	80.00	24.69	2.00	1.00
40.00	64.00	2	156.00	95.00	39.04	1.00	0.00
269.00	64.00	2	160.00	80.00	31.25	1.00	1.00
168.00	64.00	2	150.00	70.00	31.11	2.00	0.00
165.00	64.00	2	167.00	90.00	32.27	2.00	0.00
141.00	64.00	2	165.00	80.00	29.38	2.00	1.00
75.00	65.00	1	173.00	88.00	29.40	1.00	1.00
233.00	65.00	1	171.00	80.00	27.36	1.00	1.00
147.00	65.00	1	176.00	72.00	23.24	2.00	1.00
58.00	65.00	2	160.00	55.00	21.48	1.00	0.00
18.00	65.00	2	156.00	93.00	38.21	1.00	0.00
25.00	65.00	2	155.00	68.00	28.30	1.00	1.00
46.00	65.00	2	160.00	80.00	31.25	1.00	1.00
183.00	65.00	2	168.00	62.00	21.97	2.00	1.00
115.00	65.00	2	163.00	82.00	30.86	2.00	1.00
92.00	66.00	1	175.00	75.00	24.49	1.00	1.00
69.00	66.00	1	160.00	71.00	27.73	1.00	1.00
258.00	66.00	1	157.00	50.00	20.28	1.00	1.00
225.00	66.00	1	178.00	70.00	22.09	1.00	1.00
139.00	66.00	1	168.00	80.00	28.34	2.00	1.00
163.00	66.00	1	170.00	82.00	28.37	2.00	1.00
98.00	66.00	2	161.00	62.00	23.92	1.00	1.00
267.00	66.00	2	165.00	80.00	29.38	1.00	1.00
158.00	66.00	2	155.00	56.00	23.31	2.00	1.00
145.00	66.00	2	151.00	70.00	30.70	2.00	0.00
43.00	67.00	1	168.00	79.00	21.99	1.00	0.00
206.00	67.00	1	182.00	96.00	28.98	2.00	0.00
42.00	67.00	2	156.00	58.00	23.83	1.00	0.00
99.00	67.00	2	160.00	55.00	21.48	1.00	1.00
278.00	67.00	2	152.00	46.00	19.91	1.00	1.00
172.00	67.00	2	162.00	75.00	28.58	2.00	0.00
275.00	68.00	1	170.00	80.00	27.68	1.00	1.00

9.00	68.00	2	151.00	71.00	31.14	1.00	0.00
24.00	68.00	2	163.00	59.00	22.21	1.00	1.00
33.00	68.00	2	160.00	80.00	31.25	1.00	1.00
79.00	68.00	2	160.00	80.00	31.25	1.00	1.00
169.00	68.00	2	166.00	78.00	28.31	2.00	0.00
179.00	68.00	2	162.00	80.00	30.48	2.00	1.00
15.00	69.00	1	150.00	65.00	28.89	1.00	0.00
106.00	69.00	1	182.00	89.00	26.87	2.00	0.00
7.00	69.00	2	165.00	78.00	28.65	1.00	0.00
251.00	69.00	2	164.00	81.00	30.12	1.00	1.00
72.00	69.00	2	165.00	83.00	30.49	1.00	1.00
114.00	69.00	2	169.00	67.00	23.46	2.00	1.00
198.00	69.00	2	170.00	85.00	29.41	2.00	1.00
153.00	69.00	2	168.00	72.00	25.51	2.00	0.00
133.00	69.00	2	158.00	106.00	42.46	2.00	0.00
78.00	70.00	1	168.00	78.00	27.64	1.00	0.00
10.00	70.00	1	175.00	70.00	22.86	1.00	0.00
276.00	70.00	1	173.00	69.00	23.05	1.00	1.00
83.00	70.00	1	176.00	75.00	24.21	1.00	1.00
55.00	70.00	2	160.00	90.00	35.16	1.00	0.00
44.00	70.00	2	160.00	93.00	36.33	1.00	1.00
184.00	70.00	2	165.00	74.00	27.18	2.00	0.00
262.00	71.00	1	184.00	105.00	31.01	1.00	0.00
95.00	71.00	1	175.00	73.00	23.84	1.00	1.00
173.00	71.00	1	163.00	66.00	24.84	2.00	0.00
97.00	71.00	2	159.00	65.00	25.71	1.00	1.00
81.00	71.00	2	168.00	66.00	23.38	1.00	1.00
125.00	71.00	2	155.00	80.00	33.30	2.00	1.00
90.00	72.00	1	160.00	65.00	25.39	1.00	0.00
253.00	72.00	1	160.00	62.00	24.22	1.00	1.00
85.00	72.00	1	160.00	76.00	29.69	1.00	1.00
86.00	72.00	1	172.00	70.00	23.66	1.00	1.00
230.00	73.00	1	170.00	72.00	26.91	1.00	0.00
281.00	73.00	1	168.00	95.00	33.66	1.00	1.00
94.00	73.00	1	170.00	73.00	25.26	1.00	1.00
121.00	73.00	1	165.00	67.00	24.61	2.00	1.00
284.00	74.00	2	167.00	68.00	24.38	1.00	1.00
16.00	75.00	1	167.00	73.00	26.18	1.00	1.00
112.00	75.00	1	166.00	70.00	25.40	2.00	1.00
218.00	75.00	2	150.00	51.00	22.67	1.00	1.00
35.00	75.00	2	158.00	75.00	30.04	1.00	1.00
257.00	75.00	2	162.00	60.00	22.86	1.00	1.00
61.00	75.00	2	165.00	70.00	25.71	1.00	1.00
177.00	75.00	2	165.00	90.00	33.06	2.00	0.00
234.00	76.00	1	170.00	118.00	38.83	1.00	0.00
109.00	76.00	1	168.00	78.00	27.64	2.00	0.00
56.00	76.00	2	160.00	70.00	27.34	1.00	1.00
209.00	76.00	2	155.00	81.00	33.71	2.00	1.00
105.00	77.00	1	154.00	60.00	25.30	1.00	1.00
123.00	77.00	1	165.00	60.00	22.04	2.00	0.00
162.00	78.00	1	170.00	64.00	22.15	2.00	0.00
65.00	78.00	2	170.00	68.00	23.53	1.00	0.00
49.00	78.00	2	170.00	90.00	31.14	1.00	1.00

256.00	79.00	1	176.00	79.00	35.50	1.00	0.00
54.00	79.00	1	175.00	75.00	24.49	1.00	1.00
151.00	79.00	1	165.00	80.00	29.38	2.00	1.00
192.00	80.00	1	151.00	67.00	29.38	2.00	1.00
119.00	80.00	2	10.00	70.00	27.00	2.00	0.00
73.00	81.00	2	170.00	95.00	32.87	1.00	0.00
66.00	81.00	2	160.00	65.00	25.39	1.00	1.00
13.00	81.00	2	140.00	53.00	27.04	1.00	1.00
190.00	81.00	2	158.00	87.00	34.85	2.00	0.00
155.00	82.00	1	166.00	70.00	25.40	2.00	1.00
201.00	82.00	1	165.00	68.00	24.98	2.00	1.00
188.00	82.00	1	170.00	110.00	38.06	2.00	1.00
118.00	82.00	1	180.00	90.00	27.78	2.00	0.00
103.00	82.00	2	173.00	70.00	23.39	1.00	1.00
22.00	82.00	2	156.00	61.00	25.07	1.00	1.00
132.00	82.00	2	160.00	50.00	19.53	2.00	1.00
138.00	82.00	2	150.00	96.00	42.67	2.00	1.00
170.00	83.00	1	169.00	63.00	22.06	2.00	0.00
135.00	83.00	1	165.00	70.00	25.71	2.00	0.00
96.00	83.00	2	170.00	75.00	25.95	1.00	1.00
60.00	84.00	1	170.00	80.00	27.68	1.00	1.00
128.00	85.00	1	165.00	55.00	20.20	2.00	1.00
171.00	86.00	1	172.00	70.00	23.66	2.00	0.00
30.00	86.00	2	160.00	76.00	29.69	1.00	0.00
194.00	86.00	2	156.00	70.00	28.76	2.00	1.00
186.00	86.00	2	160.00	55.00	21.48	2.00	0.00
107.00	88.00	1	12.00	90.00	25.00	2.00	1.00
48.00	89.00	2	157.00	72.00	29.21	1.00	1.00
272.00	90.00	1	158.00	60.00	24.03	1.00	1.00
149.00	91.00	1	178.00	75.00	23.67	2.00	0.00
197.00	94.00	1	169.00	79.00	27.66	2.00	0.00

SOCIAL_E	SMOKING	Marial_stat	Physical_a	Physical_a	WBC	RBC	Hgb
2.00	0.00	0.00	1590.00	2.00	12.00	5.22	14.90
3.00	1.00	0.00	700.00	2.00	9.10	5.57	16.90
1.00	1.00	0.00	5240.00	3.00	4.00	4.07	15.00
2.00	0.00	0.00	1378.00	2.00	12.20	4.44	12.30
1.00	0.00	0.00	1519.50	2.00	9.40	4.32	10.90
2.00	0.00	0.00	1393.00	2.00	7.30	4.94	14.90
1.00	0.00	0.00	1251.00	2.00	8.10	3.38	10.90
2.00	0.00	0.00	4426.00	3.00	5.50	8.00	12.20
1.00	0.00	0.00	1418.00	2.00	12.20	4.86	14.50
1.00	0.00	0.00	1560.00	2.00	6.40	4.95	14.70
1.00	0.00	0.00	940.00	2.00	4.90	4.09	11.40
2.00	0.00	0.00	1180.00	2.00	3.30	5.23	14.50
1.00	0.00	0.00	1420.00	2.00	4.70	5.09	15.20
2.00	0.00	0.00	1124.00	2.00	5.10	2.94	9.50
2.00	0.00	0.00	1308.00	2.00	8.40	3.85	11.90
2.00	0.00	0.00	1420.00	2.00	3.00	4.98	14.30
3.00	0.00	0.00	865.50	2.00	1.70	4.35	13.10
2.00	0.00	0.00	2542.00	3.00	#NULL!	#NULL!	#NULL!
3.00	0.00	0.00	597.00	1.00	7.50	3.67	10.60
1.00	1.00	0.00	1540.00	2.00	7.10	5.57	17.30
1.00	1.00	0.00	140.00	1.00	3.90	4.70	14.40
2.00	1.00	0.00	3508.00	3.00	9.30	5.53	15.50
3.00	0.00	0.00	2240.00	3.00	2.40	3.83	11.50
2.00	0.00	0.00	2146.00	3.00	4.10	3.60	11.40
3.00	0.00	0.00	580.50	1.00	14.80	4.33	13.10
3.00	0.00	0.00	1414.00	2.00	7.10	4.90	11.70
1.00	0.00	0.00	440.00	1.00	8.80	4.57	12.50
3.00	0.00	0.00	1816.00	3.00	4.80	5.59	15.90
1.00	0.00	0.00	940.00	2.00	7.80	4.53	13.30
1.00	0.00	0.00	1504.00	2.00	4.60	4.53	13.00
3.00	0.00	0.00	1360.00	2.00	5.70	4.41	13.80
2.00	0.00	0.00	848.50	2.00	3.30	5.01	14.40
2.00	0.00	0.00	713.00	2.00	5.00	5.53	16.50
3.00	0.00	0.00	460.00	1.00	7.10	5.03	14.60
2.00	0.00	0.00	1540.00	2.00	10.00	5.39	15.30
2.00	0.00	0.00	1240.00	2.00	9.30	4.82	14.10
1.00	0.00	0.00	1338.00	2.00	5.80	4.92	12.60
1.00	0.00	0.00	460.00	1.00	4.60	4.15	11.60
1.00	1.00	0.00	1006.50	2.00	9.30	4.90	14.80
1.00	1.00	0.00	2906.00	3.00	10.60	4.26	9.00
2.00	0.00	0.00	1562.00	2.00	4.00	6.35	12.20
3.00	1.00	1.00	1590.00	2.00	8.60	5.33	15.40
1.00	0.00	1.00	1079.00	2.00	4.10	4.25	13.10
1.00	0.00	1.00	550.00	1.00	10.30	4.84	13.90
3.00	0.00	1.00	1120.00	2.00	4.40	5.94	17.40
2.00	0.00	1.00	1504.00	2.00	6.70	5.21	15.80
2.00	0.00	1.00	1851.00	2.00	13.10	4.72	14.80
2.00	0.00	1.00	546.50	1.00	4.60	4.69	13.90
2.00	0.00	1.00	472.00	1.00	6.80	2.97	8.60
2.00	0.00	1.00	895.00	2.00	8.00	5.38	14.40
2.00	0.00	1.00	1590.00	2.00	7.70	4.38	12.70
2.00	0.00	1.00	1564.00	2.00	8.30	4.03	12.00

1.00	0.00	1.00	1204.00	2.00	8.00	5.23	13.80
1.00	0.00	1.00	1533.00	2.00	#NULL!	#NULL!	#NULL!
1.00	0.00	1.00	1060.00	2.00	15.40	4.66	8.20
2.00	0.00	1.00	424.00	1.00	2.80	3.93	10.30
2.00	0.00	1.00	300.00	1.00	5.70	5.32	11.60
3.00	0.00	1.00	1500.00	2.00	12.50	4.83	13.70
2.00	0.00	1.00	1240.00	2.00	3.90	4.65	13.70
3.00	0.00	1.00	1478.00	2.00	6.90	4.63	12.10
2.00	0.00	1.00	1840.00	3.00	7.90	4.04	11.60
2.00	0.00	1.00	1420.00	2.00	6.80	4.87	15.10
2.00	0.00	1.00	1260.00	2.00	5.80	6.26	18.50
2.00	1.00	1.00	4354.00	3.00	6.60	5.96	17.90
1.00	0.00	1.00	938.00	2.00	4.00	4.35	13.10
3.00	0.00	1.00	1300.00	2.00	6.90	4.56	14.90
1.00	0.00	1.00	1268.50	2.00	5.00	4.33	13.00
2.00	0.00	1.00	940.00	2.00	12.40	4.17	11.60
2.00	0.00	1.00	1138.00	2.00	5.20	4.89	14.30
1.00	0.00	1.00	567.00	1.00	8.90	4.62	14.50
1.00	1.00	1.00	2330.00	3.00	2.40	4.79	12.10
2.00	0.00	1.00	397.00	1.00	3.00	5.28	16.20
2.00	0.00	1.00	1556.00	2.00	7.20	4.38	13.30
2.00	0.00	1.00	1472.00	2.00	2.60	5.11	15.50
1.00	0.00	1.00	1294.00	2.00	6.40	4.33	12.30
2.00	0.00	1.00	1526.00	2.00	5.40	4.47	11.30
2.00	0.00	1.00	1397.00	2.00	18.20	4.29	8.70
2.00	0.00	1.00	496.00	1.00	4.90	5.46	15.10
2.00	0.00	1.00	1128.00	2.00	9.40	4.60	15.00
2.00	1.00	1.00	1393.00	2.00	5.10	1.57	5.40
1.00	1.00	1.00	1128.00	2.00	4.90	5.10	14.20
3.00	0.00	1.00	1446.00	2.00	7.50	5.84	17.80
2.00	0.00	1.00	1180.00	2.00	8.50	4.86	14.90
1.00	0.00	1.00	1540.00	2.00	4.50	4.75	13.80
2.00	0.00	1.00	1458.00	2.00	5.20	4.89	13.70
3.00	0.00	1.00	848.50	2.00	9.40	4.44	12.70
2.00	0.00	1.00	347.50	1.00	9.40	4.75	12.00
2.00	0.00	1.00	358.00	1.00	11.90	4.89	14.20
1.00	1.00	1.00	1420.00	2.00	#NULL!	#NULL!	#NULL!
2.00	0.00	1.00	546.50	1.00	7.70	5.15	14.40
2.00	0.00	1.00	2086.00	3.00	3.90	5.25	16.00
3.00	0.00	1.00	460.00	1.00	5.60	4.12	11.50
1.00	0.00	1.00	568.00	1.00	8.70	4.90	15.50
1.00	0.00	1.00	340.00	1.00	3.00	4.65	13.50
3.00	0.00	1.00	353.20	1.00	7.60	4.02	13.90
3.00	0.00	1.00	536.00	1.00	9.00	5.30	15.90
2.00	0.00	1.00	460.00	1.00	8.70	4.82	12.60
1.00	0.00	1.00	446.50	1.00	#NULL!	#NULL!	#NULL!
1.00	0.00	1.00	1180.00	2.00	7.90	4.91	12.20
3.00	0.00	1.00	2608.00	3.00	14.60	3.72	11.80
1.00	0.00	1.00	524.00	1.00	4.00	2.88	7.80
2.00	0.00	1.00	204.00	1.00	5.80	4.21	12.60
2.00	0.00	1.00	820.00	2.00	4.90	4.75	13.50
2.00	0.00	1.00	520.00	1.00	8.30	3.72	11.10
2.00	0.00	1.00	446.50	1.00	4.30	4.34	13.40

2.00	0.00	1.00	397.00	1.00	7.20	4.61	13.80
1.00	0.00	1.00	1040.00	2.00	7.70	4.42	12.30
1.00	1.00	1.00	1216.00	2.00	17.30	4.37	14.70
2.00	0.00	1.00	1180.00	2.00	8.80	5.10	14.10
2.00	0.00	1.00	2066.00	3.00	4.80	4.38	12.80
3.00	0.00	1.00	1294.00	2.00	8.50	5.95	16.20
1.00	0.00	1.00	1374.00	2.00	5.70	5.16	13.20
3.00	0.00	1.00	984.00	2.00	4.00	4.63	14.40
2.00	0.00	1.00	1482.00	2.00	11.50	4.85	14.50
1.00	0.00	1.00	1060.00	2.00	5.20	6.08	10.40
1.00	0.00	1.00	340.00	1.00	5.60	4.60	13.20
1.00	1.00	1.00	1060.00	2.00	3.40	4.73	15.10
3.00	0.00	1.00	460.00	1.00	4.30	6.05	11.40
2.00	0.00	1.00	1282.00	2.00	8.80	5.36	14.50
3.00	0.00	1.00	964.00	2.00	17.50	5.71	15.80
1.00	1.00	1.00	1426.00	2.00	13.00	5.01	15.30
1.00	0.00	1.00	1014.00	2.00	6.90	4.33	11.00
2.00	0.00	1.00	1180.00	2.00	11.30	4.91	14.10
2.00	0.00	1.00	1456.00	2.00	5.40	4.86	12.80
3.00	0.00	1.00	490.50	1.00	5.10	3.98	11.60
2.00	0.00	1.00	1460.00	2.00	9.30	4.96	16.40
1.00	0.00	1.00	340.00	1.00	12.60	4.43	12.70
2.00	0.00	1.00	1060.00	2.00	7.80	4.69	14.30
3.00	1.00	1.00	460.00	1.00	3.60	4.38	12.70
1.00	0.00	1.00	1414.00	2.00	4.20	6.93	18.70
2.00	0.00	1.00	1072.00	2.00	4.00	4.21	11.90
3.00	0.00	0.00	1315.00	2.00	6.20	4.84	13.40
1.00	0.00	1.00	232.00	1.00	3.10	5.10	15.00
1.00	0.00	1.00	780.00	2.00	11.90	4.51	14.30
3.00	0.00	1.00	3523.00	3.00	5.10	4.43	13.00
1.00	1.00	1.00	1180.00	2.00	9.10	5.37	16.40
1.00	1.00	0.00	1420.00	2.00	4.10	3.57	10.80
1.00	0.00	1.00	1180.00	2.00	8.40	5.30	16.10
2.00	0.00	1.00	832.00	2.00	8.00	4.97	10.30
2.00	0.00	1.00	460.00	1.00	5.70	3.96	11.50
2.00	0.00	1.00	1140.00	2.00	5.30	4.73	13.80
1.00	0.00	1.00	1420.00	2.00	9.20	4.31	12.50
2.00	0.00	1.00	338.00	1.00	7.00	3.55	10.30
2.00	0.00	1.00	298.00	1.00	11.60	4.36	12.20
3.00	0.00	1.00	940.00	2.00	11.10	5.06	12.80
3.00	0.00	1.00	397.00	1.00	10.10	6.05	15.70
3.00	0.00	1.00	1537.00	2.00	5.50	4.44	15.10
1.00	0.00	1.00	1120.00	2.00	13.70	5.38	13.80
2.00	1.00	1.00	1294.00	2.00	17.00	4.25	12.60
2.00	0.00	1.00	1346.00	2.00	5.60	4.25	15.50
2.00	0.00	1.00	2086.00	3.00	10.60	4.90	14.40
3.00	0.00	1.00	1133.00	2.00	8.50	5.00	16.90
3.00	0.00	1.00	496.00	1.00	6.10	5.34	11.00
2.00	1.00	1.00	397.00	1.00	12.00	4.28	12.70
2.00	0.00	0.00	1233.00	2.00	6.50	4.83	13.60
1.00	0.00	1.00	1420.00	2.00	1.80	4.83	9.30
2.00	0.00	1.00	1198.50	2.00	3.90	4.60	12.90
2.00	0.00	1.00	1020.00	2.00	5.30	3.90	12.50

3.00	0.00	0.00	997.00	2.00	12.30	3.76	11.10
1.00	0.00	1.00	524.00	1.00	10.60	4.58	11.00
3.00	1.00	0.00	1444.00	2.00	4.70	5.40	14.60
2.00	0.00	1.00	1420.00	2.00	3.80	5.84	16.70
2.00	0.00	1.00	997.00	2.00	12.40	4.69	14.30
1.00	1.00	1.00	900.00	2.00	9.20	5.23	15.70
1.00	0.00	1.00	1572.00	2.00	5.60	4.98	14.70
3.00	0.00	1.00	526.50	1.00	12.10	4.39	13.90
3.00	0.00	1.00	1380.00	2.00	8.90	4.80	12.60
3.00	1.00	1.00	1576.00	2.00	15.80	0.60	13.40
1.00	0.00	1.00	1240.00	2.00	5.40	5.45	16.80
1.00	0.00	1.00	997.00	2.00	5.50	5.70	16.20
1.00	1.00	1.00	953.20	2.00	6.00	4.32	14.20
2.00	0.00	1.00	248.50	1.00	5.90	5.06	12.20
3.00	0.00	1.00	1378.00	2.00	#NULL!	#NULL!	#NULL!
1.00	0.00	0.00	340.00	1.00	4.00	5.01	14.30
1.00	0.00	1.00	1452.00	2.00	4.30	4.32	11.20
1.00	0.00	1.00	839.00	2.00	5.80	4.64	13.20
2.00	0.00	1.00	898.00	2.00	5.90	4.86	15.90
2.00	1.00	1.00	1477.00	2.00	5.40	4.83	14.70
2.00	0.00	1.00	298.00	1.00	5.50	4.19	12.60
1.00	0.00	1.00	2113.00	3.00	7.40	4.33	12.60
1.00	0.00	1.00	567.00	1.00	8.60	4.55	13.00
2.00	0.00	1.00	446.50	1.00	11.90	4.99	12.20
2.00	0.00	1.00	460.00	1.00	4.20	4.63	13.80
1.00	0.00	1.00	446.50	1.00	5.30	4.97	14.50
1.00	0.00	0.00	464.00	1.00	6.00	4.99	14.30
2.00	0.00	1.00	997.00	2.00	7.20	4.55	14.20
3.00	0.00	1.00	1300.00	2.00	9.80	5.07	14.70
3.00	1.00	1.00	1653.00	3.00	21.60	4.35	14.40
1.00	0.00	1.00	3866.00	3.00	6.30	3.93	12.30
1.00	0.00	1.00	472.00	1.00	#NULL!	#NULL!	#NULL!
2.00	0.00	1.00	1159.00	2.00	5.60	4.16	11.90
2.00	0.00	1.00	460.00	1.00	6.60	4.45	13.20
3.00	0.00	1.00	1172.00	2.00	8.00	5.87	16.90
1.00	0.00	1.00	420.00	1.00	4.50	3.86	11.00
2.00	0.00	1.00	1237.00	2.00	6.10	5.16	14.80
2.00	0.00	1.00	1240.00	2.00	10.40	4.85	15.30
3.00	0.00	0.00	1413.00	2.00	6.10	3.67	10.00
3.00	0.00	1.00	3236.00	3.00	9.80	5.12	15.20
1.00	0.00	1.00	1145.50	2.00	13.60	5.15	15.00
1.00	0.00	1.00	1137.20	2.00	5.40	5.24	14.60
2.00	0.00	1.00	2606.00	3.00	3.50	3.72	11.20
2.00	0.00	1.00	997.00	2.00	6.70	4.46	12.40
3.00	0.00	1.00	1586.00	2.00	8.30	3.93	11.60
1.00	0.00	1.00	460.00	1.00	11.60	4.06	11.60
1.00	0.00	1.00	3613.00	3.00	5.30	5.08	15.40
1.00	0.00	1.00	1060.00	2.00	18.10	4.16	12.00
3.00	0.00	1.00	959.50	2.00	11.00	4.09	11.80
3.00	0.00	1.00	3502.00	3.00	10.20	3.73	11.70
3.00	0.00	1.00	3862.00	3.00	9.20	4.58	14.40
1.00	0.00	1.00	1136.00	2.00	7.30	3.59	10.30
3.00	0.00	0.00	1360.00	2.00	9.90	4.70	13.60

1.00	0.00	1.00	420.00	1.00	5.90	4.51	12.10
2.00	0.00	1.00	2591.00	3.00	3.90	4.09	11.80
3.00	0.00	1.00	397.00	1.00	3.80	4.39	14.40
2.00	0.00	0.00	446.50	1.00	5.50	4.41	13.30
1.00	0.00	1.00	1180.00	2.00	7.00	4.98	142.00
1.00	0.00	1.00	700.00	2.00	13.30	4.36	12.30
1.00	0.00	1.00	1060.00	2.00	3.40	4.80	13.30
1.00	0.00	0.00	1492.00	2.00	4.60	5.17	15.80
1.00	0.00	1.00	1096.00	2.00	#NULL!	#NULL!	#NULL!
3.00	0.00	1.00	940.00	2.00	4.00	4.96	13.00
2.00	0.00	1.00	1436.00	2.00	3.20	3.95	9.70
2.00	0.00	1.00	1520.00	2.00	4.70	4.67	12.00
2.00	0.00	1.00	947.50	2.00	9.80	4.07	11.70
1.00	0.00	1.00	1116.00	2.00	3.30	4.09	11.40
1.00	0.00	1.00	460.00	1.00	18.90	5.74	15.70
1.00	0.00	1.00	1294.00	2.00	#NULL!	#NULL!	#NULL!
1.00	0.00	0.00	2028.00	3.00	5.40	5.12	13.50
2.00	0.00	1.00	1826.00	3.00	4.40	4.70	14.00
2.00	0.00	0.00	1551.00	2.00	5.20	5.21	13.80
1.00	0.00	1.00	239.00	1.00	8.90	4.07	12.40
2.00	0.00	1.00	524.00	1.00	4.20	4.25	12.40
2.00	0.00	1.00	518.00	1.00	3.50	4.34	12.40
1.00	0.00	1.00	1460.00	2.00	4.80	4.69	13.60
3.00	0.00	0.00	1950.00	3.00	7.90	2.97	9.20
1.00	1.00	1.00	1098.00	2.00	4.30	4.42	12.30
2.00	0.00	1.00	1580.00	2.00	8.70	4.92	13.20
2.00	0.00	1.00	1571.00	2.00	26.80	4.56	12.40
1.00	0.00	1.00	340.00	1.00	3.80	3.18	9.60
1.00	0.00	0.00	1894.00	3.00	3.00	5.13	14.70
2.00	0.00	1.00	1446.00	2.00	6.50	4.17	13.20
2.00	0.00	1.00	940.00	2.00	5.30	4.67	16.20
3.00	0.00	1.00	1466.00	2.00	9.80	5.66	17.10
1.00	1.00	0.00	1456.00	2.00	2.90	4.42	13.60
2.00	0.00	1.00	340.00	1.00	4.00	4.96	14.10
2.00	0.00	1.00	900.00	2.00	4.20	4.55	14.00
2.00	0.00	1.00	1128.00	2.00	6.80	5.54	15.10
3.00	0.00	1.00	1368.00	2.00	3.30	3.92	7.70
2.00	0.00	1.00	1524.00	2.00	#NULL!	#NULL!	#NULL!
3.00	0.00	1.00	1588.00	2.00	5.10	5.59	14.30
2.00	0.00	1.00	2066.00	3.00	8.30	4.48	12.50
3.00	0.00	0.00	898.00	2.00	2.40	3.75	11.80
3.00	0.00	0.00	1994.00	3.00	5.90	4.19	12.10
3.00	0.00	1.00	1113.00	2.00	4.10	4.30	13.00
1.00	0.00	1.00	588.00	1.00	10.90	4.57	13.60
1.00	0.00	1.00	460.00	1.00	2.90	5.61	16.60
1.00	0.00	0.00	1295.00	2.00	7.90	4.39	15.30
3.00	0.00	1.00	1300.00	2.00	7.50	4.14	12.60
1.00	0.00	1.00	340.00	1.00	7.50	4.32	12.70
3.00	1.00	0.00	578.00	1.00	9.80	5.41	15.80
1.00	1.00	1.00	1482.00	2.00	8.40	5.07	16.50
1.00	0.00	1.00	2954.00	3.00	#NULL!	#NULL!	#NULL!
3.00	0.00	1.00	1226.00	2.00	4.70	5.13	14.10
2.00	0.00	1.00	424.00	1.00	3.80	5.33	15.80

1.00	0.00	1.00	248.50	1.00	6.90	4.75	14.80
2.00	0.00	0.00	1140.00	2.00	5.80	5.20	16.40
1.00	0.00	1.00	997.00	2.00	3.10	4.68	14.60
2.00	0.00	1.00	997.00	2.00	6.30	4.40	14.60
2.00	0.00	1.00	511.00	1.00	6.50	4.95	14.60
1.00	0.00	1.00	364.00	1.00	8.30	3.88	13.00
2.00	0.00	0.00	1894.00	3.00	11.40	3.54	8.40
3.00	0.00	1.00	1420.00	2.00	13.00	4.95	14.60
2.00	0.00	1.00	683.00	2.00	#NULL!	#NULL!	#NULL!
2.00	0.00	1.00	1835.00	3.00	19.70	4.93	14.40
3.00	0.00	1.00	2086.00	3.00	25.20	4.98	15.90
3.00	1.00	1.00	1520.00	2.00	12.10	4.47	14.90
1.00	0.00	0.00	1240.00	2.00	11.50	3.66	10.70
2.00	0.00	1.00	1514.00	2.00	6.40	4.67	13.00
3.00	0.00	1.00	900.00	2.00	7.20	4.16	13.20
3.00	0.00	1.00	1566.00	2.00	4.50	4.48	9.20
2.00	0.00	1.00	460.00	1.00	14.00	4.44	13.50
2.00	0.00	1.00	3320.00	3.00	5.40	4.58	14.40
1.00	0.00	1.00	1559.00	2.00	6.20	5.52	16.60
2.00	0.00	0.00	1690.00	3.00	11.00	3.84	11.30
2.00	0.00	1.00	1285.00	2.00	6.90	3.68	10.80
3.00	0.00	1.00	3853.00	3.00	12.00	2.94	8.30
2.00	1.00	1.00	2160.00	3.00	8.30	5.02	15.60
3.00	0.00	1.00	540.00	1.00	17.90	4.43	12.90
3.00	0.00	1.00	1060.00	2.00	7.00	4.28	12.90
1.00	0.00	0.00	1152.00	2.00	8.10	4.72	14.60
3.00	1.00	1.00	1086.00	2.00	14.00	3.85	11.70
2.00	0.00	1.00	1060.00	2.00	#NULL!	4.77	13.40
2.00	0.00	1.00	1530.00	2.00	7.40	4.93	16.90
1.00	0.00	1.00	1125.50	2.00	5.60	5.30	16.90
1.00	0.00	1.00	1354.00	2.00	4.00	4.89	16.60

HCT	MCV	MCH	MCHC	Plat	RDW	MDV	MPV
43.90	84.10	28.50	33.90	144.00	12.50	#NULL!	11.10
47.10	84.60	30.30	35.90	196.00	12.80	#NULL!	9.40
34.60	85.00	36.90	43.40	123.00	12.10	#NULL!	11.40
34.90	78.60	27.70	35.20	151.00	13.50	#NULL!	10.20
34.20	79.20	25.20	31.90	445.00	14.10	#NULL!	9.50
42.20	85.40	30.20	35.30	155.00	12.20	#NULL!	11.30
32.00	94.70	32.20	34.10	187.00	12.90	#NULL!	9.90
34.00	83.30	29.90	35.90	361.00	13.50	#NULL!	8.80
41.40	85.20	29.80	35.00	211.00	14.40	#NULL!	11.90
42.80	86.50	29.70	34.30	124.00	12.80	#NULL!	10.70
35.40	86.60	27.90	32.20	174.00	13.10	#NULL!	9.70
41.60	79.50	27.70	34.90	144.00	12.50	#NULL!	10.30
43.40	85.30	29.90	35.00	142.00	12.80	#NULL!	10.80
25.40	86.40	32.30	37.40	150.00	#NULL!	#NULL!	#NULL!
33.90	88.10	30.90	35.10	183.00	#NULL!	#NULL!	#NULL!
40.30	80.90	28.70	35.50	117.00	12.60	#NULL!	9.90
37.60	86.40	30.10	34.80	72.00	12.30	#NULL!	11.50
#NULL!	#NULL!	#NULL!	#NULL!	#NULL!	#NULL!	#NULL!	#NULL!
31.00	84.50	28.90	34.20	185.00	13.70	#NULL!	9.50
49.30	88.50	31.10	35.10	249.00	13.60	#NULL!	9.90
42.80	91.10	30.60	33.60	131.00	12.90	#NULL!	10.90
44.50	80.50	28.00	34.80	234.00	13.90	#NULL!	10.10
35.00	91.40	30.00	32.90	145.00	12.60	#NULL!	12.10
34.10	94.70	31.70	33.40	463.00	#NULL!	#NULL!	#NULL!
39.00	90.10	30.30	33.60	263.00	13.70	#NULL!	10.30
38.30	78.20	23.90	30.50	200.00	16.80	#NULL!	10.30
36.60	80.10	27.40	34.20	204.00	17.70	#NULL!	10.40
44.50	79.60	28.40	35.70	118.00	12.70	#NULL!	12.20
39.10	86.30	29.40	34.00	194.00	12.80	#NULL!	11.50
39.40	87.00	28.70	33.00	241.00	#NULL!	#NULL!	#NULL!
40.10	90.90	31.30	34.40	88.00	13.50	#NULL!	11.30
41.00	81.80	28.70	35.10	105.00	#NULL!	#NULL!	#NULL!
47.10	85.20	29.80	35.00	153.00	14.30	#NULL!	8.20
3.50	86.50	29.00	33.60	181.00	12.10	#NULL!	10.00
44.70	82.90	28.40	34.20	171.00	13.40	#NULL!	9.40
41.20	85.50	29.30	34.20	163.00	12.00	#NULL!	10.00
37.00	75.20	25.60	3.10	137.00	12.40	#NULL!	12.10
35.40	85.30	28.00	32.80	347.00	14.50	#NULL!	8.20
41.60	84.90	30.20	35.60	235.00	12.50	#NULL!	8.70
31.20	73.20	21.10	28.80	252.00	13.90	#NULL!	9.20
37.80	59.50	19.20	32.30	210.00	16.80	#NULL!	9.60
42.40	79.50	28.90	36.30	161.00	13.90	#NULL!	11.70
38.20	89.90	30.80	34.30	186.00	13.70	#NULL!	10.30
40.30	83.30	28.70	34.50	233.00	12.80	#NULL!	9.80
49.20	82.80	29.30	35.40	117.00	14.80	#NULL!	10.20
45.40	87.10	30.30	34.80	224.00	12.70	#NULL!	9.90
43.70	92.60	31.40	33.90	275.00	14.20	#NULL!	11.90
38.00	82.90	29.60	35.70	140.00	12.60	#NULL!	9.00
27.60	92.90	29.00	31.20	31.00	19.70	#NULL!	#NULL!
44.10	82.00	26.80	32.70	385.00	12.70	#NULL!	9.90
36.50	83.30	29.00	34.80	280.00	12.90	#NULL!	9.60
34.80	86.40	29.80	34.50	182.00	12.90	#NULL!	10.90

40.80	78.00	26.40	33.80	176.00	13.40	#NULL!	11.00
#NULL!	#NULL!	#NULL!	#NULL!	#NULL!	#NULL!	#NULL!	#NULL!
28.60	61.40	17.60	28.70	395.00	20.80	#NULL!	9.40
31.30	79.60	26.20	32.90	134.00	14.20	#NULL!	9.00
37.00	69.50	21.80	31.40	339.00	16.40	#NULL!	8.70
43.30	89.60	28.40	31.60	167.00	12.50	#NULL!	15.10
38.80	83.40	29.50	35.30	345.00	12.40	#NULL!	9.30
36.20	78.20	26.10	33.40	140.00	13.00	#NULL!	9.40
33.60	83.20	28.70	34.50	170.00	13.80	#NULL!	10.60
40.50	83.20	31.00	37.30	208.00	12.90	#NULL!	8.90
53.40	85.30	29.60	34.60	195.00	13.10	#NULL!	8.90
50.00	83.90	30.00	35.80	198.00	12.50	#NULL!	10.00
37.80	86.90	30.10	34.70	161.00	12.30	#NULL!	11.00
41.50	91.00	32.70	35.90	135.00	12.90	#NULL!	11.20
38.10	88.00	30.00	34.10	149.00	13.30	#NULL!	12.30
35.70	85.60	27.80	32.50	192.00	14.00	#NULL!	10.70
40.30	82.40	29.20	35.50	94.00	12.70	#NULL!	10.90
39.50	85.50	31.40	36.70	119.00	13.00	#NULL!	11.00
37.40	78.10	25.30	32.40	130.00	14.30	#NULL!	9.70
46.30	87.70	30.70	35.00	106.00	14.10	#NULL!	10.70
38.90	88.80	30.40	34.20	171.00	#NULL!	#NULL!	#NULL!
43.40	84.90	30.30	35.70	110.00	12.30	#NULL!	10.90
35.50	82.00	28.40	34.60	134.00	13.00	#NULL!	10.60
36.00	80.50	25.30	31.40	228.00	19.90	#NULL!	11.00
28.60	66.70	20.30	3.40	390.00	20.30	#NULL!	10.10
45.00	82.40	27.70	33.60	180.00	13.20	#NULL!	10.00
43.70	95.00	32.60	34.30	134.00	13.10	#NULL!	11.00
15.70	100.00	34.40	34.40	11.00	16.00	#NULL!	#NULL!
42.20	82.70	27.80	33.30	170.00	13.20	#NULL!	10.80
50.50	86.50	30.50	35.20	238.00	13.80	#NULL!	10.90
41.20	84.80	30.70	36.20	210.00	12.70	#NULL!	8.70
41.10	86.50	29.10	33.60	253.00	11.60	#NULL!	10.10
39.90	81.60	28.00	34.30	237.00	13.30	#NULL!	9.30
37.00	83.30	28.60	34.30	276.00	13.20	#NULL!	10.30
35.70	75.20	25.30	33.60	158.00	15.60	#NULL!	11.00
40.50	82.80	29.00	35.10	263.00	14.70	#NULL!	9.80
#NULL!	#NULL!	#NULL!	#NULL!	#NULL!	#NULL!	#NULL!	#NULL!
41.60	80.80	28.00	34.60	235.00	13.40	#NULL!	9.40
43.10	82.10	30.50	37.10	171.00	13.00	#NULL!	11.10
35.40	85.90	27.90	32.50	190.00	12.50	#NULL!	10.70
43.30	88.40	31.60	35.80	338.00	12.70	#NULL!	9.10
38.40	82.60	29.00	35.20	147.00	12.80	#NULL!	10.20
38.50	95.80	34.60	36.10	225.00	13.60	#NULL!	9.50
46.20	87.20	30.00	34.40	241.00	#NULL!	#NULL!	#NULL!
39.70	82.40	26.10	31.70	184.00	12.90	#NULL!	10.50
#NULL!	#NULL!	#NULL!	#NULL!	#NULL!	#NULL!	#NULL!	#NULL!
37.30	76.00	24.80	32.70	181.00	17.00	#NULL!	12.00
34.00	91.40	31.70	34.70	293.00	21.50	#NULL!	8.90
23.70	82.30	27.10	32.90	173.00	15.50	#NULL!	8.70
37.80	89.80	29.90	33.30	141.00	13.90	#NULL!	11.70
38.50	81.10	28.40	35.10	409.00	13.40	#NULL!	10.00
33.70	90.60	29.80	32.90	41.00	#NULL!	#NULL!	#NULL!
38.10	87.80	30.90	35.20	176.00	12.10	#NULL!	9.70

38.60	83.70	29.90	35.80	277.00	13.90	#NULL!	10.00
36.10	81.70	27.80	34.10	167.00	14.70	#NULL!	9.90
38.90	89.00	33.60	37.80	207.00	13.60	#NULL!	11.10
42.20	82.70	27.60	33.40	205.00	13.20	#NULL!	9.20
38.90	88.80	29.20	32.90	107.00	13.10	#NULL!	10.50
47.90	80.50	27.20	33.80	274.00	14.40	#NULL!	11.10
39.60	76.70	25.60	33.30	248.00	14.90	#NULL!	11.20
39.80	86.00	31.10	36.20	122.00	12.00	#NULL!	9.80
42.50	87.60	29.90	34.10	148.00	13.50	#NULL!	11.30
35.30	58.10	17.10	29.50	225.00	16.50	#NULL!	#NULL!
38.00	82.60	28.70	3.70	113.00	12.70	#NULL!	11.60
42.00	88.80	31.90	36.00	124.00	11.60	#NULL!	9.60
36.50	60.30	18.80	31.20	187.00	16.20	#NULL!	#NULL!
44.70	83.40	28.20	33.80	236.00	12.70	#NULL!	9.70
46.40	81.30	27.70	34.10	151.00	14.20	#NULL!	9.30
42.20	84.20	30.50	36.30	198.00	13.60	#NULL!	10.90
32.00	73.90	25.40	34.40	276.00	16.40	#NULL!	8.50
40.20	81.90	28.70	35.10	316.00	12.10	#NULL!	8.80
40.40	83.10	26.30	31.70	198.00	14.00	#NULL!	9.30
35.20	88.40	29.10	33.00	245.00	15.40	#NULL!	10.60
46.20	93.10	33.10	35.50	178.00	13.30	#NULL!	10.90
37.40	84.40	28.70	34.00	180.00	16.10	#NULL!	11.30
40.70	86.80	30.50	35.10	273.00	13.30	#NULL!	9.60
37.90	86.50	29.00	33.50	110.00	13.10	#NULL!	10.70
54.90	79.20	27.00	34.10	84.00	15.50	#NULL!	11.90
35.90	85.30	28.30	33.10	108.00	13.20	#NULL!	10.80
39.60	81.80	27.70	33.80	234.00	13.10	#NULL!	9.80
45.30	88.80	29.40	33.10	144.00	13.10	#NULL!	9.20
39.50	87.60	31.70	36.20	324.00	13.70	#NULL!	8.80
36.20	81.70	29.30	35.90	140.00	15.40	#NULL!	11.60
46.40	86.40	30.50	35.30	140.00	12.70	#NULL!	#NULL!
33.40	93.60	30.30	32.30	124.00	14.90	#NULL!	9.30
45.60	86.00	30.40	35.30	214.00	13.10	#NULL!	9.20
33.80	68.00	20.70	30.50	262.00	15.40	#NULL!	#NULL!
34.10	86.10	29.00	33.70	178.00	12.50	#NULL!	10.90
40.10	84.80	29.20	34.40	200.00	13.10	#NULL!	9.60
36.00	83.50	29.00	34.70	185.00	14.10	#NULL!	9.20
30.30	85.40	29.00	34.00	239.00	19.40	#NULL!	9.30
35.90	82.30	28.00	34.00	200.00	13.70	#NULL!	11.10
38.60	76.30	25.30	33.20	340.00	14.80	#NULL!	10.10
45.80	75.70	26.00	34.30	191.00	16.40	#NULL!	11.60
41.40	93.20	34.00	36.50	158.00	12.20	#NULL!	10.70
43.30	80.50	25.70	31.90	182.00	25.70	#NULL!	#NULL!
35.80	84.20	29.60	35.20	168.00	14.40	#NULL!	11.40
37.50	88.20	36.50	41.30	152.00	12.30	#NULL!	10.40
41.50	84.70	29.40	34.70	258.00	12.40	#NULL!	9.90
48.00	95.00	33.50	35.20	167.00	#NULL!	#NULL!	#NULL!
34.10	63.90	20.60	32.30	217.00	15.90	#NULL!	9.80
34.80	81.30	29.70	36.50	182.00	12.90	#NULL!	10.10
39.20	81.20	28.20	34.70	301.00	13.50	#NULL!	10.10
29.50	61.10	19.30	31.50	172.00	15.10	#NULL!	#NULL!
37.10	80.70	28.00	34.80	119.00	12.80	#NULL!	104.00
34.70	89.00	32.10	36.00	174.00	11.90	#NULL!	10.10

33.50	89.10	29.50	33.10	246.00	15.50	#NULL!	10.50
33.60	73.40	24.00	32.70	237.00	18.40	#NULL!	1.80
43.80	81.10	27.00	33.30	177.00	13.00	#NULL!	9.70
47.30	81.00	28.60	35.30	99.00	13.10	#NULL!	11.00
41.20	87.80	30.50	34.70	183.00	14.50	#NULL!	10.80
44.30	84.70	30.00	35.40	201.00	13.40	#NULL!	10.70
43.70	87.80	29.50	33.60	229.00	13.90	#NULL!	10.70
38.40	87.50	31.70	36.20	221.00	13.20	#NULL!	9.50
36.90	76.90	26.30	34.10	437.00	13.70	#NULL!	9.60
34.50	75.00	29.10	38.80	339.00	15.00	#NULL!	9.50
51.80	95.00	30.80	32.40	88.00	13.50	#NULL!	12.50
46.90	82.30	28.40	34.50	173.00	13.80	#NULL!	10.70
39.60	91.70	32.90	35.90	170.00	13.80	#NULL!	9.60
39.80	78.70	24.10	30.70	225.00	15.80	#NULL!	9.50
#NULL!	#NULL!	#NULL!	#NULL!	#NULL!	#NULL!	#NULL!	#NULL!
41.10	82.00	28.50	34.80	143.00	14.30	#NULL!	10.80
33.70	78.00	25.90	33.20	146.00	17.60	#NULL!	10.20
37.60	81.00	28.40	35.10	208.00	#NULL!	#NULL!	#NULL!
43.20	88.90	32.70	36.80	114.00	12.00	#NULL!	10.30
39.40	81.60	30.40	37.30	155.00	12.60	#NULL!	10.30
36.90	88.10	30.10	34.10	292.00	13.60	#NULL!	9.10
36.70	84.80	29.10	34.30	130.00	14.20	#NULL!	11.60
37.50	82.40	28.60	34.70	227.00	13.60	#NULL!	10.70
38.00	76.20	24.40	32.10	510.00	14.00	#NULL!	9.00
39.30	84.90	29.80	35.10	185.00	13.00	#NULL!	10.10
41.30	83.10	29.20	35.10	164.00	#NULL!	#NULL!	#NULL!
44.70	89.60	28.70	32.00	155.00	18.90	#NULL!	9.60
41.00	90.10	31.20	34.60	235.00	13.30	#NULL!	9.90
43.80	86.40	29.00	33.60	259.00	13.10	#NULL!	9.90
40.50	93.10	33.10	35.60	166.00	13.70	#NULL!	12.40
35.50	90.30	31.30	34.60	307.00	15.10	#NULL!	11.60
#NULL!	#NULL!	#NULL!	#NULL!	#NULL!	#NULL!	#NULL!	#NULL!
34.00	81.70	28.60	35.00	253.00	#NULL!	#NULL!	#NULL!
39.60	89.00	29.70	33.30	155.00	12.80	#NULL!	10.10
48.30	82.30	28.80	35.00	210.00	14.30	#NULL!	10.30
32.60	84.50	28.50	33.70	271.00	14.10	#NULL!	9.30
42.90	83.10	28.70	34.50	128.00	13.10	#NULL!	11.40
43.90	90.50	31.50	34.90	141.00	13.70	#NULL!	10.70
31.70	86.40	27.20	31.50	148.00	14.40	#NULL!	8.60
42.90	83.80	29.70	35.40	315.00	12.50	#NULL!	10.80
43.40	84.30	29.10	34.60	220.00	13.80	#NULL!	9.60
49.90	95.20	27.90	29.30	301.00	19.40	#NULL!	9.20
32.20	86.60	30.10	34.80	136.00	13.00	#NULL!	10.20
36.20	81.20	27.80	34.30	167.00	13.40	#NULL!	10.20
36.40	92.60	29.50	31.90	177.00	15.70	#NULL!	10.50
34.50	85.00	28.60	33.60	183.00	13.70	#NULL!	11.00
44.20	87.00	30.30	34.80	273.00	13.00	12.60	10.20
35.40	85.10	28.80	33.90	153.00	12.50	#NULL!	11.00
34.10	83.40	28.90	34.60	161.00	13.70	#NULL!	10.70
33.80	90.60	31.40	34.60	179.00	11.80	#NULL!	10.50
42.20	92.10	31.40	34.10	321.00	14.10	#NULL!	11.40
32.60	90.80	28.70	31.60	164.00	#NULL!	#NULL!	#NULL!
38.80	82.60	28.90	35.10	266.00	13.00	#NULL!	9.70

37.50	83.10	26.80	32.30	192.00	12.40	#NULL!	9.70
32.30	79.00	28.90	36.50	147.00	13.50	#NULL!	9.80
41.20	93.80	32.80	35.00	166.00	13.50	#NULL!	9.50
37.90	85.90	30.20	35.10	246.00	13.30	#NULL!	10.40
42.80	85.90	28.50	33.20	240.00	13.10	#NULL!	10.80
36.60	83.90	28.20	33.60	200.00	13.20	#NULL!	10.50
37.30	77.70	27.70	35.70	163.00	13.50	#NULL!	11.80
44.00	85.10	30.60	35.90	110.00	13.20	#NULL!	11.00
#NULL!	#NULL!	#NULL!	#NULL!	#NULL!	#NULL!	#NULL!	#NULL!
38.40	77.40	26.20	33.90	167.00	13.40	#NULL!	11.50
32.10	71.30	24.60	30.20	115.00	16.00	#NULL!	9.80
37.30	79.90	25.70	32.20	137.00	13.70	#NULL!	#NULL!
34.90	85.70	28.70	33.50	37.00	14.00	#NULL!	#NULL!
34.00	83.10	27.90	33.50	188.00	13.40	#NULL!	9.30
48.50	84.50	27.40	32.40	217.00	18.30	#NULL!	10.80
#NULL!	#NULL!	#NULL!	#NULL!	#NULL!	#NULL!	#NULL!	#NULL!
40.80	79.70	26.40	33.10	154.00	14.90	#NULL!	12.10
40.70	86.60	29.80	34.40	127.00	13.90	#NULL!	9.90
41.80	80.20	26.50	33.00	175.00	14.90	#NULL!	10.90
35.90	88.20	30.50	34.50	276.00	14.80	#NULL!	10.20
36.00	84.70	29.20	34.40	194.00	12.70	#NULL!	8.90
35.60	82.00	28.60	34.80	110.00	13.70	#NULL!	11.50
38.70	82.50	29.00	35.10	113.00	13.40	#NULL!	11.40
27.20	91.60	31.00	33.80	159.00	14.40	#NULL!	13.30
35.20	79.60	27.80	34.90	125.00	13.20	#NULL!	10.50
38.10	77.40	26.80	34.60	100.00	#NULL!	#NULL!	#NULL!
37.20	81.60	27.20	33.30	389.00	14.60	#NULL!	9.50
27.80	87.40	30.20	34.50	165.00	14.30	#NULL!	9.60
42.40	82.70	28.70	34.70	77.00	15.60	#NULL!	11.00
38.10	91.40	31.70	34.60	185.00	12.80	#NULL!	10.70
45.40	97.20	34.70	35.70	120.00	14.10	#NULL!	11.20
49.20	86.90	30.20	34.80	185.00	14.80	#NULL!	11.50
39.90	90.30	30.80	34.10	80.00	13.70	#NULL!	11.70
41.60	83.90	28.40	33.90	100.00	13.90	#NULL!	11.30
40.60	89.20	30.80	34.50	118.00	13.50	#NULL!	10.70
42.60	76.90	27.30	35.40	195.00	13.50	#NULL!	9.30
26.20	66.80	19.60	29.40	254.00	17.10	#NULL!	8.30
#NULL!	#NULL!	#NULL!	#NULL!	#NULL!	#NULL!	#NULL!	#NULL!
42.90	76.70	25.60	33.30	222.00	13.10	#NULL!	10.70
37.40	83.50	27.90	33.40	318.00	14.40	#NULL!	10.10
34.20	91.20	31.50	34.50	88.00	12.70	#NULL!	10.60
33.10	79.00	28.90	36.60	189.00	12.80	#NULL!	11.20
36.30	84.40	30.20	35.80	91.00	12.20	#NULL!	11.70
39.10	85.60	29.80	34.80	283.00	13.10	#NULL!	9.50
49.40	88.10	29.60	33.60	130.00	15.50	#NULL!	10.10
41.00	93.40	34.90	37.30	194.00	14.00	#NULL!	9.10
37.60	90.80	30.40	33.50	133.00	14.00	#NULL!	12.40
39.20	90.70	29.40	32.40	274.00	16.70	#NULL!	9.10
44.80	82.80	29.20	35.30	165.00	16.80	#NULL!	11.10
44.50	87.80	32.50	37.10	124.00	13.80	#NULL!	12.10
#NULL!	#NULL!	#NULL!	#NULL!	#NULL!	#NULL!	#NULL!	#NULL!
40.90	79.70	27.50	34.50	198.00	13.70	#NULL!	9.30
43.50	81.60	29.60	36.30	173.00	12.80	#NULL!	10.30

43.00	90.50	31.20	34.40	210.00	13.30	#NULL!	9.90
46.20	88.80	31.50	35.50	147.00	12.80	#NULL!	10.10
14.30	41.30	88.10	31.10	35.40	114.00	#NULL!	#NULL!
40.20	91.40	33.20	36.30	115.00	12.50	#NULL!	11.00
42.80	86.50	29.50	34.10	272.00	13.00	#NULL!	9.90
39.20	101.00	33.50	33.20	248.00	15.10	#NULL!	9.90
27.90	78.80	23.70	30.10	89.00	18.10	#NULL!	#NULL!
43.80	88.50	29.50	33.30	310.00	13.90	#NULL!	10.80
#NULL!	#NULL!	#NULL!	#NULL!	#NULL!	#NULL!	#NULL!	#NULL!
42.10	85.40	29.20	34.20	250.00	13.30	#NULL!	9.00
43.20	86.70	31.90	36.80	174.00	14.20	#NULL!	10.10
39.90	89.30	33.30	37.30	245.00	13.40	#NULL!	10.00
32.10	87.70	29.20	33.30	138.00	16.70	#NULL!	10.90
38.70	82.90	27.80	33.60	176.00	15.00	#NULL!	11.20
35.60	85.60	24.00	28.10	162.00	14.90	#NULL!	14.00
28.40	63.40	20.50	32.40	219.00	#NULL!	#NULL!	#NULL!
37.70	84.90	30.40	35.80	240.00	13.20	#NULL!	9.70
40.40	88.20	31.40	35.60	114.00	13.90	#NULL!	10.60
50.10	90.80	30.10	33.10	126.00	13.30	#NULL!	9.30
34.90	90.90	29.40	32.40	224.00	15.10	#NULL!	10.60
31.40	85.30	29.30	34.40	76.00	13.70	#NULL!	10.20
25.40	86.40	28.20	32.70	285.00	14.50	#NULL!	9.70
47.60	94.80	31.10	32.80	192.00	15.20	#NULL!	11.10
37.60	84.90	29.10	34.30	44.00	14.60	#NULL!	#NULL!
36.60	85.50	30.10	35.20	249.00	12.90	#NULL!	9.80
41.60	88.10	30.90	35.10	145.00	13.10	#NULL!	12.00
35.70	92.70	30.40	32.80	252.00	13.50	#NULL!	9.70
38.70	81.10	28.10	34.60	92.00	13.60	#NULL!	10.80
40.70	82.60	34.30	41.50	134.00	14.40	#NULL!	9.20
47.40	89.40	31.90	35.70	131.00	14.20	#NULL!	10.70
45.30	92.60	33.90	36.60	80.00	13.10	#NULL!	#NULL!

DDimer	Glu	total.chole	TG	HDL	LDL	AST	ALT
0.49	146.00	47.20	0.84	138.00	3.80	37.00	35.00
3.12	#NULL!	#NULL!	#NULL!	#NULL!	#NULL!	33.00	62.00
0.16	#NULL!	32.00	1.00	138.00	3.70	27.00	19.00
0.80	#NULL!	#NULL!	#NULL!	#NULL!	#NULL!	28.00	20.00
1.70	55.00	14.00	0.60	136.00	4.30	38.00	47.00
0.45	90.00	38.00	1.30	137.00	4.50	42.00	20.00
1.00	#NULL!	#NULL!	#NULL!	#NULL!	#NULL!	34.00	30.00
0.34	#NULL!	#NULL!	#NULL!	#NULL!	#NULL!	38.00	30.00
0.37	#NULL!	#NULL!	#NULL!	#NULL!	#NULL!	32.00	56.00
0.46	#NULL!	27.00	0.94	135.00	3.90	35.00	38.00
3.12	#NULL!	#NULL!	#NULL!	#NULL!	#NULL!	35.00	28.00
0.36	122.00	22.00	0.90	140.00	3.90	135.00	56.00
0.10	#NULL!	#NULL!	#NULL!	#NULL!	#NULL!	36.00	36.00
1.45	#NULL!	#NULL!	#NULL!	#NULL!	#NULL!	52.00	66.00
1.78	#NULL!	#NULL!	#NULL!	#NULL!	#NULL!	44.00	40.00
0.30	#NULL!	#NULL!	#NULL!	#NULL!	#NULL!	99.00	157.00
0.19	72.00	13.00	0.80	138.00	4.50	43.00	28.00
0.45	92.00	14.00	0.70	137.00	2.30	130.00	69.00
0.35	#NULL!	#NULL!	#NULL!	#NULL!	#NULL!	42.00	44.00
1.17	#NULL!	#NULL!	#NULL!	#NULL!	#NULL!	64.00	75.00
4.35	#NULL!	#NULL!	#NULL!	#NULL!	#NULL!	36.00	3.00
3.02	#NULL!	#NULL!	#NULL!	#NULL!	#NULL!	56.00	67.00
0.34	#NULL!	28.00	0.60	140.00	4.10	30.00	39.00
0.51	#NULL!	#NULL!	#NULL!	#NULL!	#NULL!	46.00	31.00
0.34	#NULL!	28.00	0.90	139.00	3.90	53.00	73.00
0.65	#NULL!	#NULL!	#NULL!	#NULL!	#NULL!	33.00	34.00
0.24	#NULL!	#NULL!	#NULL!	#NULL!	#NULL!	221.00	268.00
4.02	#NULL!	#NULL!	#NULL!	#NULL!	0.00	57.00	38.00
0.97	104.00	26.00	0.80	141.00	4.80	35.00	55.00
0.39	#NULL!	#NULL!	#NULL!	#NULL!	#NULL!	36.00	48.00
0.75	#NULL!	#NULL!	#NULL!	#NULL!	#NULL!	46.00	34.00
0.90	107.00	21.20	1.15	138.00	4.00	30.00	24.00
0.32	#NULL!	21.00	1.70	139.00	3.90	49.00	41.00
0.30	106.00	29.50	1.27	134.00	4.10	30.00	27.00
1.16	#NULL!	#NULL!	#NULL!	#NULL!	#NULL!	50.00	50.00
0.88	#NULL!	#NULL!	#NULL!	#NULL!	#NULL!	78.00	126.00
0.25	#NULL!	27.00	0.80	138.00	4.20	35.00	52.00
0.85	#NULL!	#NULL!	#NULL!	#NULL!	#NULL!	37.00	28.00
0.25	#NULL!	#NULL!	#NULL!	#NULL!	#NULL!	37.00	61.00
0.21	#NULL!	#NULL!	#NULL!	#NULL!	#NULL!	31.00	36.00
0.68	#NULL!	#NULL!	#NULL!	#NULL!	#NULL!	42.00	44.00
0.50	#NULL!	37.00	1.50	139.00	4.50	38.00	59.00
0.50	#NULL!	26.00	0.80	138.00	4.10	9.00	13.00
0.51	85.00	22.00	1.50	137.00	4.30	59.00	89.00
0.20	121.00	24.00	1.10	138.00	4.00	78.00	118.00
0.54	#NULL!	#NULL!	#NULL!	#NULL!	#NULL!	58.00	41.00
0.65	137.00	32.00	0.80	140.00	4.60	27.00	45.00
3.15	#NULL!	#NULL!	#NULL!	#NULL!	#NULL!	55.00	30.00
1.23	#NULL!	#NULL!	#NULL!	#NULL!	#NULL!	128.00	68.00
0.65	#NULL!	#NULL!	#NULL!	#NULL!	#NULL!	35.00	71.00
0.30	135.00	18.00	0.70	138.00	4.10	35.00	44.00
0.19	#NULL!	#NULL!	#NULL!	#NULL!	#NULL!	37.00	28.00

3.57	#NULL!	#NULL!	#NULL!	#NULL!	#NULL!	44.00	42.00
0.54	109.00	19.00	0.90	137.00	4.20	25.00	40.00
1.32	#NULL!	#NULL!	#NULL!	#NULL!	#NULL!	45.00	55.00
0.37	159.00	30.10	0.75	139.00	4.00	24.00	20.00
0.35	#NULL!	#NULL!	#NULL!	#NULL!	#NULL!	39.00	37.00
0.14	#NULL!	#NULL!	#NULL!	#NULL!	#NULL!	41.00	33.00
0.40	116.00	17.00	0.80	134.00	4.80	218.00	98.00
0.54	178.00	33.00	1.30	138.00	4.10	36.00	30.00
0.78	#NULL!	#NULL!	#NULL!	#NULL!	#NULL!	57.00	68.00
0.30	75.00	36.00	1.10	136.00	3.60	24.00	27.00
0.57	#NULL!	32.00	1.00	143.00	4.50	70.00	42.00
0.89	#NULL!	#NULL!	#NULL!	#NULL!	#NULL!	52.00	71.00
0.36	#NULL!	23.70	0.76	134.00	3.80	39.00	17.00
0.63	133.00	43.00	1.20	137.00	3.40	23.00	57.00
0.37	#NULL!	#NULL!	#NULL!	#NULL!	#NULL!	58.00	83.00
0.40	#NULL!	#NULL!	#NULL!	#NULL!	#NULL!	34.00	27.00
1.34	105.00	40.60	1.36	139.00	4.00	43.00	39.00
0.78	#NULL!	#NULL!	#NULL!	#NULL!	#NULL!	45.00	32.00
0.43	89.00	12.00	0.70	142.00	4.40	112.00	80.00
0.34	71.00	67.00	0.90	136.00	3.70	24.00	11.00
0.84	#NULL!	56.00	1.00	140.00	4.00	55.00	94.00
0.29	119.00	17.00	0.90	138.00	4.40	48.00	53.00
2.45	#NULL!	#NULL!	#NULL!	#NULL!	#NULL!	26.00	25.00
1.32	#NULL!	27.00	0.70	145.00	4.30	35.00	15.00
0.45	#NULL!	#NULL!	#NULL!	#NULL!	#NULL!	#NULL!	#NULL!
1.10	#NULL!	#NULL!	#NULL!	#NULL!	#NULL!	28.00	36.00
0.90	86.00	52.00	1.00	145.00	3.90	22.00	26.00
2.04	#NULL!	#NULL!	#NULL!	#NULL!	#NULL!	52.00	28.00
0.89	#NULL!	#NULL!	#NULL!	#NULL!	#NULL!	48.00	45.00
0.65	#NULL!	#NULL!	#NULL!	#NULL!	#NULL!	100.00	105.00
0.89	#NULL!	#NULL!	#NULL!	#NULL!	#NULL!	30.00	34.00
0.35	#NULL!	24.00	0.70	143.00	4.50	33.00	29.00
0.78	174.00	16.00	0.90	141.00	3.40	89.00	50.00
0.30	366.00	37.00	0.70	130.00	4.20	46.00	40.00
0.23	#NULL!	#NULL!	#NULL!	#NULL!	#NULL!	24.00	26.00
0.30	#NULL!	#NULL!	#NULL!	#NULL!	#NULL!	202.00	267.00
3.54	#NULL!	52.00	1.40	135.00	5.00	40.00	66.00
0.23	#NULL!	#NULL!	#NULL!	#NULL!	#NULL!	51.00	69.00
0.30	#NULL!	#NULL!	#NULL!	#NULL!	#NULL!	92.00	78.00
0.75	#NULL!	#NULL!	#NULL!	#NULL!	#NULL!	59.00	41.00
1.25	#NULL!	#NULL!	#NULL!	#NULL!	#NULL!	39.00	28.00
0.45	#NULL!	#NULL!	#NULL!	#NULL!	#NULL!	#NULL!	#NULL!
0.34	#NULL!	#NULL!	#NULL!	#NULL!	#NULL!	30.00	35.00
3.32	#NULL!	#NULL!	#NULL!	#NULL!	#NULL!	62.00	64.00
0.37	#NULL!	#NULL!	#NULL!	#NULL!	#NULL!	31.00	45.00
1.35	#NULL!	#NULL!	#NULL!	#NULL!	#NULL!	42.00	31.00
2.52	87.00	42.00	1.10	140.00	4.20	28.00	14.00
0.68	#NULL!	#NULL!	#NULL!	#NULL!	#NULL!	162.00	127.00
1.10	#NULL!	#NULL!	#NULL!	#NULL!	#NULL!	44.00	53.00
2.70	#NULL!	#NULL!	#NULL!	#NULL!	#NULL!	67.00	72.00
1.00	#NULL!	#NULL!	#NULL!	#NULL!	#NULL!	56.00	75.00
3.20	#NULL!	#NULL!	#NULL!	#NULL!	#NULL!	40.00	61.00
0.51	143.00	28.00	0.80	147.00	4.40	56.00	27.00

0.17	#NULL!	#NULL!	#NULL!	#NULL!	#NULL!	59.00	97.00
0.36	#NULL!	#NULL!	#NULL!	#NULL!	#NULL!	54.00	53.00
0.87	123.00	52.00	0.90	138.00	3.60	14.00	33.00
0.36	128.00	30.70	0.89	137.00	3.80	61.00	65.00
0.75	#NULL!	#NULL!	#NULL!	#NULL!	#NULL!	37.00	39.00
0.56	#NULL!	#NULL!	#NULL!	#NULL!	#NULL!	42.00	47.00
0.36	#NULL!	25.00	1.20	133.00	4.00	#NULL!	#NULL!
0.32	#NULL!	30.00	0.60	137.00	4.10	21.00	27.00
2.31	#NULL!	#NULL!	#NULL!	#NULL!	#NULL!	30.00	75.00
#NULL!	#NULL!	#NULL!	#NULL!	#NULL!	#NULL!	35.00	44.00
0.38	125.00	34.00	1.10	135.00	3.90	21.00	11.00
0.59	107.00	46.10	1.53	140.00	4.60	65.00	52.00
0.75	139.00	38.00	1.18	142.00	4.00	42.00	54.00
0.26	154.00	51.00	1.10	135.00	4.20	103.00	92.00
0.31	#NULL!	#NULL!	#NULL!	#NULL!	#NULL!	86.00	119.00
1.40	#NULL!	#NULL!	#NULL!	#NULL!	#NULL!	46.00	40.00
0.17	#NULL!	#NULL!	#NULL!	#NULL!	#NULL!	34.00	41.00
0.10	325.00	37.00	1.00	138.00	4.90	64.00	37.00
0.19	#NULL!	#NULL!	#NULL!	#NULL!	#NULL!	38.00	50.00
0.58	#NULL!	#NULL!	#NULL!	#NULL!	#NULL!	106.00	88.00
0.45	#NULL!	#NULL!	#NULL!	#NULL!	#NULL!	42.00	48.00
0.10	#NULL!	#NULL!	#NULL!	#NULL!	#NULL!	40.00	49.00
0.42	120.00	39.00	1.20	137.00	4.00	29.00	25.00
4.50	#NULL!	#NULL!	#NULL!	#NULL!	#NULL!	78.00	89.00
0.49	100.00	27.00	0.80	141.00	3.80	33.00	51.00
0.40	#NULL!	37.00	1.00	135.00	4.90	32.00	33.00
0.21	100.00	17.00	0.80	131.00	3.90	26.00	46.00
8.60	#NULL!	#NULL!	#NULL!	#NULL!	#NULL!	85.00	64.00
0.84	#NULL!	#NULL!	#NULL!	#NULL!	#NULL!	53.00	105.00
0.45	161.00	166.60	1.61	138.00	3.80	121.00	52.00
0.14	#NULL!	#NULL!	#NULL!	#NULL!	#NULL!	50.00	86.00
1.20	#NULL!	#NULL!	#NULL!	#NULL!	#NULL!	47.00	31.00
0.16	#NULL!	#NULL!	#NULL!	#NULL!	#NULL!	53.00	37.00
0.35	110.00	39.00	0.80	138.00	4.10	20.00	41.00
2.15	#NULL!	#NULL!	#NULL!	#NULL!	#NULL!	49.00	9.00
0.58	178.00	47.00	0.80	135.00	3.90	47.00	33.00
0.46	#NULL!	#NULL!	#NULL!	#NULL!	#NULL!	77.00	68.00
2.01	#NULL!	#NULL!	#NULL!	#NULL!	#NULL!	45.00	46.00
0.16	130.00	53.00	0.90	141.00	4.00	26.00	39.00
5.10	#NULL!	32.00	0.90	137.00	3.70	20.00	28.00
0.85	#NULL!	#NULL!	#NULL!	#NULL!	#NULL!	45.00	56.00
0.13	#NULL!	174.00	115.00	51.00	100.00	45.00	38.00
2.00	#NULL!	#NULL!	#NULL!	#NULL!	#NULL!	873.00	927.00
1.00	106.00	42.00	0.90	133.00	4.00	31.00	26.00
0.26	157.00	42.00	1.20	139.00	3.80	#NULL!	#NULL!
0.17	#NULL!	#NULL!	#NULL!	#NULL!	#NULL!	106.00	89.00
0.35	#NULL!	#NULL!	#NULL!	#NULL!	#NULL!	42.00	46.00
3.01	#NULL!	#NULL!	#NULL!	#NULL!	#NULL!	31.00	36.00
3.21	#NULL!	#NULL!	#NULL!	#NULL!	#NULL!	33.00	45.00
0.78	#NULL!	#NULL!	#NULL!	#NULL!	#NULL!	52.00	61.00
0.14	#NULL!	#NULL!	#NULL!	#NULL!	#NULL!	74.00	7.00
0.33	140.00	28.90	0.95	132.00	4.30	48.00	38.00
1.50	177.00	29.20	0.84	136.00	4.40	40.00	24.00

0.86	#NULL!	#NULL!	#NULL!	#NULL!	#NULL!	27.00	16.00
1.00	#NULL!	#NULL!	#NULL!	#NULL!	#NULL!	56.00	22.00
0.78	#NULL!	#NULL!	#NULL!	#NULL!	#NULL!	39.00	43.00
1.13	#NULL!	#NULL!	#NULL!	#NULL!	#NULL!	51.00	54.00
2.54	#NULL!	#NULL!	#NULL!	#NULL!	#NULL!	295.00	279.00
0.24	#NULL!	#NULL!	#NULL!	#NULL!	#NULL!	69.00	62.00
0.62	#NULL!	21.00	0.80	136.00	3.70	39.00	9.00
0.34	#NULL!	198.00	122.00	#NULL!	#NULL!	198.00	122.00
0.66	#NULL!	#NULL!	#NULL!	#NULL!	#NULL!	44.00	37.00
0.65	105.00	13.00	0.80	125.00	3.70	37.00	43.00
1.70	#NULL!	#NULL!	#NULL!	#NULL!	#NULL!	59.00	41.00
5.20	#NULL!	#NULL!	#NULL!	#NULL!	#NULL!	72.00	89.00
0.53	#NULL!	#NULL!	#NULL!	#NULL!	#NULL!	67.00	66.00
1.14	#NULL!	#NULL!	#NULL!	#NULL!	#NULL!	49.00	69.00
0.16	#NULL!	#NULL!	#NULL!	#NULL!	#NULL!	2.00	38.00
0.91	#NULL!	#NULL!	#NULL!	#NULL!	0.00	55.00	26.00
0.31	#NULL!	107.00	115.00	40.00	44.00	131.00	43.00
0.54	#NULL!	#NULL!	#NULL!	#NULL!	#NULL!	36.00	28.00
0.35	#NULL!	#NULL!	#NULL!	#NULL!	#NULL!	112.00	73.00
3.54	#NULL!	#NULL!	#NULL!	#NULL!	#NULL!	39.00	74.00
2.40	99.00	63.00	0.90	131.00	5.40	65.00	67.00
0.10	#NULL!	#NULL!	#NULL!	#NULL!	#NULL!	60.00	60.00
0.85	#NULL!	#NULL!	#NULL!	#NULL!	#NULL!	41.00	38.00
0.46	228.00	49.00	1.00	132.00	4.90	45.00	55.00
0.16	#NULL!	#NULL!	#NULL!	#NULL!	#NULL!	56.00	50.00
0.75	#NULL!	#NULL!	#NULL!	#NULL!	#NULL!	90.00	78.00
3.45	#NULL!	135.00	228.00	40.00	49.40	36.00	37.00
1.05	#NULL!	#NULL!	#NULL!	#NULL!	#NULL!	48.00	55.00
0.32	132.00	64.80	1.20	140.00	4.00	37.00	34.00
2.31	#NULL!	#NULL!	#NULL!	#NULL!	#NULL!	54.00	41.00
0.55	#NULL!	#NULL!	#NULL!	#NULL!	0.00	103.00	76.00
5.13	#NULL!	#NULL!	#NULL!	#NULL!	#NULL!	35.00	28.00
0.41	#NULL!	#NULL!	#NULL!	#NULL!	#NULL!	71.00	29.00
0.75	#NULL!	#NULL!	#NULL!	#NULL!	#NULL!	72.00	91.00
0.40	#NULL!	#NULL!	#NULL!	#NULL!	#NULL!	56.00	51.00
0.85	#NULL!	#NULL!	#NULL!	#NULL!	#NULL!	34.00	37.00
0.85	#NULL!	#NULL!	#NULL!	#NULL!	#NULL!	41.00	28.00
2.35	#NULL!	#NULL!	#NULL!	#NULL!	#NULL!	60.00	35.00
7.40	97.00	40.00	1.10	141.00	3.50	41.00	18.00
1.49	403.00	75.00	1.20	141.00	4.50	61.00	27.00
1.00	#NULL!	#NULL!	#NULL!	#NULL!	#NULL!	78.00	62.00
4.00	#NULL!	#NULL!	#NULL!	#NULL!	#NULL!	44.00	45.00
1.50	#NULL!	#NULL!	#NULL!	#NULL!	#NULL!	23.00	22.00
0.31	295.00	47.00	1.26	130.00	3.60	22.00	15.00
0.35	#NULL!	#NULL!	#NULL!	#NULL!	#NULL!	71.00	87.00
0.86	#NULL!	#NULL!	#NULL!	#NULL!	#NULL!	98.00	95.00
0.89	#NULL!	#NULL!	#NULL!	#NULL!	#NULL!	45.00	45.00
0.80	#NULL!	#NULL!	#NULL!	#NULL!	#NULL!	140.00	118.00
0.35	#NULL!	#NULL!	#NULL!	#NULL!	#NULL!	49.00	37.00
0.45	#NULL!	#NULL!	#NULL!	#NULL!	#NULL!	51.00	53.00
0.53	#NULL!	21.00	0.80	140.00	4.00	65.00	41.00
0.45	#NULL!	#NULL!	#NULL!	#NULL!	#NULL!	35.00	40.00
1.02	130.00	44.00	1.10	131.00	4.80	72.00	51.00

0.19	#NULL!	#NULL!	#NULL!	#NULL!	#NULL!	55.00	53.00
0.50	#NULL!	#NULL!	#NULL!	#NULL!	#NULL!	31.00	37.00
0.14	#NULL!	#NULL!	#NULL!	#NULL!	#NULL!	50.00	35.00
0.84	#NULL!	112.00	133.00	#NULL!	44.40	50.00	36.00
3.54	#NULL!	#NULL!	#NULL!	#NULL!	#NULL!	51.00	46.00
3.50	#NULL!	#NULL!	#NULL!	#NULL!	#NULL!	40.00	46.00
0.34	#NULL!	#NULL!	#NULL!	#NULL!	#NULL!	56.00	72.00
0.75	#NULL!	94.00	73.00	40.00	39.40	77.00	75.00
0.42	#NULL!	#NULL!	#NULL!	#NULL!	#NULL!	194.00	259.00
0.66	151.00	52.50	1.32	135.00	4.10	18.00	15.00
0.70	#NULL!	#NULL!	#NULL!	#NULL!	#NULL!	90.00	52.00
1.57	#NULL!	#NULL!	#NULL!	#NULL!	#NULL!	38.00	52.00
0.86	#NULL!	#NULL!	#NULL!	#NULL!	#NULL!	54.00	55.00
0.89	#NULL!	#NULL!	83.00	#NULL!	#NULL!	74.00	43.00
2.45	#NULL!	#NULL!	#NULL!	#NULL!	#NULL!	30.00	45.00
0.75	#NULL!	#NULL!	#NULL!	#NULL!	#NULL!	126.00	293.00
0.82	#NULL!	#NULL!	#NULL!	#NULL!	#NULL!	42.00	41.00
3.14	#NULL!	57.00	1.50	136.00	3.80	79.00	42.00
0.47	#NULL!	#NULL!	#NULL!	#NULL!	#NULL!	54.00	48.00
2.54	#NULL!	#NULL!	#NULL!	#NULL!	#NULL!	66.00	40.00
0.44	#NULL!	#NULL!	#NULL!	#NULL!	#NULL!	39.00	40.00
0.89	#NULL!	#NULL!	#NULL!	#NULL!	#NULL!	65.00	74.00
0.47	224.00	31.00	0.80	130.00	4.00	48.00	51.00
0.98	#NULL!	#NULL!	#NULL!	#NULL!	#NULL!	23.00	17.00
0.34	#NULL!	#NULL!	#NULL!	#NULL!	#NULL!	48.00	35.00
0.43	#NULL!	#NULL!	#NULL!	#NULL!	#NULL!	36.00	29.00
4.12	#NULL!	#NULL!	#NULL!	#NULL!	#NULL!	110.00	76.00
0.12	#NULL!	#NULL!	#NULL!	#NULL!	#NULL!	53.00	30.00
0.14	#NULL!	#NULL!	#NULL!	#NULL!	#NULL!	234.00	110.00
0.93	100.00	27.00	1.10	139.00	3.80	80.00	31.00
0.31	#NULL!	#NULL!	#NULL!	#NULL!	#NULL!	60.00	48.00
0.35	#NULL!	#NULL!	#NULL!	#NULL!	#NULL!	#NULL!	#NULL!
0.77	250.00	27.00	1.10	138.00	4.40	87.00	49.00
0.59	155.00	30.00	1.00	134.00	4.10	54.00	56.00
0.33	#NULL!	#NULL!	#NULL!	#NULL!	#NULL!	28.00	30.00
0.35	#NULL!	#NULL!	#NULL!	#NULL!	#NULL!	84.00	72.00
0.30	182.00	41.20	0.96	137.00	4.30	46.00	79.00
0.95	#NULL!	#NULL!	#NULL!	#NULL!	#NULL!	47.00	46.00
1.35	#NULL!	#NULL!	#NULL!	#NULL!	#NULL!	42.00	37.00
0.54	162.00	23.00	0.90	141.00	4.40	30.00	20.00
4.30	#NULL!	#NULL!	#NULL!	#NULL!	0.00	68.00	61.00
0.60	234.00	39.00	0.90	136.00	4.30	51.00	32.00
0.58	#NULL!	#NULL!	#NULL!	#NULL!	#NULL!	40.00	19.00
0.56	#NULL!	#NULL!	#NULL!	#NULL!	#NULL!	44.00	33.00
0.80	153.00	52.00	1.00	139.00	3.80	29.00	21.00
0.19	#NULL!	#NULL!	#NULL!	#NULL!	#NULL!	95.00	74.00
3.20	#NULL!	#NULL!	#NULL!	#NULL!	#NULL!	53.00	24.00
0.35	#NULL!	#NULL!	#NULL!	#NULL!	#NULL!	14.00	13.00
0.45	#NULL!	#NULL!	#NULL!	#NULL!	#NULL!	73.00	164.00
2.31	#NULL!	#NULL!	#NULL!	#NULL!	#NULL!	#NULL!	#NULL!
0.25	#NULL!	#NULL!	#NULL!	#NULL!	#NULL!	271.00	232.00
0.87	#NULL!	#NULL!	#NULL!	#NULL!	#NULL!	40.00	27.00
3.45	#NULL!	#NULL!	#NULL!	#NULL!	#NULL!	30.00	38.00

0.83	105.00	35.00	1.20	138.00	4.10	36.00	26.00
1.54	#NULL!	#NULL!	#NULL!	#NULL!	0.00	37.00	31.00
1.25	#NULL!	#NULL!	#NULL!	#NULL!	#NULL!	60.00	52.00
0.88	#NULL!	#NULL!	#NULL!	#NULL!	#NULL!	62.00	59.00
0.67	#NULL!	#NULL!	#NULL!	#NULL!	#NULL!	62.00	91.00
0.56	#NULL!	#NULL!	#NULL!	#NULL!	#NULL!	79.00	2.00
1.02	#NULL!	#NULL!	#NULL!	#NULL!	#NULL!	44.00	35.00
3.01	#NULL!	#NULL!	#NULL!	#NULL!	#NULL!	37.00	50.00
4.52	#NULL!	#NULL!	#NULL!	#NULL!	#NULL!	29.00	18.00
0.87	#NULL!	#NULL!	#NULL!	#NULL!	#NULL!	58.00	38.00
0.35	#NULL!	#NULL!	#NULL!	#NULL!	0.00	66.00	32.00
1.60	#NULL!	#NULL!	#NULL!	#NULL!	#NULL!	51.00	55.00
2.32	#NULL!	#NULL!	#NULL!	#NULL!	#NULL!	62.00	52.00
0.75	#NULL!	#NULL!	#NULL!	#NULL!	#NULL!	48.00	35.00
0.40	#NULL!	#NULL!	#NULL!	#NULL!	#NULL!	#NULL!	#NULL!
2.02	#NULL!	#NULL!	#NULL!	#NULL!	#NULL!	50.00	43.00
3.41	#NULL!	#NULL!	#NULL!	#NULL!	#NULL!	61.00	62.00
1.35	#NULL!	#NULL!	#NULL!	#NULL!	#NULL!	58.00	39.00
4.21	#NULL!	#NULL!	#NULL!	#NULL!	0.00	245.00	261.00
0.65	#NULL!	#NULL!	#NULL!	#NULL!	0.00	35.00	31.00
2.41	#NULL!	#NULL!	#NULL!	#NULL!	#NULL!	54.00	39.00
5.01	#NULL!	#NULL!	#NULL!	#NULL!	#NULL!	19.00	25.00
0.56	#NULL!	#NULL!	#NULL!	#NULL!	#NULL!	31.00	45.00
0.98	#NULL!	#NULL!	#NULL!	#NULL!	#NULL!	72.00	54.00
1.30	#NULL!	#NULL!	#NULL!	#NULL!	#NULL!	79.00	89.00
8.76	#NULL!	#NULL!	#NULL!	#NULL!	#NULL!	101.00	98.00
0.56	#NULL!	140.00	75.00	30.00	95.00	30.00	27.00
0.78	#NULL!	#NULL!	#NULL!	#NULL!	#NULL!	27.00	17.00
0.45	78.00	52.10	0.90	134.00	4.40	95.00	112.00
0.54	#NULL!	#NULL!	#NULL!	#NULL!	#NULL!	67.00	48.00
0.75	#NULL!	#NULL!	#NULL!	#NULL!	#NULL!	56.00	40.00

ALP	HSCRP	Urea	Cr	TBili	DBili	LDH	CPK
143.00	0.47	0.23	3.90	125.00	10.00	765.00	8.10
131.00	4.00	41.00	1.40	0.70	0.30	401.00	40.00
203.00	0.50	0.20	#NULL!	253.00	24.00	225.00	8.50
206.00	20.00	34.00	1.30	0.60	0.30	675.00	243.00
434.00	0.50	0.20	4.20	50.00	12.00	905.00	8.50
70.00	0.70	0.20	4.00	1880.00	10.00	920.00	87.00
308.00	5.50	18.00	0.80	0.60	0.30	452.00	42.00
162.00	16.10	53.00	1.10	0.50	0.20	473.00	52.00
186.00	16.00	17.00	0.90	0.70	0.30	451.00	50.00
82.00	0.40	0.10	4.00	250.00	35.70	615.00	8.40
92.00	15.00	15.00	0.80	0.50	0.20	928.00	94.00
189.00	1.10	0.50	#NULL!	435.00	25.00	562.00	9.60
216.00	11.00	43.00	1.40	0.80	0.40	595.00	119.00
160.00	12.30	44.00	1.80	0.60	0.30	608.00	53.00
306.00	9.60	12.00	0.80	1.40	0.70	385.00	40.00
162.00	28.00	19.00	1.10	0.90	0.40	548.00	1217.00
110.00	0.50	0.20	3.60	65.00	8.00	539.00	9.00
3958.00	3.60	1.70	4.20	55.00	12.00	809.00	9.30
249.00	57.00	13.00	0.60	0.80	0.30	375.00	45.00
165.00	0.98	23.00	1.00	0.50	0.20	712.00	81.00
134.00	4.80	15.00	0.90	0.50	0.20	655.00	196.00
415.00	12.00	42.00	1.40	0.50	0.20	648.00	268.00
103.00	0.50	0.20	4.10	79.00	14.30	356.00	9.10
209.00	2.00	18.00	0.80	0.50	0.20	544.00	40.00
150.00	0.50	0.20	3.30	188.00	22.00	341.00	7.50
90.00	13.00	24.00	1.00	0.70	0.40	750.00	40.00
380.00	12.00	17.00	0.80	0.50	0.20	995.00	409.00
322.00	9.50	32.00	1.50	0.60	0.30	860.00	18.00
117.00	0.90	0.30	#NULL!	45.00	15.00	485.00	9.60
172.00	78.00	32.00	0.90	0.90	0.40	577.00	120.00
102.00	87.00	31.00	0.90	0.40	0.20	766.00	288.00
167.00	0.42	0.22	4.00	361.00	40.00	375.00	8.20
165.00	0.80	0.40	3.00	982.00	72.00	504.00	8.40
159.00	0.40	0.16	3.00	76.00	12.50	490.00	8.60
129.00	22.00	29.00	1.30	0.60	0.30	652.00	86.00
168.00	9.80	37.00	1.10	#NULL!	#NULL!	371.00	39.00
257.00	0.60	0.20	#NULL!	#NULL!	#NULL!	474.00	8.00
105.00	9.50	20.00	0.90	0.60	0.20	1050.00	138.00
158.00	1.60	27.00	1.50	0.60	0.30	329.00	132.00
121.00	10.00	10.00	0.80	0.40	0.20	431.00	45.00
222.00	23.00	19.00	1.10	0.50	0.20	647.00	104.00
207.00	0.50	0.20	4.00	70.00	19.00	624.00	9.00
142.00	0.50	0.20	#NULL!	37.00	15.00	434.00	8.60
236.00	0.50	0.20	#NULL!	256.00	125.00	742.00	7.30
208.00	0.50	0.20	3.50	2056.00	250.00	950.00	8.60
167.00	3.00	24.00	1.10	0.60	0.20	565.00	230.00
178.00	0.40	0.20	#NULL!	45.00	#NULL!	506.00	8.00
127.00	9.80	18.00	0.90	0.50	0.20	852.00	170.00
211.00	9.80	60.00	1.00	4.30	2.10	752.00	152.00
94.00	1.00	27.00	1.00	0.60	0.30	425.00	190.00
139.00	0.50	0.20	3.90	105.00	11.00	822.00	8.20
160.00	19.00	15.00	0.80	0.40	0.20	764.00	95.00

115.00	4.00	41.00	1.40	0.80	0.30	557.00	740.00
117.00	0.50	0.20	#NULL!	73.00	10.00	297.00	8.90
373.00	11.00	22.00	1.00	0.70	0.30	606.00	63.00
269.00	0.37	0.20	#NULL!	90.00	22.80	350.00	8.90
378.00	5.30	22.00	1.20	0.50	0.20	641.00	212.00
271.00	10.00	29.00	0.90	0.50	0.20	899.00	1343.00
132.00	0.50	0.20	#NULL!	4679.00	520.00	850.00	7.90
124.00	0.70	0.30	4.00	73.00	11.00	828.00	7.50
217.00	17.00	13.00	1.00	0.50	0.20	999.00	73.00
112.00	0.60	0.30	3.50	59.00	10.00	450.00	8.60
142.00	0.80	0.30	#NULL!	556.00	28.00	762.00	9.30
183.00	2.00	51.00	1.10	1.10	0.60	354.00	54.00
136.00	0.34	0.16	#NULL!	72.00	20.30	505.00	8.70
189.00	0.50	0.20	3.00	58.00	20.00	330.00	8.00
251.00	5.00	38.00	1.20	0.70	0.20	548.00	10.00
135.00	10.50	19.00	0.90	0.40	0.20	796.00	339.00
168.00	0.63	0.37	#NULL!	#NULL!	#NULL!	378.00	7.90
176.00	13.50	39.00	1.00	0.60	0.20	970.00	86.00
130.00	0.50	0.20	#NULL!	116.00	12.80	712.00	8.00
90.00	9.00	0.40	#NULL!	80.00	18.00	664.00	10.00
116.00	0.50	0.20	4.30	326.00	25.00	#NULL!	8.80
115.00	0.60	0.30	#NULL!	119.00	19.00	606.00	8.00
156.00	9.90	17.00	0.80	0.50	0.20	633.00	51.00
144.00	0.40	0.20	3.80	37.00	7.00	522.00	9.30
#NULL!	9.90	27.00	1.30	#NULL!	#NULL!	#NULL!	#NULL!
151.00	99.00	21.00	0.90	0.70	0.20	402.00	91.00
126.00	0.50	0.20	4.00	#NULL!	#NULL!	546.00	10.00
114.00	14.00	49.00	0.90	0.60	0.30	1351.00	447.00
167.00	9.80	28.00	1.10	0.50	0.20	542.00	218.00
277.00	5.50	43.00	1.30	1.60	0.60	621.00	248.00
220.00	95.00	38.00	1.00	0.90	0.30	554.00	115.00
137.00	0.60	0.20	4.00	50.00	21.00	459.00	9.70
112.00	0.50	0.20	4.00	123.00	15.00	635.00	8.00
204.00	0.50	#NULL!	#NULL!	58.00	10.00	357.00	84.00
115.00	3.00	19.00	0.70	0.40	0.20	393.00	46.00
243.00	6.00	25.00	0.90	0.60	0.30	690.00	141.00
201.00	1.00	0.50	#NULL!	201.00	#NULL!	752.00	9.50
161.00	10.00	45.00	1.10	0.80	0.20	360.00	293.00
288.00	13.00	29.00	1.10	0.70	0.30	802.00	115.00
176.00	2.50	14.00	0.90	0.50	0.20	653.00	95.00
170.00	9.10	26.00	1.10	0.50	0.20	591.00	350.00
#NULL!	21.00	40.00	1.40	#NULL!	#NULL!	810.00	110.00
100.00	5.20	28.00	0.70	0.50	0.20	286.00	174.00
222.00	2.30	54.00	1.30	0.60	0.40	1611.00	121.00
188.00	9.80	38.00	0.90	0.80	0.40	380.00	50.00
135.00	2.30	23.00	1.00	0.70	0.20	948.00	121.00
144.00	0.60	0.20	4.50	49.00	10.00	673.00	8.20
1364.00	8.80	32.00	0.60	28.50	14.10	452.00	152.00
356.00	2.50	135.00	4.60	0.80	0.40	1088.00	413.00
241.00	15.90	24.00	1.20	0.50	0.20	1327.00	122.00
217.00	23.00	24.00	0.90	0.60	0.30	380.00	50.00
125.00	2.60	22.00	1.00	1.10	0.60	396.00	56.00
222.00	0.50	0.20	#NULL!	95.00	16.00	394.00	9.20

154.00	11.00	19.00	0.70	1.00	0.50	#NULL!	257.00
170.00	9.90	34.00	0.90	0.70	0.30	930.00	55.00
238.00	7.00	0.30	#NULL!	85.00	23.00	400.00	7.50
133.00	0.72	0.18	4.50	84.00	10.20	587.00	8.60
117.00	1.00	21.00	0.90	0.60	0.30	451.00	352.00
138.00	30.00	50.00	1.00	0.60	0.30	450.00	10.00
#NULL!	13.00	#NULL!	3.60	#NULL!	#NULL!	750.00	78.00
90.00	0.50	0.20	3.50	120.00	16.00	420.00	8.80
172.00	6.80	28.00	0.80	0.50	0.20	713.00	45.00
209.00	7.00	21.00	1.00	1.60	0.80	469.00	201.00
213.00	0.50	0.20	4.30	192.00	16.50	726.00	8.80
122.00	0.88	0.33	3.30	411.00	#NULL!	663.00	9.00
127.00	0.92	0.33	3.80	121.00	28.00	367.00	8.60
110.00	0.50	0.20	#NULL!	389.00	20.00	409.00	8.00
244.00	15.00	47.00	1.40	2.40	1.10	375.00	122.00
189.00	62.00	26.00	0.80	0.50	0.20	620.00	68.00
98.00	26.00	49.00	1.50	0.30	0.10	625.00	175.00
205.00	0.50	0.20	4.00	60.00	13.00	789.00	8.80
107.00	7.00	34.00	0.80	0.60	0.20	365.00	36.00
111.00	9.50	17.00	0.80	0.50	0.20	852.00	411.00
346.00	3.00	25.00	1.00	1.00	0.50	520.00	47.00
124.00	2.30	56.00	1.00	0.70	0.30	537.00	34.00
81.00	0.40	0.20	#NULL!	140.00	10.00	584.00	9.40
88.00	6.00	42.00	1.40	0.50	0.20	372.00	224.00
162.00	0.40	0.20	#NULL!	43.00	12.00	456.00	8.00
162.00	0.50	0.20	#NULL!	254.00	28.00	643.00	9.40
139.00	0.50	0.20	4.00	105.00	10.00	877.00	9.30
122.00	6.90	28.00	1.10	0.40	0.20	793.00	614.00
147.00	2.00	42.00	0.90	0.70	0.40	627.00	357.00
136.00	1.22	0.67	4.40	82.00	10.00	650.00	9.00
213.00	4.00	28.00	1.10	1.20	0.60	652.00	180.00
169.00	15.00	124.00	2.40	0.70	0.20	439.00	285.00
177.00	10.00	48.00	1.00	0.80	0.40	650.00	145.00
189.00	0.60	0.20	#NULL!	#NULL!	#NULL!	377.00	45.00
266.00	2.00	37.00	0.90	0.40	0.20	329.00	54.00
99.00	8.00	0.20	#NULL!	104.00	10.20	1041.00	85.00
100.00	15.00	36.00	1.20	0.90	0.30	716.00	369.00
445.00	3.30	36.00	1.20	0.60	0.30	351.00	38.00
121.00	0.90	0.40	4.00	#NULL!	#NULL!	#NULL!	8.80
150.00	0.60	0.20	#NULL!	165.00	18.00	845.00	8.30
179.00	12.00	63.00	2.10	0.60	0.30	637.00	71.00
192.00	3.10	27.00	0.80	0.90	0.40	697.00	159.00
736.00	9.90	108.00	1.90	3.00	1.50	1505.00	538.00
199.00	0.50	0.20	4.00	84.00	15.00	617.00	8.60
#NULL!	9.00	#NULL!	3.00	#NULL!	#NULL!	542.00	10.00
327.00	8.20	64.00	1.40	0.60	0.30	604.00	62.00
129.00	9.20	35.00	1.00	1.00	0.50	324.00	61.00
164.00	9.80	22.00	0.80	1.00	0.40	581.00	102.00
119.00	12.00	46.00	1.20	0.70	0.30	821.00	111.00
172.00	11.00	70.00	1.40	1.40	0.70	1382.00	200.00
214.00	3.00	33.00	1.30	0.60	0.20	497.00	110.00
120.00	0.36	0.15	#NULL!	689.00	71.70	711.00	9.70
180.00	0.90	0.42	3.10	71.00	27.80	569.00	9.00

94.00	92.00	62.00	0.90	0.40	0.20	513.00	65.00
168.00	9.80	64.00	1.10	1.30	0.60	852.00	278.00
212.00	9.50	24.00	1.00	0.60	0.30	435.00	94.00
280.00	30.00	33.00	0.10	1.10	0.60	334.00	84.00
506.00	8.90	17.00	1.00	1.10	0.60	859.00	444.00
178.00	9.80	50.00	1.20	1.10	0.50	620.00	970.00
161.00	0.60	0.30	4.30	54.00	22.00	941.00	9.50
53.00	76.00	36.00	1.20	0.50	0.20	580.00	366.00
230.00	61.00	57.00	1.10	0.40	0.20	632.00	794.00
152.00	0.90	0.30	4.10	556.00	22.00	300.00	7.80
136.00	20.00	38.00	1.10	1.00	0.50	660.00	176.00
210.00	12.80	39.00	1.10	0.60	0.20	923.00	70.00
145.00	20.40	21.00	1.00	0.80	0.40	833.00	200.00
266.00	2.00	37.00	0.90	0.40	0.20	329.00	54.00
220.00	9.80	25.00	0.70	0.50	0.20	620.00	170.00
127.00	2.00	17.00	0.70	#NULL!	#NULL!	670.00	130.00
236.00	2.40	60.00	1.30	0.50	0.20	730.00	230.00
176.00	11.00	25.00	0.70	0.70	0.20	656.00	51.00
171.00	13.00	40.00	1.70	1.00	0.40	746.00	214.00
144.00	#NULL!	32.00	0.80	0.50	0.20	608.00	94.00
121.00	0.50	0.20	4.00	45.00	9.00	794.00	8.30
368.00	7.80	25.00	1.10	0.40	0.20	586.00	163.00
158.00	23.00	37.00	0.80	0.50	0.20	504.00	124.00
245.00	0.50	0.20	#NULL!	74.00	10.00	453.00	9.90
141.00	3.00	20.00	0.90	0.40	0.20	467.00	344.00
152.00	16.00	32.00	1.40	1.00	0.50	1265.00	179.00
148.00	11.00	27.00	1.00	0.60	0.20	990.00	50.00
368.00	11.00	41.00	1.20	1.10	0.50	478.00	93.00
107.00	0.40	0.20	#NULL!	46.00	18.00	739.00	10.00
155.00	9.80	43.00	1.50	0.50	0.20	789.00	85.00
307.00	9.80	19.00	0.90	0.90	0.50	615.00	93.00
531.00	150.00	281.00	8.20	0.80	0.50	984.00	18.00
99.00	12.00	44.00	1.50	0.50	0.20	914.00	81.00
136.00	7.80	20.00	0.80	0.50	0.20	662.00	102.00
36.00	41.00	27.00	1.20	0.50	0.20	482.00	40.00
208.00	9.80	37.00	1.10	0.50	0.20	328.00	60.00
141.00	29.00	23.00	0.90	0.80	0.40	649.00	364.00
181.00	9.20	58.00	1.00	#NULL!	#NULL!	456.00	190.00
446.00	1.50	0.50	3.30	40.00	10.00	310.00	8.90
226.00	1.10	0.50	#NULL!	45.00	10.00	626.00	8.90
125.00	98.00	48.00	1.20	0.70	0.30	654.00	430.00
152.00	11.00	24.00	0.60	1.10	0.60	849.00	49.00
210.00	68.00	37.00	1.10	0.50	0.20	345.00	67.00
227.00	0.43	0.21	4.00	56.00	18.00	331.00	10.00
162.00	8.00	31.00	0.80	0.80	0.40	752.00	112.00
103.00	16.00	30.00	1.00	0.50	0.20	1317.00	118.00
172.00	11.00	40.00	1.30	1.10	0.60	678.00	286.00
214.00	53.00	64.00	1.40	1.40	0.70	742.00	189.00
184.00	0.50	27.00	0.80	0.90	0.40	520.00	174.00
186.00	2.20	30.00	0.80	1.20	0.60	706.00	105.00
132.00	0.50	0.20	4.10	730.00	51.00	680.00	7.70
423.00	15.00	104.00	1.90	0.80	0.40	439.00	235.00
162.00	1.00	0.30	#NULL!	79.00	11.00	745.00	8.60

109.00	9.80	50.00	1.10	0.70	0.40	430.00	66.00
207.00	9.70	39.00	1.30	0.50	0.20	551.00	92.00
164.00	16.00	31.00	1.00	1.50	0.50	788.00	220.00
308.00	67.00	52.00	1.30	0.60	0.30	459.00	121.00
226.00	8.40	23.00	1.10	0.90	0.30	846.00	51.00
245.00	13.00	64.00	1.10	1.00	0.50	832.00	46.00
131.00	15.00	38.00	0.80	0.50	0.20	542.00	78.00
166.00	18.00	50.00	1.10	0.70	0.30	903.00	107.00
197.00	47.00	87.00	1.10	#NULL!	#NULL!	1208.00	129.00
141.00	0.50	0.20	3.90	54.00	14.90	279.00	8.80
220.00	16.30	39.00	1.10	0.50	0.20	1100.00	128.00
203.00	10.00	67.00	1.40	1.20	0.50	751.00	76.00
132.00	3.10	35.00	1.10	0.60	0.30	651.00	56.00
149.00	93.00	21.00	1.30	0.60	0.30	651.00	136.00
238.00	7.50	40.00	1.00	1.20	0.60	651.00	35.00
737.00	12.00	36.00	1.00	2.30	1.30	339.00	114.00
173.00	2.60	40.00	1.30	0.70	0.30	123.00	65.00
129.00	1.10	0.60	3.30	#NULL!	#NULL!	604.00	9.00
256.00	17.00	95.00	2.10	0.40	0.20	597.00	32.00
125.00	16.00	66.00	1.90	0.60	0.30	965.00	303.00
300.00	50.00	28.00	1.00	0.40	0.20	472.00	96.00
134.00	3.00	29.00	0.90	0.50	0.20	596.00	82.00
144.00	5.00	0.20	4.00	516.00	53.00	710.00	7.80
292.00	5.60	94.00	8.60	0.50	0.20	352.00	46.00
10.00	9.80	81.00	1.50	0.50	0.20	765.00	171.00
173.00	1.70	21.00	1.30	0.50	0.20	760.00	157.00
1233.00	9.50	40.00	1.20	1.90	0.80	1455.00	98.00
157.00	8.00	69.00	1.90	0.70	0.30	1064.00	83.00
138.00	11.00	48.00	1.70	0.60	0.30	831.00	2484.00
72.00	9.00	0.40	3.30	1058.00	150.00	1019.00	9.50
331.00	0.50	73.00	5.70	0.50	0.20	721.00	88.00
#NULL!	1.00	36.00	1.10	#NULL!	#NULL!	380.00	78.00
316.00	0.70	0.20	4.00	140.00	15.00	643.00	8.00
111.00	0.60	0.30	3.90	63.00	17.00	536.00	7.80
149.00	15.30	27.00	1.10	0.70	0.40	486.00	103.00
222.00	9.60	20.00	0.90	0.70	0.40	565.00	160.00
364.00	0.30	0.12	4.50	216.00	24.00	535.00	7.80
179.00	11.00	26.00	1.10	1.20	0.60	377.00	78.00
294.00	12.00	38.00	1.10	0.90	0.40	372.00	160.00
109.00	0.50	0.20	4.20	67.00	13.00	536.00	9.00
228.00	11.00	23.00	0.90	0.60	0.30	689.00	80.00
75.00	0.40	0.20	3.30	91.00	11.00	630.00	10.00
149.00	7.30	17.00	0.80	0.60	0.20	859.00	85.00
285.00	17.00	49.00	1.10	0.50	0.20	757.00	85.00
115.00	3.00	#NULL!	3.00	75.00	8.00	421.00	10.00
257.00	10.00	58.00	1.40	1.40	0.70	1192.00	160.00
140.00	9.80	95.00	3.10	0.80	0.40	453.00	550.00
195.00	13.00	109.00	3.40	0.40	0.20	386.00	72.00
197.00	12.00	60.00	1.50	1.40	0.70	852.00	15.00
#NULL!	6.00	51.00	1.00	#NULL!	#NULL!	850.00	320.00
707.00	6.70	31.00	0.50	32.10	16.00	420.00	40.00
125.00	4.50	25.00	0.90	0.50	0.20	508.00	205.00
235.00	5.00	40.00	1.40	0.30	0.10	492.00	74.00

178.00	7.00	0.30	3.00	362.00	21.00	437.00	9.90
155.00	9.80	28.00	1.10	0.60	0.30	854.00	138.00
110.00	2.00	75.00	1.90	0.60	0.30	345.00	329.00
202.00	6.80	38.00	1.30	0.70	0.30	945.00	655.00
244.00	8.00	37.00	1.00	0.90	0.30	768.00	78.00
177.00	12.50	44.00	1.40	0.60	0.20	1313.00	241.00
133.00	8.90	165.00	1.50	1.40	0.70	536.00	60.00
190.00	2.10	57.00	0.90	0.50	0.20	548.00	44.00
193.00	5.70	19.00	1.20	0.50	0.20	537.00	141.00
414.00	9.80	81.00	1.60	0.60	0.40	214.00	212.00
170.00	8.80	64.00	1.50	0.80	0.40	230.00	360.00
143.00	13.20	31.00	1.00	1.20	0.50	785.00	68.00
#NULL!	28.00	189.00	3.20	3.20	1.20	714.00	120.00
97.00	7.90	36.00	1.20	0.50	0.20	135.00	35.00
#NULL!	0.50	20.00	0.90	#NULL!	#NULL!	279.00	114.00
143.00	22.00	35.00	0.90	1.80	0.90	570.00	89.00
179.00	9.80	32.00	1.10	0.90	0.40	453.00	179.00
232.00	4.70	46.00	1.50	0.60	0.30	929.00	354.00
222.00	4.80	60.00	1.80	0.60	0.30	1113.00	1310.00
246.00	3.00	78.00	1.90	#NULL!	#NULL!	513.00	97.00
150.00	9.80	64.00	1.40	0.90	0.50	466.00	954.00
116.00	2.30	100.00	1.70	0.50	0.20	193.00	26.00
227.00	7.00	39.00	1.10	1.00	0.50	639.00	132.00
187.00	12.00	136.00	1.90	1.10	0.50	1002.00	113.00
188.00	37.00	48.00	1.00	0.50	0.20	596.00	40.00
92.00	7.00	83.00	1.60	0.50	0.20	1137.00	1984.00
326.00	11.00	46.00	1.10	1.10	0.50	316.00	49.00
201.00	11.00	25.00	1.00	0.80	0.30	304.00	45.00
132.00	0.50	0.38	#NULL!	31.00	8.00	675.00	8.10
139.00	22.00	84.00	1.80	0.80	0.40	685.00	517.00
218.00	2.00	95.00	2.50	0.80	0.40	402.00	25.00

CKMB	Na	k	ca	Mg	Alb	Amylase	PH
2.00	2.90	155.87	#NULL!	#NULL!	#NULL!	#NULL!	7.48
2.00	140.00	4.20	9.30	2.00	4.30	#NULL!	7.44
14.00	3.90	29.00	100.00	95.00	30.00	51.00	7.35
22.00	143.00	5.00	7.50	2.20	2.00	#NULL!	7.39
2.00	2.50	156.00	#NULL!	#NULL!	#NULL!	#NULL!	7.35
1.80	#NULL!	73.00	#NULL!	#NULL!	#NULL!	#NULL!	7.44
34.00	136.00	3.80	9.50	1.90	3.30	#NULL!	6.00
9.00	135.00	4.60	10.60	1.90	1.70	#NULL!	7.43
10.00	141.00	4.30	9.50	1.80	#NULL!	41.00	7.31
2.30	3.30	75.00	#NULL!	#NULL!	#NULL!	#NULL!	7.38
8.00	144.00	3.90	9.00	#NULL!	#NULL!	54.00	7.18
10.00	4.80	3.00	#NULL!	#NULL!	#NULL!	#NULL!	7.42
45.00	139.00	3.80	8.50	1.90	4.60	#NULL!	7.29
9.00	137.00	4.30	8.20	#NULL!	2.40	#NULL!	7.39
7.00	141.00	3.50	8.50	1.90	1.90	#NULL!	7.30
130.00	133.00	3.60	9.50	1.90	4.00	#NULL!	7.43
1.90	3.30	10.00	#NULL!	#NULL!	#NULL!	#NULL!	7.25
15.00	2.80	95.00	#NULL!	#NULL!	#NULL!	#NULL!	8.00
85.00	140.00	3.40	8.60	#NULL!	2.20	#NULL!	7.46
21.00	139.00	4.00	#NULL!	2.20	#NULL!	#NULL!	7.28
36.00	141.00	3.70	9.50	1.90	3.90	#NULL!	7.27
45.00	136.00	3.70	8.40	1.90	3.30	#NULL!	7.27
2.20	3.00	6.00	#NULL!	#NULL!	#NULL!	#NULL!	7.35
17.00	140.00	3.70	8.70	#NULL!	#NULL!	#NULL!	7.42
1.80	3.40	29.00	#NULL!	#NULL!	#NULL!	#NULL!	7.38
10.00	141.00	3.80	9.50	2.20	3.80	#NULL!	7.37
55.00	136.00	3.60	9.00	2.10	#NULL!	#NULL!	7.40
14.00	145.00	4.60	#NULL!	1.80	#NULL!	#NULL!	7.34
2.00	4.20	86.00	96.00	271.00	49.00	-7.20	7.38
13.00	144.00	4.10	#NULL!	2.00	3.90	#NULL!	7.39
32.00	138.00	3.60	#NULL!	#NULL!	3.30	#NULL!	7.43
2.00	#NULL!	32.18	#NULL!	#NULL!	#NULL!	#NULL!	7.37
2.00	3.80	78.00	#NULL!	#NULL!	#NULL!	#NULL!	7.35
2.00	#NULL!	98.00	#NULL!	#NULL!	#NULL!	#NULL!	7.39
10.00	134.00	3.60	8.90	1.80	#NULL!	57.00	7.35
24.00	133.00	4.60	9.00	2.00	3.40	#NULL!	7.39
1.90	3.90	68.00	96.00	77.00	48.00	32.60	7.39
14.00	139.00	4.20	#NULL!	1.80	#NULL!	#NULL!	7.33
10.00	138.00	4.70	#NULL!	1.90	#NULL!	#NULL!	7.23
10.00	138.00	4.30	10.00	1.80	#NULL!	#NULL!	7.36
10.00	132.00	4.60	8.80	2.00	4.00	44.00	7.38
2.30	#NULL!	98.00	109.00	93.00	40.00	50.40	7.29
2.00	3.30	57.00	#NULL!	#NULL!	#NULL!	#NULL!	7.40
10.00	#NULL!	98.00	130.00	143.00	#NULL!	101.40	7.37
2.10	#NULL!	114.00	#NULL!	#NULL!	#NULL!	#NULL!	7.44
3.00	135.00	4.50	8.40	1.90	3.50	#NULL!	7.33
1.90	2.60	80.00	#NULL!	#NULL!	#NULL!	#NULL!	7.45
60.00	134.00	3.70	8.00	#NULL!	3.20	41.00	7.34
50.00	144.00	4.00	8.50	2.10	3.00	55.00	7.44
25.00	138.00	3.80	10.50	2.00	#NULL!	#NULL!	7.41
2.30	3.50	107.00	#NULL!	#NULL!	#NULL!	#NULL!	7.36
10.00	135.00	4.20	8.00	#NULL!	3.00	47.00	7.33

23.00	140.00	4.50	9.10	2.00	#NULL!	#NULL!	7.34
2.40	4.00	46.00	#NULL!	#NULL!	#NULL!	#NULL!	8.00
9.00	140.00	3.40	#NULL!	#NULL!	#NULL!	#NULL!	7.41
#NULL!	3.40	1.00	#NULL!	#NULL!	#NULL!	#NULL!	7.35
40.00	136.00	3.60	9.30	2.00	4.30	66.00	7.37
250.00	141.00	4.30	9.80	#NULL!	#NULL!	#NULL!	7.27
2.00	2.80	98.00	#NULL!	#NULL!	#NULL!	#NULL!	7.44
2.00	3.40	105.00	150.00	275.00	48.00	47.00	7.31
15.00	138.00	4.10	9.30	1.90	3.40	#NULL!	7.31
2.10	#NULL!	15.00	93.00	75.00	40.00	38.00	7.27
2.00	4.40	12.00	#NULL!	#NULL!	#NULL!	#NULL!	7.29
15.00	139.00	3.90	9.60	2.00	4.10	#NULL!	#NULL!
5.00	3.90	56.00	#NULL!	#NULL!	#NULL!	#NULL!	7.35
2.10	3.40	27.00	#NULL!	#NULL!	#NULL!	#NULL!	7.42
13.00	141.00	4.00	#NULL!	1.90	#NULL!	#NULL!	7.41
37.00	136.00	4.00	#NULL!	1.80	#NULL!	#NULL!	7.42
2.20	3.30	33.23	#NULL!	#NULL!	#NULL!	#NULL!	7.42
10.00	139.00	3.60	#NULL!	#NULL!	#NULL!	#NULL!	7.19
2.30	3.00	156.00	#NULL!	#NULL!	#NULL!	#NULL!	7.38
10.00	3.60	23.00	#NULL!	#NULL!	#NULL!	#NULL!	7.40
2.10	3.30	10.00	137.00	94.00	42.00	76.20	7.40
2.00	3.80	114.00	#NULL!	#NULL!	#NULL!	#NULL!	7.44
17.00	140.00	3.80	#NULL!	2.10	#NULL!	#NULL!	7.47
2.00	3.70	2.00	#NULL!	#NULL!	#NULL!	#NULL!	7.40
#NULL!	136.00	4.00	#NULL!	#NULL!	#NULL!	#NULL!	7.35
26.00	136.00	4.00	#NULL!	2.00	#NULL!	#NULL!	7.44
10.00	3.20	4.00	#NULL!	#NULL!	#NULL!	#NULL!	7.39
54.00	129.00	4.50	7.50	#NULL!	2.70	#NULL!	7.32
50.00	137.00	3.80	9.30	2.00	4.10	#NULL!	7.26
71.00	138.00	4.60	#NULL!	#NULL!	#NULL!	#NULL!	7.41
11.00	138.00	3.80	9.20	#NULL!	3.60	#NULL!	7.32
2.20	4.20	2.00	#NULL!	#NULL!	#NULL!	#NULL!	7.36
1.80	4.00	3.00	#NULL!	#NULL!	#NULL!	#NULL!	7.41
2.10	#NULL!	76.00	#NULL!	#NULL!	#NULL!	#NULL!	7.39
8.00	137.00	3.40	10.00	#NULL!	#NULL!	#NULL!	7.46
15.00	136.00	4.40	#NULL!	1.80	#NULL!	#NULL!	7.45
1.90	#NULL!	#NULL!	#NULL!	#NULL!	#NULL!	#NULL!	6.00
70.00	135.00	4.00	#NULL!	#NULL!	#NULL!	#NULL!	7.47
12.00	139.00	3.60	#NULL!	1.80	3.30	#NULL!	7.38
9.00	136.00	4.60	9.20	#NULL!	3.20	#NULL!	7.41
21.00	140.00	4.00	#NULL!	1.90	#NULL!	#NULL!	7.54
12.00	136.00	3.60	8.60	#NULL!	2.70	#NULL!	7.34
10.00	137.00	4.80	10.50	2.00	#NULL!	#NULL!	8.00
13.00	136.00	4.10	9.30	#NULL!	#NULL!	52.00	7.29
18.00	142.00	4.40	9.50	2.00	3.90	#NULL!	7.35
12.00	135.00	4.00	9.40	1.80	#NULL!	#NULL!	8.00
1.80	2.40	177.00	#NULL!	#NULL!	#NULL!	#NULL!	7.37
80.00	136.00	3.80	10.30	#NULL!	2.10	164.00	8.00
42.00	134.00	5.10	9.10	#NULL!	2.40	53.00	7.32
15.00	136.00	4.90	9.70	2.10	3.30	#NULL!	7.32
6.00	138.00	4.80	8.90	2.30	3.90	#NULL!	7.47
8.00	134.00	4.00	8.90	2.20	3.30	65.00	7.49
#NULL!	4.20	5.00	133.00	80.00	40.00	77.00	7.34

19.00	134.00	4.10	#NULL!	1.90	#NULL!	#NULL!	7.44
6.00	136.00	4.40	8.90	1.90	#NULL!	#NULL!	6.00
1.80	3.40	95.00	#NULL!	#NULL!	#NULL!	#NULL!	7.40
2.00	3.50	77.64	#NULL!	#NULL!	#NULL!	#NULL!	7.40
90.00	130.00	4.30	#NULL!	2.00	#NULL!	#NULL!	7.35
11.00	137.00	3.90	7.80	1.80	#NULL!	#NULL!	7.38
1.80	132.00	#NULL!	#NULL!	#NULL!	#NULL!	#NULL!	7.37
2.20	#NULL!	5.00	105.00	97.00	423.00	#NULL!	7.43
14.00	136.00	3.60	8.50	2.00	#NULL!	#NULL!	7.31
10.00	141.00	4.30	12.20	1.80	3.70	#NULL!	7.45
2.10	2.60	90.00	#NULL!	#NULL!	#NULL!	#NULL!	7.48
2.00	3.80	5.00	#NULL!	#NULL!	#NULL!	#NULL!	7.31
2.00	3.70	4.59	#NULL!	#NULL!	#NULL!	#NULL!	7.40
2.00	38.00	18.00	#NULL!	#NULL!	#NULL!	#NULL!	7.15
13.00	136.00	4.60	9.60	1.90	#NULL!	132.00	7.39
10.00	133.00	4.90	#NULL!	#NULL!	#NULL!	#NULL!	7.47
19.00	137.00	3.80	9.20	1.90	4.30	#NULL!	7.38
2.00	#NULL!	86.00	#NULL!	#NULL!	#NULL!	#NULL!	7.44
7.00	141.00	3.60	9.20	1.80	#NULL!	#NULL!	7.28
80.00	139.00	5.20	#NULL!	#NULL!	#NULL!	#NULL!	7.27
13.00	136.00	3.80	#NULL!	#NULL!	#NULL!	#NULL!	7.35
4.00	142.00	3.80	11.40	#NULL!	3.50	26.00	7.36
1.90	3.50	3.00	#NULL!	#NULL!	#NULL!	#NULL!	7.36
30.00	136.00	3.60	9.60	1.90	3.20	#NULL!	7.41
2.00	3.20	79.00	123.00	62.00	48.00	62.60	7.27
32.00	#NULL!	84.00	#NULL!	#NULL!	#NULL!	#NULL!	7.31
2.00	4.00	6.60	#NULL!	#NULL!	#NULL!	#NULL!	7.39
156.00	138.00	4.00	8.00	2.20	3.20	#NULL!	7.38
2.00	140.00	3.90	10.00	2.10	3.20	#NULL!	7.39
1.90	2.00	152.38	#NULL!	#NULL!	#NULL!	#NULL!	7.43
50.00	140.00	4.00	9.50	#NULL!	4.10	#NULL!	7.39
31.00	138.00	4.60	7.40	1.70	3.30	#NULL!	7.23
10.00	140.00	4.00	9.00	1.80	#NULL!	#NULL!	7.40
12.00	#NULL!	6.00	#NULL!	#NULL!	#NULL!	#NULL!	7.44
21.00	135.00	4.00	9.50	#NULL!	#NULL!	3.60	7.46
2.00	2.90	152.00	#NULL!	#NULL!	#NULL!	#NULL!	7.23
19.00	135.00	4.20	9.00	#NULL!	2.90	47.00	7.35
15.00	135.00	3.90	#NULL!	1.80	3.00	#NULL!	7.42
2.00	#NULL!	43.00	130.00	117.00	48.00	58.60	7.40
1.80	2.70	27.00	#NULL!	#NULL!	#NULL!	#NULL!	7.44
10.00	136.00	3.60	10.00	1.90	3.50	#NULL!	7.33
10.00	135.00	4.80	7.80	2.10	#NULL!	#NULL!	7.37
90.00	129.00	3.70	9.90	#NULL!	3.10	#NULL!	7.10
1.90	3.10	120.00	#NULL!	#NULL!	#NULL!	#NULL!	7.38
2.00	3.80	6.00	101.00	85.00	33.00	51.00	7.38
12.00	142.00	4.10	9.20	2.00	#NULL!	#NULL!	7.45
30.00	136.00	4.90	9.40	1.80	3.70	65.00	7.25
24.00	135.00	4.00	#NULL!	1.90	#NULL!	#NULL!	7.33
15.00	136.00	3.60	#NULL!	#NULL!	#NULL!	18.00	7.39
2.00	136.00	3.50	9.10	2.10	3.30	#NULL!	7.48
12.00	123.00	4.40	#NULL!	2.00	4.00	#NULL!	7.36
2.50	3.00	98.00	#NULL!	#NULL!	#NULL!	#NULL!	7.45
2.10	4.40	11.77	#NULL!	#NULL!	#NULL!	#NULL!	7.46

6.00	138.00	4.40	7.60	2.30	2.30	#NULL!	7.34
25.00	138.00	4.30	9.30	2.30	4.10	41.00	7.37
50.00	135.00	4.00	9.00	2.00	3.50	#NULL!	7.38
10.00	137.00	3.30	8.90	1.90	3.90	#NULL!	7.22
45.00	145.00	4.10	8.00	1.90	2.70	#NULL!	7.45
400.00	138.00	4.40	9.20	1.90	#NULL!	33.00	7.13
2.00	3.20	99.00	#NULL!	#NULL!	#NULL!	#NULL!	7.48
10.00	140.00	4.00	8.30	1.90	3.50	#NULL!	7.29
71.00	140.00	4.40	9.10	#NULL!	3.20	#NULL!	7.37
2.20	2.70	2.00	#NULL!	#NULL!	#NULL!	#NULL!	7.47
25.00	135.00	3.60	8.50	1.80	2.90	187.00	7.25
7.00	137.00	4.90	#NULL!	1.90	#NULL!	#NULL!	7.33
2.00	131.00	4.70	#NULL!	#NULL!	#NULL!	#NULL!	7.41
21.00	135.00	4.00	9.50	1.90	3.60	#NULL!	7.25
40.00	134.00	4.10	8.00	1.80	3.30	#NULL!	7.33
10.00	137.00	4.90	7.80	2.00	#NULL!	#NULL!	7.30
21.00	137.00	3.70	9.30	2.00	3.40	#NULL!	7.39
10.00	147.00	4.20	9.00	1.90	3.60	55.00	7.36
25.00	137.00	3.90	8.60	1.90	#NULL!	72.00	7.36
10.00	133.00	4.50	7.90	2.00	3.20	65.00	7.40
1.80	3.00	72.00	180.00	139.00	40.00	112.20	7.36
18.00	138.00	4.50	8.00	2.30	2.80	27.00	7.36
20.00	135.00	3.70	8.30	1.90	#NULL!	#NULL!	7.71
1.80	3.40	98.00	#NULL!	#NULL!	#NULL!	#NULL!	7.42
24.00	140.00	3.50	#NULL!	#NULL!	#NULL!	57.00	7.33
19.00	135.00	3.60	10.00	#NULL!	3.00	#NULL!	7.36
13.00	138.00	4.70	9.60	2.00	3.30	23.00	7.33
16.00	144.00	3.50	8.20	1.90	2.90	42.00	7.44
2.00	2.80	98.00	#NULL!	#NULL!	#NULL!	#NULL!	7.34
12.00	143.00	4.40	8.10	#NULL!	3.30	#NULL!	7.27
18.00	134.00	4.10	8.50	#NULL!	2.60	#NULL!	7.44
5.00	134.00	7.50	7.60	#NULL!	#NULL!	#NULL!	7.20
2.00	136.00	5.10	8.40	1.90	2.90	#NULL!	7.34
48.00	135.00	4.00	#NULL!	1.90	#NULL!	#NULL!	7.43
10.00	136.00	3.80	9.90	2.00	4.50	#NULL!	7.40
20.00	130.00	4.80	9.50	2.00	#NULL!	39.00	7.45
35.00	138.00	3.90	9.50	2.00	#NULL!	#NULL!	7.43
80.00	136.00	4.30	8.90	#NULL!	3.40	99.00	7.44
1.90	2.00	134.00	#NULL!	#NULL!	#NULL!	#NULL!	7.40
3.00	3.40	82.00	#NULL!	#NULL!	#NULL!	#NULL!	7.34
87.00	136.00	4.70	8.60	1.90	3.30	#NULL!	7.30
8.00	145.00	3.40	8.00	2.20	2.50	#NULL!	7.30
8.00	141.00	3.70	#NULL!	1.90	#NULL!	#NULL!	7.37
2.00	#NULL!	56.17	#NULL!	#NULL!	#NULL!	#NULL!	7.38
40.00	143.00	4.00	#NULL!	#NULL!	#NULL!	#NULL!	7.41
131.00	42.00	3.40	9.20	1.80	3.50	#NULL!	7.37
35.00	138.00	4.60	#NULL!	2.00	#NULL!	#NULL!	7.36
25.00	133.00	4.90	9.00	1.90	#NULL!	#NULL!	7.57
85.00	135.00	3.80	9.70	2.00	#NULL!	#NULL!	7.42
9.00	137.00	3.70	9.10	2.10	#NULL!	#NULL!	7.40
1.90	3.40	113.00	#NULL!	#NULL!	#NULL!	#NULL!	7.45
32.00	133.00	5.00	9.60	2.00	3.90	49.00	7.22
3.00	#NULL!	135.00	#NULL!	#NULL!	#NULL!	#NULL!	7.48

12.00	137.00	4.20	8.80	1.80	#NULL!	#NULL!	7.39
45.00	133.00	4.60	7.90	2.10	#NULL!	#NULL!	7.36
24.00	141.00	4.00	8.40	1.80	3.70	98.00	7.30
18.00	139.00	3.70	9.90	#NULL!	4.00	66.00	7.33
20.00	136.00	3.70	9.30	2.00	3.30	85.00	7.36
10.00	140.00	4.00	9.00	1.90	#NULL!	#NULL!	7.34
3.00	140.00	4.20	#NULL!	2.00	#NULL!	#NULL!	7.41
2.00	138.00	4.10	#NULL!	1.90	3.10	#NULL!	7.28
14.00	139.00	4.20	8.40	#NULL!	3.30	22.00	7.40
1.80	3.30	24.59	#NULL!	#NULL!	#NULL!	#NULL!	7.41
12.00	139.00	4.70	#NULL!	#NULL!	#NULL!	42.00	7.33
10.00	136.00	3.60	#NULL!	1.80	#NULL!	#NULL!	7.35
25.00	130.00	4.90	#NULL!	#NULL!	#NULL!	#NULL!	7.39
13.00	134.00	4.40	8.50	1.90	#NULL!	#NULL!	7.32
10.00	145.00	3.50	8.50	1.90	2.60	#NULL!	7.34
15.00	136.00	3.60	9.70	2.00	3.40	61.00	7.50
8.00	137.00	5.10	#NULL!	2.10	#NULL!	#NULL!	7.42
2.00	#NULL!	46.50	#NULL!	#NULL!	#NULL!	#NULL!	7.42
10.00	139.00	3.90	9.60	2.00	#NULL!	#NULL!	7.32
31.00	137.00	4.50	8.70	1.80	2.90	#NULL!	7.33
10.00	139.00	3.80	7.90	2.00	3.40	135.00	7.36
2.00	134.00	4.20	8.20	2.20	3.40	105.00	7.42
2.00	3.10	65.00	#NULL!	#NULL!	#NULL!	#NULL!	7.41
8.00	139.00	3.90	8.30	2.10	2.50	65.00	7.27
85.00	135.00	4.90	9.50	1.80	3.40	#NULL!	7.44
15.00	129.00	4.80	#NULL!	#NULL!	#NULL!	#NULL!	7.27
20.00	139.00	5.50	#NULL!	#NULL!	5.80	#NULL!	7.34
8.00	137.00	5.00	8.00	2.30	3.30	89.00	7.12
250.00	136.00	3.70	9.10	2.00	#NULL!	#NULL!	7.36
2.30	3.00	160.00	#NULL!	#NULL!	#NULL!	#NULL!	7.42
21.00	138.00	5.50	8.50	2.30	3.70	#NULL!	7.31
16.00	133.00	#NULL!	#NULL!	#NULL!	#NULL!	#NULL!	7.26
2.00	2.90	131.00	#NULL!	#NULL!	#NULL!	#NULL!	7.41
1.90	3.20	17.00	#NULL!	#NULL!	#NULL!	#NULL!	7.37
10.00	134.00	4.20	#NULL!	2.00	#NULL!	#NULL!	7.27
15.00	130.00	4.60	9.00	#NULL!	3.30	#NULL!	7.40
2.00	3.30	76.57	#NULL!	#NULL!	#NULL!	#NULL!	7.45
10.00	137.00	4.60	9.70	2.00	2.60	#NULL!	7.32
12.00	140.00	4.00	10.00	1.80	4.00	#NULL!	7.40
2.00	3.70	6.00	#NULL!	#NULL!	#NULL!	#NULL!	7.35
15.00	145.00	4.50	#NULL!	2.00	#NULL!	#NULL!	#NULL!
2.30	3.00	151.00	#NULL!	#NULL!	#NULL!	#NULL!	7.10
10.00	134.00	3.90	8.50	2.00	3.80	#NULL!	7.31
16.00	136.00	5.00	9.10	2.10	3.00	#NULL!	7.37
2.00	3.30	7.40	#NULL!	#NULL!	#NULL!	#NULL!	7.37
20.00	136.00	3.80	9.10	2.30	3.40	#NULL!	7.34
200.00	142.00	3.60	8.40	1.80	3.20	100.00	7.07
9.00	132.00	5.10	9.80	2.20	3.60	105.00	7.27
2.00	139.00	3.70	#NULL!	#NULL!	#NULL!	#NULL!	7.45
15.00	141.00	4.50	9.10	1.80	3.70	110.00	7.35
2.00	141.00	3.80	8.20	1.80	2.20	51.00	7.11
98.00	134.00	3.70	9.00	2.00	#NULL!	#NULL!	7.34
16.00	140.00	4.00	#NULL!	1.90	#NULL!	#NULL!	7.46

2.00	4.50	88.00	#NULL!	#NULL!	#NULL!	#NULL!	7.36
40.00	134.00	3.80	9.80	#NULL!	3.70	#NULL!	7.34
150.00	136.00	3.60	8.60	2.00	#NULL!	#NULL!	7.31
34.00	129.00	4.90	#NULL!	1.90	#NULL!	#NULL!	7.50
10.00	140.00	4.90	9.80	2.00	3.80	42.00	7.43
26.00	136.00	3.60	9.00	1.90	3.20	#NULL!	7.23
12.00	135.00	5.20	9.80	1.80	3.00	78.00	7.34
5.00	135.00	5.20	#NULL!	1.90	3.30	#NULL!	7.41
42.00	140.00	3.80	9.40	1.90	#NULL!	#NULL!	7.39
2.00	140.00	4.00	8.90	2.00	2.90	#NULL!	7.36
49.00	135.00	4.10	#NULL!	#NULL!	#NULL!	#NULL!	7.43
9.00	132.00	5.10	8.60	#NULL!	3.10	66.00	7.42
54.00	144.00	5.30	9.30	1.80	2.00	#NULL!	7.27
2.00	136.00	4.10	#NULL!	#NULL!	#NULL!	#NULL!	7.35
10.00	135.00	3.60	9.00	1.90	2.10	#NULL!	7.41
9.00	139.00	3.20	8.90	2.00	2.90	#NULL!	7.33
70.00	130.00	3.90	8.50	2.00	3.00	22.00	7.43
50.00	139.00	4.70	9.00	2.00	#NULL!	72.00	7.35
210.00	140.00	4.00	10.00	#NULL!	#NULL!	40.00	7.35
26.00	140.00	6.00	#NULL!	#NULL!	3.10	#NULL!	7.15
190.00	140.00	3.60	8.90	1.80	3.60	81.00	7.42
8.00	133.00	6.50	8.30	1.90	2.10	#NULL!	7.38
14.00	140.00	4.10	10.00	1.80	3.80	#NULL!	7.36
123.00	136.00	2.80	#NULL!	#NULL!	#NULL!	#NULL!	7.46
10.00	140.00	4.20	8.60	2.10	3.00	58.00	7.51
68.00	140.00	3.40	#NULL!	2.00	#NULL!	#NULL!	7.49
11.00	138.00	4.10	10.10	1.90	2.90	38.00	7.29
12.00	137.00	3.10	7.50	2.00	2.90	#NULL!	7.44
74.00	165.00	58.09	#NULL!	#NULL!	#NULL!	#NULL!	7.53
57.00	131.00	4.90	7.50	2.00	3.40	82.00	7.30
14.00	140.00	4.40	8.50	2.00	#NULL!	#NULL!	7.42

PCO2	BE	BEecf	BB	HCO3	PO2	O2sat	Ferritin
34.60	2.40	2.00	50.40	25.40	59.60	92.60	347.40
45.10	4.70	5.50	52.70	29.70	57.80	21.00	405.00
45.20	2.80	2.70	48.80	17.30	37.30	65.25	188.30
33.10	-4.10	-5.10	43.90	19.70	24.90	43.30	372.00
42.80	-2.50	-2.50	43.60	23.40	32.00	58.00	65.90
37.80	1.30	1.10	49.30	25.20	22.80	43.30	154.00
41.20	2.10	2.20	51.00	25.10	58.50	77.10	451.00
30.50	-3.30	-4.60	50.00	19.70	39.70	76.20	456.00
62.90	2.70	4.60	38.20	30.80	26.30	42.50	140.00
36.20	-3.60	-4.30	44.40	20.80	21.50	87.00	397.20
36.90	-13.80	-14.80	34.20	13.50	31.40	47.70	207.00
42.80	2.80	3.20	50.80	27.60	24.60	46.00	276.50
50.60	-3.30	-2.70	42.60	24.10	36.00	61.30	499.50
31.20	-5.00	-6.20	43.00	18.60	59.10	89.60	198.00
39.70	-6.60	-7.10	41.40	19.20	76.20	93.00	208.00
36.80	-0.10	-0.50	47.80	23.70	61.70	91.90	254.00
68.70	-0.10	1.90	47.90	29.10	33.40	52.80	235.00
38.50	2.10	6.00	47.60	20.10	42.50	68.45	219.00
31.80	-0.60	-1.50	49.80	22.20	93.10	47.60	157.00
62.30	3.00	1.60	48.00	33.30	17.60	71.20	315.00
38.50	-8.90	-9.60	39.10	17.20	24.50	68.30	680.00
44.50	-7.20	-7.40	41.50	19.60	26.80	42.30	86.00
31.20	-7.30	-8.70	40.70	16.80	34.80	64.10	102.60
31.40	-3.80	-5.00	44.20	19.50	35.00	68.00	115.00
39.60	-2.10	-2.40	45.90	22.70	43.80	78.10	198.00
42.10	-1.70	-1.80	46.30	23.50	18.40	28.20	175.00
24.20	-8.10	-10.30	39.90	14.50	54.50	89.20	385.00
60.60	3.90	5.80	51.90	31.60	21.70	33.80	450.00
47.90	1.90	2.70	49.90	27.80	19.40	58.65	721.00
46.40	1.80	2.40	49.80	27.40	37.40	70.80	560.00
45.10	4.00	4.80	52.00	29.10	38.80	75.70	140.00
40.10	-2.30	-2.60	45.60	22.60	38.50	70.60	390.00
48.70	0.00	0.60	47.90	26.20	36.70	66.70	466.50
52.70	4.40	5.80	52.40	30.80	19.30	29.40	512.00
45.70	-1.30	-1.00	46.70	24.60	36.30	65.10	371.00
44.70	0.50	0.90	48.50	25.90	25.60	46.10	388.40
38.90	-1.80	-2.10	46.10	22.80	112.10	98.20	166.00
57.50	2.70	4.20	50.70	30.00	29.60	51.20	136.00
64.80	-2.90	-2.50	37.00	25.10	42.90	55.17	254.00
51.20	-3.50	-3.80	44.50	21.70	19.30	63.15	295.00
39.30	-2.00	-2.40	46.00	22.70	25.10	65.30	400.00
62.90	0.70	2.30	48.70	29.00	40.40	66.80	312.10
37.40	-2.00	-2.50	46.00	22.30	30.90	58.60	530.00
46.20	0.40	0.90	48.40	26.10	20.80	61.60	706.80
46.00	5.40	6.30	53.40	30.40	32.20	62.90	118.00
46.50	-2.70	-2.50	45.30	23.50	25.80	43.20	360.00
23.50	-5.80	-8.00	42.20	15.90	56.70	91.60	412.00
47.70	-1.00	-0.60	47.00	25.10	20.20	71.20	750.00
31.40	-2.30	-3.40	45.60	20.70	69.90	94.30	97.00
33.10	-3.60	-4.60	44.40	20.10	33.50	64.50	280.00
36.10	-5.20	-6.00	42.50	19.50	42.20	75.10	287.80
33.30	-7.80	-9.00	40.20	16.90	35.80	64.10	463.00

39.40	-4.70	-5.20	43.30	20.60	41.10	71.10	745.00
22.50	0.40	1.20	35.50	18.20	56.40	77.52	380.00
28.80	-5.30	-6.90	42.70	17.70	45.00	41.80	460.00
28.50	-8.50	-10.20	39.50	15.40	39.50	72.00	261.00
45.40	-0.40	-0.10	47.60	25.20	39.30	70.80	430.00
57.90	-2.20	-1.00	45.80	25.80	20.70	25.50	368.00
31.50	-1.70	-2.70	53.20	21.60	100.00	60.10	712.00
43.60	-4.70	-4.70	43.30	21.40	37.98	64.90	446.60
35.40	-7.90	-8.90	40.10	17.30	51.20	42.90	375.00
55.30	-3.00	-2.10	41.30	25.10	29.00	45.20	407.50
54.20	-1.00	0.00	21.00	26.60	22.00	75.20	365.00
35.20	2.40	0.80	37.20	12.50	21.00	78.10	630.00
44.80	-2.20	-2.00	45.80	23.60	39.50	70.00	241.00
33.70	-2.70	-3.70	45.30	20.90	52.70	88.00	354.00
45.80	2.90	3.50	50.90	28.20	27.00	62.51	378.00
34.10	-2.00	-2.80	45.90	21.60	48.90	84.80	514.00
41.30	1.10	1.10	49.10	25.70	26.10	49.00	380.00
54.00	-8.20	-7.80	39.80	20.30	36.00	55.20	295.00
38.60	-2.90	-3.40	45.10	21.80	29.30	73.50	386.90
45.60	2.10	2.70	49.00	27.50	37.80	72.10	432.00
36.50	-2.30	-2.90	45.70	21.90	25.80	37.90	414.50
40.30	2.20	2.20	50.20	26.40	26.70	51.90	469.00
32.20	-0.30	-1.20	47.70	22.50	27.10	75.53	765.00
33.30	-3.70	-4.70	44.30	20.10	38.30	72.40	56.20
25.80	-9.80	-11.60	44.50	14.20	43.00	77.40	198.00
42.00	3.00	3.30	51.00	27.50	24.20	75.40	72.00
38.80	-1.40	-1.60	46.80	23.50	47.00	56.30	208.90
47.90	-2.30	-2.00	45.70	24.10	23.90	88.90	170.00
37.40	-9.80	-10.70	38.20	16.30	16.30	19.40	354.00
41.60	0.90	1.00	48.90	25.60	36.40	69.10	430.00
37.80	-6.00	-6.70	45.50	19.30	26.60	45.80	421.00
45.80	-1.00	-0.70	47.00	24.80	25.20	43.60	311.30
38.20	-1.00	-1.40	47.00	23.30	31.20	59.70	395.00
38.60	-13.20	-14.00	34.80	14.20	109.90	96.90	245.00
31.00	-1.10	-2.20	46.80	21.60	54.00	89.50	378.00
34.60	0.30	-0.20	48.30	23.70	55.80	90.30	402.00
25.20	2.40	1.70	43.00	18.20	20.10	55.13	548.00
36.70	2.40	2.10	50.40	25.90	41.40	80.60	367.00
44.70	0.60	1.00	48.60	26.10	31.10	58.80	365.00
64.80	12.60	15.80	60.60	40.30	40.00	73.30	521.00
35.00	6.50	6.50	54.50	29.00	50.80	90.70	167.00
35.40	-6.20	-7.10	41.80	18.60	28.40	50.60	65.00
42.30	2.80	6.10	50.30	18.50	25.40	78.10	345.00
38.00	-8.70	-6.10	39.70	17.60	48.90	59.64	198.00
42.60	-2.50	-2.60	45.50	23.00	40.90	72.60	187.00
28.20	2.40	5.00	48.50	27.40	32.00	77.10	321.00
26.40	-8.30	-10.20	39.70	15.00	55.30	80.65	705.00
28.40	2.40	3.10	51.20	28.20	20.40	99.10	286.00
25.30	-11.30	-13.30	28.90	12.70	44.00	73.00	560.00
44.60	-3.70	-3.60	44.30	22.40	13.30	73.15	702.00
35.70	1.80	1.40	49.80	21.00	35.40	72.60	308.00
29.40	-0.60	-1.90	47.40	21.50	26.50	55.00	504.00
39.70	-4.20	-4.60	43.70	21.10	36.70	65.40	465.00

46.60	5.80	6.90	53.80	31.00	30.50	62.30	380.00
38.50	2.50	8.20	33.70	35.20	31.20	72.10	257.00
24.10	-8.10	-10.30	39.90	14.50	46.00	82.80	320.00
32.60	-3.40	-4.40	49.10	20.50	60.00	91.10	130.00
38.60	-4.30	-4.90	39.20	20.70	28.00	50.10	512.00
42.30	-0.70	-0.60	47.20	24.40	37.70	70.20	394.00
32.70	-5.90	-7.10	42.10	18.20	29.80	55.10	43.70
28.20	-4.30	-5.90	43.70	18.30	23.10	44.30	201.30
59.50	1.10	2.60	49.10	28.90	49.50	79.70	418.00
42.00	4.40	4.90	52.40	28.80	38.90	77.30	354.00
23.00	-4.60	-6.90	43.40	16.60	49.50	88.60	106.00
57.00	0.60	1.80	48.60	28.00	18.90	66.30	421.60
41.70	0.50	0.60	48.50	25.30	20.80	35.90	432.00
34.10	-0.80	-1.50	47.10	22.60	27.00	52.50	451.10
24.30	-8.50	-10.70	39.50	14.30	28.10	54.10	370.00
35.70	1.80	1.40	49.80	25.10	23.70	46.80	205.00
47.60	1.60	2.30	49.60	27.40	29.00	53.40	370.00
45.90	5.70	6.70	53.70	30.80	28.50	58.10	320.00
40.20	-7.60	-8.20	40.40	18.50	40.10	68.00	365.00
34.70	-10.40	-11.50	37.60	15.40	41.40	69.50	260.00
54.20	1.80	3.00	49.80	28.60	59.60	88.90	364.00
34.50	-5.40	-6.40	42.60	19.00	88.90	97.88	116.00
37.60	-4.40	-5.00	43.60	20.50	38.40	69.60	563.00
43.90	2.40	2.80	50.40	27.40	36.90	71.80	265.00
46.50	-6.10	-6.10	41.80	20.80	39.20	64.00	572.00
45.10	-3.90	-3.80	40.60	22.70	27.00	44.20	386.70
40.80	-0.60	-0.70	47.40	24.20	23.20	65.21	158.50
53.50	4.30	5.70	52.30	30.80	23.50	62.15	613.90
21.50	-9.80	-12.40	38.20	12.60	46.70	43.25	365.00
32.70	-2.00	-2.00	71.40	21.70	36.00	71.40	438.00
36.60	-2.80	-3.50	45.20	21.50	22.80	64.50	351.00
28.20	-14.50	-16.20	33.50	11.40	20.30	67.80	417.00
46.40	2.60	3.30	50.60	28.10	19.90	21.30	475.00
44.30	4.80	5.50	52.80	29.60	39.10	74.65	142.60
39.90	3.80	4.00	51.80	27.80	39.40	71.68	362.00
44.40	-9.30	-9.50	38.60	18.00	49.20	71.30	750.00
40.10	-3.60	-4.00	44.40	21.60	46.10	79.40	204.00
44.00	2.70	3.20	38.20	27.70	47.70	83.60	437.00
41.40	0.30	0.30	44.60	25.10	35.10	66.40	146.90
26.40	-5.20	-7.10	42.80	17.20	28.10	56.30	298.00
37.50	-6.20	-7.00	39.50	19.00	51.30	83.60	116.00
32.80	-5.30	-6.50	42.70	18.70	28.30	52.60	513.00
54.50	-13.30	-13.10	49.50	16.50	35.80	49.80	135.00
45.00	0.70	1.10	48.70	26.10	23.90	72.20	93.00
39.40	-2.10	-2.40	45.90	22.70	28.80	53.10	240.80
319.00	-1.20	-2.20	46.80	21.70	39.50	77.00	702.00
32.50	-12.00	-13.30	36.00	13.90	22.50	73.40	470.00
51.80	-0.20	0.60	47.80	26.50	43.40	74.30	532.60
38.10	-2.30	-2.80	45.70	22.20	36.70	68.50	215.00
36.40	3.30	3.10	51.30	26.60	40.80	30.90	182.00
31.60	-6.40	-7.70	41.50	17.60	36.90	66.90	730.00
38.10	1.70	1.60	49.70	25.60	33.40	67.70	416.10
28.70	-2.40	-3.90	45.60	19.90	63.30	33.70	178.00

49.00	-0.90	-0.30	47.10	25.50	27.90	48.30	53.00
34.90	-5.10	-6.00	48.80	19.40	36.00	66.50	92.00
35.70	-3.80	-4.60	44.20	20.50	29.50	55.10	116.00
71.80	-1.50	0.40	46.50	28.20	35.00	53.70	378.00
34.80	0.30	-0.30	48.30	23.60	24.80	48.80	261.00
42.20	-15.20	-15.80	32.80	13.50	31.30	44.20	212.00
25.90	-2.80	-4.50	44.60	19.20	43.00	65.25	376.10
33.70	-9.30	-10.50	38.70	16.00	73.70	43.60	168.00
29.40	-7.00	-8.50	40.90	16.60	26.60	45.30	210.00
23.90	-4.10	-6.30	43.90	17.20	34.90	72.20	230.00
78.00	3.30	6.10	51.30	33.30	20.50	26.60	312.00
30.40	-8.90	-10.40	39.10	15.60	44.30	77.10	281.00
29.40	-5.00	-6.50	43.00	18.10	47.50	84.20	413.00
46.10	-7.50	-7.50	40.40	19.70	64.60	66.21	340.00
53.40	0.00	0.90	46.30	26.90	30.30	52.10	542.00
38.40	-7.20	-7.90	33.50	18.50	36.10	62.80	395.00
35.10	-3.00	-3.70	39.10	21.40	30.00	57.60	185.60
30.70	-7.00	-8.40	50.00	17.00	23.50	41.40	320.00
32.30	-6.10	-7.30	41.90	18.00	19.50	31.70	352.00
35.00	-2.80	-3.50	45.20	21.20	68.70	93.30	528.00
61.20	6.00	8.10	54.00	33.50	28.40	49.60	835.20
34.50	-5.60	-6.60	42.70	18.80	24.50	43.10	113.00
15.70	2.60	-0.70	50.60	19.10	122.80	99.20	765.00
39.80	1.00	1.00	49.00	25.40	43.90	81.00	142.10
43.20	-3.60	-3.60	51.30	22.20	35.30	66.00	367.00
39.80	-3.50	-3.90	44.50	21.60	38.60	69.70	421.00
55.10	0.70	1.80	48.70	27.80	37.30	64.70	184.00
35.60	-0.20	-0.80	47.80	23.40	49.40	86.30	356.00
47.90	-0.50	0.10	46.00	26.00	26.00	68.90	512.00
25.40	-13.40	-15.40	34.60	11.40	27.70	46.80	624.00
33.40	-1.30	-2.20	46.70	22.00	38.60	74.80	402.00
24.00	-17.00	-18.90	30.90	9.10	126.90	40.27	379.30
27.90	-9.40	-11.20	38.60	14.60	37.00	67.50	480.00
33.20	-2.20	-3.20	45.80	21.20	35.60	69.30	365.00
27.10	-6.30	-8.10	41.70	10.60	44.70	81.50	172.30
34.00	0.10	-0.50	48.10	23.30	56.20	90.50	210.00
44.00	3.50	4.00	51.50	28.40	33.90	67.70	715.00
34.70	-0.70	-1.30	47.30	22.80	42.20	79.60	391.00
47.00	3.10	3.90	51.00	28.60	29.50	56.00	264.00
45.50	-1.90	-1.70	46.00	24.00	28.60	38.80	365.00
53.20	-2.00	-1.30	46.00	25.20	24.50	38.50	385.00
74.20	5.70	8.60	53.70	35.10	21.70	52.41	175.00
43.60	-0.60	-0.40	47.40	24.80	34.60	64.40	351.00
45.30	0.60	70.50	46.80	26.10	37.80	70.50	358.00
37.90	-0.50	-0.80	47.40	23.70	39.00	74.30	352.00
35.00	-4.30	-5.20	43.60	20.00	92.90	38.10	91.00
33.20	-6.00	-7.20	48.50	18.20	41.10	74.30	614.00
24.50	1.40	-0.40	49.40	21.70	27.60	63.00	980.00
32.70	-3.10	-4.10	44.90	20.40	53.70	88.70	354.00
28.30	-6.20	-7.80	31.80	17.00	30.70	59.40	298.00
25.60	-4.80	-6.80	43.20	17.20	38.30	75.70	394.50
45.80	-9.40	-9.60	38.50	18.10	59.80	83.00	375.00
20.70	-5.80	-8.40	42.20	15.00	30.10	64.00	250.00

43.00	0.30	0.50	48.30	25.50	42.30	77.40	289.00
34.00	-5.70	-6.80	55.20	18.70	26.90	48.50	211.00
37.60	-7.70	-8.50	40.30	17.90	21.60	33.30	258.00
43.70	-3.00	-3.10	45.00	22.70	114.00	97.80	369.00
38.50	-3.70	-4.30	44.30	21.10	30.40	55.40	142.00
39.10	-5.00	-5.60	39.40	20.20	32.10	57.30	152.00
39.00	-0.10	-0.30	47.80	24.20	40.00	75.70	351.00
51.60	-3.60	-3.10	44.40	23.60	33.50	55.80	212.00
42.00	0.40	0.50	48.40	25.30	39.00	73.60	285.00
43.60	2.20	2.60	50.00	27.20	35.60	69.40	470.00
40.90	-44.90	-5.30	43.10	20.70	29.60	51.60	552.00
22.50	-11.00	-13.40	28.60	12.10	105.70	37.70	436.00
43.70	0.20	0.40	48.20	25.40	24.50	43.70	245.50
27.00	-10.60	-12.50	37.40	13.50	38.40	68.80	95.00
51.50	0.20	1.00	48.20	26.80	35.60	62.80	398.00
22.20	-0.90	-3.20	47.10	19.10	45.90	28.50	375.00
39.00	0.60	0.50	48.60	24.90	63.10	42.30	400.00
37.50	-0.80	-1.20	47.20	23.30	25.00	77.50	236.00
47.70	-2.70	-2.40	53.30	23.70	31.20	53.90	462.00
27.60	-10.30	-12.10	39.50	13.90	40.10	71.60	489.00
43.70	-1.10	-1.00	46.80	24.30	38.10	69.70	356.00
45.00	3.60	4.30	51.60	28.60	35.30	67.70	140.00
29.80	-4.90	-6.40	43.10	18.30	38.50	73.50	726.00
35.80	-9.70	-10.80	38.30	16.10	41.30	69.50	211.00
28.90	-3.70	-5.10	28.50	19.00	65.20	33.00	353.00
54.70	-3.10	-2.30	-44.90	24.50	20.30	28.00	370.00
49.80	-0.10	0.50	47.80	26.30	65.70	71.20	385.00
80.70	2.40	6.10	39.50	25.60	29.90	88.10	101.00
38.30	-3.80	-4.30	44.20	21.10	25.30	44.80	502.00
41.00	1.70	1.90	50.40	26.60	32.00	62.70	290.60
43.50	-4.60	-4.80	43.40	21.40	26.00	43.40	345.00
52.00	-5.10	-4.60	42.90	22.50	37.20	60.80	380.00
54.50	2.00	2.40	50.00	27.10	44.90	65.34	744.30
47.80	1.10	1.70	49.10	26.90	27.80	70.10	405.10
48.10	-5.60	-5.50	42.40	21.40	29.80	48.50	395.00
35.20	-3.20	-4.00	44.80	20.90	37.90	71.20	450.00
27.20	-3.60	-5.10	44.00	19.00	95.00	97.60	76.50
36.40	-6.70	-7.60	41.30	18.40	23.40	68.60	725.40
40.70	0.00	0.00	48.00	24.80	23.50	61.00	312.00
29.90	-8.10	-9.70	39.90	16.00	33.90	62.30	113.60
38.40	2.40	2.50	42.20	28.50	51.50	75.20	465.00
50.90	-14.20	-14.30	33.80	15.40	40.80	57.80	250.00
35.60	-7.80	-8.70	-7.80	17.50	41.80	72.30	365.00
30.10	-6.50	-8.00	52.20	17.10	40.80	75.00	370.00
40.80	-2.20	-2.50	45.80	22.60	33.90	62.40	365.00
36.10	-6.00	-6.90	45.90	18.00	17.00	24.00	165.00
79.10	-9.80	-8.10	38.20	22.10	54.90	72.30	440.00
89.10	8.90	12.90	56.90	39.80	26.50	89.80	20.30
32.20	-1.00	-2.00	46.90	21.90	36.30	72.60	356.00
31.80	-7.60	-8.90	40.40	16.80	17.70	26.30	98.00
39.00	-16.30	-17.10	28.20	12.20	48.20	70.00	202.00
44.90	-2.70	-2.60	45.30	23.20	27.70	48.00	331.00
44.80	6.50	7.50	54.50	31.30	50.30	58.25	618.00

50.40	2.10	3.10	50.10	28.40	20.60	31.30	435.00
45.90	-1.80	-1.50	46.20	24.20	21.60	34.70	95.00
28.40	-10.80	-12.60	37.20	13.70	35.90	63.80	173.00
24.60	-2.10	-4.00	45.90	19.00	40.30	81.40	555.60
43.70	3.90	4.50	51.90	28.70	35.90	71.80	432.00
57.10	-5.30	-4.60	42.70	23.00	23.00	81.90	253.00
21.40	-12.10	-14.60	35.90	11.20	39.80	98.80	315.00
30.30	-4.30	-5.60	43.60	18.90	81.60	45.90	504.00
41.80	-0.30	-0.20	47.60	24.70	28.30	52.30	132.00
38.00	-4.30	-4.90	43.70	20.60	20.20	64.92	541.00
22.50	-7.30	-9.70	40.70	14.60	31.90	64.50	375.00
41.20	1.60	1.70	49.60	26.10	27.40	52.50	461.00
41.10	-8.10	-8.50	39.90	18.30	26.30	37.10	435.00
25.30	-10.00	-12.10	38.00	13.50	36.30	67.20	82.00
40.80	0.70	0.70	48.70	25.30	47.00	81.10	345.00
43.70	-3.40	-3.50	44.60	22.40	28.20	49.00	364.00
31.20	-3.10	-4.30	44.90	20.00	32.90	64.90	167.00
38.00	-5.00	-5.60	43.00	20.10	40.10	71.50	195.00
35.40	-5.50	-6.40	42.40	19.10	94.90	96.70	342.00
48.60	-12.50	-12.60	35.50	16.30	19.90	82.50	460.00
41.80	2.10	2.40	50.10	26.80	37.60	73.40	362.00
42.40	-0.90	-0.80	47.00	24.30	42.80	77.00	351.00
29.60	-7.80	-9.40	40.20	16.10	39.50	72.10	178.00
34.50	0.70	0.10	48.70	24.00	30.30	62.30	540.00
24.20	-2.20	-4.20	45.80	18.80	40.70	52.50	94.00
28.80	-0.30	-1.50	46.00	22.00	32.00	68.60	213.00
32.50	-9.90	-11.20	38.10	15.30	40.50	70.00	388.00
41.30	3.00	3.20	51.00	27.30	34.70	67.90	513.00
25.10	-0.40	-2.30	47.60	20.40	43.00	85.00	195.00
36.80	-8.10	-9.00	39.90	17.50	33.60	58.20	132.00
29.70	-3.90	-5.30	44.00	19.00	79.20	95.70	87.00

vitD	nanelavasl	nanebarba	nanesanga	nanetaftoo	nanebaget	berenj	makaroni
25.00	11.00	160.00	8.55	4.93	1.88	71.43	4.67
14.50	0.00	0.00	55.71	0.00	0.27	125.00	2.33
44.70	220.00	20.00	27.86	0.00	70.00	214.29	2.33
26.40	15.71	0.00	26.00	0.00	0.00	125.00	3.50
17.50	0.00	28.00	39.00	96.43	6.53	375.00	1.15
36.50	0.00	160.00	0.00	128.57	0.00	107.14	10.00
39.50	7.86	0.00	6.50	0.00	0.00	250.00	1.17
15.80	0.00	80.00	139.29	0.00	0.00	250.00	0.00
11.20	0.00	26.00	0.00	0.00	312.50	3.50	5.19
40.50	110.00	80.00	55.71	32.14	0.00	178.57	2.33
11.50	93.33	55.71	0.00	0.00	214.29	10.00	10.38
15.50	15.71	120.00	111.43	32.14	0.00	178.57	10.00
30.00	33.57	69.00	12.00	4.90	170.00	18.67	2.42
27.50	0.00	120.00	111.43	15.00	0.00	250.00	4.67
46.20	0.00	0.00	0.00	96.43	0.00	125.00	10.00
39.70	15.71	0.00	26.00	0.00	0.00	125.00	2.33
25.50	55.00	40.00	13.00	1.23	0.27	250.00	9.33
31.00	0.00	18.67	26.00	96.43	0.00	250.00	2.33
27.00	110.00	18.67	111.43	0.00	0.00	107.14	2.33
36.25	110.00	80.00	1.07	0.62	0.27	178.57	2.33
15.30	0.00	0.00	222.86	0.00	3.27	125.00	2.33
42.50	7.86	14.00	19.50	16.07	0.00	250.00	3.50
60.00	7.33	18.67	195.00	0.00	0.00	107.14	4.67
37.50	0.00	0.00	222.86	0.00	70.00	142.86	2.33
41.20	0.00	0.00	55.71	0.00	0.27	125.00	2.33
19.00	9.17	40.00	83.57	0.00	0.00	375.00	0.00
31.50	31.43	40.00	111.43	32.14	0.81	35.71	0.58
41.20	0.00	120.00	111.43	0.00	0.00	142.86	1.15
33.00	18.33	80.00	0.53	0.62	0.54	178.57	2.33
41.00	0.90	2.30	13.00	7.50	3.20	250.00	15.00
8.90	3.67	14.00	13.00	0.00	0.81	250.00	20.00
36.70	3.01	40.00	16.71	9.64	0.00	325.00	10.00
39.00	7.86	0.00	6.50	0.00	0.00	125.00	1.17
9.00	1.81	140.00	26.00	96.43	6.53	250.00	10.00
40.50	4.67	167.14	3.75	0.00	107.14	5.00	20.76
12.10	0.77	83.57	0.92	0.40	125.00	3.50	7.27
6.00	110.00	80.00	4.27	0.00	0.00	178.57	7.00
19.20	7.33	160.00	13.00	15.00	1.61	107.14	20.00
48.20	31.43	80.00	27.86	0.00	0.00	71.43	10.00
36.20	105.00	2.58	2.84	0.00	4.90	170.00	20.00
43.50	110.00	0.00	0.00	0.00	4.88	504.50	2.33
19.00	0.00	4.67	26.00	3.75	7.00	250.00	20.00
52.90	36.67	0.00	83.57	0.00	3.27	125.00	2.33
41.00	78.57	80.00	13.00	7.50	3.27	250.00	2.33
10.20	31.43	40.00	55.71	32.14	0.00	35.71	0.00
22.50	1.21	160.00	167.14	0.00	1.07	142.86	2.33
29.20	110.00	40.00	0.00	32.14	3.27	142.86	4.67
15.20	4.67	13.00	3.75	0.00	312.50	10.00	62.29
8.00	220.00	0.00	0.00	0.00	0.00	71.43	2.33
33.50	6.18	0.00	180.00	6.53	85.00	9.33	4.84
43.10	110.00	120.00	26.00	0.00	3.27	142.86	10.00
40.20	7.86	0.00	6.50	0.00	0.00	250.00	1.17

19.00	47.14	120.00	167.14	0.00	0.00	107.14	4.67
36.20	62.86	80.00	83.57	32.14	21.00	160.71	3.50
28.00	110.00	40.00	55.71	0.00	6.53	250.00	4.67
27.40	1.53	55.71	0.62	0.27	125.00	2.33	5.19
27.50	0.00	0.00	96.43	0.00	250.00	2.33	8.48
37.00	0.00	40.00	167.14	96.43	0.00	107.14	4.67
33.00	0.00	26.00	0.00	0.00	125.00	3.50	5.19
30.90	0.00	0.00	0.00	450.00	28.00	250.00	10.00
9.40	3.50	6.27	2.30	24.00	7.00	255.00	80.00
9.20	1.53	13.00	64.29	0.00	250.00	2.33	20.76
28.60	0.77	55.71	0.00	0.00	142.86	2.33	10.38
35.50	0.60	1.53	55.71	0.62	0.27	125.00	2.33
22.30	165.00	140.00	26.00	0.00	0.00	250.00	4.67
32.00	7.86	20.00	167.14	0.62	0.00	107.14	0.77
47.60	110.00	28.00	39.00	3.70	0.00	250.00	10.00
39.50	33.57	69.00	12.00	4.90	170.00	18.67	2.42
33.50	0.00	0.00	32.14	0.00	125.00	10.19	12.11
30.20	47.14	80.00	6.41	3.70	1.61	250.00	2.33
18.20	105.00	2.58	2.84	0.00	4.90	170.00	20.00
25.00	15.71	4.67	55.71	1.23	28.00	125.00	2.33
18.10	53.71	19.71	0.00	9.80	109.29	80.00	2.42
47.10	20.00	39.00	0.62	0.27	196.43	4.67	20.76
33.20	0.00	0.00	55.71	96.43	14.00	250.00	2.33
50.00	110.00	20.00	55.71	32.14	4.90	107.14	9.33
28.10	280.00	27.86	3.75	0.00	196.43	5.00	20.76
37.50	110.00	140.00	111.43	0.00	0.00	500.00	10.00
35.20	0.00	0.00	167.14	96.43	0.00	71.43	4.67
17.60	110.00	28.00	167.14	7.50	3.27	250.00	7.00
22.50	20.00	27.86	0.00	14.00	125.00	2.33	5.19
12.50	15.71	0.00	222.86	0.00	0.00	142.86	0.19
46.20	0.00	250.71	0.00	0.27	107.14	2.33	10.38
30.00	3.01	20.00	27.86	16.07	14.00	250.00	10.00
19.50	15.71	4.67	111.43	0.00	0.67	250.00	4.67
43.00	0.00	70.00	167.14	0.00	1.34	26.67	4.67
35.30	40.00	55.71	0.00	0.54	125.00	2.33	5.19
8.00	7.33	120.00	167.14	96.43	6.53	250.00	4.67
38.50	0.00	40.00	55.71	32.14	1.61	107.14	10.00
37.60	47.14	40.00	111.43	32.14	3.27	250.00	4.67
29.40	0.00	9.33	13.00	0.00	0.00	142.86	3.50
18.50	3.67	4.67	13.00	0.00	0.00	250.00	10.00
20.50	80.00	26.00	32.14	3.20	250.00	50.00	62.29
17.80	0.00	13.00	0.00	0.00	125.00	2.33	5.19
11.00	7.33	80.00	55.71	15.00	3.27	160.71	10.00
8.40	7.00	12.53	27.60	18.00	1.63	340.00	40.00
11.00	0.00	0.77	26.00	0.62	0.27	187.50	1.17
21.70	31.43	80.00	111.43	64.29	1.07	107.14	4.67
47.20	0.00	0.00	0.00	96.43	0.00	500.00	1.17
37.50	0.00	0.00	0.00	112.50	6.53	250.00	2.33
28.20	39.29	14.00	111.43	0.00	0.54	250.00	3.50
38.40	0.00	0.00	167.14	0.00	0.00	500.00	10.00
19.60	110.00	40.00	13.00	0.00	1.61	250.00	2.33
27.30	15.71	40.00	55.71	0.00	0.54	125.00	2.33
25.20	20.00	13.00	0.62	0.27	375.00	9.33	31.14

32.20	40.00	0.00	0.00	0.00	250.00	2.33	2.42
29.00	47.00	9.86	12.00	3.27	170.00	9.33	2.42
24.80	3.67	20.00	39.00	0.62	0.27	160.71	2.33
38.00	0.00	40.00	55.71	160.71	0.00	71.43	7.00
41.30	15.71	80.00	222.86	0.00	3.27	142.86	10.00
44.00	80.00	139.29	0.00	0.00	250.00	20.00	23.19
30.00	0.00	160.00	13.00	0.00	0.00	250.00	0.19
32.30	0.00	80.00	0.00	64.29	0.00	71.43	4.67
34.50	0.00	0.00	55.71	3.70	7.00	250.00	2.33
25.50	0.00	0.00	167.14	0.00	0.00	71.43	4.67
20.30	110.00	40.00	55.71	0.00	0.00	375.00	20.00
37.00	110.00	80.00	0.00	0.00	1.61	250.00	2.33
24.10	78.57	80.00	111.43	0.00	0.00	250.00	0.77
40.50	80.00	0.00	0.00	0.00	250.00	0.00	0.80
35.70	0.00	40.00	167.14	96.43	0.00	142.86	1.15
9.00	47.14	7.67	167.14	6.16	42.00	35.71	1.15
31.00	0.00	80.00	0.00	0.00	0.00	250.00	2.33
11.20	7.33	40.00	55.71	2.47	6.53	107.14	2.33
35.20	0.00	14.00	167.14	0.00	4.90	107.14	4.67
18.50	80.00	111.43	0.00	0.00	375.00	4.67	2.42
24.10	0.00	0.77	52.00	0.62	0.27	375.00	7.00
43.50	1.81	120.00	111.43	0.00	0.00	107.14	4.67
48.50	0.00	120.00	167.14	0.00	6.53	250.00	10.00
21.50	5.50	14.00	19.50	0.00	0.81	375.00	10.00
61.00	0.00	0.00	195.00	32.14	6.53	250.00	4.67
40.00	0.00	120.00	167.14	0.00	0.00	178.57	10.00
31.00	0.00	0.00	55.71	0.00	0.54	125.00	2.33
6.00	1.05	120.00	55.71	7.50	1.21	250.00	20.00
39.50	3.67	14.00	55.71	0.00	3.27	142.86	10.00
32.50	78.57	0.00	13.00	225.00	6.53	178.57	2.33
19.50	0.00	80.00	97.50	16.07	3.27	71.43	4.67
14.20	47.00	9.86	12.00	3.27	170.00	9.33	2.42
11.50	10.00	26.86	13.14	5.14	3.27	85.00	18.67
19.00	2.80	33.57	69.00	12.00	4.90	170.00	18.67
16.50	31.43	18.67	55.71	1.23	14.00	107.14	10.00
52.00	0.00	1.53	13.00	32.14	0.00	375.00	4.67
19.50	0.00	1.53	167.14	1.23	0.00	142.86	3.50
24.50	0.00	3.84	26.00	0.00	0.00	375.00	2.33
52.20	3.67	9.33	167.14	64.29	3.27	250.00	5.00
20.50	0.58	33.57	16.43	6.86	3.27	85.00	40.00
27.50	110.00	120.00	167.14	7.50	0.54	178.57	0.38
14.50	1.51	18.67	32.50	7.50	0.27	250.00	10.00
42.60	47.14	40.00	55.71	7.50	3.27	142.86	2.33
33.00	3.67	9.33	55.71	1.23	0.00	71.43	10.00
26.70	1.51	3.84	111.43	3.70	1.34	250.00	2.33
28.40	3.67	120.00	55.71	7.50	2.68	250.00	1.15
26.50	40.00	111.43	0.00	0.00	142.86	10.00	2.42
10.00	3.67	120.00	55.71	7.50	0.00	250.00	7.00
18.30	0.00	107.43	6.90	4.00	0.94	127.50	9.33
19.30	7.33	120.00	167.14	7.50	1.07	250.00	4.67
18.20	220.00	18.67	26.00	0.00	0.00	250.00	2.33
37.50	0.45	1.15	83.57	0.62	0.27	175.00	2.33
45.20	0.60	1.53	27.86	0.00	0.00	125.00	2.33

23.50	0.00	0.00	167.14	96.43	6.53	178.57	4.67
14.50	0.00	120.00	111.43	0.00	0.00	107.14	30.00
24.50	110.00	160.00	111.43	0.00	0.00	250.00	10.00
35.20	20.00	27.86	0.00	14.00	125.00	2.33	5.19
12.20	2.80	33.57	69.00	12.00	4.90	170.00	18.67
16.70	31.43	0.00	13.00	0.00	0.00	250.00	2.33
72.00	110.00	80.00	111.43	0.00	14.00	250.00	10.00
19.70	15.71	120.00	55.71	0.00	1.61	285.71	10.00
19.00	3.67	0.00	55.71	32.14	0.00	250.00	5.00
38.00	0.00	0.00	167.14	0.00	0.00	107.14	4.67
26.10	140.00	27.86	3.75	0.00	250.00	12.50	20.76
9.50	20.00	55.71	0.00	0.00	107.14	10.00	20.76
31.20	0.77	27.86	0.00	0.00	178.57	2.33	20.76
8.20	3.67	1.53	55.71	0.00	0.00	142.86	2.33
10.80	55.00	40.00	6.50	0.62	0.27	375.00	11.67
26.20	0.00	120.00	222.86	15.00	3.27	125.00	4.67
43.40	0.00	80.00	111.43	0.00	0.00	250.00	2.33
24.50	40.00	0.00	16.07	0.00	89.29	0.38	4.84
18.50	15.71	40.00	55.71	32.14	14.00	107.14	4.67
19.60	172.86	89.33	225.53	7.50	4.90	342.86	27.00
12.50	55.00	40.00	55.71	15.00	6.53	250.00	0.00
25.50	0.00	222.86	0.00	8.17	250.00	2.33	2.42
19.80	110.00	40.00	55.71	0.00	0.00	107.14	10.00
23.40	0.77	39.00	0.62	0.27	187.50	7.00	20.76
46.00	0.00	0.00	111.43	0.00	0.00	250.00	20.00
8.00	0.00	9.33	55.71	0.00	0.67	250.00	0.00
25.00	120.00	0.53	0.62	0.54	196.43	2.33	10.38
18.20	47.14	80.00	167.14	64.29	6.53	250.00	4.67
29.70	0.00	0.00	96.43	0.00	250.00	30.00	4.84
27.60	31.43	40.00	111.43	64.29	1.61	35.71	4.67
32.10	0.00	80.00	55.71	0.00	1.07	107.14	2.33
28.00	15.71	13.20	1.07	0.62	0.54	178.57	2.33
40.40	15.71	0.00	55.71	32.14	0.81	250.00	0.00
34.50	40.00	1.07	0.62	0.54	178.57	2.33	20.76
31.20	0.60	28.00	39.00	22.50	0.00	250.00	10.00
28.20	20.00	27.86	16.07	0.00	125.00	0.19	2.42
25.50	1.81	4.60	111.43	3.70	1.61	107.14	4.67
37.50	0.00	0.00	195.00	0.00	0.00	107.14	4.67
35.20	0.30	18.67	195.00	0.00	3.27	107.14	7.00
22.50	15.71	0.77	222.86	0.00	0.27	142.86	0.38
16.70	40.00	55.71	32.14	0.00	250.00	10.00	62.29
14.20	0.00	13.00	0.00	0.00	125.00	2.33	5.19
27.20	23.57	18.67	150.43	7.50	28.00	178.57	4.67
36.00	9.33	55.71	0.00	3.27	250.00	4.67	41.53
38.50	15.71	40.00	13.00	64.29	3.20	375.00	10.00
6.00	15.71	0.00	13.00	0.00	0.00	125.00	1.17
29.20	7.86	60.00	111.43	48.21	0.00	35.71	4.67
21.00	0.58	33.57	16.43	6.86	3.27	85.00	40.00
32.10	47.14	0.00	13.00	64.29	6.53	250.00	4.67
37.50	1.81	18.67	390.00	1.23	0.81	250.00	2.33
30.00	110.00	10.00	13.00	32.14	0.00	312.50	9.33
45.10	47.14	4.60	6.41	3.70	1.61	107.14	1.15
31.20	140.00	69.64	9.38	0.00	169.64	12.50	20.76

36.20	47.14	80.00	0.00	0.00	42.00	250.00	0.00
39.00	0.00	80.00	167.14	15.00	6.53	107.14	0.96
24.50	0.00	28.00	111.43	15.00	6.53	125.00	7.00
43.00	0.00	111.43	0.00	98.00	250.00	20.00	0.00
17.20	0.00	80.00	167.14	3.70	1.61	250.00	4.67
15.50	9.33	13.00	0.00	3.27	214.29	11.67	20.76
41.20	0.00	28.00	167.14	7.50	0.00	178.57	20.00
9.20	0.00	0.00	167.14	0.00	0.00	214.29	10.00
45.00	7.33	120.00	111.43	32.14	0.00	250.00	4.67
63.10	140.00	55.71	7.50	0.00	285.71	10.00	51.91
35.00	4.67	55.71	1.23	28.00	160.71	2.33	20.76
12.30	7.33	40.00	1.07	0.62	0.27	214.29	2.33
21.30	62.86	120.00	26.00	3.70	1.61	142.86	4.67
17.60	0.00	120.00	139.29	64.29	0.27	250.00	1.15
41.20	0.00	0.77	111.43	1.23	0.27	125.00	2.33
19.50	31.43	40.00	167.14	96.43	0.00	250.00	2.33
32.10	110.00	280.00	0.00	0.00	0.00	89.29	0.00
36.90	2.80	33.57	69.00	12.00	4.90	170.00	18.67
19.50	47.14	40.00	55.71	7.50	3.27	142.86	2.33
24.00	39.29	80.00	55.71	7.50	3.27	250.00	4.67
43.10	15.71	0.00	13.00	0.00	0.00	125.00	1.17
39.00	15.71	120.00	111.43	32.14	9.80	142.86	2.33
21.00	7.00	23.50	69.00	0.00	0.27	170.00	18.67
29.50	0.00	40.00	111.43	64.29	14.00	250.00	10.00
31.20	31.43	0.00	13.00	0.00	0.00	250.00	2.33
36.50	0.00	0.00	0.00	450.00	3.27	4.50	0.00
43.00	3.67	20.00	0.00	64.29	0.00	107.14	20.96
29.60	0.00	83.57	0.00	16.33	178.57	5.83	20.76
45.00	110.00	280.00	0.00	225.00	0.00	250.00	15.00
39.10	3.67	14.00	26.00	32.14	0.00	178.57	4.67
9.00	0.00	4.60	55.71	225.00	6.53	250.00	10.00
25.50	1.83	20.00	27.86	16.07	0.00	125.00	0.19
41.20	47.14	80.00	111.43	0.00	0.00	250.00	10.00
60.00	0.30	0.77	83.57	0.92	0.40	125.00	3.50
14.50	0.00	9.33	167.14	32.14	0.00	107.14	2.33
29.50	1.83	140.00	83.57	11.25	0.00	214.29	20.00
42.20	0.30	0.77	278.57	0.00	0.00	178.57	0.38
46.00	0.00	120.00	167.14	32.14	3.27	250.00	10.00
8.50	55.00	40.00	55.71	0.00	0.00	107.14	2.33
17.50	1.81	80.00	167.14	3.70	0.81	370.00	0.96
34.20	0.00	0.00	390.00	0.00	0.00	71.43	10.00
43.20	0.00	80.00	111.43	0.00	0.00	250.00	4.67
46.20	7.86	20.00	167.14	7.50	0.00	178.57	0.10
22.70	345.71	178.67	283.91	15.00	9.80	378.57	44.00
23.50	1.83	4.67	39.00	0.00	3.20	250.00	20.00
12.40	0.00	1.53	32.50	96.43	3.27	107.14	20.00
38.50	1.83	0.00	0.00	32.14	0.00	125.00	10.19
30.10	0.77	55.71	0.00	0.00	142.86	2.33	10.38
28.50	0.00	0.00	97.50	0.00	0.00	375.00	2.33
14.50	0.00	80.00	111.43	0.00	0.81	107.14	2.33
9.50	3.67	40.00	111.43	48.21	0.00	125.00	0.38
37.50	1.51	280.00	0.00	0.00	0.27	250.00	0.38
45.00	47.14	18.67	13.00	0.00	14.00	250.00	10.00

33.50	3.67	37.33	52.00	112.50	3.27	250.00	10.00
33.50	78.57	37.33	111.43	0.00	0.00	178.57	0.00
28.30	0.77	83.57	0.92	0.40	175.00	3.50	7.27
26.10	55.00	9.33	13.00	0.00	3.27	107.14	11.67
31.20	0.00	0.00	111.43	96.43	0.00	500.00	0.19
45.20	0.00	0.00	0.00	337.50	6.53	500.00	15.00
27.20	0.00	107.43	6.90	4.00	0.94	127.50	9.33
33.50	0.00	80.00	111.43	32.14	6.53	250.00	4.67
17.20	0.00	0.00	139.29	96.43	0.54	71.43	0.19
13.60	220.00	20.00	27.86	0.00	70.00	214.29	2.33
11.20	1.51	4.67	13.00	18.75	0.00	375.00	10.00
34.20	77.00	40.00	13.00	1.23	0.27	312.50	9.33
14.30	3.67	0.77	55.71	0.00	0.00	71.43	1.17
29.40	15.71	0.00	13.00	0.00	0.00	125.00	2.33
35.20	0.00	120.00	167.14	7.50	3.27	107.14	2.33
9.00	110.00	40.00	167.14	0.00	0.00	142.86	10.00
37.80	125.71	40.00	260.00	0.00	0.00	500.00	2.33
14.00	2.80	33.57	69.00	12.00	4.90	170.00	18.67
17.90	7.86	0.00	6.50	0.00	0.00	250.00	1.17
18.00	0.00	40.00	111.43	1.23	0.00	107.14	20.00
24.60	0.00	26.00	0.00	0.00	312.50	3.50	5.19
31.50	0.00	280.00	55.71	0.00	0.00	160.71	20.00
17.50	0.00	80.00	111.43	15.00	6.53	250.00	10.00
18.20	15.71	0.00	167.14	0.00	14.00	250.00	1.73
22.30	55.00	40.00	55.71	0.00	0.00	107.14	2.33
22.20	0.60	120.00	167.14	0.00	0.00	250.00	0.38
31.00	110.00	40.00	13.00	0.62	0.27	250.00	4.67
22.20	47.14	80.00	111.43	64.29	0.00	250.00	4.67
41.20	0.00	0.77	55.71	59.95	0.00	178.57	0.58
25.00	11.00	28.00	39.00	22.50	0.00	250.00	0.00
11.50	0.00	80.00	111.43	96.43	0.00	250.00	4.67

sibzamini	sibzaminis	reshteyeso	reshteyeas	biscuit	keraker	keikebaste	zorat
10.53	0.05	4.67	7.83	3.43	2.14	3.33	17.86
5.19	0.04	0.00	33.57	0.27	0.00	1.67	0.17
5.19	0.86	0.00	0.00	1.14	0.00	0.00	0.00
5.19	1.00	0.00	0.00	0.53	0.00	1.67	0.17
22.26	0.50	1.53	2.58	0.00	0.00	0.00	1.03
10.38	0.00	2.30	3.86	0.00	0.00	0.82	1.03
51.91	2.00	9.33	0.00	2.29	0.00	3.33	0.34
2.42	0.00	0.00	0.00	1.14	0.00	0.00	0.00
0.08	0.00	0.00	0.27	0.00	1.67	0.17	3.53
20.76	0.00	0.00	0.00	0.00	0.00	1.67	0.00
2.00	4.67	1.29	5.71	0.00	1.67	0.00	0.00
11.67	0.43	0.00	6.44	8.00	0.17	0.00	2.08
3.29	0.38	0.64	2.29	0.00	7.14	0.68	1.45
5.44	0.20	0.00	3.86	0.00	0.00	0.00	1.03
2.42	0.10	0.00	33.57	1.14	0.00	0.00	0.86
10.38	0.20	4.67	0.00	1.14	0.00	1.67	0.17
31.14	4.71	4.67	67.14	0.27	0.01	1.67	0.17
2.42	0.00	0.00	2.58	0.00	0.00	0.00	0.00
3.41	0.33	3.84	6.44	1.14	0.00	0.00	0.00
10.38	2.14	4.67	67.14	0.27	0.01	1.67	0.00
2.42	0.10	4.67	1.29	0.04	0.02	1.67	2.08
62.29	3.00	1.92	0.00	5.71	0.14	1.67	0.00
4.84	0.00	1.15	15.67	0.04	0.03	0.00	4.17
2.53	0.25	0.00	1.29	0.11	0.00	0.41	0.00
5.19	0.04	0.00	33.57	0.27	0.00	1.67	0.17
10.38	4.29	7.00	0.00	13.71	0.00	5.00	0.00
27.19	2.14	7.00	0.64	5.71	0.00	0.00	2.08
36.29	1.71	0.00	3.22	0.00	0.00	0.00	1.03
10.38	12.86	4.67	16.79	1.33	0.01	1.67	0.00
25.95	1.00	9.33	15.67	3.43	0.00	3.33	0.00
67.48	0.41	9.33	15.67	6.29	0.83	5.00	0.00
31.14	2.00	2.33	3.92	2.86	0.71	2.50	0.26
51.91	2.00	9.33	0.00	2.29	0.00	3.33	0.34
35.64	1.50	9.33	3.86	1.14	0.17	0.55	0.00
2.14	10.00	3.92	2.29	0.03	1.67	2.08	3.53
0.08	0.00	0.64	0.53	0.00	1.67	0.34	5.30
23.76	1.00	0.00	3.86	0.11	0.00	0.00	0.00
20.96	0.07	0.00	3.86	0.53	0.00	0.82	4.17
20.76	0.43	20.00	0.00	2.29	0.00	0.00	0.00
14.53	6.67	1.53	7.83	0.48	0.00	6.67	0.51
2.42	0.10	11.67	0.00	0.00	0.00	1.67	0.00
36.34	5.00	4.67	7.83	2.29	0.00	8.33	0.00
51.91	2.50	0.00	78.33	5.71	0.33	7.14	0.00
46.03	1.50	14.00	7.83	3.43	0.00	0.00	0.00
59.33	12.86	20.00	15.67	0.00	0.00	0.00	8.93
8.42	2.00	0.00	1.29	0.00	0.00	0.00	0.68
31.14	0.00	0.00	7.83	3.43	0.00	21.43	2.08
2.20	0.00	0.00	11.43	0.00	8.33	0.00	7.07
0.00	0.00	0.00	7.83	0.00	0.08	0.14	0.17
13.33	2.30	3.86	0.24	0.00	7.14	0.51	3.53
31.14	0.00	4.67	67.14	0.00	0.17	1.67	4.17
5.19	0.20	0.00	0.00	0.53	0.00	1.67	0.17

31.74	0.20	2.30	3.86	3.43	0.00	0.00	1.03
4.84	0.90	4.67	1.29	0.02	0.02	8.33	4.17
85.53	4.29	4.67	2.58	0.53	0.00	0.00	0.00
0.04	0.00	16.79	0.27	0.00	1.67	0.17	3.53
0.25	11.67	0.00	4.00	0.00	3.33	0.00	3.53
4.84	0.00	9.33	15.67	0.00	0.00	0.00	0.00
1.00	0.00	0.00	0.53	0.00	1.67	0.17	3.53
10.38	0.86	0.00	33.57	1.14	0.00	0.27	0.17
10.38	28.57	4.67	7.83	1.87	0.00	7.14	2.08
0.86	0.00	15.67	2.29	0.00	35.71	2.08	0.58
0.16	0.00	0.64	0.27	0.01	1.67	0.34	5.30
5.19	0.04	0.00	16.79	0.27	0.00	1.67	0.17
0.00	0.00	0.00	7.83	3.43	0.00	3.33	1.03
20.76	1.20	0.77	1.29	2.29	0.03	1.67	6.25
8.70	1.29	9.33	15.67	0.00	0.00	0.00	0.00
3.29	0.38	0.64	2.29	0.00	7.14	0.68	1.45
0.00	0.00	0.00	1.14	0.00	0.68	0.17	10.60
31.14	0.00	9.33	3.86	0.11	0.00	0.82	1.03
14.53	6.67	1.53	7.83	0.48	0.00	6.67	0.51
31.14	0.86	0.00	7.83	1.14	0.00	14.29	2.08
6.67	0.58	0.64	32.00	0.00	1.67	0.68	0.29
0.86	4.67	33.57	0.27	0.01	1.67	0.00	0.58
2.42	0.00	4.67	0.00	0.00	0.00	0.00	0.17
25.26	1.50	4.67	7.83	1.07	0.00	0.00	2.08
36.00	2.33	1.29	11.43	0.03	3.33	1.04	0.00
10.38	1.29	20.00	1.29	0.04	0.04	0.00	0.00
15.57	0.00	4.67	0.00	3.43	0.00	1.67	2.08
10.99	3.00	3.84	5.15	9.14	0.17	3.33	1.03
0.50	2.33	0.00	2.29	0.00	3.33	0.00	0.00
10.38	0.50	1.15	1.93	3.43	0.03	1.67	6.25
0.86	0.00	11.75	1.14	0.01	0.83	4.17	45.43
31.14	2.50	2.33	3.92	2.86	0.71	2.50	0.26
124.58	12.86	1.92	0.00	5.71	0.00	5.00	2.08
1.19	0.00	1.92	33.57	3.43	0.00	7.14	26.79
0.08	0.00	33.57	0.27	0.00	1.67	0.86	7.07
10.98	0.20	2.30	3.86	3.43	0.00	0.82	1.03
1.29	0.43	2.30	0.00	0.13	0.00	0.82	4.17
72.97	0.10	0.00	1.29	2.29	0.00	1.67	0.00
20.76	2.14	4.67	0.00	4.57	0.17	1.67	0.00
62.29	1.20	4.67	7.83	5.71	0.00	5.00	0.00
12.86	9.33	15.67	2.29	0.00	3.33	0.00	3.53
0.08	0.00	33.57	0.27	0.00	1.67	0.86	7.07
26.76	2.00	11.67	7.83	2.67	0.00	1.67	2.08
20.76	1.10	0.00	1.29	0.48	0.00	3.33	0.34
20.76	2.14	2.33	7.83	0.13	0.01	3.33	8.93
4.99	0.05	9.33	33.57	0.00	0.00	0.00	2.08
1.21	0.10	4.67	0.64	0.02	0.03	6.67	8.33
23.24	4.29	0.00	7.83	3.43	0.00	7.14	0.00
51.91	2.14	0.38	7.83	11.43	0.71	3.33	0.00
10.38	0.43	0.00	0.00	1.14	0.00	0.00	0.00
1.29	0.43	0.00	7.83	8.00	0.00	7.14	0.00
5.19	0.08	0.00	33.57	0.27	0.00	1.67	0.86
6.43	4.67	67.14	0.27	0.01	1.67	0.17	0.29

0.01	0.38	0.00	4.57	0.00	5.00	0.00	0.29
6.67	20.00	7.83	0.53	0.00	1.67	4.17	7.07
10.38	4.29	4.67	16.79	1.33	0.01	1.67	0.00
16.38	2.00	0.00	0.00	0.27	0.17	1.67	0.00
34.14	1.00	1.53	33.57	0.27	0.17	1.67	4.17
0.86	40.00	0.00	1.14	0.00	1.67	0.00	0.00
2.42	0.00	0.00	0.00	0.00	0.00	0.00	8.33
8.01	0.25	40.00	0.00	0.53	0.00	0.00	0.34
23.24	4.29	2.30	7.83	0.00	0.00	0.00	2.08
115.34	0.00	70.00	7.83	16.00	0.00	4.17	0.26
51.91	21.43	40.00	0.00	0.00	0.00	0.00	0.00
4.84	0.20	4.67	0.00	0.00	0.00	0.00	0.00
20.76	0.00	0.00	33.57	0.00	0.00	0.00	2.08
0.30	2.30	11.75	0.40	0.00	0.00	0.00	14.13
31.14	0.00	2.30	15.67	8.00	0.00	0.00	0.86
29.67	6.43	60.00	3.86	0.13	0.08	0.82	4.17
0.00	0.10	0.00	0.00	0.53	0.00	0.00	0.00
6.92	1.50	1.53	2.58	0.09	0.05	0.00	27.47
14.88	1.50	9.33	2.90	1.14	0.00	1.67	4.17
0.10	0.00	0.00	0.80	0.00	0.00	0.00	0.44
10.38	0.86	2.33	16.79	0.27	0.01	1.67	0.00
5.44	0.20	9.33	3.86	0.53	0.08	0.82	4.17
10.84	2.00	1.53	2.58	0.00	0.00	0.00	0.34
57.10	0.41	9.33	15.67	6.29	0.83	5.00	0.00
10.38	0.00	9.33	2.58	0.00	0.00	0.41	2.08
2.30	0.50	9.33	0.00	0.04	0.00	0.82	0.00
5.19	0.08	0.00	33.57	0.27	0.00	1.67	0.86
23.24	4.29	50.00	7.83	0.40	0.00	2.50	0.86
11.67	0.43	4.67	2.58	6.86	0.00	3.33	0.68
10.38	4.29	40.00	7.83	1.14	0.17	7.14	2.08
2.42	0.00	4.67	0.00	0.00	0.00	0.00	2.08
6.67	20.00	7.83	0.53	0.00	1.67	4.17	7.07
20.76	13.33	9.33	7.83	16.00	0.00	3.33	35.71
2.42	3.29	0.38	0.64	2.29	0.00	7.14	0.68
29.67	6.43	9.33	0.64	2.29	0.00	3.33	2.08
20.76	4.29	0.00	15.67	2.29	0.00	14.29	2.08
20.76	2.14	0.00	3.92	0.57	0.01	0.83	6.25
20.76	0.86	0.00	15.67	2.29	0.00	35.71	2.08
31.05	3.43	4.67	7.83	2.86	0.00	0.55	2.08
4.84	85.71	4.67	5.15	8.00	0.00	7.14	2.08
2.40	0.80	40.00	67.14	1.14	0.00	0.00	0.86
41.53	0.16	0.00	23.50	6.29	0.83	5.00	0.00
10.38	0.00	0.00	7.83	3.43	0.00	1.67	0.00
24.13	6.43	20.00	33.57	0.00	0.00	0.82	2.08
22.00	0.41	1.92	6.44	0.00	0.00	0.00	0.86
45.60	15.00	0.00	3.22	0.13	0.07	0.82	2.08
0.43	20.00	0.00	1.14	0.00	1.67	0.00	0.00
10.68	0.10	9.33	0.00	8.00	0.00	7.14	0.00
1.99	1.10	1.15	3.22	3.43	0.00	3.33	0.51
21.36	0.20	4.67	0.00	0.53	0.08	0.00	4.17
2.42	0.20	9.33	1.93	0.04	0.01	3.33	2.08
5.19	0.04	0.00	0.64	0.27	0.00	1.67	0.17
5.19	0.08	0.00	0.64	0.53	0.00	1.67	0.34

1.34	0.05	0.00	3.86	0.00	0.00	0.00	0.00
20.76	0.00	9.33	0.00	1.14	0.00	0.00	0.00
33.72	0.86	9.33	33.57	0.00	0.00	0.00	0.86
0.50	2.33	0.00	2.29	0.00	3.33	0.00	0.00
2.42	3.29	0.38	0.64	2.29	0.00	7.14	0.68
31.14	1.00	7.00	0.00	2.29	0.00	3.33	0.34
33.62	4.29	0.00	7.83	0.00	0.00	3.33	2.08
164.63	6.43	4.67	3.86	0.13	0.00	0.82	0.34
20.76	5.14	4.67	0.00	13.71	0.00	5.00	0.00
24.62	1.29	0.00	3.86	8.00	1.43	0.68	17.86
5.14	2.33	1.93	3.43	0.00	5.00	0.00	0.00
10.71	20.00	7.83	1.14	0.01	0.00	2.08	0.58
0.00	0.00	1.29	0.00	0.01	1.67	0.34	5.30
10.38	1.00	0.00	1.29	0.00	0.01	1.67	0.34
31.14	6.43	4.67	67.14	0.27	0.01	1.67	0.17
3.16	0.25	1.15	3.86	0.13	0.00	0.00	1.03
23.24	4.29	0.00	0.00	0.53	0.00	0.00	1.03
0.86	9.33	0.00	1.14	0.00	0.00	0.00	10.60
26.76	2.00	9.33	3.86	5.71	0.14	3.33	1.03
71.29	3.00	34.00	52.46	24.53	0.00	10.48	4.17
47.53	2.00	20.00	0.00	2.29	0.00	7.14	1.03
0.01	0.96	0.00	4.00	0.00	3.33	0.00	3.53
20.76	0.43	20.00	0.00	0.00	0.00	0.00	0.00
2.14	4.67	3.92	0.27	0.01	1.67	0.00	1.45
4.84	0.20	0.00	134.29	4.57	0.00	0.00	0.34
23.33	0.86	0.00	3.22	0.04	0.00	7.14	0.34
12.86	4.67	16.79	1.33	0.01	1.67	0.00	0.58
48.95	12.86	20.00	7.83	2.29	2.14	1.67	2.08
0.10	4.67	0.64	0.02	0.04	3.33	4.17	0.00
13.84	3.00	0.00	3.86	2.29	0.17	3.33	1.20
12.69	1.00	1.53	2.58	8.00	0.00	0.27	0.68
10.38	12.86	4.67	16.79	1.33	0.01	1.67	0.00
62.29	0.08	9.33	15.67	5.14	0.00	3.33	0.00
2.14	2.33	50.36	1.33	0.01	1.67	0.00	0.58
41.53	2.00	0.00	23.50	11.43	0.00	8.33	0.00
0.43	4.67	0.00	1.14	0.00	0.00	8.93	3.53
10.98	0.20	2.30	3.86	8.00	0.08	0.82	1.03
10.38	0.00	9.33	15.67	0.04	0.00	0.00	1.20
19.38	3.00	9.33	15.67	1.14	0.00	0.00	0.34
20.76	1.00	0.38	0.64	5.71	0.00	1.67	12.50
1.50	0.00	7.83	3.43	0.00	2.50	0.00	0.00
0.08	0.00	33.57	0.27	0.00	1.67	0.17	3.53
10.84	2.00	2.68	7.83	0.53	0.00	1.67	0.34
5.00	0.00	7.83	2.29	0.17	7.14	0.00	3.53
51.91	3.00	4.67	0.00	6.86	0.08	1.67	0.17
5.19	0.50	4.67	0.00	0.27	1.43	1.67	0.17
34.14	1.00	4.67	7.83	0.80	0.00	0.00	2.08
4.84	85.71	4.67	5.15	8.00	0.00	7.14	2.08
10.38	4.29	20.00	7.83	11.43	0.17	35.71	2.08
13.70	2.14	1.53	2.58	2.29	0.00	7.14	0.00
0.00	0.00	0.00	0.00	0.00	0.00	0.00	0.00
14.24	1.29	2.30	3.86	0.13	0.08	0.82	1.03
5.14	2.33	1.29	11.43	0.03	5.00	0.00	0.00

4.84	0.00	0.00	7.83	0.00	0.00	0.82	0.00
24.13	6.43	1.53	1.29	0.00	0.00	3.33	2.08
13.27	2.00	9.33	1.93	0.53	0.00	0.00	4.17
0.00	0.00	33.57	1.14	0.00	0.55	0.00	0.87
10.63	0.08	2.30	15.67	0.00	0.00	0.00	0.34
0.08	40.00	15.67	17.14	0.00	7.14	2.08	3.53
51.91	1.00	9.33	0.00	5.71	0.00	10.00	0.00
23.76	1.00	0.00	7.83	2.29	0.00	0.00	0.00
33.72	0.86	4.67	15.67	1.14	0.00	7.14	0.00
30.00	4.67	1.29	11.43	0.00	1.67	0.00	0.00
2.14	0.00	7.83	2.29	0.00	14.29	2.08	0.58
10.38	12.86	4.67	16.79	1.33	0.01	1.67	0.00
10.98	0.20	2.30	3.86	0.13	0.08	0.68	8.93
21.06	0.10	0.00	15.67	4.57	0.00	1.67	0.51
5.19	0.04	0.00	16.79	0.27	0.00	1.67	0.17
3.57	0.86	60.00	15.67	0.00	0.00	0.00	0.17
5.19	0.43	20.00	0.00	2.29	0.00	0.00	0.17
2.42	3.29	0.38	0.64	2.29	0.00	7.14	0.68
10.38	0.00	0.00	7.83	3.43	0.00	1.67	0.00
15.28	4.29	30.00	15.67	24.00	0.00	10.71	0.17
5.19	0.50	4.67	0.00	0.27	1.43	1.67	0.17
27.19	2.14	0.77	3.22	0.27	0.17	0.68	4.17
2.42	9.86	4.67	7.83	0.53	0.00	3.33	1.20
14.88	1.50	20.00	7.83	0.27	0.00	0.00	0.00
31.14	1.00	7.00	0.00	2.29	0.00	3.33	0.34
0.00	0.00	0.00	39.17	0.27	0.17	1.67	4.17
4.84	1.00	0.00	0.00	1.14	0.00	0.00	0.00
1.00	0.00	7.83	2.29	0.17	7.14	0.00	3.53
0.00	0.00	0.00	33.57	1.14	0.00	0.14	0.00
51.91	6.43	4.67	0.00	4.57	0.17	1.67	0.00
6.13	0.43	9.33	15.67	1.14	0.08	0.82	4.17
2.42	0.43	4.67	0.00	1.14	0.00	0.00	8.93
10.38	0.00	9.33	15.67	0.00	0.00	0.00	0.00
7.27	0.08	0.00	0.64	0.53	0.00	1.67	0.34
4.84	0.00	4.67	2.58	0.02	0.00	0.00	0.68
62.29	30.00	4.67	1.29	11.43	0.00	1.67	0.00
31.14	2.14	0.00	3.92	0.57	0.00	1.67	12.50
14.88	1.50	9.33	7.83	0.00	0.00	0.00	2.08
10.38	0.43	20.00	0.00	1.14	0.00	0.00	0.00
19.38	3.00	4.67	33.57	0.53	0.00	1.67	0.68
10.98	0.20	9.33	1.29	0.00	0.00	7.14	2.08
10.14	2.57	9.33	0.00	8.00	0.00	0.00	2.08
5.19	0.50	0.38	0.64	1.14	0.00	1.67	6.25
132.20	6.00	63.33	97.08	29.07	0.00	19.29	8.33
41.53	2.20	9.33	15.67	11.43	1.67	5.00	0.17
18.57	1.00	7.00	7.83	0.00	0.00	1.37	0.00
12.11	0.00	0.00	0.00	1.14	0.00	0.68	0.17
0.16	0.00	0.64	0.27	0.01	1.67	0.34	5.30
9.34	1.50	0.00	3.86	0.80	0.00	0.00	1.71
3.92	0.50	0.00	0.00	0.00	0.00	0.27	0.17
4.84	0.86	9.33	0.00	1.14	0.00	0.00	8.93
0.00	0.00	0.00	83.93	2.29	0.00	0.68	0.17
20.76	0.00	4.67	3.86	0.00	0.00	0.00	2.08

46.03	1.50	0.00	7.83	0.00	0.00	0.00	2.08
0.00	0.00	0.00	7.83	1.14	0.00	0.00	8.93
0.04	0.00	0.64	0.27	0.00	1.67	0.34	5.30
51.91	8.57	40.00	15.67	17.14	0.00	7.14	4.17
10.38	0.43	0.00	0.00	0.00	0.00	0.00	0.68
0.00	0.00	0.00	33.57	1.14	0.00	0.34	0.00
1.99	1.10	1.15	3.22	3.43	0.00	3.33	0.51
37.57	2.14	1.92	6.44	1.14	0.00	3.33	0.68
6.34	0.50	0.00	2.58	0.07	0.00	0.68	1.03
5.19	0.86	0.00	0.00	1.14	0.00	0.00	0.00
51.91	1.00	0.00	0.00	11.43	0.00	8.33	0.00
31.14	4.71	4.67	67.14	0.27	0.01	1.67	0.17
31.14	0.00	0.00	15.67	0.00	0.01	1.67	0.34
51.91	1.00	4.67	0.00	2.29	0.00	3.33	0.34
4.84	0.00	4.67	100.71	0.02	0.71	7.14	0.68
43.42	12.86	14.00	47.00	0.00	0.00	0.00	0.00
25.95	0.01	0.38	0.00	0.53	0.00	1.67	0.00
2.42	3.29	0.38	0.64	2.29	0.00	7.14	0.68
5.19	0.20	0.00	0.00	0.53	0.00	1.67	0.17
2.42	0.00	4.67	1.29	2.29	0.03	3.33	2.08
0.08	0.00	0.00	0.27	0.00	1.67	0.17	3.53
62.29	0.16	4.67	1.29	5.71	0.00	1.67	0.00
45.38	1.29	40.00	15.67	8.00	0.00	3.33	0.86
42.81	0.43	9.33	23.50	0.00	0.00	0.00	8.93
10.38	0.43	20.00	0.00	1.14	0.00	0.00	0.00
10.87	0.16	4.67	0.64	0.53	0.00	0.27	0.51
20.76	4.29	4.67	67.14	0.27	0.01	1.67	0.17
4.84	0.00	1.15	1.93	0.53	0.33	3.33	0.51
23.76	1.00	9.33	7.83	0.00	0.00	0.00	0.00
10.98	0.20	1.15	7.83	0.00	0.00	0.00	0.00
2.42	0.00	0.00	7.83	0.00	0.00	0.00	0.00

jo	adas	loobia	nokhod	bagholapol	soya	mash	lape	
45.43	12.30	12.00	13.30	8.40	2.01	12.00	12.30	
3.53	17.57	17.14	19.00	12.00	0.00	0.00	4.10	
0.00	8.79	8.57	9.50	0.00	30.00	0.00	0.00	
3.53	8.79	17.14	19.00	0.00	0.00	0.66	0.00	
7.07	8.20	17.14	0.00	0.23	0.00	0.00	2.02	
1.74	17.57	17.14	8.87	1.38	7.00	1.97	8.20	
3.53	17.57	17.14	19.00	2.80	0.00	0.66	0.00	
0.00	0.00	0.00	8.87	0.00	0.00	0.00	17.57	
35.14	17.14	19.00	0.00	0.00	0.00	8.20	0.79	
3.53	4.10	8.00	4.43	0.00	0.00	0.00	4.10	
8.20	8.00	22.17	0.00	0.00	0.00	26.36	3.14	
7.07	4.10	4.00	0.00	2.80	0.00	0.00	0.00	
4.10	4.00	4.43	0.23	3.50	0.33	0.34	9.43	
7.07	8.20	0.00	2.19	0.00	0.00	0.00	8.20	
0.29	0.34	12.00	15.52	2.80	0.00	0.00	4.10	
3.53	8.79	17.14	19.00	0.00	0.00	0.66	0.00	
0.29	16.40	1.64	0.73	0.23	0.00	0.33	4.10	
0.00	4.10	4.00	4.43	2.80	3.50	0.99	4.10	
0.00	0.00	4.00	4.43	0.92	3.50	0.00	8.20	
0.58	16.40	1.64	0.73	0.23	0.00	0.33	4.10	
0.00	17.57	17.14	19.00	0.00	12.25	0.00	4.10	
1.45	4.10	17.14	0.73	0.46	0.00	4.00	8.79	
15.14	8.20	8.00	4.43	0.69	0.00	0.00	4.10	
7.07	17.57	8.00	8.87	0.00	37.50	4.00	8.20	
3.53	17.57	17.14	19.00	12.00	0.00	0.00	4.10	
0.00	4.10	4.00	2.22	0.00	3.50	4.00	8.79	
7.07	8.20	0.33	0.73	0.46	0.00	0.00	8.20	
15.14	17.57	17.14	38.00	5.60	1.73	1.97	17.57	
0.58	16.40	0.33	0.36	5.60	3.50	0.33	2.05	
7.07	0.67	6.00	6.65	0.35	0.00	4.00	6.15	
0.00	1.68	6.00	4.43	0.00	0.00	0.00	8.79	
0.00	2.05	6.00	2.22	0.00	22.50	6.00	12.30	
3.53	35.14	25.71	57.00	2.80	0.00	0.66	0.00	
0.00	8.20	8.00	8.87	0.00	7.00	0.00	8.20	
17.57	17.14	19.00	12.00	0.00	0.00	8.20	3.14	
12.30	2.00	2.22	0.00	0.00	0.00	2.05	0.79	
3.53	17.57	17.14	8.87	0.00	15.00	0.00	12.30	
1.74	17.57	17.14	2.19	1.38	0.00	1.97	12.30	
0.00	17.57	17.14	19.00	0.00	30.00	0.00	0.00	
0.29	8.20	16.00	4.43	1.84	28.00	0.00	8.20	
3.53	4.10	0.00	0.00	0.00	0.00	0.00	0.00	
0.00	6.15	6.00	8.87	0.00	0.00	1.64	17.57	
3.53	8.20	17.14	19.00	5.60	15.00	0.00	8.20	
3.53	35.14	17.14	8.87	2.80	3.50	0.00	8.20	
7.07	4.10	8.00	4.43	5.60	15.00	4.00	8.20	
1.16	1.35	1.32	1.46	0.92	3.50	0.33	4.10	
0.00	4.10	4.00	4.43	2.80	0.00	0.00	4.10	
0.67	8.00	4.43	0.00	3.50	0.00	35.14	4.71	
7.07	4.10	4.00	19.00	0.00	15.00	0.00	17.57	
8.20	4.00	4.43	0.69	0.58	4.00	8.20	5.50	
3.53	12.30	8.00	8.87	0.46	45.00	0.00	8.20	
3.53	8.79	17.14	19.00	0.00	0.00	0.66	0.00	

0.00	0.00	1.97	2.19	1.38	7.00	0.00	8.20
0.00	17.57	68.57	38.00	0.00	0.00	0.00	0.00
15.14	17.57	17.14	13.30	0.00	0.00	0.00	8.20
17.57	4.00	0.00	0.00	3.50	0.00	4.10	1.57
8.20	4.00	4.43	0.00	0.00	0.00	4.10	8.64
15.14	52.71	17.14	8.87	2.80	0.00	8.00	8.20
8.79	17.14	19.00	0.00	0.00	0.66	0.00	0.79
0.29	0.00	8.00	0.73	0.00	0.00	0.00	0.00
0.00	8.20	8.00	0.36	0.00	30.00	4.00	17.57
8.20	8.00	13.30	0.00	0.00	0.00	35.14	3.14
8.79	2.00	2.22	0.23	0.00	0.00	2.05	0.79
3.53	17.57	4.00	0.00	0.00	3.50	0.00	4.10
15.14	70.29	34.29	19.00	0.00	15.00	17.14	8.20
45.43	17.57	17.14	19.00	0.00	7.00	0.00	17.57
15.14	17.57	17.14	8.87	2.80	0.00	0.00	8.20
4.10	4.00	4.43	0.23	3.50	0.33	0.34	9.43
52.71	12.00	13.30	0.00	3.50	2.00	4.10	1.57
1.74	8.20	8.00	2.19	1.38	1.73	1.97	8.20
0.29	8.20	16.00	4.43	1.84	28.00	0.00	8.20
0.58	8.20	4.29	0.26	0.00	15.00	8.00	35.14
4.10	12.00	0.73	0.46	0.00	0.00	4.10	16.50
8.20	0.33	95.00	14.00	3.50	0.66	35.14	1.57
0.00	17.57	17.14	4.43	0.00	3.50	0.00	4.10
5.30	10.25	8.00	4.43	1.15	15.00	8.00	4.10
2.05	5.00	2.22	0.00	22.50	17.14	8.79	0.79
0.00	17.57	51.43	57.00	0.00	15.00	0.00	0.00
0.00	4.10	8.00	8.87	2.30	5.25	4.00	8.20
15.14	17.57	17.14	8.87	5.60	15.00	1.97	8.20
35.14	34.29	38.00	2.80	15.00	0.00	0.00	3.14
53.00	35.14	34.29	38.00	0.00	7.00	0.00	52.71
43.93	42.86	47.50	0.00	3.50	4.00	52.71	3.14
0.00	2.05	4.00	4.43	2.80	0.00	0.00	17.57
1.45	4.10	17.14	0.73	0.46	0.00	4.00	17.57
15.14	8.20	34.29	1.46	1.38	7.00	0.00	8.20
35.14	25.71	28.50	0.00	15.00	0.00	12.30	3.93
7.07	0.00	0.00	0.00	0.69	0.00	0.00	8.20
7.07	52.71	1.97	0.00	5.60	0.00	0.00	2.02
1.16	4.10	17.14	4.43	12.00	7.00	0.00	8.20
0.00	17.57	17.14	8.87	0.00	3.50	0.00	4.10
3.53	0.67	8.00	4.43	0.00	3.50	0.00	17.57
4.10	4.00	4.43	0.23	0.00	0.00	0.34	16.50
35.14	25.71	28.50	0.00	0.00	0.00	12.30	3.93
0.87	10.25	10.00	4.43	0.35	0.00	0.00	2.02
1.16	4.10	4.00	2.19	2.80	0.00	0.00	4.10
75.71	35.14	0.33	38.00	2.80	3.50	0.66	52.71
7.07	35.14	8.00	19.00	2.80	7.00	0.00	17.57
0.00	17.57	51.43	38.00	0.00	0.00	0.00	0.00
1.74	17.57	17.14	8.87	0.46	0.00	0.00	17.57
1.45	4.10	4.29	0.26	0.00	0.00	0.00	26.36
0.00	0.00	0.00	8.87	0.00	0.00	0.00	0.00
0.00	0.00	0.00	4.43	0.00	0.00	17.14	35.14
7.07	35.14	25.71	28.50	0.00	15.00	0.00	12.30
16.40	1.64	0.73	0.23	0.00	0.33	4.10	11.00

6.15	6.00	8.87	0.00	0.00	0.00	4.10	4.71
8.20	8.00	8.87	5.60	0.00	0.00	8.20	99.00
0.58	8.20	0.33	95.00	14.00	3.50	0.66	35.14
7.07	8.20	0.00	0.00	0.00	10.50	1.97	0.00
7.07	35.14	8.00	13.30	0.46	7.00	0.00	8.20
35.14	17.14	19.00	0.00	30.00	0.00	17.57	4.71
0.00	1.01	1.97	0.00	0.00	105.00	0.00	4.10
7.07	4.10	0.00	0.00	2.80	7.00	0.00	2.02
3.53	17.57	17.14	8.87	0.92	0.00	0.00	12.30
106.00	17.57	4.00	0.00	0.00	15.00	0.00	10.25
0.00	35.14	34.29	38.00	0.00	30.00	0.00	0.00
0.00	0.34	8.00	8.87	4.20	7.00	0.00	4.10
15.14	35.14	17.14	19.00	0.46	0.00	4.00	17.57
8.20	0.00	0.00	0.00	0.00	0.00	1.01	9.29
0.00	8.20	8.00	8.87	0.69	0.00	1.32	2.02
15.14	2.02	85.71	57.00	1.15	1.73	1.97	8.20
0.73	0.00	0.00	0.00	0.00	37.50	0.00	0.00
7.07	8.20	8.00	1.46	2.80	0.86	1.32	1.35
3.53	17.57	17.14	6.65	0.46	0.00	0.00	8.20
0.00	0.00	0.00	0.00	37.50	0.00	0.00	3.00
0.58	8.20	0.33	38.00	2.80	3.50	0.66	17.57
0.00	8.20	8.00	8.87	24.00	30.00	8.00	8.20
7.07	8.20	8.00	19.00	24.00	15.00	0.00	17.57
0.00	2.05	4.00	4.43	0.00	0.00	0.00	8.79
7.07	4.10	8.00	19.00	2.80	7.00	0.00	8.20
0.00	2.02	1.97	8.87	5.60	0.00	8.00	8.20
7.07	35.14	25.71	28.50	0.00	15.00	0.00	12.30
5.30	6.15	6.00	28.50	0.23	7.00	8.00	0.84
15.14	0.00	4.00	4.43	2.80	15.00	0.00	4.10
3.53	4.10	17.14	4.43	0.00	0.00	4.00	8.20
0.00	8.20	17.14	8.87	0.00	15.00	0.00	17.57
8.20	8.00	8.87	5.60	0.00	0.00	8.20	99.00
1.74	41.00	8.00	0.73	0.46	0.00	0.00	4.10
1.45	4.10	4.00	4.43	0.23	3.50	0.33	0.34
7.07	17.57	17.14	4.43	5.60	0.00	0.33	4.10
0.58	8.20	8.00	13.30	0.00	0.00	0.00	35.14
30.29	35.14	34.29	38.00	5.60	7.00	8.00	35.14
0.58	8.20	8.00	13.30	0.00	15.00	0.00	17.57
3.53	17.57	17.14	4.43	2.80	0.00	0.00	17.57
3.53	4.10	17.14	4.43	0.92	0.58	0.00	17.57
1.16	8.20	8.00	0.73	1.38	0.00	0.00	4.10
7.07	0.67	8.00	4.43	0.00	0.00	0.00	8.79
3.53	17.57	4.00	4.43	0.46	3.50	4.00	4.10
3.53	14.66	115.14	8.87	70.46	3.50	0.00	4.10
1.45	1.68	4.00	4.43	2.80	0.00	0.66	2.36
7.07	17.57	12.00	3.64	0.46	0.00	0.00	4.10
17.57	0.00	0.00	0.00	0.00	0.00	17.57	1.57
7.07	17.57	17.14	19.00	5.60	1.73	1.97	17.57
8.13	8.20	5.59	4.43	0.00	2.01	0.66	20.50
0.00	4.10	4.00	4.43	0.00	0.00	0.00	4.10
0.00	35.14	17.14	19.00	0.00	0.00	0.00	4.10
3.53	17.57	6.00	6.65	0.00	3.50	0.00	4.10
5.30	8.79	4.00	4.43	0.00	0.00	0.00	2.05

0.00	2.02	0.00	2.19	0.00	0.00	0.00	2.70
7.07	8.20	51.43	19.00	5.60	105.00	1.97	17.57
10.60	8.20	12.00	38.00	0.00	15.00	0.00	17.57
35.14	34.29	38.00	2.80	15.00	0.00	0.00	3.14
1.45	4.10	4.00	4.43	0.23	3.50	0.33	0.34
0.00	35.14	25.71	28.50	2.80	0.00	0.66	0.00
3.53	17.57	34.29	19.00	1.38	0.00	8.57	17.57
1.74	17.57	8.00	4.43	0.92	1.73	0.33	8.20
0.00	4.10	4.00	2.22	0.00	0.00	0.00	8.79
30.29	35.14	34.29	19.00	12.00	0.00	0.00	17.57
2.05	1.00	2.22	1.40	0.00	0.00	8.79	0.79
2.05	2.00	2.22	0.35	15.00	0.33	2.05	4.71
8.79	2.00	4.43	0.00	0.00	0.00	2.05	1.57
5.30	8.79	2.00	2.22	0.23	0.00	0.00	2.05
0.29	16.40	1.64	0.73	0.23	0.00	0.33	4.10
0.00	4.10	4.00	4.43	2.80	0.00	0.00	4.10
1.74	8.20	8.00	4.43	0.92	7.00	0.00	4.10
70.29	8.00	8.87	0.00	7.00	8.00	8.20	3.14
0.29	8.20	8.00	38.00	5.60	15.00	0.00	8.20
37.35	65.01	76.57	46.87	32.40	45.00	4.00	69.11
10.60	4.10	4.00	0.36	2.80	0.29	0.00	4.10
4.10	4.00	4.43	0.00	0.00	0.00	4.10	3.14
0.00	70.29	68.57	76.00	0.00	30.00	0.00	0.00
35.14	0.33	38.00	2.80	3.50	0.66	52.71	3.14
0.29	0.34	12.00	13.30	8.40	0.00	0.00	4.10
15.14	17.57	17.14	19.00	0.58	0.00	0.82	8.20
16.40	0.33	0.36	5.60	3.50	0.33	2.05	16.50
15.14	17.57	34.29	4.43	2.80	7.00	0.00	17.57
17.57	51.43	38.00	0.00	0.00	0.00	0.00	2.36
7.07	17.57	17.14	38.00	2.80	3.50	0.00	8.20
0.58	4.10	1.97	1.09	0.92	30.00	0.66	2.02
0.58	16.40	0.33	0.36	5.60	0.00	0.33	4.10
0.00	2.05	4.00	2.22	1.40	0.00	0.00	8.79
16.40	0.33	0.36	0.46	0.00	0.33	4.10	11.00
7.07	0.67	8.00	4.43	0.00	0.00	0.00	17.57
17.57	4.00	4.43	0.00	0.00	0.00	8.20	1.57
1.74	8.20	8.00	8.87	1.38	1.73	1.97	8.20
1.45	52.71	8.00	0.00	5.60	7.00	0.00	8.20
7.07	17.57	17.14	8.87	1.61	7.00	0.00	4.10
30.29	26.36	8.57	9.50	0.00	0.00	0.00	35.14
2.05	8.57	2.22	0.23	0.00	4.00	8.79	3.14
35.14	17.14	19.00	0.00	0.00	0.00	12.30	2.36
1.16	17.57	8.00	4.43	0.92	3.50	4.00	4.10
8.20	17.14	19.00	2.80	7.00	0.00	17.57	11.00
0.00	0.67	2.00	6.65	0.46	0.00	0.00	2.70
3.53	8.79	17.14	19.00	0.00	0.00	0.66	0.00
15.14	17.57	17.14	8.87	5.60	0.00	0.00	17.57
3.53	4.10	17.14	4.43	0.92	0.58	0.00	17.57
3.53	8.20	17.14	19.00	5.60	15.00	0.00	8.20
3.53	4.10	8.00	1.46	0.69	15.00	0.00	4.10
0.00	0.67	0.00	0.00	0.69	0.00	0.00	2.05
1.74	8.20	8.00	8.87	1.38	7.00	0.00	8.20
2.05	5.00	2.22	1.40	0.00	17.14	8.79	0.79

2.90	8.20	4.00	8.87	5.60	0.00	0.00	8.20
7.07	8.20	8.00	8.87	5.60	0.00	0.00	8.20
15.14	17.57	17.14	13.30	2.80	7.00	12.00	12.30
0.00	8.00	8.87	5.60	0.00	0.00	0.00	2.20
30.29	17.57	17.14	19.00	0.46	15.00	0.99	17.57
4.10	4.00	4.43	11.20	0.00	0.00	0.00	6.29
3.53	8.20	8.00	22.17	0.00	0.00	0.00	17.57
1.74	17.57	17.14	19.00	2.80	0.00	0.00	4.10
7.07	17.57	8.00	38.00	0.00	7.00	0.00	17.57
2.05	2.00	2.22	0.00	15.00	0.00	26.36	3.14
8.20	4.29	0.26	0.00	15.00	8.00	35.14	11.00
0.58	8.20	0.33	0.36	1.40	1.75	0.66	2.05
15.14	4.10	4.00	8.87	0.46	0.00	1.97	2.02
0.29	4.10	8.00	4.43	2.80	0.00	0.00	10.25
3.53	17.57	4.00	0.00	0.00	3.50	0.00	4.10
7.07	17.57	0.00	8.87	0.46	0.00	0.00	17.57
0.00	0.00	8.00	0.73	0.00	0.00	0.00	0.00
1.45	4.10	4.00	4.43	0.23	3.50	0.33	0.34
3.53	17.57	4.00	4.43	0.46	3.50	4.00	4.10
15.14	17.57	0.82	11.08	0.76	5.25	0.00	0.84
3.53	8.79	17.14	19.00	0.00	0.00	0.66	0.00
3.53	17.57	17.14	19.00	2.80	7.00	0.00	17.57
3.53	4.10	8.00	4.43	2.80	0.29	0.33	4.10
30.29	17.57	17.14	8.87	2.80	15.00	4.00	4.10
0.00	35.14	25.71	28.50	2.80	0.00	0.66	0.00
3.53	4.10	0.00	0.00	0.00	0.00	0.00	0.00
10.60	52.71	12.00	13.30	0.00	3.50	2.00	4.10
8.20	17.14	19.00	5.60	0.00	0.00	26.36	3.14
0.29	4.10	4.00	4.43	2.80	0.00	0.00	0.00
7.07	4.10	34.29	8.87	0.00	3.50	0.00	4.10
7.07	17.57	8.00	19.00	0.46	15.00	1.97	8.20
3.53	17.57	4.00	4.43	0.00	0.00	0.00	8.20
3.53	8.20	17.14	19.00	0.69	0.00	0.00	4.10
5.30	12.30	2.00	2.22	0.00	0.00	0.00	2.05
3.53	4.10	10.00	0.73	0.35	7.00	0.66	1.52
0.00	2.05	2.00	2.22	0.00	22.50	17.14	8.79
75.71	52.71	34.29	57.00	8.40	0.00	0.00	52.71
3.53	8.20	4.00	8.87	1.38	3.50	0.00	12.30
0.00	35.14	34.29	76.00	2.80	15.00	0.00	0.00
7.07	4.10	17.14	19.00	0.69	15.00	0.99	17.57
3.53	17.57	17.14	19.00	0.00	0.00	17.14	17.57
0.00	70.29	4.00	8.87	1.38	45.00	8.00	8.20
37.86	26.36	25.71	38.00	0.00	3.50	0.00	17.57
71.17	81.83	145.14	84.87	62.00	75.00	8.00	80.03
7.07	0.67	8.00	4.43	4.20	5.25	0.00	4.10
15.14	17.57	17.14	4.43	0.58	8.75	0.00	35.14
10.60	52.71	12.00	13.30	0.00	3.50	2.00	4.10
8.79	2.00	2.22	0.23	0.00	0.00	2.05	0.79
3.53	17.57	4.00	4.43	0.00	0.00	0.00	4.10
0.58	4.10	4.00	2.19	0.92	37.50	0.33	8.20
3.53	17.57	8.00	8.87	2.80	3.50	0.00	4.10
0.29	0.00	12.00	13.30	8.40	10.50	0.00	0.00
30.29	8.20	8.00	0.00	0.92	105.00	0.00	1.01

15.14	12.30	4.00	8.87	2.80	10.50	0.00	17.57
0.00	4.10	4.00	4.43	0.00	0.00	0.00	4.10
8.79	6.00	6.65	0.00	3.50	0.00	4.10	3.14
7.07	8.20	4.00	8.87	2.80	0.00	0.00	0.00
0.29	0.34	4.00	4.43	2.80	3.50	0.00	4.10
0.58	0.00	6.00	4.43	2.80	0.00	0.00	0.00
8.13	8.20	5.59	4.43	0.00	2.01	0.66	20.50
1.74	17.57	1.97	3.64	0.00	7.00	0.00	35.14
0.00	4.10	4.00	19.00	2.07	10.50	0.00	4.10
0.00	8.79	8.57	9.50	0.00	30.00	0.00	0.00
7.07	0.67	8.00	4.43	0.00	14.00	0.00	26.36
0.29	16.40	1.64	0.73	0.23	0.00	0.33	4.10
5.30	8.79	2.00	4.43	0.00	0.00	0.00	2.05
3.53	35.14	25.71	57.00	2.80	0.00	0.66	0.00
3.53	4.10	4.00	4.43	0.69	60.00	0.00	4.10
0.00	4.10	4.00	4.43	0.00	10.50	0.00	8.20
0.58	8.20	8.00	13.30	0.00	0.00	0.00	8.20
1.45	4.10	4.00	4.43	0.23	3.50	0.33	0.34
3.53	8.79	17.14	19.00	0.00	0.00	0.66	0.00
3.53	8.20	8.00	8.87	0.00	8.75	8.00	4.10
35.14	17.14	19.00	0.00	0.00	0.00	8.20	0.79
0.00	4.10	4.00	8.87	0.58	0.00	0.00	26.36
45.43	8.20	17.14	4.43	0.92	7.00	0.00	8.20
30.29	52.71	68.57	0.00	1.38	0.00	1.97	12.30
0.00	35.14	34.29	76.00	2.80	15.00	0.00	0.00
3.53	4.10	17.14	19.00	0.23	0.00	1.97	17.57
0.29	16.40	1.64	0.73	0.23	0.00	0.33	4.10
1.16	1.35	8.00	2.19	0.92	0.00	0.00	8.20
0.58	4.10	4.00	4.43	2.80	10.50	4.00	0.00
3.53	4.10	4.00	8.87	0.00	0.00	0.00	4.10
15.14	35.14	34.29	19.00	2.80	30.00	17.14	17.57

gooshtega	gooshtego	gooshtecha	morgh	mahi	tonemahi	jegar	hamberger
4.71	0.21	1.86	36.43	1.94	1.48	0.37	1.07
4.71	0.37	1.86	24.29	6.75	0.49	0.00	0.09
4.71	0.00	1.86	60.71	0.28	0.00	0.00	0.00
0.79	0.18	0.93	42.50	1.69	0.49	0.00	0.18
0.00	1.57	5.57	48.57	3.37	3.00	0.06	0.00
0.00	0.73	0.87	36.43	1.94	0.00	0.37	0.00
1.57	0.37	1.86	36.43	6.75	0.49	0.00	0.18
0.00	3.14	0.00	36.43	28.91	0.00	0.12	0.00
0.18	0.93	42.50	1.69	0.49	0.00	0.18	0.00
11.00	1.57	1.86	24.29	0.00	0.00	0.00	0.00
22.00	26.00	24.29	6.75	15.00	1.50	4.33	0.27
4.71	0.37	1.86	24.29	14.46	1.23	0.18	0.53
66.00	5.57	18.86	13.49	1.33	0.75	0.36	0.05
0.00	0.73	0.00	36.43	3.37	3.00	0.06	0.18
1.57	0.00	0.00	12.14	0.00	0.00	0.00	9.29
1.57	0.37	1.86	24.29	6.75	0.49	0.00	0.18
11.00	4.71	5.57	18.21	0.55	12.86	0.12	0.18
0.73	1.57	0.87	12.14	14.46	0.00	0.00	2.17
1.57	1.57	1.86	2.83	3.37	0.00	0.00	0.00
11.00	1.57	14.86	24.29	0.28	12.86	1.00	0.18
4.71	0.37	0.43	2.83	0.00	0.00	0.00	0.00
0.79	22.00	29.71	12.14	5.06	9.00	0.00	10.83
3.14	3.14	3.71	24.29	3.37	0.37	0.06	0.89
0.00	1.57	5.57	12.14	1.39	0.00	0.00	0.00
4.71	0.37	1.86	24.29	6.75	0.49	0.00	0.09
0.79	7.86	11.14	72.86	1.69	15.00	0.75	2.17
11.00	11.00	3.71	24.29	14.46	0.00	0.00	0.00
3.14	1.57	1.86	24.29	1.66	0.00	0.00	0.00
16.50	2.36	7.43	30.36	0.28	38.57	1.00	0.18
7.86	7.86	5.57	48.57	0.28	18.00	0.00	6.50
1.57	6.29	5.57	36.43	1.69	6.00	0.75	7.58
1.57	11.00	21.36	60.71	3.37	15.00	1.50	2.17
1.57	0.37	1.86	36.43	6.75	0.49	0.00	0.18
4.71	1.57	3.71	24.29	1.11	3.00	0.00	0.00
3.14	5.57	48.57	14.46	3.00	0.00	0.00	0.33
0.73	0.87	42.50	3.37	0.00	0.00	0.00	0.00
0.00	7.86	1.30	48.57	0.83	0.49	0.25	0.00
0.00	4.71	5.57	36.43	1.66	1.48	0.37	1.07
1.57	0.00	1.86	36.43	0.00	0.00	0.00	0.00
1.10	0.00	14.30	0.72	28.91	3.33	0.62	1.07
7.86	0.00	4.64	12.14	0.00	0.00	0.00	0.00
9.43	11.00	13.00	72.86	6.75	6.00	1.50	4.33
3.14	1.57	9.29	14.17	0.55	3.00	0.00	6.50
0.00	3.14	1.86	12.14	0.00	3.00	0.18	0.00
0.00	3.14	1.86	24.29	14.46	0.00	0.12	2.17
1.57	3.14	1.86	24.29	0.55	0.00	0.18	0.00
4.71	4.71	5.57	24.29	3.37	0.00	0.00	0.00
7.86	9.29	60.71	3.37	15.00	0.75	7.58	0.27
4.71	3.14	1.86	0.00	0.55	0.00	0.00	0.00
23.57	6.93	18.86	21.69	0.77	6.00	0.53	0.66
11.00	11.00	26.00	85.00	1.66	6.00	0.00	0.18
1.57	0.37	0.93	42.50	1.69	0.49	0.00	0.18

1.57	0.73	5.57	0.00	6.75	1.48	0.37	0.00
3.93	0.73	13.43	85.00	0.00	0.00	0.00	0.00
4.71	4.71	5.57	12.14	0.00	0.00	0.00	0.00
0.73	0.87	36.43	3.37	0.00	0.00	0.00	0.00
3.93	0.00	42.50	0.00	12.86	0.00	9.29	1.00
3.14	1.57	0.00	12.14	28.91	0.00	0.00	2.17
0.18	0.93	42.50	1.69	0.49	0.00	0.18	0.00
3.14	3.14	0.00	24.29	14.46	0.00	0.06	0.18
9.43	9.43	2.17	2.20	6.75	1.33	1.50	2.17
0.73	3.71	48.57	6.75	6.00	1.50	6.50	0.00
0.73	0.87	42.50	3.37	0.00	0.00	0.00	0.05
1.57	0.73	0.87	36.43	3.37	0.00	0.00	0.00
4.71	1.57	5.57	12.14	0.00	0.00	0.00	0.00
1.57	1.57	4.64	72.86	43.37	3.00	0.00	0.00
0.00	4.71	1.86	36.43	0.00	0.00	0.00	0.00
66.00	5.57	18.86	13.49	1.33	0.75	0.36	0.05
11.00	0.87	24.29	1.39	0.00	0.00	0.36	0.00
4.71	0.18	5.57	36.43	1.66	1.48	0.31	0.89
1.10	0.00	14.30	0.72	28.91	3.33	0.62	1.07
11.00	22.00	3.71	60.71	0.55	3.00	0.75	2.17
0.00	14.86	28.29	1.11	0.71	1.85	2.17	10.67
1.57	1.86	85.00	1.52	6.43	1.00	0.18	2.00
1.57	1.57	0.00	0.00	0.00	0.00	0.00	0.00
0.00	4.71	3.71	24.29	3.37	0.49	0.25	0.00
11.00	9.29	60.71	3.37	13.50	0.38	6.50	0.55
4.71	0.00	13.00	85.00	0.00	0.00	0.00	0.00
0.00	0.00	1.86	36.43	6.75	3.00	0.12	2.17
1.57	1.57	5.57	36.43	6.75	1.48	0.37	0.53
0.37	1.86	48.57	6.75	0.49	0.00	0.00	0.00
1.57	1.57	5.57	36.43	28.91	1.50	0.00	0.00
1.57	4.64	72.86	43.37	4.50	0.00	0.00	0.05
7.86	14.30	16.90	72.86	3.37	10.50	0.75	2.17
1.57	16.50	30.64	12.14	5.06	9.00	0.75	4.33
0.00	4.71	5.57	48.57	14.46	25.71	1.50	0.00
0.92	0.93	42.50	6.75	0.49	0.00	0.09	0.00
0.18	0.18	0.87	36.43	1.94	0.00	0.37	0.00
4.71	0.00	5.57	12.14	43.37	0.00	1.50	4.33
4.71	0.00	1.30	24.29	0.00	0.00	0.00	0.00
1.57	0.00	3.71	24.29	0.28	6.00	0.00	0.00
7.86	5.50	6.50	42.50	3.37	15.00	1.50	4.33
2.36	32.50	60.71	0.55	15.00	0.75	2.17	0.00
0.92	0.93	42.50	3.37	0.74	0.00	0.18	0.00
1.57	1.57	3.71	24.29	1.11	0.99	3.21	0.00
6.60	6.60	2.17	2.20	3.37	0.77	0.25	0.71
3.14	1.57	3.71	97.14	6.75	0.25	1.00	0.18
1.57	0.73	0.87	36.43	1.66	0.74	0.37	0.00
3.14	0.73	13.87	85.00	0.00	0.00	0.00	0.00
0.00	3.14	5.57	18.21	3.37	3.00	0.00	0.00
7.86	11.00	16.71	24.29	5.06	9.00	0.75	2.17
4.71	4.71	1.86	24.29	14.46	0.00	0.00	0.00
4.71	1.57	3.71	12.14	14.46	3.00	0.18	0.00
3.93	0.92	0.93	42.50	6.75	0.49	0.00	0.09
3.14	7.43	12.14	0.55	12.86	0.12	0.18	0.67

0.37	0.00	85.00	0.00	12.86	0.00	9.29	1.00
14.14	3.71	4.71	6.75	2.00	1.50	0.36	2.00
0.79	1.57	1.86	85.00	1.52	6.43	1.00	0.18
3.14	0.00	1.86	24.29	14.46	3.00	0.00	0.00
3.14	3.14	3.71	12.14	6.75	3.00	0.12	0.00
1.57	3.71	36.43	5.06	0.00	0.00	0.00	0.00
0.00	0.00	1.86	24.29	0.00	0.00	0.00	0.00
0.00	0.00	1.86	60.71	0.00	0.00	0.00	0.00
0.00	4.71	3.71	36.43	1.39	2.47	0.25	2.17
0.00	0.00	0.87	12.14	6.75	12.86	0.12	0.00
7.86	0.00	1.86	60.71	0.00	6.00	0.00	0.00
3.00	0.00	0.43	92.08	0.00	0.00	0.00	18.57
3.14	3.14	3.71	12.14	0.00	3.21	0.00	0.00
0.00	0.87	24.29	0.00	0.00	0.00	0.00	0.00
0.00	4.71	5.57	36.43	1.39	0.00	0.00	0.00
0.73	1.57	9.29	12.14	6.75	6.00	5.64	1.07
3.00	3.93	0.00	18.21	0.00	0.00	0.00	18.57
0.18	6.29	13.00	36.43	1.11	0.49	0.18	0.36
4.71	4.71	1.86	12.14	0.55	7.50	0.00	0.00
3.93	0.00	18.21	0.00	0.00	0.00	18.57	4.29
2.36	1.57	3.71	97.14	6.75	0.49	1.00	0.18
4.71	0.73	5.57	36.43	14.46	6.00	1.50	0.00
0.00	7.86	5.57	12.14	1.39	0.00	0.31	0.00
1.57	6.29	5.57	36.43	1.69	6.00	1.50	4.33
3.14	3.14	3.71	24.29	0.00	3.00	0.00	2.17
4.71	1.57	1.86	24.29	28.91	25.71	0.00	2.17
4.71	0.92	1.86	24.29	6.75	0.49	0.00	0.09
0.00	27.50	1.86	30.36	6.75	0.25	0.15	0.45
1.57	1.57	1.30	30.36	6.75	0.00	0.00	0.00
1.57	1.10	1.86	17.00	1.39	3.00	1.50	4.33
0.00	4.71	7.43	5.67	3.37	0.00	0.00	0.00
14.14	3.71	4.71	6.75	2.00	1.50	0.36	2.00
9.43	9.43	3.71	4.71	6.75	1.33	0.00	0.71
9.43	66.00	5.57	18.86	13.49	1.33	0.75	0.36
4.71	0.06	7.43	24.29	14.46	1.50	0.18	0.53
3.14	0.73	3.71	48.57	3.37	15.00	0.75	4.33
3.14	1.57	1.86	48.57	36.14	3.00	0.00	0.00
1.57	1.83	3.71	48.57	6.75	6.00	1.50	6.50
11.00	1.57	3.71	24.29	14.46	1.48	0.25	0.00
4.71	9.43	5.57	14.14	14.46	2.86	0.12	0.71
1.57	3.14	5.57	2.83	14.46	3.00	0.12	0.00
1.57	7.86	9.29	60.71	3.37	15.00	0.75	7.58
0.00	3.14	3.71	24.29	3.37	0.25	0.06	0.18
1.57	3.14	1.86	36.43	43.37	25.71	0.37	0.00
3.14	0.18	1.30	36.43	1.66	0.49	0.00	0.00
4.71	2.36	1.86	12.14	3.37	0.00	0.00	0.89
1.57	1.86	12.14	5.06	0.00	0.00	0.00	0.00
4.71	4.71	3.71	24.29	28.91	12.86	0.75	0.00
0.27	13.20	4.33	11.30	20.24	0.27	0.31	2.17
1.57	1.57	1.86	24.29	14.46	3.00	0.25	0.53
3.14	0.73	13.43	85.00	0.00	0.00	0.00	0.00
1.57	0.73	0.87	85.00	3.37	0.00	0.00	0.00
0.79	0.73	0.87	42.50	3.37	0.00	0.00	0.00

0.00	7.86	7.43	24.29	14.46	1.48	0.75	0.00
4.71	1.57	3.71	36.43	1.66	0.00	0.31	0.00
3.14	3.14	5.57	24.29	1.39	0.00	0.18	0.00
0.37	1.86	48.57	6.75	0.49	0.00	0.00	0.00
9.43	66.00	5.57	18.86	13.49	1.33	0.75	0.36
2.36	0.37	4.64	54.64	6.75	0.49	0.00	0.18
0.73	0.73	3.71	18.21	3.37	3.00	0.09	0.00
3.14	3.14	3.71	48.57	3.37	1.48	0.37	0.36
0.79	7.86	11.14	72.86	1.69	15.00	0.75	2.17
1.57	4.71	5.57	36.43	28.91	0.00	0.00	0.00
33.00	7.43	48.57	3.37	13.50	0.38	6.50	0.55
3.14	1.86	24.29	0.55	12.86	3.21	9.29	2.86
0.37	0.87	42.50	6.75	0.00	0.00	0.00	0.05
0.79	0.73	0.87	24.29	6.75	0.00	0.00	0.00
11.00	3.14	7.43	12.14	0.55	12.86	0.12	0.18
4.71	4.71	3.71	5.67	1.66	1.48	0.18	0.00
0.00	0.12	7.43	36.43	14.46	0.00	0.00	0.00
11.00	0.87	36.43	1.39	25.71	0.00	0.36	0.00
0.73	4.71	5.57	36.43	14.46	6.00	0.18	4.33
7.86	14.46	19.00	127.50	21.62	7.73	0.87	4.33
1.57	1.57	3.71	36.43	6.75	0.00	0.00	0.00
0.00	3.71	24.29	0.00	12.86	0.00	9.29	1.00
4.71	0.00	1.86	60.71	0.00	0.00	0.00	0.00
1.57	3.71	97.14	6.75	0.25	1.00	0.18	0.05
9.43	4.71	11.14	24.29	0.00	12.86	0.00	9.29
0.00	4.71	1.86	18.21	14.46	0.25	0.00	2.17
3.93	8.36	12.14	0.28	45.00	1.00	0.18	2.00
3.14	3.14	3.71	24.29	6.75	3.00	0.37	0.89
1.10	13.87	85.00	0.00	0.00	0.00	0.00	0.00
1.57	0.73	0.87	36.43	6.75	0.74	0.12	0.18
0.00	3.14	3.71	5.67	3.37	0.00	0.00	0.00
11.00	4.71	9.29	24.29	0.28	25.71	1.00	0.18
7.86	7.86	9.29	60.71	3.37	6.00	1.50	4.33
4.71	9.29	24.29	0.28	25.71	1.00	0.18	1.00
3.14	9.43	9.29	60.71	3.37	15.00	0.75	7.58
3.93	0.87	42.50	0.55	25.71	0.00	0.36	0.00
4.71	0.18	5.57	36.43	14.46	0.00	0.18	1.07
0.00	1.57	0.87	36.43	0.00	0.00	0.00	0.00
3.14	3.14	3.71	24.29	6.75	3.00	0.25	0.00
3.93	3.93	4.64	60.71	36.14	3.00	0.06	0.18
11.00	23.21	66.79	10.12	12.00	0.00	2.17	0.11
0.18	0.93	42.50	1.69	0.49	0.00	0.18	0.00
0.12	3.14	0.43	5.67	0.83	3.00	0.12	0.18
0.00	3.71	14.17	28.91	12.00	0.00	2.17	0.67
22.00	3.14	26.00	48.57	0.28	18.00	1.50	4.33
1.57	0.37	1.86	24.29	1.69	0.49	0.00	0.18
0.00	1.57	1.86	2.83	1.11	3.00	0.75	0.00
4.71	9.43	5.57	14.14	14.46	2.86	0.12	0.71
1.57	1.57	9.29	5.67	1.11	12.00	0.75	6.50
1.57	3.14	3.71	12.14	3.37	0.74	0.12	0.18
0.00	9.43	1.86	24.29	4.44	0.00	0.00	0.00
1.57	0.18	1.86	12.14	6.75	0.00	0.37	1.07
33.00	7.43	48.57	3.37	13.50	0.38	6.50	0.55

0.73	3.14	3.71	12.14	14.46	12.86	0.25	0.00
4.71	4.71	5.57	36.43	5.06	0.00	0.00	0.00
1.10	6.29	3.71	36.43	28.91	9.00	0.00	0.00
2.20	1.86	12.14	14.46	0.00	0.00	0.00	1.33
3.14	3.14	3.71	24.29	14.46	0.00	0.12	0.36
1.47	3.71	14.17	0.28	15.00	1.50	10.83	0.00
9.43	11.00	13.00	72.86	6.75	15.00	1.50	4.33
9.43	0.00	3.71	12.14	0.00	6.00	0.00	0.00
0.18	0.18	0.87	24.29	6.75	6.00	0.75	0.00
22.00	26.00	24.29	6.75	10.50	1.13	4.33	0.27
11.00	13.00	60.71	0.55	12.00	1.50	4.33	14.29
16.50	2.36	7.43	30.36	0.28	19.29	1.00	0.18
0.00	4.71	5.57	36.43	6.75	0.00	0.25	1.07
0.00	3.14	4.64	12.14	6.75	0.00	0.12	0.00
1.57	0.37	0.43	36.43	3.37	0.00	0.00	0.00
1.57	1.57	3.71	24.29	0.00	0.00	0.00	0.00
3.14	3.14	3.71	24.29	14.46	0.00	0.00	0.00
9.43	66.00	5.57	18.86	13.49	1.33	0.75	0.36
0.00	3.14	3.71	24.29	3.37	0.25	0.06	0.18
0.00	0.73	1.86	85.00	14.46	4.50	0.06	0.27
1.57	0.37	1.86	24.29	1.69	0.49	0.00	0.18
3.14	4.71	3.71	36.43	6.75	6.00	0.75	0.00
2.20	2.20	3.03	1.10	1.66	0.00	0.18	1.25
3.14	3.14	3.71	48.57	0.00	0.00	0.00	0.00
2.36	0.37	4.64	54.64	6.75	0.49	0.00	0.18
1.57	1.57	1.86	18.21	0.00	0.00	0.00	0.00
3.14	11.00	0.87	36.43	1.39	0.00	0.00	0.36
0.00	3.71	2.83	14.46	3.00	0.00	4.33	1.33
0.37	0.00	0.65	0.00	0.00	0.00	0.00	0.00
3.14	0.73	3.71	24.29	0.28	6.00	0.00	0.00
4.71	1.57	3.71	85.00	14.46	6.00	0.37	4.33
1.57	3.93	0.87	42.50	0.55	25.71	0.00	0.36
0.00	4.71	0.87	12.14	14.46	0.00	0.00	0.00
0.79	0.73	0.87	42.50	3.37	0.00	0.00	0.00
4.71	4.71	3.71	36.43	14.46	0.00	0.00	0.00
0.79	11.00	13.00	12.14	10.12	24.00	1.13	4.33
4.71	1.57	1.86	42.50	50.60	3.00	0.00	0.00
3.14	0.00	0.00	36.43	6.75	6.00	0.12	0.00
1.57	0.37	1.86	60.71	6.75	0.00	0.00	0.00
1.57	3.14	3.71	12.14	1.11	0.74	0.18	0.36
1.57	1.57	1.86	36.43	1.66	0.00	0.37	0.00
0.00	0.37	0.43	85.00	3.37	0.00	0.00	0.00
3.14	3.14	0.43	48.57	43.37	4.50	0.75	0.00
11.00	24.20	32.44	128.57	28.78	14.96	1.75	8.67
4.71	4.71	5.57	36.43	6.75	12.00	0.75	2.17
0.00	7.07	2.79	12.14	0.55	3.00	0.06	0.00
1.57	11.00	0.87	24.29	1.39	0.00	0.00	0.36
0.73	0.87	42.50	3.37	0.00	0.00	0.00	0.05
1.57	3.14	0.00	0.00	6.75	0.00	0.25	0.00
0.00	3.14	6.50	18.21	1.11	0.00	0.00	0.00
1.57	11.00	0.87	42.50	0.55	25.71	0.00	0.36
6.29	0.00	3.71	24.29	28.91	0.00	0.00	0.00
3.14	0.00	1.86	48.57	0.00	0.00	0.37	0.00

1.57	1.57	5.57	48.57	0.83	0.49	0.00	0.00
3.14	3.14	3.71	11.33	28.91	0.00	0.00	0.27
0.73	0.87	42.50	3.37	0.00	0.00	0.00	0.00
6.29	1.47	3.71	14.17	0.28	15.00	0.00	10.83
2.30	0.00	0.00	8.50	0.00	12.86	0.00	9.29
2.20	2.20	1.86	12.14	14.46	0.00	0.00	0.00
0.27	13.20	4.33	11.30	20.24	0.27	0.31	2.17
0.18	11.00	1.86	5.67	1.39	0.00	0.00	0.00
0.00	0.73	1.73	8.50	0.83	0.00	0.00	0.00
4.71	0.00	1.86	60.71	0.28	0.00	0.00	0.00
3.14	7.86	9.29	60.71	3.37	15.00	0.75	7.58
11.00	4.71	5.57	18.21	0.55	12.86	0.12	0.18
3.14	0.73	0.87	24.29	1.39	0.00	0.00	0.00
3.14	0.37	1.86	30.36	6.75	0.49	0.00	0.18
3.14	4.71	3.71	24.29	3.37	0.25	0.18	0.00
6.29	2.93	5.57	48.57	0.00	1.48	0.18	0.00
1.57	2.36	0.00	30.36	0.00	12.86	0.00	9.29
9.43	66.00	5.57	18.86	13.49	1.33	0.75	0.36
1.57	0.37	0.93	42.50	1.69	0.49	0.00	0.18
0.00	4.71	5.57	12.14	0.00	0.00	0.06	0.00
0.18	0.93	42.50	1.69	0.49	0.00	0.18	0.00
3.14	22.00	26.00	24.29	6.75	6.00	1.50	4.33
3.14	1.57	1.86	12.14	10.12	0.00	0.00	0.00
4.71	3.14	3.71	48.57	6.75	0.00	0.37	0.00
1.57	0.37	1.86	60.71	6.75	0.00	0.00	0.00
0.73	6.29	3.71	24.29	14.46	0.00	0.12	0.18
11.00	3.14	9.29	24.29	0.55	12.86	0.12	0.18
4.71	0.12	5.57	36.43	6.75	1.23	0.00	0.00
1.57	1.57	1.86	48.57	1.39	0.00	0.00	0.00
4.71	3.14	3.71	24.29	3.37	3.00	0.00	0.00
0.00	4.71	3.71	85.00	28.91	0.00	0.00	0.00

kalbas	sosis	tokhmemo	sirabi	zaban	maghz	kalle	pache
0.33	0.99	22.89	0.14	1.01	0.25	0.82	0.43
0.00	0.00	19.07	0.00	0.00	0.25	0.00	0.14
0.00	0.00	15.26	0.00	0.00	0.00	0.00	0.00
0.00	0.00	15.26	0.00	0.00	0.25	0.00	0.00
0.16	0.49	7.63	0.00	0.50	0.25	0.55	0.28
0.33	0.99	7.63	0.00	0.00	0.00	0.00	0.00
0.00	0.00	22.89	0.00	0.00	0.25	0.00	0.00
0.00	0.00	1.78	0.00	0.00	0.00	0.00	0.85
0.00	19.07	0.00	0.00	0.25	0.00	0.00	0.00
0.00	0.00	1.78	0.00	0.00	0.00	0.00	0.00
17.14	15.26	0.00	0.00	0.00	0.27	0.00	0.00
0.11	3.62	15.26	0.00	0.25	0.25	0.27	0.14
0.16	15.26	0.03	0.25	0.00	0.00	0.28	1.44
0.33	0.99	3.56	0.67	0.76	0.25	0.82	0.43
1.00	0.00	8.01	0.33	0.00	0.00	0.00	0.00
0.00	0.00	22.89	0.00	0.00	0.25	0.00	0.00
1.00	3.00	30.51	0.03	1.53	0.00	1.67	0.28
0.00	0.00	3.56	0.33	1.01	0.25	1.10	0.57
0.00	0.00	0.00	0.05	0.50	0.25	0.55	0.28
1.00	3.00	15.26	0.03	1.53	0.00	1.67	0.28
0.00	0.00	22.89	0.00	0.00	0.00	0.00	0.00
0.27	8.57	22.89	0.00	0.00	0.49	0.55	1.73
0.00	0.00	7.63	0.00	0.25	0.25	0.27	0.14
0.00	0.00	3.56	0.05	0.50	0.25	0.55	0.28
0.00	0.00	19.07	0.00	0.00	0.25	0.00	0.14
0.11	17.14	7.63	0.00	13.14	0.49	0.55	1.73
0.00	0.00	19.07	0.05	0.25	0.00	0.00	0.00
0.00	0.00	22.89	0.00	0.00	0.00	0.00	0.00
2.00	4.00	22.89	0.03	3.07	0.49	1.67	0.28
0.27	6.00	5.34	0.33	0.00	0.00	0.00	0.28
0.27	30.00	26.70	0.33	3.07	0.00	0.00	1.73
0.11	25.71	11.44	0.67	0.00	3.00	0.00	0.00
0.00	0.00	22.89	0.00	0.00	0.25	0.00	0.00
1.33	4.00	3.56	0.00	0.00	0.00	0.00	0.00
0.00	30.51	0.00	0.00	0.24	0.27	0.14	1.23
0.00	5.34	0.00	0.00	0.00	0.00	0.00	0.00
0.00	0.00	8.90	0.14	0.50	0.25	0.55	0.28
0.33	0.99	22.89	0.16	1.01	0.49	0.55	0.28
0.00	0.00	30.51	0.00	0.00	0.00	0.00	0.00
1.10	0.99	23.14	2.74	0.25	0.49	0.55	0.71
1.00	2.00	7.63	0.00	0.00	0.00	0.00	0.00
0.27	42.86	38.14	0.00	0.00	0.00	0.00	0.14
1.33	0.00	30.51	0.33	0.25	3.00	0.00	0.00
0.22	0.66	22.89	0.00	0.00	0.00	0.55	0.00
2.86	8.57	15.26	0.05	0.00	0.00	0.00	0.14
0.00	0.00	3.56	0.05	0.00	0.00	0.00	0.00
0.00	0.00	7.63	0.00	0.00	0.00	0.00	0.00
4.29	22.89	0.67	6.13	0.00	0.00	1.73	6.16
0.00	0.00	1.78	0.03	0.50	0.00	0.55	0.28
1.32	45.77	0.27	2.27	1.48	3.33	1.00	20.00
0.33	0.99	3.56	0.03	0.25	0.25	0.27	0.14
0.00	0.00	19.07	0.00	0.00	0.25	0.00	0.00

0.33	0.99	22.89	0.16	0.00	0.00	0.00	0.00
0.00	0.00	11.44	0.00	0.00	0.00	0.00	0.00
0.33	0.99	5.34	0.00	0.00	0.00	0.00	0.00
0.00	3.56	0.00	0.00	0.00	0.00	0.14	1.23
0.00	26.70	0.33	0.00	0.00	0.00	0.00	7.40
0.00	0.00	22.89	0.00	0.00	0.00	0.00	0.00
0.00	15.26	0.00	0.00	0.25	0.00	0.00	0.00
0.00	0.00	22.89	0.00	0.00	0.00	0.00	0.00
14.67	10.00	0.00	0.00	0.00	0.00	1.64	0.00
8.57	15.26	0.00	6.13	6.00	0.00	0.00	45.00
0.16	19.07	0.00	0.00	0.00	0.00	0.00	2.47
0.00	0.00	3.56	0.00	0.00	0.00	0.00	0.14
0.00	0.00	7.63	0.05	0.50	0.25	0.82	0.43
0.00	0.00	22.89	0.00	3.07	0.24	0.27	0.00
0.16	0.49	3.56	0.00	0.00	0.00	0.00	0.00
0.16	15.26	0.03	0.25	0.00	0.00	0.28	1.44
0.00	22.89	0.00	0.00	0.00	0.00	0.14	0.00
0.27	0.82	22.89	0.11	0.76	0.25	1.10	0.57
1.10	0.99	23.14	2.74	0.25	0.49	0.55	0.71
2.86	8.57	15.26	0.00	0.00	0.00	0.00	1.73
10.00	44.50	0.55	6.13	3.00	13.33	0.14	3.29
4.00	30.51	0.03	3.07	0.49	1.67	0.28	2.47
0.00	0.00	7.63	0.00	0.00	0.00	0.00	0.00
2.00	2.00	15.26	1.33	0.00	0.25	0.27	0.14
17.14	30.51	0.00	0.00	0.00	0.68	1.73	0.00
0.00	0.00	22.89	0.00	0.00	0.00	0.00	0.00
0.67	4.00	22.89	0.00	0.00	0.00	0.00	0.00
0.16	8.57	22.89	0.16	0.76	0.74	0.82	5.43
0.00	22.89	0.00	0.00	0.00	0.00	0.00	0.00
0.00	0.00	26.70	0.00	3.07	0.24	0.00	0.00
0.16	22.89	0.00	0.25	0.24	0.27	0.00	1.23
0.11	34.29	11.44	0.67	3.07	3.00	0.00	0.00
0.27	8.57	22.89	0.00	0.00	0.49	0.55	1.73
2.86	8.57	15.26	0.00	0.00	0.00	0.00	0.28
0.00	19.07	0.00	0.00	0.25	0.00	0.14	0.00
1.33	4.00	22.89	0.00	0.00	0.00	0.00	0.00
0.33	4.00	22.89	0.16	0.76	0.25	0.82	0.43
0.05	0.16	0.15	2.86	0.00	0.00	0.00	0.00
0.00	0.16	30.51	0.00	0.76	0.74	0.00	0.00
0.11	25.71	26.70	0.00	6.13	0.49	0.00	3.47
10.00	3.56	0.00	0.00	0.25	0.55	0.28	2.47
0.00	19.07	0.00	0.00	0.25	0.00	0.00	0.00
1.33	4.00	22.89	0.08	0.25	0.00	0.00	0.14
1.10	0.82	15.26	0.00	0.76	0.25	0.00	0.00
0.05	0.16	22.89	0.01	0.13	0.49	1.67	0.14
0.00	0.00	7.63	0.00	0.00	0.25	0.55	0.28
0.00	0.00	30.51	0.00	0.00	0.00	0.00	0.00
0.00	0.00	38.14	0.00	0.00	0.00	0.55	0.28
0.00	4.29	15.26	0.00	0.00	0.49	0.55	1.73
0.00	0.00	3.56	0.00	0.00	0.00	0.00	0.00
0.00	0.00	7.63	0.00	0.76	0.74	0.82	0.43
0.00	0.00	19.07	0.00	0.00	0.25	0.00	0.14
2.00	30.51	0.03	1.53	0.00	1.67	0.28	30.00

0.00	2.67	0.33	0.00	0.00	0.00	0.00	2.47
0.49	3.56	0.33	3.07	1.48	0.27	0.00	1.64
2.00	4.00	30.51	0.03	3.07	0.49	1.67	0.28
0.00	0.00	22.89	0.00	0.00	0.00	0.00	0.00
0.11	0.33	15.26	0.00	0.00	0.00	0.00	0.00
0.00	24.67	0.00	0.00	0.00	0.00	0.00	0.00
0.00	0.00	0.59	0.00	0.50	0.49	0.55	0.28
0.00	0.00	30.51	0.00	0.00	0.00	0.00	0.00
0.00	0.00	15.26	0.00	0.25	0.25	0.27	0.14
0.00	0.00	0.00	0.00	0.00	0.00	0.00	0.00
0.00	0.00	15.26	0.00	0.00	0.00	0.00	0.00
0.00	0.00	7.63	0.00	0.00	0.00	0.00	0.00
0.00	0.00	15.26	0.33	0.00	0.00	0.00	0.00
0.00	1.78	0.00	0.00	0.00	0.00	0.00	0.00
0.00	0.00	3.56	0.05	0.25	0.49	0.55	0.28
0.33	0.99	22.89	0.16	1.51	0.49	0.82	0.43
4.29	12.86	0.00	0.00	0.00	0.00	0.00	0.00
1.33	4.00	7.63	0.08	0.76	0.25	0.82	0.43
0.67	0.33	1.78	0.00	0.25	0.25	0.27	0.14
12.86	0.00	0.00	0.00	0.00	0.00	0.00	4.32
2.00	4.00	30.51	0.01	0.13	0.49	1.67	0.14
1.33	4.00	15.26	0.16	0.00	0.00	0.00	0.00
0.00	0.00	7.63	0.00	0.00	0.00	0.00	0.00
0.27	17.14	7.63	0.00	13.14	0.00	0.00	1.73
0.67	2.00	15.26	0.00	0.00	0.00	0.00	0.00
0.00	0.00	22.89	0.00	0.25	0.25	0.27	0.14
0.00	0.00	19.07	0.00	0.00	0.25	0.00	0.14
0.00	0.00	7.63	0.10	0.00	0.00	0.96	0.50
0.00	2.00	15.26	0.00	0.00	0.00	0.00	0.00
0.67	0.00	15.26	1.43	0.00	3.00	0.00	0.28
0.00	0.00	15.26	0.33	0.50	0.49	0.55	0.28
0.49	3.56	0.33	3.07	1.48	0.27	0.00	1.64
2.00	0.33	22.89	0.00	0.00	0.00	0.00	0.00
0.05	0.16	15.26	0.03	0.25	0.00	0.00	0.28
0.05	0.00	15.26	0.05	0.25	0.00	0.27	0.28
0.00	8.57	15.26	0.00	6.13	6.00	0.00	0.00
0.05	0.16	30.51	0.00	0.25	0.24	0.27	0.00
0.00	8.57	15.26	0.00	0.00	0.00	0.00	3.47
0.22	0.66	7.63	0.00	0.50	0.00	0.00	0.00
0.33	0.99	15.26	0.00	0.25	0.25	0.27	0.14
0.00	0.00	7.63	0.11	1.01	0.25	0.27	0.14
0.27	30.00	26.70	0.33	3.07	0.00	0.00	1.73
0.05	0.16	15.26	0.03	0.25	0.25	0.27	0.14
0.00	0.00	22.89	1.00	0.00	0.00	6.67	3.47
0.00	0.16	1.78	0.00	0.00	0.00	0.00	0.00
0.16	0.49	1.78	0.00	0.00	0.00	0.00	0.00
0.00	1.78	0.00	0.00	0.00	0.00	0.00	0.00
0.00	0.00	53.40	0.33	0.50	0.00	0.55	0.28
1.97	2.96	15.26	0.55	0.38	0.00	0.82	0.85
0.05	0.16	7.63	0.11	0.00	0.00	0.00	0.00
0.00	0.00	15.26	0.00	0.00	0.00	0.00	0.00
0.00	0.00	3.56	0.00	0.00	0.00	0.00	0.14
0.00	0.00	22.89	0.00	0.00	0.00	0.00	0.00

0.00	0.00	7.63	0.16	0.25	0.25	0.27	0.14
0.00	0.00	22.89	0.00	0.25	0.00	0.00	0.00
0.00	0.00	4.45	0.33	0.00	0.00	0.00	0.00
0.00	22.89	0.00	0.00	0.00	0.00	0.00	0.00
0.05	0.16	15.26	0.03	0.25	0.00	0.00	0.28
0.00	0.00	15.26	0.00	0.00	0.25	0.00	0.00
0.67	2.00	3.56	0.00	1.51	0.00	1.64	0.85
0.11	0.33	30.51	0.16	0.25	0.49	0.55	0.28
0.11	17.14	7.63	0.00	13.14	0.00	1.10	3.47
0.00	0.00	30.51	0.00	0.25	0.00	0.00	0.00
17.14	30.51	0.03	0.00	0.00	1.10	3.47	0.00
8.57	30.51	0.33	6.13	0.49	0.55	0.28	2.47
0.16	19.07	0.00	0.00	0.00	0.00	0.00	0.00
0.05	0.16	19.07	0.00	0.00	0.00	0.00	0.00
1.00	3.00	34.33	0.03	1.53	0.00	1.67	0.28
2.86	8.57	15.26	0.03	0.00	0.00	0.27	0.28
0.00	0.00	15.26	0.00	0.00	0.00	0.00	0.00
0.00	15.26	0.00	0.00	0.00	0.00	0.14	2.47
0.00	0.00	22.89	0.00	1.26	0.49	1.37	0.71
0.67	2.00	53.40	0.42	3.57	1.97	4.43	2.59
0.00	0.00	7.63	0.00	0.00	0.00	0.00	0.00
0.00	26.70	0.33	0.00	0.00	0.00	0.00	7.40
0.00	0.00	22.89	0.00	0.00	0.00	0.00	0.00
0.16	22.89	0.01	0.13	0.49	1.67	0.14	1.23
1.00	0.00	22.89	0.33	0.00	0.00	1.37	0.71
0.19	0.66	11.44	0.00	0.00	0.00	0.00	0.00
4.00	30.51	0.03	1.53	0.00	1.67	0.28	30.00
0.00	0.00	22.89	0.16	0.76	0.00	0.00	0.00
0.00	30.51	0.00	0.00	0.00	0.00	0.00	0.00
0.05	0.16	0.15	0.16	0.50	0.49	0.55	0.28
0.00	0.00	7.63	0.00	0.00	0.00	0.00	0.00
2.00	4.00	30.51	0.03	1.53	0.00	1.67	0.28
0.27	17.14	7.63	0.00	13.14	0.00	0.00	1.73
3.00	22.89	0.03	1.53	0.00	1.67	0.28	30.00
0.27	30.00	26.70	0.33	3.07	0.00	0.00	1.73
0.00	15.26	0.00	0.00	0.00	0.00	0.00	2.47
0.33	0.99	0.88	0.08	0.76	0.74	0.82	0.43
0.00	0.00	7.63	0.08	0.76	0.00	0.55	0.28
0.00	0.00	0.00	0.11	0.25	0.25	0.27	0.14
0.00	0.00	30.51	0.00	0.00	0.24	0.27	0.00
25.71	11.44	0.67	0.00	0.49	0.55	0.00	30.00
0.00	19.07	0.00	0.00	0.25	0.00	0.00	0.00
0.05	0.33	7.63	0.05	0.00	0.06	0.82	0.43
0.00	7.63	0.00	1.26	0.00	0.00	0.00	15.00
0.11	4.00	5.34	0.00	0.00	0.49	0.27	0.14
0.00	0.00	19.07	0.00	0.00	0.25	0.00	0.00
0.00	0.00	7.63	0.33	0.25	0.25	0.27	0.14
0.33	0.99	15.26	0.00	0.25	0.25	0.27	0.14
1.33	0.00	53.40	0.67	0.25	6.00	0.00	0.00
0.00	0.00	5.34	0.03	0.25	0.25	0.27	0.14
0.00	0.00	1.78	0.00	0.00	0.00	0.00	0.00
1.33	4.00	22.89	0.11	0.00	0.00	0.00	0.00
17.14	30.51	0.00	0.00	0.00	0.68	1.73	0.00

0.33	0.99	7.63	0.00	1.01	0.49	0.82	0.43
0.00	0.00	22.89	0.08	0.76	0.25	0.82	0.43
0.00	0.00	15.26	0.33	0.76	0.25	0.82	0.43
4.00	7.63	0.00	0.00	0.00	0.00	0.14	0.00
0.00	0.00	7.63	0.33	0.00	0.00	0.00	1.73
0.00	38.14	0.00	3.07	3.00	0.00	0.28	6.16
0.27	17.14	15.26	0.00	0.00	0.00	0.27	0.00
0.00	0.00	5.34	0.00	0.00	0.00	0.00	0.00
0.00	0.00	15.26	0.00	0.25	0.25	0.27	0.00
17.14	30.51	0.00	4.60	4.50	0.27	1.73	0.00
8.57	19.07	0.00	13.14	0.00	0.00	1.73	15.00
2.00	4.00	22.89	0.03	3.07	0.49	1.67	0.28
0.33	0.99	22.89	0.05	0.76	0.00	0.00	0.00
0.00	0.00	7.63	0.11	0.25	0.25	0.27	0.14
0.00	0.00	3.56	0.00	0.00	0.00	0.00	0.14
0.00	0.00	15.26	0.03	0.25	0.25	0.27	0.14
0.00	0.00	22.89	0.00	0.00	0.00	0.00	0.00
0.05	0.16	15.26	0.03	0.25	0.00	0.00	0.28
0.05	0.16	15.26	0.03	0.25	0.25	0.27	0.14
2.86	0.33	7.63	0.04	0.50	0.00	0.55	0.00
0.00	0.00	19.07	0.00	0.00	0.25	0.00	0.00
0.16	0.49	22.89	0.14	0.50	0.25	0.55	0.28
0.27	0.82	15.26	0.00	0.00	0.00	0.00	0.00
0.00	0.00	15.26	0.00	0.25	0.25	0.27	0.14
0.00	0.00	15.26	0.00	0.00	0.25	0.00	0.00
1.00	2.00	7.63	0.00	0.00	0.00	0.00	0.00
0.00	0.00	22.89	0.00	0.00	0.00	0.00	0.14
0.00	15.26	0.00	1.26	3.00	0.00	0.00	30.00
0.67	2.00	15.26	0.00	0.00	0.00	0.00	0.14
0.00	0.00	15.26	0.00	0.76	0.74	0.00	0.00
0.33	0.99	22.89	0.00	0.25	0.25	0.27	0.14
0.00	0.00	15.26	0.00	0.00	0.00	0.00	0.00
0.00	0.00	7.63	0.00	0.00	0.00	0.00	0.00
0.00	0.00	5.34	0.00	0.00	0.00	0.00	0.00
0.00	0.00	15.26	0.00	0.50	0.00	0.00	0.28
0.27	17.14	30.51	0.00	0.00	0.00	0.68	1.73
0.00	0.00	22.89	0.00	0.25	0.24	0.27	0.00
0.00	4.00	15.26	0.00	0.00	0.00	0.00	0.00
0.00	0.00	22.89	0.00	0.00	0.00	0.00	0.00
0.00	0.00	7.63	0.00	0.00	0.00	0.00	0.00
0.00	0.00	22.89	0.00	0.00	0.00	0.00	0.00
0.00	0.00	7.63	1.00	0.25	0.25	0.27	0.14
0.00	0.00	22.89	0.00	3.07	0.24	0.00	0.00
1.33	4.00	60.54	0.80	6.89	2.70	8.58	5.03
0.11	6.00	22.89	0.33	3.07	0.00	0.00	0.00
0.00	0.00	15.26	0.83	0.00	0.00	0.27	0.14
0.00	0.00	22.89	0.00	0.00	0.00	0.00	0.14
0.16	19.07	0.00	0.00	0.00	0.00	0.00	2.47
0.00	0.00	0.00	0.00	0.00	0.00	0.00	0.00
0.00	0.00	3.56	0.00	0.00	0.00	0.00	0.00
0.00	0.00	15.26	0.00	0.00	0.00	0.00	0.00
1.33	4.00	7.63	0.00	0.00	0.00	0.00	0.14
0.00	0.00	1.78	0.00	0.00	0.00	0.00	0.00

0.00	0.00	22.89	0.03	0.50	0.25	0.55	0.28
0.08	0.25	0.29	0.00	0.00	0.00	0.00	0.00
0.00	3.56	0.00	0.00	0.00	0.00	0.14	1.23
0.00	0.00	15.26	2.86	3.07	3.00	0.00	0.28
1.00	0.00	7.63	0.33	0.00	0.00	0.00	0.00
0.67	2.00	7.63	0.00	0.00	0.00	0.00	0.14
1.97	2.96	15.26	0.55	0.38	0.00	0.82	0.85
0.00	0.00	7.63	0.08	0.00	0.00	0.00	0.00
0.00	0.00	15.26	0.03	0.25	0.00	0.27	0.14
0.00	0.00	15.26	0.00	0.00	0.00	0.00	0.00
0.27	8.57	7.63	0.67	6.13	0.00	0.00	1.73
1.00	3.00	30.51	0.03	1.53	0.00	1.67	0.28
0.11	0.16	22.89	0.00	0.00	0.00	0.00	0.00
0.00	0.00	22.89	0.00	0.00	0.25	0.00	0.00
6.57	0.00	22.89	0.03	0.25	0.25	0.27	0.14
0.22	0.00	0.00	0.00	0.00	0.00	0.00	0.00
1.00	0.00	7.63	0.33	0.00	0.00	0.00	0.00
0.05	0.16	15.26	0.03	0.25	0.00	0.00	0.28
0.00	0.00	19.07	0.00	0.00	0.25	0.00	0.00
0.00	0.00	15.26	0.00	0.25	0.25	0.27	0.14
0.00	19.07	0.00	0.00	0.25	0.00	0.00	0.00
0.27	17.14	30.51	0.00	3.07	3.00	0.27	0.00
0.00	0.00	22.89	0.05	0.00	0.00	0.27	0.28
0.00	0.66	7.63	0.14	0.00	0.25	0.00	0.57
0.00	0.00	22.89	0.00	0.00	0.00	0.00	0.00
0.00	0.00	7.12	0.05	3.07	0.00	0.55	0.28
0.67	2.00	30.51	0.03	1.53	0.00	1.67	0.28
1.33	0.00	3.56	0.03	0.76	0.25	0.82	0.43
0.00	0.00	15.26	0.33	0.25	0.25	0.27	0.14
0.00	0.00	7.63	0.00	0.00	0.00	0.27	0.14
0.00	0.00	22.89	0.05	0.00	0.00	0.00	0.00

pitza	shirekamcl	shireporcha	shircacao	mastecheg	mastemar	masteporcl	panir
7.40	98.57	3.78	4.11	0.49	98.57	15.33	30.00
0.00	65.71	0.00	0.00	2.00	98.57	15.33	15.00
2.47	32.86	0.00	16.67	17.14	131.43	0.00	30.00
0.00	82.14	1.26	0.00	2.00	98.57	15.33	30.00
7.40	15.33	0.00	0.00	0.00	0.00	164.29	30.00
0.00	230.00	0.00	0.00	12.86	230.00	0.00	0.00
0.00	98.57	1.26	35.71	1.00	98.57	7.67	30.00
0.00	230.00	0.00	0.00	0.00	230.00	230.00	30.00
98.57	1.26	0.00	2.00	98.57	15.33	45.00	1.00
0.00	230.00	98.57	0.00	0.00	0.00	230.00	0.00
65.71	164.29	0.00	8.57	0.00	98.57	12.86	30.00
15.00	460.00	15.33	2.05	0.00	230.00	15.33	30.00
98.57	32.86	8.33	21.43	32.86	32.86	45.00	0.00
2.47	3.78	3.78	0.00	0.00	3.78	4.41	30.00
0.00	0.00	0.00	0.00	0.00	230.00	0.00	30.00
0.00	131.43	1.26	0.00	1.00	164.29	7.67	30.00
30.00	2.52	115.00	16.67	17.14	15.33	147.86	4.29
0.00	230.00	0.00	0.00	0.00	98.57	0.00	30.00
7.40	65.71	0.00	0.00	4.29	98.57	0.00	4.29
15.00	3.15	49.29	50.00	12.86	15.33	115.00	4.29
0.00	230.00	0.00	71.43	0.00	230.00	7.67	30.00
30.00	0.00	65.71	16.67	0.00	15.33	164.29	45.00
3.08	0.00	0.00	0.00	2.00	0.00	98.57	30.00
2.47	0.00	3.15	0.00	0.00	0.00	65.71	30.00
0.00	65.71	0.00	0.00	2.00	98.57	15.33	15.00
0.00	0.00	197.14	0.00	3.00	11.50	65.71	12.86
0.00	230.00	0.00	8.33	0.00	230.00	0.00	30.00
7.40	0.00	0.00	0.00	0.00	230.00	0.00	30.00
30.00	3.15	131.43	50.00	17.14	15.33	115.00	4.29
4.93	15.33	65.71	0.00	8.57	15.33	131.43	8.57
0.00	0.00	147.86	0.00	4.29	7.67	98.57	21.43
45.00	82.14	82.14	0.00	4.29	19.17	65.71	30.00
0.00	65.71	1.26	0.00	1.00	164.29	7.67	30.00
4.93	0.00	0.00	2.05	0.00	0.00	0.00	30.00
0.00	0.00	8.33	2.14	164.29	0.00	30.00	0.50
49.29	1.26	0.00	0.00	131.43	0.00	30.00	0.50
1.23	131.43	0.00	0.00	0.00	23.00	0.00	30.00
7.40	131.43	0.00	0.00	0.33	230.00	3.78	30.00
0.00	65.71	0.00	8.33	4.29	32.86	0.00	30.00
1.64	11.34	11.34	8.33	0.00	32.86	32.86	17.14
0.00	0.00	7.67	0.00	0.00	0.00	115.00	30.00
2.47	82.14	82.14	41.67	21.43	0.00	65.71	4.29
30.00	0.00	164.29	16.67	0.00	7.67	197.14	8.57
4.93	15.33	15.33	0.00	0.00	0.00	98.57	30.00
4.93	0.00	0.00	0.00	4.29	230.00	0.00	30.00
4.93	0.00	0.00	0.00	0.33	7.67	7.67	30.00
15.00	0.00	0.00	0.00	0.00	0.00	98.57	30.00
7.67	147.86	8.33	4.29	7.67	65.71	8.57	37.50
1.23	0.00	0.00	0.00	0.00	230.00	0.00	30.00
38.33	191.67	0.68	1.23	65.71	98.57	4.29	4.29
7.40	460.00	0.00	71.43	0.00	230.00	0.00	30.00
0.00	82.14	1.26	0.00	1.00	164.29	7.67	30.00

7.40	98.57	3.78	4.11	0.00	98.57	0.00	30.00
0.00	19.17	0.00	0.00	0.00	32.86	11.50	25.71
3.70	0.00	0.00	0.00	0.00	32.86	0.00	30.00
82.14	0.00	0.00	0.00	131.43	0.00	15.00	0.50
0.00	115.00	125.00	0.00	0.00	0.00	30.00	0.00
0.00	460.00	0.00	0.00	0.00	360.00	0.00	30.00
82.14	1.26	0.00	2.00	98.57	15.33	30.00	1.00
0.00	23.00	0.00	0.00	0.00	0.00	0.00	0.00
20.00	32.86	0.00	35.71	15.00	7.67	0.00	30.00
65.71	164.29	41.67	0.00	7.67	164.29	45.00	30.00
82.14	1.26	8.33	0.00	115.00	0.00	30.00	1.00
1.23	82.14	0.00	0.00	0.00	131.43	0.00	15.00
0.00	65.71	0.00	0.00	0.00	230.00	0.00	30.00
1.23	197.14	1.26	0.00	0.16	115.00	0.63	30.00
1.23	32.86	0.00	0.00	0.00	32.86	32.86	30.00
98.57	32.86	8.33	21.43	32.86	32.86	45.00	0.00
131.43	1.89	0.00	0.16	115.00	1.26	45.00	0.00
0.00	3.78	3.78	4.11	0.49	3.78	3.78	17.14
1.64	11.34	11.34	8.33	0.00	32.86	32.86	17.14
30.00	0.00	131.43	16.67	0.00	7.67	131.43	30.00
46.00	1.26	5.48	0.00	0.00	65.71	2.00	0.16
180.71	32.86	8.33	4.29	15.33	115.00	30.00	4.29
0.00	0.00	0.00	0.00	0.00	7.67	7.67	30.00
7.40	98.57	0.00	0.00	1.00	115.00	7.67	15.00
49.29	131.43	0.00	15.00	3.83	32.86	4.29	45.00
0.00	32.86	0.00	0.00	30.00	230.00	0.00	30.00
12.33	3.78	0.00	16.67	0.00	98.57	0.00	30.00
4.93	32.86	32.86	4.11	0.49	3.78	98.57	30.00
82.14	1.26	0.00	0.00	131.43	0.00	30.00	0.00
2.47	115.00	0.63	0.00	0.16	0.00	0.63	30.00
164.29	1.89	8.33	0.08	131.43	0.63	30.00	0.08
6.16	82.14	82.14	41.67	4.29	19.17	65.71	30.00
30.00	0.00	65.71	16.67	0.00	15.33	115.00	30.00
321.43	0.00	0.00	0.00	0.00	32.86	0.00	21.43
98.57	0.00	0.00	2.00	98.57	15.33	15.00	0.50
30.00	98.57	0.00	4.11	0.00	230.00	0.00	30.00
7.40	98.57	0.00	0.00	0.00	230.00	0.00	4.29
88.57	0.00	0.00	0.00	0.00	98.57	0.00	30.00
1.23	131.43	0.00	2.05	8.57	65.71	0.00	30.00
2.47	15.33	197.14	25.00	12.86	7.67	131.43	8.57
7.67	65.71	33.33	8.57	15.33	65.71	30.00	45.00
98.57	0.00	0.00	2.00	98.57	15.33	15.00	1.00
3.70	65.71	4.41	1.37	0.00	3.15	3.15	8.57
20.00	7.67	230.00	2.74	0.82	131.43	65.71	30.00
1.23	197.14	3.83	8.33	1.00	15.33	16.43	30.00
0.00	32.86	98.57	0.00	4.29	32.86	65.71	30.00
0.00	3.83	0.00	0.00	0.00	0.00	7.67	30.00
0.00	32.86	0.00	8.33	0.00	0.00	98.57	30.00
30.00	0.00	65.71	16.67	0.00	15.33	164.29	30.00
0.00	230.00	0.00	0.00	0.00	230.00	0.00	30.00
3.70	32.86	32.86	0.00	4.29	32.86	0.00	0.00
0.00	98.57	0.00	0.00	2.00	98.57	15.33	15.00
2.52	98.57	16.67	19.29	15.33	147.86	4.29	21.43

0.00	131.43	16.67	0.00	82.14	98.57	30.00	0.00
23.00	23.00	1.37	2.00	15.33	0.00	30.00	4.29
7.50	65.71	32.86	8.33	4.29	15.33	115.00	30.00
0.00	230.00	0.00	0.00	0.00	230.00	0.00	30.00
6.16	98.57	0.00	0.00	0.00	131.43	98.57	30.00
65.71	0.00	8.33	8.57	98.57	0.00	60.00	0.00
0.00	30.67	0.00	0.00	0.00	32.86	0.00	30.00
2.47	32.86	0.00	0.00	0.00	230.00	0.00	8.57
15.00	0.00	0.00	0.00	0.16	32.86	0.00	30.00
0.00	0.00	32.86	62.50	0.16	0.00	230.00	30.00
45.00	32.86	0.00	0.00	0.00	65.71	0.00	30.00
0.00	49.29	0.00	0.00	0.00	230.00	0.00	30.00
0.00	230.00	0.00	0.00	4.29	230.00	0.00	30.00
345.00	0.00	0.00	0.00	0.00	0.00	30.00	0.00
0.00	0.00	0.00	0.00	2.00	15.33	15.33	30.00
3.70	98.57	3.78	4.11	4.29	98.57	3.78	21.43
15.00	0.00	0.00	0.00	0.00	0.00	0.00	0.00
4.93	32.86	2.52	2.74	0.33	98.57	2.52	12.86
7.40	230.00	0.00	0.00	0.00	98.57	98.57	30.00
0.00	0.00	0.00	0.00	0.00	0.00	30.00	0.00
1.23	197.14	7.67	8.33	4.29	15.33	164.29	30.00
0.00	32.86	0.00	0.00	2.00	230.00	0.00	30.00
0.00	98.57	0.00	0.00	0.00	98.57	131.43	30.00
0.00	0.00	147.86	0.00	4.29	7.67	98.57	21.43
8.63	0.00	0.00	0.00	0.00	32.86	7.67	30.00
7.40	230.00	0.00	0.00	1.00	230.00	0.00	30.00
0.00	65.71	0.00	0.00	2.00	98.57	15.33	15.00
1.23	115.00	0.00	0.00	0.12	82.14	0.00	30.00
30.00	131.43	0.00	8.33	1.00	98.57	0.00	30.00
30.00	0.00	164.29	16.67	1.00	15.33	65.71	8.57
15.00	230.00	0.00	0.00	0.00	230.00	0.00	30.00
23.00	23.00	1.37	2.00	15.33	0.00	30.00	4.29
7.50	230.00	1.26	1.37	1.00	98.57	65.71	30.00
1.44	98.57	32.86	8.33	21.43	32.86	32.86	45.00
0.62	230.00	7.67	0.00	0.16	230.00	98.57	30.00
45.00	65.71	164.29	16.67	0.00	7.67	164.29	45.00
1.23	197.14	3.15	8.33	0.08	131.43	0.63	30.00
45.00	65.71	164.29	41.67	0.00	7.67	164.29	45.00
4.93	30.67	0.00	0.00	0.00	98.57	0.00	30.00
1.64	131.43	7.67	8.33	0.00	131.43	7.67	30.00
15.00	230.00	0.00	0.00	0.00	0.00	0.00	30.00
0.00	7.67	147.86	8.33	4.29	7.67	65.71	8.57
3.70	345.00	0.00	0.00	0.16	230.00	7.67	30.00
0.00	65.71	0.00	0.00	0.00	0.00	164.29	30.00
4.93	0.00	0.00	0.00	0.00	98.57	0.00	30.00
2.47	32.86	0.00	0.00	0.08	115.00	0.00	30.00
32.86	0.00	8.33	4.29	32.86	0.00	30.00	0.00
2.47	98.57	32.86	0.00	0.00	230.00	32.86	30.00
20.00	131.43	12.60	1.37	0.00	3.78	345.00	12.86
7.40	0.00	0.00	0.00	0.00	131.43	0.00	17.14
0.00	230.00	0.00	0.00	0.00	230.00	7.67	30.00
1.23	82.14	1.26	0.00	0.00	131.43	0.00	15.00
2.47	82.14	1.26	8.33	0.00	131.43	0.00	45.00

7.40	0.00	23.00	0.00	0.00	7.67	98.57	60.00
0.00	0.00	0.00	0.00	0.00	0.00	230.00	30.00
0.00	0.00	164.29	0.00	0.00	65.71	65.71	30.00
82.14	1.26	0.00	0.00	131.43	0.00	30.00	0.00
1.44	98.57	32.86	8.33	21.43	32.86	32.86	45.00
1.23	131.43	0.63	35.71	1.00	98.57	7.67	30.00
4.93	0.00	0.00	0.00	0.00	0.00	0.00	30.00
4.93	7.67	7.67	1.37	0.16	32.86	3.15	30.00
0.00	0.00	197.14	0.00	3.00	11.50	65.71	10.71
7.40	230.00	0.00	35.71	30.00	230.00	0.00	30.00
17.25	172.50	0.00	30.00	11.50	32.86	4.29	45.00
0.00	98.57	16.67	8.57	65.71	0.00	30.00	8.57
65.71	1.26	0.00	0.00	115.00	0.00	30.00	0.00
2.47	82.14	1.26	8.33	0.00	115.00	0.00	30.00
30.00	2.52	98.57	16.67	19.29	15.33	147.86	4.29
9.86	460.00	160.00	0.00	0.00	360.00	0.00	30.00
0.00	230.00	0.00	0.00	0.00	230.00	0.00	30.00
98.57	1.89	0.00	0.16	98.57	7.67	30.00	8.57
30.00	230.00	0.00	0.00	4.29	0.00	98.57	30.00
16.23	241.50	0.00	1.71	4.29	300.52	131.43	98.57
1.23	460.00	15.33	0.00	0.08	230.00	65.71	60.00
0.00	65.71	0.00	0.00	82.14	0.00	30.00	0.00
0.00	32.86	0.00	0.00	17.14	131.43	0.00	30.00
197.14	3.83	8.33	1.00	15.33	16.43	30.00	1.00
0.00	65.71	0.00	35.71	0.00	65.71	0.00	17.14
1.23	7.67	0.00	0.00	1.00	15.33	0.00	30.00
3.15	131.43	50.00	17.14	15.33	115.00	4.29	21.43
12.33	23.00	15.33	4.11	0.49	0.00	65.71	30.00
3.83	0.00	0.00	0.00	65.71	7.67	30.00	0.00
4.93	98.57	23.00	2.74	0.00	98.57	15.33	30.00
0.00	460.00	0.00	0.00	0.00	65.71	0.00	17.14
30.00	3.15	131.43	50.00	17.14	15.33	115.00	4.29
0.00	0.00	147.86	0.00	8.57	23.00	98.57	21.43
3.15	131.43	50.00	17.14	15.33	115.00	4.29	21.43
6.16	7.67	147.86	8.33	4.29	7.67	65.71	8.57
65.71	3.15	0.00	0.16	98.57	15.33	30.00	0.00
7.40	98.57	3.78	4.11	0.49	3.78	3.78	30.00
0.00	0.00	0.00	0.00	0.00	3.78	3.78	30.00
4.93	32.86	0.00	35.71	0.00	98.57	0.00	30.00
1.23	164.29	3.15	0.00	0.16	230.00	0.63	30.00
0.00	65.71	0.00	0.00	23.00	164.29	30.00	8.57
131.43	0.63	0.00	2.00	98.57	15.33	45.00	1.00
2.47	2.52	7.67	1.37	0.25	23.00	23.00	0.25
0.00	65.71	41.67	8.57	32.86	32.86	8.57	30.00
9.86	7.67	164.29	41.67	4.29	7.67	131.43	45.00
0.00	131.43	1.26	0.00	1.00	164.29	7.67	30.00
15.00	32.86	0.00	0.00	0.00	32.86	0.00	12.86
1.64	131.43	7.67	8.33	0.00	131.43	7.67	30.00
15.00	0.00	32.86	8.33	1.00	7.67	197.14	8.57
7.40	38.33	23.00	0.00	0.00	30.67	23.00	30.00
0.00	7.67	0.00	0.00	6.00	7.67	0.00	30.00
7.40	32.86	32.86	4.11	0.49	230.00	230.00	30.00
17.25	147.86	0.00	30.00	3.83	32.86	4.29	45.00

15.00	98.57	0.00	0.00	0.00	0.00	32.86	12.86
3.70	0.00	65.71	0.00	0.00	0.00	0.00	30.00
15.00	131.43	32.86	8.33	1.00	230.00	32.86	30.00
230.00	0.00	0.00	0.00	7.67	7.67	30.00	0.00
0.00	0.00	0.00	0.00	0.00	65.71	65.71	2.00
0.00	164.29	41.67	2.00	38.33	65.71	12.86	30.00
0.00	98.57	98.57	0.00	8.57	0.00	98.57	12.86
0.00	15.33	0.00	0.00	0.00	65.71	0.00	30.00
7.40	15.33	0.00	0.00	0.00	65.71	65.71	30.00
49.29	131.43	0.00	19.29	0.00	32.86	4.29	45.00
0.00	98.57	50.00	0.00	7.67	131.43	30.00	10.71
30.00	3.15	131.43	50.00	17.14	15.33	115.00	4.29
30.00	98.57	0.00	0.00	4.29	230.00	0.00	30.00
6.16	82.14	0.00	0.00	0.00	98.57	0.00	30.00
1.23	65.71	0.00	8.33	1.00	131.43	0.00	15.00
0.00	164.29	15.33	0.00	17.14	98.57	0.00	30.00
0.00	23.00	0.00	0.00	0.00	34.50	0.00	30.00
1.44	98.57	32.86	8.33	21.43	32.86	32.86	45.00
3.70	345.00	0.00	0.00	0.16	230.00	7.67	30.00
1.23	11.50	11.50	1.03	0.12	0.00	115.00	30.00
0.00	131.43	1.26	0.00	1.00	164.29	7.67	30.00
7.40	15.33	0.00	0.00	0.33	98.57	65.71	12.86
3.29	230.00	1.26	16.67	0.00	115.00	0.63	30.00
6.16	65.71	0.00	0.00	1.00	32.86	230.00	30.00
1.23	131.43	0.63	35.71	1.00	98.57	7.67	30.00
0.00	0.00	7.67	0.00	0.00	32.86	0.00	30.00
0.00	98.57	1.89	0.00	0.16	98.57	1.26	30.00
0.00	131.43	16.67	4.29	32.86	164.29	8.57	30.00
0.00	230.00	0.00	0.00	0.00	7.67	0.00	30.00
1.23	98.57	7.67	0.68	8.57	65.71	0.00	30.00
7.40	230.00	0.00	0.00	0.00	230.00	3.78	30.00
2.47	65.71	3.15	0.00	0.16	98.57	15.33	30.00
0.00	0.00	0.00	0.00	0.00	131.43	0.00	30.00
0.00	49.29	1.26	0.00	0.00	131.43	0.00	30.00
2.47	15.33	0.00	0.00	0.16	230.00	0.00	30.00
0.00	49.29	131.43	0.00	19.29	0.00	32.86	4.29
1.23	230.00	3.15	0.00	0.08	131.43	0.63	45.00
2.47	15.33	0.00	0.00	30.00	0.00	0.00	30.00
0.00	32.86	0.00	0.00	0.00	131.43	0.00	30.00
0.00	164.29	0.00	0.00	4.29	65.71	1.89	30.00
2.47	230.00	32.86	0.00	0.00	230.00	0.00	30.00
0.00	65.71	0.00	0.00	4.29	32.86	0.00	30.00
3.70	230.00	0.63	0.00	0.16	32.86	0.63	30.00
32.47	483.00	0.00	3.42	8.57	301.05	164.29	108.57
4.93	15.33	131.43	0.00	4.29	7.67	131.43	8.57
1.23	0.00	0.00	0.00	0.00	0.00	82.14	12.86
0.00	131.43	1.89	0.00	0.16	115.00	1.26	45.00
82.14	1.26	8.33	0.00	115.00	0.00	30.00	1.00
0.00	0.00	0.00	0.00	0.00	32.86	0.00	30.00
2.47	230.00	0.00	0.00	0.21	230.00	0.00	30.00
2.47	65.71	3.15	0.00	0.16	98.57	7.67	30.00
0.00	230.00	0.00	0.00	0.00	7.67	7.67	30.00
15.00	230.00	0.00	0.00	0.00	98.57	0.00	12.86

2.47	0.00	0.00	0.00	0.25	32.86	32.86	30.00
2.47	600.00	0.00	0.00	0.00	230.00	0.00	30.00
49.29	1.26	0.00	0.00	131.43	0.00	30.00	0.50
6.16	0.00	32.86	8.33	2.00	38.33	65.71	12.86
0.00	0.00	32.86	89.29	0.00	460.00	0.00	30.00
0.00	230.00	0.00	0.00	0.00	7.67	7.67	30.00
20.00	131.43	12.60	1.37	0.00	3.78	345.00	12.86
7.40	32.86	98.57	16.67	0.00	164.29	2.52	60.00
2.47	131.43	0.00	0.00	0.00	164.29	0.00	30.00
2.47	32.86	0.00	16.67	17.14	131.43	0.00	30.00
6.16	7.67	147.86	8.33	4.29	7.67	65.71	8.57
30.00	2.52	98.57	16.67	12.86	15.33	115.00	4.29
0.00	115.00	1.26	0.00	0.00	115.00	0.00	45.00
0.00	197.14	1.26	0.00	1.00	164.29	7.67	30.00
2.47	230.00	0.00	0.00	0.00	230.00	0.00	30.00
0.00	49.29	49.29	0.00	0.00	164.29	23.00	30.00
3.08	46.00	49.29	0.00	0.00	0.00	230.00	30.00
1.44	98.57	32.86	8.33	21.43	32.86	32.86	45.00
0.00	82.14	1.26	0.00	1.00	164.29	7.67	30.00
1.23	98.57	0.00	0.00	4.29	65.71	15.33	30.00
98.57	1.26	0.00	2.00	98.57	15.33	45.00	1.00
0.00	32.86	32.86	0.00	8.57	0.00	32.86	4.29
7.40	230.00	0.00	2.74	4.00	98.57	0.00	4.29
0.00	32.86	0.00	4.11	30.00	131.43	0.00	0.00
0.00	32.86	0.00	0.00	0.00	131.43	0.00	30.00
4.93	230.00	32.86	0.00	3.00	230.00	32.86	30.00
30.00	2.52	98.57	8.33	19.29	23.00	147.86	4.29
0.00	1.26	0.00	0.00	0.00	32.86	230.00	30.00
0.00	15.33	0.00	0.00	0.00	15.33	0.00	30.00
0.00	0.00	98.57	4.11	0.00	0.00	230.00	30.00
0.00	0.00	0.00	0.00	0.00	230.00	0.00	4.29

panirekhar	doogh	khame	bastanison	bastanighe	kare	margarin	kashk
0.49	5.78	0.25	1.40	5.00	0.41	0.41	0.16
0.50	12.38	0.00	2.83	5.00	0.83	0.00	0.71
0.00	12.38	0.00	12.14	2.50	0.00	0.00	10.00
1.00	55.71	0.00	2.83	5.00	0.83	0.00	0.71
0.00	0.00	0.50	2.83	1.23	0.00	0.00	0.00
0.00	86.66	0.00	0.00	5.00	1.67	0.00	1.43
1.00	30.95	0.00	2.83	2.50	1.67	0.00	0.71
0.00	37.14	0.00	2.83	0.00	0.00	0.00	0.00
37.14	0.00	2.83	5.00	0.83	0.00	0.71	14.29
0.00	5.78	0.00	0.00	0.00	0.00	0.00	0.00
8.67	6.43	5.67	2.50	17.86	1.67	0.00	3.33
1.00	86.66	0.50	12.14	10.71	0.83	0.00	0.27
8.66	1.00	2.83	7.50	0.53	0.07	1.00	7.14
0.16	5.78	0.04	0.70	1.23	0.14	0.07	15.00
0.00	12.38	0.00	0.00	0.00	0.00	0.00	0.00
1.00	61.90	0.00	5.67	2.50	1.67	0.00	0.71
21.43	0.47	0.75	5.67	2.50	14.29	14.29	0.05
0.00	5.78	0.00	0.00	10.71	0.00	0.00	0.00
0.00	12.38	1.00	24.29	10.71	3.57	0.83	0.33
21.43	0.47	3.00	5.67	2.50	17.86	10.71	0.05
0.00	12.38	0.00	0.00	0.00	0.00	0.00	0.00
21.43	5.78	4.29	14.17	2.50	16.07	1.67	0.14
2.00	12.38	0.10	5.67	5.00	1.67	0.00	0.67
0.00	86.66	0.00	2.83	2.50	0.00	0.00	0.00
0.50	12.38	0.00	2.83	5.00	0.83	0.00	0.71
15.00	0.00	7.50	14.17	12.50	17.86	0.83	0.00
2.00	24.76	0.00	0.00	2.50	10.71	0.00	0.33
17.14	86.66	0.00	1.40	0.00	0.00	0.00	0.16
21.43	0.47	3.00	5.67	2.50	17.86	10.71	0.05
60.00	2.89	4.29	17.00	5.00	17.86	0.83	0.00
30.00	0.00	10.71	14.17	12.50	17.86	0.83	0.33
12.86	10.11	6.43	5.67	2.50	14.29	2.08	0.00
1.00	61.90	0.00	2.83	2.50	1.67	0.00	0.71
0.00	37.14	0.00	12.14	0.00	0.00	0.00	0.67
12.38	0.04	2.83	2.50	0.83	0.07	0.33	28.57
12.38	0.00	2.83	2.50	3.57	0.00	0.00	32.14
0.00	43.33	0.12	0.47	0.41	0.00	0.00	0.27
0.49	5.78	0.25	0.00	1.23	0.27	0.34	0.03
0.00	12.38	0.00	0.00	0.00	0.00	0.00	10.00
0.00	2.89	1.50	0.47	2.50	2.29	0.00	0.00
30.00	2.89	15.00	0.00	2.50	0.83	0.00	0.00
30.00	5.78	4.29	5.67	2.50	3.57	2.08	0.00
45.00	61.90	4.29	14.17	5.00	17.86	1.67	0.00
0.00	24.76	0.50	2.83	0.00	0.00	0.00	0.33
17.14	0.00	0.00	2.83	2.50	0.00	0.00	1.43
0.00	2.89	0.00	2.83	2.50	0.00	0.00	0.67
0.00	37.14	0.50	12.14	10.71	0.00	0.00	0.00
0.00	7.50	5.67	2.50	3.57	0.00	0.00	1.67
0.00	0.00	0.00	0.00	0.00	0.00	0.00	1.43
37.11	0.00	24.29	0.00	8.00	0.00	1.23	7.14
0.00	86.66	0.00	12.14	10.71	0.00	0.00	0.67
1.00	55.71	0.00	2.83	5.00	0.83	0.00	0.71

0.00	37.14	0.50	1.40	1.23	0.00	0.00	0.67
0.00	6.19	0.00	0.00	0.00	0.00	0.00	0.00
0.00	12.38	0.00	0.00	1.23	0.00	0.00	0.00
12.38	0.00	2.83	2.50	3.57	0.00	0.00	21.43
8.67	0.25	0.00	0.00	0.00	0.00	0.00	14.29
0.00	0.00	0.00	0.00	1.23	3.57	0.00	1.43
55.71	0.00	2.83	5.00	0.83	0.00	0.71	14.29
30.00	24.76	0.00	0.00	2.50	1.25	0.00	10.00
1.00	0.00	1.00	2.83	5.00	1.14	0.00	0.50
5.78	4.29	14.17	2.50	7.14	1.67	0.00	3.33
2.89	0.00	2.83	2.50	3.57	0.14	0.00	32.14
0.50	12.38	0.00	2.83	2.50	3.57	0.00	0.00
0.00	173.32	0.00	12.14	10.71	0.00	0.00	2.86
0.08	12.38	0.04	12.14	2.50	0.83	0.14	1.67
12.86	5.78	1.00	0.93	0.82	0.21	0.00	0.67
8.66	1.00	2.83	7.50	0.53	0.07	1.00	7.14
2.89	0.00	5.67	5.00	10.71	0.00	1.43	14.29
0.33	37.14	0.16	0.93	5.00	0.34	0.00	0.16
0.00	2.89	1.50	0.47	2.50	2.29	0.00	0.00
10.71	8.67	6.43	5.67	5.00	14.29	1.67	0.00
14.43	0.50	5.67	10.00	18.67	0.00	0.29	28.57
0.47	0.50	2.83	2.50	3.57	3.57	0.03	35.71
0.00	0.00	0.00	0.00	0.41	3.57	0.00	0.00
1.00	30.95	2.14	2.83	2.50	0.00	0.00	0.33
0.00	10.71	5.67	2.50	7.14	4.17	0.33	1.67
0.00	12.38	0.00	0.00	0.00	0.00	0.00	10.00
0.00	12.38	0.50	24.29	0.00	7.14	0.00	0.33
0.49	1.42	6.43	5.67	5.00	3.57	0.21	0.67
37.14	0.00	0.00	0.00	1.67	0.00	1.43	0.00
0.08	12.38	0.04	12.14	2.50	0.83	0.14	1.67
30.95	0.00	12.14	5.00	1.67	0.14	1.67	17.86
12.86	10.11	6.43	11.33	5.00	14.29	2.08	0.00
10.71	5.78	4.29	14.17	2.50	16.07	1.67	0.14
0.16	0.00	0.16	12.14	10.71	0.00	0.00	0.67
61.90	0.00	2.83	5.00	0.83	0.00	0.71	14.29
0.00	1.42	0.00	12.14	10.71	0.00	0.00	0.00
0.49	37.14	0.25	1.40	1.23	0.00	0.00	0.03
0.00	24.76	0.00	0.00	32.14	0.41	0.00	0.00
4.29	12.38	0.50	6.07	2.50	0.00	0.00	10.00
30.00	2.89	11.79	5.67	2.50	7.14	0.83	0.00
2.89	4.29	5.67	12.50	14.29	0.83	0.00	3.33
61.90	0.00	2.83	5.00	0.83	0.00	0.71	14.29
0.00	37.14	0.50	1.63	1.44	0.83	0.00	2.86
0.00	24.74	0.41	0.93	0.62	1.32	0.00	1.03
1.00	0.47	0.50	2.83	2.50	3.57	0.07	0.03
0.49	12.38	0.00	12.14	7.50	0.83	0.83	1.43
0.00	12.38	0.00	0.00	0.00	0.00	0.00	0.00
0.00	12.38	1.50	36.43	0.00	7.14	0.00	0.00
10.71	2.89	10.71	14.17	2.50	16.07	1.67	0.14
0.00	49.52	0.00	2.83	0.00	0.00	0.00	10.00
0.00	12.38	0.12	12.14	0.00	3.57	0.00	0.00
0.50	61.90	0.00	2.83	5.00	0.83	0.00	0.71
0.47	0.75	5.67	2.50	14.29	14.29	0.05	0.14

86.66	0.50	0.00	0.00	0.00	0.00	0.00	14.29
61.86	0.00	2.83	2.50	0.53	0.00	2.50	21.43
4.29	0.47	0.50	2.83	2.50	3.57	3.57	0.03
0.00	0.00	0.00	0.00	0.00	25.00	0.83	1.43
0.00	24.76	0.00	2.83	0.00	0.00	0.00	0.67
24.76	0.00	0.00	0.00	0.00	0.00	20.00	0.00
12.86	0.00	0.04	0.47	0.00	0.14	0.00	5.71
0.00	0.00	0.00	0.47	1.44	3.33	0.00	0.67
0.00	0.00	0.00	0.70	0.41	0.00	0.00	0.33
0.00	0.00	0.00	0.00	10.71	0.00	0.00	1.00
0.00	12.38	0.00	0.00	0.00	7.14	0.00	0.00
0.00	12.38	0.00	0.00	0.41	7.14	0.00	0.33
0.00	86.66	0.00	12.14	10.71	0.00	0.00	1.43
0.00	0.00	0.00	0.00	0.00	0.00	0.00	4.17
0.00	86.66	0.00	5.67	0.00	0.00	0.00	0.67
0.49	74.28	0.25	1.40	1.23	0.41	0.41	0.16
30.00	0.00	0.00	0.00	0.62	3.57	0.00	0.00
0.33	5.78	1.00	0.93	10.71	1.67	0.00	0.08
0.00	2.89	1.00	0.00	21.43	0.00	0.00	0.33
0.00	0.00	0.00	0.00	3.57	0.00	0.00	3.33
1.00	0.47	0.50	2.83	2.50	3.57	1.79	0.03
0.00	5.78	0.25	5.67	5.00	0.00	0.00	0.00
2.00	86.66	2.14	12.14	5.00	1.67	0.00	0.67
30.00	0.00	10.71	14.17	12.50	17.86	0.83	0.33
0.00	0.00	0.00	2.83	2.50	0.83	0.00	0.67
0.00	37.14	0.00	0.23	5.00	0.00	0.00	1.43
0.50	61.90	0.00	2.83	5.00	0.83	0.00	0.71
10.71	30.95	2.14	0.23	5.00	0.31	0.00	0.67
0.00	37.14	0.00	2.83	0.00	0.00	0.00	0.00
45.00	5.78	10.71	14.17	5.00	17.86	1.67	0.00
0.00	173.32	0.00	2.83	0.00	0.00	0.00	0.33
61.86	0.00	2.83	2.50	0.53	0.00	2.50	21.43
0.00	5.77	2.14	1.40	19.93	0.09	0.00	0.08
0.00	8.66	1.00	2.83	7.50	0.53	0.07	1.00
0.08	61.90	0.04	1.42	2.50	0.00	0.00	0.05
30.00	5.78	4.29	14.17	2.50	7.14	1.67	0.00
0.08	30.95	0.00	24.29	5.00	1.67	0.14	1.67
30.00	5.78	4.29	14.17	2.50	7.14	1.67	0.00
0.00	2.89	0.50	12.14	10.71	0.00	0.00	0.67
0.16	37.11	1.00	0.93	5.17	0.27	0.00	1.00
2.14	12.38	0.00	12.14	0.00	3.57	0.00	1.43
37.50	0.00	12.86	14.17	5.00	14.29	0.00	0.00
1.00	49.52	0.50	2.83	2.50	0.83	0.00	0.67
0.00	173.32	3.00	0.00	5.00	3.57	0.00	1.67
0.00	12.38	0.08	0.93	0.00	0.00	0.00	0.67
0.00	0.00	0.00	0.47	2.50	0.07	0.00	0.05
12.38	0.00	0.00	0.00	0.00	0.00	10.00	0.00
0.00	12.38	1.00	85.00	0.00	7.14	0.00	2.86
0.00	5.77	0.00	0.93	1.23	0.09	0.66	0.12
0.25	86.66	0.12	1.40	0.21	0.21	0.00	0.00
0.00	12.38	0.00	0.00	0.00	0.00	0.00	0.00
0.50	12.38	0.00	2.83	2.50	3.57	0.00	0.00
1.00	2.89	0.00	2.83	2.50	3.57	0.00	0.00

0.00	37.14	0.12	0.00	21.43	0.00	0.00	0.00
0.00	0.00	0.00	1.40	1.23	0.00	0.00	0.67
1.00	86.66	0.75	5.67	0.00	0.00	0.00	0.00
37.14	0.00	0.00	0.00	1.67	0.00	1.43	0.00
0.00	8.66	1.00	2.83	7.50	0.53	0.07	1.00
1.00	30.95	0.00	2.83	2.50	1.67	0.00	0.71
0.00	0.00	0.00	12.14	10.71	1.67	0.00	0.67
0.49	37.14	0.50	1.40	1.23	3.57	0.00	0.16
45.00	86.66	12.86	14.17	12.50	17.86	0.83	0.00
0.00	86.66	0.00	12.14	0.00	3.57	0.00	10.00
0.00	10.71	11.33	10.00	7.14	4.17	0.33	1.67
12.38	2.14	2.83	5.00	2.50	0.00	10.00	0.00
2.89	0.00	5.67	5.00	7.14	0.14	0.00	14.29
0.00	2.89	0.00	2.83	2.50	3.57	0.14	0.00
21.43	0.47	0.75	5.67	2.50	14.29	14.29	0.05
0.00	173.32	0.12	5.67	5.00	0.21	0.21	1.43
0.00	37.14	0.00	0.23	0.41	3.57	0.00	0.05
2.89	0.00	5.67	5.00	3.57	0.00	0.00	35.71
0.00	12.38	1.00	0.00	5.00	0.00	0.00	0.67
1.00	42.92	1.00	14.98	26.43	0.83	0.00	8.57
0.08	0.47	0.04	12.14	0.21	0.21	0.00	0.33
8.67	0.50	0.00	0.00	0.00	0.00	0.00	14.29
0.00	12.38	0.00	0.00	0.00	0.00	0.00	10.00
0.47	0.50	2.83	2.50	3.57	0.07	0.03	42.86
0.00	37.14	0.00	0.00	0.00	0.00	0.00	0.00
0.00	2.89	0.00	0.00	2.50	3.57	0.00	0.33
0.47	3.00	5.67	2.50	17.86	10.71	0.05	0.14
17.14	24.76	2.14	2.83	5.00	3.57	1.67	0.33
24.76	0.00	0.00	0.00	0.00	0.00	0.00	0.00
3.00	12.38	0.00	0.00	10.71	0.00	1.67	4.29
0.00	12.38	0.50	2.83	0.00	0.00	0.00	0.00
21.43	0.47	3.00	5.67	2.50	17.86	10.71	0.05
30.00	0.00	10.71	14.17	12.50	17.86	0.83	0.00
0.47	3.00	5.67	2.50	17.86	10.71	0.05	0.14
37.50	0.00	12.86	5.67	2.50	3.57	0.00	0.00
14.44	0.00	5.67	5.00	3.57	0.00	0.00	35.71
0.49	86.66	0.25	1.40	1.23	0.41	0.41	0.16
0.00	5.78	0.00	5.67	0.00	0.00	0.00	0.67
0.00	12.38	0.50	0.00	5.00	0.00	0.00	1.43
0.08	12.38	0.08	24.29	2.50	0.83	0.14	1.67
10.11	6.43	14.17	2.50	12.50	1.25	0.00	2.50
37.14	0.00	2.83	5.00	0.83	0.00	0.71	14.29
2.00	24.76	2.14	1.16	5.00	2.50	0.00	0.14
24.76	10.71	14.17	5.00	17.86	1.67	0.00	3.33
30.00	2.89	4.29	8.50	12.50	14.29	0.83	0.00
1.00	55.71	0.00	5.67	2.50	1.67	0.00	0.71
1.00	0.00	2.14	12.14	10.71	3.57	0.00	0.33
0.16	37.11	1.00	0.93	5.17	0.27	0.00	1.00
45.00	2.89	4.29	14.17	5.00	17.86	1.67	0.00
0.00	2.89	0.00	12.14	0.00	0.00	0.00	0.00
0.00	0.00	30.00	0.00	0.00	0.00	0.00	0.00
0.49	173.32	0.12	1.40	10.71	0.21	0.21	0.67
0.00	10.71	12.75	3.13	7.14	4.17	0.33	1.67

0.00	37.14	0.25	0.00	0.00	7.14	0.00	2.86
0.33	12.38	2.14	24.29	21.43	10.71	0.00	0.67
0.00	24.76	6.43	2.83	7.50	0.00	0.00	0.33
5.78	0.00	0.00	2.50	0.83	0.00	10.00	50.00
0.00	12.38	0.00	2.83	2.50	0.00	0.00	0.05
14.44	10.71	0.00	5.00	17.86	1.67	0.50	1.67
30.00	8.67	4.29	5.67	2.50	17.86	1.67	0.00
0.00	49.52	0.00	0.47	5.00	0.00	0.00	0.33
1.00	0.00	1.00	0.00	5.00	0.00	0.00	2.86
0.00	10.71	5.67	2.50	7.14	4.17	0.33	1.67
8.67	6.43	5.67	5.00	14.29	1.67	0.00	3.33
21.43	0.47	3.00	5.67	2.50	14.29	12.50	0.05
2.00	12.38	0.25	5.67	0.00	0.00	0.00	0.00
0.08	24.76	0.04	2.83	5.00	0.83	0.00	0.33
0.50	12.38	0.00	2.83	2.50	3.57	0.00	0.00
0.00	37.14	0.00	5.67	0.00	3.57	0.00	2.86
0.00	0.00	0.00	2.83	0.00	0.00	0.00	10.00
0.00	8.66	1.00	2.83	7.50	0.53	0.07	1.00
1.00	49.52	0.50	2.83	2.50	0.83	0.00	0.67
0.00	18.57	15.00	0.23	2.50	0.17	0.00	0.83
1.00	55.71	0.00	5.67	2.50	1.67	0.00	0.71
0.25	12.38	0.08	12.14	10.71	0.83	0.00	0.33
0.08	1.19	0.29	0.93	2.26	1.33	0.00	10.71
1.00	24.76	0.00	12.14	0.00	0.00	0.00	1.43
1.00	30.95	0.00	2.83	2.50	1.67	0.00	0.71
0.00	2.89	0.00	0.00	2.50	0.83	0.00	0.00
0.00	2.89	0.00	5.67	5.00	3.57	0.00	1.43
24.76	10.71	14.17	5.00	17.86	1.67	0.00	3.33
0.00	2.89	0.00	0.00	3.75	0.83	0.00	10.00
4.29	49.52	0.50	0.00	5.00	0.00	0.00	10.00
0.00	37.14	0.00	0.00	1.23	0.00	0.00	1.43
0.00	14.44	0.00	5.67	5.00	3.57	0.00	0.00
0.00	86.66	0.00	1.16	0.00	0.00	0.00	0.16
0.50	12.38	0.00	2.83	2.50	3.57	0.00	0.00
0.00	86.66	0.00	12.14	5.00	0.00	0.00	2.86
45.00	0.00	10.71	5.67	2.50	7.14	4.17	0.33
0.08	30.95	0.08	24.29	5.00	1.67	0.14	1.67
0.00	61.90	0.00	2.83	10.71	0.00	0.00	0.67
0.00	12.38	0.00	0.00	0.00	0.00	0.00	10.00
0.49	37.14	0.00	5.67	0.00	0.00	0.00	1.43
0.00	37.14	0.00	0.70	0.41	0.14	0.00	0.33
0.00	2.89	0.00	2.83	0.00	0.00	0.00	0.00
0.08	12.38	0.04	12.14	2.50	0.83	0.14	1.67
2.00	73.45	2.00	29.95	42.14	1.67	0.00	14.29
30.00	2.89	11.79	5.67	12.50	7.14	0.83	0.00
0.00	173.32	1.00	5.67	2.05	0.00	0.00	0.50
0.00	2.89	0.00	5.67	5.00	10.71	0.00	1.43
2.89	0.00	2.83	2.50	3.57	0.14	0.00	32.14
0.00	0.00	15.00	85.00	0.00	25.00	0.00	2.86
0.00	30.95	0.06	2.33	1.64	0.00	0.00	0.00
0.00	14.44	0.00	5.67	5.00	3.57	0.00	0.00
0.00	5.78	0.00	0.00	2.50	1.25	0.00	10.00
0.00	0.00	0.00	1.16	1.03	10.71	0.00	0.33

0.16	5.78	0.00	1.40	7.50	0.00	0.00	0.67
0.00	0.00	0.00	12.14	0.00	0.00	0.00	0.33
12.38	0.00	2.83	2.50	3.57	0.00	0.00	21.43
30.00	14.44	10.71	0.00	5.00	7.14	1.67	0.50
0.00	86.66	0.00	0.00	0.00	0.00	0.00	0.00
0.00	5.78	0.00	0.00	2.50	0.83	0.00	10.00
0.00	5.77	0.00	0.93	1.23	0.09	0.66	0.12
0.82	12.38	0.08	0.00	5.00	3.57	0.00	4.29
0.08	5.78	0.00	0.47	0.62	0.00	0.00	0.00
0.00	12.38	0.00	12.14	2.50	0.00	0.00	10.00
37.50	0.00	4.29	5.67	2.50	3.57	0.00	0.00
21.43	0.47	0.75	5.67	2.50	14.29	14.29	0.05
0.00	2.89	0.00	5.67	5.00	7.14	0.21	0.00
1.00	37.14	0.00	2.83	2.50	1.67	0.00	0.71
0.00	0.00	0.00	36.43	0.00	3.57	0.00	4.29
0.00	37.14	0.00	0.00	2.50	0.00	0.00	2.00
0.00	86.66	0.50	0.00	0.00	0.00	0.00	0.00
0.00	8.66	1.00	2.83	7.50	0.53	0.07	1.00
1.00	55.71	0.00	2.83	5.00	0.83	0.00	0.71
0.00	12.38	0.12	0.70	0.62	0.00	0.00	0.33
37.14	0.00	2.83	5.00	0.83	0.00	0.71	14.29
45.00	0.00	6.43	5.67	2.50	17.86	1.67	0.00
0.49	37.14	0.33	8.50	2.50	1.67	0.00	2.86
0.00	8.67	0.25	0.00	10.71	10.71	0.00	0.16
0.00	12.38	0.00	0.00	0.00	0.00	0.00	10.00
0.00	5.78	0.08	1.40	0.41	0.00	0.00	0.33
21.43	0.47	0.75	5.67	2.50	14.29	14.29	0.05
2.00	5.78	0.00	5.67	5.00	12.50	0.00	0.67
0.00	5.78	0.50	0.00	10.71	25.00	0.00	0.33
1.00	2.89	0.00	5.67	2.50	0.00	0.00	0.67
0.00	37.14	0.00	2.83	0.00	0.00	0.00	0.00

kahoo	gojefarang	khiar	sabzikhord	sabzipokht	kadoohalv	kadookhor	bademjoor
3.33	58.80	33.00	12.86	21.86	0.48	1.92	1.90
14.29	98.00	44.00	5.00	109.29	0.00	3.90	82.57
0.00	137.20	33.00	0.00	0.00	0.97	0.00	49.54
14.29	19.60	33.00	2.00	21.86	0.00	7.80	33.03
7.14	137.20	0.84	2.00	1.68	0.48	16.72	0.00
35.71	137.20	33.00	30.00	10.20	0.48	50.17	49.54
14.29	19.60	33.00	1.00	43.71	0.00	15.61	49.54
3.33	58.80	11.00	0.00	0.00	0.08	0.00	0.00
19.60	33.00	2.00	21.86	0.00	7.80	33.03	0.00
7.14	78.40	77.00	8.57	10.20	0.00	3.90	7.71
9.15	88.00	3.00	5.10	0.00	0.00	7.71	0.22
50.00	137.20	77.00	12.86	43.71	0.16	16.72	16.51
78.40	22.00	12.86	43.71	0.16	7.80	7.71	0.11
3.33	137.20	5.13	0.49	10.20	0.24	7.80	7.71
14.29	19.60	44.00	4.29	32.79	0.00	5.85	5.78
14.29	19.60	33.00	1.00	43.71	0.00	15.61	49.54
0.14	9.15	11.00	0.41	25.50	0.00	3.90	1.93
21.43	137.20	77.00	30.00	5.10	0.24	3.90	3.85
3.33	78.40	11.00	8.57	21.86	0.97	3.90	3.85
0.14	9.15	11.00	0.33	10.20	0.00	3.90	1.93
0.00	13.72	2.57	4.29	0.00	0.00	0.00	33.03
2.50	13.72	27.50	1.00	2.55	0.00	3.90	3.85
3.33	19.60	11.00	30.00	153.00	0.08	16.72	7.71
21.43	137.20	77.00	30.00	21.86	0.00	7.80	7.71
14.29	98.00	44.00	5.00	109.29	0.00	3.90	82.57
2.50	2.29	22.00	1.00	2.55	0.08	0.32	7.71
50.00	137.20	77.00	12.86	153.00	0.97	16.72	16.51
100.00	137.20	77.00	30.00	153.00	1.93	16.72	82.57
0.14	9.15	11.00	0.33	10.20	0.00	3.90	1.93
3.33	4.57	16.50	1.00	2.55	0.00	1.95	3.85
1.67	4.57	11.00	1.00	2.55	0.00	0.00	7.71
0.83	2.29	38.50	0.70	7.65	0.16	3.90	3.85
14.29	19.60	33.00	1.00	43.71	0.00	15.61	49.54
35.71	274.40	231.00	90.00	21.86	0.24	0.96	7.71
9.15	44.00	2.00	7.65	0.16	15.61	16.51	0.67
98.00	55.00	4.00	43.71	0.00	15.61	82.57	1.33
5.00	98.00	33.00	12.86	10.20	0.16	3.90	11.56
21.43	98.00	77.00	3.00	21.86	0.24	7.80	7.71
0.00	137.20	55.00	0.00	0.00	0.00	0.00	49.54
3.33	39.20	8.44	0.33	43.71	0.00	0.96	7.71
50.00	137.20	77.00	21.43	21.86	0.48	0.00	7.71
0.83	22.87	88.00	1.50	25.50	0.00	7.80	7.71
3.33	13.72	11.00	1.00	5.10	0.16	0.00	7.71
7.14	137.20	55.00	2.00	21.86	0.97	16.72	16.51
35.71	137.20	77.00	8.57	10.20	0.24	16.72	16.51
7.14	19.60	22.00	8.57	5.10	0.08	3.90	3.85
50.00	137.20	44.00	4.29	21.86	0.00	16.72	16.51
4.57	11.00	1.00	2.55	0.00	1.95	0.00	0.33
14.29	137.20	77.00	1.00	21.86	0.00	3.90	3.85
274.40	44.00	30.00	5.10	0.00	0.00	3.85	0.11
0.00	137.20	77.00	2.00	10.20	0.00	7.80	7.71
14.29	19.60	33.00	2.00	21.86	0.00	7.80	33.03

3.33	137.20	5.13	2.00	21.86	0.48	0.00	0.00
0.00	13.72	2.57	12.86	0.00	0.00	0.00	16.51
1.67	137.20	11.00	30.00	10.20	0.24	0.96	16.51
98.00	38.50	2.00	21.86	0.00	3.90	82.57	1.33
39.20	77.00	8.57	43.71	0.97	3.90	3.85	0.00
100.00	137.20	33.00	30.00	153.00	4.14	16.72	16.51
19.60	33.00	2.00	21.86	0.00	7.80	33.03	0.00
0.00	58.80	77.00	8.57	21.86	0.97	0.00	3.85
0.82	4.57	33.00	1.00	5.10	0.00	0.00	0.00
4.57	22.00	2.00	5.10	0.97	0.00	3.85	0.00
98.00	55.00	4.00	65.57	1.93	7.80	16.51	1.33
21.43	98.00	38.50	2.00	21.86	0.00	3.90	82.57
21.43	137.20	1.05	2.00	21.86	0.00	16.72	16.51
42.86	39.20	55.00	2.00	109.29	0.16	11.71	49.54
3.33	137.20	77.00	3.00	10.20	0.00	7.80	16.51
78.40	22.00	12.86	43.71	0.16	7.80	7.71	0.11
39.20	88.00	6.00	109.29	0.00	11.71	16.51	0.00
0.82	78.40	44.00	4.29	21.86	0.48	50.17	7.71
3.33	39.20	8.44	0.33	43.71	0.00	0.96	7.71
3.33	9.15	33.00	1.00	10.20	0.97	0.00	1.93
137.20	539.00	1.00	10.20	0.00	11.71	23.12	2.67
22.87	55.00	0.33	10.20	4.14	33.45	3.85	1.33
0.00	137.20	0.00	1.00	5.10	0.00	1.92	0.00
7.14	98.00	22.00	2.00	10.20	0.00	16.72	16.51
2.29	22.00	1.00	2.55	0.00	0.00	3.85	0.22
0.00	137.20	33.00	8.57	0.00	0.00	0.00	33.03
7.14	98.00	27.50	1.00	7.65	0.00	3.90	11.56
14.29	27.44	33.00	1.00	5.10	0.40	33.45	16.51
19.60	33.00	1.00	43.71	0.00	0.00	49.54	0.00
17.86	49.00	27.50	5.00	109.29	0.16	11.71	49.54
9.15	55.00	5.00	25.50	0.16	17.56	16.51	6.67
0.83	2.29	38.50	1.50	2.55	0.00	7.80	7.71
2.50	13.72	27.50	1.00	2.55	0.00	3.90	3.85
50.00	137.20	1.48	12.86	21.86	0.00	7.80	7.71
98.00	22.00	2.00	21.86	0.97	3.90	82.57	0.00
0.96	137.20	5.13	12.86	2.52	0.00	0.00	7.71
3.33	19.60	11.00	0.49	10.20	0.08	1.92	1.90
7.14	137.20	33.00	8.57	10.20	0.24	1.60	16.51
21.43	39.20	33.00	2.00	10.20	0.97	0.00	33.03
1.67	4.57	22.00	1.00	2.55	0.00	3.90	0.00
4.57	22.00	1.00	5.10	0.97	1.60	1.93	1.33
19.60	33.00	2.00	21.86	0.97	3.90	82.57	0.00
6.67	137.20	77.00	2.50	87.43	0.00	7.80	7.71
6.67	1.88	22.00	2.00	25.50	0.00	33.45	16.51
42.86	22.87	66.00	8.57	10.20	4.14	33.45	19.27
50.00	9.15	77.00	30.00	21.86	0.24	16.72	49.54
0.00	18.29	5.13	12.86	0.00	0.00	0.00	16.51
0.00	58.80	22.00	2.00	21.86	0.00	0.00	49.54
2.50	13.72	27.50	1.00	2.55	0.00	5.85	3.85
0.00	39.20	77.00	21.43	43.71	1.93	0.00	3.85
0.00	19.60	11.00	4.29	5.10	0.00	0.00	3.85
14.29	98.00	22.00	2.00	21.86	0.97	3.90	82.57
9.15	11.00	0.41	25.50	0.00	3.90	1.93	1.33

39.20	33.00	8.57	43.71	0.97	3.90	3.85	0.00
39.20	22.00	8.57	65.57	0.00	33.45	33.03	2.67
35.71	22.87	55.00	0.33	10.20	4.14	33.45	3.85
21.43	137.20	77.00	4.29	21.86	2.90	50.17	0.00
28.57	78.40	44.00	4.29	20.40	0.00	0.64	7.71
274.40	55.00	0.00	10.20	0.00	0.00	66.06	0.00
7.14	78.40	44.00	1.00	1.68	0.16	11.71	1.90
1.67	137.20	44.00	0.00	21.86	0.00	3.90	33.03
7.14	78.40	11.00	30.00	15.30	0.32	0.00	3.85
50.00	205.80	44.00	30.00	153.00	8.29	50.17	33.03
0.00	4.57	33.00	0.00	0.00	0.00	0.00	49.54
1.67	19.60	44.00	4.29	32.79	0.00	5.85	5.78
14.29	137.20	5.13	30.00	153.00	1.93	0.00	0.00
29.40	33.00	6.43	43.71	0.00	7.80	7.71	0.00
21.43	137.20	33.00	30.00	10.20	0.00	7.80	7.71
0.82	411.60	77.00	12.86	65.57	0.48	7.80	1.90
3.33	19.60	22.00	4.29	21.86	0.00	3.90	3.85
21.43	98.00	55.00	12.86	65.57	0.40	16.72	7.71
21.43	137.20	77.00	21.43	5.10	0.97	0.00	3.85
19.60	22.00	4.29	21.86	0.00	3.90	3.85	0.00
35.71	22.87	66.00	8.57	10.20	4.14	33.45	3.85
50.00	137.20	77.00	30.00	21.86	1.93	16.72	16.51
35.71	137.20	77.00	21.43	10.20	0.24	7.80	7.71
1.67	4.57	11.00	1.00	2.55	0.00	1.60	7.71
14.29	98.00	33.00	30.00	10.20	0.24	50.17	49.54
35.71	274.40	154.00	17.14	65.57	0.97	33.45	16.51
14.29	98.00	44.00	5.00	109.29	0.97	3.90	82.57
25.00	68.60	38.50	30.00	54.64	0.12	4.17	4.12
50.00	137.20	77.00	12.86	0.00	1.93	7.80	7.71
3.33	4.57	55.00	2.00	5.10	0.00	0.00	7.71
21.43	137.20	22.00	30.00	21.86	0.00	3.90	7.71
39.20	22.00	8.57	65.57	0.00	33.45	33.03	2.67
25.00	137.20	77.00	1.00	5.10	0.00	15.61	1.90
7.14	78.40	22.00	12.86	43.71	0.16	7.80	7.71
14.29	137.20	77.00	8.57	43.71	0.00	7.80	16.51
3.33	18.29	22.00	2.00	5.10	0.97	0.00	3.85
17.86	9.15	55.00	5.00	25.50	0.16	17.56	33.03
5.00	4.57	22.00	2.00	20.40	0.97	0.00	3.85
50.00	137.20	77.00	10.71	43.71	0.00	7.80	7.71
28.57	78.40	66.00	4.29	21.86	1.93	3.90	3.85
50.00	137.20	77.00	60.00	5.10	0.00	16.72	16.51
1.67	4.57	11.00	1.00	2.55	0.00	0.00	7.71
21.43	137.20	77.00	30.00	21.86	0.97	3.90	3.85
5.00	98.00	231.00	21.43	21.86	0.48	11.71	7.71
7.14	78.40	44.00	3.00	10.20	0.40	3.90	3.85
50.00	137.20	5.13	30.00	43.71	0.00	3.90	33.03
137.20	22.00	0.00	10.20	0.00	0.00	16.51	0.00
21.43	137.20	77.00	8.57	21.86	2.90	7.80	16.51
3.33	58.80	7.70	4.00	10.20	0.16	1.92	3.80
50.00	274.40	154.00	0.49	2.52	0.00	7.80	16.51
0.00	9.15	5.13	12.86	0.00	0.00	0.00	33.03
21.43	98.00	38.50	3.00	21.86	0.16	3.90	82.57
32.14	98.00	55.00	4.00	43.71	0.00	15.61	33.03

5.00	137.20	7.70	21.43	65.57	0.00	33.45	33.03
0.00	137.20	77.00	21.43	10.20	1.93	7.80	1.90
7.14	98.00	77.00	60.00	21.86	0.97	3.90	9.63
19.60	33.00	1.00	43.71	0.00	0.00	49.54	0.00
7.14	78.40	22.00	12.86	43.71	0.16	7.80	7.71
0.00	19.60	55.00	1.00	43.71	0.00	19.51	49.54
0.00	137.20	11.00	30.00	21.86	0.00	7.80	7.71
7.14	137.20	22.00	4.29	21.86	0.00	3.90	3.85
2.50	2.29	22.00	1.00	2.55	0.08	0.32	7.71
50.00	9.15	77.00	12.86	65.57	4.14	33.45	33.03
2.29	22.00	1.00	2.55	0.00	0.64	3.85	0.22
4.57	11.00	1.00	5.10	0.08	0.96	16.51	1.33
39.20	22.00	5.00	65.57	1.93	7.80	16.51	2.67
32.14	98.00	55.00	5.00	65.57	1.93	7.80	16.51
0.14	9.15	11.00	0.41	25.50	0.00	3.90	1.93
50.00	274.40	154.00	17.14	21.86	0.97	7.80	7.71
50.00	137.20	77.00	30.00	65.57	0.97	16.72	16.51
22.87	88.00	6.00	109.29	0.00	19.51	16.51	2.67
7.14	137.20	55.00	12.86	10.20	0.00	16.72	16.51
50.00	448.80	253.00	68.57	229.50	4.34	21.27	31.93
14.29	137.20	77.00	8.57	65.57	0.40	11.71	3.85
39.20	77.00	8.57	43.71	0.97	3.90	3.85	0.00
0.00	137.20	33.00	0.00	0.00	0.00	0.00	49.54
22.87	66.00	8.57	10.20	4.14	33.45	3.85	1.33
14.29	19.60	33.00	4.29	32.79	0.00	9.76	9.63
28.57	137.20	77.00	8.57	43.71	0.24	1.28	0.00
9.15	11.00	0.33	10.20	0.00	3.90	1.93	1.33
21.43	137.20	22.00	21.43	43.71	0.97	33.45	7.71
18.29	5.13	12.86	0.00	0.00	0.00	16.51	0.00
3.33	58.80	77.00	3.00	10.20	0.16	3.90	7.71
14.29	0.00	7.70	2.00	10.20	0.00	2.25	1.90
0.14	9.15	11.00	0.33	10.20	0.00	3.90	1.93
2.50	9.15	33.00	2.00	2.55	0.00	1.60	7.71
9.15	11.00	0.33	10.20	0.00	3.90	1.93	1.33
1.67	4.57	11.00	1.00	2.55	0.00	0.00	7.71
22.87	88.00	6.00	109.29	0.16	19.51	16.51	6.67
0.00	19.60	33.00	0.49	2.52	0.48	1.92	33.03
0.41	137.20	115.50	2.00	21.86	0.40	0.00	1.58
21.43	137.20	77.00	17.14	43.71	0.32	7.80	7.71
35.71	98.00	38.50	2.00	65.57	0.16	11.71	49.54
16.01	38.50	1.50	7.65	0.16	3.90	3.85	8.57
19.60	33.00	2.00	21.86	0.00	7.80	33.03	0.00
1.67	137.20	22.00	8.57	5.10	0.16	0.96	7.71
13.72	33.00	2.00	10.20	0.00	0.00	7.71	11.43
1.67	9.15	22.00	1.00	5.10	0.97	0.00	5.78
14.29	19.60	33.00	2.00	21.86	0.00	15.61	49.54
1.67	19.60	33.00	4.29	21.86	0.32	16.72	16.51
28.57	78.40	66.00	4.29	21.86	1.93	3.90	3.85
3.33	4.57	22.00	1.00	10.20	0.97	0.00	3.85
17.86	39.20	44.00	17.14	5.10	0.08	3.90	1.27
125.00	274.40	231.00	2.00	15.30	2.38	0.96	33.03
3.33	274.40	154.00	4.29	2.52	0.48	7.80	7.71
2.29	22.00	1.00	2.55	0.00	0.64	3.85	0.22

1.67	137.20	55.00	8.57	5.10	0.00	1.60	16.51
3.33	58.80	33.00	30.00	10.20	0.72	7.80	7.71
50.00	137.20	77.00	60.00	43.71	4.14	50.17	0.00
137.20	77.00	8.57	21.86	1.45	0.00	7.71	0.00
35.71	39.20	22.00	21.43	21.86	0.97	33.45	33.03
9.15	55.00	2.00	5.10	0.00	3.90	7.71	5.71
3.33	9.15	88.00	2.00	12.75	0.00	0.00	7.71
28.57	78.40	44.00	3.00	21.86	4.14	16.72	0.00
7.14	137.20	33.00	30.00	21.86	0.00	7.80	7.71
9.15	22.00	3.00	5.10	0.00	0.00	3.85	0.33
9.15	33.00	1.00	10.20	0.97	0.00	1.93	0.00
0.14	9.15	11.00	0.33	10.20	0.00	3.90	1.93
50.00	137.20	77.00	12.86	10.20	0.32	7.80	115.60
3.33	137.20	33.00	2.00	10.20	0.97	33.45	11.56
21.43	98.00	44.00	5.00	43.71	0.00	3.90	82.57
1.67	137.20	33.00	12.86	43.71	0.00	0.00	16.51
0.00	58.80	77.00	6.43	32.79	0.97	0.00	3.85
7.14	78.40	22.00	12.86	43.71	0.16	7.80	7.71
21.43	137.20	77.00	30.00	21.86	0.97	3.90	3.85
50.00	137.20	77.00	1.00	5.10	0.12	0.32	33.03
14.29	19.60	33.00	2.00	21.86	0.00	15.61	49.54
14.29	39.20	22.00	8.57	43.71	0.32	16.72	3.85
14.29	19.60	22.00	8.57	7.55	0.00	0.00	3.85
21.43	137.20	77.00	17.14	43.71	4.14	33.45	16.51
0.00	19.60	55.00	1.00	43.71	0.00	19.51	49.54
50.00	137.20	77.00	21.43	21.86	0.48	0.00	7.71
25.00	39.20	88.00	6.00	109.29	0.00	11.71	16.51
13.72	11.00	1.00	5.10	0.16	0.00	7.71	11.43
50.00	137.20	77.00	21.43	21.86	1.45	0.00	11.56
21.43	39.20	33.00	1.00	5.10	1.93	3.90	33.03
50.00	137.20	77.00	30.00	65.57	1.93	0.00	16.51
35.71	22.87	88.00	6.00	109.29	0.16	19.51	16.51
3.33	98.00	77.00	30.00	15.30	0.00	3.21	3.85
32.14	98.00	55.00	4.00	43.71	0.00	15.61	82.57
35.71	137.20	77.00	12.86	65.57	0.24	0.96	3.85
1.67	9.15	22.00	1.00	2.55	0.00	0.00	3.85
17.86	9.15	55.00	2.00	65.57	0.16	17.56	33.03
21.43	137.20	77.00	2.50	10.20	0.97	3.90	11.56
0.00	137.20	33.00	0.00	0.00	0.00	0.00	49.54
14.29	98.00	33.00	12.86	65.57	0.24	16.72	7.71
50.00	137.20	77.00	17.14	21.86	4.14	3.90	0.00
50.00	137.20	0.00	2.00	0.00	0.00	0.00	16.51
14.29	98.00	55.00	21.43	120.21	0.16	11.71	49.54
78.57	460.40	429.00	107.14	306.00	4.54	25.81	56.15
1.67	4.57	22.00	1.00	2.55	0.00	3.90	0.00
17.86	68.60	110.00	8.57	76.50	0.40	16.72	33.03
14.29	39.20	88.00	6.00	109.29	0.00	11.71	16.51
98.00	55.00	4.00	65.57	1.93	7.80	16.51	1.33
35.71	0.00	77.00	5.00	43.71	0.00	0.00	0.00
0.55	19.60	7.70	4.29	2.52	0.00	17.56	11.56
35.71	22.87	88.00	6.00	109.29	0.40	19.51	16.51
100.00	137.20	77.00	8.57	21.86	1.45	0.00	7.71
50.00	58.80	77.00	12.86	21.86	4.14	16.72	3.85

5.00	137.20	10.27	4.29	43.71	0.00	3.90	7.71
21.43	39.20	5.13	12.86	5.10	0.97	5.85	5.78
98.00	55.00	4.00	43.71	0.16	3.90	82.57	1.33
1.67	9.15	55.00	2.00	5.10	0.00	3.90	7.71
14.29	19.60	33.00	4.29	32.79	0.00	3.90	3.85
50.00	137.20	77.00	12.86	21.86	1.45	0.00	11.56
3.33	58.80	7.70	4.00	10.20	0.16	1.92	3.80
3.33	137.20	77.00	12.86	21.86	0.40	7.80	16.51
6.67	137.20	55.00	4.00	15.30	0.08	15.61	23.12
0.00	137.20	33.00	0.00	0.00	0.97	0.00	49.54
1.67	4.57	11.00	1.00	2.55	0.00	3.90	0.00
0.14	9.15	11.00	0.41	25.50	0.00	3.90	1.93
14.29	78.40	33.00	5.00	65.57	1.93	7.80	16.51
14.29	19.60	33.00	1.00	43.71	0.00	15.61	49.54
0.00	137.20	77.00	4.29	21.86	0.00	16.72	16.51
1.67	137.20	5.13	2.00	21.86	1.93	3.90	3.85
28.57	19.60	55.00	4.29	21.86	0.00	3.90	3.85
7.14	78.40	22.00	12.86	43.71	0.16	7.80	7.71
14.29	19.60	33.00	2.00	21.86	0.00	7.80	33.03
7.14	58.80	11.00	12.86	43.71	0.08	0.00	0.00
19.60	33.00	2.00	21.86	0.00	7.80	33.03	0.00
3.33	9.15	88.00	3.00	5.10	0.00	0.00	3.85
21.43	137.20	77.00	4.29	43.71	0.79	50.17	49.54
28.57	137.20	77.00	12.86	65.57	0.97	7.80	3.85
0.00	137.20	33.00	0.00	0.00	0.00	0.00	49.54
21.43	137.20	77.00	8.57	43.71	0.16	1.60	16.51
0.14	4.57	22.00	0.41	25.50	0.00	3.90	1.93
0.00	137.20	77.00	4.29	65.57	0.40	7.80	16.51
6.67	39.20	55.00	21.43	21.86	0.00	7.80	15.41
21.43	39.20	77.00	30.00	10.20	0.08	0.32	16.51
50.00	137.20	77.00	8.57	5.10	0.00	7.80	7.71

karafsepok	nokhodsab	loobiasabz	havijekham	havijepokh	sir	piazekham	paizesorkh
2.67	6.40	6.27	25.50	38.21	0.05	0.55	8.14
0.00	9.60	9.40	34.00	30.57	0.20	9.50	2.71
0.00	0.00	0.00	8.50	15.29	0.00	0.00	5.43
0.00	6.40	3.13	34.00	30.57	0.20	9.50	13.57
2.67	0.00	1.55	0.98	0.59	1.29	4.75	19.00
1.33	0.00	13.43	119.00	3.57	3.00	4.75	19.00
0.00	0.00	6.27	8.50	7.64	0.40	23.75	13.57
0.00	0.00	0.00	0.00	0.00	3.00	2.22	0.00
6.40	3.13	34.00	30.57	0.20	9.50	13.57	13.29
0.22	1.58	3.13	17.00	22.93	0.86	33.25	19.00
4.80	4.70	12.75	0.29	0.20	47.50	27.14	0.00
1.33	0.00	13.43	59.50	7.64	0.86	4.75	19.00
0.26	0.26	0.33	5.57	0.29	1.11	2.71	0.13
2.67	6.40	6.27	3.97	3.57	0.05	0.55	19.00
0.00	3.20	3.13	8.50	7.64	0.43	0.00	0.00
0.00	0.00	6.27	8.50	7.64	0.40	23.75	13.57
1.33	3.20	0.64	0.16	15.29	0.10	4.75	13.57
1.33	3.20	6.27	8.50	0.00	3.00	4.75	19.00
1.33	0.00	26.86	1.98	1.78	0.10	4.75	5.43
1.33	8.00	0.64	0.16	3.82	0.10	9.50	13.57
0.00	3.20	0.00	17.00	11.46	0.10	0.00	19.00
8.57	6.40	6.27	4.25	0.29	0.15	9.50	6.79
2.67	3.20	6.27	25.50	7.64	0.20	33.25	19.00
1.33	0.00	3.13	1.98	3.57	0.00	4.75	19.00
0.00	9.60	9.40	34.00	30.57	0.20	9.50	2.71
0.33	1.32	13.43	8.50	0.29	0.20	4.75	5.43
5.71	13.71	13.43	3.97	30.57	3.00	16.63	8.14
2.67	1.58	13.43	59.50	53.50	1.29	33.25	2.71
1.33	3.20	0.26	0.16	3.82	0.10	4.75	8.14
5.71	0.53	13.43	8.50	0.15	0.50	23.75	4.07
0.33	0.53	13.43	17.00	0.59	0.20	4.75	5.43
0.00	4.80	4.70	4.25	0.73	0.15	9.50	6.79
0.00	0.00	6.27	8.50	7.64	0.40	23.75	13.57
1.33	1.05	6.27	59.50	3.57	2.14	66.50	19.00
1.60	3.13	0.33	0.29	0.04	33.25	5.43	13.29
3.20	3.13	8.50	15.29	0.20	26.13	5.43	6.20
1.33	0.00	2.58	9.92	8.92	0.03	1.11	19.00
0.66	1.58	13.43	0.98	1.47	0.43	0.55	19.00
0.00	0.00	0.00	8.50	15.29	0.00	0.00	0.00
5.71	0.26	3.13	0.82	0.00	0.33	2.22	19.00
0.00	0.26	0.26	17.00	7.64	0.00	0.00	0.00
0.22	4.80	4.70	4.25	0.73	1.00	9.50	13.57
11.43	3.20	6.27	8.50	0.00	0.00	4.75	19.00
0.00	1.58	3.13	1.98	1.78	0.10	9.50	13.57
0.00	27.43	6.27	25.50	15.29	0.43	4.75	2.71
0.44	3.20	3.13	0.00	3.57	0.10	1.11	19.00
5.71	6.40	13.43	0.00	15.29	0.86	33.25	19.00
0.53	13.43	25.50	0.15	0.20	23.75	13.57	0.00
0.66	3.20	6.27	59.50	30.57	3.00	33.25	0.00
0.53	3.13	1.98	2.20	0.10	3.33	19.00	3.10
0.22	6.40	6.27	5.95	5.35	0.10	2.22	19.00
0.00	0.00	6.27	17.00	15.29	0.20	9.50	13.57

0.66	0.00	1.55	0.00	0.88	0.20	0.00	13.57
0.00	9.60	0.00	25.50	7.64	0.10	0.00	19.00
5.71	13.71	6.27	0.00	3.57	0.03	2.22	19.00
3.20	3.13	8.50	30.57	0.20	21.38	2.71	6.20
0.53	0.52	8.50	22.93	0.43	0.00	0.00	13.29
2.67	69.00	6.27	59.50	30.57	0.20	33.25	19.00
6.40	3.13	34.00	30.57	0.20	9.50	13.57	13.29
0.00	0.00	0.00	12.75	11.46	0.00	0.00	0.00
0.00	3.20	3.13	1.98	2.64	0.03	6.65	38.00
6.40	6.27	8.50	0.15	0.20	4.75	2.71	0.00
3.20	3.13	8.50	15.29	0.20	26.13	5.43	6.20
1.33	3.20	3.13	8.50	30.57	0.20	21.38	2.71
0.00	3.20	3.13	0.00	0.00	0.86	23.75	19.00
4.00	9.60	9.40	42.50	7.64	0.43	33.25	13.57
2.67	0.00	6.27	8.50	7.64	0.43	4.75	19.00
0.26	0.26	0.33	5.57	0.29	1.11	2.71	0.13
0.00	12.53	25.50	22.93	0.60	19.00	8.14	6.20
2.67	6.40	6.27	0.98	3.57	0.20	14.25	19.00
5.71	0.26	3.13	0.82	0.00	0.33	2.22	19.00
0.00	6.40	6.27	8.50	0.15	0.20	4.75	8.14
2.10	3.13	8.50	4.40	3.00	2.22	40.71	12.40
16.00	0.26	0.16	15.29	0.10	23.75	8.14	7.75
0.66	0.00	0.00	0.00	0.00	0.00	33.25	10.86
1.33	3.20	6.27	8.50	5.35	0.35	4.75	19.00
0.00	0.00	12.75	0.29	0.20	23.75	13.57	0.00
0.00	6.40	0.00	17.00	19.11	0.00	0.00	10.86
1.33	1.58	1.55	0.00	19.11	0.00	0.00	19.00
0.00	0.00	13.43	0.00	7.64	0.00	9.50	19.00
0.00	0.00	8.50	15.29	0.20	0.00	0.00	26.57
4.00	9.60	9.40	17.00	15.29	0.86	66.50	5.43
16.00	15.67	34.00	15.29	2.14	33.25	4.07	26.57
0.22	4.80	4.70	4.25	0.73	0.15	9.50	6.79
8.57	3.20	3.13	4.25	0.29	0.15	9.50	6.79
40.00	13.71	6.27	59.50	53.50	0.02	4.75	13.57
9.60	9.40	34.00	30.57	0.20	9.50	2.71	26.57
0.00	1.58	0.00	0.98	0.88	0.03	9.50	19.00
0.66	41.14	13.43	0.98	3.57	0.05	4.75	13.57
2.67	0.00	0.26	5.95	1.78	3.00	33.25	0.00
11.43	0.00	0.00	8.50	15.29	0.43	0.00	13.57
0.00	0.00	13.43	8.50	0.15	0.20	19.00	10.86
0.53	3.13	1.98	0.88	0.20	23.75	13.57	6.20
9.60	9.40	34.00	30.57	0.20	14.25	5.43	13.29
0.66	0.00	3.13	0.00	0.88	0.15	9.50	5.43
5.71	0.00	12.53	7.93	3.57	0.03	1.11	19.00
6.67	8.00	0.26	17.00	15.29	0.50	28.50	6.79
5.71	6.40	67.14	25.50	22.93	3.00	4.75	13.57
0.00	9.60	0.00	25.50	7.64	0.10	0.00	19.00
0.00	0.00	9.40	0.00	3.57	0.00	0.00	19.00
8.57	3.20	3.13	4.25	0.29	0.15	4.75	6.79
0.00	0.00	0.00	8.50	15.29	0.00	0.00	0.00
0.00	3.20	0.00	0.00	0.00	0.00	4.75	19.00
0.00	9.60	9.40	34.00	30.57	0.20	9.50	2.71
3.20	0.64	0.16	7.64	0.20	9.50	13.57	6.20

0.53	0.52	8.50	19.11	0.43	0.00	0.00	13.29
0.00	0.00	17.00	23.37	2.14	4.75	38.00	6.64
1.33	16.00	0.26	0.16	15.29	0.10	23.75	8.14
1.33	3.20	6.27	25.50	7.64	3.00	0.00	8.14
2.67	9.60	6.27	0.00	15.29	0.30	19.00	19.00
0.00	3.13	17.00	30.57	0.00	0.00	0.00	53.14
0.11	0.53	3.13	3.97	3.57	1.71	33.25	19.00
2.67	0.00	6.27	34.00	15.29	0.00	2.22	10.86
0.00	3.20	6.27	1.98	3.57	0.43	9.50	5.43
0.00	1.32	13.43	25.50	30.57	0.64	0.00	19.00
2.67	0.00	0.00	8.50	0.00	0.00	0.00	0.00
0.00	3.20	3.13	12.75	7.64	0.00	0.00	0.00
5.71	27.43	0.00	17.00	15.29	3.00	33.25	19.00
0.00	7.83	12.75	7.64	0.30	0.00	0.00	13.29
2.67	6.40	6.27	3.97	3.57	1.29	14.25	19.00
17.14	6.40	6.27	8.50	22.93	3.00	14.25	2.71
0.00	3.20	3.13	12.75	7.64	0.00	0.00	0.00
0.55	6.40	13.43	42.50	38.21	1.29	14.25	19.00
1.33	13.71	0.77	25.50	3.57	0.10	2.22	19.00
3.20	3.13	12.75	7.64	0.00	0.00	0.00	26.57
1.33	19.20	0.26	17.00	15.29	0.50	28.50	6.79
17.14	41.14	40.29	25.50	0.00	3.00	14.25	19.00
0.33	27.43	6.27	3.97	7.64	0.43	9.50	19.00
0.33	0.53	13.43	8.50	0.29	0.20	4.75	5.43
1.33	3.20	26.86	8.50	7.64	0.20	14.25	19.00
5.71	13.71	13.43	17.00	15.29	1.29	14.25	5.43
0.00	9.60	9.40	34.00	30.57	0.20	9.50	2.71
0.82	0.53	3.86	12.75	7.64	0.64	7.13	19.00
0.44	3.20	3.13	59.50	3.57	0.86	1.11	0.63
5.71	3.20	6.27	17.00	0.73	0.20	9.50	19.00
0.00	3.20	13.43	8.50	0.00	0.43	0.00	19.00
0.00	0.00	17.00	23.37	2.14	4.75	38.00	6.64
5.33	1.32	12.53	1.98	5.35	0.08	1.11	19.00
0.11	0.26	0.26	0.33	5.57	0.29	1.11	2.71
0.22	6.40	3.13	59.50	7.64	0.43	1.11	19.00
0.00	6.40	6.27	8.50	0.15	0.10	4.75	2.71
2.67	6.40	6.27	34.00	15.29	2.14	33.25	4.07
0.00	6.40	6.27	8.50	0.15	0.20	4.75	10.86
5.71	3.20	13.43	2.98	1.78	0.00	0.00	19.00
1.33	3.20	3.13	17.00	8.38	0.10	9.50	8.14
40.00	0.00	3.13	59.50	7.64	0.43	0.00	19.00
0.33	0.53	13.43	25.50	0.15	0.20	4.75	5.43
1.33	3.20	3.13	119.00	15.29	0.43	9.50	38.00
0.88	41.14	0.77	0.98	22.93	0.86	4.75	13.57
0.88	1.32	1.03	0.65	0.73	0.00	1.11	19.00
1.33	6.40	6.27	3.97	1.78	1.29	0.00	19.00
0.00	3.13	8.50	15.29	0.00	0.00	0.00	13.29
2.67	13.71	13.43	25.50	7.64	0.86	4.75	19.00
4.00	0.26	6.27	0.16	3.22	0.50	0.09	38.00
2.67	6.40	6.27	0.33	7.64	0.86	2.22	5.43
0.00	6.40	0.00	17.00	11.46	0.10	0.00	19.00
1.33	3.20	3.13	8.50	15.29	0.20	26.13	5.43
2.67	6.40	6.27	8.50	15.29	0.20	26.13	5.43

0.66	1.58	0.00	3.97	3.57	0.20	0.00	8.14
2.67	6.40	1.55	3.97	3.57	0.43	33.25	19.00
11.43	3.20	13.43	0.00	22.93	0.00	33.25	19.00
0.00	0.00	8.50	15.29	0.20	0.00	0.00	26.57
0.11	0.26	0.26	0.33	5.57	0.29	1.11	2.71
0.00	0.00	6.27	8.50	7.64	0.40	23.75	13.57
5.71	0.00	6.27	0.00	3.57	0.00	0.00	19.00
0.66	1.58	3.13	8.50	1.78	0.05	9.50	19.00
0.27	0.00	13.43	8.50	0.29	0.20	4.75	5.43
5.71	27.43	13.43	17.00	15.29	3.00	2.22	19.00
0.00	13.43	0.00	0.29	0.20	23.75	13.57	0.00
3.20	3.13	0.33	0.29	0.10	1.11	19.00	26.57
6.40	6.27	17.00	7.64	0.10	19.00	8.14	6.20
1.33	3.20	3.13	17.00	7.64	0.10	19.00	8.14
1.33	3.20	0.64	0.16	15.29	0.10	4.75	13.57
1.33	6.40	6.27	59.50	0.44	3.00	9.50	19.00
11.43	13.71	13.43	8.50	3.57	0.43	33.25	19.00
0.00	12.53	21.25	22.93	0.35	9.50	5.43	6.20
2.67	6.40	6.27	25.50	22.93	0.00	2.22	19.00
6.67	11.20	29.10	23.94	22.93	4.50	38.00	57.00
1.33	3.20	3.13	59.50	22.93	0.00	4.75	19.00
0.53	0.52	8.50	19.11	0.43	0.00	0.00	13.29
0.00	0.00	0.00	8.50	15.29	0.00	0.00	0.00
19.20	0.26	17.00	15.29	0.50	28.50	6.79	7.75
0.00	6.40	6.27	17.00	7.64	0.86	0.00	0.00
2.00	1.84	13.43	21.25	19.11	0.86	33.25	19.00
3.20	0.26	0.16	3.82	0.10	14.25	8.14	6.20
5.71	13.71	13.43	8.50	7.64	3.00	14.25	19.00
9.60	0.00	25.50	7.64	0.10	0.00	19.00	13.29
2.67	9.60	1.03	3.97	3.57	0.05	0.46	13.57
0.44	3.20	3.13	3.97	1.78	0.02	0.00	19.00
1.33	3.20	0.26	0.16	3.82	0.10	14.25	8.14
0.33	0.53	13.43	8.50	0.29	0.20	4.75	5.43
3.20	0.26	0.16	3.82	0.10	14.25	8.14	6.20
0.33	0.53	13.43	25.50	0.15	0.20	4.75	5.43
0.00	12.53	21.25	22.93	0.35	23.75	13.57	6.20
40.00	6.40	6.27	1.98	0.88	0.43	0.55	19.00
0.66	0.00	3.13	2.98	1.78	0.00	4.75	0.63
2.67	6.40	6.27	17.00	7.64	1.71	19.00	13.57
4.00	9.60	9.40	17.00	15.29	0.86	33.25	5.43
6.40	6.27	4.25	0.73	0.15	9.50	6.79	0.51
9.60	9.40	34.00	30.57	0.20	9.50	13.57	13.29
0.22	1.32	13.43	0.98	7.64	0.86	0.09	38.00
3.20	13.43	8.50	0.00	0.00	14.25	19.00	0.00
0.00	0.53	3.13	1.98	0.73	0.40	9.50	5.43
0.00	0.00	6.27	8.50	7.64	0.40	23.75	13.57
1.33	3.20	3.13	17.00	15.29	1.29	19.00	19.00
1.33	3.20	3.13	17.00	8.38	0.10	9.50	8.14
5.71	3.20	6.27	8.50	0.00	0.20	4.75	19.00
0.55	0.79	3.13	25.50	15.29	0.20	0.55	19.00
0.66	0.00	9.40	51.00	0.00	2.14	33.25	1.27
34.29	68.57	67.14	3.97	7.64	0.20	4.75	19.00
0.00	0.00	12.75	0.29	0.20	23.75	13.57	0.00

1.33	0.00	6.27	1.98	1.78	0.10	1.11	19.00
2.67	1.32	6.27	3.97	7.64	1.29	14.25	19.00
11.43	27.43	13.43	25.50	7.64	0.00	14.25	1.27
0.26	0.26	8.50	7.64	0.00	0.00	0.00	26.57
1.33	13.71	6.27	0.98	22.93	0.10	2.22	19.00
3.20	3.13	12.75	0.15	0.20	9.50	19.00	0.00
0.22	4.80	4.70	4.25	0.29	0.20	47.50	27.14
5.71	6.40	0.00	34.00	7.64	0.00	0.00	13.57
1.33	0.00	3.13	1.98	1.78	3.00	0.00	19.00
4.80	4.70	12.75	0.29	0.20	47.50	5.43	0.00
6.40	6.27	8.50	0.15	0.20	4.75	8.14	0.00
1.33	3.20	0.26	0.16	3.82	0.10	4.75	8.14
2.67	3.20	1.03	3.97	7.64	3.00	33.25	19.00
2.00	3.20	3.13	0.00	3.57	0.86	9.50	19.00
1.33	3.20	3.13	8.50	30.57	0.20	21.38	2.71
5.71	27.43	26.86	8.50	7.64	0.20	9.50	19.00
0.00	0.00	0.00	12.75	11.46	0.00	0.00	0.00
0.11	0.26	0.26	0.33	5.57	0.29	1.11	2.71
1.33	3.20	3.13	119.00	15.29	0.43	9.50	38.00
0.16	3.20	3.13	21.25	0.00	0.64	7.13	19.00
0.00	0.00	6.27	8.50	7.64	0.40	23.75	13.57
1.33	3.20	6.27	5.95	5.35	0.10	4.75	19.00
5.33	2.10	0.00	8.50	8.96	0.00	4.75	28.50
5.71	0.00	13.43	59.50	7.64	1.29	33.25	19.00
0.00	0.00	6.27	8.50	7.64	0.40	23.75	13.57
0.00	0.26	0.26	17.00	7.64	0.00	0.00	0.00
0.00	0.00	12.53	21.25	22.93	0.60	19.00	8.14
3.20	13.43	8.50	0.00	0.00	4.75	19.00	0.00
0.00	0.26	0.26	17.00	7.64	0.00	0.00	0.00
11.43	0.00	0.00	8.50	15.29	0.43	0.00	13.57
0.00	6.40	1.03	17.00	22.93	2.14	33.25	19.00
6.67	0.00	12.53	21.25	22.93	0.35	23.75	13.57
1.33	0.00	9.40	8.50	22.93	1.29	33.25	1.27
1.33	3.20	3.13	8.50	15.29	0.20	26.13	5.43
20.00	0.00	6.27	42.50	5.35	1.71	0.00	19.00
0.33	4.80	4.70	12.75	0.29	0.20	23.75	13.57
2.67	6.40	9.40	25.50	22.93	1.29	33.25	4.07
0.00	6.40	6.27	17.00	15.29	0.43	33.25	19.00
0.00	0.00	0.00	8.50	15.29	0.00	0.00	0.00
0.00	0.00	0.00	8.50	7.64	0.10	9.50	8.14
0.00	0.53	13.43	59.50	1.78	1.71	33.25	19.00
5.71	0.00	0.00	0.00	0.00	3.00	14.25	19.00
4.00	9.60	9.40	25.50	22.93	2.14	33.25	2.71
12.00	16.00	51.92	39.38	38.21	6.00	42.75	65.00
11.43	0.53	13.43	8.50	0.15	0.20	19.00	5.43
1.33	3.20	13.43	25.50	0.00	0.10	14.25	19.00
0.00	0.00	12.53	25.50	22.93	0.60	19.00	8.14
3.20	3.13	8.50	15.29	0.20	26.13	5.43	6.20
0.00	41.14	0.00	42.50	0.00	0.00	33.25	19.00
0.66	0.00	0.26	0.00	3.57	3.00	0.00	19.00
6.67	0.00	12.53	21.25	22.93	0.35	23.75	13.57
0.00	0.26	0.26	8.50	7.64	0.00	0.00	0.00
40.00	3.20	0.00	0.00	0.00	3.00	1.11	19.00

2.67	1.58	6.27	29.75	0.00	0.20	4.43	19.00
2.67	0.00	3.13	89.25	0.00	4.50	33.25	0.00
3.20	3.13	8.50	15.29	0.20	26.13	5.43	6.20
5.71	3.20	3.13	12.75	0.15	0.20	9.50	28.50
0.00	3.20	3.13	17.00	7.64	0.86	0.00	0.00
0.00	0.26	0.26	17.00	7.64	0.00	0.00	0.00
4.00	0.26	6.27	0.16	3.22	0.50	0.09	38.00
2.67	6.40	40.29	59.50	3.57	0.43	33.25	19.00
0.33	3.20	12.53	7.93	15.29	0.00	14.25	19.00
0.00	0.00	0.00	8.50	15.29	0.00	0.00	5.43
0.33	0.53	13.43	25.50	0.15	0.20	4.75	13.57
1.33	3.20	0.64	0.16	15.29	0.10	4.75	13.57
2.67	6.40	9.40	25.50	22.93	0.10	19.00	8.14
0.00	0.00	6.27	8.50	7.64	0.40	23.75	13.57
1.33	3.20	3.13	59.50	30.57	3.00	0.00	19.00
1.33	0.00	9.40	0.00	5.35	0.03	0.00	19.00
0.00	0.26	0.26	8.50	19.11	0.43	0.00	0.00
0.11	0.26	0.26	0.33	5.57	0.29	1.11	2.71
0.00	0.00	6.27	17.00	15.29	0.20	9.50	13.57
2.67	0.53	6.27	25.50	22.93	1.29	33.25	19.00
6.40	3.13	34.00	30.57	0.20	9.50	13.57	13.29
0.22	4.80	4.70	12.75	0.29	0.20	47.50	5.43
5.71	13.71	13.43	59.50	7.64	0.86	2.22	13.57
2.67	0.00	1.55	25.50	22.93	2.14	23.75	2.71
0.00	0.00	0.00	8.50	15.29	0.00	0.00	0.00
5.33	3.20	13.43	8.50	7.64	0.20	0.18	19.00
1.33	3.20	0.64	0.16	7.64	0.20	9.50	13.57
5.71	13.71	13.43	0.00	3.57	0.20	33.25	19.00
0.22	0.53	1.29	0.00	15.29	0.86	1.11	19.00
0.66	3.20	3.13	0.00	7.64	0.06	9.50	38.00
40.00	0.00	0.52	119.00	1.78	0.86	33.25	38.00

kalam	felfeldolme	esfenajepc	shalgham	felfelsabze	sosegherm	torshi	shoor
1.53	1.51	1.48	0.94	0.03	0.30	2.09	2.09
26.57	26.23	3.00	1.91	0.11	0.60	54.43	0.00
13.29	0.00	0.00	0.00	0.23	0.00	54.43	0.00
13.29	26.23	3.00	1.91	0.11	0.60	54.43	0.00
0.00	6.12	12.86	0.63	0.11	2.57	0.00	0.00
39.86	91.80	38.57	8.20	0.03	0.00	2.09	2.09
13.29	26.23	3.00	0.00	0.11	0.00	54.43	0.00
13.29	0.00	0.00	8.20	0.00	0.00	0.00	0.00
26.23	3.00	1.91	0.11	0.60	54.43	0.00	0.00
3.10	3.06	0.00	0.47	0.00	1.80	0.00	0.00
0.00	0.00	0.00	0.00	0.60	4.23	0.00	0.00
26.57	13.11	3.00	1.26	0.00	2.57	2.09	2.09
6.12	3.00	1.91	0.11	5.14	2.86	1.33	4.29
0.51	3.06	1.48	8.20	0.03	0.30	8.47	8.47
26.57	0.00	3.00	0.00	0.00	0.00	4.23	0.00
13.29	26.23	3.00	0.00	0.11	5.14	54.43	0.00
6.20	0.00	0.00	0.00	0.00	0.60	54.43	12.70
39.86	6.12	3.00	0.79	0.11	0.60	0.00	0.00
6.20	3.06	6.00	0.00	0.46	0.60	36.29	0.00
6.20	0.00	0.00	0.00	0.00	0.60	36.29	12.70
13.29	6.12	6.00	0.00	0.00	0.00	36.29	0.00
0.51	3.06	3.00	1.91	0.00	0.90	4.23	4.23
0.76	45.90	3.00	0.00	0.02	0.15	0.00	0.00
39.86	6.12	3.00	0.16	0.01	0.60	0.00	0.00
26.57	26.23	3.00	1.91	0.11	0.60	54.43	0.00
0.00	0.38	0.62	0.24	0.11	0.60	0.00	0.00
26.57	4.59	3.00	16.41	0.34	0.60	0.00	0.00
93.00	39.34	6.00	3.83	0.16	0.00	54.43	0.00
7.75	4.59	0.00	0.00	0.00	0.60	36.29	12.70
0.00	0.25	6.00	0.16	0.16	1.20	4.23	0.00
0.00	0.50	0.00	0.00	0.05	1.20	0.00	0.00
0.51	0.00	0.49	0.00	0.05	1.20	0.00	0.00
13.29	26.23	3.00	0.00	0.11	0.00	54.43	0.00
6.20	39.34	1.48	0.94	0.69	2.57	36.29	2.09
12.24	6.00	3.83	0.27	0.05	54.43	4.23	2.14
39.34	6.00	3.83	0.11	2.40	18.14	0.00	0.50
1.27	9.18	0.99	0.16	0.04	0.00	1.04	1.04
39.86	91.80	12.86	0.94	0.03	0.30	1.04	1.04
26.57	0.00	0.00	0.00	0.00	0.00	18.14	0.00
1.55	1.51	0.25	0.00	0.44	0.60	0.67	0.67
39.86	0.00	3.00	0.00	0.00	0.00	12.70	0.00
13.29	0.00	0.00	0.00	0.00	3.00	4.23	0.00
0.00	0.00	0.00	1.91	0.00	2.57	36.29	0.00
6.20	3.06	12.86	3.83	0.00	0.60	0.00	4.23
0.00	26.23	6.00	1.10	2.29	1.20	4.23	4.23
26.57	6.12	3.00	0.63	0.05	0.10	0.00	0.00
13.29	13.11	3.00	1.91	0.00	0.00	18.14	0.00
0.50	3.00	0.00	0.05	1.20	0.00	4.23	2.14
0.00	3.06	1.48	8.20	0.05	2.57	18.14	0.00
13.11	0.25	7.08	0.22	12.00	17.14	17.14	8.57
3.10	3.06	0.49	0.00	0.00	0.00	0.00	18.14
13.29	26.23	3.00	1.91	0.11	0.60	54.43	0.00

0.00	6.12	1.48	8.20	0.00	1.20	2.09	2.09
13.29	3.06	3.00	0.00	0.00	0.00	36.29	0.00
26.57	26.23	3.00	0.47	0.23	0.00	8.47	1.74
26.23	3.00	1.91	0.11	0.60	18.14	0.00	0.50
0.00	3.00	0.00	0.00	0.00	12.70	0.00	2.14
6.20	45.90	25.71	0.00	0.00	0.60	8.47	0.00
26.23	3.00	1.91	0.11	0.60	54.43	0.00	0.00
26.57	0.00	0.00	0.00	0.00	0.00	12.70	0.00
1.55	3.06	3.00	0.00	0.00	15.43	2.30	2.30
0.00	0.00	0.00	0.05	2.57	18.14	6.35	3.21
39.34	3.00	1.91	0.05	0.60	9.07	0.00	0.50
6.20	26.23	3.00	1.91	0.11	0.60	18.14	0.00
0.00	13.11	0.00	0.63	0.69	0.00	0.00	90.71
66.43	15.30	15.00	5.74	1.07	3.00	0.00	8.47
0.51	6.12	0.49	0.00	0.02	1.20	54.43	8.47
6.12	3.00	1.91	0.11	5.14	2.86	1.33	4.29
13.11	0.00	3.83	0.05	0.60	9.07	0.00	0.50
6.20	1.51	6.00	0.94	0.11	0.30	2.09	2.09
1.55	1.51	0.25	0.00	0.44	0.60	0.67	0.67
0.00	3.06	3.00	1.91	0.05	5.14	18.14	4.23
39.34	0.74	0.00	2.35	7.71	1.33	2.67	15.00
4.59	0.00	0.00	0.00	1.20	18.14	4.23	2.14
39.86	3.06	3.00	0.00	0.05	0.00	0.00	4.23
6.20	12.24	6.00	16.41	0.37	1.20	2.12	0.00
0.75	0.00	0.00	0.05	0.00	8.47	0.00	0.00
13.29	3.06	3.00	0.00	0.00	0.00	36.29	0.00
6.20	65.57	3.00	3.83	0.00	0.00	36.29	1.74
13.29	26.23	25.71	0.00	0.00	0.00	0.00	0.00
13.11	0.00	0.00	0.00	0.00	54.43	0.00	4.29
26.57	52.46	15.00	5.74	1.07	3.00	0.00	8.47
6.12	9.00	5.74	0.16	0.54	18.14	4.23	0.00
0.25	0.25	0.25	0.00	0.08	0.90	0.00	0.00
0.51	0.00	6.00	1.91	0.00	0.90	4.23	4.23
66.43	39.34	6.00	0.31	0.69	7.71	36.29	0.00
26.23	3.00	1.91	0.11	0.60	54.43	0.00	0.00
0.00	1.51	0.00	0.94	0.69	7.71	1.74	0.00
1.53	6.12	1.48	1.91	0.00	0.00	1.74	2.09
0.00	13.11	0.00	8.20	0.00	0.10	0.70	0.70
13.29	0.00	0.00	0.00	0.23	2.57	54.43	0.00
0.00	0.50	3.00	0.00	0.11	0.90	4.23	0.00
0.00	0.00	0.00	0.11	0.60	4.23	4.23	0.50
26.23	3.00	1.91	0.11	0.60	54.43	0.00	0.00
12.40	3.06	6.00	7.66	0.00	0.60	18.14	4.23
1.55	3.06	6.00	1.26	0.03	1.80	1.33	2.67
7.75	6.12	0.00	0.00	0.00	1.20	18.14	4.23
39.86	39.34	25.71	0.00	0.23	0.00	1.39	1.39
13.29	3.06	3.00	0.00	0.00	0.00	36.29	0.00
0.00	13.11	0.00	3.83	0.00	0.00	0.00	0.00
0.76	0.00	3.00	1.91	0.08	0.90	0.00	0.00
39.86	0.00	0.00	0.00	0.00	0.00	54.43	0.00
0.51	0.00	1.48	0.00	0.00	0.60	54.43	54.43
26.57	26.23	3.00	1.91	0.11	0.60	54.43	0.00
0.00	0.00	0.00	0.00	0.60	54.43	12.70	6.43

0.00	3.00	0.00	0.00	0.00	16.93	0.00	3.21
0.50	6.00	0.00	0.27	7.71	14.29	0.00	12.86
7.75	4.59	0.00	0.00	0.00	1.20	36.29	12.70
0.00	13.11	12.86	0.00	0.00	1.20	54.43	54.43
39.86	52.46	25.71	0.79	0.00	0.20	72.57	0.70
0.00	0.00	0.00	0.00	0.00	72.57	0.00	8.57
0.00	3.06	18.00	8.20	0.69	0.00	0.00	0.00
13.29	4.59	0.00	0.00	0.00	0.30	0.00	0.00
0.51	13.11	1.48	0.31	0.23	0.60	12.70	4.23
0.00	91.80	12.86	16.41	2.40	0.60	0.00	0.00
39.86	0.00	0.00	0.00	0.23	0.00	54.43	0.00
26.57	0.00	3.00	0.00	0.00	0.00	8.47	0.00
39.86	26.23	90.00	47.42	0.00	0.00	0.00	0.00
0.00	4.50	1.91	0.00	0.00	10.58	0.00	4.29
13.29	39.34	0.99	0.63	0.23	0.00	1.74	1.74
39.86	26.23	0.00	3.83	0.11	0.30	18.14	1.74
26.57	0.00	3.00	0.00	0.00	0.00	8.47	0.00
6.20	6.12	25.71	3.83	0.02	0.39	1.04	1.04
39.86	32.79	3.00	8.20	0.69	0.60	0.00	0.00
0.00	3.00	0.00	0.00	0.00	12.70	0.00	2.14
7.75	4.59	0.00	0.00	0.00	1.20	18.14	4.23
39.86	91.80	12.86	0.94	1.60	0.30	0.00	0.00
6.20	39.34	6.00	0.94	0.03	0.00	36.29	8.47
0.00	0.50	0.00	0.00	0.05	1.20	0.00	0.00
6.20	3.06	0.00	0.94	0.46	2.57	4.23	8.47
13.29	26.23	3.00	1.91	0.01	0.00	4.23	0.00
26.57	26.23	3.00	1.91	0.11	0.60	54.43	0.00
0.00	19.67	3.70	12.30	0.34	0.00	0.00	0.52
26.57	91.80	38.57	0.63	0.05	0.00	0.00	0.00
0.00	0.00	0.00	3.83	0.00	2.57	18.14	4.23
26.57	0.00	3.00	0.00	0.00	0.00	36.29	4.23
0.50	6.00	0.00	0.27	7.71	14.29	0.00	12.86
3.10	3.06	3.00	1.57	0.00	5.14	0.00	0.00
0.13	6.12	3.00	1.91	0.11	5.14	2.86	1.33
26.57	13.11	3.00	0.63	0.46	2.57	9.07	0.00
0.00	0.00	0.00	0.00	0.05	2.57	18.14	6.35
26.57	6.12	9.00	5.74	0.16	0.54	18.14	4.23
0.00	0.00	0.00	0.00	0.05	12.86	18.14	6.35
7.75	13.11	3.00	0.00	0.11	0.60	18.14	0.70
6.64	3.06	1.23	0.47	0.01	1.20	2.86	1.33
93.00	52.46	12.86	24.61	1.60	2.57	54.43	18.14
0.00	0.50	0.00	0.00	0.05	1.20	0.00	4.23
13.29	13.11	6.00	1.57	0.91	2.57	12.70	4.23
1.53	26.23	1.48	1.26	0.91	1.20	36.29	0.70
39.86	52.46	3.00	0.79	0.00	0.30	8.47	0.00
1.53	13.11	6.00	24.61	0.46	0.60	36.29	0.00
0.00	0.00	0.00	0.00	0.00	18.14	0.00	4.29
93.00	26.23	25.71	8.20	0.46	0.00	36.29	18.14
0.38	26.23	1.73	3.15	0.00	1.20	20.00	20.00
1.53	6.12	1.48	3.83	1.60	0.00	127.00	2.09
13.29	3.06	3.00	0.00	0.00	0.00	36.29	0.00
6.20	26.23	3.00	1.91	0.11	0.60	18.14	0.00
6.20	39.34	6.00	3.83	0.11	2.40	18.14	0.00

0.76	13.11	1.48	0.31	0.00	0.00	0.70	2.09
26.57	91.80	1.48	0.00	0.00	0.00	36.29	0.00
13.29	0.00	3.00	5.74	0.23	1.20	36.29	54.43
13.11	0.00	0.00	0.00	0.00	54.43	0.00	4.29
0.13	6.12	3.00	1.91	0.11	5.14	2.86	1.33
13.29	26.23	0.00	0.00	0.11	0.00	54.43	0.00
0.00	13.11	0.00	0.94	0.46	0.00	36.29	0.00
3.10	1.51	1.48	0.63	0.03	0.30	4.23	2.09
0.00	0.38	0.62	0.24	0.11	0.60	0.00	0.00
6.20	6.12	0.00	0.94	0.69	0.00	36.29	0.00
0.25	0.49	0.16	0.05	0.00	8.47	0.00	0.00
3.06	3.00	1.91	0.05	0.05	54.43	4.23	4.29
39.34	3.00	1.91	0.05	0.60	9.07	0.00	0.50
6.20	39.34	3.00	1.91	0.05	0.60	9.07	0.00
6.20	0.00	0.00	0.00	0.00	0.60	54.43	12.70
93.00	0.50	3.00	0.31	1.60	2.57	4.23	4.23
39.86	91.80	38.57	8.20	0.23	0.00	8.47	0.00
13.11	6.00	1.91	0.00	1.20	36.29	0.00	0.50
6.20	39.34	0.25	0.31	0.11	0.30	8.47	8.47
10.83	78.69	16.48	7.00	1.33	5.14	62.90	19.01
93.00	13.11	6.00	3.83	0.23	2.57	8.47	0.00
0.00	3.00	0.00	0.00	0.00	16.93	0.00	3.21
39.86	0.00	0.00	0.00	0.00	0.00	54.43	0.00
6.12	0.00	0.00	0.00	1.20	18.14	4.23	2.14
26.57	0.00	3.00	0.00	0.00	0.00	4.23	0.00
13.29	32.79	0.86	5.74	1.60	0.00	8.47	0.70
0.00	0.00	0.00	0.00	0.60	36.29	12.70	2.14
13.29	26.23	0.00	16.41	0.23	5.14	4.23	4.23
3.06	3.00	0.00	0.00	0.00	36.29	0.00	4.29
0.00	0.50	1.48	0.31	0.05	0.00	4.23	0.00
0.00	6.12	0.49	0.00	0.00	1.20	8.47	0.00
6.20	0.00	0.00	0.00	0.00	0.60	36.29	12.70
0.00	0.50	0.00	0.00	0.05	0.60	0.00	0.00
0.00	0.00	0.00	0.00	0.60	36.29	12.70	2.14
0.00	0.50	3.00	0.00	0.05	1.20	0.00	4.23
13.11	9.00	1.91	0.00	1.20	36.29	0.00	0.50
93.00	3.06	12.86	0.94	1.60	0.30	54.43	2.09
33.21	0.25	0.00	8.20	0.00	0.60	0.00	0.00
1.53	26.23	25.71	8.20	1.60	18.00	36.29	0.00
26.57	15.30	15.00	5.74	1.07	3.00	0.00	8.47
0.00	0.00	2.87	0.00	0.90	0.00	0.00	0.00
26.23	3.00	1.91	0.11	0.60	54.43	0.00	0.00
1.27	26.23	0.74	0.31	0.23	0.00	0.00	2.12
0.00	6.00	1.91	0.00	0.00	18.14	0.00	2.14
3.10	0.25	0.00	0.00	0.00	1.20	0.00	0.00
13.29	26.23	3.00	1.91	0.11	0.60	54.43	0.00
3.10	3.06	3.00	8.20	0.00	0.60	4.23	4.23
6.64	3.06	1.23	0.47	0.01	1.20	2.86	1.33
0.00	13.11	12.86	1.91	0.00	2.57	18.14	4.23
0.00	0.00	0.99	0.00	0.00	0.60	0.00	0.35
0.00	52.46	0.25	0.00	1.14	0.00	54.43	0.00
66.43	13.11	0.74	1.91	0.01	0.30	8.47	2.09
0.75	0.00	0.16	0.05	0.00	8.47	0.00	0.00

0.00	1.01	0.00	0.00	0.23	18.00	54.43	0.00
6.20	0.00	0.49	5.74	0.11	1.20	8.47	0.00
6.20	0.00	25.71	0.00	0.00	1.80	18.14	0.00
0.00	0.00	0.00	0.00	0.00	12.70	0.00	2.14
26.57	26.23	0.49	0.94	0.23	0.60	4.23	4.23
0.00	3.00	3.83	0.05	0.00	18.14	4.23	2.14
0.00	0.00	0.00	0.00	0.00	0.60	4.23	0.00
0.00	52.46	12.86	0.00	0.00	0.00	36.29	0.00
6.20	13.11	0.49	0.63	0.11	0.30	54.43	0.00
0.75	0.00	0.00	0.05	0.00	8.47	0.00	0.00
3.06	3.00	1.91	0.05	5.14	18.14	4.23	2.14
7.75	4.59	0.00	0.00	0.00	1.20	36.29	12.70
13.29	39.34	0.74	0.47	0.69	0.15	1.39	0.00
6.20	39.34	0.49	1.57	0.16	0.90	36.29	36.29
6.20	26.23	3.00	1.91	0.11	0.60	18.14	0.00
3.10	3.06	25.71	0.94	0.00	0.00	8.47	8.47
26.57	0.00	0.00	0.00	0.00	0.00	12.70	0.00
0.13	6.12	3.00	1.91	0.11	5.14	2.86	1.33
13.29	13.11	6.00	1.57	0.91	2.57	12.70	4.23
19.93	19.67	0.37	0.24	0.08	0.60	6.35	27.21
13.29	26.23	3.00	1.91	0.11	0.60	54.43	0.00
3.10	13.11	12.86	8.20	0.05	0.60	4.23	8.47
3.10	3.06	3.00	0.79	0.01	0.05	12.00	1.64
26.57	39.34	12.86	1.91	0.00	0.00	0.00	0.00
13.29	26.23	0.00	0.00	0.11	0.00	54.43	0.00
39.86	0.00	3.00	0.00	0.00	0.00	12.70	0.00
6.20	13.11	0.00	3.83	0.05	1.20	36.29	0.00
0.00	6.00	1.91	0.00	0.00	36.29	0.00	10.71
39.86	0.00	0.00	0.00	0.00	0.00	12.70	0.00
13.29	0.00	0.00	0.00	1.14	5.14	18.14	4.23
39.86	65.57	6.00	0.47	0.23	7.71	8.47	0.00
6.20	13.11	9.00	1.91	0.00	1.20	36.29	0.00
13.29	3.06	3.00	0.47	0.00	0.00	4.23	0.00
6.20	39.34	6.00	3.83	0.11	2.40	18.14	0.00
6.20	3.06	25.71	8.20	0.23	0.60	0.00	0.00
0.00	0.75	0.00	0.00	0.05	0.00	8.47	0.00
26.57	6.12	15.00	5.74	0.05	0.54	0.00	8.47
6.20	26.23	12.86	0.47	1.60	1.20	18.14	0.00
26.57	13.11	0.00	0.00	0.00	0.00	54.43	0.00
0.76	13.11	3.00	0.79	0.11	2.57	8.47	8.47
13.29	91.80	6.00	3.83	0.91	2.57	54.43	54.43
0.00	39.34	0.00	0.00	1.60	1.20	0.00	0.00
13.29	65.57	6.00	3.83	0.11	3.00	0.00	8.47
20.13	104.26	31.48	10.17	1.97	10.29	121.56	97.00
0.00	0.75	6.00	0.16	0.11	0.60	4.23	0.00
0.51	1.01	0.00	24.61	6.00	6.00	18.14	0.00
6.20	13.11	0.00	3.83	0.05	0.60	9.07	0.00
39.34	3.00	1.91	0.05	0.60	9.07	0.00	0.50
66.43	0.00	0.00	0.00	0.00	0.00	0.00	0.00
0.25	91.80	0.37	0.00	0.18	0.00	0.00	0.70
6.20	13.11	6.00	1.91	0.00	1.20	36.29	0.00
26.57	0.00	0.00	0.00	0.00	0.00	12.70	0.00
9.30	52.46	0.00	3.83	0.00	0.00	0.00	0.00

6.20	6.12	6.00	0.31	0.05	0.20	0.00	0.00
26.57	3.06	0.00	8.20	0.00	1.20	0.00	0.00
39.34	6.00	3.83	0.11	0.60	18.14	0.00	0.50
0.00	0.00	3.00	3.83	0.05	0.00	18.14	4.23
26.57	0.00	6.00	0.00	0.00	0.00	12.70	0.00
39.86	0.00	0.00	0.00	0.00	0.00	12.70	0.00
0.38	26.23	1.73	3.15	0.00	1.20	20.00	20.00
6.20	39.34	6.00	3.83	0.00	0.00	1.39	0.00
66.43	26.23	0.49	8.20	0.00	0.00	0.00	0.00
13.29	0.00	0.00	0.00	0.23	0.00	54.43	0.00
0.00	0.50	3.00	0.00	0.05	1.20	0.00	4.23
6.20	0.00	0.00	0.00	0.00	0.60	36.29	12.70
6.20	39.34	3.00	1.91	0.05	0.60	9.07	0.00
13.29	26.23	3.00	0.00	0.11	0.00	54.43	0.00
26.57	13.11	0.99	5.74	0.23	0.00	54.43	0.00
0.00	13.11	0.74	0.63	0.23	0.00	18.14	0.00
13.29	0.00	3.00	0.00	0.00	0.00	12.70	0.00
0.13	6.12	3.00	1.91	0.11	5.14	2.86	1.33
13.29	26.23	3.00	1.91	0.11	0.60	54.43	0.00
0.00	3.06	12.86	0.31	0.01	0.00	0.00	0.00
26.23	3.00	1.91	0.11	0.60	54.43	0.00	0.00
0.00	0.00	0.00	0.00	0.05	0.00	8.47	0.00
3.10	13.11	6.00	0.00	0.00	1.20	0.00	0.00
6.20	9.18	6.00	1.91	0.23	0.60	4.23	4.23
26.57	13.11	0.00	0.00	0.00	0.00	54.43	0.00
6.20	26.23	6.00	0.79	0.05	1.20	4.23	0.00
6.20	0.00	0.00	0.00	0.00	0.60	54.43	12.70
0.00	13.11	1.23	0.94	0.03	0.30	2.09	1.04
0.00	26.23	3.00	0.00	0.46	0.00	0.00	1.04
6.20	13.11	3.00	0.79	1.60	5.14	4.23	4.23
26.57	0.00	3.00	0.00	4.80	0.00	18.14	0.00

khiarshoor	talebi	kharboze	hendevane	golabi	zardaloo	gilas	sib
0.25	39.29	2.63	68.57	1.49	8.06	17.86	48.21
0.00	117.86	5.33	22.86	19.43	4.03	17.86	16.07
4.29	0.00	0.00	0.00	4.53	0.94	0.00	32.14
0.00	39.29	0.00	68.57	0.00	20.14	35.71	80.36
0.16	9.17	10.67	10.67	2.24	0.46	2.05	1.85
0.00	9.17	0.00	10.67	4.53	0.46	4.11	225.00
0.00	39.29	0.00	114.29	38.86	40.29	35.71	80.36
0.00	39.29	22.86	22.86	0.00	0.00	0.00	64.29
39.29	0.00	91.43	19.43	40.29	35.71	80.36	0.00
0.00	9.17	5.33	5.33	0.00	0.00	1.37	16.07
41.25	13.33	45.71	0.00	4.03	8.33	64.29	5.55
0.25	3.77	2.19	22.86	13.60	2.82	12.50	112.50
18.33	2.67	2.67	1.86	1.88	1.17	16.07	11.10
1.00	18.33	10.67	10.67	1.12	1.88	3.42	7.50
2.14	18.33	5.33	0.00	9.07	0.00	0.00	96.43
0.00	39.29	0.00	91.43	38.86	20.14	35.71	80.36
6.43	9.17	0.66	1.32	9.07	2.82	0.34	32.14
0.00	4.52	2.63	2.63	0.75	4.03	4.17	112.50
0.00	9.17	5.33	2.19	2.24	1.88	8.33	32.14
4.29	9.17	0.66	1.32	9.07	2.82	0.34	32.14
4.29	0.00	0.00	0.00	0.00	0.00	0.00	32.14
0.00	18.33	26.67	34.29	4.53	6.04	8.33	48.21
0.00	117.86	22.86	10.67	19.43	8.06	17.86	80.36
0.12	117.86	10.67	68.57	38.86	1.88	8.33	112.50
0.00	117.86	5.33	22.86	19.43	4.03	17.86	16.07
4.29	78.57	13.33	22.86	0.00	8.06	8.33	16.07
0.00	9.17	5.33	68.57	19.43	12.09	17.86	32.14
0.00	4.52	5.33	5.33	0.00	0.46	2.05	0.00
2.14	9.17	10.67	2.67	9.07	2.82	0.34	32.14
0.00	58.93	5.33	45.71	0.00	4.03	4.17	16.07
4.29	39.29	13.33	22.86	4.53	20.14	4.17	16.07
0.00	18.33	26.67	57.14	0.00	6.04	8.33	24.11
0.00	39.29	0.00	114.29	38.86	40.29	35.71	80.36
1.50	9.17	5.33	16.00	4.53	0.94	1.37	16.07
0.00	26.67	26.67	9.07	2.82	12.50	96.43	23.79
58.93	5.33	26.67	0.00	20.14	35.71	32.14	5.55
1.23	3.77	2.19	5.33	0.75	0.94	0.68	64.29
0.12	3.01	1.75	22.86	13.60	0.94	4.17	112.50
4.29	0.00	0.00	0.00	0.00	0.00	0.00	32.14
2.50	2.26	0.22	0.22	2.61	1.16	0.30	11.25
2.14	18.33	10.67	21.33	4.53	0.00	0.00	16.07
0.00	41.25	13.33	114.29	0.00	20.14	8.33	24.11
10.71	0.00	0.00	22.86	19.43	8.06	4.17	16.07
0.50	3.77	10.67	22.86	2.24	0.94	4.17	48.21
0.50	39.29	22.86	22.86	13.60	28.20	71.43	32.14
0.00	9.17	5.33	5.33	4.53	0.94	4.17	3.75
2.14	9.17	5.33	5.33	4.53	8.06	12.50	112.50
39.29	13.33	22.86	4.53	20.14	0.00	32.14	0.00
0.00	0.00	0.00	0.00	58.29	2.82	20.83	112.50
45.21	13.15	65.75	16.77	23.18	13.68	32.14	41.05
2.14	18.33	10.67	22.86	2.24	4.03	17.86	112.50
0.00	39.29	0.00	68.57	0.00	20.14	35.71	80.36

0.25	4.52	4.38	5.33	0.00	0.54	1.71	16.07
4.29	0.00	0.00	0.00	0.00	0.00	0.00	64.29
0.21	9.17	0.88	1.32	0.37	0.46	0.34	48.21
58.93	5.33	10.67	19.43	8.06	17.86	16.07	0.00
18.33	10.67	16.00	18.13	0.00	0.00	64.29	47.57
0.00	39.29	45.71	68.57	4.53	4.03	17.86	80.36
39.29	0.00	68.57	0.00	20.14	35.71	80.36	0.00
2.14	13.75	8.00	5.33	4.53	0.00	0.00	64.29
8.57	0.75	1.10	0.44	0.00	2.32	0.27	22.50
9.17	10.67	45.71	0.00	8.06	4.17	0.00	0.00
58.93	5.33	26.67	0.00	20.14	35.71	32.14	5.55
0.50	58.93	5.33	10.67	19.43	8.06	17.86	16.07
10.71	9.17	0.00	0.00	0.00	0.62	1.37	1.85
0.08	196.43	68.57	68.57	4.53	9.40	4.17	80.36
0.00	4.52	5.33	5.33	4.53	0.94	4.17	48.21
18.33	2.67	2.67	1.86	1.88	1.17	16.07	11.10
58.93	10.67	10.67	0.00	8.06	0.00	48.21	5.55
0.00	117.86	91.43	68.57	2.24	12.09	1.37	1.85
2.50	2.26	0.22	0.22	2.61	1.16	0.30	11.25
2.14	9.17	10.67	114.29	0.00	16.11	6.25	32.14
550.00	285.71	1.32	291.43	80.57	0.45	37.50	11.10
18.33	26.67	26.67	13.60	2.82	0.34	80.36	2.78
0.50	39.29	2.63	2.63	9.07	1.88	8.33	112.50
0.50	9.17	45.71	22.86	4.53	8.06	2.05	48.21
41.25	13.33	80.00	0.00	40.29	16.67	16.07	11.10
4.29	0.00	0.00	0.00	0.00	0.00	0.00	64.29
2.14	39.29	22.86	22.86	1.12	0.46	1.71	7.50
0.00	4.52	2.63	5.33	2.24	0.62	0.68	225.00
39.29	0.00	0.00	0.00	8.06	0.00	32.14	0.00
0.08	196.43	68.57	68.57	9.07	11.28	0.00	80.36
196.43	114.29	68.57	9.07	1.88	20.83	96.43	118.93
0.00	18.33	5.33	34.29	0.75	6.04	8.33	24.11
0.00	18.33	26.67	34.29	4.53	6.04	8.33	48.21
0.00	9.17	16.00	22.86	22.67	1.88	8.33	80.36
117.86	5.33	91.43	19.43	20.14	17.86	16.07	0.00
0.00	9.17	5.33	22.86	0.00	0.93	1.71	7.50
0.25	18.33	10.67	2.63	0.00	4.03	17.86	16.07
0.00	117.86	10.67	68.57	38.86	8.06	8.33	112.50
4.29	0.00	0.00	91.43	9.07	1.88	8.33	32.14
2.14	39.29	5.33	45.71	0.00	8.06	2.08	32.14
39.29	5.33	22.86	0.00	8.06	4.17	16.07	0.00
117.86	5.33	91.43	19.43	20.14	17.86	16.07	0.00
2.14	4.52	2.63	5.33	1.12	0.31	17.86	3.75
2.50	1.51	1.32	1.97	3.73	2.32	0.03	16.07
2.14	45.83	26.67	26.67	45.33	2.82	0.34	80.36
0.00	39.29	22.86	22.86	2.61	0.46	1.37	48.21
4.29	0.00	0.00	0.00	0.00	0.00	0.00	32.14
4.29	18.33	10.67	91.43	0.00	1.88	4.17	112.50
0.00	18.33	26.67	34.29	4.53	6.04	8.33	48.21
4.29	0.00	0.00	0.00	0.00	0.00	0.00	32.14
15.00	18.33	10.67	10.67	19.43	1.88	8.33	16.07
0.00	117.86	5.33	91.43	19.43	20.14	17.86	16.07
9.17	0.66	1.32	9.07	2.82	0.34	32.14	2.78

9.17	26.67	21.33	4.53	0.00	0.00	96.43	71.36
0.75	2.19	2.19	3.73	0.77	0.55	15.41	13.68
2.14	18.33	26.67	26.67	13.60	2.82	0.34	80.36
0.00	0.00	2.19	68.57	0.00	0.94	4.17	112.50
0.21	39.29	22.86	22.86	19.43	4.03	17.86	32.14
0.00	0.00	16.00	0.00	0.00	0.00	64.29	0.00
0.00	4.52	0.00	0.00	0.00	0.00	0.00	7.50
0.08	78.57	0.00	0.00	0.00	0.62	1.37	112.50
0.00	6.03	3.51	3.51	1.49	0.94	8.33	112.50
0.12	39.29	0.88	160.00	19.43	0.15	0.68	112.50
10.71	0.00	0.00	0.00	0.00	0.00	0.00	80.36
2.14	18.33	0.88	0.00	0.75	0.00	0.00	48.21
0.00	78.57	45.71	45.71	38.86	4.03	8.33	112.50
18.33	0.88	0.00	0.75	0.00	0.00	48.21	71.36
0.21	9.17	5.33	5.33	1.49	0.31	0.68	7.50
1.00	4.52	1.75	10.67	38.86	0.46	35.71	112.50
2.14	18.33	0.88	0.00	0.75	0.00	0.00	48.21
1.00	9.17	10.67	68.57	2.61	0.77	0.34	0.62
0.50	0.00	0.88	5.33	2.61	0.46	4.17	112.50
18.33	0.88	0.00	0.75	0.00	0.00	48.21	71.36
2.14	45.83	26.67	26.67	45.33	2.82	0.34	80.36
0.00	55.00	32.00	32.00	4.53	2.82	12.50	48.21
1.00	4.52	45.71	68.57	19.43	4.03	4.17	80.36
4.29	39.29	13.33	22.86	4.53	12.09	4.17	16.07
0.50	9.17	10.67	22.86	0.37	0.94	0.68	80.36
0.12	39.29	0.00	45.71	2.24	0.77	3.42	80.36
0.00	117.86	5.33	91.43	19.43	20.14	17.86	16.07
0.06	9.17	0.00	10.67	4.53	4.03	17.86	112.50
0.00	18.33	10.67	10.67	9.07	1.88	8.33	112.50
2.14	0.00	5.33	22.86	4.53	8.06	4.17	32.14
0.50	78.57	45.71	45.71	19.43	12.09	17.86	32.14
0.75	2.19	2.19	3.73	0.77	0.55	15.41	13.68
0.00	3.01	1.97	8.77	3.73	1.55	0.75	48.21
4.29	18.33	2.67	2.67	1.86	1.88	1.17	16.07
0.00	18.33	10.67	10.67	13.60	4.03	0.00	112.50
3.21	9.17	10.67	114.29	0.00	8.06	4.17	0.00
0.00	78.57	45.71	228.57	4.53	1.88	4.17	48.21
3.21	9.17	10.67	114.29	0.00	8.06	4.17	0.00
0.25	9.17	5.33	10.67	4.53	0.94	6.25	16.07
0.50	1.51	0.22	1.32	2.98	2.32	3.01	64.29
0.08	39.29	22.86	22.86	58.29	16.11	53.57	112.50
2.14	39.29	13.33	22.86	4.53	20.14	4.17	16.07
2.14	39.29	45.71	68.57	38.86	8.06	17.86	32.14
4.29	18.33	10.67	16.00	2.24	0.46	1.03	48.21
0.25	3.77	2.19	5.33	1.49	0.94	4.17	64.29
0.00	18.33	10.67	22.86	19.43	0.94	35.71	64.29
0.00	0.00	16.00	0.00	0.00	0.00	32.14	0.00
2.14	117.86	10.67	10.67	58.29	12.09	8.33	112.50
2.00	6.78	4.82	7.23	7.45	1.55	0.14	128.57
0.00	3.01	45.71	16.00	0.75	4.03	0.68	1.85
4.29	0.00	0.00	0.00	0.00	0.00	0.00	80.36
0.50	58.93	5.33	16.00	19.43	8.06	17.86	16.07
0.50	58.93	5.33	26.67	0.00	20.14	35.71	32.14

0.08	7.53	4.38	2.63	1.12	0.39	3.42	7.50
0.00	78.57	45.71	16.00	9.07	6.04	17.86	112.50
0.00	78.57	0.00	91.43	2.24	0.46	0.00	11.25
39.29	0.00	0.00	0.00	8.06	0.00	32.14	0.00
4.29	18.33	2.67	2.67	1.86	1.88	1.17	16.07
0.00	39.29	0.00	114.29	38.86	4.03	0.00	80.36
0.00	9.17	10.67	10.67	4.53	0.94	1.37	0.00
0.50	4.52	2.63	1.75	1.12	0.46	2.05	3.75
0.00	41.25	13.33	22.86	0.00	8.06	8.33	16.07
0.00	117.86	68.57	68.57	0.00	1.88	8.33	112.50
41.25	13.33	22.86	19.43	4.03	8.33	16.07	5.55
9.17	5.33	5.33	4.53	1.41	4.17	32.14	0.91
58.93	5.33	26.67	9.07	8.06	35.71	32.14	5.55
0.50	58.93	5.33	26.67	0.00	8.06	35.71	32.14
6.43	9.17	0.66	1.32	9.07	2.82	0.34	32.14
2.14	117.86	68.57	68.57	9.07	8.06	35.71	112.50
0.50	117.86	68.57	68.57	4.53	8.06	3.42	112.50
39.29	5.33	5.33	0.00	16.11	8.33	32.14	0.00
6.43	4.52	5.33	5.33	9.07	0.94	35.71	48.21
4.39	115.24	54.86	61.71	37.56	26.05	42.86	385.71
0.50	4.52	0.44	22.86	22.67	3.76	17.86	48.21
18.33	10.67	10.67	9.07	0.00	0.00	64.29	47.57
4.29	0.00	0.00	0.00	0.00	0.00	0.00	32.14
45.83	26.67	26.67	45.33	2.82	0.34	80.36	2.78
2.14	18.33	5.33	0.00	9.07	0.00	0.00	96.43
0.10	39.29	1.53	68.57	9.07	4.03	17.86	112.50
9.17	10.67	2.67	9.07	2.82	0.34	24.11	2.78
2.14	39.29	22.86	22.86	13.60	2.82	12.50	112.50
0.00	0.00	0.00	0.00	0.00	0.00	32.14	0.00
0.00	0.00	0.00	45.71	4.53	0.94	8.33	16.07
1.50	9.17	10.67	68.57	13.60	4.03	17.86	11.25
2.14	9.17	0.44	0.44	9.07	2.82	0.34	24.11
4.29	117.86	13.33	22.86	4.53	12.09	4.17	16.07
9.17	0.44	0.44	9.07	2.82	0.34	24.11	2.78
2.14	39.29	13.33	22.86	4.53	20.14	4.17	16.07
39.29	5.33	5.33	0.00	20.14	2.08	8.04	0.00
0.25	4.52	2.63	22.86	2.24	0.46	17.86	48.21
0.00	39.29	45.71	68.57	0.00	4.03	0.68	96.43
1.00	5.27	68.57	10.67	19.43	8.06	17.86	112.50
0.08	196.43	68.57	68.57	4.53	9.40	4.17	64.29
18.33	26.67	57.14	0.00	6.04	8.33	48.21	0.00
117.86	5.33	91.43	19.43	40.29	35.71	80.36	0.00
1.00	117.86	1.75	1.75	38.86	0.62	35.71	64.29
0.00	0.00	45.71	0.00	8.06	17.86	80.36	0.00
1.00	78.57	5.33	22.86	0.00	20.14	8.33	16.07
0.00	39.29	0.00	91.43	38.86	20.14	35.71	80.36
0.50	39.29	22.86	22.86	4.53	0.94	4.17	16.07
0.50	1.51	0.22	1.32	2.98	2.32	3.01	64.29
2.14	0.00	0.00	22.86	19.43	8.06	4.17	32.14
0.12	0.00	0.00	5.26	58.29	12.09	53.57	112.50
0.00	78.57	0.00	91.43	58.29	16.11	8.33	0.00
0.25	117.86	45.71	91.43	2.24	0.94	4.17	225.00
41.25	13.33	45.71	19.43	4.03	4.17	32.14	11.10

6.43	18.33	10.67	16.00	0.00	0.54	4.17	80.36
1.00	27.50	22.86	45.71	1.12	8.06	8.33	96.43
0.00	117.86	10.67	10.67	38.86	8.06	35.71	112.50
13.75	8.00	5.33	4.53	0.00	0.00	64.29	23.79
0.50	18.33	5.33	10.67	4.53	1.88	8.33	112.50
18.33	10.67	45.71	4.53	8.06	8.33	80.36	0.00
0.00	41.25	13.33	22.86	0.00	20.14	8.33	64.29
0.00	0.00	68.57	45.71	9.07	4.03	17.86	64.29
0.00	5.27	22.86	22.86	0.75	0.46	3.42	32.14
41.25	13.33	80.00	0.00	4.03	16.67	16.07	5.55
9.17	10.67	114.29	0.00	16.11	6.25	32.14	0.00
2.14	9.17	10.67	2.67	9.07	2.82	0.34	32.14
0.00	2.26	2.63	2.63	1.86	0.39	1.71	48.21
0.16	39.29	5.33	45.71	2.24	0.94	4.17	7.50
0.00	117.86	5.33	10.67	19.43	8.06	17.86	16.07
1.00	9.17	5.33	22.86	0.75	0.94	0.34	80.36
2.14	18.33	10.67	5.33	4.53	0.00	0.00	32.14
4.29	18.33	2.67	2.67	1.86	1.88	1.17	16.07
2.14	39.29	45.71	68.57	38.86	8.06	17.86	32.14
3.21	18.33	0.66	68.57	0.37	0.12	0.86	112.50
0.00	39.29	0.00	91.43	38.86	20.14	35.71	80.36
1.00	39.29	0.88	22.86	19.43	4.03	17.86	48.21
1.00	2.26	0.66	0.66	22.36	4.64	0.70	112.50
1.50	9.17	5.33	5.33	1.49	0.94	0.00	32.14
0.00	39.29	0.00	114.29	38.86	4.03	0.00	80.36
2.14	18.33	10.67	21.33	4.53	0.00	0.00	16.07
0.50	39.29	5.33	5.33	0.00	8.06	0.00	16.07
0.00	0.00	91.43	1.86	8.06	17.86	80.36	0.00
2.14	18.33	10.67	5.33	4.53	0.00	0.00	64.29
4.29	0.00	0.00	91.43	9.07	1.88	8.33	32.14
1.50	4.52	2.63	2.63	2.24	0.46	2.05	16.07
0.50	39.29	5.33	5.33	0.00	20.14	2.08	8.04
0.00	0.00	1.32	5.33	4.53	0.00	0.00	112.50
0.50	58.93	5.33	26.67	0.00	20.14	35.71	32.14
0.00	9.17	0.44	10.67	4.53	8.06	35.71	112.50
0.00	41.25	13.33	80.00	0.00	40.29	16.67	16.07
0.00	235.71	137.14	137.14	4.53	1.88	4.17	80.36
0.50	18.33	3.51	22.86	4.53	1.88	8.33	48.21
4.29	39.29	0.00	0.00	0.00	0.00	0.00	32.14
2.14	117.86	91.43	91.43	4.53	0.94	12.50	16.07
2.14	39.29	68.57	68.57	19.43	4.03	17.86	112.50
0.00	18.33	45.71	45.71	0.00	1.88	35.71	112.50
0.08	39.29	45.71	114.29	9.07	11.28	0.00	32.14
8.78	112.14	99.05	118.10	75.12	40.02	77.38	358.93
0.00	58.93	5.33	45.71	0.00	8.06	2.08	40.18
0.00	3.01	22.86	22.86	1.49	0.31	0.68	112.50
0.50	58.93	10.67	10.67	0.00	8.06	0.00	48.21
58.93	5.33	26.67	0.00	20.14	35.71	32.14	5.55
0.00	0.00	0.00	0.00	4.53	8.06	17.86	168.75
0.08	6.03	34.29	22.86	2.98	1.88	15.41	48.21
0.50	39.29	5.33	5.33	0.00	20.14	2.08	32.14
2.14	13.75	8.00	5.33	4.53	0.00	0.00	64.29
0.00	39.29	22.86	22.86	19.43	4.03	17.86	112.50

0.00	3.01	10.67	10.67	0.75	1.88	0.00	0.00
0.23	18.33	10.67	22.86	9.07	1.88	0.00	80.36
58.93	5.33	26.67	19.43	8.06	17.86	16.07	0.00
2.14	18.33	10.67	45.71	4.53	8.06	8.33	64.29
3.21	9.17	16.00	0.00	4.53	0.00	0.00	96.43
2.14	13.75	8.00	5.33	4.53	0.00	0.00	64.29
2.00	6.78	4.82	7.23	7.45	1.55	0.14	128.57
0.00	9.17	5.33	5.33	1.86	0.46	1.71	112.50
0.00	0.00	0.00	10.67	0.00	1.88	1.03	16.07
4.29	0.00	0.00	0.00	4.53	0.94	0.00	32.14
2.14	39.29	13.33	22.86	4.53	20.14	0.00	16.07
4.29	9.17	0.66	1.32	9.07	2.82	0.34	32.14
0.50	58.93	5.33	10.67	13.60	8.06	35.71	32.14
0.00	39.29	0.00	91.43	38.86	20.14	35.71	80.36
0.00	117.86	68.57	0.00	58.29	12.09	53.57	112.50
0.16	6.78	3.95	5.33	9.07	0.94	0.34	3.75
3.21	9.17	26.67	21.33	4.53	0.00	0.00	96.43
4.29	18.33	2.67	2.67	1.86	1.88	1.17	16.07
0.00	39.29	0.00	68.57	0.00	20.14	35.71	80.36
0.50	3.77	2.63	2.63	9.07	1.88	8.33	112.50
39.29	0.00	91.43	19.43	40.29	35.71	80.36	0.00
0.00	41.25	13.33	45.71	0.00	4.03	8.33	64.29
2.00	3.01	10.67	21.33	2.24	0.94	8.33	64.29
0.50	9.17	0.00	5.33	1.49	0.46	1.37	16.07
4.29	39.29	0.00	0.00	0.00	0.00	0.00	32.14
0.08	18.33	10.67	10.67	2.98	1.88	8.33	112.50
6.43	9.17	0.66	1.32	9.07	2.82	0.34	32.14
0.25	27.50	22.86	45.71	0.75	0.31	12.50	80.36
0.12	78.57	45.71	68.57	38.86	0.00	0.00	18.75
0.50	9.17	5.33	5.33	2.24	1.88	17.86	16.07
0.00	18.33	10.67	10.67	9.07	8.06	1.37	112.50

holoo	shalil	gojesabz	anjiretaze	anjirekhosl	angoor	kivi	gripfruit
11.10	12.86	0.80	0.14	0.08	59.14	6.00	1.34
0.00	1.50	13.93	0.00	0.71	98.57	6.00	0.67
0.00	6.43	0.00	0.00	0.71	0.00	0.00	0.00
0.00	1.50	13.93	0.00	0.71	98.57	6.00	0.67
1.82	0.74	0.13	0.28	1.43	59.14	3.00	0.00
5.55	1.50	1.39	0.00	0.67	59.14	12.86	0.67
0.00	1.50	0.00	0.00	0.71	98.57	6.00	0.67
11.10	3.00	0.00	0.00	0.05	9.20	6.00	0.00
1.50	13.93	0.00	0.71	98.57	6.00	0.67	42.86
11.10	3.00	0.00	1.15	0.17	39.43	12.86	0.00
0.00	0.33	0.00	0.00	4.60	3.00	4.08	0.00
11.10	3.00	0.11	0.71	0.05	19.71	12.86	0.00
1.50	0.13	0.33	0.10	4.60	3.00	0.00	10.00
11.10	3.00	0.21	0.14	0.03	9.20	0.99	1.01
71.36	3.00	0.00	0.00	0.00	18.40	12.86	0.00
0.00	1.50	13.93	0.00	0.71	98.57	6.00	0.67
2.78	0.75	0.13	1.15	0.03	2.30	3.00	0.34
5.55	1.50	0.00	0.24	0.05	19.71	12.86	0.67
16.65	4.50	0.05	1.15	0.00	3.02	3.00	0.00
5.55	0.75	0.13	1.15	0.03	2.30	3.00	0.34
0.00	2.25	0.65	0.00	0.00	19.71	0.00	0.00
5.55	0.00	0.00	0.00	0.00	0.00	6.43	8.75
23.79	12.86	0.27	2.47	0.00	19.71	1.48	0.00
71.36	19.29	0.00	1.15	0.50	59.14	6.00	0.00
0.00	1.50	13.93	0.00	0.71	98.57	6.00	0.67
5.55	0.00	0.00	0.09	0.00	4.60	3.00	4.08
11.10	3.00	2.79	7.41	3.57	19.71	12.86	4.08
47.57	0.74	0.16	4.94	0.08	19.71	51.43	1.34
2.78	0.75	0.16	0.00	0.03	2.30	3.00	0.34
0.00	1.50	0.65	0.00	0.00	4.60	3.00	0.34
11.10	0.00	0.00	0.09	0.71	9.20	3.00	0.00
0.00	0.00	0.00	2.47	0.71	0.00	12.86	0.00
0.00	1.50	0.00	0.00	0.71	98.57	6.00	0.67
5.55	1.50	0.11	0.86	0.00	59.14	9.00	0.00
6.43	1.39	2.47	0.00	4.60	6.00	0.00	0.82
0.00	5.57	0.58	0.00	0.00	3.00	0.34	42.86
11.10	3.00	0.11	0.28	1.00	3.78	6.00	8.17
23.79	6.43	1.39	0.47	0.14	39.43	1.48	1.01
0.00	0.00	0.00	0.00	0.71	0.00	0.00	0.00
0.00	12.33	1.18	0.47	0.01	3.40	0.74	0.00
47.57	0.12	0.00	0.00	0.00	19.71	0.00	0.00
0.00	0.00	0.00	2.47	0.71	4.60	0.00	0.00
0.00	1.50	0.00	0.47	1.43	4.60	0.00	0.00
5.55	1.50	0.21	0.58	0.17	9.20	3.00	0.00
71.36	19.29	2.60	9.87	0.14	59.14	9.00	52.50
2.28	0.62	0.13	0.24	0.00	0.00	0.99	0.00
47.57	12.86	0.00	0.58	0.71	39.43	25.71	0.00
1.50	0.00	0.09	0.71	9.20	3.00	0.00	5.00
47.57	12.86	3.25	0.47	0.17	9.20	6.00	0.00
11.10	0.27	4.73	1.37	68.05	22.19	8.39	82.19
23.79	6.43	0.16	2.47	0.03	19.71	38.57	8.17
0.00	1.50	13.93	0.00	0.71	98.57	6.00	0.67

3.19	0.86	0.00	0.00	0.00	0.00	27.00	0.00
0.00	1.50	0.33	0.00	0.71	19.71	0.00	0.00
1.82	0.74	0.08	0.09	0.00	9.20	1.23	0.00
1.50	5.57	0.58	0.71	19.71	6.00	0.67	42.86
0.12	0.00	0.00	0.00	19.71	0.00	0.00	15.00
23.79	12.86	0.33	2.47	0.00	59.14	0.00	0.00
1.50	13.93	0.00	0.71	98.57	6.00	0.67	42.86
23.79	0.12	0.00	0.00	0.00	19.71	0.00	0.00
2.28	0.86	1.34	0.24	0.00	0.38	0.74	0.00
3.00	3.25	0.58	0.00	9.20	0.00	0.00	21.43
0.00	5.57	0.58	0.00	0.00	6.00	0.67	42.86
0.00	1.50	5.57	0.58	0.71	19.71	6.00	0.67
0.00	0.00	0.00	0.24	0.00	13.80	1.48	0.00
23.79	12.86	4.18	24.69	0.33	23.00	6.00	8.17
3.19	1.50	0.33	0.58	0.00	0.76	6.00	0.00
1.50	0.13	0.33	0.10	4.60	3.00	0.00	10.00
0.00	2.79	0.58	0.00	9.20	7.50	0.00	42.86
2.74	19.29	0.16	2.47	0.08	59.14	3.00	2.01
0.00	12.33	1.18	0.47	0.01	3.40	0.74	0.00
0.00	0.00	0.65	0.58	0.00	11.50	12.86	0.00
30.00	3.58	1.73	0.00	4.60	6.00	1.34	150.00
0.75	0.16	0.00	0.03	2.30	3.00	0.34	10.00
11.10	3.00	0.16	0.28	0.00	2.27	1.48	2.01
11.10	0.62	0.98	1.73	0.00	9.20	6.00	0.00
1.50	0.00	0.09	0.00	4.60	3.00	4.08	10.00
0.00	1.50	0.33	0.00	0.71	0.00	0.00	0.00
5.55	3.00	0.08	0.28	0.00	9.20	6.00	17.50
2.74	0.99	0.16	0.33	0.08	4.60	3.00	0.00
1.50	0.00	0.00	0.71	0.00	3.00	0.00	42.86
23.79	12.86	4.18	24.69	0.33	23.00	6.00	8.17
19.29	6.96	12.34	0.33	4.60	6.00	8.17	107.14
0.00	0.00	0.00	2.47	0.71	0.00	12.86	0.00
0.00	1.50	0.33	0.58	0.00	0.00	6.43	8.75
23.79	6.43	0.98	2.30	0.71	9.20	38.57	0.34
1.50	13.93	0.00	0.71	98.57	6.00	0.67	42.86
0.91	0.25	0.00	0.00	0.00	4.60	3.00	0.34
23.79	6.43	0.27	0.00	0.00	4.60	3.00	0.00
71.36	19.29	0.13	0.14	0.01	59.14	12.86	0.00
23.79	6.43	0.00	0.00	0.71	4.60	3.00	4.08
0.00	1.50	0.00	0.09	0.71	9.20	3.00	4.08
1.50	0.33	0.00	0.33	0.00	0.00	1.34	10.00
1.50	13.93	0.00	0.71	98.57	6.00	0.67	42.86
47.57	12.86	6.50	0.00	0.17	4.60	2.47	0.00
4.56	2.47	1.07	0.95	0.14	1.51	3.70	0.00
2.78	0.75	0.16	0.00	0.03	2.30	3.00	0.34
3.19	0.86	0.00	0.24	0.00	39.43	38.57	0.67
0.00	1.50	0.33	0.00	0.71	0.00	0.00	0.00
71.36	19.29	0.00	12.34	0.00	116.86	90.00	0.00
0.00	0.00	0.00	0.00	0.00	0.00	12.86	17.50
0.00	0.00	0.00	0.00	0.71	0.00	0.00	0.00
11.10	3.00	0.65	1.15	0.00	0.00	6.00	0.00
0.00	1.50	13.93	0.00	0.71	98.57	6.00	0.67
0.75	0.13	1.15	0.03	2.30	3.00	0.34	5.00

0.49	0.00	0.00	0.00	19.71	0.00	0.00	20.00
2.47	1.60	0.47	0.41	3.78	2.47	0.00	20.55
2.78	0.75	0.16	0.00	0.03	2.30	3.00	0.34
71.36	3.00	0.00	0.00	0.03	59.14	6.00	1.34
11.10	3.00	0.19	1.73	0.00	19.71	12.86	0.00
0.00	0.00	0.00	1.43	0.00	0.00	0.00	42.86
4.56	1.23	0.00	0.19	0.00	2.27	0.74	0.00
4.56	1.23	0.00	0.00	0.00	39.43	0.00	0.00
0.00	12.86	0.21	0.19	0.05	4.60	6.00	1.01
71.36	0.37	0.00	2.47	0.08	59.14	0.00	0.00
0.00	7.50	0.00	0.00	0.71	0.00	0.00	0.00
71.36	1.50	0.00	0.00	0.00	13.80	12.86	0.00
47.57	12.86	0.65	1.15	5.00	9.20	25.71	35.00
1.50	0.00	0.00	0.00	9.20	12.86	0.00	15.00
1.82	0.49	0.19	0.38	0.07	9.20	0.99	0.00
23.79	47.86	9.61	11.52	1.43	19.71	1.48	8.17
71.36	1.50	0.00	0.00	0.00	13.80	12.86	0.00
5.55	3.00	0.13	0.58	0.07	39.43	6.00	0.67
5.55	1.50	0.19	0.05	0.05	39.43	6.00	0.00
1.50	0.00	0.00	0.00	13.80	12.86	0.00	15.00
2.78	0.75	0.16	0.00	0.03	2.30	3.00	0.34
11.10	4.50	0.27	0.58	5.00	59.14	18.00	4.08
23.79	12.86	0.16	2.47	0.83	59.14	12.86	0.00
11.10	0.00	0.00	0.09	0.71	9.20	3.00	0.00
11.10	6.43	0.05	0.58	0.14	4.60	6.00	0.00
11.10	3.00	0.33	0.57	0.00	9.20	0.00	8.17
0.00	1.50	13.93	0.00	0.71	98.57	6.00	0.67
23.79	6.43	1.39	2.47	0.03	19.71	12.86	0.00
47.57	12.86	0.13	0.24	0.17	19.71	9.00	0.67
0.00	3.00	0.33	0.00	0.71	9.20	12.86	0.00
47.57	12.86	0.00	4.94	2.14	49.29	25.71	0.00
2.47	1.60	0.47	0.41	3.78	2.47	0.00	20.55
3.19	1.23	0.13	0.47	0.00	5.29	2.47	1.01
11.10	1.50	0.13	0.33	0.10	4.60	3.00	0.00
47.57	12.86	0.13	2.47	0.50	59.14	25.71	12.25
0.00	3.00	3.25	0.58	0.00	9.20	0.00	0.00
47.57	32.14	2.79	4.94	0.33	4.60	6.00	8.17
0.00	3.00	3.25	0.58	0.00	9.20	0.00	0.00
47.57	12.86	0.98	0.58	0.42	9.20	25.71	0.00
9.12	2.47	0.80	0.95	0.17	3.78	1.23	1.34
23.79	6.43	0.65	4.94	14.00	9.20	51.43	0.00
11.10	0.00	0.00	0.09	0.71	9.20	3.00	0.00
23.79	6.43	0.98	3.46	0.83	18.40	25.71	1.01
4.56	1.23	0.16	0.47	0.00	9.20	3.00	0.00
5.55	1.50	0.13	0.58	0.15	9.20	6.00	1.01
47.57	12.86	1.39	4.94	0.00	19.71	0.00	4.08
0.00	0.00	0.00	0.71	0.00	0.00	0.00	42.86
11.10	3.00	4.18	0.14	0.71	59.14	6.00	17.50
11.40	2.47	1.34	1.42	0.83	9.45	4.93	5.03
2.74	0.74	0.00	1.73	0.00	2.27	6.00	4.08
0.00	2.25	0.65	0.00	0.00	19.71	0.00	0.00
0.00	1.50	5.57	0.58	0.71	19.71	3.00	0.34
5.55	0.00	5.57	0.58	0.00	0.00	3.00	0.34

3.65	0.99	0.00	0.33	0.08	9.20	2.22	0.00
23.79	6.43	0.00	1.15	0.00	9.20	38.57	0.00
0.00	6.43	0.27	14.56	0.00	59.14	1.97	0.00
1.50	0.00	0.00	0.71	0.00	3.00	0.00	42.86
11.10	1.50	0.13	0.33	0.10	4.60	3.00	0.00
0.00	1.50	0.00	0.00	0.71	98.57	6.00	0.67
5.55	1.50	0.00	1.15	0.17	39.43	6.00	0.00
5.55	3.00	0.05	0.28	0.08	2.27	1.48	0.34
5.55	0.00	0.00	0.09	0.00	4.60	3.00	4.08
11.10	1.50	0.40	1.15	3.57	59.14	38.57	1.01
0.00	0.00	0.09	0.00	4.60	3.00	4.08	10.00
0.25	0.27	0.47	1.43	9.20	6.00	4.08	0.41
0.00	1.39	0.58	0.00	0.00	6.00	0.67	42.86
5.55	0.00	1.39	0.58	0.00	0.00	6.00	0.67
2.78	0.75	0.13	1.15	0.03	2.30	3.00	0.34
47.57	12.86	1.63	1.15	0.08	19.71	12.86	17.50
47.57	12.86	0.80	1.73	2.14	9.20	38.57	0.34
6.43	2.79	0.58	0.00	9.20	7.50	0.00	85.71
5.55	1.50	0.65	0.24	0.71	9.20	6.00	0.00
58.67	32.14	9.02	11.03	1.48	118.29	12.86	8.17
23.79	7.50	1.95	2.88	0.07	19.71	12.86	0.00
0.12	0.00	0.00	0.00	19.71	0.00	0.00	15.00
0.00	0.00	0.00	0.00	0.71	0.00	0.00	0.00
0.75	0.16	0.00	0.03	2.30	3.00	0.34	10.00
71.36	3.00	0.00	0.00	0.00	18.40	12.86	0.00
23.79	19.29	4.18	7.41	12.00	59.14	0.00	52.50
1.50	0.33	0.00	0.03	2.30	3.00	0.34	10.00
23.79	6.43	1.63	2.47	2.14	39.43	6.00	0.00
1.50	0.33	0.00	0.71	0.00	0.00	0.00	85.71
47.57	19.29	0.16	0.19	0.50	39.43	9.00	1.34
23.79	12.86	0.53	1.15	0.00	19.71	0.00	0.00
2.78	1.50	0.03	0.00	0.03	2.30	3.00	0.34
11.10	0.00	0.00	0.09	0.00	4.60	3.00	0.00
1.50	0.03	0.00	0.03	2.30	3.00	0.34	10.00
0.00	0.00	0.00	0.09	0.71	9.20	3.00	0.00
6.43	2.79	0.58	0.00	9.20	7.50	0.00	64.29
23.79	6.43	0.16	2.47	0.08	39.43	25.71	1.34
5.55	0.00	0.27	0.09	0.05	19.71	12.86	0.00
47.57	12.86	0.16	2.47	0.00	19.71	25.71	0.00
71.36	19.29	4.18	7.41	0.33	23.00	6.00	8.17
0.00	2.79	4.94	0.00	0.00	0.00	0.00	42.86
1.50	13.93	0.00	0.71	98.57	6.00	0.67	42.86
71.36	19.29	6.96	0.38	0.03	19.71	25.71	0.00
1.50	0.00	0.00	1.43	4.60	0.00	0.00	25.00
0.00	3.00	0.33	0.00	0.33	0.00	0.00	1.34
0.00	1.50	13.93	0.00	0.71	98.57	6.00	0.67
5.55	6.43	0.21	0.58	0.71	19.71	3.00	1.68
9.12	2.47	0.80	0.95	0.17	3.78	1.23	1.34
0.00	1.50	0.00	0.47	1.43	4.60	12.86	0.00
71.36	19.29	0.11	0.19	2.14	39.43	51.43	26.25
47.57	12.86	5.57	0.33	0.00	0.38	0.00	1.01
47.57	12.86	0.16	0.14	0.08	29.57	2.47	0.67
1.50	0.00	0.09	0.00	4.60	3.00	4.08	10.00

5.55	1.50	0.27	0.00	0.08	9.20	0.00	0.00
23.79	12.86	0.98	1.73	0.04	19.71	3.00	17.50
47.57	12.86	2.79	2.47	0.33	39.43	12.86	4.08
0.12	0.00	0.00	0.00	19.71	0.00	0.00	5.00
23.79	6.43	0.21	7.41	0.50	9.20	1.48	3.36
3.00	0.65	0.00	0.71	9.20	12.86	0.00	42.86
5.55	0.00	0.33	0.00	0.00	4.60	0.00	0.00
11.10	3.00	0.65	2.47	0.00	39.43	12.86	0.00
6.84	0.74	0.53	0.47	0.33	13.80	0.74	0.00
0.00	0.33	0.09	0.00	4.60	3.00	4.08	10.00
0.00	0.65	0.58	0.00	11.50	12.86	0.00	21.43
2.78	0.75	0.16	0.00	0.03	2.30	3.00	0.34
2.28	0.86	0.27	0.33	0.33	9.20	0.74	0.34
47.57	12.86	0.03	0.47	0.00	19.71	12.86	0.00
0.00	1.50	6.96	0.58	0.71	19.71	6.00	0.67
3.65	1.50	0.08	0.28	0.00	39.43	6.00	0.00
0.00	0.25	0.00	0.00	0.71	0.00	0.00	0.00
11.10	1.50	0.13	0.33	0.10	4.60	3.00	0.00
23.79	6.43	0.98	3.46	0.83	18.40	25.71	1.01
23.79	3.00	0.04	0.00	0.00	9.20	3.00	0.00
0.00	1.50	13.93	0.00	0.71	98.57	6.00	0.67
23.79	6.43	0.65	1.15	0.04	19.71	3.00	0.00
27.37	7.40	0.88	0.85	0.14	4.54	4.93	0.34
5.55	1.50	0.05	0.00	0.00	9.20	3.00	4.08
0.00	1.50	0.00	0.00	0.71	98.57	6.00	0.67
47.57	0.12	0.00	0.00	0.00	19.71	0.00	0.00
5.55	0.00	2.79	0.58	0.00	9.20	7.50	0.00
1.50	0.00	0.47	1.43	4.60	0.00	0.00	42.86
23.79	0.12	0.00	0.00	0.00	19.71	0.00	0.00
47.57	12.86	0.00	0.00	0.71	4.60	3.00	4.08
23.79	6.43	0.40	0.38	0.33	19.71	3.00	0.00
0.00	6.43	2.79	0.58	0.00	9.20	7.50	0.00
0.00	0.00	0.05	0.00	0.00	39.43	1.23	0.00
5.55	0.00	5.57	0.58	0.00	0.00	3.00	0.34
47.57	12.86	2.79	4.94	5.00	39.43	25.71	0.00
5.55	0.00	0.33	0.09	0.00	4.60	3.00	4.08
47.57	32.14	2.79	4.94	0.33	4.60	6.00	8.17
23.79	0.99	0.00	0.05	0.17	0.00	12.86	4.08
0.00	1.50	0.00	0.00	0.71	0.00	0.00	0.00
47.57	12.86	0.65	1.15	0.50	39.43	0.74	1.01
23.79	19.29	4.18	2.47	0.71	19.71	0.00	8.17
71.36	19.29	1.63	4.94	4.29	19.71	0.00	1.34
118.93	19.29	6.96	4.94	0.33	23.00	6.00	8.17
93.56	57.86	7.86	13.90	2.25	216.86	25.71	8.17
0.00	1.50	1.63	0.09	0.71	9.20	3.00	4.08
11.10	3.00	0.53	0.62	0.10	4.60	12.86	0.00
5.55	0.00	2.79	0.58	0.00	9.20	7.50	0.00
0.00	5.57	0.58	0.00	0.00	6.00	0.67	42.86
47.57	12.86	2.79	0.00	0.00	0.00	0.00	0.00
5.55	3.00	0.27	4.94	0.27	59.14	3.00	0.00
0.00	6.43	2.79	0.58	0.00	9.20	7.50	0.00
23.79	0.12	0.00	0.00	0.00	19.71	0.00	0.00
23.79	6.43	1.39	0.00	0.00	19.71	0.00	0.00

1.82	0.49	0.08	0.14	0.00	3.02	0.99	0.00
23.79	6.43	0.19	1.15	0.00	19.71	4.50	52.50
1.50	5.57	0.58	0.71	19.71	3.00	0.34	42.86
0.00	3.00	0.65	0.00	0.71	9.20	12.86	0.00
71.36	3.00	0.00	0.00	0.00	39.43	0.00	0.00
23.79	0.12	0.00	0.00	0.00	19.71	0.00	0.00
11.40	2.47	1.34	1.42	0.83	9.45	4.93	5.03
23.79	6.43	0.00	1.15	0.33	39.43	3.00	1.68
11.10	1.50	0.00	1.42	0.08	19.71	12.86	0.00
0.00	6.43	0.00	0.00	0.71	0.00	0.00	0.00
0.00	1.50	0.00	0.09	0.71	9.20	3.00	0.00
5.55	0.75	0.13	1.15	0.03	2.30	3.00	0.34
5.55	0.00	1.39	0.58	0.00	9.20	6.00	0.67
0.00	1.50	0.00	0.00	0.71	98.57	6.00	0.67
47.57	12.86	0.65	1.73	0.00	59.14	15.00	4.08
5.55	1.62	0.16	0.09	0.00	3.02	1.48	0.00
71.36	6.00	0.00	0.00	0.00	19.71	0.00	0.00
11.10	1.50	0.13	0.33	0.10	4.60	3.00	0.00
0.00	1.50	13.93	0.00	0.71	98.57	6.00	0.67
11.10	6.43	0.00	4.94	0.50	59.14	0.00	17.50
1.50	13.93	0.00	0.71	98.57	6.00	0.67	42.86
5.55	0.00	0.33	0.00	0.00	4.60	3.00	4.08
2.74	3.00	0.16	0.58	4.29	9.20	6.00	0.00
5.55	0.74	0.27	0.38	0.08	9.20	3.00	1.34
0.00	1.50	0.00	0.00	0.71	0.00	0.00	0.00
11.10	0.99	0.21	0.00	0.14	9.20	2.47	4.08
2.78	0.75	0.13	1.15	0.03	2.30	3.00	0.67
0.91	0.37	0.00	2.47	0.14	59.14	25.71	17.50
47.57	2.25	0.00	0.00	0.00	9.20	38.57	0.00
47.57	12.86	0.27	0.38	0.33	1.51	25.71	1.01
47.57	12.86	0.33	0.00	0.05	59.14	25.71	1.68

porteghal	khormaloo	narengi	anar	khorma	aloo	tootfarangi	moz
64.29	1.21	5.60	6.48	1.62	5.36	0.44	41.79
42.86	4.90	0.00	55.51	8.08	6.25	0.95	3.25
0.00	0.00	0.00	0.00	3.23	0.00	0.00	0.00
42.86	4.90	0.00	55.51	8.08	2.50	1.89	0.00
10.00	1.61	2.80	2.66	0.12	0.62	0.11	1.34
21.43	1.61	5.60	0.00	33.93	1.23	1.11	13.93
42.86	9.80	0.00	0.00	8.08	2.50	1.89	0.00
32.14	4.90	12.00	27.76	3.23	0.00	0.00	55.71
4.90	0.00	55.51	8.08	2.50	1.89	0.00	0.00
21.43	9.80	12.00	0.00	4.85	0.00	0.00	13.93
0.00	12.00	0.00	1.62	1.25	0.22	13.93	3.50
64.29	2.01	36.00	2.13	11.31	10.71	0.11	41.79
4.90	5.60	6.48	2.64	2.50	2.43	3.25	7.00
64.29	1.61	36.00	19.43	0.06	2.50	0.04	6.50
10.00	4.90	2.80	12.95	0.75	0.00	0.22	9.75
42.86	4.90	0.00	0.00	8.08	2.50	1.89	0.00
5.00	0.40	0.46	12.95	1.62	1.25	0.04	3.25
21.43	4.90	12.00	6.48	1.62	1.25	0.04	27.86
21.43	14.70	12.00	12.95	1.62	3.75	0.02	6.50
10.00	2.01	0.46	12.95	8.08	1.25	0.04	3.25
64.29	0.00	0.00	0.00	4.04	1.25	0.33	0.00
107.14	4.90	12.00	138.79	3.23	0.00	0.00	13.93
64.29	21.00	24.00	27.76	11.31	10.71	0.95	6.50
64.29	14.70	36.00	3.73	22.62	10.71	0.44	1.60
42.86	4.90	0.00	55.51	8.08	6.25	0.95	3.25
10.00	0.00	12.00	0.00	9.69	5.36	0.00	27.86
21.43	42.00	12.00	55.51	11.31	1.25	2.84	41.79
64.29	2.01	36.00	83.27	11.31	0.62	0.09	0.00
10.00	2.01	0.46	12.95	8.08	1.25	0.04	3.25
5.00	0.00	36.00	6.48	12.93	5.36	0.22	6.96
5.00	0.00	12.00	6.48	16.16	5.36	0.11	20.89
21.43	0.00	60.00	0.00	1.62	0.00	0.33	34.82
42.86	9.80	0.00	0.00	8.08	2.50	1.89	0.00
64.29	63.00	36.00	12.95	11.31	5.36	0.44	41.79
0.81	60.00	6.48	3.23	1.25	0.07	6.50	3.50
9.80	0.00	27.76	3.23	6.25	3.79	3.25	3.50
10.00	9.80	2.80	2.13	45.24	2.50	0.02	1.07
64.29	2.01	36.00	55.51	22.62	10.71	0.09	1.60
21.43	0.00	0.00	0.00	4.85	0.00	0.00	0.00
4.93	14.10	1.38	0.53	0.31	0.31	0.18	2.67
10.00	9.80	2.80	12.95	0.75	0.00	0.22	3.25
21.43	0.00	30.00	0.00	1.62	0.00	0.33	34.82
21.43	0.00	12.00	6.48	6.46	0.00	0.95	6.50
10.00	2.42	5.60	12.95	0.00	1.25	0.11	6.50
64.29	42.00	24.00	55.51	22.62	16.07	2.84	6.50
21.43	3.22	12.00	6.48	0.38	0.41	0.00	3.25
64.29	42.00	36.00	27.76	4.85	10.71	0.95	3.25
0.00	24.00	6.48	8.08	5.36	0.11	20.89	1.75
64.29	9.80	36.00	55.51	11.31	1.25	1.11	0.00
120.82	46.03	47.91	33.93	5.00	0.11	27.86	34.52
64.29	9.80	5.60	27.76	1.62	2.50	0.11	6.50
42.86	4.90	0.00	55.51	8.08	2.50	1.89	0.00

35.00	0.81	5.60	3.19	0.00	0.62	0.11	1.60
85.71	0.00	0.00	0.00	1.62	1.25	0.22	0.00
21.43	0.40	2.80	0.00	4.85	0.62	0.00	13.93
4.90	0.00	27.76	3.23	6.25	4.74	3.25	3.50
12.25	2.80	51.81	0.75	0.00	0.22	6.50	0.00
64.29	0.00	24.00	27.76	22.62	5.36	0.22	69.64
4.90	0.00	55.51	8.08	2.50	1.89	0.00	0.00
5.00	4.90	2.80	12.95	0.75	0.00	0.44	3.25
4.11	0.81	0.46	0.53	0.22	1.23	0.18	3.21
4.03	12.00	27.76	3.23	0.00	0.00	27.86	3.50
9.80	0.00	55.51	1.62	1.25	3.79	3.25	3.50
42.86	4.90	0.00	27.76	3.23	6.25	4.74	3.25
10.00	0.00	1.38	1.06	11.31	0.82	0.11	0.00
42.86	0.81	72.00	12.95	3.23	2.50	2.37	9.75
15.00	3.22	5.60	6.48	4.85	1.25	0.22	1.60
4.90	5.60	6.48	2.64	2.50	2.43	3.25	7.00
0.00	2.80	55.51	3.23	2.50	0.95	3.25	3.50
64.29	2.01	36.00	1.60	0.19	1.25	0.07	6.50
4.93	14.10	1.38	0.53	0.31	0.31	0.18	2.67
21.43	4.90	36.00	55.51	16.16	0.00	0.44	20.89
24.50	84.00	12.95	7.54	62.50	0.18	69.64	0.58
4.03	24.00	32.38	8.08	1.25	0.04	16.25	3.50
0.00	0.00	1.38	0.00	11.31	2.50	0.00	0.00
42.86	4.03	24.00	19.43	11.31	5.36	0.44	27.86
0.00	12.00	0.00	19.39	0.00	0.00	6.96	3.50
107.14	0.00	0.00	0.00	4.85	1.25	0.22	0.00
10.00	0.40	5.60	3.19	3.23	1.25	0.13	13.93
64.29	1.21	2.80	3.73	0.75	0.00	0.15	1.60
4.90	0.00	0.00	3.23	0.00	0.00	0.00	0.00
42.86	0.81	72.00	12.95	3.23	2.50	2.37	9.75
0.81	60.00	6.48	3.23	1.25	1.89	9.75	7.00
21.43	0.00	60.00	0.53	1.62	0.00	0.33	34.82
107.14	4.90	12.00	27.76	9.69	0.00	0.00	13.93
64.29	9.80	24.00	12.95	8.08	5.36	0.44	69.64
4.90	0.00	55.51	8.08	6.25	0.95	0.00	0.00
4.11	0.81	2.80	12.95	11.31	0.82	0.15	13.93
5.00	0.00	2.80	12.95	45.24	1.25	0.18	41.79
64.29	4.90	36.00	0.00	0.00	16.07	0.05	27.86
5.00	42.00	0.00	0.00	4.85	0.00	0.00	0.00
5.00	0.00	24.00	6.48	9.69	10.71	0.22	20.89
0.81	12.00	6.48	3.23	10.71	0.44	13.93	3.50
4.90	0.00	55.51	8.08	6.25	0.95	0.00	0.00
64.29	1.61	36.00	12.95	2.42	1.25	4.42	1.60
8.22	2.01	6.90	1.60	1.55	3.08	0.36	13.36
10.00	4.03	24.00	32.38	3.23	1.25	0.04	6.50
64.29	1.21	36.00	2.13	8.08	0.62	0.15	6.50
85.71	0.00	0.00	0.00	1.62	1.25	0.22	0.00
64.29	63.00	48.00	0.00	33.93	10.71	0.00	13.93
107.14	4.90	36.00	55.51	8.08	2.50	0.33	20.89
10.00	14.70	2.80	12.95	50.90	0.00	0.44	3.25
64.29	9.80	36.00	12.95	3.23	2.50	0.44	3.25
42.86	4.90	0.00	55.51	8.08	6.25	0.95	0.00
0.40	0.46	12.95	1.62	1.25	0.04	3.25	3.50

4.90	2.80	12.95	0.75	0.00	0.22	6.50	0.00
2.01	2.30	26.62	0.31	1.03	0.36	1.34	5.75
10.00	4.03	24.00	32.38	8.08	1.25	0.04	16.25
64.29	9.80	36.00	12.95	11.31	3.75	0.44	41.79
21.43	21.00	12.00	19.43	4.85	5.36	0.18	13.93
0.00	0.00	0.00	6.46	1.25	0.00	6.50	0.00
21.43	14.70	12.00	19.43	0.75	0.21	0.04	0.53
0.00	3.22	0.00	55.51	16.97	0.82	0.04	97.50
64.29	4.90	36.00	9.72	22.62	5.36	2.84	13.93
64.29	0.00	36.00	27.76	33.93	21.43	3.79	97.50
0.00	0.00	24.00	0.00	3.23	0.00	0.00	0.00
15.00	4.90	2.80	6.48	0.75	0.00	0.22	6.50
42.86	42.00	36.00	55.51	22.62	10.71	1.11	97.50
4.90	2.80	6.48	0.75	0.00	0.22	6.50	0.00
5.00	1.61	2.80	2.66	0.31	0.41	0.18	2.14
64.29	4.90	11.20	83.27	11.31	10.71	0.11	1.60
15.00	4.90	2.80	6.48	0.75	0.00	0.22	6.50
21.43	2.01	12.00	12.95	0.75	1.25	0.15	41.79
64.29	9.80	5.60	19.43	22.62	2.50	0.44	13.93
4.90	2.80	6.48	0.75	0.00	0.22	6.50	0.00
10.00	4.03	24.00	32.38	8.08	1.25	0.04	16.25
30.00	7.35	16.80	38.86	11.31	2.50	0.22	97.50
42.86	14.70	12.00	27.76	11.31	37.50	0.11	13.93
10.00	0.00	12.00	6.48	16.16	5.36	0.11	20.89
42.86	0.00	2.80	0.53	3.23	5.36	0.02	0.27
21.43	3.22	5.60	12.95	8.08	2.50	3.79	27.86
42.86	4.90	0.00	55.51	8.08	6.25	0.95	0.00
21.43	21.00	12.00	27.76	0.57	5.36	0.44	1.34
42.86	9.80	24.00	27.76	1.62	1.25	0.18	27.86
42.86	0.00	12.00	12.95	1.62	0.00	1.89	6.50
64.29	21.00	36.00	0.00	33.93	10.71	0.00	6.50
2.01	2.30	26.62	0.31	1.03	0.36	1.34	5.75
1.23	4.03	5.75	1.06	0.19	12.50	0.18	27.86
10.00	4.90	5.60	6.48	2.64	2.50	2.43	3.25
64.29	4.90	36.00	55.51	6.46	10.71	0.66	41.79
42.86	4.03	48.00	27.76	3.23	0.00	0.00	27.86
107.14	0.81	60.00	6.48	3.23	1.25	2.84	9.75
21.43	4.03	12.00	27.76	3.23	0.00	0.00	27.86
64.29	4.90	36.00	6.48	12.12	2.50	0.44	34.82
110.37	6.04	13.81	7.98	11.31	1.03	0.36	41.79
64.29	63.00	48.00	27.76	8.08	16.07	1.89	13.93
5.00	0.00	12.00	6.48	16.16	5.36	0.11	20.89
64.29	9.80	36.00	19.43	11.31	10.71	0.66	27.86
10.00	1.61	5.60	2.66	22.62	0.41	0.11	69.64
42.86	2.82	24.00	0.00	0.38	1.25	0.22	2.67
64.29	4.90	36.00	6.48	11.31	5.36	0.44	0.00
0.00	0.00	0.00	3.23	1.25	0.00	6.50	0.00
42.86	21.00	12.00	27.76	11.31	16.07	0.22	27.86
24.66	8.05	4.60	15.97	24.24	48.21	0.27	22.75
5.00	21.00	12.00	3.19	8.08	0.82	0.22	9.75
64.29	0.00	0.00	0.00	4.04	1.25	0.22	0.00
42.86	4.90	0.00	27.76	3.23	6.25	4.74	3.25
42.86	9.80	0.00	55.51	1.62	1.25	3.79	3.25

5.00	1.61	1.38	3.19	11.31	0.72	0.15	27.86
64.29	31.50	36.00	83.27	8.08	8.04	0.11	13.93
32.14	4.90	18.00	12.95	33.93	1.25	0.22	0.00
4.90	0.00	0.00	3.23	0.00	0.00	0.00	0.00
10.00	4.90	5.60	6.48	2.64	2.50	2.43	3.25
64.29	9.80	0.00	0.00	8.08	0.00	1.89	0.00
10.00	4.90	5.60	6.48	0.00	1.25	0.00	6.50
2.47	0.81	1.38	2.13	0.38	0.41	0.11	1.60
10.00	0.00	12.00	0.00	9.69	5.36	0.00	27.86
64.29	9.80	36.00	55.51	11.31	1.54	0.27	13.93
0.00	12.00	0.00	19.39	0.00	0.00	6.96	3.50
0.40	0.23	6.48	8.08	1.25	0.09	3.25	3.50
9.80	0.00	55.51	3.23	1.25	0.95	3.25	3.50
42.86	9.80	0.00	55.51	1.62	1.25	0.95	3.25
5.00	0.40	0.46	12.95	1.62	1.25	0.04	3.25
64.29	42.00	36.00	27.76	11.31	16.07	0.66	6.50
64.29	14.70	36.00	6.48	3.23	10.71	0.22	27.86
0.00	11.20	0.00	4.85	2.50	0.95	3.25	3.50
10.00	2.42	5.60	12.95	0.75	1.25	0.22	1.60
150.00	96.00	84.00	38.86	29.03	13.73	0.55	65.46
21.43	14.70	24.00	27.76	33.93	0.82	0.27	27.86
12.25	2.80	51.81	0.75	0.00	0.22	6.50	0.00
0.00	0.00	0.00	0.00	3.23	0.00	0.00	0.00
4.03	24.00	32.38	3.23	1.25	0.04	6.50	17.50
10.00	4.90	2.80	12.95	0.75	0.00	0.22	9.75
64.29	63.00	36.00	83.27	11.31	32.14	3.79	0.00
2.01	0.46	12.95	8.08	1.25	0.04	3.25	3.50
42.86	42.00	24.00	6.48	11.31	5.36	1.11	27.86
0.00	0.00	0.00	1.62	1.25	0.22	0.00	0.00
32.14	1.61	18.00	6.48	4.85	5.36	0.22	6.50
10.00	3.22	5.60	6.48	16.97	0.00	0.36	9.75
10.00	2.01	0.46	12.95	8.08	1.25	0.04	3.25
10.00	0.00	12.00	6.48	9.69	5.36	0.11	20.89
2.01	0.46	12.95	8.08	1.25	0.04	3.25	3.50
5.00	0.00	12.00	6.48	16.16	5.36	0.11	20.89
0.00	5.60	0.00	4.85	2.50	0.95	3.25	3.50
64.29	4.90	36.00	6.48	0.37	0.62	0.11	1.60
64.29	0.81	24.00	27.76	11.31	5.36	0.05	0.00
64.29	42.00	24.00	6.48	11.31	10.71	0.11	41.79
42.86	0.81	72.00	6.48	3.23	1.25	1.89	9.75
0.00	12.00	0.00	8.08	2.68	0.00	34.82	5.25
4.90	0.00	55.51	8.08	2.50	0.95	0.00	0.00
64.29	0.00	36.00	6.48	6.46	10.71	0.18	13.00
0.00	24.00	6.48	9.69	0.00	0.00	27.86	3.50
10.00	0.81	24.00	12.95	3.23	10.71	0.44	27.86
42.86	4.90	0.00	55.51	8.08	2.50	1.89	0.00
21.43	4.90	12.00	6.48	3.23	5.36	0.00	13.93
110.37	6.04	13.81	7.98	11.31	1.03	0.36	41.79
21.43	0.00	12.00	12.95	1.62	0.00	0.95	6.50
64.29	9.80	36.00	6.48	1.62	16.07	0.44	6.50
64.29	0.00	60.00	55.51	0.00	16.07	0.73	97.50
64.29	1.61	36.00	1.60	4.85	5.36	0.09	97.50
0.00	12.00	0.00	19.39	0.00	0.00	6.96	3.50

0.00	0.81	4.14	12.95	1.13	0.00	0.18	1.34
64.29	21.00	2.07	27.76	0.75	10.71	0.22	41.79
64.29	42.00	36.00	55.51	22.62	10.71	1.89	41.79
4.90	2.80	12.95	0.75	0.00	0.44	3.25	0.00
64.29	21.00	1.38	6.48	11.31	1.25	0.22	6.50
0.40	24.00	0.00	1.62	0.00	0.95	16.25	3.50
0.00	0.00	12.00	0.00	1.62	1.25	0.22	13.93
64.29	2.42	36.00	27.76	11.31	1.25	0.22	13.93
2.47	3.22	1.15	1.06	8.08	1.23	0.22	13.93
0.00	12.00	0.00	9.69	0.00	0.00	13.93	3.50
4.90	36.00	55.51	16.16	0.00	0.00	20.89	3.50
10.00	0.81	1.15	12.95	8.08	1.25	0.04	3.25
5.00	2.01	0.92	2.13	11.31	1.03	0.22	41.79
64.29	4.90	0.00	12.95	11.31	0.82	0.04	6.50
42.86	4.90	0.00	27.76	3.23	6.25	0.95	3.25
15.00	4.90	5.60	2.66	0.19	1.25	0.09	6.50
5.00	4.90	2.80	12.95	0.75	0.00	0.44	3.25
10.00	4.90	5.60	6.48	2.64	2.50	2.43	3.25
64.29	9.80	36.00	19.43	11.31	10.71	0.66	27.86
10.00	0.81	5.60	12.95	11.31	0.15	0.03	20.89
42.86	4.90	0.00	55.51	8.08	2.50	1.89	0.00
21.43	21.00	12.00	12.95	11.31	5.36	0.11	27.86
32.88	4.03	4.60	10.65	0.93	9.25	0.18	3.25
10.00	4.90	5.60	6.48	1.62	1.25	0.05	13.93
64.29	9.80	0.00	0.00	8.08	0.00	1.89	0.00
10.00	9.80	2.80	12.95	0.75	0.00	0.22	3.25
42.86	0.00	2.80	0.00	1.62	2.50	0.95	3.25
0.00	12.00	6.48	3.23	0.00	0.00	13.93	3.50
5.00	4.90	2.80	12.95	0.75	0.00	0.44	3.25
5.00	42.00	0.00	0.00	4.85	0.00	4.74	3.25
5.00	1.61	2.80	2.13	11.31	1.23	0.18	13.93
64.29	0.00	5.60	0.00	4.85	2.50	0.95	3.25
5.00	1.21	2.80	1.60	0.38	3.75	0.00	2.67
42.86	9.80	0.00	27.76	3.23	6.25	3.79	3.25
64.29	21.00	36.00	55.51	11.31	10.71	1.89	55.71
10.00	0.00	12.00	0.00	19.39	0.00	0.00	13.93
107.14	0.81	60.00	6.48	3.23	1.25	2.84	9.75
64.29	9.80	36.00	6.48	33.93	5.36	0.27	6.50
0.00	0.00	0.00	0.00	3.23	0.00	0.00	0.00
0.00	1.61	0.00	2.66	0.75	2.50	0.15	41.79
64.29	21.00	12.00	27.76	11.31	5.36	0.95	13.93
150.00	42.00	84.00	55.51	11.31	10.71	1.89	97.50
42.86	0.81	72.00	12.95	3.23	2.50	3.32	6.50
157.14	90.00	100.00	64.77	57.30	24.96	0.99	101.18
5.00	0.00	24.00	6.48	9.69	10.71	0.22	20.89
64.29	4.03	12.00	6.48	33.93	0.82	0.18	2.67
42.86	0.00	2.80	55.51	3.23	2.50	0.95	3.25
9.80	0.00	55.51	1.62	1.25	3.79	3.25	3.50
64.29	9.80	36.00	55.51	16.97	10.71	0.00	41.79
64.29	42.00	36.00	12.95	11.31	2.50	0.36	1.07
64.29	0.00	5.60	0.00	4.85	2.50	0.95	3.25
5.00	4.90	2.80	12.95	0.75	0.00	0.44	3.25
5.00	0.81	12.00	3.19	11.31	2.50	0.00	0.00

64.29	1.61	1.38	0.00	0.38	0.41	0.02	13.00
42.86	42.00	12.00	27.76	33.93	10.71	1.89	27.86
9.80	0.00	27.76	3.23	6.25	9.47	3.25	3.50
42.86	0.40	24.00	0.00	1.62	0.00	0.95	6.50
10.00	4.90	2.80	12.95	0.75	0.00	0.22	6.50
5.00	4.90	2.80	12.95	0.75	0.00	0.44	3.25
24.66	8.05	4.60	15.97	24.24	48.21	0.27	22.75
10.00	1.21	5.60	2.13	4.85	2.50	0.22	6.50
21.43	21.00	0.00	41.64	11.31	2.50	0.00	3.25
0.00	0.00	0.00	0.00	3.23	0.00	0.00	0.00
5.00	0.00	12.00	6.48	16.16	5.36	0.11	20.89
10.00	0.40	0.46	12.95	8.08	1.25	0.04	3.25
42.86	9.80	0.00	55.51	3.23	1.25	0.95	3.25
42.86	4.90	0.00	0.00	8.08	2.50	1.89	0.00
64.29	42.00	36.00	6.48	11.31	10.71	2.84	27.86
32.14	9.80	5.60	2.66	0.38	2.50	0.02	9.75
20.00	4.90	2.80	12.95	0.75	0.00	0.22	6.50
10.00	4.90	5.60	6.48	2.64	2.50	2.43	3.25
42.86	4.90	0.00	55.51	8.08	2.50	1.89	0.00
64.29	63.00	36.00	83.27	11.31	16.07	0.22	27.86
4.90	0.00	55.51	8.08	2.50	1.89	0.00	0.00
10.00	0.00	12.00	0.00	1.62	1.25	0.22	13.93
10.00	2.42	5.60	3.19	22.62	1.25	0.11	27.86
5.00	2.82	1.61	4.26	6.46	0.72	0.09	6.50
0.00	0.00	0.00	0.00	3.23	0.00	0.00	0.00
64.29	4.90	36.00	6.48	3.23	2.50	1.55	13.93
10.00	0.40	0.46	12.95	1.62	1.25	0.04	3.25
10.00	1.21	12.00	27.76	1.62	0.41	0.18	0.00
32.14	42.00	18.00	27.76	22.62	1.25	0.00	13.93
64.29	21.00	36.00	4.26	22.62	5.36	0.11	6.50
64.29	19.60	36.00	6.48	22.62	10.71	0.00	13.93

limooshirin	limootorsh	abporteghal	absib	abtalebi	keshmesh	tooteraze	tootekhosh
45.00	0.35	3.78	3.78	0.00	0.20	0.95	0.01
0.00	13.58	7.67	7.67	16.67	8.57	16.57	0.24
0.00	10.56	0.00	0.00	0.00	3.43	0.00	0.00
0.00	13.58	7.67	7.67	16.67	8.57	0.00	0.24
0.86	6.03	0.00	0.00	0.00	0.20	0.32	0.00
7.00	12.07	0.00	0.00	0.00	5.14	8.29	0.12
0.00	13.58	38.33	0.00	16.67	8.57	0.00	0.24
15.00	0.29	0.00	0.00	0.00	0.00	0.00	0.00
13.58	7.67	7.67	16.67	8.57	0.00	0.24	1.00
0.00	6.03	0.00	0.00	0.00	12.00	0.00	0.00
10.56	0.63	0.00	0.00	8.00	8.29	0.00	0.16
45.00	6.03	2.52	2.52	0.00	0.40	8.29	0.02
0.70	0.63	0.63	0.68	0.40	0.32	0.00	0.12
45.00	1.41	0.00	0.00	0.00	0.00	0.32	0.00
0.00	10.56	0.63	0.00	0.00	1.71	0.00	0.00
0.00	13.58	38.33	7.67	16.67	8.57	0.00	0.24
3.50	10.73	0.00	0.00	0.00	3.43	0.00	0.03
15.00	0.29	0.00	0.00	0.00	5.14	0.16	0.36
15.00	2.11	0.00	0.00	0.00	0.00	1.93	0.02
3.50	10.73	0.00	0.00	0.00	3.43	0.00	0.01
0.00	12.32	0.00	15.33	0.00	1.71	0.00	0.00
3.50	11.26	0.00	0.00	0.00	17.14	0.00	0.00
30.00	6.03	15.33	0.00	0.00	5.14	2.38	0.00
0.00	0.23	7.67	0.00	0.00	0.80	0.79	0.00
0.00	13.58	7.67	7.67	16.67	8.57	16.57	0.24
3.50	10.56	0.00	0.00	0.00	4.80	0.00	0.00
15.00	0.00	15.33	0.00	0.00	1.71	0.16	0.02
0.00	0.70	0.00	0.00	0.00	1.71	0.79	0.36
3.50	10.73	0.00	0.00	0.00	3.43	0.00	0.01
1.75	10.59	0.00	1.26	0.00	0.80	1.93	0.03
1.75	10.68	0.00	0.00	1.37	4.00	0.00	0.00
5.25	11.62	0.63	0.00	0.00	20.57	99.43	0.00
0.00	13.58	38.33	0.00	16.67	8.57	0.00	0.24
45.00	3.02	0.00	0.00	0.00	5.14	0.64	0.00
0.70	0.63	0.63	0.00	3.43	3.87	0.06	0.20
13.58	0.00	0.00	0.00	8.57	16.57	0.00	0.20
7.00	0.12	0.00	0.00	0.00	0.13	0.00	0.14
3.50	0.35	0.00	0.00	0.00	0.00	1.59	0.00
0.00	10.56	0.00	0.00	0.00	3.43	0.00	0.00
0.00	0.00	0.00	0.00	0.00	0.20	0.16	0.04
0.00	10.56	0.63	0.00	0.00	5.14	0.00	0.00
5.25	11.62	0.63	0.00	0.00	0.00	41.43	0.00
3.50	10.56	0.00	7.67	0.00	0.00	0.00	0.00
7.00	0.00	0.00	0.00	0.00	5.14	0.16	0.61
45.00	6.03	23.00	23.00	8.33	1.71	41.43	0.61
1.44	0.29	0.00	0.00	2.74	0.00	0.48	0.28
45.00	0.00	0.00	0.00	0.00	5.14	1.93	1.70
10.68	0.00	0.63	0.00	4.00	0.00	0.00	0.00
7.00	0.00	0.00	0.00	0.00	12.00	8.29	0.28
0.58	1.26	15.75	61.64	3.43	1.93	0.45	0.00
7.00	0.70	0.00	0.00	0.00	0.80	8.29	0.00
0.00	13.58	7.67	7.67	16.67	8.57	0.00	0.24

1.73	0.00	0.00	0.00	0.00	0.00	0.00	0.00
0.00	10.56	0.00	0.00	0.00	4.29	0.00	0.00
3.50	2.11	0.00	0.00	0.00	12.00	0.64	0.00
13.58	0.00	0.00	25.00	1.71	16.57	0.00	0.20
10.56	0.63	0.00	0.00	5.14	0.00	0.00	0.00
30.00	15.09	0.00	0.00	0.00	1.71	0.00	0.00
13.58	7.67	7.67	16.67	8.57	0.00	0.24	1.00
0.00	10.56	0.95	0.00	0.00	3.43	0.00	0.00
0.00	0.35	1.26	0.00	3.42	0.26	0.95	0.29
11.97	0.00	0.00	41.67	20.57	0.00	0.00	0.10
13.58	0.00	0.00	0.00	0.00	0.00	0.61	0.20
3.50	13.58	0.00	0.00	25.00	1.71	16.57	0.00
0.00	2.11	0.00	0.00	0.00	0.80	0.16	0.00
7.00	1.41	38.33	1.26	178.57	3.43	9.67	0.14
7.00	0.00	0.00	0.00	0.00	0.80	0.00	0.00
0.70	0.63	0.63	0.68	0.40	0.32	0.00	0.12
13.58	0.00	15.33	0.00	0.00	0.00	0.61	0.00
0.00	1.41	3.15	1.89	0.00	0.20	1.27	0.02
0.00	0.00	0.00	0.00	0.00	0.20	0.16	0.04
3.50	11.97	38.33	0.00	41.67	34.29	16.57	0.61
60.34	1.26	0.63	142.86	1.20	15.47	0.00	1.26
10.73	1.26	0.00	0.00	3.43	0.00	0.01	0.02
0.00	0.00	0.00	0.00	0.00	0.00	0.00	0.00
30.00	6.03	0.00	0.00	16.67	0.00	0.16	0.34
10.56	0.32	0.00	0.00	8.00	16.57	0.00	0.08
0.00	10.56	0.00	0.00	0.00	4.29	0.00	0.00
7.00	2.11	0.00	7.67	0.00	0.20	0.00	0.00
3.50	3.02	7.67	1.89	0.00	0.20	0.32	0.02
13.58	0.00	0.00	16.67	8.57	0.00	0.00	0.00
7.00	1.41	38.33	1.26	0.00	20.57	0.00	0.00
1.41	38.33	0.00	71.43	3.43	3.87	0.06	0.10
5.25	11.62	0.63	0.00	0.00	0.00	124.29	0.00
3.50	11.26	0.00	15.33	0.00	0.00	16.57	0.61
30.00	2.11	0.00	0.00	0.00	0.20	0.48	0.01
13.58	7.67	7.67	16.67	8.57	0.00	0.12	0.20
1.44	0.23	0.63	0.63	0.00	0.00	0.79	0.00
3.50	0.00	3.78	0.00	0.00	1.71	0.00	0.00
0.00	0.00	0.00	0.00	0.00	0.10	0.00	0.00
0.00	10.56	0.00	0.00	71.43	0.00	1.93	0.00
3.50	10.68	0.00	0.63	0.00	2.00	3.87	0.06
10.62	0.00	0.63	0.00	0.00	0.00	0.00	0.50
13.58	7.67	7.67	16.67	8.57	0.00	0.12	0.20
45.00	0.58	15.33	0.00	0.00	0.00	38.67	0.00
11.51	0.41	12.60	3.15	1.37	2.00	0.16	0.21
17.50	10.73	1.26	0.00	0.00	3.43	0.00	0.01
45.00	9.05	0.00	0.00	0.00	5.14	0.00	0.02
0.00	10.56	0.00	0.00	0.00	4.29	0.00	0.00
0.00	3.02	0.00	0.00	0.00	0.00	0.00	0.00
3.50	11.26	0.00	0.00	0.00	0.00	16.57	0.61
0.00	10.56	0.00	0.00	0.00	3.43	0.00	0.00
45.00	0.70	15.33	7.67	107.14	0.00	0.00	0.00
0.00	13.58	7.67	7.67	16.67	8.57	0.00	0.12
10.73	0.00	0.00	0.00	3.43	0.00	0.03	0.00

10.56	0.63	0.00	0.00	5.14	0.00	0.00	0.00
4.05	6.30	0.00	6.85	0.00	3.18	0.00	0.00
3.50	10.73	1.26	0.00	0.00	3.43	0.00	0.01
45.00	9.05	0.00	0.00	0.00	0.80	0.00	0.03
15.00	2.11	0.00	0.00	0.00	0.00	0.32	0.00
21.12	0.00	0.00	0.00	6.86	0.00	0.00	0.00
0.58	0.12	1.26	2.52	3.42	0.03	0.00	0.00
0.00	0.46	0.00	0.00	0.00	12.00	0.00	0.36
30.00	0.70	0.00	0.00	0.00	0.80	1.93	0.06
45.00	9.05	65.71	65.71	0.00	1.71	0.48	0.00
0.00	10.56	0.00	0.00	71.43	5.14	0.00	0.00
0.00	10.56	0.00	0.00	0.00	3.43	0.00	0.00
30.00	6.03	0.00	0.00	0.00	0.00	0.00	0.00
10.56	0.00	0.00	0.00	5.14	3.87	0.00	0.43
1.15	0.23	0.00	0.00	0.00	0.80	0.32	0.06
45.00	3.02	1.89	1.89	0.00	12.00	0.95	0.01
0.00	10.56	0.00	0.00	0.00	4.29	0.00	0.00
15.00	12.07	1.26	1.26	0.00	5.14	0.16	0.02
30.00	3.02	0.00	0.00	0.00	0.23	0.16	0.00
10.56	0.00	0.00	0.00	4.29	0.00	0.00	0.43
17.50	10.73	1.26	0.00	0.00	3.43	0.00	0.01
21.00	9.05	32.86	32.86	0.00	12.00	1.93	0.36
15.00	9.05	1.26	0.00	0.00	12.00	1.93	0.06
1.75	10.68	0.00	0.00	0.00	4.80	0.00	0.00
15.00	2.11	0.00	0.00	0.00	1.71	0.16	0.00
7.00	12.07	15.33	0.00	0.00	0.80	0.32	0.36
0.00	13.58	7.67	7.67	16.67	8.57	16.57	0.24
15.00	0.70	0.00	0.95	89.29	4.29	8.29	0.00
7.00	2.11	23.00	0.00	3.42	0.16	0.64	0.12
7.00	10.56	0.00	7.67	1.37	17.14	0.00	0.28
45.00	6.03	0.00	0.00	0.00	17.14	1.59	0.00
4.05	6.30	0.00	6.85	0.00	3.18	0.00	0.00
2.88	1.41	23.00	1.26	4.11	0.40	0.64	0.08
7.00	0.70	0.63	0.63	0.68	0.40	0.32	0.00
45.00	6.03	2.52	2.52	0.00	5.14	3.87	0.09
3.50	11.97	0.00	0.00	41.67	8.57	0.00	0.85
7.00	1.41	38.33	0.00	71.43	3.43	3.87	0.06
3.50	11.97	38.33	0.00	41.67	20.57	0.00	0.00
15.00	9.05	0.00	0.00	0.00	5.14	0.95	0.43
2.88	1.74	32.86	32.86	1.37	0.80	0.48	0.12
45.00	6.03	0.00	0.00	0.00	1.71	0.32	0.73
1.75	10.68	0.00	0.00	1.37	4.00	0.00	0.00
3.50	1.41	1.89	0.00	2.05	5.14	5.80	0.00
3.50	0.70	0.00	0.00	0.00	5.14	0.00	0.00
3.50	0.70	0.00	0.00	0.00	0.16	0.64	0.00
45.00	6.03	32.86	0.00	0.00	0.40	0.00	0.00
10.56	0.00	0.00	0.00	3.43	0.00	0.00	0.00
30.00	6.03	0.00	0.00	0.00	12.00	8.29	0.12
1.44	1.91	1.26	0.00	4.79	1.71	1.27	0.00
1.15	9.05	0.00	0.00	0.00	0.20	1.59	0.00
0.00	11.97	0.00	7.67	0.00	6.86	0.00	0.00
3.50	13.58	0.00	0.00	25.00	8.57	16.57	0.00
3.50	13.58	0.00	0.00	0.00	8.57	16.57	0.00

3.50	0.35	0.00	0.00	0.00	0.80	0.95	0.00
15.00	0.00	0.00	0.00	0.00	0.80	0.00	0.00
22.50	6.03	0.00	0.00	0.00	0.80	0.48	0.49
13.58	0.00	0.00	16.67	8.57	0.00	0.00	0.00
7.00	0.70	0.63	0.63	0.68	0.40	0.32	0.00
0.00	13.58	38.33	0.00	16.67	8.57	0.00	0.24
7.00	0.00	0.00	0.00	0.00	0.40	0.00	0.00
1.73	0.35	3.78	3.78	4.11	0.07	0.16	0.00
3.50	10.56	0.00	0.00	0.00	4.00	41.43	0.00
30.00	9.05	65.71	32.86	0.00	1.71	1.59	0.12
10.56	0.32	0.00	0.00	8.00	16.57	0.00	0.00
0.70	0.00	0.00	0.00	3.43	3.87	0.06	0.20
13.58	0.00	0.00	0.00	0.00	0.00	0.61	0.20
3.50	13.58	0.00	0.00	0.00	0.00	0.00	0.61
3.50	10.73	0.00	0.00	0.00	3.43	0.00	0.03
45.00	6.03	0.00	0.00	0.00	5.14	1.93	0.00
45.00	6.03	0.00	3.78	4.79	0.00	4.77	0.02
13.58	0.00	15.33	0.00	0.00	0.00	0.00	0.00
7.00	9.05	0.00	0.00	0.00	0.20	0.00	0.00
16.29	4.57	15.33	32.86	103.81	16.69	2.49	0.00
45.00	3.02	1.89	0.63	0.00	0.80	0.95	0.09
10.56	0.63	0.00	0.00	5.14	0.00	0.00	0.00
0.00	10.56	0.00	0.00	0.00	3.43	0.00	0.00
10.73	1.26	0.00	0.00	3.43	0.00	0.01	0.02
0.00	10.56	0.63	0.00	0.00	1.71	0.00	0.00
45.00	9.05	0.00	0.00	0.00	6.86	0.32	0.02
10.73	0.00	0.00	0.00	3.43	0.00	0.01	0.02
15.00	9.05	7.67	7.67	0.00	1.71	0.79	0.07
10.56	0.00	0.00	0.00	4.29	0.00	0.00	0.00
3.50	1.41	0.00	0.00	0.00	0.80	0.32	0.12
0.00	0.23	0.00	0.00	0.00	0.00	0.79	0.00
3.50	10.73	0.00	0.00	0.00	3.43	0.00	0.01
1.75	10.68	0.00	0.00	0.00	4.80	0.00	0.00
10.73	0.00	0.00	0.00	3.43	0.00	0.01	0.02
1.75	10.68	0.00	0.63	0.00	4.00	0.00	0.00
13.58	0.00	15.33	0.00	0.00	0.00	0.28	0.00
1.73	1.41	3.78	3.78	0.00	36.00	0.95	0.01
30.00	6.03	0.00	0.00	0.00	0.10	5.80	0.00
7.00	6.03	0.00	0.00	0.00	1.71	5.80	0.28
7.00	1.41	38.33	1.26	178.57	3.43	9.67	0.14
11.62	0.00	0.00	0.00	25.71	82.86	0.00	0.43
13.58	7.67	7.67	16.67	8.57	0.00	0.12	0.20
45.00	0.17	0.00	0.00	0.00	0.00	0.16	0.00
10.56	0.00	7.67	0.00	0.00	0.00	0.00	0.00
1.75	10.59	0.00	1.26	0.00	4.00	19.33	0.00
0.00	13.58	38.33	7.67	16.67	8.57	0.00	0.24
15.00	1.41	1.26	1.26	0.00	4.00	1.93	0.28
2.88	1.74	32.86	32.86	1.37	0.80	0.48	0.12
3.50	10.56	0.00	7.67	0.00	17.14	0.00	0.00
45.00	9.05	0.00	0.00	0.00	12.00	1.93	5.95
45.00	21.12	32.86	0.00	0.00	0.00	1.11	3.40
45.00	0.29	3.78	3.78	4.11	0.16	0.95	0.00
10.56	0.32	0.00	0.00	8.00	16.57	0.00	0.08

7.00	1.41	0.00	0.00	0.00	0.00	0.32	0.19
2.30	6.03	0.00	0.00	0.00	0.80	3.87	0.00
7.00	6.03	32.86	0.00	0.00	3.43	5.80	0.36
10.56	0.95	0.00	0.00	1.71	0.00	0.00	0.00
45.00	2.11	0.63	0.63	0.00	1.20	1.93	0.02
11.26	0.00	0.00	0.00	8.57	0.00	0.14	0.20
3.50	10.56	0.63	0.00	0.00	0.00	8.29	0.00
0.00	3.02	0.00	0.00	0.00	0.00	0.32	0.00
1.44	9.05	0.00	0.00	0.00	1.60	1.59	0.00
10.56	0.63	0.00	0.00	4.00	8.29	0.00	0.08
11.97	38.33	0.00	41.67	34.29	16.57	0.61	0.10
3.50	10.73	0.00	0.00	0.00	3.43	0.00	0.01
3.50	6.03	0.63	0.63	0.00	0.10	0.16	0.01
30.00	3.02	3.15	0.00	0.00	0.03	0.48	0.00
3.50	13.58	7.67	7.67	16.67	1.71	16.57	0.00
7.00	3.02	0.00	0.00	0.00	8.57	0.16	0.00
0.00	10.56	0.00	0.00	0.00	3.43	0.00	0.00
7.00	0.70	0.63	0.63	0.68	0.40	0.32	0.00
3.50	1.41	1.89	0.00	2.05	5.14	5.80	0.00
3.50	0.70	0.00	0.00	0.00	2.57	0.32	0.00
0.00	13.58	38.33	7.67	16.67	8.57	0.00	0.24
15.00	6.03	0.63	0.63	0.68	0.80	0.00	0.01
22.44	1.74	15.33	7.67	4.79	0.80	0.32	0.70
7.00	6.03	0.00	0.00	0.00	3.43	0.64	0.12
0.00	13.58	38.33	0.00	16.67	8.57	0.00	0.24
0.00	10.56	0.63	0.00	0.00	5.14	0.00	0.00
3.50	13.58	0.00	15.33	0.00	0.00	0.00	0.00
10.56	0.00	7.67	0.00	0.00	0.00	0.00	0.00
0.00	10.56	0.95	0.00	0.00	5.14	0.00	0.00
0.00	10.56	0.00	0.00	71.43	0.00	1.93	0.00
3.50	0.29	0.00	0.00	0.00	1.71	0.00	0.12
3.50	13.58	0.00	15.33	0.00	0.00	0.00	0.28
3.50	6.03	1.26	0.00	0.00	0.33	0.00	0.00
3.50	13.58	0.00	0.00	0.00	8.57	16.57	0.00
45.00	0.00	0.00	0.00	0.00	5.14	8.29	0.36
3.50	10.56	0.63	0.00	0.00	8.00	16.57	0.00
7.00	1.41	38.33	0.00	71.43	3.43	3.87	0.06
45.00	0.87	0.00	0.00	0.00	34.00	0.00	0.00
0.00	10.56	0.00	0.00	0.00	3.43	0.00	0.00
45.00	1.41	2.52	0.00	0.00	0.80	0.95	0.00
45.00	0.00	0.00	0.00	8.33	12.00	1.93	0.24
45.00	6.03	7.67	3.78	0.00	0.00	0.00	0.00
7.00	0.70	7.67	1.26	0.00	20.57	0.00	0.00
32.01	7.73	30.67	65.71	107.62	21.37	3.05	0.00
7.00	10.62	0.00	0.00	0.00	2.00	3.87	0.06
45.00	3.02	0.00	0.00	0.00	0.40	0.16	0.00
3.50	13.58	0.00	15.33	0.00	0.00	0.00	0.61
13.58	0.00	0.00	0.00	0.00	0.00	0.61	0.20
0.00	1.41	0.00	0.00	0.00	1.20	0.00	0.00
0.00	6.03	0.00	0.00	0.00	4.29	0.00	0.57
3.50	13.58	0.00	15.33	0.00	0.00	0.00	0.28
0.00	10.56	0.95	0.00	0.00	1.71	0.00	0.00
7.00	3.02	0.00	0.00	0.00	0.00	0.00	0.00

7.00	0.69	0.00	0.00	0.00	0.10	0.00	0.02
7.00	0.00	0.00	0.00	0.00	0.03	3.87	0.00
13.58	0.00	0.00	25.00	8.57	16.57	0.00	0.20
3.50	11.26	0.00	0.00	0.00	8.57	0.00	0.14
0.00	10.56	0.63	0.00	0.00	5.14	0.00	0.00
0.00	10.56	0.95	0.00	0.00	5.14	0.00	0.00
1.44	1.91	1.26	0.00	4.79	1.71	1.27	0.00
7.00	2.11	0.00	0.00	0.00	1.71	0.48	0.00
22.50	3.02	0.00	0.00	0.00	6.86	0.00	0.00
0.00	10.56	0.00	0.00	0.00	3.43	0.00	0.00
1.75	10.68	0.00	0.63	0.00	4.00	0.00	0.00
3.50	10.73	0.00	0.00	0.00	3.43	0.00	0.03
3.50	13.58	0.00	15.33	0.00	0.00	0.00	0.61
0.00	13.58	38.33	0.00	16.67	8.57	0.00	0.24
3.50	9.05	0.00	0.00	0.00	12.00	0.32	0.85
1.73	6.03	0.00	0.00	0.00	0.80	0.16	0.02
0.00	10.56	0.63	0.00	0.00	5.14	0.00	0.00
7.00	0.70	0.63	0.63	0.68	0.40	0.32	0.00
0.00	13.58	7.67	7.67	16.67	8.57	0.00	0.24
1.73	9.05	0.00	0.00	2.05	5.14	1.93	0.36
13.58	7.67	7.67	16.67	8.57	0.00	0.24	1.00
3.50	10.56	0.63	0.00	0.00	4.00	8.29	0.00
7.00	1.41	15.33	0.00	0.00	5.14	0.95	0.00
3.50	2.11	3.78	0.00	0.00	0.80	1.43	0.00
0.00	10.56	0.00	0.00	0.00	3.43	0.00	0.00
15.00	0.12	0.63	0.63	0.00	12.00	0.16	0.02
3.50	10.73	0.00	0.00	0.00	3.43	0.00	0.03
1.44	15.09	0.00	0.00	0.00	0.20	0.00	0.02
45.00	0.29	0.00	0.00	0.00	0.80	8.29	0.00
45.00	1.41	0.00	0.00	0.00	5.14	1.59	0.01
0.00	6.03	0.00	0.00	0.00	17.14	0.16	0.00

bargeha	zeitoonesa	kompoot	abmivebas	roghannab	roghanema	roghaneze	roghanehe
0.43	0.03	7.23	3.29	0.37	6.00	0.12	0.37
0.20	0.57	1.21	0.00	6.43	1.71	0.00	6.43
0.00	0.86	0.00	0.00	16.07	2.57	0.00	0.00
1.00	0.57	1.21	0.00	8.04	2.14	1.71	6.43
0.05	0.13	0.00	0.00	9.64	6.00	0.00	0.00
3.00	0.29	0.00	0.00	0.00	6.00	6.00	0.00
2.00	2.86	1.21	0.00	9.64	3.43	1.71	3.21
0.00	0.00	0.00	0.00	0.00	0.86	1.71	0.75
0.57	1.21	0.00	8.04	4.29	2.57	3.21	0.08
0.00	0.07	0.00	0.00	0.00	1.71	0.00	12.86
0.00	314.29	33.33	22.50	4.29	0.86	8.04	0.50
0.02	0.20	0.00	3.29	0.00	12.00	2.57	0.00
0.47	0.00	0.55	0.00	0.20	0.20	1.50	0.12
0.20	0.13	7.23	3.29	0.25	6.00	0.07	0.00
0.00	0.29	0.00	0.00	45.00	2.57	6.00	3.21
1.00	0.57	1.21	0.00	6.43	2.57	0.86	6.43
0.00	0.13	29.33	20.00	45.00	4.71	0.03	1.61
0.03	0.00	0.00	0.00	0.00	6.00	1.71	1.50
0.00	0.13	0.00	1.64	6.43	1.71	0.00	0.00
0.02	0.13	14.67	13.33	33.75	0.86	0.03	16.07
0.00	0.29	0.00	0.00	16.07	3.43	0.00	0.00
0.00	0.03	125.71	13.33	22.50	6.00	0.00	12.86
0.08	0.29	0.00	0.00	0.00	6.00	6.00	0.00
0.00	0.00	0.00	0.00	0.00	12.00	0.00	0.31
0.20	0.57	1.21	0.00	6.43	1.71	0.00	6.43
0.00	0.00	125.71	20.00	0.00	12.00	2.57	9.64
2.57	0.29	0.00	0.00	0.00	6.00	12.00	0.00
0.05	0.03	0.00	0.00	0.00	2.57	6.00	9.64
0.02	0.13	14.67	20.00	56.25	0.43	0.00	14.46
0.10	0.13	188.57	26.67	0.00	6.00	2.57	16.07
0.43	0.29	125.71	20.00	0.00	6.00	0.86	14.46
0.86	0.00	125.71	6.67	22.50	6.00	1.29	8.04
2.00	2.86	1.21	0.00	9.64	3.43	1.71	3.21
0.00	0.20	0.00	0.00	6.43	6.00	0.40	0.00
0.86	0.00	33.33	0.75	3.43	5.14	0.75	10.71
0.57	1.21	0.00	6.43	3.43	0.00	3.21	0.04
0.08	0.01	0.00	6.67	3.21	6.00	0.03	0.00
0.02	0.03	7.23	3.29	0.37	6.00	0.10	0.37
0.00	0.86	0.00	0.00	12.86	2.57	0.00	0.00
0.08	0.16	0.00	13.33	0.12	9.43	0.20	0.25
0.00	0.57	0.00	0.00	67.50	6.00	6.00	3.21
0.16	0.00	188.57	20.00	22.50	4.29	2.14	4.82
0.00	0.00	125.71	53.33	22.50	0.86	0.20	16.07
0.08	0.20	0.00	6.67	12.86	6.00	0.00	0.00
0.50	2.00	0.00	28.57	0.00	6.00	6.00	0.00
0.50	0.00	14.67	6.67	0.00	6.00	0.20	0.00
1.29	0.00	0.00	0.00	0.00	3.43	6.00	0.75
0.13	125.71	26.67	0.00	6.00	0.86	14.46	2.00
0.86	0.33	0.00	0.00	0.00	6.00	0.00	0.75
8.00	1.21	28.57	0.37	18.00	0.60	0.00	0.50
0.02	0.67	0.00	0.00	0.00	6.00	0.00	0.00
1.00	0.57	1.21	0.00	8.04	2.14	1.71	6.43

0.20	0.03	0.00	0.00	0.00	2.57	0.10	3.21
0.00	1.00	0.00	0.00	12.86	3.43	0.00	0.00
0.00	0.00	0.00	0.00	22.50	2.57	0.00	0.00
0.57	1.21	0.00	6.43	3.00	0.00	3.21	0.04
0.57	0.00	0.00	67.50	6.00	6.00	3.21	0.00
0.05	0.57	29.33	0.00	0.00	6.00	12.00	0.00
0.57	1.21	0.00	8.04	2.14	1.71	6.43	0.08
0.00	0.86	0.00	0.00	45.00	6.00	6.00	3.21
0.00	0.02	0.00	6.67	22.50	18.00	0.00	0.00
0.05	62.86	6.67	22.50	1.71	0.86	3.21	8.57
0.57	0.00	0.00	6.43	3.43	0.00	3.21	0.04
0.20	0.57	1.21	0.00	6.43	3.00	0.00	3.21
0.00	0.00	0.00	0.00	0.00	6.00	0.00	0.00
0.20	2.86	1.21	6.67	0.75	1.71	12.00	0.06
0.00	0.03	0.00	0.00	22.50	0.00	0.00	0.00
0.47	0.00	0.55	0.00	0.20	0.20	1.50	0.12
0.57	0.00	0.00	6.43	2.57	1.71	0.00	0.04
0.05	0.13	7.23	3.29	3.21	12.00	0.10	0.00
0.08	0.16	0.00	13.33	0.12	9.43	0.20	0.25
0.10	0.05	125.71	13.33	22.50	2.00	1.71	6.43
2.00	0.00	0.00	0.00	42.00	12.86	6.00	1.00
1.33	14.67	20.00	3.21	5.14	1.71	0.75	1.00
0.00	0.00	0.00	0.00	0.00	6.00	0.00	0.00
0.35	0.33	0.00	0.00	0.00	6.00	0.20	0.00
0.00	188.57	20.00	0.00	2.57	1.71	12.86	3.00
0.00	1.00	0.00	0.00	12.86	3.43	0.00	0.00
0.30	0.07	0.00	0.00	0.00	6.00	2.57	0.00
0.20	0.10	3.62	13.33	9.64	12.00	0.10	0.37
2.86	0.00	0.00	9.64	2.57	4.29	0.75	0.00
1.00	2.86	1.21	6.67	2.25	2.57	12.00	0.06
2.86	1.21	0.00	1.50	4.29	4.71	0.00	10.71
0.00	0.00	125.71	33.33	22.50	1.71	1.29	8.04
0.10	0.03	125.71	13.33	22.50	6.00	1.71	0.00
0.43	0.86	0.00	60.00	0.00	6.00	0.86	0.00
0.57	1.21	0.00	6.43	4.29	4.29	3.21	0.04
0.00	0.00	0.00	0.00	0.00	6.00	0.86	0.00
0.00	0.13	7.23	3.29	0.00	2.57	0.86	0.00
0.02	0.00	2.41	0.00	22.50	0.00	0.00	0.12
0.00	1.43	0.00	0.00	22.50	4.29	0.00	3.21
0.00	0.13	125.71	26.67	0.00	6.00	0.86	16.07
0.13	125.71	33.33	45.00	6.00	0.86	6.43	1.00
0.57	1.21	0.00	6.43	4.29	4.29	3.21	0.08
0.30	0.67	0.00	2.19	0.00	12.00	0.20	0.18
0.16	0.11	12.05	2.74	0.62	6.00	1.00	0.00
0.02	1.33	14.67	20.00	1.61	9.00	4.29	0.75
0.00	0.03	7.23	0.00	22.50	0.40	0.00	0.00
0.00	1.00	0.00	0.00	12.86	3.43	0.00	0.00
0.00	0.00	0.00	0.00	0.00	6.00	0.00	0.00
0.10	0.03	125.71	13.33	33.75	6.00	1.29	0.00
0.00	0.86	0.00	0.00	9.64	2.57	0.00	0.00
0.00	0.33	0.00	0.00	0.00	6.00	0.00	22.50
0.20	0.57	1.21	0.00	6.43	4.29	4.29	3.21
0.13	29.33	20.00	22.50	4.71	0.03	1.61	2.00

1.14	0.00	0.00	45.00	18.00	6.00	3.21	0.00
0.33	0.00	5.48	0.00	18.00	0.16	0.00	10.71
0.02	1.33	14.67	20.00	3.21	5.14	1.71	0.75
0.00	2.00	0.00	0.00	0.00	6.00	0.86	0.00
0.02	0.20	0.00	0.00	6.43	6.00	1.71	0.00
1.14	0.00	0.00	25.71	4.29	0.00	0.00	0.00
0.00	1.14	0.00	0.00	22.50	1.71	0.20	0.00
0.00	0.00	0.00	1.10	0.00	2.57	0.20	0.12
0.05	0.86	0.00	0.00	0.00	12.00	6.00	0.00
0.00	0.13	2.41	6.67	0.00	12.00	6.00	0.00
0.00	1.43	73.33	0.00	16.07	12.00	1.71	0.00
0.43	0.29	0.00	0.00	67.50	6.00	6.00	4.82
0.30	0.29	0.00	0.00	0.00	6.00	0.86	0.00
0.29	0.00	0.00	45.00	12.00	6.00	4.82	0.00
0.00	0.57	0.00	0.00	0.31	6.00	0.40	1.50
1.29	6.00	7.23	3.29	0.18	0.10	0.86	9.64
0.43	0.29	0.00	0.00	67.50	12.00	6.00	4.82
0.00	0.03	0.00	0.00	0.25	12.00	2.57	0.00
0.10	0.23	0.00	0.00	6.43	6.00	1.71	3.21
0.29	0.00	0.00	45.00	12.00	6.00	4.82	0.00
0.02	1.33	14.67	20.00	4.82	6.00	4.29	0.75
0.20	0.05	0.00	13.33	0.00	6.00	6.00	0.00
0.43	0.86	0.00	0.00	0.00	6.00	0.00	0.25
0.43	0.29	125.71	20.00	0.00	6.00	0.86	14.46
0.00	0.13	0.00	0.00	22.50	0.86	0.03	6.43
1.29	2.00	0.00	0.00	0.00	6.00	2.57	0.00
0.20	0.57	1.21	0.00	6.43	4.29	4.29	3.21
0.08	2.00	0.00	0.00	0.00	6.00	2.14	0.00
0.43	0.13	0.00	13.33	0.00	6.00	0.40	0.00
0.00	2.00	125.71	13.33	45.00	0.86	0.20	16.07
0.10	0.07	0.00	0.00	0.00	12.00	0.86	6.43
0.33	0.00	5.48	0.00	18.00	0.16	0.00	10.71
0.18	0.00	1.21	13.33	0.43	14.57	3.43	0.31
0.12	0.47	0.00	0.55	0.00	0.20	0.20	1.50
0.07	0.27	14.67	6.67	0.00	6.00	1.71	0.00
0.10	0.05	62.86	6.67	33.75	1.71	0.86	3.21
0.10	2.86	1.21	0.00	1.50	4.29	6.00	0.00
0.10	0.05	62.86	6.67	22.50	1.71	0.86	16.07
0.30	0.07	0.00	0.00	0.00	6.00	1.71	1.61
0.04	0.07	1.21	2.74	0.25	12.00	0.40	0.18
0.43	0.00	0.00	0.00	0.00	0.86	0.00	3.21
0.00	0.00	251.43	26.67	0.00	6.00	0.86	14.46
0.08	0.57	0.00	0.00	11.25	6.00	0.86	0.75
0.80	0.57	0.00	0.00	0.37	6.00	0.00	1.50
0.00	0.04	0.00	1.10	0.00	12.00	2.57	2.25
0.04	0.29	0.00	0.55	0.00	6.00	6.00	0.00
0.29	0.00	0.00	16.07	1.71	0.00	0.00	0.00
0.43	10.00	0.00	0.00	22.50	0.00	6.00	6.43
0.50	0.33	0.00	10.96	0.00	6.00	12.00	9.64
0.00	2.00	0.00	0.00	3.21	6.00	0.20	0.00
0.00	0.29	0.00	0.00	12.86	3.43	0.00	0.00
0.20	0.57	1.21	0.00	6.43	3.00	0.00	3.21
0.20	0.57	0.00	0.00	6.43	3.43	0.00	3.21

0.05	0.00	0.00	0.00	0.00	0.00	0.00	0.00
1.29	0.00	0.00	0.00	9.64	1.71	0.00	0.00
0.43	1.14	0.00	0.00	3.21	9.00	1.71	6.43
2.86	0.00	0.00	9.64	2.57	4.29	0.75	0.00
0.12	0.47	0.00	0.55	0.00	0.20	0.20	1.50
2.00	2.86	0.00	0.00	9.64	3.43	3.43	1.61
0.00	0.00	0.00	0.00	0.00	12.00	0.00	0.00
0.04	0.03	1.21	6.67	0.75	6.00	0.10	0.06
0.00	0.00	125.71	20.00	0.00	2.57	2.57	9.64
1.29	0.57	0.00	0.00	9.64	0.00	6.00	0.31
0.00	125.71	13.33	0.00	2.57	1.71	12.86	3.00
0.86	0.00	33.33	45.00	0.86	0.00	22.50	10.71
0.57	0.00	0.00	6.43	1.71	1.71	0.00	0.04
0.20	0.57	0.00	0.00	3.21	4.29	0.00	0.00
0.00	0.13	29.33	20.00	22.50	4.71	0.03	1.61
0.00	0.08	0.00	0.00	0.00	6.00	12.00	0.00
0.40	3.43	0.00	0.00	0.00	6.00	6.00	0.00
1.43	0.00	0.00	9.64	2.57	2.57	0.00	0.04
0.43	2.57	14.67	6.67	0.00	6.00	2.57	0.37
4.29	0.12	62.86	28.57	48.21	19.71	8.14	0.00
0.02	1.20	1.21	0.55	0.00	12.00	0.02	0.00
0.57	0.00	0.00	67.50	6.00	6.00	3.21	0.00
0.00	0.86	0.00	0.00	9.64	2.57	0.00	0.00
1.33	14.67	20.00	1.61	9.00	4.29	0.75	1.00
0.00	0.29	0.00	0.00	45.00	2.57	6.00	3.21
0.35	4.00	0.00	0.00	0.00	12.00	6.00	0.00
0.13	14.67	20.00	45.00	0.86	0.00	12.86	2.00
0.08	1.14	2.41	1.10	9.64	6.00	1.71	0.75
1.00	0.00	0.00	12.86	3.43	0.00	0.00	0.00
0.43	0.00	0.00	0.00	3.21	0.20	0.40	0.00
0.00	0.00	0.00	0.55	0.00	6.00	0.00	0.00
0.02	0.13	14.67	20.00	45.00	0.86	0.00	12.86
0.00	0.29	125.71	20.00	0.00	6.00	0.86	12.86
0.13	14.67	20.00	22.50	1.29	0.00	9.64	1.00
0.00	0.00	251.43	26.67	0.00	6.00	0.86	14.46
1.43	0.00	0.00	9.64	2.57	2.57	0.00	0.21
0.05	0.03	7.23	3.29	0.37	6.00	6.00	0.37
0.00	0.03	0.00	0.00	0.00	0.86	0.00	0.00
0.03	0.13	0.00	0.00	9.64	6.00	6.00	3.21
0.20	0.57	1.21	6.67	0.75	1.71	12.00	0.06
0.03	125.71	13.33	45.00	6.00	0.00	8.04	1.75
0.57	1.21	0.00	8.04	4.29	2.57	3.21	0.08
0.40	0.07	14.67	6.67	22.50	0.60	0.00	0.00
0.57	125.71	33.33	22.50	4.29	0.20	6.43	4.29
0.00	0.00	62.86	13.33	22.50	6.00	0.86	3.21
1.00	0.57	1.21	0.00	6.43	2.57	0.86	6.43
0.43	0.00	14.67	6.67	0.00	0.86	0.00	0.00
0.04	0.07	1.21	2.74	0.25	12.00	0.40	0.18
0.00	2.00	125.71	53.33	45.00	1.71	0.20	3.21
0.00	0.29	0.00	0.00	3.21	6.00	0.86	3.21
9.00	6.00	0.00	0.00	45.00	0.00	0.00	0.00
0.05	0.13	7.23	3.29	0.31	6.00	0.08	0.31
0.00	188.57	20.00	0.00	2.57	1.71	12.86	3.00

0.10	0.11	0.00	57.14	0.00	6.00	0.00	0.00
0.00	0.20	0.00	0.00	0.18	6.00	0.60	6.43
0.10	2.00	0.00	0.00	3.21	12.00	6.00	0.75
0.57	0.00	0.00	45.00	6.00	6.00	3.21	0.00
0.30	0.20	0.00	0.00	0.00	2.57	6.00	6.43
0.00	62.86	6.67	45.00	6.00	0.20	16.07	2.14
0.16	0.00	314.29	33.33	22.50	0.86	0.86	16.07
0.00	0.00	0.00	0.00	0.00	30.00	0.00	0.00
0.40	0.33	0.00	0.00	0.00	6.00	1.71	0.75
0.00	314.29	33.33	0.00	5.14	0.86	6.43	3.00
0.05	125.71	13.33	22.50	2.00	1.71	6.43	5.36
0.02	0.13	14.67	20.00	56.25	0.43	0.00	14.46
0.05	0.86	0.00	0.00	0.00	6.00	2.57	0.18
1.29	0.57	0.00	0.00	0.75	0.86	6.00	9.64
0.20	0.57	1.21	0.00	6.43	3.00	0.00	3.21
0.30	0.33	0.00	0.00	11.25	6.00	6.00	0.00
0.00	0.86	0.00	0.00	67.50	12.00	0.00	0.00
0.12	0.47	0.00	0.55	0.00	0.20	0.20	1.50
0.08	0.57	0.00	0.00	11.25	6.00	0.86	0.75
0.00	1.00	0.00	1.10	33.75	0.20	6.00	0.00
1.00	0.57	1.21	0.00	6.43	2.57	0.86	6.43
0.02	0.02	0.00	0.00	0.18	6.00	0.86	0.00
0.12	0.16	0.00	6.03	0.00	12.00	0.05	0.00
0.00	0.29	0.00	0.00	0.75	6.00	1.71	0.00
2.00	2.86	0.00	0.00	9.64	3.43	3.43	1.61
0.00	0.57	0.00	0.00	67.50	6.00	6.00	3.21
0.00	0.29	0.00	0.00	6.43	2.57	1.71	0.00
0.57	125.71	33.33	45.00	1.71	0.20	3.21	2.14
0.00	0.57	0.00	0.00	45.00	6.00	6.00	3.21
0.00	1.43	62.86	0.00	9.64	12.00	0.00	3.21
0.20	0.07	0.00	2.74	0.00	6.00	6.00	0.00
0.00	1.43	0.00	0.00	9.64	2.57	2.57	0.00
0.05	0.86	0.00	0.00	0.00	1.71	0.00	0.00
0.20	0.57	1.21	0.00	6.43	3.43	0.00	3.21
1.50	2.86	0.00	0.00	0.00	0.00	6.00	0.06
0.08	0.00	314.29	33.33	0.00	5.14	0.86	6.43
0.10	3.43	1.21	0.00	1.50	4.29	12.00	0.12
0.04	0.29	0.00	0.00	0.00	6.00	0.50	0.00
0.00	0.86	0.00	0.00	9.64	2.57	0.00	0.00
0.00	0.13	0.00	0.00	0.00	6.00	2.57	1.50
0.00	2.86	0.00	0.00	0.00	6.00	6.00	0.00
0.00	0.00	0.00	0.00	9.64	6.00	0.00	0.00
1.00	2.86	1.21	6.67	2.25	2.57	12.00	0.06
7.29	0.18	65.71	57.14	73.93	33.43	10.29	0.00
0.00	0.13	188.57	26.67	0.00	6.00	0.86	16.07
2.14	0.40	0.00	0.00	12.86	4.29	0.00	0.00
0.00	0.57	0.00	0.00	6.43	2.57	1.71	0.00
0.57	0.00	0.00	6.43	3.43	0.00	3.21	0.04
0.00	0.67	0.00	0.00	0.37	6.00	0.86	0.00
8.57	1.33	0.00	0.00	0.00	6.00	6.00	0.00
0.00	1.43	0.00	0.00	9.64	2.57	2.57	0.00
0.00	0.57	0.00	0.00	45.00	6.00	6.00	3.21
0.00	0.07	0.00	0.00	6.43	3.43	2.57	0.00

0.00	0.00	0.00	0.27	0.00	6.00	0.10	0.00
0.00	0.00	0.00	0.00	0.00	6.00	12.00	0.00
0.57	1.21	0.00	6.43	3.43	0.00	3.21	0.04
0.20	0.00	62.86	6.67	22.50	6.00	0.20	16.07
0.00	1.43	0.00	0.00	45.00	2.14	6.00	3.21
0.00	0.57	0.00	0.00	45.00	6.00	6.00	3.21
0.50	0.33	0.00	10.96	0.00	6.00	12.00	9.64
0.20	0.00	7.23	0.00	0.00	6.00	0.40	0.00
0.00	0.20	0.00	0.00	11.25	2.57	0.86	0.00
0.00	0.86	0.00	0.00	16.07	2.57	0.00	0.00
0.00	0.13	251.43	26.67	0.00	6.00	0.86	14.46
0.00	0.13	29.33	20.00	45.00	3.43	0.03	1.61
0.20	0.57	0.00	0.00	6.43	2.57	1.71	0.00
1.00	0.57	1.21	0.00	9.64	3.43	1.71	3.21
0.43	2.00	0.00	0.00	0.00	6.00	6.00	0.00
0.00	0.13	0.00	0.00	3.21	6.00	0.00	0.00
0.00	1.43	0.00	0.00	45.00	2.14	6.00	3.21
0.12	0.47	0.00	0.55	0.00	0.20	0.20	1.50
1.00	0.57	1.21	0.00	8.04	2.14	1.71	6.43
0.07	8.00	0.00	0.00	0.00	6.00	6.00	0.00
0.57	1.21	0.00	8.04	4.29	2.57	3.21	0.08
0.16	0.00	314.29	33.33	0.00	5.14	0.86	8.04
4.29	6.00	2.41	28.57	0.00	3.43	2.57	0.00
0.00	0.03	29.33	20.00	0.00	18.00	0.00	0.31
0.00	0.86	0.00	0.00	9.64	2.57	0.00	0.00
0.04	1.14	0.00	0.55	0.00	6.00	0.86	0.12
0.00	0.13	29.33	20.00	22.50	4.71	0.03	1.61
0.00	0.05	0.00	0.00	0.37	6.00	0.10	0.00
0.00	12.00	0.00	0.00	0.00	9.00	2.57	0.00
0.05	0.29	0.00	13.33	0.06	6.00	6.00	9.64
0.20	0.00	0.00	0.00	0.00	6.00	6.00	1.61

sosemayor	badamzam	badam	gerdoo	peste	fandogh	tokhme	ghand
0.25	0.22	0.33	1.14	0.06	0.08	23.57	0.05
0.04	0.00	0.00	2.86	0.00	0.00	0.00	0.86
0.00	0.00	0.71	0.57	0.00	0.00	0.00	0.43
0.08	0.00	0.00	1.14	0.00	0.00	0.00	0.43
0.00	0.02	0.03	0.11	0.02	0.03	0.00	3.00
0.00	0.03	0.43	8.00	0.23	0.41	55.00	0.00
0.08	0.00	0.71	0.57	0.00	0.00	0.00	0.43
0.00	0.65	0.33	16.00	0.23	0.32	1.83	0.00
0.00	0.00	1.14	0.00	0.00	0.00	0.43	0.93
0.00	0.22	0.33	12.00	0.23	0.32	0.15	0.00
0.00	0.05	2.86	0.00	0.00	0.00	0.43	6.50
2.14	0.93	1.43	5.71	1.00	1.36	1.83	3.00
0.02	0.23	1.14	0.47	0.03	0.04	2.14	0.65
0.00	0.00	0.00	1.71	0.02	0.03	0.90	0.05
0.00	0.09	0.29	1.14	0.00	0.00	0.00	0.43
0.08	0.00	0.71	0.57	0.00	0.00	0.00	0.43
2.00	0.00	0.00	0.57	0.01	0.01	5.50	9.00
0.50	0.00	1.43	5.71	1.00	1.36	0.00	0.00
2.14	0.00	0.07	4.00	1.00	0.32	7.86	15.00
2.00	0.00	0.00	0.57	0.02	0.01	1.83	6.00
0.00	0.19	0.14	0.57	0.00	0.00	0.00	2.14
2.50	0.00	0.00	2.86	0.23	0.00	0.45	0.43
0.00	0.46	0.71	1.71	0.23	0.32	3.67	0.00
0.00	0.00	0.00	0.67	0.00	0.00	1.83	0.43
0.04	0.00	0.00	2.86	0.00	0.00	0.00	0.86
3.00	0.00	0.05	2.86	0.02	0.00	1.83	0.86
0.00	0.43	0.67	4.00	1.00	0.32	7.86	0.00
0.00	0.00	0.04	8.00	0.03	0.04	3.67	0.00
1.00	0.00	0.00	1.14	0.01	0.00	1.83	6.00
1.00	0.02	0.00	0.86	0.00	0.00	3.67	2.14
2.00	0.02	0.00	2.86	0.01	0.00	3.67	0.86
1.00	0.22	0.17	1.71	0.50	0.00	0.00	2.14
0.08	0.00	0.71	0.57	0.00	0.00	0.00	0.43
4.29	0.00	0.00	1.71	0.01	0.01	0.00	0.00
0.00	0.71	2.86	0.00	0.00	0.00	0.86	1.86
0.00	0.00	1.14	0.00	0.32	0.00	0.86	1.86
0.00	0.00	2.86	2.29	0.01	0.02	0.00	0.00
1.00	0.03	0.03	0.53	0.02	0.03	0.90	0.00
0.00	1.86	0.57	0.57	0.00	0.00	0.00	0.43
6.43	0.01	0.01	1.10	0.01	0.00	0.17	15.00
0.00	0.28	0.14	0.57	0.00	0.00	0.00	0.43
0.50	0.00	0.05	2.86	0.00	0.00	0.00	2.14
4.29	0.00	0.00	1.14	0.10	0.00	0.00	1.71
2.14	0.22	0.08	4.00	0.06	0.08	0.75	15.00
2.14	0.22	0.33	0.57	1.00	0.00	7.86	6.00
0.00	0.00	0.00	0.27	0.00	0.00	0.15	3.00
2.14	1.86	7.14	4.00	0.10	0.63	1.83	0.00
0.02	0.00	1.71	0.01	0.00	3.67	0.86	13.00
2.14	0.00	0.08	0.00	0.93	0.00	1.83	0.00
0.37	0.07	1.71	0.70	0.00	1.17	30.00	3.71
0.00	3.25	0.33	8.00	0.23	0.01	0.30	0.00
0.08	0.00	0.00	1.14	0.00	0.00	0.00	0.43

0.25	0.02	0.03	0.11	0.02	0.00	0.90	6.00
0.00	0.19	0.71	2.86	0.00	0.00	0.00	2.14
0.00	0.04	0.71	8.00	0.23	0.06	0.00	0.08
0.00	0.00	1.14	0.00	0.32	0.00	0.86	1.86
0.28	0.14	0.57	0.00	0.00	0.00	0.43	0.93
0.00	0.46	0.71	3.43	0.04	0.05	3.67	0.00
0.00	0.00	1.14	0.00	0.00	0.00	0.43	0.93
0.00	0.28	0.14	0.57	0.00	0.00	0.00	0.43
4.29	0.33	0.07	0.13	0.28	0.00	3.57	12.00
0.00	0.00	0.57	0.00	0.00	7.86	0.43	6.50
0.00	0.00	1.14	0.00	0.32	1.83	0.86	1.86
0.04	0.00	0.00	1.14	0.00	0.32	0.00	0.86
0.00	0.00	1.00	4.00	0.06	0.00	0.00	9.00
4.29	0.00	0.00	4.00	0.47	0.32	3.67	0.86
0.10	0.05	0.08	0.33	0.06	0.08	0.00	0.20
0.02	0.23	1.14	0.47	0.03	0.04	2.14	0.65
0.00	0.00	2.29	0.00	0.00	3.67	1.07	2.32
0.25	0.02	0.03	0.11	0.02	0.03	0.90	6.00
6.43	0.01	0.01	1.10	0.01	0.00	0.17	15.00
4.29	0.00	0.00	2.86	0.28	0.00	11.79	3.00
0.00	0.00	2.00	0.00	0.00	1.50	0.20	5.00
0.00	0.00	2.86	0.01	0.00	1.83	3.00	1.86
0.50	0.00	0.00	1.14	0.00	0.00	0.90	0.00
3.21	0.00	0.20	2.29	0.28	0.00	1.83	0.00
0.00	0.05	2.86	0.00	0.00	1.83	0.43	6.50
0.00	0.93	0.14	0.57	0.00	0.00	0.00	0.86
2.14	0.93	0.33	0.27	0.00	0.00	0.00	0.00
0.25	0.06	0.10	0.80	0.47	0.09	0.90	18.00
0.00	0.57	0.57	0.00	0.00	0.00	0.43	0.93
0.00	0.00	0.00	4.00	0.47	0.32	3.67	0.86
0.00	1.43	4.00	0.00	0.00	3.67	0.86	1.86
1.00	0.22	0.17	1.71	0.23	0.00	0.00	2.14
6.43	0.00	0.00	2.86	0.23	0.00	0.45	0.43
2.14	0.01	0.43	4.00	0.30	0.41	15.71	0.00
0.00	0.00	2.86	0.00	0.00	0.00	0.86	1.86
0.00	0.04	0.03	0.16	0.04	0.04	7.33	0.00
0.25	0.01	0.03	0.53	0.09	0.13	0.90	0.00
0.08	0.00	0.01	1.71	0.01	0.01	0.00	0.86
2.14	0.00	0.00	2.86	0.00	0.00	1.83	3.00
1.00	0.00	0.00	1.14	0.01	0.00	3.67	0.86
0.04	0.00	1.14	0.00	0.00	0.75	1.29	1.86
0.00	0.00	2.86	0.00	0.00	0.00	0.43	0.93
0.16	0.05	0.08	2.29	0.01	0.00	0.00	3.00
5.00	0.43	0.67	0.33	0.47	0.03	0.21	6.00
1.00	0.00	0.00	2.86	0.01	0.00	1.83	0.86
0.25	0.02	0.33	0.53	0.23	0.32	0.45	6.00
0.00	0.93	0.14	0.57	0.00	0.00	0.00	0.86
2.14	0.18	0.00	0.57	0.19	0.26	1.83	0.00
3.21	0.00	0.00	2.86	0.23	0.00	0.75	1.71
0.00	0.09	0.14	0.57	0.00	0.00	0.00	0.43
0.50	0.02	0.05	0.11	0.23	0.00	1.83	0.00
0.04	0.00	0.00	2.86	0.00	0.00	0.00	0.86
0.00	0.00	0.57	0.01	0.01	5.50	6.00	0.93

0.46	0.14	0.57	0.00	0.00	0.00	0.43	0.93
0.00	0.71	0.00	1.00	0.00	7.14	3.00	0.00
1.00	0.00	0.00	2.86	0.01	0.00	1.83	3.00
2.14	1.95	0.00	4.00	0.06	0.05	0.00	0.00
1.50	0.05	0.08	4.00	0.23	0.08	1.83	3.00
3.71	1.14	1.14	0.00	0.00	0.00	0.86	1.86
0.50	0.09	0.00	1.71	0.00	0.00	0.00	9.00
0.00	0.11	0.17	0.67	0.12	0.16	0.15	6.00
4.29	0.09	0.33	8.00	0.35	0.00	0.00	0.00
0.00	0.00	0.00	4.00	0.00	0.00	0.00	0.00
4.29	3.71	1.43	1.14	0.00	0.00	0.00	2.14
0.00	0.00	0.29	1.14	0.00	0.00	0.00	1.29
6.43	0.00	0.29	8.00	0.00	0.00	0.00	9.00
0.00	0.43	0.86	0.00	0.00	0.00	1.29	0.93
0.00	0.28	0.43	4.00	0.01	0.01	7.86	0.20
0.25	0.05	2.86	4.00	1.20	0.81	7.86	0.00
0.00	0.00	0.29	1.14	0.00	0.00	0.00	1.29
1.00	0.33	0.08	8.00	0.35	0.08	3.67	0.00
0.00	0.30	0.71	4.00	0.50	0.00	0.00	0.64
0.00	0.43	0.86	0.00	0.00	0.00	1.29	0.93
1.00	0.00	0.00	2.86	0.01	0.00	1.83	3.00
0.16	0.00	1.00	4.00	0.00	0.00	15.71	3.00
7.50	0.04	1.00	4.00	1.00	0.05	3.67	6.00
2.00	0.00	0.03	2.86	0.01	0.00	3.67	0.86
0.00	0.00	0.00	8.00	0.00	0.01	0.00	0.00
0.00	0.02	0.07	2.86	0.02	0.03	3.67	0.00
0.04	0.00	0.00	2.86	0.00	0.00	0.00	0.86
2.14	0.04	0.05	8.00	0.02	0.03	0.38	1.71
0.50	0.07	0.29	2.29	0.05	0.06	7.86	3.00
2.14	0.22	0.00	0.57	0.00	0.00	7.86	1.71
0.00	0.00	5.00	12.00	0.00	0.00	0.00	3.00
0.00	0.71	0.00	1.00	0.00	7.14	3.00	0.00
1.00	0.09	0.57	2.00	2.10	0.00	5.00	0.04
0.12	0.02	0.23	1.14	0.47	0.03	0.04	2.14
0.04	1.86	2.29	8.00	0.19	0.25	7.86	0.01
8.57	0.00	0.00	0.57	0.00	0.00	7.86	0.43
10.71	0.00	1.43	4.00	0.02	0.03	3.67	0.86
2.14	0.00	0.00	0.57	0.00	0.00	7.86	0.43
3.75	1.39	0.05	2.00	0.23	0.16	15.71	0.00
0.50	0.04	0.08	1.71	0.06	0.05	0.33	0.70
2.14	0.00	0.43	4.00	0.30	0.00	0.00	0.00
2.00	0.02	0.00	2.86	0.01	0.00	3.67	0.86
6.43	0.28	0.43	1.71	0.30	0.41	1.83	0.86
0.00	0.00	0.00	4.00	0.12	0.16	3.67	24.00
0.50	0.09	0.14	0.57	0.10	0.13	0.45	0.00
0.00	0.19	0.03	4.00	0.02	0.03	1.83	3.00
1.86	0.57	0.57	0.00	0.00	0.00	0.43	0.93
0.00	0.93	5.00	8.00	0.23	0.32	7.86	0.00
6.43	0.00	5.00	0.27	0.00	0.00	0.03	1.71
0.25	0.02	0.07	0.27	0.01	0.00	0.00	0.00
0.00	0.19	0.71	2.86	0.00	0.00	0.00	2.14
0.04	0.00	0.00	1.14	0.00	0.32	0.00	0.86
0.04	0.00	0.00	1.14	0.00	0.32	0.00	0.86

0.00	0.00	0.04	3.43	0.00	0.00	0.00	0.00
0.00	0.02	0.03	2.86	0.03	0.04	0.90	0.00
2.14	1.95	0.33	1.33	0.23	0.32	0.00	0.00
0.00	0.57	0.57	0.00	0.00	0.00	0.43	0.93
0.12	0.02	0.23	1.14	0.47	0.03	0.04	2.14
0.08	0.00	0.71	0.57	0.00	0.00	0.00	0.43
2.14	0.00	0.00	4.00	0.23	0.00	0.00	9.00
0.50	0.02	0.20	4.00	2.00	2.71	7.86	15.00
3.00	0.00	0.05	2.86	0.02	0.00	1.83	0.86
0.00	0.00	0.14	4.00	0.10	0.14	1.51	0.43
0.00	0.05	2.86	0.02	0.00	1.83	0.43	6.50
0.19	0.57	0.57	0.02	0.03	1.83	4.29	9.29
0.00	0.00	1.14	0.00	0.32	1.83	0.43	1.86
0.04	0.00	0.00	1.14	0.00	0.32	1.83	0.43
2.00	0.00	0.00	0.57	0.01	0.01	5.50	6.00
0.00	0.11	0.01	0.57	0.40	0.01	1.83	0.00
0.00	0.46	0.71	4.00	0.35	0.48	0.60	0.00
0.00	0.00	1.14	0.00	0.00	3.67	1.07	2.32
0.25	0.05	0.08	8.00	0.06	0.08	23.57	0.00
4.29	0.17	0.35	23.43	0.60	0.13	1.05	19.50
0.50	0.01	0.13	1.71	0.23	0.01	0.00	0.86
0.46	0.14	0.57	0.00	0.00	0.00	0.43	0.93
0.00	1.86	0.57	0.57	0.00	0.00	0.00	0.43
0.00	0.00	2.86	0.01	0.00	1.83	0.86	1.86
0.00	0.09	0.29	1.14	0.00	0.00	0.00	0.43
0.50	0.05	1.43	4.00	0.47	0.63	1.83	0.00
0.00	0.00	1.14	0.01	0.00	1.83	6.00	0.93
2.14	0.22	0.67	1.33	0.47	0.32	7.86	3.00
0.19	0.71	2.86	0.00	0.00	0.00	2.14	2.79
0.00	0.00	0.71	4.00	0.23	0.32	18.33	0.00
4.29	0.07	0.11	0.13	0.08	0.00	0.30	0.00
1.00	0.00	0.00	0.57	0.02	0.01	1.83	6.00
3.00	0.00	0.03	2.86	0.01	0.00	3.67	0.86
0.00	0.00	0.57	0.02	0.01	1.83	6.00	0.93
2.00	0.02	0.00	1.71	0.01	0.00	3.67	0.86
0.22	0.00	1.14	0.00	0.00	3.67	1.07	2.32
0.25	0.28	5.00	8.00	0.07	0.00	0.90	0.05
0.25	0.01	0.43	4.00	0.02	0.00	0.38	0.00
2.14	0.22	1.43	1.71	0.47	0.63	0.90	6.00
4.29	0.00	0.00	4.00	0.47	0.32	3.67	2.14
0.00	0.00	1.71	0.50	0.00	0.00	2.14	6.50
0.00	0.00	2.86	0.00	0.00	0.00	0.43	0.93
0.16	0.01	0.17	8.00	0.35	0.16	0.60	18.00
0.00	0.00	2.86	0.10	0.68	0.00	1.71	13.00
2.50	0.11	1.43	1.14	0.12	0.16	1.83	1.29
0.08	0.00	0.00	1.14	0.00	0.00	0.00	0.43
2.14	0.22	1.43	1.14	0.50	0.32	7.86	0.86
0.50	0.04	0.08	1.71	0.06	0.05	0.33	0.70
5.36	0.00	0.00	2.29	0.00	0.00	7.86	1.71
0.00	0.93	1.43	5.71	1.00	0.10	15.71	0.00
0.00	0.00	0.05	4.00	0.10	0.00	0.00	0.00
1.00	0.02	0.43	1.71	0.23	0.27	0.90	9.00
0.00	0.05	2.86	0.02	0.00	1.83	0.43	6.50

0.50	0.05	0.05	0.57	0.00	0.00	15.71	0.00
0.12	0.46	0.71	2.86	0.50	0.68	15.71	3.00
2.14	0.22	0.33	12.00	0.23	0.02	7.86	0.43
0.28	0.14	0.57	0.00	0.00	0.00	0.43	0.93
0.00	0.04	0.05	0.22	0.23	0.32	3.67	0.00
0.00	0.00	0.57	0.00	0.00	15.71	1.71	13.00
0.50	0.00	0.05	2.86	0.00	0.00	0.00	0.43
0.00	0.22	0.33	16.00	0.47	0.00	0.00	0.00
0.50	0.22	0.17	8.00	0.12	0.16	9.17	3.00
0.00	0.05	2.86	0.00	0.00	0.00	0.43	6.50
0.00	0.00	2.86	0.23	0.00	11.79	3.00	19.50
1.00	0.00	0.00	1.14	0.01	0.00	1.83	3.00
0.25	0.04	0.14	4.00	0.05	0.14	0.90	2.14
0.00	0.01	0.08	2.86	0.47	0.63	0.00	0.00
0.04	0.00	0.00	1.14	0.00	0.00	0.00	0.86
0.00	0.22	0.00	0.05	0.01	0.01	1.83	0.86
0.00	0.28	0.14	0.57	0.00	0.00	0.00	0.43
0.12	0.02	0.23	1.14	0.47	0.03	0.04	2.14
6.43	0.28	0.43	1.71	0.30	0.41	1.83	0.86
0.75	0.01	0.01	8.00	0.02	0.00	1.83	7.50
0.08	0.00	0.00	1.14	0.00	0.00	0.00	0.43
2.14	0.01	0.01	2.86	0.01	0.01	7.86	1.29
2.00	0.03	0.04	0.16	0.03	0.03	0.11	14.23
4.29	0.04	0.05	0.22	0.04	0.05	7.86	3.00
0.08	0.00	0.71	0.57	0.00	0.00	0.00	0.43
0.00	0.28	0.14	0.57	0.00	0.00	0.00	0.43
0.04	0.00	0.00	0.57	0.50	0.00	3.67	1.07
0.00	0.00	2.86	0.10	0.68	0.00	1.71	13.00
0.00	0.28	0.14	0.57	0.00	0.00	0.00	0.43
2.14	0.00	0.00	2.86	0.00	0.00	1.83	6.00
1.00	0.22	0.33	16.00	0.23	0.04	7.86	0.00
0.21	0.22	0.00	1.14	0.00	0.00	3.67	1.07
0.00	0.00	0.33	0.67	0.06	0.00	0.00	3.00
0.04	0.00	0.00	1.14	0.00	0.32	0.00	0.86
0.00	0.01	0.57	4.00	0.40	0.41	3.67	0.00
3.00	0.00	0.05	2.86	0.00	0.00	1.83	0.43
10.71	0.00	1.43	4.00	0.02	0.03	3.67	1.29
0.00	0.22	0.17	4.00	0.23	0.32	3.67	0.00
0.00	0.00	0.57	0.57	0.00	0.00	0.00	0.43
1.00	0.05	0.33	4.00	0.00	0.32	1.83	9.00
0.00	3.25	0.43	8.00	0.30	0.41	15.71	0.00
6.43	0.00	0.33	4.00	0.47	0.00	3.67	6.00
0.00	0.00	0.00	4.00	0.23	0.32	18.33	0.86
8.57	0.35	0.62	25.14	1.19	0.26	1.66	36.00
1.00	0.00	0.00	2.29	0.00	0.00	3.67	1.29
2.14	0.09	0.14	2.29	0.10	0.00	1.83	3.00
0.04	0.00	0.00	2.29	0.00	0.00	3.67	1.07
0.00	0.00	1.14	0.00	0.32	1.83	0.86	1.86
0.00	0.33	0.50	12.00	0.23	0.32	2.75	6.00
0.00	0.04	5.00	12.00	7.00	2.04	15.71	0.00
0.08	0.00	0.00	1.14	0.00	0.00	3.67	1.07
0.00	0.28	0.14	0.57	0.00	0.00	0.00	0.43
0.00	0.04	0.43	0.13	0.00	0.00	0.00	0.20

0.21	0.00	0.00	0.05	0.00	0.00	0.08	6.00
0.50	0.00	0.00	16.00	0.00	0.00	0.00	0.00
0.00	0.00	1.14	0.00	0.32	0.00	0.86	1.86
2.14	0.00	0.00	0.57	0.00	0.00	15.71	1.71
0.00	0.93	0.14	0.57	0.00	0.00	0.00	0.43
0.00	0.28	0.14	0.57	0.00	0.00	0.00	0.43
6.43	0.00	5.00	0.27	0.00	0.00	0.03	1.71
0.25	0.05	0.08	1.71	0.02	0.00	15.71	0.00
0.00	0.04	0.05	8.00	0.04	0.05	0.00	0.00
0.00	0.00	0.71	0.57	0.00	0.00	0.00	0.43
2.00	0.02	0.00	1.71	0.01	0.00	3.67	0.86
2.00	0.00	0.00	0.57	0.01	0.01	5.50	15.00
0.04	0.00	0.00	1.14	0.00	0.32	1.83	1.07
0.08	0.00	0.71	0.57	0.00	0.00	0.00	0.43
0.00	0.00	1.43	4.00	1.00	0.32	0.75	0.00
0.00	0.00	0.10	0.40	0.23	0.00	0.00	0.00
0.00	0.93	0.14	0.57	0.00	0.00	0.00	0.43
0.12	0.02	0.23	1.14	0.47	0.03	0.04	2.14
0.08	0.00	0.00	1.14	0.00	0.00	0.00	0.43
0.12	0.22	2.14	8.00	1.00	0.01	0.00	1.29
0.00	0.00	1.14	0.00	0.00	0.00	0.43	0.93
0.50	0.00	0.05	2.86	0.00	0.00	0.00	0.43
0.00	2.60	4.00	4.00	0.07	0.10	1.83	0.00
0.25	0.00	0.43	2.29	0.00	0.00	1.51	0.00
0.00	0.00	0.57	0.57	0.00	0.00	0.00	0.43
0.04	0.43	0.70	8.00	0.65	0.89	7.86	0.00
2.00	0.00	0.00	0.57	0.01	0.01	5.50	6.00
0.16	0.33	0.50	4.00	0.35	0.48	0.60	0.20
0.50	0.00	0.50	0.00	0.03	0.00	15.71	0.00
2.14	0.04	0.07	0.27	0.05	0.06	0.00	0.00
0.00	0.22	0.33	8.00	0.23	0.00	15.71	6.00

shekar	asal	morab	nooshabe	shirinikhos	shirinitar	gaz	sohan
0.11	0.47	0.33	3.84	2.14	1.47	0.29	0.33
1.86	5.00	1.33	0.55	2.14	0.73	2.50	0.00
0.93	5.00	0.00	0.00	2.14	0.00	2.50	0.00
0.93	5.00	1.33	0.55	4.29	0.73	5.00	0.00
6.50	0.00	0.22	85.71	1.00	1.47	0.05	0.00
0.00	7.00	0.00	0.00	0.00	0.00	0.00	0.00
0.93	5.00	1.33	0.55	4.29	0.73	5.00	0.00
0.00	7.00	2.86	6.67	15.00	1.47	0.10	0.11
5.00	1.33	0.55	4.29	0.73	5.00	0.00	0.19
0.00	0.00	8.57	0.00	0.00	0.00	0.00	0.00
5.00	2.67	85.71	10.71	2.20	2.50	1.33	1.17
6.50	7.00	5.71	6.67	2.14	3.14	10.00	5.71
3.00	2.00	13.33	1.50	0.73	1.17	1.33	0.10
0.22	0.23	0.67	13.33	0.16	0.30	0.24	0.00
0.93	2.00	0.00	0.00	3.21	1.10	2.50	0.00
0.93	5.00	1.33	0.55	4.29	0.73	5.00	0.00
0.93	2.00	14.29	13.33	3.21	1.83	0.24	0.05
0.00	2.00	0.00	6.67	0.16	0.24	0.58	0.11
0.00	2.00	0.00	57.14	0.50	0.24	0.19	0.22
0.93	4.00	11.43	13.33	3.21	1.83	0.24	0.05
2.79	4.00	0.00	0.00	4.29	0.00	5.00	0.67
6.50	1.00	4.00	28.57	2.14	7.33	5.00	0.00
6.50	2.00	2.86	0.00	0.12	0.18	0.00	0.05
0.00	1.50	0.16	0.00	0.08	0.24	7.50	1.33
1.86	5.00	1.33	0.55	2.14	0.73	2.50	0.00
13.00	5.00	2.67	171.43	4.29	3.67	5.00	2.00
0.00	7.00	20.00	0.55	0.00	0.73	0.00	0.00
6.50	0.00	0.00	0.00	0.00	0.36	0.29	0.00
0.93	4.00	11.43	13.33	3.21	2.20	0.24	0.05
2.32	2.00	1.33	85.71	6.43	3.67	2.50	0.67
13.00	6.00	3.33	142.86	10.71	3.67	5.00	2.00
13.00	2.00	2.67	128.57	9.64	3.67	5.00	0.00
0.93	5.00	1.33	0.55	4.29	0.73	5.00	0.00
0.00	3.00	0.00	0.00	2.14	3.14	0.19	0.22
5.00	2.86	0.00	2.14	0.73	0.00	0.67	0.00
5.00	2.00	0.55	2.14	0.12	0.00	0.00	0.19
0.00	1.00	0.00	6.67	0.00	0.00	0.10	0.11
0.00	1.00	11.43	0.00	0.25	0.36	0.29	0.33
0.93	5.00	0.00	0.00	2.14	0.00	2.50	0.00
0.93	0.70	1.63	13.33	0.50	0.73	0.05	0.05
0.93	2.00	0.00	0.00	2.14	0.00	2.50	0.00
13.00	2.00	2.67	128.57	6.43	2.20	2.50	1.33
13.00	4.00	1.33	28.57	2.14	6.29	1.17	1.33
0.00	2.00	8.57	6.67	0.50	0.73	0.00	0.00
0.00	2.00	0.00	6.67	1.00	0.00	5.00	0.00
6.50	0.00	0.00	20.00	0.25	0.36	0.10	0.11
0.00	0.23	0.67	28.57	2.14	0.73	0.00	0.00
6.00	3.33	142.86	10.71	3.67	5.00	3.33	5.83
0.00	0.00	0.00	6.67	0.50	0.00	0.29	0.00
2.10	0.38	57.14	1.50	2.20	1.17	0.55	1.17
6.50	2.00	0.00	28.57	0.08	0.73	0.10	0.11
0.93	5.00	1.33	0.55	4.29	0.73	5.00	0.00

0.11	0.12	0.33	13.33	0.25	0.36	0.00	0.00
2.79	4.00	0.00	0.00	4.29	0.00	5.00	0.67
0.00	0.00	0.00	13.33	0.16	0.24	0.00	0.00
5.00	2.00	0.55	2.14	0.12	0.00	0.00	0.19
2.00	0.00	0.00	2.14	1.47	2.50	0.00	0.00
1.86	7.00	20.00	0.00	0.00	0.00	0.00	0.00
5.00	1.33	0.55	4.29	0.73	5.00	0.00	0.19
0.93	2.00	0.00	0.00	2.14	0.00	2.50	0.00
19.50	1.17	1.40	57.14	1.00	1.47	0.10	0.00
5.00	3.33	57.14	10.71	15.71	0.00	14.29	5.00
2.00	2.00	0.55	2.14	0.12	0.00	0.00	0.19
1.86	5.00	2.00	0.55	2.14	0.12	0.00	0.00
0.00	1.00	0.00	3.29	1.00	0.00	0.00	0.00
1.86	2.00	1.33	0.00	4.29	0.73	0.00	0.00
0.43	0.47	1.33	13.33	0.16	0.24	0.00	0.00
3.00	2.00	13.33	1.50	0.73	1.17	1.33	0.10
2.50	2.00	0.55	2.14	0.12	1.17	0.00	0.29
0.00	7.00	0.33	13.33	0.21	0.30	0.05	8.57
0.93	0.70	1.63	13.33	0.50	0.73	0.05	0.05
19.50	5.00	3.33	114.29	4.29	14.14	0.00	0.00
2.00	2.00	132.00	5.00	2.00	2.00	1.00	1.00
1.50	5.71	13.33	3.21	2.20	0.24	0.05	1.17
6.50	0.00	0.00	0.00	0.50	0.73	0.00	0.00
1.86	1.00	8.57	57.14	6.43	0.73	0.58	0.67
5.00	2.67	171.43	4.29	3.67	5.00	2.00	2.33
2.79	4.00	0.00	0.00	4.29	0.00	5.00	0.67
0.22	3.00	1.33	42.86	0.75	0.36	0.00	0.00
0.11	3.00	0.33	13.33	6.43	3.14	0.29	0.33
5.00	0.00	1.10	2.14	0.00	2.50	0.00	0.00
1.86	3.00	1.33	0.00	4.29	0.73	0.00	0.00
2.00	0.00	0.00	1.07	0.37	0.58	0.00	0.10
13.00	2.00	2.67	128.57	9.64	3.67	5.00	0.00
6.50	1.00	4.00	28.57	2.14	6.29	5.00	0.00
0.00	5.00	0.00	3.29	6.43	0.30	1.17	1.33
5.00	1.33	0.55	4.29	0.73	5.00	0.00	0.19
0.00	0.00	0.33	13.33	6.43	0.00	0.29	0.33
6.50	0.47	0.33	13.33	0.16	0.30	0.00	0.16
0.00	2.00	2.86	2.19	0.16	0.24	0.00	0.00
0.93	5.00	0.00	0.00	4.29	0.00	0.00	0.00
13.00	5.00	1.33	57.14	4.29	0.73	2.50	0.67
2.00	1.33	57.14	10.71	3.67	7.50	0.00	3.50
5.00	1.33	0.55	4.29	0.73	5.00	0.00	0.19
1.86	0.23	0.67	6.67	0.50	0.73	0.24	0.27
19.50	14.00	14.00	57.14	1.00	2.20	0.24	0.11
1.86	1.50	2.86	13.33	3.21	2.20	0.24	0.05
6.50	0.70	0.00	3.29	0.12	0.18	0.10	0.11
2.79	4.00	0.00	0.00	4.29	0.00	5.00	0.67
6.50	0.00	0.00	28.57	0.50	0.73	0.00	0.00
13.00	2.00	1.33	28.57	2.14	15.71	2.50	0.00
0.93	5.00	0.00	0.00	2.14	0.00	2.50	0.00
0.00	7.00	0.67	28.57	0.25	0.18	0.14	0.16
1.86	5.00	1.33	0.55	4.29	0.73	5.00	0.00
2.00	14.29	13.33	3.21	1.83	0.24	0.05	2.33

2.00	0.00	0.00	2.14	0.73	2.50	0.00	0.00
2.33	2.33	13.33	0.00	0.00	0.00	0.00	0.00
1.86	1.50	5.71	13.33	3.21	2.20	0.24	0.05
0.00	1.00	20.00	0.00	0.50	0.73	0.05	0.05
0.00	0.23	0.67	6.67	0.08	0.73	0.05	0.05
7.00	0.00	0.00	4.29	0.00	5.00	0.00	0.00
6.50	2.00	2.67	3.29	0.00	0.00	0.00	0.16
0.00	1.00	5.71	0.00	0.00	0.00	0.00	0.00
0.93	7.00	0.00	0.00	0.25	0.36	0.00	0.11
0.00	7.00	1.00	0.00	0.21	0.30	0.00	0.00
4.64	2.00	1.33	0.00	10.71	15.71	0.00	0.00
0.93	2.00	0.00	0.00	3.21	0.73	2.50	0.00
6.50	1.00	20.00	0.00	0.50	1.57	0.00	2.86
2.00	0.00	0.00	3.21	0.73	2.50	0.00	0.00
0.00	3.00	0.00	0.00	0.25	0.36	2.50	0.00
0.11	3.00	0.33	57.14	0.25	0.36	0.29	0.16
0.93	2.00	0.00	0.00	3.21	0.73	2.50	0.00
6.50	1.00	2.86	2.74	6.43	0.24	0.10	0.11
0.00	3.00	0.14	6.67	8.57	0.24	0.05	0.05
2.00	0.00	0.00	3.21	0.73	2.50	0.00	0.00
1.86	1.50	5.71	13.33	3.21	2.20	0.24	0.05
6.50	7.00	0.00	6.67	0.16	0.36	0.10	0.11
6.50	7.00	1.33	6.67	0.50	0.73	0.58	0.16
13.00	6.00	3.33	142.86	10.71	3.67	5.00	2.00
0.00	7.00	20.00	28.57	0.16	0.24	0.00	0.00
0.00	0.47	0.00	0.00	0.00	0.00	0.00	0.00
1.86	5.00	1.33	0.55	4.29	0.73	5.00	0.00
0.00	1.00	0.25	1.37	0.08	0.00	0.07	0.08
2.79	3.00	2.86	13.33	0.00	0.00	0.00	0.00
6.50	1.00	3.33	142.86	4.29	6.29	2.92	0.67
6.50	14.00	5.71	28.57	0.00	0.00	0.00	0.00
2.33	2.33	13.33	0.00	0.00	0.00	0.00	0.00
0.43	3.00	7.00	13.33	2.50	2.93	1.75	0.38
0.65	3.00	2.00	13.33	1.50	0.73	1.17	1.33
6.50	2.00	0.67	28.57	0.00	0.73	0.19	0.22
6.50	5.00	3.33	57.14	10.71	15.71	0.00	0.00
1.86	2.00	0.00	0.00	1.07	0.37	0.58	0.00
6.50	5.00	3.33	142.86	10.71	15.71	0.00	14.29
0.22	7.00	0.33	0.00	2.14	0.73	6.25	0.00
0.22	3.00	2.00	6.67	2.14	0.73	0.14	0.16
0.00	7.00	0.67	6.67	2.14	0.00	2.50	0.11
13.00	6.00	3.33	142.86	10.71	3.67	5.00	2.00
0.43	7.00	2.86	6.67	1.00	0.06	0.00	0.00
0.00	7.00	2.86	13.33	0.21	0.00	0.29	0.49
0.00	0.23	0.00	2.19	0.25	0.36	0.10	0.05
6.50	0.23	2.86	0.00	0.50	0.73	0.00	0.33
2.00	0.00	0.00	2.14	0.00	2.50	0.00	0.00
0.00	7.00	2.86	0.00	2.14	0.73	0.10	0.11
0.00	13.00	2.57	20.00	1.50	0.73	0.58	5.33
0.00	1.00	5.71	1.10	0.00	0.00	0.29	0.33
2.79	4.00	0.00	0.00	4.29	0.00	5.00	0.67
1.86	5.00	2.00	0.55	2.14	0.12	0.00	0.00
1.86	2.00	2.00	0.55	2.14	0.12	0.00	0.00

0.00	7.00	0.00	28.57	0.25	0.18	1.17	1.33
0.00	0.00	0.00	0.00	0.25	0.36	0.00	0.33
0.22	7.00	2.86	0.00	1.00	1.47	0.10	0.11
5.00	0.00	1.10	2.14	0.00	2.50	0.00	0.00
0.65	3.00	2.00	13.33	1.50	0.73	1.17	1.33
0.93	5.00	1.33	0.55	4.29	0.73	5.00	0.00
0.00	1.00	0.00	28.57	0.50	1.47	0.00	0.00
6.50	7.00	20.00	14.29	4.29	0.73	0.58	0.33
13.00	5.00	2.67	171.43	4.29	3.67	5.00	2.00
0.00	7.00	0.33	2.74	6.43	0.00	2.50	2.86
5.00	2.67	171.43	4.29	3.67	5.00	2.00	2.33
5.00	5.71	57.14	2.14	12.57	2.50	5.71	10.00
3.00	2.00	0.55	2.14	0.12	1.17	0.00	0.19
1.86	3.00	2.00	0.55	2.14	0.12	0.00	0.00
0.93	2.00	14.29	13.33	3.21	1.83	0.24	0.05
0.00	7.00	20.00	6.67	0.08	0.12	0.00	0.00
0.00	7.00	0.00	0.00	0.00	0.18	0.10	0.11
2.50	2.00	0.55	2.14	0.18	1.17	0.00	0.29
0.02	3.00	0.16	13.33	1.00	1.47	0.10	0.11
21.82	7.50	17.14	35.24	5.03	2.19	0.87	0.52
0.00	1.00	5.71	0.55	4.29	0.73	0.05	0.05
2.00	0.00	0.00	2.14	1.47	2.50	0.00	0.00
0.93	5.00	0.00	0.00	2.14	0.00	2.50	0.00
1.50	2.86	13.33	3.21	2.20	0.24	0.05	0.58
0.93	2.00	0.00	0.00	3.21	1.10	2.50	0.00
0.00	1.00	2.86	0.00	0.50	6.29	0.00	0.00
4.00	11.43	13.33	3.21	2.20	0.24	0.05	1.17
6.50	2.00	5.71	0.00	1.00	0.73	0.58	0.67
4.00	0.00	0.00	4.29	0.00	5.00	0.67	0.00
0.00	7.00	0.00	0.00	1.50	0.00	0.00	0.22
0.65	1.00	0.00	0.00	0.16	0.24	0.10	0.00
0.93	4.00	11.43	13.33	3.21	2.20	0.24	0.05
13.00	6.00	3.33	142.86	10.71	3.67	5.00	2.00
4.00	11.43	13.33	3.21	2.20	0.24	0.05	1.17
13.00	6.00	3.33	142.86	10.71	3.67	5.00	3.33
2.50	2.00	0.55	2.14	0.12	1.17	0.11	0.29
0.11	7.00	0.33	3.29	0.25	0.36	0.14	0.16
0.00	0.23	0.00	0.00	0.00	0.00	0.14	0.11
0.00	3.00	0.67	13.33	0.50	0.73	0.00	0.67
0.93	3.00	3.33	0.00	2.14	0.73	2.50	2.86
1.00	3.33	128.57	10.71	3.67	5.00	0.00	0.00
5.00	1.33	0.55	4.29	0.73	5.00	0.00	0.19
0.65	0.10	0.67	6.67	0.50	0.73	0.24	0.11
4.00	1.33	28.57	10.71	6.29	0.00	5.71	10.00
1.86	2.00	1.33	57.14	4.29	1.47	5.00	0.67
0.93	5.00	1.33	0.55	4.29	0.73	5.00	0.00
1.86	3.00	2.86	20.00	2.14	0.73	0.00	0.11
0.22	3.00	2.00	6.67	2.14	0.73	0.14	0.16
13.00	4.00	1.33	28.57	2.14	6.29	1.17	1.33
0.00	7.00	0.67	3.29	6.43	2.20	0.10	0.11
0.00	14.00	0.00	0.00	0.00	0.60	0.48	0.00
6.50	0.00	0.00	13.33	0.21	0.30	0.05	0.11
5.00	2.67	171.43	4.29	3.67	5.00	2.00	2.33

0.00	7.00	1.33	0.00	0.21	0.30	0.00	0.33
0.93	0.00	8.57	28.57	1.00	1.47	0.58	0.67
0.93	14.00	2.86	13.33	0.50	0.00	0.58	0.00
2.00	0.00	0.00	8.57	0.00	2.50	0.00	0.00
0.00	2.00	0.00	0.00	0.16	0.00	0.00	0.11
5.00	3.33	57.14	2.14	15.71	0.00	0.00	0.00
6.50	5.00	2.67	85.71	4.29	2.20	2.50	1.33
0.00	0.00	0.00	0.00	4.29	0.00	0.00	0.00
1.86	2.00	0.67	6.67	0.50	1.47	0.29	0.33
5.00	2.67	171.43	4.29	3.67	5.00	2.00	1.17
5.00	3.33	114.29	4.29	14.14	0.00	0.00	5.00
0.93	1.50	5.71	13.33	3.21	2.20	0.24	0.05
0.43	7.00	8.57	0.00	0.25	0.36	0.29	0.33
0.00	7.00	0.00	0.00	1.00	1.47	0.00	0.05
1.86	5.00	1.33	0.55	2.14	0.12	0.00	0.00
0.00	2.00	0.67	6.67	0.00	0.00	0.00	0.00
0.93	2.00	0.00	0.00	2.14	0.00	2.50	0.00
0.65	3.00	2.00	13.33	1.50	0.73	1.17	1.33
0.43	7.00	2.86	6.67	1.00	0.06	0.00	0.00
0.54	7.00	7.14	28.57	2.14	0.09	0.05	0.05
0.93	5.00	1.33	0.55	4.29	0.73	5.00	0.00
0.93	0.47	5.71	6.67	0.50	0.73	0.05	0.05
4.77	10.00	28.00	0.55	2.22	1.81	0.05	0.27
0.00	1.00	8.57	20.00	7.50	11.00	0.00	0.11
0.93	5.00	1.33	0.55	4.29	0.73	5.00	0.00
0.93	2.00	0.00	0.00	2.14	0.00	2.50	0.00
2.32	2.50	2.00	0.55	2.14	0.12	1.17	0.00
4.00	1.33	28.57	10.71	6.29	0.00	0.00	5.00
0.93	2.00	0.00	0.00	2.14	0.00	2.50	0.00
1.86	5.00	0.00	0.00	4.29	0.00	0.00	0.00
0.00	7.00	0.00	28.57	1.00	1.47	0.29	0.16
2.32	2.50	2.00	0.55	2.14	0.12	1.17	0.11
0.00	0.12	0.00	28.57	0.00	0.00	0.00	0.00
1.86	5.00	2.00	0.55	2.14	0.12	0.00	0.00
0.00	7.00	0.16	0.00	0.08	0.12	0.10	0.05
6.50	5.00	2.67	171.43	4.29	3.67	5.00	2.00
2.79	3.00	1.33	0.00	1.07	0.37	0.58	0.00
0.00	7.00	1.33	6.67	0.08	0.00	0.58	0.11
0.93	5.00	0.00	0.00	2.14	0.00	2.50	0.00
6.50	5.00	0.00	6.67	0.50	0.30	0.00	0.00
0.00	7.00	0.00	0.00	0.00	0.00	0.05	0.05
0.00	21.00	0.00	6.67	0.00	0.73	0.00	0.00
1.86	3.00	1.33	0.00	4.29	0.73	0.00	0.00
17.14	24.00	21.71	70.48	9.56	4.02	1.45	0.71
1.86	3.50	1.33	85.71	6.43	2.20	2.50	0.67
0.93	2.00	0.00	1.10	2.14	0.06	2.50	0.11
2.32	2.50	2.00	0.55	2.14	0.12	1.17	0.00
2.00	2.00	0.55	2.14	0.12	0.00	0.00	0.19
6.50	0.00	0.00	0.00	0.50	2.20	0.00	1.33
0.00	14.00	11.43	0.00	0.00	0.00	0.00	0.19
2.32	2.50	2.00	0.55	2.14	0.18	1.17	0.11
0.93	2.00	0.00	0.00	8.57	0.00	2.50	0.00
0.00	7.00	0.00	0.00	0.00	0.00	0.00	0.00

0.00	0.00	0.00	2.19	0.50	0.73	0.00	0.00
0.00	3.00	8.57	0.00	0.50	0.00	0.00	0.00
5.00	2.00	0.55	2.14	0.12	0.00	0.00	0.19
13.00	5.00	3.33	142.86	4.29	6.29	0.00	0.00
0.93	2.00	0.00	0.00	3.21	1.10	2.50	0.00
0.93	2.00	0.00	0.00	2.14	0.00	2.50	0.00
0.00	13.00	2.57	20.00	1.50	0.73	0.58	5.33
0.00	7.00	1.33	13.33	0.50	0.18	0.10	0.11
0.00	3.00	0.00	0.00	0.06	0.09	0.14	0.16
0.93	5.00	0.00	0.00	2.14	0.00	2.50	0.00
13.00	6.00	3.33	142.86	10.71	3.67	5.00	3.33
0.93	4.00	11.43	13.33	3.21	1.83	0.24	0.05
2.32	2.50	2.00	0.55	2.14	0.12	1.17	0.00
0.93	5.00	1.33	0.55	4.29	0.73	5.00	0.00
0.00	7.00	20.00	28.57	15.00	0.36	0.14	0.16
0.00	0.23	0.00	6.67	0.08	0.06	0.00	0.00
0.93	2.00	0.00	0.00	2.14	0.73	2.50	0.00
0.65	3.00	2.00	13.33	1.50	0.73	1.17	1.33
0.93	5.00	1.33	0.55	4.29	0.73	5.00	0.00
0.00	7.00	0.00	1.64	15.00	0.73	0.10	0.11
5.00	1.33	0.55	4.29	0.73	5.00	0.00	0.19
6.50	5.00	2.67	285.71	4.29	2.20	2.50	1.33
5.57	0.00	0.00	28.57	12.86	9.43	0.10	0.11
6.50	7.00	1.33	13.33	0.25	0.00	0.29	0.00
0.93	5.00	0.00	0.00	2.14	0.00	2.50	0.00
0.00	7.00	5.71	0.00	0.50	0.36	0.96	0.11
0.93	2.00	14.29	13.33	3.21	1.83	0.24	0.05
0.00	0.47	1.33	0.00	2.14	3.14	0.24	0.00
6.50	7.00	0.67	28.57	0.50	0.12	0.00	0.11
0.00	2.00	0.00	0.00	0.08	0.12	0.00	0.00
0.00	7.00	0.00	6.67	0.25	0.00	0.00	0.00

pofak	shokolat	chay	namak	chips	ghahve	ablimo	abnabat
0.58	1.71	1250.00	0.05	1.81	9.33	0.08	0.08
0.19	0.57	500.00	3.00	0.00	0.19	0.71	0.71
0.00	1.14	1250.00	3.00	0.00	0.00	15.00	0.71
0.19	1.14	750.00	3.00	0.00	0.38	0.71	0.71
0.38	0.04	750.00	3.00	0.00	4.67	0.00	8.00
0.00	0.00	250.00	3.00	0.00	0.00	5.00	0.71
0.19	1.14	750.00	3.00	0.00	0.00	1.43	0.36
0.00	0.27	500.00	0.00	0.00	4.67	0.00	1.43
1.14	750.00	3.00	0.00	0.19	0.71	0.71	5.33
0.00	0.57	0.00	9.00	0.00	0.00	0.17	0.00
0.57	500.00	3.00	18.33	0.38	0.17	0.17	0.00
0.00	2.29	1000.00	3.00	3.67	40.00	5.00	0.50
0.02	500.00	6.00	0.30	0.77	0.71	1.83	2.67
0.00	0.07	250.00	3.00	7.33	2.30	0.00	0.00
0.00	8.00	1250.00	3.00	0.00	0.00	0.17	1.43
0.19	1.14	750.00	3.00	0.00	0.00	1.43	0.36
2.33	2.29	250.00	6.00	11.00	4.67	0.17	0.71
2.33	0.00	625.00	0.00	0.00	0.00	2.14	0.33
0.00	0.57	1250.00	0.00	0.00	0.00	0.17	0.33
2.33	2.29	500.00	6.00	18.33	4.67	0.17	0.71
0.00	1.71	35.71	3.00	0.00	0.00	15.00	2.86
0.00	2.86	500.00	3.00	18.33	0.77	0.17	0.17
0.00	0.27	750.00	6.00	0.45	140.00	3.57	0.17
0.00	0.00	35.71	3.00	0.00	0.00	0.00	0.17
0.19	0.57	500.00	3.00	0.00	0.19	0.71	0.71
2.33	2.57	500.00	4.50	11.00	0.38	0.17	0.83
0.00	0.00	750.00	3.00	0.00	0.00	10.00	0.71
0.00	0.00	750.00	9.00	0.00	0.00	3.57	0.00
1.17	2.29	500.00	6.00	7.33	4.67	0.42	1.79
7.00	5.71	500.00	3.00	11.00	0.38	0.25	0.50
3.50	2.86	500.00	3.00	11.00	0.38	0.17	0.33
1.17	5.71	500.00	3.00	11.00	0.58	0.42	0.50
0.19	1.14	750.00	3.00	0.00	0.00	1.43	0.36
10.00	0.00	750.00	3.00	0.00	0.00	5.00	5.00
0.57	500.00	3.00	0.00	0.38	0.01	2.86	0.22
0.57	500.00	3.00	0.00	0.19	1.43	1.43	10.67
1.17	0.03	750.00	3.00	0.30	0.00	0.00	0.67
0.58	0.07	750.00	3.00	1.81	2.30	1.43	0.08
0.00	1.14	1250.00	3.00	0.00	0.00	15.00	0.71
0.10	0.80	607.14	3.00	3.67	0.38	0.21	0.17
0.00	8.00	1250.00	3.00	0.00	0.00	0.67	0.71
1.17	0.57	500.00	3.00	9.17	0.38	0.17	0.17
10.00	0.00	500.00	3.00	0.00	0.77	0.33	0.33
1.17	4.00	1250.00	0.00	0.00	0.00	5.00	0.33
10.00	0.00	500.00	3.00	0.90	40.00	5.00	1.43
0.38	0.13	1000.00	0.00	0.00	0.00	2.14	0.00
1.17	0.13	250.00	3.00	0.00	60.00	0.71	1.43
2.29	500.00	3.00	14.67	0.38	0.17	0.33	0.00
0.00	0.00	250.00	0.00	0.00	0.00	0.00	0.00
0.00	1250.00	3.00	3.67	1.53	1.00	1.17	2.67
0.00	0.00	250.00	3.00	7.33	4.67	5.00	0.71
0.19	1.14	750.00	3.00	0.00	0.38	0.71	0.71

0.00	0.00	500.00	6.00	0.00	0.00	3.57	2.14
0.00	1.71	35.71	3.00	0.00	0.00	15.00	1.43
0.00	0.07	250.00	3.00	0.30	0.00	0.14	0.00
0.57	500.00	3.00	0.00	0.19	0.71	0.71	6.67
8.00	1250.00	3.00	0.00	0.00	0.33	0.71	0.00
0.00	0.57	250.00	3.00	0.00	0.00	0.00	0.00
1.14	750.00	3.00	0.00	0.38	0.71	0.71	5.33
0.00	1.71	1250.00	3.00	0.00	0.00	1.43	0.71
1.17	0.40	500.00	3.00	11.00	4.67	0.50	1.43
0.57	500.00	3.00	0.00	0.38	0.17	0.17	0.00
0.57	500.00	3.00	0.00	0.00	0.71	0.71	5.33
0.19	0.57	500.00	3.00	0.00	0.19	0.71	0.71
0.00	0.00	1250.00	2.57	0.00	0.00	5.00	0.71
0.10	1.14	500.00	3.00	0.00	0.00	0.03	0.71
0.00	0.02	750.00	3.00	0.00	0.00	2.14	0.33
0.02	500.00	6.00	0.30	0.77	0.71	1.83	2.67
1.14	500.00	3.00	0.30	0.00	0.50	0.00	0.00
0.10	0.27	250.00	3.00	0.00	2.30	10.00	0.08
0.10	0.80	607.14	3.00	3.67	0.38	0.21	0.17
5.00	2.86	1000.00	3.00	0.00	0.58	0.33	0.83
2.00	500.00	4.00	0.30	4.67	10.00	0.67	0.22
2.29	500.00	3.00	1.83	11.67	0.33	1.79	0.22
0.00	0.13	250.00	3.00	0.00	0.00	5.00	2.14
0.00	0.27	500.00	4.50	0.00	0.00	0.71	2.14
2.29	500.00	7.50	11.00	0.96	0.17	0.17	0.44
0.00	1.71	35.71	3.00	0.00	0.00	15.00	0.71
5.00	12.00	1000.00	3.00	0.00	40.00	10.00	10.00
0.58	0.07	750.00	3.00	1.81	1.15	5.00	1.43
1.14	1250.00	3.00	0.00	0.00	2.14	0.71	2.67
0.10	1.71	500.00	3.00	0.00	0.00	0.04	2.14
1.14	500.00	3.00	0.00	0.00	0.01	2.14	0.22
2.33	2.29	500.00	3.00	7.33	0.38	0.17	0.17
0.00	2.86	500.00	3.00	0.00	0.38	0.17	0.17
1.17	0.57	107.14	3.00	0.00	140.00	2.14	5.00
1.14	750.00	3.00	0.00	0.19	0.71	0.71	2.67
0.00	0.57	1250.00	3.00	1.81	0.00	2.14	0.08
0.58	0.03	750.00	3.00	1.81	0.00	2.14	0.08
0.38	0.57	1000.00	3.00	0.30	0.77	0.01	0.71
0.00	0.57	1250.00	3.00	0.00	0.00	15.00	0.71
5.83	2.29	500.00	3.00	18.33	0.58	0.17	0.50
1.71	750.00	3.00	11.00	0.77	0.17	0.17	2.67
1.14	750.00	3.00	0.00	0.19	0.71	0.71	2.67
0.19	0.67	250.00	6.00	100.00	9.33	10.00	1.67
0.48	0.11	250.00	1.50	1.51	0.77	1.43	0.33
0.58	2.29	500.00	3.00	1.83	14.00	0.33	1.79
0.00	0.05	750.00	3.00	0.00	9.33	5.00	0.00
0.00	1.71	35.71	3.00	0.00	0.00	15.00	1.43
10.00	12.00	250.00	9.00	7.33	0.00	0.00	0.17
0.00	1.71	500.00	3.00	0.00	0.38	0.33	0.25
0.00	1.14	1250.00	3.00	0.00	0.00	15.00	0.71
0.00	4.00	750.00	3.00	0.00	0.00	15.00	0.00
0.19	1.14	750.00	3.00	0.00	0.19	0.71	0.71
2.29	375.00	6.00	11.00	4.67	0.17	0.71	0.44

8.00	1250.00	3.00	0.00	0.00	0.25	0.71	0.00
1.33	8.33	3.00	0.00	0.00	1.43	0.53	5.33
1.17	2.29	500.00	3.00	1.83	11.67	0.33	1.79
0.00	4.00	250.00	3.00	0.00	140.00	0.17	0.71
0.00	0.57	1000.00	3.00	0.00	4.67	1.43	0.00
2.29	2500.00	6.00	0.00	0.00	30.00	1.43	0.00
1.17	0.00	250.00	3.00	0.00	0.00	2.14	0.67
0.00	0.67	500.00	0.00	0.00	0.00	0.00	0.17
0.00	0.00	750.00	3.00	0.00	0.00	2.86	0.03
0.00	0.00	500.00	1.29	0.00	0.00	3.57	0.17
2.33	1.14	1250.00	3.00	15.71	0.00	15.00	1.43
0.00	4.00	1250.00	3.00	0.00	0.00	0.33	1.43
0.00	0.00	750.00	0.00	0.00	0.00	5.00	0.71
4.00	1250.00	3.00	0.00	0.00	0.33	2.50	0.00
0.00	1.14	750.00	6.00	0.00	0.00	2.14	0.08
0.29	0.03	750.00	12.00	0.90	0.77	0.33	0.08
0.00	4.00	1250.00	3.00	0.00	0.00	0.33	1.43
2.33	0.04	1250.00	3.00	0.60	9.33	0.05	1.43
0.00	2.86	500.00	3.00	3.67	4.67	0.33	0.17
4.00	1250.00	3.00	0.00	0.00	0.33	1.43	0.00
0.58	2.29	500.00	3.00	1.83	11.67	0.33	1.79
0.00	0.00	750.00	3.00	0.00	140.00	5.00	1.43
0.00	0.27	1250.00	3.00	0.00	0.00	5.00	0.71
3.50	2.86	500.00	3.00	11.00	0.38	0.17	0.67
0.00	1.71	250.00	3.00	0.00	0.00	2.14	0.00
0.00	0.00	750.00	3.00	0.00	60.00	10.00	0.00
0.19	1.14	750.00	3.00	0.00	0.19	0.71	0.71
0.14	0.11	1000.00	3.00	0.00	0.00	2.50	0.25
2.33	4.00	500.00	3.00	7.33	40.00	5.00	0.33
0.00	0.57	500.00	3.00	7.33	0.58	0.17	0.17
0.00	0.00	750.00	3.00	0.00	0.00	15.00	0.00
1.33	8.33	3.00	0.00	0.00	1.43	0.53	5.33
0.29	2.86	250.00	1.50	11.00	9.33	1.43	0.10
0.10	0.02	500.00	6.00	0.30	0.77	0.71	1.83
0.19	0.57	1000.00	1.50	0.30	0.77	0.03	0.33
5.00	0.57	500.00	3.00	0.00	0.38	0.17	0.17
0.10	1.14	500.00	3.00	0.00	0.00	0.01	1.43
5.00	0.57	500.00	3.00	0.00	0.38	0.17	0.17
0.00	4.00	1250.00	1.50	0.60	20.00	5.00	0.33
1.17	1.71	1000.00	3.00	3.67	20.00	0.71	0.07
0.10	0.57	1250.00	0.00	0.00	4.67	5.00	0.71
3.50	2.86	500.00	3.00	11.00	0.38	0.17	0.33
0.00	0.13	1000.00	3.00	0.30	4.67	5.00	0.71
0.10	0.27	1000.00	3.00	0.00	0.00	5.00	1.43
0.29	0.05	500.00	3.00	0.30	0.00	0.17	0.00
0.00	0.11	250.00	3.00	0.00	20.00	0.05	0.17
1.14	1250.00	3.00	0.00	0.00	15.00	0.71	0.00
0.00	0.57	250.00	3.00	0.00	40.00	0.71	0.71
1.17	0.13	1500.00	6.00	0.30	0.00	7.14	7.86
0.00	0.00	1000.00	3.00	0.00	2.30	5.00	2.14
0.00	1.71	35.71	3.00	0.00	0.00	15.00	2.86
0.19	0.57	500.00	3.00	0.00	0.19	0.71	0.71
0.19	0.57	500.00	3.00	0.00	0.19	1.43	0.71

0.00	0.00	750.00	9.00	0.00	2.30	0.00	10.00
0.00	2.86	250.00	3.00	0.00	140.00	0.00	0.00
0.00	8.00	1250.00	1.29	1.51	0.00	5.00	2.14
1.14	1250.00	3.00	0.00	0.00	2.14	0.71	2.67
0.10	0.02	500.00	6.00	0.30	0.77	0.71	1.83
0.00	1.14	750.00	3.00	0.00	0.00	1.43	0.36
2.33	0.27	750.00	9.00	0.30	0.00	2.14	0.00
0.10	0.57	1000.00	3.00	1.81	0.00	5.00	0.71
2.33	2.57	500.00	4.50	11.00	0.38	0.17	0.83
0.00	0.57	250.00	3.00	0.00	0.00	0.00	0.00
2.57	500.00	6.00	11.00	0.38	0.17	0.83	0.22
1.14	1250.00	3.00	31.43	9.33	15.00	3.57	2.67
1.14	500.00	3.00	0.00	0.00	0.71	0.71	6.67
0.19	0.57	500.00	3.00	0.00	0.00	0.71	0.71
2.33	2.29	375.00	6.00	11.00	4.67	0.17	0.71
0.00	0.00	1000.00	3.00	0.00	20.00	5.00	0.17
0.00	0.00	500.00	0.00	0.00	140.00	15.00	0.00
2.86	500.00	3.00	0.00	0.00	0.50	0.00	0.00
0.00	0.27	750.00	3.00	47.14	60.00	5.00	0.04
2.57	0.33	1262.50	12.00	9.17	4.67	11.05	1.60
0.00	0.13	750.00	0.00	0.00	0.00	0.50	0.01
8.00	1250.00	3.00	0.00	0.00	0.33	0.71	0.00
0.00	1.14	1250.00	3.00	0.00	0.00	15.00	0.71
2.29	500.00	3.00	1.83	14.00	0.33	1.79	0.22
0.00	8.00	1250.00	3.00	0.00	0.00	0.17	1.43
0.19	0.02	750.00	3.00	0.00	0.96	0.00	0.00
2.29	500.00	6.00	7.33	4.67	0.42	1.79	0.22
0.00	0.13	750.00	3.00	0.00	0.00	3.57	0.33
1.71	35.71	3.00	0.00	0.00	15.00	1.43	0.00
0.29	0.40	250.00	0.00	0.00	0.77	1.43	0.33
0.00	0.27	0.00	3.00	0.00	0.00	0.00	0.00
1.17	2.29	500.00	6.00	7.33	2.33	0.17	0.71
2.33	2.57	500.00	4.50	11.00	0.58	0.17	0.67
2.29	500.00	6.00	7.33	2.33	0.17	0.71	0.22
5.83	1.71	500.00	3.00	11.00	0.38	0.17	0.33
2.86	500.00	3.00	0.00	0.00	0.50	0.03	0.00
0.58	0.07	750.00	3.00	1.81	2.30	0.00	3.57
0.00	0.27	250.00	0.00	0.00	0.00	0.71	0.03
0.00	1.71	750.00	3.00	3.67	4.67	5.00	0.00
0.10	1.14	500.00	3.00	0.00	0.00	0.03	0.71
2.86	500.00	3.00	7.33	9.33	0.25	0.50	0.00
1.14	750.00	3.00	0.00	0.19	0.71	0.71	2.67
0.19	0.07	1500.00	0.75	0.00	0.00	1.43	5.00
0.00	500.00	3.00	0.00	0.77	0.67	0.33	0.00
2.33	1.14	750.00	3.00	3.67	0.38	0.33	0.33
0.19	1.14	750.00	3.00	0.00	0.38	1.43	0.36
1.17	1.14	107.14	3.00	0.00	0.00	2.14	0.71
1.17	1.71	1000.00	3.00	3.67	20.00	0.71	0.07
5.00	0.57	500.00	3.00	7.33	0.38	0.00	0.17
0.19	0.02	250.00	0.00	3.67	0.00	0.71	0.71
0.00	0.00	250.00	3.00	0.00	0.00	5.00	0.00
0.58	0.27	1000.00	6.00	0.90	0.00	3.57	2.14
2.29	500.00	7.50	11.00	0.96	0.17	0.83	0.44

0.00	0.00	750.00	3.00	0.00	0.00	5.00	0.00
1.17	0.57	142.86	3.00	2.41	0.77	5.00	0.05
0.00	0.57	750.00	3.00	0.00	0.00	10.00	0.00
8.00	1250.00	3.00	0.00	0.00	0.71	0.71	0.00
0.00	0.00	25.00	3.00	0.00	0.00	5.00	0.04
0.57	500.00	3.00	18.33	0.38	0.17	0.83	0.00
1.17	0.57	500.00	3.00	18.33	0.38	0.17	0.17
0.00	1.71	750.00	3.00	0.00	0.00	1.43	0.00
1.17	1.14	250.00	0.00	1.81	2.30	5.00	5.00
0.57	500.00	3.00	18.33	0.38	0.17	0.17	0.00
1.14	1000.00	3.00	0.00	0.38	0.33	0.83	0.00
1.17	2.29	500.00	6.00	7.33	4.67	0.42	1.79
0.00	0.05	750.00	3.00	0.00	14.00	0.50	0.33
0.00	0.27	250.00	3.00	0.00	0.00	0.00	0.07
0.19	0.57	500.00	3.00	0.00	0.19	0.71	0.71
0.00	0.27	750.00	2.14	0.00	0.00	3.57	0.33
0.00	1.71	1250.00	3.00	0.00	0.00	1.43	0.71
0.10	0.02	500.00	6.00	0.30	0.77	0.71	1.83
0.00	0.13	1000.00	3.00	0.30	4.67	5.00	0.71
0.24	0.57	500.00	1.50	0.60	0.00	7.50	0.02
0.19	1.14	750.00	3.00	0.00	0.38	1.43	0.36
1.17	0.13	1250.00	3.00	1.83	0.00	2.14	1.43
0.19	0.57	1000.00	1.50	2.41	4.67	3.00	0.67
0.00	0.57	500.00	3.00	11.00	0.00	5.00	1.43
0.00	1.14	750.00	3.00	0.00	0.00	1.43	0.36
0.00	8.00	1250.00	3.00	0.00	0.00	0.67	0.71
0.29	2.86	500.00	3.00	0.30	0.00	0.50	0.00
0.00	500.00	3.00	0.00	0.77	0.17	0.17	0.00
0.00	8.00	1250.00	3.00	0.00	0.00	1.07	2.14
0.00	0.57	500.00	3.00	0.00	0.00	15.00	0.71
2.33	0.07	750.00	3.00	0.60	9.33	5.00	0.08
0.29	2.86	500.00	3.00	0.00	0.00	0.50	0.03
0.00	0.00	1250.00	0.00	0.00	0.00	5.00	0.00
0.19	0.57	500.00	3.00	0.00	0.19	1.43	1.43
0.00	0.13	250.00	3.00	0.00	4.67	0.00	0.03
2.33	0.57	500.00	3.00	18.33	0.38	0.17	0.17
0.10	1.14	500.00	3.00	0.00	0.00	0.01	0.71
0.00	2.57	500.00	3.00	0.00	0.00	5.00	0.01
0.00	1.14	1250.00	3.00	0.00	0.00	2.14	0.71
0.00	0.00	750.00	9.00	0.00	60.00	3.57	0.17
0.00	4.00	250.00	3.00	0.00	140.00	5.00	0.00
0.00	8.00	250.00	3.00	1.81	0.00	5.00	0.00
0.10	1.71	500.00	3.00	0.30	0.38	0.01	0.71
5.15	0.60	1275.00	10.00	18.33	9.33	21.76	2.48
5.83	2.29	500.00	3.00	18.33	0.58	0.17	0.75
0.00	0.00	500.00	3.00	0.00	0.00	5.00	5.00
0.29	1.14	500.00	3.00	0.30	0.00	0.50	0.00
0.57	500.00	3.00	0.00	0.00	0.71	0.71	5.33
0.00	0.57	750.00	6.00	0.00	0.00	0.17	0.00
0.19	0.57	500.00	3.00	0.00	0.00	0.00	0.03
0.29	2.86	500.00	3.00	0.00	0.00	0.50	0.03
0.00	8.00	1250.00	3.00	0.00	0.00	1.43	1.43
0.00	0.00	250.00	0.00	0.00	0.00	0.17	0.71

0.00	0.13	750.00	3.00	0.00	0.00	5.00	0.00
0.00	0.27	250.00	0.00	0.00	0.00	3.57	0.00
0.57	500.00	3.00	0.00	0.19	0.71	0.36	8.00
0.00	0.57	500.00	3.00	18.33	0.38	0.17	0.33
0.00	8.00	1250.00	3.00	0.00	0.00	0.17	1.43
0.00	8.00	1250.00	3.00	0.00	0.00	1.43	0.71
1.17	0.13	1500.00	6.00	0.30	0.00	7.14	7.86
0.00	4.00	750.00	3.00	0.00	0.00	0.00	0.71
0.10	0.01	500.00	3.00	0.00	0.77	0.17	0.33
0.00	1.14	1250.00	3.00	0.00	0.00	15.00	0.71
5.83	2.29	500.00	3.00	11.00	0.38	0.17	0.33
2.33	2.29	250.00	6.00	11.00	4.67	0.17	0.71
0.19	1.14	500.00	3.00	0.00	0.00	0.71	0.71
0.19	1.14	750.00	3.00	0.00	0.00	1.43	0.36
0.00	4.00	250.00	3.00	0.00	0.00	0.00	0.00
0.00	2.29	250.00	1.29	0.00	0.00	0.71	0.17
0.00	8.00	1250.00	3.00	0.00	0.00	0.25	0.71
0.10	0.02	500.00	6.00	0.30	0.77	0.71	1.83
0.19	1.14	750.00	3.00	0.00	0.38	0.71	0.71
1.17	0.05	250.00	3.00	1.81	1.53	1.43	0.17
1.14	750.00	3.00	0.00	0.19	0.71	0.71	5.33
1.17	0.57	500.00	3.00	18.33	0.38	0.17	0.17
0.00	4.00	250.00	3.00	0.00	60.00	3.57	0.17
0.00	0.00	750.00	9.00	0.00	0.00	2.14	10.00
0.00	1.14	1250.00	3.00	0.00	0.00	2.14	0.71
0.00	0.57	500.00	3.00	6.03	0.00	5.00	0.03
2.33	2.86	500.00	6.00	11.00	4.67	0.17	0.71
0.00	4.00	750.00	3.00	0.90	140.00	5.00	0.05
0.00	0.57	750.00	3.00	0.00	0.00	1.43	0.50
0.00	0.00	750.00	6.00	0.00	0.00	1.43	0.00
0.00	0.00	1000.00	3.00	0.00	0.00	10.00	5.00

gharch	halva	halvarde	advie	weight.f	kcal	protein	cho
34.29	0.33	0.25	1.29	2819.81	1772.43	74.97	274.84
2.67	0.00	0.50	0.86	#NULL!	1882.83	75.67	288.43
0.00	0.00	4.29	3.00	#NULL!	2421.79	88.03	326.36
5.33	0.00	0.50	0.86	#NULL!	1680.47	56.80	231.17
5.33	0.22	0.00	12.00	2150.05	1875.53	65.72	287.10
11.43	0.00	0.00	3.00	2604.60	2495.25	100.27	397.95
8.00	0.00	1.50	0.86	#NULL!	1949.56	66.76	283.68
0.00	0.11	0.00	3.00	2325.46	2135.41	90.82	308.44
0.00	0.50	0.86	#NULL!	2030.65	67.30	296.88	70.00
2.67	0.00	0.50	3.00	1646.91	1981.45	72.77	299.64
0.11	0.50	0.86	#NULL!	2759.67	86.00	341.63	124.18
8.00	0.67	1.50	3.00	3573.22	2634.23	103.37	401.09
0.67	2.50	1.29	#NULL!	1576.38	83.75	210.24	49.66
11.43	0.33	0.00	3.00	1509.58	1772.19	59.37	260.03
0.00	1.00	0.00	3.00	#NULL!	1742.24	52.27	205.20
2.67	0.00	0.50	0.86	#NULL!	1778.95	63.69	248.10
0.44	1.33	0.04	0.86	#NULL!	2732.87	74.92	288.78
0.00	0.33	0.50	3.00	2142.38	1559.12	59.20	243.22
5.33	0.33	0.00	3.00	2399.76	1651.78	48.73	253.86
0.22	0.67	0.04	0.43	#NULL!	2624.54	73.52	288.53
0.00	0.00	2.14	3.00	#NULL!	1943.10	71.37	263.96
6.67	0.33	0.25	0.64	#NULL!	2616.30	78.37	308.53
11.43	0.05	0.08	6.00	2509.22	1798.70	62.92	290.59
0.66	0.05	0.08	3.00	1887.34	2132.26	78.12	346.12
2.67	0.00	0.50	0.86	#NULL!	1882.83	75.67	288.43
0.33	0.08	1.50	0.43	#NULL!	2613.16	88.17	346.37
22.86	0.00	0.00	9.00	2857.29	2102.84	85.73	305.30
45.71	0.00	0.00	2.14	3111.05	2340.29	88.87	336.73
0.22	1.67	0.04	0.64	#NULL!	2608.16	79.98	234.29
0.00	0.22	0.50	0.86	#NULL!	2191.91	67.89	259.46
0.00	0.11	1.00	0.43	#NULL!	2303.98	67.35	288.75
0.33	1.00	0.75	0.43	#NULL!	2817.73	98.68	352.00
8.00	0.00	1.50	0.86	#NULL!	1910.04	70.85	264.83
5.33	0.00	0.00	3.00	2897.88	2370.59	75.99	386.89
2.86	2.14	0.86	#NULL!	1889.08	75.03	251.11	71.42
0.00	0.00	1.07	#NULL!	1459.83	59.63	217.20	48.15
0.00	0.00	6.43	3.00	1998.68	1740.58	69.66	266.73
1.32	0.22	0.16	3.00	2549.83	1821.02	76.18	288.83
0.00	0.00	2.14	3.00	#NULL!	1448.54	53.80	167.70
11.43	0.11	1.50	3.00	#NULL!	1611.68	64.81	225.58
0.00	0.00	0.00	3.00	#NULL!	2534.34	55.73	292.06
0.55	0.00	0.00	1.29	#NULL!	2528.42	92.28	311.79
0.00	0.67	0.08	0.64	#NULL!	2747.15	80.31	307.22
2.67	0.00	0.50	6.00	2640.78	1867.80	59.20	286.91
22.86	0.00	0.00	3.00	2625.24	2103.48	76.58	325.83
11.43	0.22	0.00	0.86	1890.90	1508.54	57.44	249.60
2.67	0.05	0.04	3.00	2020.34	2016.16	63.84	310.35
0.05	1.00	0.43	#NULL!	2286.41	78.53	307.47	86.34
1.32	0.67	0.50	3.00	1830.51	1684.71	56.04	279.36
0.67	5.50	1.50	#NULL!	2777.37	93.18	415.76	95.19
1.32	0.11	0.25	3.00	2732.51	2805.00	140.67	388.14
5.33	0.00	0.50	0.86	#NULL!	1797.76	60.45	252.70

5.33	0.33	0.00	3.00	1701.86	1649.00	66.68	263.45
0.00	0.00	4.29	6.00	#NULL!	2151.93	84.01	299.59
22.86	0.00	0.00	6.00	1528.66	1832.63	56.12	274.69
0.00	0.50	1.07	#NULL!	1290.48	50.93	189.92	42.65
0.00	0.50	3.00	#NULL!	2328.99	73.61	254.91	121.67
0.00	0.00	1.00	2.36	3371.78	2844.80	121.02	442.55
0.00	0.50	0.86	#NULL!	1680.47	56.80	231.17	65.19
0.00	0.00	0.00	1.29	#NULL!	2747.18	84.58	377.19
5.33	0.00	1.00	3.00	#NULL!	2101.47	62.07	258.28
0.67	0.06	0.43	#NULL!	2786.88	93.44	335.29	125.18
0.00	0.00	0.86	#NULL!	1420.25	63.79	206.51	46.86
6.67	0.00	0.50	1.07	#NULL!	1290.48	50.93	189.92
11.43	0.00	0.00	3.00	3075.69	2378.56	95.33	373.55
34.29	2.86	1.00	1.29	#NULL!	2599.78	128.11	394.42
5.33	0.11	0.08	3.00	2051.21	1871.14	56.74	252.01
0.67	2.50	1.29	#NULL!	1576.38	83.75	210.24	49.66
2.00	0.00	1.29	#NULL!	1677.56	78.23	232.02	59.52
1.32	0.27	0.21	3.00	1747.95	1634.24	56.28	257.76
11.43	0.11	1.50	3.00	#NULL!	1611.68	64.81	225.58
0.00	2.67	0.08	0.43	#NULL!	2808.82	93.89	362.83
0.00	0.00	9.00	#NULL!	3035.37	85.99	419.96	128.33
1.67	0.04	0.64	#NULL!	2047.33	91.03	270.07	71.44
0.00	0.05	0.04	3.00	1264.74	1282.43	38.94	222.55
22.86	0.67	1.00	6.00	2030.75	1783.21	67.85	284.33
0.14	0.25	1.29	#NULL!	2967.97	107.35	410.26	110.35
0.00	2.86	2.14	6.00	#NULL!	3104.20	121.52	463.21
6.67	0.00	0.00	3.00	2357.67	1964.49	71.60	269.59
7.89	0.33	0.25	1.29	2466.72	2542.80	86.93	378.59
0.00	2.14	0.86	#NULL!	1839.43	78.05	232.20	72.65
34.29	8.57	1.00	1.29	#NULL!	2656.26	116.98	430.17
2.86	0.50	0.64	#NULL!	2680.65	123.71	401.94	76.72
0.22	0.67	0.50	1.07	#NULL!	2550.41	88.80	310.36
1.33	0.33	0.00	0.64	#NULL!	2506.41	77.70	323.57
34.29	0.05	0.04	3.00	2539.59	2963.98	139.92	422.96
0.00	0.50	0.86	#NULL!	2060.43	77.89	296.20	71.36
5.33	0.33	0.00	3.00	2794.83	2273.30	92.95	358.52
5.33	0.33	0.25	3.00	1977.40	1463.12	69.00	226.75
2.67	0.22	0.08	1.29	2975.09	2345.91	86.50	372.08
0.00	1.00	3.21	3.00	#NULL!	1645.68	50.04	183.30
0.00	0.05	0.50	0.43	#NULL!	2230.08	76.75	270.46
0.27	2.50	1.07	#NULL!	2834.98	89.98	308.82	143.34
0.00	0.50	1.29	#NULL!	1750.49	62.59	245.59	64.08
22.86	0.33	0.25	3.00	1953.93	2972.59	73.28	377.71
2.67	0.55	0.00	3.00	#NULL!	1856.04	63.45	291.34
0.22	0.67	0.04	1.50	#NULL!	1820.20	83.50	246.05
5.33	1.33	0.25	3.00	2634.30	2189.04	81.46	334.20
0.00	0.00	4.29	6.00	#NULL!	2049.59	76.23	289.93
0.00	0.00	0.00	3.00	2002.21	2106.87	61.70	336.57
0.00	0.00	0.50	1.29	#NULL!	2675.89	79.91	343.16
0.00	0.00	2.14	3.00	#NULL!	2367.67	86.13	367.22
71.43	20.00	0.00	3.00	2158.39	1938.48	54.71	280.64
2.67	0.00	0.50	0.86	#NULL!	2060.43	77.89	296.20
1.33	0.04	0.86	#NULL!	2615.41	74.05	311.25	122.02

0.00	1.00	3.00	#NULL!	2762.96	96.33	324.89	125.15
0.00	0.04	3.00	#NULL!	1910.75	92.26	235.57	72.36
0.22	1.67	0.04	0.64	#NULL!	1999.14	86.11	251.66
11.43	0.00	11.00	3.00	2501.08	2388.40	85.41	322.71
22.86	0.11	0.00	3.00	2908.60	2442.07	87.26	348.38
0.00	9.64	6.00	#NULL!	1837.85	73.52	245.09	66.44
0.22	0.00	11.00	3.00	1353.23	2093.36	83.40	262.00
1.32	0.00	0.00	3.00	1944.23	1520.65	65.42	249.40
8.00	0.67	0.00	6.00	1927.82	1498.35	50.96	219.60
6.67	0.00	0.00	6.00	3256.61	2482.34	82.62	414.46
0.00	0.00	0.00	0.43	#NULL!	2936.16	99.44	398.37
0.00	1.33	0.00	3.00	#NULL!	2612.05	89.14	281.60
11.43	0.67	0.50	3.00	3657.66	2791.27	99.73	489.48
2.00	0.00	4.50	#NULL!	2872.97	88.21	364.88	124.41
5.33	0.00	1.00	7.50	1968.00	1721.45	70.83	263.32
11.43	0.16	0.12	9.00	3206.04	2367.64	91.94	384.97
0.00	1.33	0.00	3.00	#NULL!	2157.84	54.46	200.79
2.67	0.11	0.08	6.00	2597.45	1502.85	56.50	212.75
11.43	0.19	0.50	3.00	2249.78	1881.84	69.10	280.01
1.33	0.00	3.00	#NULL!	2362.11	69.01	286.41	112.60
0.22	1.67	0.04	0.64	#NULL!	2216.98	92.32	300.84
80.00	0.00	0.00	3.00	3181.90	2398.79	102.28	375.84
5.33	0.22	0.00	3.00	3497.83	2645.15	91.67	417.18
0.00	0.05	0.50	0.43	#NULL!	2406.06	68.21	320.42
2.67	0.00	0.08	3.00	1690.05	1999.69	66.54	294.87
0.00	0.00	0.00	3.00	3080.20	2112.12	101.32	324.19
2.67	0.00	0.50	0.86	#NULL!	2130.55	86.73	316.15
4.00	0.08	3.21	3.00	2738.46	2012.39	79.74	310.95
11.43	0.11	0.00	3.00	2293.07	1593.73	67.02	244.94
0.00	0.67	0.08	0.43	#NULL!	3166.69	82.22	397.43
0.00	0.67	1.00	6.00	2934.34	2365.38	87.18	341.84
0.00	0.04	3.00	#NULL!	1910.75	92.26	235.57	72.36
11.43	0.38	0.00	1.50	#NULL!	1642.10	61.97	210.21
2.67	0.67	2.50	1.29	#NULL!	1576.38	83.75	210.24
11.43	0.05	0.08	3.00	3242.15	2046.85	81.40	305.63
0.00	1.33	0.04	0.43	#NULL!	2808.70	91.42	336.17
22.86	2.86	0.50	1.29	#NULL!	2379.44	105.33	346.23
0.00	1.33	0.06	0.43	#NULL!	3070.05	99.98	380.02
11.43	0.00	0.04	6.00	2979.63	2148.07	79.33	341.24
2.67	0.67	1.50	3.00	#NULL!	1648.61	63.15	231.26
22.86	0.11	0.00	3.00	3867.60	2792.92	91.02	481.52
0.00	0.05	1.25	0.43	#NULL!	2429.87	78.56	314.59
2.67	2.91	0.00	3.00	3235.26	2127.26	78.29	310.36
11.43	0.00	0.00	3.00	4168.50	2403.85	117.07	334.81
2.67	0.11	0.04	3.00	1613.59	1367.93	49.40	200.15
2.67	0.00	0.50	3.00	1932.83	1844.77	58.56	294.03
0.00	1.07	3.00	#NULL!	1408.59	46.73	202.96	46.72
11.43	0.67	0.50	3.00	2866.57	3052.81	105.82	398.78
5.33	0.66	15.00	1.50	#NULL!	2289.52	74.57	295.29
5.33	0.33	2.14	3.00	2791.57	2258.12	75.01	318.62
0.00	0.00	8.57	6.00	#NULL!	2439.63	96.78	340.77
6.67	0.00	0.50	1.07	#NULL!	1551.07	69.61	223.98
4.00	0.00	0.00	1.07	#NULL!	1391.84	59.73	189.99

1.32	0.33	0.25	3.00	2049.15	1752.40	72.00	272.79
5.33	0.00	0.00	3.00	2482.46	2694.00	115.06	362.78
22.86	10.00	11.00	6.00	3549.55	3014.63	103.22	461.19
0.00	2.14	0.86	#NULL!	1839.43	78.05	232.20	72.65
2.67	0.67	2.50	1.29	#NULL!	1576.38	83.75	210.24
8.00	0.00	0.50	0.86	#NULL!	2080.04	77.11	297.37
5.33	1.33	0.00	7.50	2087.17	2119.39	66.50	327.28
2.67	0.33	0.25	3.00	2351.62	2006.25	73.99	316.23
0.22	0.11	1.50	0.43	#NULL!	2458.21	86.21	303.96
0.00	0.22	0.16	3.00	2833.87	2596.33	104.06	365.80
0.05	0.50	1.07	#NULL!	2524.31	88.51	329.88	100.52
2.86	8.57	3.00	#NULL!	2881.24	76.99	300.42	153.69
0.00	0.00	0.86	#NULL!	1411.82	61.43	203.89	47.21
6.67	0.00	0.00	0.86	#NULL!	1341.32	58.84	203.97
0.44	1.33	0.04	0.86	#NULL!	2662.50	76.16	318.32
2.67	0.00	0.16	3.00	4810.48	3166.91	131.89	476.88
2.67	0.00	0.00	6.00	3202.51	2201.52	89.72	343.96
0.00	0.00	0.86	#NULL!	1773.14	89.41	229.86	66.97
34.29	0.05	0.04	3.00	2491.78	2197.32	92.39	282.98
32.55	1.22	0.58	9.00	2747.85	2011.79	76.49	308.82
22.86	0.05	4.29	6.00	3193.03	2358.95	92.74	358.43
0.00	1.25	3.00	#NULL!	2531.96	75.91	298.50	125.03
0.00	0.00	8.57	3.00	#NULL!	2152.53	91.82	274.84
0.67	0.04	1.50	#NULL!	1768.41	83.01	232.81	61.42
0.00	1.00	0.00	3.00	#NULL!	2171.69	74.08	281.80
4.00	0.00	0.00	1.29	2603.93	1836.06	52.00	307.16
1.67	0.04	0.64	#NULL!	2577.52	80.86	250.87	143.72
5.33	0.22	0.12	3.00	2833.38	2773.81	93.67	439.15
0.00	4.29	6.00	#NULL!	1948.79	77.99	255.76	70.83
0.00	0.16	1.00	3.00	1530.95	1706.55	73.14	251.28
0.44	0.00	0.00	1.71	1246.80	1363.89	60.13	196.20
0.22	0.67	0.04	0.64	#NULL!	2279.58	66.85	198.54
0.00	0.05	1.50	0.64	#NULL!	2477.65	82.45	316.14
0.67	0.04	0.64	#NULL!	2140.03	69.04	218.28	113.05
0.00	0.05	0.50	0.43	#NULL!	2459.08	84.24	333.40
0.00	0.50	1.07	#NULL!	1696.96	81.43	224.19	63.82
5.33	0.33	0.25	3.00	2127.32	1788.19	63.02	241.65
4.00	0.00	0.08	9.00	1598.69	1344.09	57.56	231.21
11.43	0.22	0.08	6.00	2621.99	2138.13	71.14	318.80
57.14	2.86	1.00	1.29	#NULL!	2662.45	123.38	406.95
0.67	0.25	1.07	#NULL!	2963.28	95.57	353.03	136.42
0.00	0.50	1.29	#NULL!	1840.58	64.77	252.50	70.59
0.44	0.22	0.08	6.00	3056.82	1922.30	51.79	317.14
5.71	0.00	0.43	#NULL!	2727.90	77.96	357.02	116.33
5.33	0.16	1.00	0.86	#NULL!	2653.23	93.71	321.42
2.67	0.00	0.50	0.86	#NULL!	1699.65	55.83	238.12
11.43	2.86	0.50	3.00	4810.48	1433.36	50.54	238.79
2.67	0.67	1.50	3.00	#NULL!	1648.61	63.15	231.26
0.00	0.67	0.21	0.43	#NULL!	3045.09	79.25	354.19
0.00	0.00	0.00	3.00	2102.55	2577.47	86.50	435.24
0.00	0.00	0.00	3.00	2611.85	2341.42	57.86	338.27
1.32	0.05	0.00	3.00	3715.07	2093.80	78.70	315.94
0.03	0.25	1.29	#NULL!	2603.78	91.63	348.28	101.55

0.00	0.11	0.08	3.00	2100.13	1767.38	58.60	256.93
0.00	2.86	1.00	3.00	1639.59	2102.29	73.77	304.44
11.43	0.00	0.50	1.29	3260.38	2488.82	99.84	361.47
0.00	0.00	3.00	#NULL!	2313.81	67.33	297.38	96.31
2.67	0.16	0.00	3.00	1641.31	1977.92	75.58	326.58
2.86	4.33	0.43	#NULL!	2814.67	70.40	311.37	148.61
0.00	0.00	0.00	0.86	#NULL!	2939.61	102.91	366.80
11.43	0.00	0.00	3.00	2315.69	1968.27	59.35	266.99
5.33	0.16	0.00	6.00	1759.15	2261.20	79.83	329.83
0.30	0.75	0.86	#NULL!	2827.35	95.75	396.36	103.89
1.67	0.06	0.43	#NULL!	2912.30	99.59	369.65	123.69
0.22	1.67	0.04	0.64	#NULL!	2434.39	69.61	208.30
5.33	0.27	0.21	3.00	2419.34	1864.32	78.03	281.76
1.32	0.05	0.00	3.00	1955.27	2169.60	74.82	340.16
6.67	0.00	0.50	0.86	#NULL!	1505.28	61.35	232.04
5.33	0.00	1.00	9.00	2572.49	2343.99	85.36	360.32
0.00	0.00	0.00	1.29	#NULL!	2454.48	73.39	284.58
2.67	0.67	2.50	1.29	#NULL!	1576.38	83.75	210.24
2.67	2.91	0.00	3.00	3235.26	2127.26	78.29	310.36
2.67	0.67	0.04	3.00	2273.43	2490.01	86.96	329.05
2.67	0.00	0.50	0.86	#NULL!	1699.65	55.83	238.12
22.86	0.16	0.12	3.00	2786.35	2060.50	83.45	322.50
0.22	0.55	3.00	0.50	#NULL!	1687.97	49.51	261.11
11.43	0.67	0.50	3.00	2429.78	2357.59	91.88	349.09
8.00	0.00	0.50	0.86	#NULL!	2080.04	77.11	297.37
0.00	0.00	0.00	3.00	#NULL!	2491.36	72.64	302.65
0.00	2.00	0.00	1.29	#NULL!	1730.97	82.74	236.70
0.67	0.00	0.43	#NULL!	3198.03	81.34	401.89	145.94
0.00	0.00	0.00	6.00	#NULL!	3131.87	94.31	465.05
0.00	1.33	1.00	0.86	#NULL!	1741.71	55.17	238.32
80.00	0.00	0.00	3.00	3022.70	2650.27	125.67	372.48
0.00	0.00	0.50	1.07	#NULL!	1696.96	81.43	224.19
11.43	0.00	0.00	3.00	2706.27	1637.31	64.05	281.06
10.67	0.00	0.00	1.07	#NULL!	1459.83	59.63	217.20
0.88	0.05	0.04	3.00	2219.09	1968.15	82.01	313.52
0.44	0.14	0.25	0.86	#NULL!	2857.06	93.20	411.96
11.43	2.86	0.50	0.86	#NULL!	3012.48	134.52	465.50
22.86	0.16	0.00	3.00	2290.55	2371.04	85.04	409.20
0.00	0.00	4.29	3.00	#NULL!	1786.40	75.59	221.56
2.67	0.00	0.00	3.00	2966.58	2393.37	82.97	413.99
11.43	0.00	0.00	3.00	2993.20	2820.41	109.11	426.16
2.67	0.05	0.00	3.00	2379.64	2536.42	100.79	408.46
11.43	2.86	1.00	1.71	#NULL!	2623.24	125.56	408.24
63.79	2.33	1.08	15.00	2950.92	1807.71	68.97	289.25
0.00	0.05	0.75	0.43	#NULL!	2205.11	70.17	288.02
45.71	0.00	0.00	3.00	2149.37	1779.49	61.91	269.11
0.00	2.00	0.00	1.29	#NULL!	1677.56	78.23	232.02
0.00	0.00	0.86	#NULL!	1420.25	63.79	206.51	46.86
2.67	0.00	0.00	3.00	2390.40	2082.33	48.25	318.31
9.33	0.11	0.00	3.00	2101.46	2090.05	85.77	296.00
0.00	0.00	0.50	1.07	#NULL!	2091.26	98.19	295.24
0.00	0.00	0.00	6.00	#NULL!	2566.19	87.59	327.41
0.00	0.00	4.29	1.29	1951.84	2081.11	95.69	277.47

2.67	0.00	0.00	1.29	1985.43	1657.55	70.43	259.02
5.33	0.00	1.00	3.00	2725.98	2351.18	97.20	352.90
0.00	0.50	1.07	#NULL!	1561.43	63.23	234.88	49.59
0.00	0.00	0.04	0.43	#NULL!	2302.73	59.75	281.05
0.00	1.00	0.00	3.00	#NULL!	2784.74	92.59	383.84
0.00	0.00	0.00	3.00	#NULL!	2819.50	84.48	405.11
5.33	0.66	15.00	1.50	#NULL!	2289.52	74.57	295.29
11.43	0.11	0.08	3.00	2616.75	2124.69	79.76	330.74
11.43	0.11	0.00	9.00	1916.32	1630.94	62.99	243.52
0.00	0.00	4.29	3.00	#NULL!	2421.79	88.03	326.36
0.00	0.05	1.00	0.43	#NULL!	2461.44	82.39	348.23
0.44	1.33	0.04	0.86	#NULL!	2790.61	76.05	322.63
2.67	0.00	0.50	0.86	#NULL!	1430.22	60.91	201.62
8.00	0.00	1.50	0.86	#NULL!	1938.52	73.14	268.54
11.43	1.33	1.00	3.00	2868.28	3165.77	115.00	461.70
11.43	0.11	0.00	3.00	1648.16	1991.35	83.13	288.84
0.00	1.67	0.00	3.00	#NULL!	3282.55	103.39	473.32
2.67	0.67	2.50	1.29	#NULL!	1576.38	83.75	210.24
5.33	0.00	0.50	0.86	#NULL!	1797.76	60.45	252.70
2.67	0.33	4.29	3.00	1764.65	1820.99	58.18	288.97
0.00	0.50	0.86	#NULL!	2030.65	67.30	296.88	70.00
0.00	0.05	1.00	0.86	#NULL!	2862.14	93.91	415.09
8.00	0.05	0.04	0.01	2338.96	2227.50	80.67	374.43
8.00	0.22	0.00	3.00	2434.83	2088.93	77.52	324.17
0.00	0.00	4.29	3.00	#NULL!	1786.40	75.59	221.56
5.33	0.11	0.04	3.00	2579.83	2316.16	93.76	363.22
0.44	1.33	0.04	0.86	#NULL!	2667.65	80.50	311.10
5.33	0.16	0.16	3.00	2694.57	2262.26	80.27	337.01
5.33	0.00	0.12	6.00	2194.75	1803.94	58.79	261.02
2.67	0.00	0.50	3.00	2280.69	1784.39	58.63	253.11
22.86	0.00	0.00	1.71	3338.46	2895.28	122.84	445.90

t.fat	chol	s.fat	m.s.fat	pufa	oleic	linoleic	linoleni
50.74	208.80	16.67	16.78	11.16	14.56	9.64	0.43
60.07	163.31	13.38	15.84	8.48	14.03	6.79	0.62
83.12	175.96	18.41	20.57	18.49	17.58	15.51	1.36
65.19	171.02	17.83	16.78	8.28	14.19	6.44	0.59
53.79	160.33	19.39	19.52	10.78	16.82	8.95	0.65
69.22	118.29	18.58	24.55	18.48	22.31	16.18	1.13
68.81	202.24	17.54	18.48	9.45	15.87	7.52	0.58
65.37	187.52	26.53	18.17	13.98	15.15	10.44	1.88
196.74	19.35	18.51	9.54	15.89	7.63	0.62	0.01
58.38	116.88	23.04	17.90	12.10	15.89	9.59	1.67
301.85	48.42	42.23	21.32	38.70	18.38	1.75	0.03
80.34	226.15	27.09	27.75	16.66	25.08	14.40	0.98
238.20	19.61	15.68	6.03	13.76	4.51	0.65	0.05
41.02	100.77	12.20	13.63	8.88	11.89	7.60	0.35
86.37	104.54	22.10	34.86	22.33	32.55	20.19	1.48
67.00	206.05	19.45	17.13	7.59	14.91	6.04	0.51
145.92	300.59	40.66	47.37	30.59	44.75	27.63	2.11
44.88	117.96	14.85	15.66	9.87	14.35	8.12	0.83
54.45	78.56	13.85	13.77	11.29	12.87	9.95	0.94
134.41	257.37	44.33	43.79	25.30	40.65	22.47	1.84
73.67	176.77	20.67	21.43	13.64	20.11	12.57	0.83
124.52	392.86	50.80	41.88	21.49	38.17	18.76	1.92
52.13	135.65	18.06	19.71	9.17	17.87	7.67	0.55
58.27	100.41	17.49	19.49	15.85	18.13	14.13	0.98
60.07	163.31	13.38	15.84	8.48	14.03	6.79	0.62
102.38	330.51	41.29	35.22	16.76	30.47	13.55	1.30
71.00	224.96	25.02	27.60	12.29	25.15	10.23	0.80
81.82	211.36	23.99	22.44	12.30	19.94	10.15	1.16
154.46	324.33	47.15	52.68	32.50	49.19	28.87	2.54
101.40	279.23	48.06	32.74	10.87	28.07	8.46	1.03
101.62	383.91	47.17	32.83	12.17	28.33	9.72	1.20
118.98	349.56	43.02	41.24	23.48	36.24	19.85	1.86
71.50	207.45	18.75	19.21	9.94	16.43	7.94	0.59
69.19	96.85	16.32	21.60	13.90	19.84	12.00	0.78
254.61	16.95	19.49	11.30	16.72	8.77	0.78	0.06
119.20	15.37	14.89	8.04	12.48	6.32	0.47	0.01
48.49	143.08	14.03	14.88	11.84	12.71	9.67	0.84
48.19	223.48	17.85	16.66	8.56	14.35	7.05	0.34
62.75	157.17	12.16	13.77	12.46	12.12	10.74	0.95
54.44	211.15	15.60	18.18	14.89	16.94	12.73	1.16
129.83	145.16	35.71	49.45	33.08	45.52	29.79	2.39
107.64	383.97	36.12	39.40	20.08	34.49	16.43	1.52
137.94	419.32	53.10	42.74	20.57	39.30	17.71	2.11
57.60	172.14	18.31	19.69	14.93	17.93	13.02	1.21
66.42	185.49	21.28	24.24	12.99	21.30	10.84	0.84
37.64	78.48	12.11	13.87	7.96	12.67	7.07	0.27
65.24	139.82	18.91	19.54	10.56	17.47	8.51	0.97
339.40	36.04	28.58	12.81	24.16	0.00	0.00	0.00
42.92	74.49	12.76	10.48	10.07	9.42	8.83	0.67
456.23	30.14	31.29	17.05	27.91	14.53	0.94	0.09
84.68	245.63	29.10	26.34	21.66	21.92	17.41	1.93
66.14	192.95	18.76	17.09	8.13	14.37	6.31	0.57

42.60	167.12	15.68	14.47	7.63	13.43	6.89	0.38
73.62	168.46	17.20	21.55	14.73	18.04	11.85	0.91
60.78	82.85	15.06	20.25	18.10	19.10	15.85	1.63
98.77	12.84	12.64	7.70	10.61	6.13	0.47	0.01
221.60	27.28	48.84	33.19	45.60	29.69	2.16	0.00
79.12	239.65	26.81	29.60	12.72	27.09	10.55	0.68
171.02	17.83	16.78	8.28	14.19	6.44	0.59	0.01
106.90	176.74	24.23	42.90	27.72	40.73	25.01	1.52
94.95	107.20	24.23	35.23	27.09	33.76	24.88	1.72
443.24	45.07	39.54	21.93	35.28	18.52	1.86	0.03
178.57	15.71	15.95	8.26	13.51	6.51	0.47	0.01
42.65	98.77	12.84	12.64	7.70	10.61	6.13	0.47
59.58	158.27	22.38	18.26	13.32	16.44	11.55	1.05
73.76	274.68	21.00	30.16	14.79	26.11	10.38	1.04
73.11	107.84	18.27	19.77	13.53	17.60	11.43	1.00
238.20	19.61	15.68	6.03	13.76	4.51	0.65	0.05
222.33	22.10	20.10	9.48	18.20	7.83	0.73	0.01
46.19	188.34	12.40	17.02	11.99	15.22	10.32	0.43
54.44	211.15	15.60	18.18	14.89	16.94	12.73	1.16
117.96	328.55	41.15	38.43	22.20	33.88	18.55	1.99
444.00	35.50	53.65	28.18	50.63	24.63	0.92	0.00
319.02	23.88	22.31	13.09	18.18	9.84	0.89	0.01
30.92	74.36	11.10	10.41	6.69	9.85	6.06	0.33
48.03	153.16	15.08	14.93	11.74	13.10	9.91	0.84
368.97	41.12	36.50	19.40	31.64	15.96	1.71	0.01
86.25	236.27	21.96	25.05	17.96	20.99	14.87	0.96
74.22	224.94	23.88	22.57	10.27	19.30	8.59	0.45
82.19	389.81	26.35	28.22	19.36	25.40	16.58	1.22
198.23	16.52	20.32	12.42	17.50	10.08	0.82	0.03
70.08	234.80	18.10	29.49	14.86	26.84	11.72	0.99
277.85	21.36	25.08	16.56	21.03	12.11	1.08	0.17
110.11	368.04	42.74	38.55	18.00	33.44	14.57	1.47
105.93	347.33	38.61	36.78	20.70	33.86	18.03	1.79
91.49	278.55	22.86	29.91	17.89	25.94	14.93	1.16
180.74	15.91	19.48	12.87	16.94	10.50	0.92	0.03
59.83	214.59	21.47	21.22	10.51	18.76	9.31	0.26
36.56	219.64	12.92	13.20	6.29	11.37	4.53	0.26
65.22	116.46	19.10	23.68	16.89	21.49	14.64	1.23
80.29	231.03	18.49	22.11	15.76	20.16	13.66	1.19
96.70	418.31	42.14	32.09	12.27	27.44	9.69	1.11
289.28	50.28	50.55	29.06	45.89	25.19	2.17	0.00
176.36	14.85	17.28	9.65	14.85	7.62	0.65	0.01
140.54	169.34	40.29	41.83	43.72	39.15	41.96	0.94
52.17	183.02	21.48	17.38	7.88	16.15	6.84	0.72
61.48	265.10	17.67	20.17	12.46	16.02	9.09	0.69
68.56	152.55	21.48	23.58	16.43	21.62	13.98	1.24
66.89	247.96	16.19	19.73	12.14	16.19	9.60	0.62
64.81	299.17	26.89	21.37	9.78	18.50	8.25	0.56
115.43	316.95	41.12	40.65	24.59	37.02	21.44	2.00
65.06	122.70	16.73	16.73	9.29	14.73	7.78	0.45
70.80	142.97	19.50	19.05	9.51	17.31	7.91	0.49
71.36	180.74	15.91	19.48	12.87	16.94	10.50	0.92
293.77	36.76	37.11	21.97	34.81	19.75	1.52	0.00

171.38	31.10	47.31	32.37	42.42	28.18	1.89	0.00
228.90	20.87	26.10	14.31	24.66	13.05	0.65	0.03
76.60	313.94	23.28	22.20	13.25	18.05	10.00	0.92
95.23	249.30	30.28	22.84	13.03	20.18	10.84	0.91
86.91	162.70	21.07	22.23	14.58	20.46	12.67	1.03
184.40	16.07	19.63	12.12	16.14	9.61	0.61	0.00
85.15	90.58	20.06	26.05	28.59	24.35	25.18	2.77
35.97	220.91	13.16	12.11	7.00	9.27	5.05	0.26
51.74	150.81	11.93	18.46	13.49	16.50	11.34	1.08
68.51	104.77	22.92	23.80	15.30	21.91	13.08	1.25
110.55	190.82	24.83	32.10	27.49	28.92	23.95	1.69
131.37	187.72	32.36	51.64	36.25	46.88	31.66	2.34
61.11	174.71	21.41	18.71	14.34	16.97	12.09	1.26
538.73	31.32	46.79	30.41	44.74	27.44	2.04	0.00
49.35	121.10	16.02	17.00	10.78	14.91	9.19	0.62
64.20	256.37	19.57	19.32	11.18	16.60	9.26	0.85
133.72	88.84	29.56	53.71	39.87	51.20	36.41	2.84
54.17	140.23	15.52	19.56	13.78	17.49	11.73	1.08
62.31	123.52	22.98	20.97	11.85	19.03	10.27	0.90
79.17	24.92	44.12	32.19	41.98	29.43	2.16	0.00
75.38	327.72	25.00	24.74	13.53	20.06	9.95	0.94
67.56	189.32	20.31	23.69	16.78	21.09	13.98	1.22
78.27	154.60	24.98	20.93	14.28	18.84	12.43	1.16
98.13	315.44	45.88	31.37	12.00	27.09	9.56	1.24
68.80	144.26	19.36	24.08	18.61	22.23	16.10	1.73
54.59	231.93	18.07	19.01	10.99	16.49	8.74	0.55
71.72	166.43	15.08	20.93	12.36	18.94	10.36	0.90
57.34	162.08	18.92	18.79	13.26	16.40	10.76	1.23
45.72	157.65	14.96	14.69	11.57	12.36	9.61	0.75
147.75	332.79	51.99	49.98	28.65	46.26	25.50	2.23
84.79	193.72	24.09	23.23	17.42	21.30	15.23	1.64
228.90	20.87	26.10	14.31	24.66	13.05	0.65	0.03
66.66	206.03	22.47	24.29	14.29	22.43	12.86	0.77
49.66	238.20	19.61	15.68	6.03	13.76	4.51	0.65
63.93	206.11	22.63	19.25	12.62	16.77	10.20	1.16
126.47	436.77	45.13	41.26	23.52	37.04	19.92	2.09
74.37	294.98	21.53	24.27	15.46	21.04	11.87	1.13
134.72	389.90	49.64	43.02	22.99	38.51	19.42	2.05
60.62	142.50	18.46	19.36	11.67	17.19	9.90	0.60
58.59	175.61	17.65	19.96	14.35	18.12	12.34	1.00
69.76	138.34	18.68	16.07	10.58	14.76	8.54	0.82
100.29	393.27	44.86	33.12	12.65	27.80	9.73	1.15
71.21	190.66	23.15	23.47	14.52	21.22	12.57	0.93
72.10	349.49	24.58	18.97	11.98	15.64	8.59	0.96
44.41	86.37	13.25	16.34	8.98	14.45	7.71	0.27
57.02	73.63	13.78	17.63	9.80	16.20	8.55	0.69
58.76	11.22	14.66	10.67	13.55	9.48	0.63	0.02
124.88	396.17	39.63	39.30	22.13	35.10	18.03	2.01
95.64	236.69	31.08	33.11	11.33	30.47	9.34	0.98
83.40	110.82	12.95	15.53	9.60	13.45	8.13	0.27
78.21	214.52	21.31	21.02	14.95	17.19	11.78	1.01
48.46	137.44	14.36	14.97	9.06	11.41	6.51	0.47
51.48	209.46	18.00	15.61	7.85	13.01	6.05	0.52

47.36	179.55	21.16	14.39	7.20	12.32	5.49	0.79
99.60	213.99	26.75	26.45	26.97	23.53	22.85	2.73
93.97	138.55	27.27	26.20	16.70	23.43	14.43	1.12
198.23	16.52	20.32	12.42	17.50	10.08	0.82	0.03
49.66	238.20	19.61	15.68	6.03	13.76	4.51	0.65
72.02	188.50	18.17	20.21	10.35	17.18	8.03	0.62
64.98	116.38	16.09	18.63	13.67	17.33	12.09	0.84
54.27	249.64	17.35	18.52	11.90	16.03	9.79	0.71
104.50	396.83	48.45	34.00	12.46	28.29	9.17	1.36
86.74	288.84	26.13	26.12	13.59	23.15	10.52	1.03
423.96	41.20	32.80	13.06	28.25	10.32	1.24	0.01
310.53	40.91	48.96	33.73	45.84	30.18	2.54	0.00
187.61	16.62	16.09	7.54	13.64	5.75	0.48	0.03
41.36	163.61	13.90	13.75	7.07	11.93	5.72	0.37
123.38	310.84	37.16	37.67	22.19	35.33	19.96	1.52
96.64	267.52	38.89	35.78	12.04	33.01	10.29	0.83
63.38	186.87	20.59	22.10	12.02	19.30	9.51	0.83
187.84	18.76	21.11	10.72	18.65	8.71	0.77	0.01
84.63	252.08	25.75	26.69	22.48	23.62	19.49	1.55
58.69	193.98	17.15	17.87	16.83	15.73	13.55	1.75
69.80	199.44	26.97	20.69	11.46	17.88	9.46	0.65
195.87	26.87	49.89	33.98	47.16	30.91	2.11	0.00
77.01	195.89	16.20	18.09	16.01	15.12	13.21	1.09
265.10	17.71	20.28	12.39	16.17	9.02	0.69	0.03
89.78	201.25	21.32	36.82	23.59	34.27	21.00	1.51
54.82	124.03	14.13	19.96	11.97	18.01	10.06	0.70
332.58	45.25	48.08	28.61	45.21	25.63	2.24	0.00
82.44	219.62	26.70	28.76	17.92	26.28	15.55	1.17
255.09	17.73	20.36	13.57	16.67	10.70	0.87	0.00
51.00	116.12	16.80	15.93	11.45	13.93	9.51	0.84
43.37	98.65	13.22	13.43	10.46	12.65	9.37	0.76
138.30	332.81	44.24	46.18	26.80	42.96	23.76	2.12
103.04	346.32	46.97	32.93	13.20	27.86	10.11	1.27
296.45	39.47	35.41	18.15	32.45	15.79	1.45	0.00
93.05	365.58	38.94	32.01	12.73	26.88	9.92	1.04
174.44	17.29	20.50	9.75	17.99	7.88	0.61	0.00
70.63	132.99	15.41	19.03	11.95	16.87	9.52	1.10
28.91	105.21	9.44	8.63	7.45	7.00	5.64	0.62
74.74	96.38	17.96	24.42	14.73	22.32	12.66	0.90
74.67	309.63	23.33	29.65	14.30	25.92	10.45	0.93
311.42	46.90	49.41	28.76	44.10	24.56	2.13	0.04
199.67	19.45	18.37	10.12	15.75	7.95	0.77	0.01
59.50	82.77	15.13	21.51	17.57	20.36	15.49	1.56
218.52	39.86	37.57	22.91	34.40	19.51	1.86	0.12
114.48	308.06	46.28	38.51	19.60	34.46	16.52	1.65
64.91	185.36	19.10	16.05	7.48	13.90	6.02	0.55
38.09	102.54	13.37	12.09	6.59	11.23	5.81	0.37
58.59	175.61	17.65	19.96	14.35	18.12	12.34	1.00
152.34	543.26	50.57	50.13	32.00	46.90	28.64	2.70
69.05	110.01	19.98	23.05	17.40	21.63	15.58	1.20
94.97	70.48	19.48	28.05	23.92	23.72	20.44	1.78
65.51	245.96	28.72	19.00	9.85	16.17	7.74	0.90
383.20	40.78	33.49	13.71	29.01	10.99	1.21	0.01

58.08	136.74	14.54	13.85	8.53	12.59	7.30	0.34
74.26	259.61	27.35	24.03	12.73	21.61	10.70	0.78
83.56	225.31	23.03	27.20	20.59	23.55	16.65	1.83
113.47	22.50	37.56	24.93	35.41	22.38	1.50	0.06
50.18	142.21	14.91	19.42	9.84	17.64	8.04	0.59
417.10	49.90	50.14	30.91	46.67	27.73	2.21	0.00
126.47	324.82	48.94	43.70	21.48	38.77	17.60	1.68
81.15	90.13	16.18	24.08	23.62	22.79	21.28	1.70
74.95	157.19	17.92	19.22	14.65	17.25	12.49	1.18
464.57	39.02	33.74	18.23	30.08	15.43	1.60	0.03
379.57	43.27	41.28	22.69	36.54	18.93	2.07	0.00
150.42	310.81	45.08	51.71	32.00	48.28	28.48	2.51
53.41	217.68	18.68	18.71	10.63	16.11	8.61	0.69
66.05	126.58	19.50	21.23	8.68	19.61	7.40	0.64
45.82	97.88	13.35	14.09	8.37	12.05	6.76	0.46
69.13	164.27	20.28	25.71	13.58	23.91	12.13	0.62
118.64	170.45	22.98	46.34	37.16	44.39	33.78	2.10
49.66	238.20	19.61	15.68	6.03	13.76	4.51	0.65
71.21	190.66	23.15	23.47	14.52	21.22	12.57	0.93
97.96	176.78	26.34	35.77	26.04	30.59	21.13	2.21
64.91	185.36	19.10	16.05	7.48	13.90	6.02	0.55
57.18	219.32	18.34	19.45	12.62	17.22	10.57	0.77
50.93	138.18	16.29	16.31	9.15	15.42	8.47	0.34
71.59	218.15	27.01	23.40	14.32	20.26	12.04	0.96
72.02	188.50	18.17	20.21	10.35	17.18	8.03	0.62
123.34	91.14	25.59	50.10	34.98	47.79	32.18	2.01
61.13	201.82	16.68	19.09	9.15	16.91	7.50	0.52
312.63	47.71	45.90	29.01	42.74	25.45	2.50	0.06
107.79	117.48	24.33	42.34	28.59	40.75	26.48	1.53
65.18	167.16	17.96	20.88	14.83	18.96	12.99	0.93
82.20	269.73	23.10	27.66	21.69	23.55	17.25	2.01
63.82	174.44	17.29	20.50	9.75	17.99	7.88	0.61
33.27	107.27	12.56	10.04	5.99	8.62	4.75	0.28
48.15	119.20	15.37	14.89	8.04	12.48	6.32	0.47
53.71	198.12	19.48	19.45	9.37	16.41	7.08	0.78
103.22	339.42	38.02	33.16	19.37	29.99	16.71	1.66
83.90	284.49	25.62	32.43	17.27	29.01	13.11	1.15
56.81	151.28	15.31	17.15	11.47	14.90	9.57	0.64
66.54	197.01	14.51	15.79	12.73	12.83	10.18	0.77
53.29	105.97	15.73	17.28	12.52	16.06	10.90	0.88
91.14	206.75	22.63	26.79	16.42	23.76	13.96	1.02
69.91	155.37	18.06	22.81	22.33	18.70	18.23	1.70
71.74	249.89	18.79	30.08	15.16	26.86	11.82	0.93
49.70	138.60	20.78	16.48	8.43	14.08	6.98	0.39
90.18	286.72	38.58	28.99	13.17	24.91	10.61	1.18
59.64	142.90	17.12	18.50	13.34	16.96	11.64	0.99
59.52	222.33	22.10	20.10	9.48	18.20	7.83	0.73
178.57	15.71	15.95	8.26	13.51	6.51	0.47	0.01
74.75	177.32	34.29	21.54	13.29	19.23	10.88	1.53
73.68	109.17	20.44	25.17	20.37	23.23	17.79	2.00
71.15	179.25	18.82	23.30	11.74	20.78	9.77	0.68
105.70	138.67	24.54	40.86	27.97	38.16	24.60	1.69
72.93	116.24	20.64	22.06	23.16	19.69	19.56	2.18

42.42	201.80	14.54	14.90	9.01	12.60	7.38	0.43
69.39	148.36	22.20	25.02	16.42	23.30	13.28	1.86
117.10	15.69	15.19	8.64	12.83	6.87	0.53	0.01
109.74	277.30	38.01	36.35	21.43	33.22	19.04	1.48
105.85	143.81	30.65	40.31	24.09	37.30	22.01	1.51
100.41	111.58	23.05	39.91	26.26	37.80	23.77	1.46
95.64	236.69	31.08	33.11	11.33	30.47	9.34	0.98
60.19	149.37	24.71	18.74	10.89	17.05	9.64	0.75
54.45	132.37	16.32	18.51	14.98	17.27	13.17	1.38
83.12	175.96	18.41	20.57	18.49	17.58	15.51	1.36
86.94	275.91	35.63	28.72	13.76	24.54	10.84	1.28
136.99	293.03	39.19	46.35	30.34	43.83	27.40	2.08
51.77	202.76	19.18	17.29	7.81	15.29	6.43	0.52
71.68	208.25	19.09	19.14	9.84	16.81	7.98	0.59
108.64	250.88	29.65	29.64	20.64	26.58	17.56	1.73
59.55	115.13	18.79	18.05	12.12	15.35	10.15	0.73
115.12	157.32	32.72	43.01	26.95	39.59	23.91	1.83
49.66	238.20	19.61	15.68	6.03	13.76	4.51	0.65
66.14	192.95	18.76	17.09	8.13	14.37	6.31	0.57
57.49	139.55	16.29	19.21	12.32	17.19	10.51	1.15
196.74	19.35	18.51	9.54	15.89	7.63	0.62	0.01
102.72	408.49	41.70	33.88	14.63	30.49	12.38	1.00
53.53	175.95	16.03	17.50	10.90	15.27	9.18	0.77
59.47	152.38	16.87	20.52	14.01	18.16	12.05	0.45
66.54	197.01	14.51	15.79	12.73	12.83	10.18	0.77
63.81	160.16	21.50	20.87	14.07	18.61	11.82	1.08
125.57	305.27	37.67	38.21	22.97	35.47	20.40	1.57
71.83	189.56	30.41	22.36	11.64	19.31	9.43	0.94
67.63	208.96	26.13	23.18	11.66	18.94	9.66	0.49
64.49	169.48	26.94	23.43	7.71	21.05	6.13	0.72
81.37	234.97	20.12	27.72	20.70	23.64	16.34	1.51

epa	dha	trans.fa	sodium	potasium	vt.a.rae	b.carote	a.carote
0.01	0.03	0.00	2284.43	3673.31	904.39	7440.18	2268.60
0.03	0.08	0.00	3447.37	7306.53	1459.44	14295.20	3844.19
0.00	0.00	0.00	5182.06	2970.55	360.78	2483.17	963.37
0.01	0.02	0.00	3342.96	3740.71	898.30	7927.75	2502.54
0.01	0.05	0.00	3114.27	2383.14	243.81	1963.61	199.41
0.01	0.02	0.00	3929.98	5382.88	1320.75	11465.86	3669.72
0.03	0.08	0.00	3311.66	4903.70	722.29	6003.62	1207.52
0.12	0.35	0.00	2226.37	3635.80	314.73	1273.68	110.88
0.02	0.00	3505.00	4018.58	958.48	8220.09	2506.37	2658.79
0.00	0.00	0.00	5752.54	2973.72	572.08	4103.71	1453.46
0.11	0.00	3474.02	3425.13	719.19	2371.32	486.82	859.52
0.06	0.18	0.00	4184.37	4961.01	1134.72	7606.77	2176.42
0.17	0.00	4275.95	3811.15	599.12	3948.46	944.30	3346.42
0.01	0.05	0.00	3428.97	2284.14	286.58	2401.48	450.34
0.00	0.00	0.00	2676.38	3640.00	419.21	3822.76	1038.92
0.03	0.08	0.00	3442.38	4714.69	718.95	5804.11	1203.68
0.00	0.03	0.00	5191.86	3324.01	704.61	3158.65	973.41
0.06	0.18	0.00	1556.87	2810.65	380.80	1966.14	417.36
0.01	0.04	0.00	2425.57	2413.87	355.70	2520.45	233.64
0.00	0.03	0.00	5270.97	2796.94	547.60	1388.20	322.89
0.00	0.00	0.00	3850.13	2770.11	462.66	2515.11	936.58
0.02	0.09	0.00	3263.20	3702.01	498.85	1625.40	288.95
0.01	0.04	0.00	4387.45	3102.89	1173.11	12350.01	1116.49
0.01	0.02	0.00	3498.32	3850.49	528.69	5338.43	405.91
0.03	0.08	0.00	3447.37	7306.53	1459.44	14295.20	3844.19
0.01	0.06	0.00	4050.37	2918.18	609.68	2721.01	328.62
0.06	0.17	0.00	3537.28	4353.94	1343.63	11683.95	1486.10
0.01	0.02	0.00	6616.13	4964.45	1723.66	16794.64	3954.96
0.00	0.09	0.00	4800.41	2877.84	586.14	1373.46	322.26
0.00	0.04	0.00	2737.71	2585.10	676.73	2723.74	342.56
0.01	0.04	0.00	3009.42	2770.36	688.43	2559.42	577.13
0.01	0.09	0.00	3405.81	3533.01	586.43	1652.24	341.28
0.03	0.08	0.00	3376.08	5165.25	703.62	6009.92	1207.52
0.00	0.02	0.00	4267.45	4271.05	741.16	6815.55	2194.36
0.18	0.00	4051.21	3054.42	252.72	1376.88	216.77	2144.83
0.04	0.00	2961.88	4306.96	746.06	6843.44	1591.36	4311.53
0.00	0.01	0.00	3349.23	2751.51	399.01	2578.43	729.42
0.01	0.03	0.00	3365.81	3705.22	475.87	3205.01	290.48
0.00	0.00	0.00	3218.42	2617.97	345.51	2458.44	959.65
0.12	0.36	0.01	2865.48	3623.58	412.97	2755.75	693.54
0.00	0.00	0.00	2995.21	3203.83	589.17	4350.86	1244.42
0.03	0.09	0.00	3203.37	3968.98	741.92	3462.52	598.19
0.00	0.03	0.00	3982.32	3284.01	561.06	1260.37	354.90
0.00	0.01	0.00	1981.56	2883.75	381.08	3047.27	290.71
0.06	0.17	0.00	3421.46	4535.13	777.33	6707.89	1541.85
0.00	0.01	0.00	2417.32	2015.53	179.87	1224.60	180.75
0.01	0.04	0.00	3527.99	3162.86	464.77	3975.78	790.26
0.00	0.00	0.00	0.00	0.00	0.00	0.00	0.00
0.00	0.01	0.00	2458.62	3129.99	867.08	8165.01	3052.06
0.28	0.00	3941.05	5690.26	1184.91	5004.77	613.20	1497.34
0.01	0.03	0.00	4378.47	5329.53	609.59	2779.92	557.99
0.01	0.02	0.00	3205.91	3761.89	669.49	5655.66	1445.06

0.03	0.08	0.00	4957.26	2625.86	349.18	2011.39	189.75
0.00	0.00	0.00	4619.46	2270.81	322.51	2539.71	1035.18
0.00	0.00	0.00	3307.21	2458.44	229.42	2008.61	296.39
0.04	0.00	2516.50	3364.37	766.53	6974.67	1852.23	2468.68
0.03	0.00	3094.46	4322.73	683.60	5794.12	1817.56	3435.54
0.12	0.35	0.00	4238.30	5989.40	1976.76	17462.79	3098.24
0.02	0.00	3342.96	3740.71	898.30	7927.75	2502.54	2616.26
0.06	0.17	0.00	4462.94	3714.29	536.85	3620.97	1189.21
0.03	0.08	0.00	3063.57	2312.54	303.91	1120.19	244.51
0.13	0.00	3886.37	3444.60	698.60	2338.94	379.36	849.65
0.04	0.00	2812.16	4981.74	890.11	7829.66	1924.52	5055.05
0.01	0.04	0.00	2516.50	3364.37	766.53	6974.67	1852.23
0.00	0.00	0.00	4435.60	4594.18	304.79	2074.91	175.76
0.17	0.53	0.00	4234.57	9491.30	1958.22	18687.37	3256.77
0.00	0.00	0.00	4005.29	2289.66	356.45	2548.60	691.37
0.17	0.00	4275.95	3811.15	599.12	3948.46	944.30	3346.42
0.02	0.00	3086.17	6768.17	1322.99	11061.54	3247.27	7012.02
0.01	0.03	0.00	2722.29	2578.10	554.03	5164.55	318.75
0.12	0.36	0.01	2865.48	3623.58	412.97	2755.75	693.54
0.00	0.02	0.00	3837.98	4367.42	624.87	3178.14	498.22
0.03	0.00	5937.47	7050.83	1964.52	18142.73	1016.44	2713.25
0.04	0.00	3242.14	3569.58	641.61	2910.39	827.76	2355.15
0.00	0.00	0.00	2620.85	1821.49	248.58	2003.92	148.98
0.01	0.04	0.00	4014.62	3106.90	436.01	3197.68	599.62
0.08	0.00	6245.27	3864.83	659.61	2652.49	442.13	728.68
0.00	0.00	0.00	5179.97	3621.11	458.44	3573.66	1357.38
0.03	0.09	0.00	4286.49	3026.11	543.11	3786.57	863.44
0.03	0.13	0.00	4215.72	3140.12	460.04	3083.67	342.60
0.08	0.00	4028.22	4570.49	669.11	5553.04	1484.60	2874.12
0.12	0.35	0.00	4133.72	8880.06	1525.47	14278.42	2814.80
0.53	0.00	4489.35	6432.94	1320.50	11735.71	2047.30	4527.55
0.01	0.08	0.00	3415.49	2798.40	503.16	1297.46	268.55
0.02	0.09	0.00	3362.93	3510.05	507.97	1732.38	232.45
0.06	0.23	0.00	6016.93	4855.80	1290.66	10861.44	3914.29
0.08	0.00	3563.29	4814.27	1071.07	9988.78	2597.31	2842.31
0.01	0.02	0.00	4608.29	3417.13	309.99	1239.80	216.22
0.17	0.52	0.00	2541.71	3034.80	398.70	1830.09	220.65
0.00	0.00	0.01	3894.70	4120.04	498.69	4723.07	453.03
0.00	0.02	0.00	3226.76	3030.30	617.63	4483.85	1022.96
0.01	0.09	0.00	2900.12	3091.12	609.30	2164.24	332.17
0.04	0.00	3601.99	3068.06	565.98	1697.24	202.82	676.42
0.04	0.00	3078.73	4172.94	1037.00	9631.96	2519.34	2688.71
0.00	0.02	0.00	5755.94	5957.92	825.50	5570.21	239.24
0.01	0.04	0.00	2114.35	3148.92	435.41	2843.43	743.33
0.03	0.09	0.00	2941.84	3488.91	737.49	4407.03	1306.03
0.01	0.02	0.00	3678.44	3773.51	891.46	7675.24	1670.18
0.00	0.00	0.00	3522.29	1858.21	352.70	2549.37	1041.04
0.01	0.05	0.00	5272.26	3308.18	492.33	2728.14	267.31
0.02	0.09	0.00	3478.40	3250.88	490.94	1639.81	271.03
0.06	0.17	0.00	3968.28	5007.43	626.50	4439.50	1508.60
0.06	0.19	0.00	3829.47	2859.50	397.55	3278.94	112.11
0.03	0.08	0.00	3563.29	4814.27	1071.07	9988.78	2597.31
0.03	0.00	5035.92	3317.83	634.28	2520.70	684.82	1928.94

0.03	0.00	4447.77	4654.85	602.14	5320.83	1648.28	3385.50
0.09	0.00	3565.41	4655.66	936.34	7449.46	2365.14	5605.56
0.01	0.04	0.00	3526.26	3451.15	575.79	2916.05	830.04
0.06	0.18	0.00	4592.96	4592.05	914.52	5460.77	1245.24
0.03	0.09	0.00	4917.41	4239.26	629.89	5508.26	714.46
0.00	0.00	3765.75	1895.86	335.21	2547.05	1040.08	1061.30
0.00	0.00	0.00	2847.64	3178.95	334.58	2404.25	360.23
0.00	0.00	0.00	1564.46	3341.45	736.54	6653.30	1745.41
0.01	0.02	0.00	2609.10	2228.15	278.08	2016.13	331.90
0.03	0.11	0.00	2474.89	5043.34	1450.93	15085.36	2238.55
0.00	0.01	0.00	4944.79	3804.54	331.93	2127.51	286.20
0.00	0.00	0.00	3895.01	4035.66	519.36	4037.85	1154.65
0.00	0.01	0.00	2895.74	5757.71	1814.62	18157.10	1330.11
0.00	0.00	3861.14	4484.36	857.09	4728.41	1318.97	3769.66
0.01	0.02	0.00	4897.71	2724.90	250.81	2119.06	408.69
0.03	0.10	0.00	7098.54	4991.58	1598.00	8318.40	1605.83
0.00	0.00	0.00	2492.97	3084.10	472.71	3456.44	995.99
0.00	0.02	0.00	2793.90	2998.28	1170.73	11431.65	2793.44
0.00	0.02	0.00	3220.53	3544.45	521.20	3242.89	1043.12
0.00	0.00	3336.48	3386.32	399.31	3432.86	995.99	2104.49
0.03	0.09	0.00	3223.75	3635.36	767.56	4431.91	1308.33
0.06	0.19	0.00	3580.70	5581.22	780.22	6148.93	984.04
0.01	0.02	0.00	4297.44	4738.44	486.55	3399.55	604.26
0.01	0.04	0.00	2865.55	2699.03	649.24	1931.40	325.41
0.00	0.01	0.00	3329.50	2507.44	335.53	2717.82	646.34
0.12	0.40	0.00	3874.65	4520.44	983.35	8188.19	1395.93
0.03	0.08	0.00	3531.50	7965.71	1498.12	14700.88	3849.96
0.03	0.08	0.00	3180.30	3330.42	783.32	6831.63	775.47
0.03	0.08	0.00	2740.58	3775.44	910.04	7904.27	2046.15
0.01	0.04	0.00	4607.19	3227.51	704.22	1819.69	619.88
0.01	0.05	0.00	3836.52	4825.55	616.47	4625.23	444.56
0.09	0.00	3565.41	4655.66	936.34	7449.46	2365.14	5605.56
0.03	0.08	0.00	2267.47	3166.64	448.90	2193.09	504.09
0.05	0.17	0.00	4275.95	3811.15	599.12	3948.46	944.30
0.06	0.18	0.00	2697.56	4618.99	988.23	7920.93	2205.69
0.01	0.11	0.00	3711.38	3404.74	682.13	2676.89	431.93
0.14	0.44	0.00	3844.91	5795.90	1200.11	9598.07	2026.93
0.03	0.11	0.00	3813.21	4318.73	801.95	3435.58	600.41
0.06	0.18	0.00	3231.54	3820.45	480.10	3881.71	363.12
0.06	0.18	0.00	2890.90	4189.09	546.34	3750.05	1247.74
0.06	0.18	0.00	4217.11	5282.64	981.05	7607.38	2214.95
0.01	0.08	0.00	3153.41	2904.74	712.35	3010.45	800.70
0.01	0.04	0.00	3641.27	4561.36	1456.15	11749.60	4155.55
0.17	0.59	0.00	3418.58	5350.65	618.60	4446.97	1066.51
0.01	0.02	0.00	2745.95	2003.72	182.97	1507.19	176.75
0.01	0.04	0.00	3578.53	2854.11	454.47	4375.68	378.74
0.06	0.00	3086.76	2504.43	368.29	3073.05	1110.64	1078.61
0.12	0.37	0.00	4385.32	5178.74	1243.71	8691.89	1241.30
0.08	0.25	0.00	4500.76	4003.39	332.20	1967.61	369.74
0.06	0.18	0.00	5381.52	3763.44	310.67	2591.56	616.00
0.00	0.00	0.00	4788.17	2648.64	433.32	2326.84	932.15
0.01	0.04	0.00	2933.10	3623.85	637.25	5720.43	1275.58
0.01	0.04	0.00	2889.82	4228.24	817.86	6854.54	1605.94

0.06	0.18	0.00	6018.81	2527.44	577.75	4553.51	404.72
0.01	0.02	0.00	4018.61	5681.87	482.95	3993.76	528.31
0.01	0.02	0.00	4413.46	5122.41	658.90	5808.26	1034.43
0.08	0.00	4028.22	4570.49	669.11	5553.04	1484.60	2874.12
0.05	0.17	0.00	4275.95	3811.15	599.12	3948.46	944.30
0.03	0.08	0.00	3642.08	4934.02	679.23	5363.97	1205.07
0.01	0.05	0.00	6770.69	2434.21	302.98	2300.88	293.51
0.01	0.05	0.00	3352.65	3168.39	397.52	2656.19	455.26
0.01	0.07	0.00	3889.10	2901.07	652.56	1985.46	321.55
0.12	0.35	0.00	4069.60	4963.55	1078.27	8686.26	1202.01
0.09	0.00	4707.17	2860.50	545.83	1409.79	75.76	694.97
0.04	0.00	4781.75	3179.37	507.53	914.33	112.65	1128.07
0.08	0.00	2673.69	4750.44	857.91	7256.58	1810.43	4964.12
0.03	0.08	0.00	2731.73	4914.28	861.44	7531.67	1873.08
0.00	0.03	0.00	5131.17	3370.51	706.45	3158.14	973.41
0.01	0.02	0.00	4849.51	7477.77	1371.86	9443.08	2076.26
0.06	0.17	0.00	2385.27	4902.24	1135.11	10433.52	630.69
0.07	0.00	3561.04	6885.89	1241.17	10878.31	3095.25	7971.04
0.06	0.19	0.00	3956.42	4356.39	725.87	4938.41	1759.31
0.06	0.18	0.00	3619.08	3351.88	661.29	3880.24	834.02
0.03	0.08	0.00	2777.95	4703.98	1341.19	9969.79	2747.30
0.03	0.00	3750.51	4380.65	635.48	5459.98	1673.46	3492.98
0.00	0.00	0.00	4463.45	3577.39	346.08	2470.75	959.65
0.09	0.00	2982.33	3434.61	740.52	4437.23	1306.44	2486.03
0.00	0.03	0.00	2759.79	3836.36	523.00	4345.56	1277.74
0.06	0.17	0.00	2592.90	3510.59	789.43	7533.78	1574.62
0.10	0.00	4881.22	2949.17	599.89	1367.38	321.07	1078.14
0.03	0.09	0.00	4617.13	4154.82	710.41	5254.45	733.14
0.00	0.00	3879.00	2013.15	352.20	2547.12	1040.08	1092.89
0.03	0.09	0.00	2474.64	2792.83	325.86	1860.31	344.78
0.01	0.04	0.00	2765.50	2514.12	452.19	1529.26	198.09
0.00	0.06	0.00	4362.85	2591.47	594.96	1356.25	321.06
0.01	0.06	0.00	3931.09	3146.84	784.20	3547.27	344.70
0.06	0.00	4438.38	2645.10	582.12	1355.89	321.06	1001.73
0.01	0.08	0.00	3344.26	3033.64	633.25	3135.78	801.10
0.06	0.00	3650.98	6526.74	1206.27	11077.72	3090.62	8370.45
0.06	0.18	0.00	3694.17	3150.64	293.45	1849.44	166.05
0.00	0.00	0.00	1700.53	2683.81	357.30	3262.25	357.63
0.03	0.09	0.00	4010.81	3980.63	692.61	6714.79	974.15
0.14	0.44	0.00	4180.65	8131.54	1693.87	15784.81	2257.66
0.15	0.00	3656.23	3591.00	392.65	1682.90	309.04	1111.66
0.02	0.00	3409.23	4208.26	1110.77	9849.33	2519.64	2794.48
0.00	0.02	0.00	2067.56	3340.67	464.43	4531.82	535.35
0.37	0.00	3767.07	3230.28	522.93	2039.01	445.94	1870.43
0.00	0.04	0.00	3559.21	3284.26	704.77	2646.50	228.01
0.01	0.02	0.00	3223.63	3912.61	599.69	4624.01	918.88
0.00	0.02	0.00	3139.68	2337.83	607.24	4644.11	1113.19
0.06	0.18	0.00	2890.90	4189.09	546.34	3750.05	1247.74
0.00	0.07	0.00	3698.65	3395.71	717.05	2329.94	427.76
0.01	0.04	0.00	3071.52	3725.86	508.41	4050.68	1393.36
0.02	0.05	0.00	3766.13	3975.02	840.29	7994.83	1903.43
0.03	0.08	0.00	4136.95	5635.46	715.96	6197.53	804.80
0.08	0.00	5617.04	3079.69	613.05	2140.37	435.08	649.85

0.06	0.20	0.00	4001.26	2399.34	324.03	1784.19	283.71
0.02	0.06	0.00	3802.04	2856.49	501.23	2881.76	508.06
0.12	0.37	0.00	3589.04	5336.34	1055.53	9798.22	1282.41
0.17	0.00	3638.72	3455.81	536.26	3488.77	1007.07	1891.80
0.06	0.18	0.00	3106.21	3133.72	430.56	4103.98	968.79
0.05	0.00	3413.88	3075.47	699.11	2138.30	493.24	1288.32
0.03	0.11	0.00	4004.04	3996.35	715.26	2376.47	359.67
0.00	0.01	0.00	3368.28	3135.25	590.74	5471.19	1422.87
0.03	0.09	0.00	3168.04	2995.44	343.69	2248.55	276.62
0.14	0.00	3720.09	3793.57	698.70	2455.25	480.86	961.04
0.04	0.00	3971.95	4375.97	680.24	3177.30	498.23	1621.29
0.00	0.05	0.00	4451.09	2660.89	573.28	1381.58	322.95
0.03	0.08	0.00	3577.60	3259.81	398.39	2401.66	576.61
0.03	0.08	0.00	4337.78	3524.90	382.99	2770.73	323.81
0.01	0.04	0.00	2900.93	4478.08	954.55	9184.25	2173.84
0.00	0.00	0.00	3689.13	3341.06	729.65	6017.75	693.40
0.06	0.17	0.00	4441.13	3616.09	511.86	4217.67	1346.19
0.05	0.17	0.00	4275.95	3811.15	599.12	3948.46	944.30
0.01	0.04	0.00	3641.27	4561.36	1456.15	11749.60	4155.55
0.06	0.18	0.00	3250.39	3349.50	374.38	3050.43	782.64
0.01	0.02	0.00	3223.63	3912.61	599.69	4624.01	918.88
0.03	0.10	0.00	3792.47	3757.03	607.39	5041.95	458.10
0.01	0.02	0.00	2274.84	2697.82	457.33	2379.20	728.08
0.00	0.00	0.00	3762.62	3928.51	908.84	8171.39	2154.38
0.03	0.08	0.00	3642.08	4934.02	679.23	5363.97	1205.07
0.00	0.00	0.00	4500.64	3834.57	479.95	4330.23	1245.38
0.01	0.02	0.00	3609.10	6601.41	1174.82	10384.94	3096.82
0.20	0.00	5040.09	3160.49	528.69	1893.17	357.61	1649.75
0.00	0.00	0.00	5525.20	4375.89	604.71	4089.81	1247.25
0.00	0.02	0.00	3055.23	3056.66	551.39	4307.54	955.50
0.06	0.19	0.00	4471.96	4726.02	942.15	7494.40	1531.30
0.00	0.06	0.00	3650.98	6526.74	1206.27	11077.72	3090.62
0.06	0.17	0.00	2131.87	2809.46	438.30	3931.13	1219.12
0.01	0.04	0.00	2961.88	4306.96	746.06	6843.44	1591.36
0.06	0.18	0.00	3434.33	4173.15	935.86	9042.77	1640.15
0.04	0.18	0.00	3884.40	3995.03	725.63	2718.10	451.64
0.20	0.61	0.00	4552.59	8324.45	1723.12	15212.17	2676.80
0.03	0.09	0.00	4073.74	3920.54	591.32	5067.18	1268.73
0.03	0.08	0.00	3958.50	3088.07	415.07	3276.71	969.35
0.00	0.01	0.00	5842.49	3822.34	849.44	7734.18	672.20
0.01	0.02	0.00	5534.53	5749.22	979.22	7635.61	1992.30
0.01	0.04	0.00	3371.20	4691.64	298.59	1943.20	308.47
0.17	0.53	0.00	4118.67	9048.16	1552.46	12757.23	3526.96
0.00	0.01	0.00	3186.63	4055.76	665.75	5068.05	523.11
0.03	0.10	0.00	2775.90	3017.49	591.30	2780.68	335.16
0.00	0.01	0.00	2974.51	3424.85	601.86	5854.94	831.49
0.01	0.02	0.00	3086.17	6768.17	1322.99	11061.54	3247.27
0.04	0.00	2812.16	4981.74	890.11	7829.66	1924.52	5055.05
0.03	0.08	0.00	3669.58	2828.33	884.66	5328.51	1301.63
0.00	0.01	0.00	3206.33	3953.94	328.20	1130.08	231.22
0.00	0.06	0.00	4395.37	6977.29	1192.24	10904.14	3091.44
0.12	0.35	0.00	3840.53	4123.31	554.14	3589.92	1008.11
0.00	0.00	0.00	1508.38	4056.56	500.22	2780.18	120.37

0.00	0.01	0.00	3173.48	2661.11	550.51	4982.57	994.68
0.12	0.35	0.00	2497.63	4526.53	1273.34	6662.30	2604.48
0.04	0.00	2968.74	4398.70	769.75	7145.61	1593.40	4003.40
0.00	0.05	0.01	3312.48	2922.23	437.44	2109.09	491.28
0.00	0.03	0.00	3667.87	5029.01	480.61	4317.36	1275.04
0.06	0.17	0.00	4015.65	4034.99	588.24	3992.61	1246.52
0.08	0.25	0.00	4500.76	4003.39	332.20	1967.61	369.74
0.01	0.02	0.00	3688.09	3797.32	792.69	6429.72	1983.09
0.00	0.01	0.00	3196.25	3191.43	473.55	3446.39	978.68
0.00	0.00	0.00	5182.06	2970.55	360.78	2483.17	963.37
0.01	0.08	0.00	2846.83	3166.96	603.26	3130.48	801.10
0.00	0.03	0.00	5020.03	3300.10	700.94	3164.21	973.55
0.01	0.02	0.00	2932.96	5047.18	1106.30	9172.65	2667.87
0.03	0.08	0.00	3416.96	5204.35	750.59	5723.04	1203.70
0.01	0.04	0.00	4805.98	5707.75	1351.08	10743.83	3058.09
0.00	0.00	0.00	3590.90	2712.89	322.03	2465.72	366.52
0.00	0.03	0.00	4935.74	4531.24	495.84	4084.71	1315.21
0.05	0.17	0.00	4275.95	3811.15	599.12	3948.46	944.30
0.01	0.02	0.00	3205.91	3761.89	669.49	5655.66	1445.06
0.00	0.00	0.00	3002.78	2972.94	796.13	7086.49	1742.77
0.02	0.00	3505.00	4018.58	958.48	8220.09	2506.37	2658.79
0.03	0.11	0.00	4209.17	3666.41	732.84	2386.64	487.94
0.04	0.12	0.00	3270.76	3892.60	963.06	7640.81	2140.75
0.03	0.09	0.00	5564.13	3294.27	877.64	7833.14	1751.25
0.03	0.08	0.00	3958.50	3088.07	415.07	3276.71	969.35
0.06	0.18	0.00	3916.63	4082.59	667.11	4998.56	739.32
0.00	0.03	0.00	5577.45	3415.65	633.63	2510.25	681.97
0.03	0.09	0.00	3928.36	3361.83	599.17	5143.54	324.95
0.01	0.02	0.00	3331.51	3010.17	689.21	4752.87	683.29
0.01	0.05	0.00	3977.25	2885.70	302.59	2149.44	393.07
0.12	0.35	0.00	4295.79	5740.27	984.03	9159.21	3623.63

lutein	b.crypto	lycopene	calcium	iron	vit.d.ug	vit.e.mg	a.tocoph
1843.70	335.09	4687.10	893.59	15.67	1.46	9.53	5.92
7610.48	224.21	4101.63	2277.83	118.33	0.95	8.58	11.72
559.90	77.16	3530.33	1116.49	19.50	0.69	8.84	4.85
2616.26	253.45	3809.08	1141.56	31.04	1.07	7.51	5.81
2304.40	45.68	4450.92	713.20	14.35	0.43	9.66	5.28
7160.09	205.31	4023.64	1374.93	21.39	2.42	12.75	8.56
3751.49	303.39	5865.07	1477.67	53.99	1.71	9.04	7.69
367.40	126.01	2548.96	1497.69	13.80	2.32	5.65	2.54
292.61	4844.96	160.07	34.48	1.29	9.56	7.12	1.57
935.45	169.90	2565.40	1203.83	16.11	3.31	5.86	3.08
137.89	2476.04	80.43	20.62	3.63	14.25	8.52	1.63
2725.44	359.59	5005.00	1886.11	18.98	5.06	15.83	10.40
87.79	3186.35	105.72	54.31	1.68	3.94	4.96	1.40
1368.44	260.37	4075.85	762.25	16.27	0.30	8.92	5.74
2774.89	132.11	634.64	1230.77	40.78	0.10	14.57	9.72
3733.78	264.37	5703.83	1626.55	52.31	1.68	7.60	6.81
1995.06	66.23	502.07	1170.33	38.64	2.70	17.46	11.72
1198.78	128.60	3759.33	895.83	11.86	2.35	8.55	5.24
1757.74	119.66	2218.80	763.25	13.95	0.71	6.37	3.92
1179.23	43.38	441.33	927.17	25.41	2.23	12.76	8.40
1275.43	127.50	353.36	1264.41	13.95	3.31	9.07	4.75
1263.27	230.84	2169.46	1009.28	15.78	1.88	14.25	9.51
4479.44	300.86	1013.69	882.97	17.43	0.14	12.50	9.01
1784.83	398.97	6739.89	909.32	18.45	0.08	14.83	8.73
7610.48	224.21	4101.63	2277.83	118.33	0.95	8.58	11.72
665.88	111.75	1253.44	817.00	17.92	3.21	13.85	8.92
4963.13	285.91	6786.43	1440.99	18.58	2.78	13.81	10.52
5668.55	465.62	3788.29	1387.46	22.47	0.30	12.71	9.47
1050.71	42.14	502.58	904.55	22.20	4.67	16.24	9.12
1285.34	209.09	2407.65	698.24	13.51	2.19	9.34	6.36
599.70	126.43	1368.12	766.64	14.02	2.43	8.87	6.30
1059.29	345.20	2883.14	963.28	22.05	2.88	14.04	8.09
3751.49	303.39	5865.07	1563.89	54.16	1.02	9.14	7.86
2010.48	362.23	8224.39	806.01	21.79	0.24	13.28	7.98
330.09	1482.68	102.19	21.26	0.67	8.37	5.33	1.63
165.89	4315.80	190.02	52.35	0.62	7.88	7.65	1.27
891.58	84.21	2856.21	831.39	14.86	1.46	8.30	4.86
3312.76	311.34	3619.23	1107.21	15.35	1.70	10.31	6.60
451.78	56.39	3530.33	628.71	10.42	0.63	6.46	3.87
2770.12	48.26	1294.15	1201.68	54.26	0.84	9.72	8.66
2168.14	138.77	4584.10	1048.37	35.84	0.19	20.63	12.38
2183.02	300.54	6379.37	1047.23	37.88	2.96	13.08	8.81
889.50	125.70	1846.68	1193.45	19.56	2.63	10.44	5.76
2817.89	101.16	4668.19	798.56	16.00	0.78	10.49	6.47
3256.23	425.03	5366.96	1004.88	16.51	0.20	12.24	8.24
1044.17	97.45	762.80	531.65	15.22	0.05	8.13	4.30
2332.95	338.77	3772.31	951.93	16.97	0.10	8.65	6.12
0.00	0.00	0.00	0.00	0.00	0.00	0.00	0.00
1748.02	410.11	3968.74	1089.26	14.91	0.02	9.23	6.42
432.56	12196.96	329.21	24.24	3.06	20.00	13.62	1.78
1329.80	199.09	4658.92	1900.31	21.21	5.71	11.64	6.46
2328.99	209.70	3808.74	1213.60	31.47	1.12	7.36	5.50

1192.22	87.67	3975.96	929.76	16.16	1.45	6.84	3.83
1113.66	156.26	353.53	730.05	18.26	0.34	8.93	4.59
1523.85	59.39	3589.79	655.64	16.71	0.07	9.59	5.26
156.55	3201.59	156.46	29.81	0.92	6.85	5.66	1.11
185.38	1907.45	119.75	54.79	3.51	21.66	14.19	1.37
10259.53	446.80	6741.35	2198.35	25.27	4.95	16.81	12.04
253.45	3809.08	155.25	31.04	1.07	7.51	5.81	1.12
1880.72	123.69	1841.66	939.88	43.83	0.55	19.56	11.31
883.36	41.86	2781.76	571.65	16.77	0.78	19.05	12.07
158.19	2647.22	80.40	20.36	3.37	10.83	5.69	1.82
201.73	4100.26	217.07	72.55	1.22	8.28	9.03	1.15
2468.68	156.55	3201.59	942.06	29.81	0.92	6.85	5.66
1763.97	26.97	3530.16	1540.87	23.03	0.76	9.18	5.25
9495.27	624.54	5153.96	2731.29	125.27	2.47	12.56	14.28
967.05	97.14	3976.19	730.61	14.97	0.38	7.60	4.00
87.79	3186.35	105.72	54.31	1.68	3.94	4.96	1.40
212.26	2028.48	238.92	116.17	1.78	8.51	12.00	1.25
2980.99	346.79	5198.09	613.45	13.79	0.51	13.31	7.86
2770.12	48.26	1294.15	1201.68	54.26	0.84	9.72	8.66
1609.10	385.81	6330.12	1098.54	24.66	2.06	12.33	6.94
825.02	4957.63	737.96	29.89	1.41	39.35	26.20	1.67
259.59	2045.32	102.14	23.98	3.05	9.54	6.24	1.48
883.18	35.17	3672.21	428.12	11.76	0.15	7.32	4.57
1995.25	216.65	3761.98	931.82	15.57	1.21	8.77	5.29
199.26	3741.20	82.01	23.36	3.10	11.42	6.47	2.19
1328.39	200.03	3530.50	1284.15	26.99	0.63	11.31	5.98
1608.62	116.48	3756.12	811.42	15.37	0.78	10.02	6.23
4603.76	166.01	947.77	1002.98	21.26	1.13	15.50	9.38
127.44	678.02	140.01	55.39	1.18	7.79	7.31	1.56
9590.01	612.33	5405.60	2473.69	128.44	1.60	14.06	15.29
694.77	3629.93	377.60	46.85	2.33	13.08	9.15	2.57
847.63	323.56	1775.99	851.37	14.94	3.03	10.41	5.70
1571.48	232.98	2169.46	908.19	17.79	1.88	13.56	7.71
3491.84	423.88	5883.31	1170.91	25.10	1.72	11.61	7.29
277.62	6862.20	203.43	36.58	1.27	10.43	7.79	1.64
676.14	46.45	5881.85	1124.66	20.07	1.32	9.90	5.54
1950.94	75.51	623.56	831.88	11.72	1.28	5.34	3.18
1026.30	383.54	6654.71	976.03	19.39	0.01	10.50	5.24
1078.44	177.25	5676.49	865.24	19.04	2.09	9.93	6.25
999.57	170.61	2399.18	839.36	14.25	3.71	9.08	6.53
115.94	1291.16	74.29	20.71	2.20	17.73	9.84	1.75
280.49	4844.96	188.17	32.71	1.29	9.08	7.14	1.24
3155.59	326.56	3874.35	718.49	19.14	1.08	26.75	34.17
3222.79	101.73	545.39	1382.12	35.73	2.67	7.31	6.22
2326.60	289.38	2045.79	873.96	22.43	2.46	11.28	7.48
5373.55	343.05	1279.81	1072.58	19.94	1.47	11.24	6.17
1110.88	161.96	471.20	524.69	15.84	0.44	8.03	4.30
1224.97	442.08	5656.47	811.35	15.51	1.18	10.89	7.33
1247.47	324.82	2268.78	1008.25	17.94	1.78	15.91	8.60
2787.42	130.07	1182.33	1876.18	58.41	2.35	7.44	6.22
911.52	283.86	1090.21	655.22	14.39	0.98	9.31	5.70
2842.31	277.62	6862.20	1204.84	36.58	1.27	10.43	7.79
50.79	502.07	72.06	39.35	2.53	13.25	9.76	1.89

193.99	2149.16	123.93	58.94	2.27	25.59	17.28	2.31
161.68	2680.63	135.13	78.13	0.65	15.90	14.30	1.58
2382.42	259.59	2045.32	952.96	23.52	1.90	9.56	6.30
4276.25	439.05	6858.09	1344.96	16.99	3.12	11.86	7.72
4586.92	161.30	3086.82	1239.82	22.26	1.36	11.91	6.98
153.77	471.20	74.52	14.37	0.43	8.18	4.34	1.71
2817.55	119.28	2017.32	674.73	16.02	0.32	10.83	4.56
1217.22	90.55	3581.16	751.73	11.21	0.77	7.23	4.49
1133.15	272.74	2289.97	481.10	11.04	0.34	12.18	8.11
7302.94	733.55	12649.44	1298.45	21.98	1.71	18.38	13.45
360.12	139.96	117.84	890.26	22.47	0.98	17.27	10.75
2751.86	132.01	634.72	1474.19	46.52	0.69	21.91	13.21
14731.39	376.89	5999.82	1843.28	28.38	2.69	15.38	11.44
142.34	930.26	120.16	62.20	4.84	23.15	15.97	2.52
1439.70	45.53	3771.95	728.80	16.63	0.05	9.06	5.16
2973.67	256.77	11217.52	1222.22	25.49	1.72	9.18	7.03
2027.86	122.24	591.34	601.96	32.08	0.05	24.92	14.52
5802.69	312.84	5704.91	686.93	12.41	0.53	13.74	9.71
1632.46	188.17	3874.71	1138.83	13.94	2.77	10.19	6.35
122.24	591.34	86.53	36.98	0.05	21.67	13.00	1.91
2487.72	275.85	2045.79	1125.24	23.67	2.61	10.59	6.79
4945.89	282.43	5077.78	1149.49	21.51	0.88	13.02	7.63
2808.62	242.56	6638.04	1317.10	22.25	1.11	11.75	6.36
549.87	114.71	1367.95	770.12	15.26	2.18	8.73	5.98
2237.98	142.39	3995.07	630.90	16.46	0.39	9.65	5.09
3916.41	232.37	9225.19	1384.28	19.30	4.11	12.04	7.97
7648.06	309.57	7209.29	2396.46	120.16	0.95	11.46	13.67
2222.93	162.26	2248.90	894.07	17.79	1.27	10.51	6.53
5990.87	358.97	4022.45	931.09	12.64	1.60	9.98	7.41
939.47	168.39	1611.54	1065.08	25.05	2.43	15.89	8.31
1749.92	338.12	5602.55	1580.68	16.01	2.50	14.49	8.49
161.68	2680.63	135.13	78.13	0.65	15.90	14.30	1.58
2023.17	174.81	4833.87	1058.72	15.58	2.70	13.49	10.19
3346.42	87.79	3186.35	1385.52	54.31	1.68	3.94	4.96
2403.85	382.61	4591.57	1513.29	14.01	2.66	10.96	7.41
927.82	383.05	6107.89	1207.52	19.55	3.65	12.85	6.80
4106.09	782.10	10881.13	1552.05	42.95	2.68	11.59	8.74
1712.28	279.31	7564.91	1591.06	35.84	3.37	11.17	6.77
2399.70	308.91	4115.82	872.69	19.19	0.49	10.73	6.73
2277.99	346.19	2383.29	1204.44	30.41	1.84	12.58	9.87
3487.09	425.91	5005.00	1296.78	27.34	2.63	8.76	5.12
686.92	131.21	1368.29	703.82	15.59	3.07	9.96	6.38
2371.65	475.69	7088.98	1497.12	14.19	3.67	12.90	8.12
2613.70	180.01	3450.80	1237.99	18.23	2.52	8.72	5.07
1276.71	185.91	2320.74	589.26	10.80	0.05	11.52	7.24
2640.71	359.18	4714.96	810.36	15.68	0.35	10.32	7.15
116.67	4295.94	75.49	21.32	0.44	7.27	4.72	1.50
5092.13	249.33	4213.00	1484.05	22.27	2.87	13.40	8.41
1436.84	101.55	2142.45	1288.40	23.10	1.67	9.47	7.23
1571.02	139.24	7831.80	932.94	19.23	0.28	10.20	5.80
960.67	132.71	235.68	1398.34	18.70	2.50	8.37	4.36
2384.54	132.12	3439.49	983.88	32.02	0.93	7.55	5.64
4442.90	167.65	4315.80	1403.95	50.55	1.26	7.75	7.68

2680.84	31.29	3649.47	958.46	15.64	0.42	5.46	3.53
1876.53	335.91	4255.51	1132.70	22.38	0.30	12.01	5.47
1943.20	274.43	6869.39	1416.59	25.40	1.70	14.09	8.11
127.44	678.02	140.01	55.39	1.18	7.79	7.31	1.56
3346.42	87.79	3186.35	1385.52	54.31	1.68	3.94	4.96
3428.11	295.50	5865.07	1567.09	55.69	1.93	9.16	7.62
1181.68	64.02	4013.64	708.48	20.08	0.25	12.99	7.98
1297.89	44.69	3663.94	652.19	16.91	0.70	9.18	5.84
596.68	109.45	1253.44	918.69	15.30	3.21	7.62	4.98
3918.45	566.43	3355.07	1621.34	18.96	3.10	10.28	5.96
100.24	1151.23	67.49	18.09	3.19	8.50	5.19	1.68
31.96	434.40	73.01	20.64	2.34	15.40	9.99	1.81
171.80	2587.50	206.85	71.75	1.02	6.89	8.22	1.13
5009.32	180.35	4100.43	1621.49	72.18	1.22	8.00	9.02
2003.86	66.61	502.07	1160.20	39.91	2.58	13.43	9.92
2438.72	449.23	10805.56	2885.09	22.36	9.49	14.83	9.91
7759.30	394.98	6642.24	1443.98	19.33	2.55	13.69	9.28
296.62	1468.47	249.31	117.97	2.77	9.12	11.90	1.33
1707.25	138.37	3822.76	1038.31	16.45	2.95	11.52	10.34
2303.65	519.75	6637.85	961.85	15.00	2.50	10.30	6.34
3136.29	297.70	5005.00	1878.98	17.26	4.86	13.84	9.39
173.98	1665.75	117.40	57.82	1.76	22.53	14.51	1.93
512.21	56.39	3530.33	974.36	19.73	0.63	7.73	4.42
275.09	2045.79	125.75	22.07	2.46	11.37	7.46	1.28
3045.47	136.54	634.81	1127.47	45.77	2.07	15.13	10.01
2068.37	410.84	7234.56	637.28	16.23	0.29	14.78	10.42
43.03	502.58	61.18	23.29	5.15	14.78	8.55	1.47
3039.33	265.84	5441.01	1018.35	25.33	0.95	14.40	8.42
155.29	471.20	75.10	14.95	0.44	8.04	4.34	1.85
1177.88	234.63	3600.05	923.44	15.46	1.29	6.02	3.13
728.16	134.35	3311.82	1020.86	9.64	4.70	7.50	4.07
1008.39	40.98	401.59	847.54	19.41	4.01	13.57	8.15
618.00	121.49	1383.58	842.55	16.35	2.18	9.45	6.21
40.22	401.59	53.41	20.57	3.91	9.75	6.38	1.21
1053.75	123.67	1368.29	742.59	17.20	2.92	10.11	6.30
267.14	1468.47	235.59	116.15	2.45	9.02	11.87	1.30
2237.91	295.00	1605.81	848.23	12.77	1.08	9.09	5.64
991.83	289.28	6740.21	541.62	14.45	0.10	5.71	3.08
4993.23	302.40	7075.24	934.31	18.60	0.86	14.07	9.29
7281.18	649.97	6492.83	2268.40	84.32	2.26	12.43	11.92
176.10	3185.14	102.74	22.49	1.69	17.54	9.45	1.89
293.40	4844.96	193.10	32.28	1.61	9.48	7.19	1.20
1189.13	324.46	3610.09	556.84	15.16	0.43	10.39	5.58
202.44	2465.49	74.61	27.09	2.13	13.49	8.02	2.01
638.47	180.00	1510.85	1105.37	20.93	3.47	13.02	7.52
2501.42	257.43	4844.45	1280.79	30.52	1.63	7.37	5.50
1760.50	169.62	1661.77	564.90	14.16	0.65	5.40	3.16
2277.99	346.19	2383.29	1204.44	30.41	1.84	12.58	9.87
2753.97	145.56	1631.58	1080.32	26.99	2.06	16.14	9.36
1245.93	382.61	1647.95	902.92	23.27	0.73	13.00	6.98
2250.65	643.69	11216.75	746.55	17.84	0.10	15.26	8.36
3633.40	440.68	11262.32	1696.72	12.22	1.00	10.24	6.87
134.99	2187.37	71.44	19.38	2.94	9.37	5.56	1.75

560.21	68.32	7316.65	662.23	13.36	1.94	7.35	4.90
1156.56	212.24	3987.51	714.66	16.68	1.10	10.62	6.73
6729.68	477.51	4366.62	1365.38	19.06	2.46	17.14	11.17
117.74	3858.81	90.27	36.02	2.41	16.75	10.73	2.03
2491.36	195.87	1632.63	711.78	18.94	0.10	8.10	4.93
238.21	2327.65	97.64	18.59	3.69	17.95	11.14	1.56
1327.98	132.24	1424.01	1059.92	30.32	3.30	12.83	7.75
3362.33	392.64	4089.68	762.09	15.43	0.58	25.06	15.68
1226.55	51.05	4616.42	851.35	19.80	0.71	9.80	5.84
156.00	3927.83	93.46	23.86	2.92	12.30	7.56	1.99
386.48	6330.12	137.78	25.61	2.64	12.43	6.93	1.68
1019.85	44.95	604.62	853.60	20.16	3.48	15.91	9.05
1513.20	81.07	3678.44	1087.59	14.95	1.28	8.99	5.89
2081.46	193.79	5755.29	954.70	19.34	0.93	7.76	4.77
3737.64	177.78	3288.36	1298.70	53.22	0.84	7.81	7.33
5261.35	105.41	4566.28	1122.18	21.80	2.04	13.37	8.46
2373.95	106.09	1885.04	1238.58	49.62	0.53	24.93	14.85
3346.42	87.79	3186.35	1385.52	54.31	1.68	3.94	4.96
2371.65	475.69	7088.98	1497.12	14.19	3.67	12.90	8.12
1125.42	161.76	6740.32	853.87	17.15	0.61	12.58	6.80
2501.42	257.43	4844.45	1280.79	30.52	1.63	7.37	5.50
3461.98	160.99	2146.81	884.33	19.73	0.82	10.58	6.47
1298.22	163.85	576.65	1038.94	17.22	2.70	11.15	7.70
4064.99	194.86	3819.47	1174.96	19.49	0.86	11.54	8.09
3428.11	295.50	5865.07	1567.09	55.69	1.93	9.16	7.62
2464.69	145.44	4584.10	1003.05	40.32	0.19	23.46	13.42
7064.49	200.78	1888.73	2327.75	117.94	1.35	8.43	11.53
208.98	4517.01	74.18	25.39	2.11	15.37	8.27	2.64
2190.43	125.24	3858.98	1499.27	49.08	2.51	20.37	11.93
960.31	210.84	6093.57	849.33	16.43	1.62	13.46	8.42
3335.79	162.27	4961.70	1441.20	24.01	2.95	13.50	8.26
8370.45	267.14	1468.47	2323.56	116.15	2.45	9.02	11.87
1333.73	110.45	2763.45	877.33	15.71	0.10	5.60	3.12
4311.53	165.89	4315.80	1325.19	52.35	0.62	7.88	7.65
5145.32	365.02	4116.91	1237.08	17.24	0.35	8.89	6.27
882.42	192.94	3917.70	810.35	21.80	3.72	12.86	7.90
7249.90	745.90	6896.51	2353.45	88.17	2.83	14.61	13.04
2978.99	348.45	4817.26	836.73	21.90	0.71	10.87	6.34
560.33	56.07	3530.33	842.67	15.05	0.63	7.18	4.22
2593.59	161.95	7114.45	1033.54	22.03	1.79	11.69	6.83
3044.23	367.27	7169.47	1595.86	24.73	2.93	14.43	8.91
1240.91	653.31	5821.70	826.83	20.95	0.76	13.79	7.48
9309.69	690.96	8781.70	2749.94	137.05	2.88	13.43	16.21
1094.68	147.12	10168.24	1249.04	12.09	2.35	11.10	7.01
1421.26	170.62	2348.19	791.43	14.95	2.57	9.61	6.74
2560.82	225.60	3822.34	917.34	14.58	0.38	10.70	6.47
7012.02	212.26	2028.48	2411.11	116.17	1.78	8.51	12.00
201.73	4100.26	217.07	72.55	1.22	8.28	9.03	1.15
2475.78	349.29	450.00	692.11	14.55	0.35	9.95	6.88
1381.60	262.88	1540.19	1233.53	15.54	2.35	10.85	5.94
8134.38	273.22	1468.47	2421.29	121.21	2.45	10.35	12.27
2123.14	118.13	3858.81	1229.73	39.67	2.42	18.34	10.92
1727.81	195.31	2548.88	963.91	15.47	2.47	9.91	4.57

2291.71	124.64	4047.78	662.77	15.91	0.33	9.08	5.70
967.56	252.99	2846.32	2065.28	15.51	9.20	10.36	6.90
151.56	4009.68	197.95	53.21	0.60	8.03	7.49	1.38
1232.03	237.49	2327.65	774.23	17.25	1.60	12.74	8.51
3154.19	135.96	634.81	1992.55	49.01	2.08	16.34	10.06
2003.47	124.49	3858.98	1261.35	42.79	2.41	18.68	11.14
1436.84	101.55	2142.45	1288.40	23.10	1.67	9.47	7.23
2544.37	155.16	3792.17	1214.01	17.95	1.63	10.10	6.46
1590.12	101.53	4013.95	975.58	14.52	1.51	9.18	5.22
559.90	77.16	3530.33	1116.49	19.50	0.69	8.84	4.85
1043.46	123.24	1368.29	731.43	16.86	2.67	9.85	5.96
2012.84	72.93	502.07	1132.82	40.06	2.53	16.81	11.24
5196.19	200.07	2871.17	1731.76	71.88	1.56	7.81	9.14
3739.51	264.62	4829.19	1687.99	54.24	2.33	8.85	7.61
2199.37	423.98	3577.73	1507.44	23.83	2.66	14.32	8.40
1198.21	111.49	3771.88	1113.14	18.03	1.07	9.54	5.47
2365.85	185.02	1558.08	1670.44	46.50	1.81	17.19	9.98
3346.42	87.79	3186.35	1385.52	54.31	1.68	3.94	4.96
2328.99	209.70	3808.74	1213.60	31.47	1.12	7.36	5.50
3045.07	327.91	1831.27	822.06	15.13	1.18	10.97	7.73
292.61	4844.96	160.07	34.48	1.29	9.56	7.12	1.57
1068.98	151.04	2374.00	730.96	26.22	1.66	12.30	7.48
3938.74	188.44	4702.33	1052.86	19.37	2.65	9.08	6.29
2942.82	145.83	3889.69	728.83	18.74	0.62	17.22	11.14
560.33	56.07	3530.33	842.67	15.05	0.63	7.18	4.22
2594.40	300.50	4264.32	1390.63	19.55	2.72	11.25	7.30
1966.79	59.47	388.20	1239.64	40.52	2.45	13.60	9.90
2546.33	142.79	5850.89	1103.80	19.01	0.31	11.44	6.85
1570.50	248.32	4116.72	566.63	13.34	0.70	12.06	7.85
1235.43	312.88	2136.85	944.30	11.77	1.30	8.39	5.68
1905.42	417.78	4035.00	1105.08	24.79	0.30	13.86	7.67

thiamin	riboflav	niacin	vit.b6	t.folate	df.folat	vit.b12	biotin
1.72	1.84	21.62	1.86	482.60	488.28	2.85	39.19
1.44	2.46	22.92	2.71	621.21	624.76	2.00	27.60
2.58	1.94	29.37	1.59	754.07	1005.14	2.18	17.80
1.12	1.57	16.00	1.74	396.71	429.80	2.44	21.86
1.90	1.30	22.87	1.78	488.46	625.55	2.21	23.20
2.34	2.36	25.20	2.43	612.71	603.27	3.45	54.39
1.33	1.83	18.89	2.05	539.14	613.11	2.56	23.97
2.22	2.48	21.46	2.22	458.07	507.64	4.90	40.75
1.73	19.41	2.03	566.38	674.79	2.63	26.91	5.18
2.10	2.12	21.55	1.65	462.75	583.89	3.25	33.53
1.89	23.75	1.65	452.71	527.53	5.83	33.41	5.36
2.52	3.16	25.32	2.43	612.50	635.44	5.53	54.79
2.18	21.43	1.87	401.13	451.50	5.28	20.79	4.36
2.12	1.49	23.43	1.72	428.46	520.70	1.22	29.71
1.30	1.58	13.63	1.38	428.94	413.18	2.35	18.61
1.22	2.09	15.62	1.85	458.96	482.42	3.16	24.96
1.74	1.80	20.56	1.53	493.85	548.70	3.59	22.83
1.54	1.50	15.20	1.49	447.49	531.07	2.71	28.53
1.65	1.32	15.09	1.03	439.54	522.14	1.50	19.25
1.87	1.68	23.00	1.41	494.33	564.90	4.15	20.61
1.81	2.08	14.25	1.38	434.35	452.71	3.66	33.32
1.68	1.84	20.06	1.97	437.94	512.62	5.26	25.89
1.77	1.80	20.65	1.91	436.48	462.74	1.79	28.52
2.12	1.61	22.29	1.76	635.01	732.02	1.85	35.71
1.44	2.46	22.92	2.71	621.21	624.76	2.00	27.60
1.80	1.63	29.34	1.94	466.02	607.16	5.16	26.56
1.78	2.71	21.45	2.26	475.71	467.16	4.23	45.34
2.43	2.45	24.69	2.67	737.59	728.50	3.22	52.97
1.35	1.63	22.35	1.45	372.29	412.57	5.54	22.59
1.23	1.25	20.33	1.47	370.78	434.57	3.53	16.47
1.30	1.56	18.84	1.55	372.46	439.00	4.63	21.96
1.75	1.81	27.16	2.00	496.22	582.96	5.88	22.27
1.26	1.95	17.43	2.10	575.54	591.50	2.87	27.74
2.26	1.44	25.59	2.35	630.64	721.00	1.46	38.68
1.59	20.44	1.71	441.71	443.71	2.79	28.97	4.39
1.62	19.21	1.99	415.30	442.48	1.96	24.65	3.76
1.70	1.51	21.76	1.50	505.54	627.47	2.33	20.26
1.90	1.92	20.64	2.01	504.56	507.56	3.77	35.76
1.11	1.00	12.28	1.14	436.95	456.57	1.57	20.28
1.46	1.64	17.43	1.34	529.46	641.63	2.65	13.11
1.99	1.44	19.89	1.53	672.27	920.78	1.86	15.33
1.39	1.83	26.54	1.97	461.24	537.84	5.39	28.51
1.75	2.05	17.48	1.38	500.57	499.76	4.31	24.87
1.90	1.48	18.24	1.56	611.56	734.77	2.09	28.74
1.78	1.88	21.06	2.06	490.65	467.49	2.90	34.25
1.90	1.16	20.53	1.35	402.38	458.59	1.14	25.11
1.89	1.53	20.47	1.70	506.88	604.10	2.06	22.15
0.00	0.00	0.00	0.00	0.00	0.00	0.00	0.00
1.92	1.71	16.61	1.41	544.52	666.17	2.30	18.56
2.53	23.53	2.28	524.37	523.11	9.45	54.24	7.20
2.51	3.27	35.53	2.44	730.15	739.20	6.59	42.33
1.26	1.62	16.47	1.82	449.76	521.14	2.86	21.85

2.04	1.82	19.29	1.47	440.60	477.99	2.77	34.54
2.15	1.35	28.63	1.77	540.08	648.82	1.71	22.23
2.05	1.23	20.82	1.68	561.85	719.66	1.38	24.99
1.22	15.60	1.61	371.71	385.76	1.83	22.70	3.43
1.88	22.57	1.75	486.13	560.42	2.94	23.02	4.58
2.97	4.06	26.98	3.19	815.88	784.30	5.89	72.31
1.57	16.00	1.74	396.71	429.80	2.44	21.86	3.94
2.81	1.80	30.60	1.89	622.68	707.29	1.14	38.42
1.36	1.15	14.68	1.10	473.76	520.90	3.51	8.77
2.30	22.55	1.82	466.34	532.03	6.37	24.63	5.92
1.89	19.56	2.07	439.85	476.91	2.14	23.84	3.89
1.11	1.22	15.60	1.61	371.71	385.76	1.83	22.70
2.92	2.49	25.10	2.13	910.57	1056.35	3.91	41.01
2.33	3.59	40.41	3.76	856.81	879.55	3.62	54.13
1.80	1.24	20.38	1.37	527.42	661.56	1.50	17.65
2.18	21.43	1.87	401.13	451.50	5.28	20.79	4.36
2.69	20.25	2.44	604.10	630.48	2.86	26.65	4.44
1.61	1.32	20.21	1.64	477.92	586.71	1.82	23.62
1.46	1.64	17.43	1.34	529.46	641.63	2.65	13.11
1.63	2.01	24.19	1.87	495.44	549.78	4.78	25.42
2.46	28.54	2.82	699.38	696.54	5.70	49.13	7.14
1.94	23.94	1.88	600.52	637.06	4.36	25.00	5.12
1.42	0.77	13.18	1.12	413.53	519.33	0.80	22.36
1.87	1.82	20.63	1.62	523.43	600.15	2.54	29.76
1.92	32.65	2.12	517.44	590.09	3.97	41.83	5.72
3.50	2.13	38.68	2.67	906.70	1115.62	3.09	36.86
1.80	1.58	21.51	1.74	429.84	435.46	2.32	32.55
2.57	1.89	27.34	1.84	699.95	852.52	3.47	30.96
1.92	20.61	1.85	617.70	684.55	2.50	22.46	3.80
2.55	3.31	36.57	3.46	910.03	961.21	2.34	49.99
2.83	35.35	2.93	810.39	815.17	3.65	53.15	7.52
1.51	1.70	26.07	1.81	399.85	483.77	5.71	19.13
1.89	1.69	21.77	1.90	480.12	560.41	4.88	22.99
2.88	2.35	38.08	2.70	631.55	454.01	5.21	43.46
1.76	21.25	2.16	601.51	607.86	2.41	36.66	4.91
2.68	2.18	28.26	2.05	558.69	621.90	3.40	43.60
1.55	1.72	15.85	1.59	424.32	427.71	4.57	32.80
2.42	1.63	26.03	2.22	629.60	677.37	2.31	32.39
0.87	1.27	12.19	1.14	375.69	415.21	2.52	24.92
1.26	1.69	20.59	1.65	376.18	454.51	6.22	25.74
1.60	28.51	1.91	460.90	504.50	4.73	21.09	4.56
1.55	16.96	1.86	502.67	504.85	2.37	28.61	4.24
2.06	1.97	28.98	3.39	524.11	566.05	3.89	99.16
1.67	2.00	15.27	1.52	477.88	562.37	3.86	21.78
1.29	1.58	24.25	1.85	524.25	580.75	3.39	24.42
2.36	2.04	24.17	2.05	683.40	687.22	2.43	37.08
1.99	0.91	26.06	1.80	580.69	772.74	1.74	18.16
1.67	1.59	17.98	1.61	484.70	549.93	2.42	31.33
2.04	1.85	22.88	1.84	488.24	576.20	4.59	22.30
2.33	2.40	24.47	2.23	649.09	802.73	3.70	24.74
1.81	1.07	18.42	1.23	542.27	690.35	2.06	14.03
1.64	1.76	21.25	2.16	601.51	607.86	2.41	36.66
1.72	20.83	1.57	561.50	665.41	3.48	20.91	4.63

2.59	33.51	2.22	665.25	842.70	3.65	20.44	4.53
2.19	22.75	2.08	503.74	569.56	6.15	14.63	3.90
1.38	1.70	23.02	1.83	563.95	597.88	3.91	22.69
1.90	2.25	21.79	2.14	470.55	462.89	3.59	49.88
2.53	2.26	24.31	2.19	684.49	694.06	3.16	47.44
1.01	24.62	1.66	454.31	545.47	1.66	19.48	3.68
1.58	1.03	17.27	1.45	582.15	681.49	1.19	18.54
1.34	1.48	20.12	2.03	356.97	323.99	2.28	36.59
1.34	0.88	16.38	1.43	416.59	498.64	1.69	20.49
2.17	2.37	25.18	2.79	641.74	551.86	2.76	46.31
2.64	1.49	30.87	2.14	856.28	1000.13	2.16	23.98
2.09	2.03	29.96	2.06	598.09	722.59	2.92	17.81
2.83	3.33	27.48	3.13	949.03	1025.18	4.05	59.27
2.73	25.78	1.68	792.66	1022.86	3.09	46.33	6.53
2.01	1.39	23.15	1.81	453.27	486.18	1.88	32.16
2.34	2.59	26.58	2.49	706.44	710.00	10.55	46.62
1.17	0.76	15.32	1.10	448.81	535.48	1.08	10.43
1.30	1.37	17.42	1.66	435.98	470.51	2.29	29.19
1.73	1.91	17.98	1.75	447.02	462.77	3.46	36.00
1.14	21.51	1.56	580.23	718.40	1.41	16.59	3.94
1.75	1.91	26.79	2.12	577.37	689.96	4.33	26.04
2.31	2.42	29.49	2.89	703.31	696.38	4.28	55.66
2.59	2.36	24.03	2.29	698.07	759.99	3.57	43.03
1.51	1.51	20.50	1.63	429.18	551.87	5.35	18.36
1.98	1.24	21.50	1.66	507.12	593.29	1.65	29.29
2.30	2.46	28.19	2.44	588.63	608.75	4.80	52.99
1.56	2.60	23.68	2.83	720.65	718.50	2.26	33.60
1.93	1.76	22.74	1.87	596.79	627.20	3.14	31.83
1.45	1.55	17.35	1.80	493.80	508.75	2.57	33.52
2.39	2.08	24.63	1.53	552.49	623.08	4.08	30.74
1.93	2.61	17.34	1.98	517.45	480.28	4.79	47.70
2.19	22.75	2.08	503.74	569.56	6.15	14.63	3.90
1.23	1.88	13.37	1.47	404.03	407.36	3.67	34.74
1.40	2.18	21.43	1.87	401.13	451.50	5.28	20.79
1.83	2.52	19.08	2.12	535.60	548.10	4.73	41.25
1.87	2.09	23.66	1.89	524.57	634.28	5.91	25.80
2.16	2.50	27.91	2.49	722.13	745.63	3.66	53.93
1.93	2.43	24.46	2.06	582.93	700.07	5.97	24.60
2.17	1.64	24.18	2.03	612.52	682.28	2.47	35.40
1.33	1.74	15.81	1.81	433.10	415.83	3.43	24.54
3.02	2.42	30.41	2.25	846.45	875.50	2.57	45.48
1.35	1.53	24.39	1.66	372.90	445.28	4.81	24.00
1.85	2.62	19.43	1.95	571.82	615.42	4.36	43.31
1.89	2.41	23.97	2.50	910.27	867.35	5.51	31.26
1.50	0.92	16.62	1.44	391.75	470.84	1.57	16.17
1.90	1.33	18.51	1.73	491.82	580.64	1.75	30.33
1.16	15.42	1.21	420.59	467.85	1.20	21.63	3.14
2.41	2.64	26.50	2.65	716.85	774.29	5.57	54.25
1.64	2.05	16.61	1.75	426.40	452.43	4.42	25.69
2.24	1.57	24.13	1.94	546.44	609.89	2.39	39.66
2.57	2.32	30.41	1.88	666.75	859.83	3.85	24.62
1.34	1.33	23.41	2.00	417.82	462.21	1.99	24.55
1.03	1.76	16.77	1.91	395.48	422.63	2.53	23.74

1.95	1.89	21.66	1.90	415.14	471.00	3.44	29.65
2.17	1.90	24.37	2.46	739.38	729.43	2.62	36.34
2.99	2.48	30.77	2.39	836.77	941.46	3.24	44.02
1.92	20.61	1.85	617.70	684.55	2.50	22.46	3.80
1.40	2.18	21.43	1.87	401.13	451.50	5.28	20.79
1.53	1.95	22.29	2.12	605.90	693.99	2.73	25.35
2.33	1.44	24.13	1.60	635.47	798.07	1.43	23.81
1.99	1.33	24.44	2.08	545.31	647.64	2.36	30.26
1.46	1.78	25.86	1.80	365.45	442.45	5.91	25.58
2.20	2.92	23.36	2.39	621.92	600.08	4.71	45.91
1.73	26.18	1.70	405.31	494.39	4.87	31.45	5.05
2.04	22.56	1.57	513.90	595.95	6.35	26.17	3.98
1.81	18.87	2.01	435.28	487.95	2.05	19.12	3.71
1.14	1.89	17.28	1.94	440.39	478.92	2.06	24.04
1.96	1.77	21.47	1.61	571.78	674.06	3.55	22.65
2.92	4.82	26.87	2.78	738.72	701.82	9.13	87.52
2.22	2.45	24.59	2.58	675.77	724.98	3.70	48.67
2.58	25.52	2.56	648.45	659.04	3.21	27.56	4.02
1.78	2.11	24.24	2.07	538.19	572.30	3.68	48.09
1.89	2.08	22.83	1.56	590.57	708.02	3.31	33.44
2.24	3.11	24.40	2.39	618.62	709.54	5.18	41.07
1.92	24.67	1.92	542.90	630.11	2.51	30.02	5.01
2.01	1.62	23.38	1.79	783.41	849.99	2.11	30.30
1.57	23.62	1.79	520.22	574.65	3.39	25.20	4.69
1.75	1.73	22.89	1.58	590.22	558.88	3.08	21.26
1.45	1.02	16.36	1.80	495.16	594.35	1.28	25.90
1.71	22.21	1.46	393.85	435.48	5.67	26.68	4.55
2.80	2.11	29.70	2.39	708.97	807.17	2.49	41.69
1.24	24.84	1.69	515.90	608.79	2.20	20.73	3.89
1.67	1.68	19.78	1.47	418.18	428.34	2.39	28.48
1.06	1.80	11.55	0.93	306.23	340.18	3.00	25.98
1.06	1.47	17.03	1.21	331.20	371.45	5.03	19.60
1.56	1.70	25.11	1.89	407.53	490.22	5.81	24.53
1.51	18.35	1.25	359.31	377.69	4.85	19.69	3.89
1.53	1.62	26.11	1.76	401.35	471.95	4.87	25.35
2.46	25.83	2.49	544.39	572.42	2.91	20.62	3.57
1.47	1.35	17.26	1.55	431.67	439.25	2.53	23.15
1.52	1.15	18.67	1.53	446.23	456.77	0.99	34.04
1.78	1.94	21.62	2.00	527.91	522.25	2.36	35.46
2.58	3.41	39.01	3.47	810.65	837.36	4.19	66.89
1.83	28.66	2.23	428.81	487.22	4.67	25.29	5.27
1.81	16.96	1.91	491.44	499.13	2.80	29.33	4.57
1.83	1.05	18.85	1.55	569.43	660.09	1.09	32.07
1.67	22.53	1.62	589.96	740.32	2.85	17.42	4.64
1.95	2.03	28.26	2.09	505.80	640.85	6.16	23.75
1.10	1.73	13.24	1.62	412.50	438.49	3.03	23.49
1.49	1.26	15.40	1.27	348.07	347.85	1.71	26.35
1.33	1.74	15.81	1.81	433.10	415.83	3.43	24.54
1.92	1.90	18.98	1.48	577.72	679.92	4.42	27.56
2.70	1.77	27.24	2.02	575.14	647.55	1.81	39.62
1.93	1.18	23.00	2.24	658.72	838.13	1.07	35.52
1.95	2.55	16.28	2.58	608.77	608.47	5.34	47.82
1.77	27.36	1.74	409.66	468.90	4.53	34.93	5.02

1.62	1.30	18.68	1.14	477.92	625.26	2.41	18.92
1.75	1.59	22.17	1.75	399.22	442.75	2.22	34.80
1.99	2.42	25.08	2.53	650.13	641.24	4.36	49.15
1.96	22.08	1.32	663.60	818.30	2.21	22.91	4.67
2.26	1.25	22.83	1.86	564.68	639.26	1.85	29.09
1.88	18.63	1.37	474.53	537.07	5.25	27.90	4.65
1.88	2.01	31.82	2.19	486.94	538.54	5.47	33.72
1.76	1.51	17.99	1.77	522.75	573.48	1.94	34.07
2.22	1.67	23.51	1.92	567.10	642.79	2.57	36.20
1.82	27.62	1.95	507.03	608.63	5.70	37.48	5.75
2.08	26.54	1.91	513.82	586.89	6.56	25.34	5.34
1.18	1.48	18.17	1.30	336.97	385.13	4.96	18.75
2.04	1.92	22.11	2.00	518.48	568.67	3.57	33.97
2.38	1.63	23.91	2.01	529.93	599.21	2.44	38.05
1.38	1.58	19.74	1.98	450.65	463.82	1.78	27.24
2.61	2.21	26.11	1.94	667.97	713.99	2.61	41.87
2.26	2.01	25.85	1.70	532.40	613.31	1.58	27.34
1.40	2.18	21.43	1.87	401.13	451.50	5.28	20.79
1.85	2.62	19.43	1.95	571.82	615.42	4.36	43.31
2.04	1.52	29.81	2.12	570.82	653.37	2.27	32.15
1.10	1.73	13.24	1.62	412.50	438.49	3.03	23.49
2.13	1.85	25.49	2.00	580.65	633.24	3.09	39.26
1.31	1.65	11.58	1.05	393.96	434.84	2.87	21.58
2.38	2.11	26.55	2.35	636.14	701.86	3.07	39.45
1.53	1.95	22.29	2.12	605.90	693.99	2.73	25.35
2.21	1.74	25.32	1.76	495.50	465.63	1.20	41.25
1.41	2.59	23.15	2.43	614.67	628.85	2.45	27.18
2.32	23.44	1.44	681.19	868.61	3.42	24.81	5.32
3.47	2.67	33.51	2.09	803.94	930.10	1.87	49.01
1.21	1.27	15.54	1.39	375.79	426.21	2.51	24.13
2.77	2.90	37.98	2.87	735.12	781.80	4.66	64.06
1.30	2.46	25.83	2.49	544.39	572.42	2.91	20.62
2.08	1.53	19.25	1.61	550.80	623.80	2.26	28.01
1.27	1.62	19.21	1.99	415.30	442.48	1.96	24.65
1.92	2.12	21.75	2.37	475.50	460.67	3.44	40.75
1.97	1.80	26.86	1.94	519.75	589.16	4.70	39.68
2.93	3.68	37.62	3.49	981.40	1018.77	4.09	61.83
2.52	1.63	28.64	2.27	590.88	662.91	1.84	44.74
1.59	1.41	20.24	1.62	603.13	642.08	2.11	26.85
2.55	2.12	25.81	2.19	729.82	884.16	2.40	39.99
2.74	2.94	29.49	2.66	669.62	652.48	4.04	63.95
2.17	1.54	29.99	2.83	737.98	818.64	1.58	44.40
2.36	3.49	37.04	3.48	819.94	878.47	3.75	45.58
1.55	2.00	18.78	2.18	476.96	544.08	3.83	42.15
1.37	1.52	20.00	1.63	407.19	468.77	4.14	25.72
1.62	1.75	17.09	1.60	441.61	436.83	2.43	33.77
1.25	2.69	20.25	2.44	604.10	630.48	2.86	26.65
1.89	19.56	2.07	439.85	476.91	2.14	23.84	3.89
1.77	1.20	15.28	1.62	562.61	707.61	1.54	21.54
1.80	2.10	16.93	1.86	442.05	451.76	3.44	32.53
1.86	2.75	31.63	2.80	631.07	657.20	3.09	28.73
2.19	2.01	25.67	1.79	658.27	692.76	2.48	34.53
1.52	1.49	20.45	1.63	660.59	760.56	2.45	17.82

1.92	1.36	23.20	1.84	521.22	606.97	1.77	28.76
2.05	3.54	20.59	1.82	519.05	598.05	6.25	49.36
1.61	20.03	2.05	452.45	498.21	2.02	23.57	3.91
1.36	1.37	17.18	1.33	409.18	433.96	3.22	19.27
2.71	2.66	24.69	2.34	700.95	823.87	5.05	33.21
2.82	2.05	28.69	2.01	746.15	907.96	2.16	36.92
1.64	2.05	16.61	1.75	426.40	452.43	4.42	25.69
2.24	2.19	20.84	1.99	580.40	652.25	3.32	38.17
1.70	1.66	16.02	1.61	436.18	442.28	2.43	37.13
2.58	1.94	29.37	1.59	754.07	1005.14	2.18	17.80
1.53	1.38	25.37	1.80	465.89	605.18	4.42	18.36
1.94	1.77	22.54	1.60	553.40	647.44	3.36	22.03
1.09	2.11	16.70	2.02	417.36	419.55	2.49	23.53
1.29	2.19	17.12	2.04	585.86	612.21	3.27	29.30
2.48	2.90	27.53	2.46	698.42	633.75	4.10	52.01
2.34	2.01	26.88	1.80	528.32	578.31	3.02	29.26
3.53	2.63	35.88	2.49	852.45	1095.36	3.47	35.11
1.40	2.18	21.43	1.87	401.13	451.50	5.28	20.79
1.26	1.62	16.47	1.82	449.76	521.14	2.86	21.85
1.44	1.53	14.77	1.63	404.67	422.17	2.12	29.82
1.73	19.41	2.03	566.38	674.79	2.63	26.91	5.18
2.22	1.80	29.20	1.95	485.88	529.61	5.03	41.39
2.23	2.01	23.25	1.98	585.91	625.78	2.59	40.17
2.03	1.37	24.45	1.94	616.87	690.55	2.05	33.93
1.59	1.41	20.24	1.62	603.13	642.08	2.11	26.85
2.49	2.43	24.57	2.25	591.32	656.37	4.17	46.91
2.00	1.94	23.56	1.58	562.93	653.27	3.58	22.14
2.40	2.05	26.23	2.10	540.70	636.75	2.75	32.11
1.27	1.11	18.53	1.53	396.90	439.18	1.37	22.01
1.59	1.51	16.43	1.55	426.92	514.69	3.01	19.64
2.75	2.05	35.77	2.86	833.74	878.94	2.83	55.25

pantho.a	vit.k	phospho	iodine	mg1	zinc	copper	mn
5.47	153.82	1395.01	#NULL!	480.19	12.70	1.86	8.47
3.97	1590.28	1357.42	#NULL!	540.40	12.24	1.80	15.68
4.03	221.11	1531.21	#NULL!	331.77	7.51	1.82	7.36
3.94	371.60	1059.65	#NULL!	286.10	7.39	1.00	5.97
4.34	95.58	1261.92	#NULL!	340.15	9.31	1.40	7.82
6.86	376.08	1980.19	#NULL!	724.24	19.03	2.54	10.15
4.61	663.10	1237.24	#NULL!	372.95	9.20	1.33	8.60
6.71	41.31	2019.61	#NULL!	467.49	13.46	1.60	8.39
374.72	1246.32	2501.00	327.81	9.13	1.31	7.11	69.74
5.19	95.00	1670.21	#NULL!	383.58	10.64	1.66	6.46
117.38	1529.88	1285.50	389.72	12.82	1.43	7.13	130.08
8.48	336.09	2295.60	#NULL!	616.73	15.84	2.08	10.73
632.44	1366.38	1580.47	378.02	13.60	3.12	9.70	111.62
4.30	90.97	1116.60	#NULL!	361.28	9.25	1.66	8.13
3.43	543.60	1085.37	#NULL!	348.61	8.83	1.04	9.59
4.25	660.80	1263.59	#NULL!	360.87	9.17	1.11	8.26
4.33	410.22	1254.06	#NULL!	319.15	10.29	1.29	6.77
4.90	144.04	1246.16	#NULL!	337.65	8.91	1.30	6.90
3.31	173.34	961.15	#NULL!	326.49	7.87	1.41	7.49
3.81	198.86	1100.50	#NULL!	304.86	9.73	1.44	6.19
5.45	69.85	1610.08	#NULL!	389.21	11.34	1.31	6.91
6.38	95.53	1404.29	#NULL!	314.62	11.82	1.29	4.61
4.39	791.43	1258.16	#NULL!	490.81	10.38	1.82	8.66
5.23	229.12	1468.99	#NULL!	481.48	11.82	1.94	8.24
3.97	1590.28	1357.42	#NULL!	540.40	12.24	1.80	15.68
4.94	66.99	1437.55	#NULL!	362.65	11.41	1.42	6.75
6.61	867.83	1758.09	#NULL!	559.30	14.02	2.07	8.20
7.71	1010.30	1870.47	#NULL!	632.94	14.88	2.62	10.56
4.13	200.66	1256.62	#NULL!	296.33	9.91	1.20	5.61
3.71	89.84	1073.59	#NULL!	255.61	8.45	0.83	3.82
4.36	60.20	1153.93	#NULL!	267.17	9.22	1.03	4.09
5.40	141.89	1480.86	#NULL!	361.35	12.25	1.54	6.30
4.49	665.18	1346.76	#NULL!	387.76	9.86	1.39	8.43
5.92	227.96	1457.32	#NULL!	505.70	11.79	2.13	10.57
195.71	1378.51	1150.21	373.77	10.47	1.29	7.45	110.80
680.07	1110.47	1387.54	362.69	8.98	1.23	9.01	71.46
4.02	88.99	1158.41	#NULL!	313.68	8.36	1.47	6.17
5.85	281.59	1527.66	#NULL!	430.11	11.18	1.53	7.68
2.93	175.78	1470.50	#NULL!	267.20	1.20	1.08	6.23
3.15	617.35	1200.00	#NULL!	312.95	9.63	1.48	8.56
4.22	438.43	988.30	#NULL!	288.46	8.49	1.34	8.53
4.91	406.92	1364.25	#NULL!	358.65	11.75	1.32	6.79
5.11	103.79	1552.14	#NULL!	348.79	10.57	1.31	5.78
4.40	221.64	1470.38	#NULL!	356.70	9.91	1.62	7.90
5.78	160.32	1417.57	#NULL!	428.52	11.41	1.86	6.02
3.81	97.49	1153.78	#NULL!	396.65	9.58	1.43	10.74
4.24	204.18	1206.90	#NULL!	322.29	8.65	1.71	4.73
4.21	107.52	1050.64	295.12	253.21	2.15	4.41	85.07
4.08	177.13	1033.53	#NULL!	267.47	7.95	1.56	3.44
148.37	1801.49	1670.31	493.55	13.46	2.30	9.01	136.12
7.87	125.87	2452.48	#NULL!	567.08	17.76	2.37	7.65
4.51	365.56	1129.13	#NULL!	286.02	8.03	1.03	6.03

4.81	145.82	1422.25	#NULL!	421.65	11.06	1.65	9.10
3.83	58.73	1353.77	#NULL!	360.38	10.29	1.64	6.92
4.21	143.13	1021.89	#NULL!	300.44	8.66	1.78	6.01
363.65	941.82	1560.31	282.49	7.13	3.02	6.12	56.80
683.68	1270.57	#NULL!	412.70	10.61	1.38	11.33	110.77
9.59	984.92	2731.47	#NULL!	793.48	2.15	2.69	11.31
371.60	1059.65	1565.50	286.10	7.39	1.00	5.97	51.56
5.40	386.01	1646.64	#NULL!	599.50	13.64	2.08	17.05
3.36	117.96	928.25	#NULL!	264.53	9.04	1.38	5.21
108.06	1759.43	1670.10	397.29	12.71	1.58	5.87	102.10
961.57	1133.26	1300.00	388.10	9.73	1.22	10.61	73.36
3.43	363.65	941.82	#NULL!	282.49	7.13	1.02	6.12
6.30	202.44	1952.80	#NULL!	504.10	14.53	2.28	10.21
7.31	1733.00	2245.07	#NULL!	811.16	19.35	2.48	19.32
3.65	103.52	995.86	#NULL!	268.65	7.48	1.37	5.88
632.44	1366.38	1321.20	378.02	13.60	8.64	9.70	111.62
1542.10	1415.90	1400.60	511.27	13.30	1.54	15.02	86.04
3.85	177.11	967.67	#NULL!	320.09	8.61	1.35	5.16
3.15	617.35	1350.64	#NULL!	312.95	7.15	1.48	8.56
5.29	197.76	1531.39	#NULL!	412.48	12.80	1.56	6.71
335.45	1503.96	1850.20	562.52	15.00	1.88	6.24	122.08
202.53	1532.14	1470.04	371.79	11.18	1.57	6.53	89.84
3.20	121.49	804.93	#NULL!	264.82	6.95	1.13	6.08
4.81	122.54	1236.28	#NULL!	351.97	9.54	1.73	5.98
72.15	1826.17	1521.30	538.68	14.35	8.50	10.64	184.78
6.99	77.54	2040.82	#NULL!	530.12	16.00	2.50	10.65
4.54	102.76	1405.60	#NULL!	427.24	10.46	1.54	9.43
5.44	210.95	1594.97	#NULL!	460.71	12.05	1.98	9.46
661.95	1342.19	1025.31	397.55	10.25	1.42	10.49	85.12
6.92	1665.04	2078.15	#NULL!	817.91	9.64	2.93	20.79
498.48	2230.95	1250.16	682.96	17.75	2.27	12.39	160.38
4.74	70.18	1360.80	#NULL!	302.85	11.30	1.14	4.81
5.85	105.81	1409.89	#NULL!	364.62	11.91	1.40	6.24
8.48	303.10	2013.28	#NULL!	595.39	17.29	2.90	8.50
403.59	1409.38	1400.26	424.78	10.46	6.50	8.52	89.66
6.53	39.12	1885.50	#NULL!	557.59	14.98	1.97	13.25
5.00	98.62	1624.31	#NULL!	345.46	10.00	1.29	6.21
6.37	98.67	1508.40	#NULL!	459.23	12.11	2.01	9.12
3.51	191.91	912.17	#NULL!	247.85	6.62	0.74	5.64
4.71	75.65	1263.48	#NULL!	285.05	9.84	1.07	3.88
118.01	1412.02	1453.21	360.67	12.27	1.32	7.30	132.72
378.08	1130.94	1830.50	320.43	8.18	1.21	6.17	54.69
5.63	570.60	1470.02	#NULL!	539.82	1.14	2.67	7.24
5.13	401.76	1308.25	#NULL!	314.01	10.30	1.15	7.01
4.76	205.39	1305.57	#NULL!	334.47	9.83	1.42	5.56
5.46	370.26	1609.97	#NULL!	523.02	12.06	2.06	10.07
4.44	68.48	1324.50	#NULL!	279.16	9.30	1.37	5.79
5.15	161.51	1252.14	#NULL!	368.05	8.98	1.52	5.52
5.78	90.48	1474.44	#NULL!	366.79	11.73	1.42	6.41
6.57	681.88	1752.73	#NULL!	507.47	13.79	1.52	14.04
3.72	91.54	910.55	#NULL!	268.66	7.25	1.32	5.14
4.91	403.59	1409.38	#NULL!	424.78	10.46	1.68	8.52
403.55	1231.37	1430.26	316.81	10.37	1.35	7.16	114.93

675.08	1604.42	#NULL!	423.71	11.77	1.67	10.80	112.09
967.62	1338.56	#NULL!	417.97	17.86	1.69	10.49	116.10
4.51	208.36	1411.59	#NULL!	352.27	10.46	1.52	6.28
6.62	261.79	1803.97	#NULL!	508.52	12.60	1.88	8.27
6.53	336.63	1882.49	#NULL!	566.16	14.21	2.16	11.66
55.77	1155.42	1152.12	297.63	9.05	1.27	5.96	99.75
3.86	127.71	1267.37	#NULL!	423.36	10.44	1.91	8.63
4.41	165.75	1202.51	#NULL!	349.79	8.32	1.25	5.98
3.75	114.39	827.98	#NULL!	252.27	6.74	1.24	5.26
6.26	880.84	1742.93	#NULL!	657.29	12.66	2.59	9.07
5.29	97.65	1544.44	#NULL!	429.66	11.89	2.10	9.25
4.18	548.58	1436.81	#NULL!	397.82	11.04	1.50	10.35
8.31	1005.09	2114.62	#NULL!	739.67	12.31	2.97	11.31
715.00	1508.76	1589.21	457.11	11.84	1.89	13.23	165.47
4.26	114.83	1401.15	#NULL!	455.00	11.83	1.71	10.20
6.76	455.25	1756.82	#NULL!	572.51	14.43	3.35	9.11
2.67	392.10	774.45	#NULL!	310.33	7.54	1.24	9.15
3.59	503.94	1022.37	#NULL!	379.50	8.61	1.41	7.12
5.73	152.57	1515.85	#NULL!	400.66	10.68	1.51	6.99
388.49	1138.32	#NULL!	423.25	10.84	1.68	12.32	127.79
5.74	205.68	1566.10	#NULL!	366.56	11.24	1.48	6.58
7.98	316.99	1908.06	#NULL!	637.75	16.44	2.89	10.35
6.93	163.24	1981.50	#NULL!	596.61	15.22	2.29	12.30
4.58	58.75	1173.66	#NULL!	279.32	9.57	1.21	4.80
4.49	108.75	1234.92	#NULL!	393.64	10.48	1.78	8.47
7.36	436.13	2035.21	#NULL!	598.90	15.15	2.02	10.71
4.58	1594.51	1544.02	#NULL!	598.86	13.61	2.02	16.73
5.29	327.76	1459.65	#NULL!	449.29	11.94	1.85	9.25
5.18	299.42	1226.46	#NULL!	370.75	9.73	1.43	5.22
5.44	121.41	1594.42	#NULL!	453.16	12.21	1.90	9.23
6.59	208.53	1905.18	#NULL!	505.68	13.52	1.87	8.21
967.62	1338.56	1600.00	417.97	17.86	1.69	10.49	116.10
4.76	138.36	1315.33	#NULL!	306.48	9.93	1.14	3.76
4.36	632.44	1366.38	#NULL!	378.02	13.60	1.41	9.70
6.71	328.67	1740.41	#NULL!	454.87	12.21	1.77	6.27
6.37	111.47	1609.69	#NULL!	349.45	12.06	1.33	5.44
7.71	487.16	1962.55	#NULL!	583.42	15.19	1.96	10.36
6.32	312.30	1824.46	#NULL!	439.23	13.52	1.76	7.27
5.78	300.61	1508.88	#NULL!	538.63	13.77	2.09	10.89
4.45	366.29	1240.47	#NULL!	357.09	9.44	1.13	7.09
7.31	323.13	1816.86	#NULL!	602.36	13.66	2.60	12.55
4.34	63.20	1230.89	#NULL!	298.65	9.93	1.13	4.76
6.67	233.32	1685.44	#NULL!	431.30	11.50	1.41	6.82
6.33	201.69	2134.36	#NULL!	607.47	14.95	2.57	9.50
3.48	153.39	917.86	#NULL!	262.94	7.24	1.02	5.69
4.63	289.89	1191.06	#NULL!	386.91	9.92	1.73	7.21
188.66	918.30	1590.61	307.82	7.40	1.13	8.55	92.13
8.11	453.78	2090.14	#NULL!	562.63	15.61	2.25	8.29
5.10	194.70	1585.31	#NULL!	402.55	10.36	1.21	8.44
5.59	99.16	1584.37	#NULL!	477.87	12.15	1.76	11.00
5.18	60.34	1604.62	#NULL!	302.04	10.89	1.59	4.35
3.81	361.89	1151.91	#NULL!	328.72	8.44	1.23	7.03
3.92	680.44	1085.49	#NULL!	319.37	8.61	1.03	7.70

4.71	354.17	1476.72	#NULL!	444.73	11.67	1.69	9.54
6.19	160.77	1989.43	#NULL!	630.50	15.06	2.73	10.20
7.42	227.44	2015.04	#NULL!	607.45	15.22	2.55	12.60
661.95	1342.19	1286.31	397.55	10.25	1.42	10.49	85.12
4.36	632.44	1366.38	#NULL!	378.02	13.60	1.41	9.70
4.85	649.46	1375.82	#NULL!	396.13	10.13	1.48	8.97
4.13	138.69	1204.83	#NULL!	383.47	9.76	2.00	8.99
4.86	151.22	1319.94	#NULL!	414.09	11.06	1.77	9.01
4.61	64.83	1431.61	#NULL!	321.80	10.70	1.15	5.21
7.55	403.18	2088.84	#NULL!	566.39	14.89	1.97	7.05
66.01	1431.53	1250.50	365.26	11.83	1.37	7.18	134.56
169.45	1260.78	1650.31	335.74	9.93	1.65	7.06	117.77
949.47	1061.15	1643.25	359.01	9.18	1.13	10.10	65.57
3.92	960.57	1098.22	#NULL!	382.69	9.54	1.20	10.62
4.78	404.86	1272.40	#NULL!	329.66	10.72	1.40	7.48
12.20	361.36	3207.94	#NULL!	771.16	22.02	2.14	12.26
6.99	648.28	1840.63	#NULL!	573.13	13.71	2.05	8.79
1573.66	1479.30	1531.00	546.40	13.63	1.62	15.55	108.22
6.05	126.35	1660.03	#NULL!	487.94	13.96	2.11	7.36
5.33	219.48	1425.67	#NULL!	403.00	10.60	1.61	6.80
8.17	540.33	2011.33	#NULL!	488.87	13.30	1.82	7.29
685.67	1414.63	#NULL!	483.43	12.08	1.59	14.08	153.13
4.15	94.23	1489.33	#NULL!	402.58	10.88	1.97	8.33
207.60	1299.91	1472.52	325.27	9.64	9.21	5.57	66.16
4.24	545.17	1258.46	#NULL!	411.83	11.19	1.45	10.97
4.91	298.38	970.89	#NULL!	337.51	7.64	1.60	5.93
196.07	1327.25	1585.60	327.76	10.70	1.29	6.57	141.25
6.25	292.99	1793.09	#NULL!	601.43	14.87	2.53	12.03
56.30	1200.28	1687.50	278.61	9.14	1.31	4.93	96.89
4.31	96.26	1406.95	#NULL!	432.11	11.94	1.69	7.26
4.63	159.64	1265.48	#NULL!	309.19	8.55	1.03	4.77
3.79	193.89	1056.99	#NULL!	226.65	8.15	0.93	3.99
4.66	66.26	1359.85	#NULL!	332.02	11.18	1.28	5.43
187.28	1111.62	1135.20	251.93	8.58	3.24	4.70	98.66
4.56	79.13	1335.83	#NULL!	336.68	10.91	1.26	5.50
1586.69	1363.34	1470.31	520.94	12.49	1.43	15.45	106.93
3.93	244.49	1205.51	#NULL!	339.13	8.51	1.29	6.19
4.11	199.64	1068.04	#NULL!	363.44	9.80	1.52	6.79
4.98	407.08	1430.07	#NULL!	491.40	11.37	1.93	8.93
8.58	1080.93	2318.33	#NULL!	746.74	19.26	2.54	15.55
143.50	1585.05	1125.12	385.11	12.58	1.35	6.88	118.54
378.29	1204.15	1601.30	317.67	8.60	9.25	6.03	58.03
4.01	80.62	1036.98	#NULL!	396.43	8.11	1.79	9.48
208.15	1324.76	1700.20	360.36	9.95	5.04	6.85	107.73
5.82	104.00	1598.32	#NULL!	377.16	13.56	1.45	7.36
4.37	365.83	1123.48	#NULL!	292.18	7.76	0.99	5.69
3.79	155.55	1037.24	#NULL!	363.11	9.01	1.50	6.68
4.45	366.29	1240.47	#NULL!	357.09	9.44	1.13	7.09
4.91	249.97	1490.15	#NULL!	386.20	11.10	1.66	6.89
6.10	85.74	1741.71	#NULL!	613.72	15.17	2.57	12.54
5.50	218.04	994.30	#NULL!	344.57	8.23	1.84	5.52
6.90	210.96	1820.70	#NULL!	445.25	12.26	1.48	5.32
65.66	1516.46	998.50	413.09	12.48	1.48	7.91	148.52

3.87	58.24	992.38	#NULL!	300.85	8.89	1.35	5.82
4.87	93.57	1343.56	#NULL!	437.66	12.22	1.64	7.94
7.25	427.60	1910.95	#NULL!	571.71	14.47	2.11	8.33
398.25	1245.64	1502.61	377.32	9.86	1.43	10.03	135.59
5.07	194.40	1426.41	#NULL!	461.16	11.66	1.98	8.83
133.27	1260.23	1520.30	325.05	10.39	1.31	4.45	108.84
5.35	220.10	1755.58	#NULL!	466.48	13.94	1.67	9.08
4.81	218.04	1218.39	#NULL!	401.77	10.33	1.71	8.17
5.17	152.17	1558.62	#NULL!	479.99	12.82	2.12	9.65
108.04	1611.61	1312.54	453.83	13.98	4.50	8.79	159.30
198.68	1574.25	1180.36	422.98	13.63	1.67	6.88	106.01
3.91	200.70	1111.01	#NULL!	251.59	8.89	1.01	4.73
5.41	129.78	1488.02	#NULL!	388.47	10.99	1.60	7.32
5.84	129.06	1599.94	#NULL!	488.09	12.56	2.00	10.22
3.80	663.90	1146.20	#NULL!	391.63	9.29	1.32	9.69
5.95	401.20	1718.51	#NULL!	537.56	13.47	2.08	11.47
3.98	535.21	1366.60	#NULL!	476.06	11.39	1.67	13.79
4.36	632.44	1366.38	#NULL!	378.02	13.60	1.41	9.70
6.67	233.32	1685.44	#NULL!	431.30	11.50	1.41	6.82
4.67	142.17	1523.58	#NULL!	409.63	10.65	1.73	7.28
4.37	365.83	1123.48	#NULL!	292.18	7.76	0.99	5.69
5.61	318.75	1586.78	#NULL!	529.40	12.88	2.10	11.04
4.25	149.45	1082.11	#NULL!	282.63	7.75	0.97	6.46
6.09	374.89	1821.49	#NULL!	532.06	13.95	2.01	9.36
4.85	649.46	1375.82	#NULL!	396.13	10.13	1.48	8.97
4.37	443.06	1540.42	#NULL!	578.54	12.90	1.88	15.90
3.92	1540.66	1453.42	#NULL!	541.21	13.63	1.63	16.11
143.03	1541.82	1260.20	364.81	10.43	1.75	6.65	124.55
6.75	428.07	2027.49	#NULL!	695.98	16.34	2.60	18.95
3.96	126.10	1047.13	#NULL!	285.55	7.97	0.99	5.08
8.94	472.62	2331.21	#NULL!	657.01	17.98	2.91	12.05
3.57	1586.69	1363.34	#NULL!	520.94	12.49	1.43	15.45
4.88	152.32	1280.64	#NULL!	388.50	10.14	1.50	9.34
3.76	680.07	1110.47	#NULL!	362.69	8.98	1.23	9.01
5.98	518.60	1632.40	#NULL!	504.28	12.51	1.90	7.67
5.64	74.72	1633.16	#NULL!	490.33	13.18	1.87	9.17
8.89	1077.69	2571.55	#NULL!	840.33	21.56	2.69	17.73
6.25	178.55	1611.73	#NULL!	540.59	12.95	2.36	11.23
3.69	74.86	1290.20	#NULL!	341.10	9.19	1.51	7.37
6.63	377.37	1650.26	#NULL!	558.92	14.16	2.36	11.64
8.18	270.28	2324.86	#NULL!	708.94	18.33	2.63	11.84
6.17	50.45	1626.35	#NULL!	519.07	12.79	2.40	8.74
6.81	1754.13	2183.43	#NULL!	834.88	12.30	2.95	21.14
6.35	141.58	1509.52	#NULL!	402.69	11.08	1.33	7.05
4.39	98.21	1219.33	#NULL!	305.23	9.18	1.09	4.59
5.66	425.64	1284.12	#NULL!	413.81	9.94	1.79	6.44
4.44	1542.10	1415.90	#NULL!	511.27	13.30	1.54	15.02
961.57	1133.26	1340.25	388.10	9.73	1.22	10.61	73.36
5.16	361.93	1030.27	#NULL!	354.34	8.67	1.46	7.40
5.53	43.53	1679.00	#NULL!	477.61	13.46	1.91	7.99
4.48	1575.31	1709.65	#NULL!	647.08	15.58	1.91	18.69
5.54	412.21	1671.06	#NULL!	530.39	13.26	1.83	13.72
4.78	168.58	1409.15	#NULL!	407.96	10.53	1.92	6.08

4.45	279.21	1249.28	#NULL!	400.05	10.09	1.60	8.77
8.82	108.21	2211.25	#NULL!	461.41	13.72	1.55	6.45
677.97	1151.38	1501.50	376.77	9.46	1.33	9.36	75.89
3.30	128.04	1042.34	#NULL!	298.01	8.99	1.11	3.97
7.18	557.13	2034.59	#NULL!	557.31	15.78	1.78	13.95
6.62	421.24	1734.59	#NULL!	568.83	14.21	2.06	15.84
5.10	194.70	1585.31	#NULL!	402.55	10.36	1.21	8.44
6.51	205.13	1675.76	#NULL!	508.86	14.57	1.93	9.55
5.07	221.28	1397.66	#NULL!	413.61	10.33	1.69	8.35
4.03	57.11	1321.02	#NULL!	331.77	9.57	1.82	7.36
4.59	78.48	1270.47	#NULL!	325.44	10.23	1.34	5.24
4.52	410.48	1237.38	#NULL!	327.66	10.52	1.43	7.21
3.90	959.50	1158.51	#NULL!	385.13	9.92	1.15	10.37
4.88	664.77	1437.18	#NULL!	398.60	10.32	1.40	8.59
7.53	216.36	2218.16	#NULL!	644.71	16.71	2.51	10.81
4.68	139.87	1530.11	#NULL!	395.48	11.59	1.78	7.59
7.15	386.18	2096.11	#NULL!	605.43	15.97	2.34	15.56
4.36	632.44	1366.38	#NULL!	378.02	13.60	1.41	9.70
4.51	365.56	1129.13	#NULL!	286.02	8.03	1.03	6.03
4.59	309.98	1154.20	#NULL!	356.22	8.68	1.45	6.15
374.72	1246.32	1312.50	327.81	9.13	1.31	7.11	69.74
5.69	115.52	1697.21	#NULL!	517.23	14.93	1.89	11.02
6.48	308.07	1658.32	#NULL!	513.38	12.37	2.02	8.77
4.90	408.64	1375.57	#NULL!	450.57	10.89	1.87	8.56
3.69	74.86	1290.20	#NULL!	341.10	9.19	1.51	7.37
7.19	304.15	1986.11	#NULL!	574.51	15.34	2.13	10.55
4.38	406.69	1304.62	#NULL!	335.80	10.68	1.44	7.48
5.40	376.79	1682.86	#NULL!	511.00	12.43	1.97	10.08
3.86	165.38	964.93	#NULL!	359.51	9.03	1.46	6.21
4.64	119.62	1224.92	#NULL!	310.77	8.50	1.18	5.74
7.64	157.54	2174.61	#NULL!	686.29	17.17	2.70	12.65

selenium	fluoride	chromium	t.fiber	s.fiber	i.fiber	c.fiber	t.sogar
111.56	4414.87	0.17	37.92	0.51	2.14	8.92	96.83
91.90	1850.11	0.06	59.60	0.48	3.23	20.80	99.02
118.43	4343.78	0.05	74.10	0.11	0.80	5.77	58.01
51.56	2720.49	0.03	30.60	0.35	2.26	9.13	89.01
108.64	2677.43	0.16	20.35	0.06	0.34	6.27	82.58
143.84	1243.95	0.29	50.42	0.95	6.31	21.61	173.31
65.97	2849.32	0.01	37.42	0.38	2.15	11.36	85.68
126.55	1886.26	0.22	27.59	0.48	2.27	20.26	133.51
2740.65	0.03	35.21	0.38	2.33	10.28	95.05	17.76
114.14	233.31	0.17	49.60	0.31	2.20	17.02	102.14
1949.22	0.15	31.68	0.35	2.19	11.08	147.77	31.29
167.06	3761.58	0.26	46.08	0.76	4.23	17.42	180.99
1821.84	0.11	29.81	0.15	0.74	9.54	69.15	8.85
126.38	946.26	0.25	30.58	0.25	1.05	8.42	80.94
81.97	4362.70	0.10	28.55	0.23	1.49	8.91	77.74
65.16	2740.62	0.03	37.60	0.35	2.05	10.47	93.95
113.64	1051.97	0.05	36.06	0.23	1.30	6.86	77.04
95.57	2351.63	0.14	24.50	0.31	1.96	11.96	88.22
90.74	4325.26	0.13	46.25	0.18	0.95	8.46	90.74
117.98	1825.71	0.08	46.20	0.32	1.67	5.93	78.10
127.39	278.18	0.22	30.05	0.13	1.04	6.88	105.52
96.86	2034.41	0.05	24.62	0.29	1.33	8.31	128.09
111.05	2744.25	0.21	38.78	0.61	3.45	10.09	121.27
137.85	324.23	0.22	45.72	0.81	3.76	10.86	129.65
91.90	1850.11	0.06	59.60	0.48	3.23	20.80	99.02
125.84	1878.80	0.12	25.92	0.49	2.16	8.25	135.42
120.03	2817.61	0.18	43.78	0.61	2.82	17.14	140.24
134.22	2800.66	0.23	51.95	0.60	4.99	18.96	140.60
119.59	1846.86	0.08	23.77	0.22	1.11	5.66	78.35
74.60	1926.55	0.02	16.90	0.61	2.92	5.32	98.89
81.81	2041.49	0.03	18.23	0.59	0.00	0.00	0.00
113.38	1927.10	0.07	27.67	0.57	2.15	8.07	127.47
59.06	2839.47	0.01	41.38	0.38	2.15	11.75	91.40
143.72	2814.99	0.26	44.03	0.79	4.64	13.66	122.75
1882.88	0.18	31.89	0.46	2.38	9.80	97.36	12.53
1860.21	0.09	34.47	0.21	1.48	12.09	84.79	16.01
87.92	2739.02	0.08	50.37	0.79	3.60	8.95	84.54
116.81	2794.29	0.19	35.36	0.64	2.95	9.54	129.74
66.31	4391.21	0.07	26.68	0.17	1.56	8.12	48.35
88.81	2138.73	0.01	50.96	0.15	0.68	7.87	45.44
80.99	4365.04	0.00	43.75	0.18	1.54	7.52	49.31
96.89	1931.13	0.03	27.87	0.57	2.42	11.07	117.41
110.03	1987.52	0.08	34.60	0.40	2.05	7.49	112.21
100.67	4467.30	0.10	42.42	0.16	0.97	10.28	84.38
94.81	1918.05	0.13	45.70	0.90	3.92	10.42	131.53
143.96	3433.54	0.33	29.11	0.13	0.81	6.95	93.57
80.39	1133.52	0.07	53.55	0.40	2.83	14.67	98.31
89.15	0.02	0.00	0.49	2.54	7.39	111.60	20.84
65.10	1055.40	0.00	77.93	0.54	3.29	7.29	92.69
4469.27	0.19	38.65	1.12	4.49	14.23	202.90	25.43
163.57	1233.66	0.15	64.43	0.37	2.18	18.42	139.14
54.50	2708.03	0.01	26.60	0.31	1.85	8.42	86.24

153.03	1884.66	0.29	40.57	0.07	0.27	6.73	99.01
124.35	241.18	0.20	46.83	0.12	1.23	9.67	89.65
96.78	1157.57	0.10	50.20	0.23	1.55	13.30	61.66
1842.78	0.06	26.88	0.25	1.67	9.88	73.15	12.20
4395.74	0.10	32.31	0.15	0.94	10.40	75.51	12.99
180.52	1242.23	0.30	61.23	1.17	5.59	19.13	212.74
2720.49	0.03	30.60	0.35	2.26	9.13	89.01	16.91
222.77	4368.83	0.45	47.24	0.20	1.45	12.65	122.82
88.36	1786.98	0.03	19.63	0.23	1.32	3.81	68.27
1925.17	0.08	23.09	0.40	1.67	7.80	131.78	20.23
1873.17	0.06	37.24	0.19	1.38	12.92	74.29	14.12
56.80	1842.78	0.06	26.88	0.25	1.67	9.88	73.15
146.43	4320.92	0.17	75.85	0.24	1.60	11.46	106.21
161.26	2059.23	0.19	79.44	1.10	6.75	26.02	167.49
85.32	2674.80	0.07	45.84	0.21	1.12	5.88	53.77
1821.84	0.11	29.81	0.15	0.74	9.54	69.15	8.85
1915.15	0.03	53.78	0.24	1.84	18.58	81.44	14.47
85.14	1030.36	0.09	33.15	0.51	2.22	6.88	79.43
88.81	2138.73	0.01	50.96	0.15	0.68	7.87	45.44
89.06	3619.80	0.06	33.53	0.70	2.86	9.62	175.68
1154.76	0.07	58.13	1.81	8.37	16.70	194.69	35.61
1940.79	0.06	32.81	0.47	2.29	9.22	95.92	11.96
91.63	1030.61	0.15	24.08	0.32	2.27	7.15	74.47
101.80	1883.94	0.11	53.43	0.53	2.27	8.30	100.25
1909.40	0.31	43.39	0.58	2.81	11.45	176.22	30.18
188.15	297.80	0.25	68.51	0.19	1.36	11.42	119.81
133.34	3524.47	0.26	30.96	0.30	1.28	8.51	114.09
152.35	2799.91	0.20	62.21	0.10	0.72	9.61	128.24
4352.40	0.05	49.64	0.17	1.32	10.20	68.40	12.18
173.43	1980.03	0.22	88.98	0.87	4.99	27.30	168.22
2016.56	0.25	65.99	0.94	5.42	19.25	176.05	25.02
99.02	1926.78	0.06	21.86	0.54	2.08	6.97	112.52
116.20	2187.85	0.12	31.62	0.39	1.73	9.30	116.76
194.74	601.83	0.24	47.25	0.94	5.28	15.45	133.19
2705.65	0.10	45.35	0.53	3.40	13.41	112.97	19.28
195.33	4357.09	0.38	36.20	0.32	1.30	8.47	127.49
94.68	2610.70	0.13	25.54	0.98	3.66	7.62	106.20
130.77	3795.36	0.18	47.04	0.61	2.72	10.14	119.17
57.64	4417.14	0.02	18.47	0.33	1.83	8.57	61.43
85.24	2033.07	0.02	18.51	0.53	2.38	6.08	99.93
2860.36	0.14	23.66	0.42	2.19	7.15	95.71	15.92
2688.13	0.01	33.96	0.53	3.26	11.58	92.70	16.84
125.34	1003.22	0.15	42.64	0.33	1.58	12.38	79.09
97.73	1046.21	0.06	22.68	0.24	1.21	7.00	109.70
63.73	1947.47	0.03	33.48	0.36	2.24	10.16	79.84
141.87	2731.81	0.26	53.37	0.56	3.25	11.63	134.19
99.66	209.49	0.10	23.62	0.11	1.11	6.98	59.73
95.91	1044.97	0.11	32.65	0.98	3.56	9.36	136.72
119.59	1981.26	0.13	36.95	0.52	2.12	9.15	136.16
139.35	4399.75	0.17	37.48	0.87	4.10	11.97	120.85
84.85	2621.89	0.05	48.48	0.38	1.90	4.34	81.79
89.66	2705.65	0.10	45.35	0.53	3.40	13.41	112.97
1469.11	0.03	35.00	0.24	1.38	6.59	69.16	8.84

4407.67	0.04	73.14	0.12	0.82	10.23	83.50	13.70
134.42	0.07	44.64	0.16	1.31	11.23	60.96	10.16
80.35	1877.25	0.06	32.99	0.43	2.09	9.09	89.72
139.80	1204.78	0.26	42.76	0.68	2.86	15.65	149.72
167.89	3628.94	0.30	47.27	0.42	2.53	13.63	123.35
225.40	0.16	26.07	0.12	1.15	7.59	71.70	9.56
112.03	970.17	0.17	37.21	0.12	0.97	6.58	73.83
90.71	1904.42	0.14	27.54	1.00	4.20	8.26	120.50
67.63	2712.56	0.06	23.55	0.66	2.83	12.26	72.89
141.53	1967.68	0.17	55.76	1.70	6.66	15.70	173.42
133.88	4494.97	0.10	60.54	0.44	3.00	13.31	95.31
101.53	4363.98	0.08	53.13	0.15	1.16	8.81	74.01
153.03	2978.94	0.19	73.32	1.44	6.38	23.85	201.71
4444.12	0.12	74.70	0.17	1.42	11.70	91.15	13.64
143.96	2706.09	0.30	31.20	0.12	1.11	11.38	87.67
157.48	2835.09	0.18	63.26	0.52	3.32	16.46	150.68
74.81	4330.86	0.08	26.67	0.14	1.12	6.91	51.24
70.38	4365.15	0.10	26.15	0.49	2.72	13.08	76.91
111.90	1959.78	0.18	33.44	0.58	3.28	12.45	127.35
4337.06	0.19	35.02	0.14	1.14	8.49	74.61	11.18
89.76	1921.33	0.05	28.26	0.50	2.75	9.19	99.00
148.37	2767.55	0.23	51.18	1.01	4.60	19.35	163.85
156.81	4423.08	0.29	47.08	0.47	2.07	14.78	164.91
86.69	2008.29	0.03	18.92	0.55	2.46	7.81	120.64
130.87	1014.27	0.23	33.94	0.20	1.41	14.77	102.14
181.04	2811.33	0.29	40.19	0.51	2.57	14.25	136.74
97.32	2695.79	0.06	65.62	0.53	3.40	21.62	109.39
130.03	3615.68	0.18	33.15	0.40	2.39	13.69	100.08
77.10	1998.59	0.07	28.45	0.53	3.14	9.60	98.07
160.51	1886.27	0.24	51.67	0.29	1.60	9.44	151.52
119.84	2753.14	0.19	40.98	0.95	4.89	20.06	177.70
134.42	0.07	44.64	0.16	1.31	11.23	60.96	10.16
78.25	1085.87	0.05	23.25	0.28	1.16	7.89	75.45
111.62	1821.84	0.11	29.81	0.15	0.74	9.54	69.15
89.89	3682.10	0.08	40.34	0.77	4.39	17.36	137.40
104.90	1931.03	0.05	21.50	0.62	2.40	5.85	119.99
132.74	1981.96	0.17	52.64	1.02	5.19	16.76	151.70
100.02	1925.17	0.03	26.56	0.47	1.87	8.67	141.45
139.65	4406.15	0.24	38.86	0.63	2.42	13.07	120.10
81.13	3573.30	0.06	29.08	0.60	2.66	9.17	92.53
198.13	4522.09	0.29	79.75	0.94	6.15	28.07	169.43
98.15	1964.61	0.06	21.92	0.58	2.82	8.70	137.63
98.35	3697.96	0.10	42.13	0.88	5.38	10.72	132.19
112.42	6919.59	0.07	47.97	0.89	3.38	14.82	130.57
78.41	1853.50	0.12	19.40	0.20	1.28	5.64	55.60
105.45	994.52	0.18	35.41	0.44	1.83	11.28	106.49
4339.97	0.15	31.99	0.19	1.26	10.29	64.48	9.86
157.02	1170.30	0.18	46.73	0.75	5.27	19.48	164.43
89.64	5258.21	0.12	28.66	0.58	2.58	8.54	124.86
150.58	3531.77	0.29	36.77	0.28	1.08	10.08	102.86
107.06	306.62	0.04	71.49	0.15	1.22	7.97	87.56
67.60	1839.94	0.09	29.23	0.23	1.60	10.24	82.67
59.23	1874.96	0.03	29.52	0.19	1.39	10.74	74.83

138.28	2654.63	0.26	28.53	0.36	1.41	9.74	109.41
145.47	1128.19	0.23	60.77	0.61	3.29	13.00	137.07
180.25	4486.37	0.27	73.37	0.95	4.63	14.27	158.65
4352.40	0.05	49.64	0.17	1.32	10.20	68.40	12.18
111.62	1821.84	0.11	29.81	0.15	0.74	9.54	69.15
73.01	2796.14	0.01	44.56	0.37	2.14	12.06	89.28
135.10	2661.99	0.19	55.52	0.13	0.63	10.05	81.10
119.58	3879.72	0.18	29.72	0.11	0.91	11.82	98.33
104.71	1924.82	0.09	20.87	0.45	1.92	7.41	128.80
123.73	1141.98	0.17	45.37	0.65	3.10	17.10	161.97
1903.61	0.17	26.34	0.51	2.42	8.77	137.77	22.99
4388.30	0.08	46.68	0.27	1.61	7.05	91.75	11.15
1883.70	0.03	34.97	0.22	1.57	11.81	68.03	12.47
74.17	1867.94	0.06	36.75	0.20	1.47	12.69	73.40
121.77	1469.29	0.05	36.12	0.24	1.33	6.88	71.98
212.94	3931.27	0.36	51.53	0.88	5.63	15.14	222.65
125.76	2084.97	0.19	42.29	0.69	3.81	16.37	142.70
1853.76	0.06	58.61	0.29	1.69	18.42	76.91	12.15
119.24	2793.98	0.13	36.09	0.16	1.50	14.60	82.00
124.35	2788.33	0.11	31.14	0.76	3.42	17.29	107.51
119.81	2982.57	0.11	46.33	1.06	6.05	10.19	143.02
4362.50	0.22	39.31	0.12	0.85	12.18	100.92	15.00
101.10	4391.21	0.10	62.63	0.17	1.56	10.75	67.91
1947.47	0.04	31.27	0.36	2.24	10.19	81.90	11.75
141.01	4381.98	0.11	32.78	0.47	2.79	9.67	79.00
65.03	2798.44	0.07	33.33	0.76	3.90	22.32	107.17
1846.90	0.12	23.92	0.23	1.25	6.20	85.45	8.89
182.62	2775.06	0.31	59.91	0.61	2.85	15.19	148.17
219.65	0.10	34.38	0.17	1.49	7.89	66.49	9.54
111.55	975.46	0.22	38.14	0.34	1.34	10.72	98.65
94.62	202.58	0.14	22.54	0.48	1.76	4.13	86.15
85.16	1846.41	0.01	17.87	0.23	1.22	4.20	63.43
99.68	2039.66	0.09	24.72	0.51	2.45	8.20	133.74
1875.44	0.04	19.81	0.29	1.53	4.66	68.96	7.81
112.48	1948.94	0.09	24.83	0.58	2.76	8.55	141.25
1823.18	0.06	51.70	0.27	1.85	17.13	72.48	11.57
82.44	2708.39	0.12	28.41	0.39	3.46	13.46	100.70
100.44	1024.88	0.20	34.86	0.48	2.61	12.23	93.08
119.65	2789.00	0.21	41.15	0.67	2.95	12.14	126.52
169.71	2047.42	0.22	69.80	1.04	5.56	23.24	179.00
2001.44	0.13	28.07	0.48	1.92	8.63	139.82	27.67
2766.85	0.01	33.53	0.52	3.19	11.51	100.24	18.61
108.67	5221.36	0.18	40.38	0.54	2.43	14.36	121.85
1977.24	0.07	54.22	0.50	2.17	11.62	116.33	18.68
134.93	2865.11	0.12	25.54	0.45	2.02	6.73	99.44
51.66	2726.18	0.01	30.22	0.34	1.99	8.60	97.76
108.78	537.97	0.22	32.68	0.36	2.01	9.17	94.95
81.13	3573.30	0.06	29.08	0.60	2.66	9.17	92.53
120.63	1853.38	0.08	39.16	0.23	1.18	9.09	118.20
192.99	1044.98	0.41	53.74	0.44	2.20	17.97	162.89
78.34	1052.03	0.06	57.39	1.11	4.83	15.92	114.44
60.83	3762.58	0.01	46.97	1.10	4.79	13.35	156.24
1911.21	0.22	32.81	0.56	2.78	10.09	156.56	27.71

99.64	2700.71	0.08	29.56	0.10	0.56	5.53	60.64
132.93	682.03	0.26	33.71	0.45	1.86	11.59	126.17
120.67	2854.24	0.15	44.14	1.02	4.91	21.65	165.63
4416.33	0.11	35.79	0.21	1.45	8.47	76.76	12.69
135.77	238.41	0.25	43.22	0.35	1.74	10.53	108.20
1949.74	0.02	32.59	0.45	2.11	5.95	104.75	17.86
147.53	2054.04	0.20	37.53	0.32	2.01	12.51	150.88
106.46	2727.83	0.17	32.66	0.57	3.15	20.79	93.19
149.01	1029.44	0.26	38.15	0.30	1.50	15.22	101.79
2008.98	0.20	38.22	0.50	2.77	11.56	159.04	33.52
3580.86	0.06	33.76	0.70	2.86	9.58	172.70	34.96
89.35	1839.00	0.04	17.34	0.22	1.12	4.73	62.21
113.07	2733.76	0.15	41.22	0.47	2.34	11.53	101.24
164.12	1035.64	0.32	39.53	0.39	1.77	12.22	127.07
83.15	1847.87	0.11	37.56	0.30	1.97	12.71	89.91
176.90	2758.28	0.30	46.62	0.29	1.56	10.40	116.23
162.06	4326.09	0.28	62.19	0.16	1.24	11.58	90.89
111.62	1821.84	0.11	29.81	0.15	0.74	9.54	69.15
98.35	3697.96	0.10	42.13	0.88	5.38	10.72	132.19
122.44	1875.73	0.14	40.01	0.57	3.10	14.45	112.43
51.66	2726.18	0.01	30.22	0.34	1.99	8.60	97.76
147.58	4384.26	0.26	42.49	0.48	1.99	10.90	116.23
84.16	3599.44	0.09	23.15	0.18	0.97	5.66	115.35
138.59	1892.87	0.22	37.72	0.41	3.26	9.34	114.97
73.01	2796.14	0.01	44.56	0.37	2.14	12.06	89.28
192.29	4331.99	0.45	46.76	0.23	1.79	13.33	129.84
102.53	1834.08	0.08	56.80	0.21	1.55	17.43	74.22
1932.22	0.08	78.61	0.33	1.55	10.84	127.95	20.60
259.67	4422.64	0.51	79.10	0.24	1.84	15.03	159.18
74.69	1968.93	0.07	23.64	0.38	1.93	9.76	87.64
189.27	2752.34	0.29	44.05	0.48	3.52	24.10	132.01
106.93	1823.18	0.06	51.70	0.27	1.85	17.13	72.48
124.01	4416.70	0.19	38.56	0.17	1.46	7.70	84.48
71.46	1860.21	0.09	34.47	0.21	1.48	12.09	84.79
116.38	1086.28	0.21	39.73	0.80	3.58	17.03	142.06
172.88	2028.12	0.23	41.74	0.65	3.06	11.67	176.00
191.07	2093.88	0.28	79.37	1.06	5.63	24.12	193.06
169.75	1886.44	0.32	46.34	0.88	4.29	15.42	171.95
85.11	4368.04	0.10	41.98	0.18	1.40	6.98	65.63
165.16	2748.98	0.25	40.98	0.59	2.60	11.99	128.71
190.32	1204.75	0.39	58.21	0.73	4.63	24.87	191.14
123.10	1095.45	0.19	53.08	1.19	4.03	17.92	163.95
168.02	1938.46	0.19	81.14	0.73	3.63	26.68	154.10
98.26	3298.78	0.13	26.71	0.88	3.21	8.96	129.89
91.24	1966.14	0.04	21.64	0.61	2.84	7.68	106.47
92.85	1869.98	0.13	37.33	0.75	3.60	11.55	114.58
86.04	1915.15	0.03	53.78	0.24	1.84	18.58	81.44
1873.17	0.06	37.24	0.19	1.38	12.92	74.29	14.12
82.62	2799.46	0.10	30.61	0.87	5.25	18.89	125.36
112.91	1855.80	0.19	33.26	0.39	2.22	20.62	124.45
159.96	1843.23	0.20	63.10	0.28	1.88	19.06	102.15
174.48	4416.95	0.28	40.84	0.27	1.76	11.26	111.14
87.78	1111.71	0.03	47.99	0.39	1.95	5.38	71.14

120.28	2742.68	0.20	30.84	0.20	1.31	6.64	73.18
135.33	1293.58	0.15	47.47	1.19	7.02	21.19	167.90
1851.74	0.09	35.88	0.24	1.64	12.80	84.52	15.69
86.73	1968.26	0.02	32.46	0.40	2.00	5.89	100.28
161.67	4398.64	0.21	37.38	0.14	1.19	11.62	115.44
201.17	4421.72	0.34	42.10	0.23	1.81	12.21	120.39
89.64	5258.21	0.12	28.66	0.58	2.58	8.54	124.86
135.97	2900.56	0.22	37.94	0.36	2.81	11.96	115.29
118.69	1850.77	0.24	33.11	0.31	2.56	14.78	106.51
118.43	4343.78	0.14	74.10	0.11	0.80	5.77	58.01
92.31	1977.98	0.04	24.85	0.54	2.54	8.06	130.98
121.55	1050.22	0.05	41.81	0.33	1.66	7.16	86.54
74.74	1959.16	0.06	38.28	0.25	1.85	12.86	80.75
66.05	2876.48	0.01	43.15	0.35	2.05	11.39	94.86
188.33	1164.89	0.29	58.79	1.00	5.51	15.55	196.45
143.20	961.86	0.21	52.19	0.24	1.03	6.41	82.64
205.19	4457.91	0.30	71.32	0.15	0.89	12.92	132.96
111.62	1821.84	0.11	29.81	0.15	0.74	9.54	69.15
54.50	2708.03	0.01	26.60	0.31	1.85	8.42	86.24
94.60	1075.74	0.15	31.83	0.69	3.69	14.86	118.57
2740.65	0.03	35.21	0.38	2.33	10.28	95.05	17.76
186.92	2052.11	0.34	44.00	0.37	2.29	14.01	189.34
146.15	1170.77	0.21	40.45	0.82	4.70	21.18	139.22
115.84	2772.26	0.17	38.85	0.31	2.14	10.81	100.82
85.11	4368.04	0.10	41.98	0.18	1.40	6.98	65.63
159.26	1971.10	0.29	40.95	0.36	1.84	17.65	145.40
121.86	1861.82	0.05	48.84	0.23	1.34	6.95	76.42
139.57	2743.03	0.26	44.95	0.26	1.64	12.50	113.33
78.67	2731.11	0.12	26.82	0.67	2.69	6.07	101.87
77.02	2675.38	0.09	24.72	0.62	2.47	5.95	88.38
164.87	3605.17	0.29	57.46	1.01	6.79	20.34	167.92

glucose	galactos	fructose	sucrose	lactose	maltose	zinc2	selenium2
13.49	1.77	17.40	14.36	10.03	1.38	1.00	1.00
19.57	2.08	19.11	24.93	8.41	0.73	1.00	1.00
11.64	2.18	13.98	7.24	7.55	3.08	0.00	1.00
16.91	2.61	20.90	18.93	10.94	0.70	0.00	1.00
10.17	2.31	11.31	20.30	7.03	0.95	1.00	1.00
22.69	4.68	32.11	29.99	23.89	1.48	1.00	1.00
16.61	2.15	20.29	21.03	10.49	0.61	1.00	1.00
15.36	7.26	19.30	10.61	30.45	1.13	1.00	1.00
2.35	21.88	20.78	11.11	0.74	#NULL!	0.00	1.00
13.42	3.33	15.13	7.85	25.91	2.06	0.00	1.00
1.75	36.24	20.39	16.90	4.42	#NULL!	0.00	1.00
19.64	4.89	25.57	26.25	37.91	1.75	1.00	1.00
1.17	9.04	11.05	10.04	0.86	#NULL!	0.00	1.00
10.76	0.24	12.55	6.76	0.99	1.37	1.00	1.00
11.31	3.56	13.93	13.34	8.97	0.76	1.00	1.00
16.43	3.50	20.31	19.44	15.87	0.86	0.00	1.00
9.10	2.37	10.24	16.40	12.95	1.56	1.00	1.00
14.12	1.55	18.76	7.95	16.28	0.76	1.00	1.00
11.39	1.66	13.78	21.33	8.81	2.04	0.00	1.00
10.01	1.97	11.90	15.50	8.28	2.14	0.00	1.00
9.30	3.65	12.08	15.09	21.21	1.27	1.00	1.00
27.33	2.66	30.28	21.07	11.30	2.19	1.00	1.00
16.49	1.77	20.62	27.01	4.55	1.34	1.00	1.00
18.81	2.39	24.92	29.44	6.01	1.67	1.00	1.00
19.57	2.08	19.11	24.93	8.41	0.73	1.00	1.00
22.81	1.33	25.78	31.04	14.58	2.37	1.00	1.00
21.22	3.82	24.22	14.98	21.53	1.45	1.00	1.00
20.60	4.54	20.49	24.08	12.06	1.33	1.00	1.00
8.48	1.97	10.08	16.11	12.63	0.92	1.00	1.00
18.30	2.23	20.69	20.37	11.63	2.62	1.00	1.00
0.00	0.00	0.00	0.00	0.00	0.00	1.00	1.00
25.07	1.43	28.19	27.47	12.66	2.10	1.00	1.00
16.78	3.54	20.42	21.67	12.36	0.70	0.00	1.00
21.22	0.64	23.24	18.18	1.79	1.57	1.00	1.00
2.68	16.62	13.50	6.75	1.00	#NULL!	0.00	1.00
2.24	17.15	15.92	8.16	0.72	#NULL!	0.00	1.00
8.85	1.00	11.94	26.40	9.32	1.73	1.00	1.00
16.61	3.43	21.60	19.23	15.76	1.36	1.00	1.00
8.30	1.27	9.97	7.40	4.60	0.71	0.00	1.00
5.75	1.00	5.48	21.35	3.85	1.42	0.00	1.00
10.98	1.78	11.91	9.04	5.39	1.42	0.00	1.00
24.30	1.13	27.61	29.83	12.08	2.87	1.00	1.00
14.80	3.89	16.75	24.96	20.04	2.80	1.00	1.00
11.60	1.82	14.53	19.45	6.27	1.55	0.00	1.00
19.70	3.41	22.16	37.05	9.01	1.50	1.00	1.00
9.35	0.27	10.98	12.83	0.88	1.62	0.00	1.00
18.40	1.98	22.76	15.70	5.89	1.94	1.00	1.00
1.29	23.26	29.31	11.89	1.90	#NULL!	0.00	1.00
19.41	3.30	24.02	18.97	8.51	2.85	0.00	1.00
3.05	28.97	66.82	20.50	1.68	#NULL!	0.00	1.00
18.50	4.55	23.95	21.02	36.51	2.51	1.00	1.00
16.33	3.42	20.20	17.70	13.09	0.52	0.00	1.00

11.14	1.92	13.19	11.25	10.45	1.89	1.00	1.00
12.60	0.87	15.88	16.06	2.87	2.17	1.00	1.00
13.03	0.67	15.78	8.06	1.73	1.86	1.00	1.00
2.26	13.11	14.74	9.80	0.58	#NULL!	0.00	1.00
0.26	14.82	11.30	6.42	0.60	#NULL!	0.00	1.00
25.65	6.74	31.46	32.98	40.99	2.32	1.00	1.00
2.61	20.90	18.93	10.94	0.70	#NULL!	0.00	1.00
15.46	0.45	19.04	8.28	2.73	2.20	1.00	1.00
6.92	0.17	7.60	37.63	2.32	1.13	1.00	1.00
2.74	22.22	17.93	19.80	1.47	#NULL!	0.00	1.00
1.78	14.23	15.67	8.92	0.53	#NULL!	0.00	1.00
12.20	2.26	13.11	14.74	9.80	0.58	0.00	1.00
9.58	5.71	10.92	18.44	19.21	2.70	1.00	1.00
26.19	2.29	29.12	40.37	15.58	1.33	1.00	1.00
9.33	1.04	11.96	7.41	4.64	1.81	0.00	1.00
1.17	9.04	11.05	10.04	0.86	#NULL!	0.00	1.00
1.83	14.39	20.03	11.75	0.47	#NULL!	0.00	1.00
13.65	0.97	16.31	19.07	2.30	1.47	1.00	1.00
5.75	1.00	5.48	21.35	3.85	1.42	0.00	1.00
35.01	2.31	39.63	46.12	13.02	2.54	1.00	1.00
2.00	39.48	57.84	6.05	1.72	#NULL!	0.00	1.00
2.03	15.29	19.04	16.26	0.97	#NULL!	0.00	1.00
9.76	0.27	13.98	19.52	0.59	0.77	0.00	1.00
14.45	2.23	17.59	18.05	11.03	2.08	1.00	1.00
0.73	34.34	30.72	11.95	3.79	#NULL!	0.00	1.00
15.91	3.58	20.00	18.19	10.68	2.95	1.00	1.00
13.30	1.71	15.71	18.47	5.13	1.28	0.00	1.00
16.53	1.57	26.05	29.94	7.71	2.48	1.00	1.00
2.61	12.99	13.99	10.58	1.24	#NULL!	0.00	1.00
30.08	0.59	32.57	36.30	7.02	1.64	1.00	1.00
2.83	30.34	41.75	15.30	1.97	#NULL!	0.00	1.00
18.88	1.43	21.31	25.43	12.96	2.13	1.00	1.00
18.72	1.97	22.38	23.68	9.29	2.53	1.00	1.00
22.51	0.66	26.08	23.18	2.09	1.81	1.00	1.00
2.79	22.04	24.15	11.95	1.19	#NULL!	0.00	1.00
12.02	3.26	14.17	8.13	14.56	1.96	1.00	1.00
8.94	3.80	10.17	34.33	15.12	0.84	0.00	1.00
19.18	1.99	25.17	20.42	5.95	1.74	1.00	1.00
8.90	1.36	11.73	16.26	10.11	0.45	0.00	1.00
16.58	2.20	19.21	27.13	17.31	1.86	1.00	1.00
1.28	17.77	12.76	8.48	2.44	#NULL!	0.00	1.00
2.79	19.19	22.19	11.96	0.77	#NULL!	0.00	1.00
10.53	0.67	11.36	15.03	5.38	1.13	1.00	1.00
13.39	3.55	14.65	31.98	20.86	0.99	0.00	1.00
11.56	0.64	14.81	15.27	11.87	0.56	1.00	1.00
16.30	1.66	19.82	26.81	11.78	2.08	1.00	1.00
8.80	0.44	10.49	14.00	0.94	0.94	0.00	1.00
17.92	1.67	23.21	36.31	7.05	0.98	0.00	1.00
20.73	2.66	24.17	30.13	11.01	2.83	0.00	1.00
10.66	4.08	11.66	29.31	22.40	0.83	1.00	1.00
12.70	1.03	15.38	20.38	5.54	1.85	0.00	1.00
19.28	2.79	22.04	24.15	11.95	1.19	1.00	1.00
2.37	9.85	13.46	12.08	1.48	#NULL!	0.00	1.00

3.90	17.20	12.79	16.86	2.46	#NULL!	0.00	1.00
1.19	9.44	11.81	5.55	1.21	#NULL!	0.00	1.00
11.86	2.03	15.20	18.90	10.28	0.89	0.00	1.00
17.76	3.32	23.92	18.47	20.47	1.45	1.00	1.00
14.32	3.65	16.89	13.31	14.65	1.78	1.00	1.00
0.55	11.63	13.26	2.23	1.17	#NULL!	0.00	1.00
7.97	0.54	8.96	23.40	3.04	0.82	1.00	1.00
20.60	3.34	25.52	24.66	10.30	0.80	1.00	1.00
11.96	0.70	16.57	19.74	1.26	0.71	0.00	1.00
30.53	3.56	40.62	37.58	10.71	1.88	1.00	1.00
14.05	1.27	17.99	19.41	4.60	2.82	1.00	1.00
11.34	3.56	12.72	13.60	11.55	1.76	1.00	1.00
26.37	4.63	31.51	46.08	24.55	2.14	1.00	1.00
0.20	15.18	14.56	18.80	2.50	#NULL!	0.00	1.00
10.80	1.76	12.51	3.30	4.53	1.42	1.00	1.00
30.80	2.66	35.65	20.07	11.97	2.46	0.00	1.00
9.38	0.15	10.63	14.04	0.54	0.49	0.00	1.00
12.03	1.55	13.78	15.93	6.29	0.81	1.00	1.00
15.04	2.91	19.95	20.24	20.28	1.18	1.00	1.00
0.15	12.87	14.19	0.00	0.94	#NULL!	0.00	1.00
12.34	2.67	15.72	20.30	17.54	0.73	1.00	1.00
30.18	3.61	34.98	32.38	10.84	1.47	1.00	1.00
22.85	4.72	28.56	29.18	17.50	1.70	1.00	1.00
20.63	1.74	22.80	31.88	13.30	2.10	0.00	1.00
14.84	0.83	19.44	9.74	1.70	1.49	1.00	1.00
18.43	3.82	23.68	13.79	22.07	1.43	1.00	1.00
21.04	2.79	21.51	27.73	10.24	0.86	1.00	1.00
14.77	1.77	19.91	14.35	10.57	1.26	1.00	0.00
17.53	2.07	22.02	21.08	11.95	0.68	1.00	1.00
27.86	1.26	31.80	19.83	13.38	3.89	1.00	1.00
25.08	6.24	28.74	39.63	26.98	1.46	1.00	0.00
1.19	9.44	11.81	5.55	1.21	#NULL!	0.00	1.00
10.49	2.49	12.66	6.89	19.22	0.80	1.00	1.00
8.85	1.17	9.04	11.05	10.04	0.86	1.00	1.00
22.50	5.60	26.95	25.84	26.97	1.42	1.00	1.00
18.13	2.74	21.08	19.95	19.80	1.48	1.00	1.00
21.58	2.62	27.68	33.40	17.49	1.55	1.00	1.00
26.23	2.72	29.09	23.98	19.80	1.71	1.00	1.00
18.58	1.72	20.56	15.59	6.22	1.54	1.00	1.00
14.78	2.59	18.11	19.40	14.02	0.95	1.00	1.00
30.18	0.47	35.55	18.93	12.87	3.28	1.00	1.00
26.90	1.28	29.39	33.83	12.28	3.53	1.00	1.00
19.70	4.31	22.99	23.11	28.79	1.49	1.00	0.00
16.45	4.98	20.27	44.73	16.12	0.90	1.00	1.00
8.47	1.58	11.28	5.26	4.14	0.72	0.00	1.00
12.39	1.69	15.88	25.55	6.09	1.15	0.00	1.00
0.71	12.18	7.99	3.38	1.25	#NULL!	0.00	1.00
23.53	4.24	28.92	22.66	19.94	1.25	1.00	1.00
16.63	5.42	22.18	26.12	20.71	0.92	0.00	1.00
12.80	3.10	14.68	7.94	8.13	1.41	1.00	1.00
11.79	3.65	16.33	15.94	21.21	2.99	1.00	1.00
14.50	2.27	15.71	14.57	9.87	0.65	1.00	1.00
15.42	2.01	16.10	15.01	9.53	0.46	1.00	1.00

12.61	2.24	14.56	18.23	7.45	1.28	1.00	1.00
23.20	3.36	27.59	23.24	8.61	1.48	1.00	1.00
18.14	3.41	21.85	27.25	16.99	2.96	1.00	1.00
2.61	12.99	13.99	10.58	1.24	#NULL!	0.00	1.00
8.85	1.17	9.04	11.05	10.04	0.86	1.00	1.00
16.69	2.16	21.09	20.12	12.16	0.94	0.00	1.00
11.02	0.08	12.43	13.47	0.87	2.33	1.00	1.00
10.39	1.28	11.73	25.78	3.62	1.15	1.00	1.00
21.43	2.51	24.05	29.10	18.30	2.18	1.00	1.00
24.84	4.82	32.16	26.59	24.09	1.30	1.00	1.00
0.83	25.99	25.82	13.26	2.51	#NULL!	0.00	1.00
1.26	13.34	24.77	8.57	1.83	#NULL!	0.00	1.00
1.82	12.66	15.53	8.25	0.44	#NULL!	0.00	1.00
14.04	1.81	14.43	14.53	8.90	0.53	1.00	1.00
9.00	2.37	10.10	13.62	12.08	1.54	1.00	1.00
28.38	9.26	35.48	24.56	72.14	2.03	1.00	1.00
24.42	4.14	30.59	20.59	21.87	1.27	1.00	1.00
1.66	11.74	20.69	9.82	0.69	#NULL!	0.00	1.00
11.63	1.70	14.22	7.39	16.28	1.21	1.00	1.00
14.98	0.67	20.74	13.43	14.09	1.21	0.00	1.00
14.65	4.21	16.87	25.52	36.11	1.40	1.00	1.00
1.41	17.22	11.07	6.84	1.10	#NULL!	0.00	1.00
10.90	2.25	12.74	9.69	7.03	2.07	1.00	1.00
0.64	15.10	15.73	11.87	0.63	#NULL!	0.00	1.00
11.88	1.61	14.58	14.27	7.22	1.27	0.00	1.00
23.10	0.39	28.05	20.68	1.18	0.69	0.00	1.00
1.97	10.25	16.09	12.63	0.96	#NULL!	0.00	1.00
19.80	1.42	25.65	27.55	5.96	2.44	1.00	1.00
1.54	11.42	15.09	3.83	1.57	#NULL!	0.00	1.00
12.95	2.08	15.44	10.82	11.53	1.68	1.00	1.00
6.45	1.16	8.47	14.35	26.91	0.77	0.00	1.00
7.26	1.97	8.19	14.85	12.63	0.62	1.00	1.00
22.75	2.03	25.56	32.23	13.86	2.37	1.00	1.00
1.97	8.84	14.97	12.63	0.85	#NULL!	0.00	1.00
27.14	1.28	29.83	33.29	11.89	3.64	1.00	1.00
1.93	10.04	19.13	8.74	0.64	#NULL!	0.00	1.00
22.63	1.58	25.92	11.61	8.92	1.06	0.00	1.00
14.52	0.25	20.10	14.78	0.69	1.22	0.00	1.00
20.62	1.72	25.03	24.98	6.03	1.91	1.00	1.00
25.57	3.99	29.39	39.86	18.64	1.59	1.00	1.00
2.82	32.05	24.35	11.52	2.39	#NULL!	0.00	1.00
2.44	23.02	24.32	12.78	0.75	#NULL!	0.00	1.00
16.29	1.19	19.57	40.49	3.44	1.66	1.00	1.00
1.43	23.39	30.32	8.04	3.24	#NULL!	0.00	1.00
15.28	2.14	17.11	14.81	15.70	1.63	1.00	1.00
18.00	3.42	22.28	18.55	15.64	0.67	0.00	1.00
12.62	0.63	14.73	13.36	3.81	1.44	0.00	1.00
14.78	2.59	18.11	19.40	14.02	0.95	0.00	1.00
21.05	3.06	23.71	23.46	10.89	2.72	1.00	1.00
24.46	1.09	29.97	16.39	5.72	2.12	1.00	1.00
32.39	0.67	35.10	19.06	0.68	2.03	1.00	1.00
25.31	9.05	35.96	44.05	27.40	1.36	1.00	1.00
0.73	32.00	27.95	11.42	3.40	#NULL!	0.00	1.00

12.53	1.24	16.17	5.44	7.72	1.74	1.00	1.00
17.23	0.25	22.34	17.94	5.69	1.49	1.00	1.00
27.42	4.65	33.08	33.26	19.64	1.40	1.00	1.00
0.40	14.90	8.20	12.85	1.60	#NULL!	0.00	1.00
14.00	2.16	19.12	16.76	5.52	1.49	1.00	1.00
1.84	22.45	26.46	13.82	2.01	#NULL!	0.00	1.00
30.11	1.74	34.26	20.92	15.13	4.69	1.00	1.00
12.78	1.65	16.41	14.68	5.29	1.08	0.00	1.00
11.07	1.97	13.87	16.15	5.89	1.41	1.00	1.00
0.69	37.86	26.06	11.81	4.76	#NULL!	0.00	1.00
2.31	39.59	45.77	11.28	2.54	#NULL!	0.00	1.00
6.83	1.89	8.08	13.11	12.63	0.62	1.00	1.00
14.25	3.63	17.01	12.36	14.32	1.52	1.00	1.00
17.13	2.05	19.76	15.33	9.15	1.81	1.00	0.00
14.86	2.31	15.89	18.25	8.95	0.80	1.00	1.00
15.33	2.00	20.40	7.94	14.57	2.11	1.00	1.00
12.96	0.56	15.12	6.80	2.57	2.48	1.00	1.00
8.85	1.17	9.04	11.05	10.04	0.86	1.00	1.00
19.70	4.31	22.99	23.11	28.79	1.49	1.00	1.00
17.92	2.14	24.33	22.37	6.27	1.53	1.00	1.00
18.00	3.42	22.28	18.55	15.64	0.67	0.00	1.00
12.60	2.59	15.70	17.16	8.21	1.55	1.00	1.00
12.70	1.99	17.44	28.25	16.48	0.88	0.00	1.00
13.66	4.09	15.62	12.67	14.49	1.24	1.00	1.00
16.69	2.16	21.09	20.12	12.16	0.94	0.00	1.00
16.70	0.63	18.99	8.67	1.84	2.11	1.00	1.00
11.52	1.58	10.52	17.33	9.43	0.75	1.00	1.00
3.28	26.88	25.90	16.38	4.69	#NULL!	0.00	1.00
19.48	0.26	23.93	9.49	12.51	3.51	1.00	1.00
14.04	1.90	17.07	22.90	10.08	1.51	0.00	1.00
17.31	4.07	18.99	13.23	22.04	1.78	1.00	1.00
11.57	1.93	10.04	19.13	8.74	0.64	1.00	1.00
11.71	3.08	16.58	9.11	8.11	1.57	0.00	1.00
16.01	2.24	17.15	15.92	8.16	0.72	0.00	1.00
25.04	4.72	29.86	18.91	13.14	1.29	1.00	1.00
35.50	0.67	39.77	32.35	11.81	4.95	1.00	1.00
26.57	2.84	32.30	42.60	19.20	1.96	1.00	1.00
32.46	1.15	37.14	25.26	3.64	1.84	1.00	1.00
10.08	2.28	12.13	9.59	7.03	1.37	0.00	1.00
16.49	1.83	20.00	29.48	12.62	1.55	1.00	1.00
27.70	4.07	34.82	20.97	23.63	2.21	1.00	1.00
32.36	1.26	38.89	32.14	4.84	1.76	1.00	1.00
27.42	0.95	28.10	29.46	14.21	1.56	1.00	1.00
24.73	3.57	31.36	19.14	20.58	0.82	0.00	1.00
21.07	2.16	24.39	19.22	14.26	2.74	0.00	1.00
12.94	3.69	17.71	32.10	9.73	1.18	0.00	1.00
14.47	1.83	14.39	20.03	11.75	0.47	1.00	1.00
1.78	14.23	15.67	8.92	0.53	#NULL!	0.00	1.00
16.40	0.52	21.97	36.06	4.11	0.77	0.00	1.00
17.12	4.12	20.47	14.53	21.77	1.25	1.00	1.00
14.44	1.82	14.28	19.93	8.46	1.24	1.00	1.00
13.83	0.41	16.86	9.28	12.85	1.60	1.00	1.00
11.68	1.66	16.64	17.58	15.69	0.98	1.00	1.00

8.65	1.03	9.86	12.20	3.02	1.20	0.00	1.00
15.08	3.36	18.84	28.69	56.76	1.78	1.00	1.00
2.27	16.61	15.40	8.16	0.67	#NULL!	0.00	1.00
20.46	1.84	24.79	26.48	6.85	2.11	0.00	1.00
12.84	7.80	16.04	13.13	21.97	0.99	1.00	1.00
15.34	0.40	18.62	8.62	12.85	1.67	1.00	1.00
16.63	5.42	22.18	26.12	20.71	0.92	1.00	1.00
16.76	2.80	22.00	12.73	13.79	1.34	1.00	1.00
15.18	2.51	16.74	10.46	13.17	1.17	1.00	1.00
11.64	2.18	13.98	7.24	7.55	3.08	0.00	1.00
26.30	1.28	28.76	34.12	11.89	3.34	0.00	1.00
10.29	1.97	11.62	25.52	10.86	1.85	0.00	1.00
13.68	1.80	14.19	17.37	10.82	0.58	0.00	1.00
15.95	3.20	19.58	20.10	18.28	0.76	1.00	1.00
25.67	3.61	31.63	27.98	21.71	2.04	1.00	1.00
9.78	3.17	11.50	4.26	13.59	2.48	1.00	0.00
16.62	4.60	21.39	13.22	16.72	2.82	1.00	1.00
8.85	1.17	9.04	11.05	10.04	0.86	1.00	1.00
16.33	3.42	20.20	17.70	13.09	0.52	0.00	1.00
19.39	1.56	24.00	18.00	8.63	1.08	1.00	1.00
2.35	21.88	20.78	11.11	0.74	#NULL!	0.00	1.00
41.30	0.70	47.74	24.85	5.73	5.39	1.00	1.00
16.68	1.92	20.11	25.76	17.39	1.38	1.00	1.00
14.56	2.21	15.91	26.53	7.35	1.69	1.00	1.00
10.08	2.28	12.13	9.59	7.03	1.37	1.00	1.00
20.36	4.05	26.46	11.32	23.71	1.64	1.00	1.00
10.01	2.48	11.36	13.50	12.36	2.16	0.00	1.00
15.08	3.82	19.76	8.13	10.45	1.74	1.00	1.00
15.89	0.64	18.63	28.33	2.04	0.90	0.00	1.00
11.34	3.39	12.25	18.52	14.16	0.97	0.00	1.00
25.77	4.06	31.12	38.01	9.98	1.67	1.00	1.00

vit.e.2	vt.a.rae2	vit.c	vit.c2	DAQS	DAQS_Tertile
0.00	1.00	143.94	1.00	4.00	2.00
0.00	1.00	302.66	1.00	4.00	2.00
0.00	0.00	41.99	1.00	1.00	0.00
0.00	1.00	155.25	1.00	3.00	1.00
0.00	0.00	59.46	0.00	2.00	0.00
0.00	1.00	187.28	1.00	4.00	2.00
0.00	0.00	205.43	1.00	3.00	1.00
0.00	0.00	101.63	1.00	3.00	1.00
0.00	0.00	1234.29	1.00	2.00	0.00
0.00	0.00	75.16	0.00	1.00	0.00
0.00	0.00	934.75	1.00	2.00	0.00
1.00	1.00	183.87	1.00	5.00	2.00
0.00	1.00	1385.52	1.00	3.00	1.00
0.00	0.00	133.60	1.00	3.00	1.00
0.00	0.00	105.69	1.00	3.00	1.00
0.00	1.00	197.24	1.00	3.00	1.00
1.00	1.00	71.80	0.00	4.00	2.00
0.00	0.00	103.68	1.00	3.00	1.00
0.00	0.00	81.34	1.00	2.00	0.00
0.00	0.00	55.64	0.00	1.00	0.00
0.00	0.00	67.52	0.00	2.00	0.00
1.00	0.00	150.54	1.00	4.00	2.00
0.00	1.00	250.95	1.00	4.00	2.00
1.00	0.00	191.74	1.00	4.00	2.00
0.00	1.00	302.66	1.00	4.00	2.00
0.00	0.00	84.39	1.00	3.00	1.00
1.00	0.00	198.89	1.00	4.00	2.00
0.00	1.00	255.22	1.00	4.00	2.00
1.00	0.00	65.11	1.00	3.00	1.00
0.00	0.00	77.34	1.00	3.00	1.00
0.00	0.00	71.27	0.00	2.00	0.00
1.00	0.00	108.88	1.00	4.00	2.00
0.00	1.00	206.41	1.00	3.00	1.00
1.00	1.00	248.09	1.00	5.00	2.00
0.00	1.00	913.54	1.00	3.00	1.00
0.00	1.00	1325.19	1.00	3.00	1.00
0.00	0.00	64.28	0.00	2.00	0.00
0.00	0.00	189.18	1.00	3.00	1.00
0.00	0.00	53.13	0.00	1.00	0.00
0.00	0.00	87.43	1.00	1.00	0.00
1.00	0.00	107.20	1.00	3.00	1.00
1.00	1.00	128.55	1.00	5.00	2.00
0.00	0.00	59.53	0.00	2.00	0.00
0.00	0.00	76.97	1.00	1.00	0.00
0.00	1.00	280.59	1.00	4.00	2.00
0.00	0.00	55.39	0.00	1.00	0.00
0.00	0.00	207.20	1.00	3.00	1.00
0.00	0.00	0.00	0.00	1.00	0.00
0.00	1.00	121.29	1.00	3.00	1.00
1.00	1.00	1213.18	1.00	4.00	2.00
0.00	0.00	150.13	1.00	3.00	1.00
0.00	0.00	153.29	1.00	2.00	0.00

0.00	0.00	84.76	1.00	2.00	0.00
0.00	0.00	77.50	1.00	3.00	1.00
0.00	0.00	90.97	1.00	3.00	1.00
0.00	1.00	942.06	1.00	3.00	1.00
1.00	1.00	1353.12	1.00	4.00	2.00
1.00	1.00	270.65	1.00	5.00	2.00
0.00	1.00	1141.56	1.00	3.00	1.00
1.00	0.00	79.84	1.00	4.00	2.00
1.00	0.00	36.07	0.00	3.00	1.00
0.00	1.00	1348.34	1.00	3.00	1.00
0.00	1.00	1627.91	1.00	3.00	1.00
0.00	1.00	156.46	1.00	3.00	1.00
0.00	0.00	59.57	1.00	2.00	0.00
0.00	1.00	483.45	1.00	4.00	2.00
0.00	0.00	65.40	0.00	1.00	0.00
0.00	1.00	1385.52	1.00	3.00	1.00
0.00	1.00	2411.11	1.00	3.00	1.00
1.00	0.00	207.45	1.00	4.00	2.00
0.00	0.00	87.43	1.00	1.00	0.00
0.00	0.00	137.84	1.00	3.00	1.00
1.00	1.00	1029.20	1.00	4.00	2.00
0.00	1.00	1069.88	1.00	3.00	1.00
0.00	0.00	70.53	1.00	1.00	0.00
0.00	0.00	155.41	1.00	3.00	1.00
0.00	1.00	869.22	1.00	3.00	1.00
0.00	0.00	105.12	1.00	3.00	1.00
0.00	0.00	137.42	1.00	2.00	0.00
0.00	0.00	108.49	1.00	2.00	0.00
0.00	1.00	1522.83	1.00	3.00	1.00
1.00	1.00	428.85	1.00	5.00	2.00
0.00	1.00	1600.27	1.00	3.00	1.00
0.00	0.00	88.97	1.00	3.00	1.00
1.00	0.00	141.83	1.00	4.00	2.00
0.00	1.00	278.61	1.00	4.00	2.00
0.00	1.00	1204.84	1.00	3.00	1.00
0.00	0.00	47.84	0.00	2.00	0.00
0.00	0.00	58.91	0.00	1.00	0.00
0.00	0.00	185.04	1.00	3.00	1.00
0.00	0.00	115.37	1.00	2.00	0.00
0.00	0.00	78.18	1.00	3.00	1.00
0.00	1.00	809.28	1.00	3.00	1.00
0.00	0.00	1102.78	1.00	2.00	0.00
1.00	1.00	252.40	1.00	5.00	2.00
0.00	0.00	79.80	0.00	1.00	0.00
0.00	1.00	125.25	1.00	4.00	2.00
0.00	1.00	228.51	1.00	4.00	2.00
0.00	0.00	74.59	1.00	1.00	0.00
0.00	0.00	228.04	1.00	2.00	0.00
1.00	0.00	150.83	1.00	3.00	1.00
0.00	0.00	110.62	1.00	3.00	1.00
0.00	0.00	173.27	1.00	2.00	0.00
0.00	1.00	203.43	1.00	4.00	2.00
0.00	1.00	1147.08	1.00	3.00	1.00

1.00	1.00	1878.13	1.00	4.00	2.00
1.00	1.00	1601.52	1.00	4.00	2.00
0.00	0.00	101.99	1.00	2.00	0.00
0.00	1.00	171.19	1.00	4.00	2.00
0.00	0.00	181.44	1.00	3.00	1.00
0.00	1.00	559.11	1.00	3.00	1.00
0.00	0.00	58.73	0.00	2.00	0.00
0.00	1.00	92.53	1.00	4.00	2.00
0.00	0.00	124.76	1.00	2.00	0.00
1.00	1.00	334.23	1.00	5.00	2.00
1.00	0.00	85.66	1.00	3.00	1.00
1.00	0.00	101.41	1.00	4.00	2.00
1.00	1.00	334.44	1.00	5.00	2.00
1.00	1.00	1591.59	1.00	4.00	2.00
0.00	0.00	82.19	1.00	2.00	0.00
0.00	0.00	242.12	1.00	2.00	0.00
1.00	0.00	86.46	1.00	3.00	1.00
0.00	1.00	115.64	1.00	4.00	2.00
0.00	0.00	159.45	1.00	3.00	1.00
1.00	1.00	806.51	1.00	4.00	2.00
0.00	1.00	124.47	1.00	4.00	2.00
0.00	1.00	285.79	1.00	4.00	2.00
0.00	0.00	190.70	1.00	3.00	1.00
0.00	0.00	71.94	0.00	1.00	0.00
0.00	0.00	95.88	1.00	3.00	1.00
0.00	1.00	161.59	1.00	4.00	2.00
0.00	1.00	310.71	1.00	4.00	2.00
0.00	0.00	150.42	0.00	1.00	0.00
0.00	1.00	214.64	0.00	3.00	1.00
1.00	1.00	79.84	0.00	4.00	2.00
1.00	0.00	244.91	1.00	3.00	1.00
0.00	0.00	1601.52	1.00	2.00	0.00
1.00	0.00	84.64	1.00	4.00	2.00
0.00	0.00	105.72	1.00	3.00	1.00
0.00	1.00	215.08	1.00	4.00	2.00
0.00	0.00	109.93	1.00	3.00	1.00
0.00	1.00	291.58	1.00	4.00	2.00
0.00	1.00	124.98	1.00	4.00	2.00
0.00	0.00	156.90	1.00	3.00	1.00
0.00	0.00	162.03	1.00	3.00	1.00
0.00	1.00	343.76	1.00	4.00	2.00
0.00	1.00	71.16	0.00	3.00	1.00
0.00	1.00	202.86	1.00	3.00	1.00
0.00	0.00	103.84	1.00	3.00	1.00
0.00	0.00	122.99	1.00	2.00	0.00
0.00	0.00	185.79	1.00	2.00	0.00
0.00	1.00	732.18	1.00	3.00	1.00
0.00	1.00	251.32	1.00	4.00	2.00
0.00	0.00	107.90	1.00	2.00	0.00
0.00	0.00	128.39	1.00	3.00	1.00
0.00	0.00	66.36	1.00	2.00	0.00
0.00	0.00	154.57	1.00	3.00	1.00
0.00	1.00	190.57	1.00	4.00	2.00

0.00	0.00	68.03	0.00	2.00	0.00
0.00	0.00	299.87	1.00	3.00	1.00
1.00	0.00	134.15	1.00	4.00	2.00
0.00	1.00	1522.83	1.00	3.00	1.00
0.00	0.00	105.72	1.00	3.00	1.00
0.00	0.00	207.56	1.00	2.00	0.00
0.00	0.00	78.73	1.00	3.00	1.00
0.00	0.00	64.49	0.00	2.00	0.00
0.00	0.00	72.07	0.00	2.00	0.00
0.00	1.00	300.35	1.00	4.00	2.00
0.00	1.00	746.86	1.00	3.00	1.00
0.00	1.00	1018.50	1.00	3.00	1.00
0.00	1.00	1575.64	1.00	3.00	1.00
0.00	1.00	213.97	1.00	4.00	2.00
1.00	1.00	72.02	0.00	4.00	2.00
0.00	1.00	347.55	1.00	3.00	1.00
0.00	0.00	380.90	1.00	3.00	1.00
0.00	1.00	2350.04	1.00	3.00	1.00
0.00	1.00	120.32	1.00	4.00	2.00
0.00	0.00	153.09	1.00	2.00	0.00
1.00	1.00	193.46	1.00	5.00	2.00
1.00	1.00	1364.31	1.00	4.00	2.00
0.00	0.00	55.55	0.00	2.00	0.00
0.00	1.00	874.44	1.00	3.00	1.00
1.00	0.00	107.01	1.00	3.00	1.00
1.00	1.00	267.89	1.00	4.00	2.00
0.00	0.00	920.34	1.00	2.00	0.00
1.00	1.00	202.46	1.00	5.00	2.00
0.00	1.00	699.17	1.00	3.00	1.00
0.00	0.00	68.22	0.00	2.00	0.00
0.00	0.00	45.91	1.00	1.00	0.00
1.00	0.00	53.79	0.00	3.00	1.00
0.00	1.00	102.00	1.00	4.00	2.00
0.00	1.00	862.94	1.00	3.00	1.00
0.00	0.00	70.59	0.00	2.00	0.00
0.00	1.00	2323.56	1.00	3.00	1.00
0.00	0.00	148.34	1.00	2.00	0.00
0.00	0.00	182.46	1.00	2.00	0.00
1.00	0.00	239.81	1.00	4.00	2.00
0.00	1.00	422.12	1.00	4.00	2.00
0.00	0.00	1005.43	1.00	2.00	0.00
0.00	1.00	1247.87	1.00	3.00	1.00
0.00	0.00	210.67	1.00	3.00	1.00
0.00	1.00	1015.46	1.00	3.00	1.00
1.00	1.00	84.63	1.00	5.00	2.00
0.00	0.00	173.00	1.00	2.00	0.00
0.00	0.00	98.74	1.00	2.00	0.00
0.00	0.00	162.03	1.00	2.00	0.00
1.00	1.00	84.76	1.00	5.00	2.00
1.00	0.00	179.36	1.00	4.00	2.00
1.00	1.00	276.67	1.00	5.00	2.00
0.00	0.00	300.89	1.00	3.00	1.00
0.00	1.00	755.11	1.00	3.00	1.00

0.00	0.00	70.34	0.00	2.00	0.00
0.00	0.00	131.91	1.00	3.00	1.00
1.00	1.00	246.79	1.00	5.00	2.00
0.00	1.00	1183.73	1.00	3.00	1.00
0.00	0.00	163.75	1.00	3.00	1.00
0.00	1.00	976.73	1.00	3.00	1.00
0.00	1.00	89.02	0.00	3.00	1.00
1.00	0.00	188.70	1.00	3.00	1.00
0.00	0.00	80.45	1.00	3.00	1.00
0.00	1.00	811.38	1.00	3.00	1.00
0.00	1.00	1091.87	1.00	3.00	1.00
1.00	0.00	63.93	0.00	3.00	1.00
0.00	0.00	91.70	1.00	3.00	1.00
0.00	0.00	164.00	1.00	2.00	0.00
0.00	0.00	204.60	1.00	3.00	1.00
1.00	1.00	88.67	0.00	4.00	2.00
1.00	0.00	91.15	1.00	4.00	2.00
0.00	0.00	105.72	1.00	3.00	1.00
0.00	1.00	202.86	1.00	4.00	2.00
0.00	0.00	92.62	1.00	3.00	1.00
0.00	0.00	173.00	1.00	2.00	0.00
0.00	0.00	116.53	1.00	3.00	1.00
0.00	0.00	93.56	1.00	2.00	0.00
0.00	1.00	127.45	1.00	4.00	2.00
0.00	0.00	207.56	1.00	2.00	0.00
1.00	0.00	106.62	1.00	4.00	2.00
0.00	1.00	222.78	1.00	4.00	2.00
0.00	0.00	1341.19	1.00	2.00	0.00
1.00	0.00	100.05	1.00	4.00	2.00
1.00	0.00	119.64	1.00	3.00	1.00
1.00	1.00	140.98	1.00	5.00	2.00
0.00	1.00	235.59	1.00	4.00	2.00
0.00	0.00	58.21	1.00	1.00	0.00
0.00	1.00	190.02	1.00	3.00	1.00
0.00	1.00	200.11	1.00	4.00	2.00
0.00	1.00	93.06	0.00	3.00	1.00
1.00	1.00	455.87	1.00	5.00	2.00
0.00	0.00	179.21	1.00	3.00	1.00
0.00	0.00	66.09	0.00	1.00	0.00
0.00	1.00	213.58	1.00	4.00	2.00
0.00	1.00	296.64	1.00	4.00	2.00
1.00	0.00	308.25	1.00	4.00	2.00
1.00	1.00	366.59	1.00	5.00	2.00
0.00	0.00	154.45	1.00	2.00	0.00
0.00	0.00	87.61	1.00	1.00	0.00
0.00	0.00	181.74	1.00	2.00	0.00
0.00	1.00	238.92	1.00	4.00	2.00
0.00	0.00	1627.91	1.00	2.00	0.00
0.00	1.00	130.95	1.00	3.00	1.00
0.00	0.00	194.93	1.00	3.00	1.00
0.00	1.00	236.84	1.00	4.00	2.00
1.00	0.00	92.90	1.00	4.00	2.00
0.00	0.00	128.08	1.00	3.00	1.00

0.00	0.00	101.34	1.00	2.00	0.00
0.00	1.00	146.89	1.00	4.00	2.00
0.00	0.00	1339.68	1.00	2.00	0.00
0.00	0.00	100.00	1.00	2.00	0.00
1.00	0.00	104.76	1.00	4.00	2.00
1.00	0.00	96.72	1.00	4.00	2.00
0.00	0.00	107.90	1.00	3.00	1.00
0.00	1.00	111.52	1.00	4.00	2.00
0.00	0.00	140.00	1.00	3.00	1.00
0.00	0.00	41.99	0.00	1.00	0.00
0.00	0.00	71.76	0.00	1.00	0.00
1.00	1.00	73.94	0.00	3.00	1.00
0.00	0.00	214.04	1.00	2.00	0.00
0.00	1.00	198.96	1.00	4.00	2.00
1.00	1.00	270.55	1.00	5.00	2.00
0.00	0.00	78.00	1.00	2.00	0.00
0.00	0.00	98.54	1.00	3.00	1.00
0.00	0.00	105.72	1.00	3.00	1.00
0.00	0.00	153.29	1.00	2.00	0.00
0.00	1.00	155.28	1.00	4.00	2.00
0.00	1.00	1234.29	1.00	3.00	1.00
0.00	1.00	90.23	1.00	4.00	2.00
0.00	1.00	112.66	1.00	4.00	2.00
0.00	1.00	85.34	1.00	4.00	2.00
0.00	0.00	66.09	0.00	2.00	0.00
0.00	0.00	155.74	1.00	3.00	1.00
1.00	0.00	73.26	0.00	2.00	0.00
0.00	0.00	139.65	1.00	3.00	1.00
0.00	0.00	227.72	1.00	2.00	0.00
0.00	0.00	165.97	1.00	2.00	0.00
0.00	0.00	181.74	0.00	2.00	0.00
