# Supplementary material for: Association between dietary antioxidant quality score and severity of coronavirus infection: a case–control study
Source: Front Nutr. 2023 Jul 6;10:1174113. doi: 10.3389/fnut.2023.1174113 (PMC10358364; doi:10.3389/fnut.2023.1174113)
Supplement: Supplementary file 4 [file Data_Sheet_4.PDF]

```
--------------------------------------------------------------------------------------------------------------------------------
-----------------------------------------------
      name:  <unnamed>
       log:  D:\Ms.Aghajani-6.14\Untitled.smcl
  log type:  smcl
 opened on:  13 May 2023, 10:29:28

. do "D:\Ms.Aghajani-6.14\DOfile.aghajani.do"


.

.

. ## TABLE 1
Unknown #command
. ttest Age, by(Bastary_Satus)


Two-sample t test with equal variances
------------------------------------------------------------------------------
   Group |     Obs        Mean    Std. err.   Std. dev.   [95% conf. interval]
---------+--------------------------------------------------------------------
       0 |     191     56.5445    1.071402    14.80707    54.43113    58.65787
 General |     104    62.44231    1.496438    15.26073    59.47448    65.41014
---------+--------------------------------------------------------------------
Combined |     295    58.62373    .8853786    15.20688    56.88125    60.36621
---------+--------------------------------------------------------------------
    diff |           -5.897805    1.824084               -9.487772   -2.307838
------------------------------------------------------------------------------
    diff = mean(0) - mean(General)                                t =  -3.2333
H0: diff = 0                                     Degrees of freedom =      293
```

Ha: diff < 0 Ha: diff != 0 Ha: diff > 0

Pr(T < t) = 0.0007 Pr(|T| > |t|) = 0.0014 Pr(T > t) = 0.9993

. ttest Wieght, by(Bastary_Satus)

Two-sample t test with equal variances

Group	Obs	Mean	Std. err.	Std. dev.	[95% conf. interval]	
0	191	76.2199	.9298451	12.85071	74.38575	78.05404
General	104	77.5	1.248898	12.73631	75.02311	79.97689
Combined	295	76.67119	.745447	12.80347	75.2041	78.13828
diff		-1.280105	1.561161		-4.352615	1.792405

diff = mean(0) - mean(General) t = -0.8200

H0: diff = 0 Degrees of freedom = 293

Ha: diff < 0 Ha: diff != 0 Ha: diff > 0

Pr(T < t) = 0.2064 Pr(|T| > |t|) = 0.4129 Pr(T > t) = 0.7936

. ttest BMI , by(Bastary_Satus)

Two-sample t test with equal variances

Group	Obs	Mean	Std. err.	Std. dev.	[95% conf. interval]	
0	191	27.45402	.2967201	4.100753	26.86873	28.0393

```
 General |     104    28.31045    .4643362    4.735318    27.38955    29.23135
---------+--------------------------------------------------------------------
Combined |     295    27.75594    .2530582    4.346418    27.25791    28.25398
---------+--------------------------------------------------------------------
    diff |           -.8564315    .5282134               -1.896005    .1831418
------------------------------------------------------------------------------
    diff = mean(0) - mean(General)                                t =  -1.6214
H0: diff = 0                                     Degrees of freedom =      293

    Ha: diff < 0                 Ha: diff != 0                 Ha: diff > 0
 Pr(T < t) = 0.0530         Pr(|T| > |t|) = 0.1060          Pr(T > t) = 0.9470

. ttest HSCRP , by(Bastary_Satus)

Two-sample t test with equal variances
------------------------------------------------------------------------------
   Group |     Obs        Mean    Std. err.   Std. dev.   [95% conf. interval]
---------+--------------------------------------------------------------------
       0 |     190    9.072842    1.295418    17.85611    6.517507    11.62818
 General |     104    15.99519    1.971656    20.10703    12.08488    19.90551
---------+--------------------------------------------------------------------
Combined |     294    11.52156    1.104696    18.94159     9.34742    13.69571
---------+--------------------------------------------------------------------
    diff |            -6.92235    2.278674               -11.40706   -2.437643
------------------------------------------------------------------------------
    diff = mean(0) - mean(General)                                t =  -3.0379
H0: diff = 0                                     Degrees of freedom =      292

    Ha: diff < 0                 Ha: diff != 0                 Ha: diff > 0
```

```
 Pr(T < t) = 0.0013         Pr(|T| > |t|) = 0.0026          Pr(T > t) = 0.9987

. ttest PH , by(Bastary_Satus)

Two-sample t test with equal variances
------------------------------------------------------------------------------
   Group |     Obs        Mean    Std. err.   Std. dev.   [95% conf. interval]
---------+--------------------------------------------------------------------
       0 |     189    7.378603    .0113214    .1556441     7.35627    7.400937
 General |     104    7.334413    .0211111    .2152914    7.292545    7.376282
---------+--------------------------------------------------------------------
Combined |     293    7.362918    .0105149    .1799867    7.342223    7.383613
---------+--------------------------------------------------------------------
    diff |            .0441897    .0218597                .0011666    .0872128
------------------------------------------------------------------------------
    diff = mean(0) - mean(General)                                t =   2.0215
H0: diff = 0                                     Degrees of freedom =      291

    Ha: diff < 0                 Ha: diff != 0                 Ha: diff > 0
 Pr(T < t) = 0.9779         Pr(|T| > |t|) = 0.0441          Pr(T > t) = 0.0221

. ttest PCO2 , by(Bastary_Satus)

Two-sample t test with equal variances
------------------------------------------------------------------------------
   Group |     Obs        Mean    Std. err.   Std. dev.   [95% conf. interval]
---------+--------------------------------------------------------------------
       0 |     191    41.32147    1.629541     22.5207    38.10715    44.53578
 General |     104    39.56442    1.206183    12.30071    37.17224     41.9566
```

```
---------+--------------------------------------------------------------------
Combined |     295    40.70203    1.137179    19.53169    38.46399    42.94008
---------+--------------------------------------------------------------------
    diff |            1.757043    2.382069               -2.931092    6.445178
------------------------------------------------------------------------------
    diff = mean(0) - mean(General)                                t =   0.7376
H0: diff = 0                                     Degrees of freedom =      293

    Ha: diff < 0                 Ha: diff != 0                 Ha: diff > 0
 Pr(T < t) = 0.7693         Pr(|T| > |t|) = 0.4613          Pr(T > t) = 0.2307

. ttest BE , by(Bastary_Satus)

Two-sample t test with equal variances
------------------------------------------------------------------------------
   Group |     Obs        Mean    Std. err.   Std. dev.   [95% conf. interval]
---------+--------------------------------------------------------------------
       0 |     191   -2.396859    .3806717    5.260988   -3.147744   -1.645973
 General |     104   -3.433654    .5279698    5.384256   -4.480757    -2.38655
---------+--------------------------------------------------------------------
Combined |     295   -2.762373    .3096733    5.318813    -3.37183   -2.152916
---------+--------------------------------------------------------------------
    diff |            1.036795    .6464489               -.2354766    2.309067
------------------------------------------------------------------------------
    diff = mean(0) - mean(General)                                t =   1.6038
H0: diff = 0                                     Degrees of freedom =      293

    Ha: diff < 0                 Ha: diff != 0                 Ha: diff > 0
 Pr(T < t) = 0.9451         Pr(|T| > |t|) = 0.1098          Pr(T > t) = 0.0549
```

```
. ttest BEecf , by(Bastary_Satus)

Two-sample t test with equal variances
------------------------------------------------------------------------------
   Group |     Obs        Mean    Std. err.   Std. dev.   [95% conf. interval]
---------+--------------------------------------------------------------------
       0 |     191   -2.113613    .5219842    7.213965   -3.143241   -1.083984
 General |     104   -3.719231    .6285334    6.409809   -4.965779   -2.472683
---------+--------------------------------------------------------------------
Combined |     295   -2.679661    .4059578    6.972555   -3.478613   -1.880709
---------+--------------------------------------------------------------------
    diff |            1.605618    .8459721               -.059334     3.27057
------------------------------------------------------------------------------
    diff = mean(0) - mean(General)                                t =   1.8980
H0: diff = 0                                     Degrees of freedom =      293

    Ha: diff < 0                 Ha: diff != 0                 Ha: diff > 0
 Pr(T < t) = 0.9707         Pr(|T| > |t|) = 0.0587          Pr(T > t) = 0.0293

. ttest BB , by(Bastary_Satus)

Two-sample t test with equal variances
------------------------------------------------------------------------------
   Group |     Obs        Mean    Std. err.   Std. dev.   [95% conf. interval]
---------+--------------------------------------------------------------------
       0 |     191    44.79948    .6671803    9.220615    43.48344    46.11551
 General |     104    44.17885    .6128885    6.250261    42.96333    45.39437
---------+--------------------------------------------------------------------
```

```
Combined |     295    44.58068    .4826454    8.289708    43.6308    45.53056
---------+--------------------------------------------------------------------
    diff |            .6206303    1.011295               -1.369692    2.610953
------------------------------------------------------------------------------
    diff = mean(0) - mean(General)                              t =   0.6137
H0: diff = 0                                   Degrees of freedom =      293

    Ha: diff < 0                 Ha: diff != 0                 Ha: diff > 0
 Pr(T < t) = 0.7301         Pr(|T| > |t|) = 0.5399          Pr(T > t) = 0.2699

. ttest HCO3 , by(Bastary_Satus)

Two-sample t test with equal variances
------------------------------------------------------------------------------
   Group |     Obs        Mean    Std. err.   Std. dev.   [95% conf. interval]
---------+--------------------------------------------------------------------
       0 |     191        22.4    .3318195    4.585836    21.74548    23.05452
 General |     104    21.56058     .585486     5.97081     20.3994    22.72175
---------+--------------------------------------------------------------------
Combined |     295    22.10407    .2982546     5.12269    21.51708    22.69105
---------+--------------------------------------------------------------------
    diff |            .8394231    .6234133               -.3875126    2.066359
------------------------------------------------------------------------------
    diff = mean(0) - mean(General)                              t =   1.3465
H0: diff = 0                                   Degrees of freedom =      293

    Ha: diff < 0                 Ha: diff != 0                 Ha: diff > 0
 Pr(T < t) = 0.9104         Pr(|T| > |t|) = 0.1792          Pr(T > t) = 0.0896
```

```
. ttest PO2 , by(Bastary_Satus)

Two-sample t test with equal variances
------------------------------------------------------------------------------
   Group |     Obs        Mean    Std. err.   Std. dev.   [95% conf. interval]
---------+--------------------------------------------------------------------
       0 |     191    38.51455    1.335824    18.46145     35.8796    41.14951
 General |     104    39.13462    1.763403    17.98325    35.63732    42.63191
---------+--------------------------------------------------------------------
Combined |     295    38.73315     1.06349    18.26604    36.64013    40.82617
---------+--------------------------------------------------------------------
    diff |           -.6200604    2.229486               -5.007896    3.767775
------------------------------------------------------------------------------
    diff = mean(0) - mean(General)                                t =  -0.2781
H0: diff = 0                                     Degrees of freedom =      293

    Ha: diff < 0                 Ha: diff != 0                 Ha: diff > 0
 Pr(T < t) = 0.3906         Pr(|T| > |t|) = 0.7811          Pr(T > t) = 0.6094

. ttest O2sat , by(Bastary_Satus)

Two-sample t test with equal variances
------------------------------------------------------------------------------
   Group |     Obs        Mean    Std. err.   Std. dev.   [95% conf. interval]
---------+--------------------------------------------------------------------
       0 |     191    65.14304    1.196214    16.53201    62.78347     67.5026
 General |     104     62.3274    1.786899    18.22287    58.78351     65.8713
---------+--------------------------------------------------------------------
Combined |     295    64.15041    .9996293     17.1692    62.18307    66.11774
```

```
---------+--------------------------------------------------------------------
    diff |          2.815633    2.08942             -1.296542    6.927808
------------------------------------------------------------------------------
    diff = mean(0) - mean(General)                          t =   1.3476
H0: diff = 0                             Degrees of freedom =      293

    Ha: diff < 0              Ha: diff != 0              Ha: diff > 0
 Pr(T < t) = 0.9106        Pr(|T| > |t|) = 0.1788        Pr(T > t) = 0.0894

. ttest Ferritin , by(Bastary_Satus)

Two-sample t test with equal variances
------------------------------------------------------------------------------
   Group |     Obs        Mean    Std. err.   Std. dev.   [95% conf. interval]
---------+--------------------------------------------------------------------
       0 |     191    376.1675    12.08065     166.958    352.3381     399.997
 General |     104    289.4683    16.01096    163.2804    257.7143    321.2222
---------+--------------------------------------------------------------------
Combined |     295    345.6024    9.927919    170.5176    326.0636    365.1412
---------+--------------------------------------------------------------------
    diff |          86.69927    20.18986              46.96374    126.4348
------------------------------------------------------------------------------
    diff = mean(0) - mean(General)                          t =   4.2942
H0: diff = 0                             Degrees of freedom =      293

    Ha: diff < 0              Ha: diff != 0              Ha: diff > 0
 Pr(T < t) = 1.0000        Pr(|T| > |t|) = 0.0000        Pr(T > t) = 0.0000

. ttest DDimer , by(Bastary_Satus)
```

```
Two-sample t test with equal variances
------------------------------------------------------------------------------
   Group |     Obs        Mean    Std. err.   Std. dev.   [95% conf. interval]
---------+--------------------------------------------------------------------
       0 |     191    .9884293     .081108    1.120935    .8284415    1.148417
 General |     103    1.398058    .1564688    1.587985    1.087703    1.708413
---------+--------------------------------------------------------------------
Combined |     294    1.131939    .0767311    1.315664    .9809248    1.282953
---------+--------------------------------------------------------------------
    diff |           -.4096289    .1593179               -.7231859   -.096072
------------------------------------------------------------------------------
    diff = mean(0) - mean(General)                                t =  -2.5711
H0: diff = 0                                     Degrees of freedom =      292

    Ha: diff < 0                 Ha: diff != 0                 Ha: diff > 0
 Pr(T < t) = 0.0053         Pr(|T| > |t|) = 0.0106          Pr(T > t) = 0.9947

. ttest LDH , by(Bastary_Satus)

Two-sample t test with equal variances
------------------------------------------------------------------------------
   Group |     Obs        Mean    Std. err.   Std. dev.   [95% conf. interval]
---------+--------------------------------------------------------------------
       0 |     187    590.7219    16.03431    219.2659    559.0894    622.3544
 General |     104    712.5769     27.1318    276.6911    658.7674    766.3864
---------+--------------------------------------------------------------------
Combined |     291    634.2715    14.53153    247.8894    605.6708    662.8721
---------+--------------------------------------------------------------------
```

```
    diff |           -121.855    29.51719              -179.9509   -63.75908
------------------------------------------------------------------------------
    diff = mean(0) - mean(General)                            t =  -4.1283
H0: diff = 0                                     Degrees of freedom =      289

    Ha: diff < 0                 Ha: diff != 0                 Ha: diff > 0
 Pr(T < t) = 0.0000         Pr(|T| > |t|) = 0.0000          Pr(T > t) = 1.0000

. ttest CPK , by(Bastary_Satus)

Two-sample t test with equal variances
------------------------------------------------------------------------------
   Group |     Obs        Mean    Std. err.   Std. dev.   [95% conf. interval]
---------+--------------------------------------------------------------------
       0 |     190    104.8411    17.68249    243.7362    69.96067    139.7214
 General |     104    213.1154    26.19894    267.1778     161.156    265.0748
---------+--------------------------------------------------------------------
Combined |     294    143.1422    14.99479     257.107     113.631    172.6533
---------+--------------------------------------------------------------------
    diff |          -108.2743    30.76932              -168.8321   -47.71657
------------------------------------------------------------------------------
    diff = mean(0) - mean(General)                            t =  -3.5189
H0: diff = 0                                     Degrees of freedom =      292

    Ha: diff < 0                 Ha: diff != 0                 Ha: diff > 0
 Pr(T < t) = 0.0003         Pr(|T| > |t|) = 0.0005          Pr(T > t) = 0.9997

. ttest CKMB , by(Bastary_Satus)
```

```
Two-sample t test with equal variances
------------------------------------------------------------------------------
   Group |     Obs        Mean    Std. err.   Std. dev.   [95% conf. interval]
---------+--------------------------------------------------------------------
       0 |     188    19.48085    2.718617    37.27579    14.11775    24.84395
 General |     104    32.78846    4.912427    50.09712    23.04582     42.5311
---------+--------------------------------------------------------------------
Combined |     292    24.22055    2.497923    42.68453    19.30426    29.13683
---------+--------------------------------------------------------------------
    diff |           -13.30761    5.166567               -23.47633   -3.138887
------------------------------------------------------------------------------
    diff = mean(0) - mean(General)                                t =  -2.5757
H0: diff = 0                                     Degrees of freedom =      290

    Ha: diff < 0                 Ha: diff != 0                 Ha: diff > 0
 Pr(T < t) = 0.0052         Pr(|T| > |t|) = 0.0105          Pr(T > t) = 0.9948


.
.
.
. ## TABLE 2
Unknown #command
.
. oneway Age DAQS_Tertile , tabulate

DAQS_Tertil |          Summary of Age
          e |        Mean   Std. dev.       Freq.
------------+------------------------------------
       0.00 |        59.4        16.6          85
```

```
      1.00 |       57.6       14.1        114
      2.00 |       59.2       15.2         96
------------+------------------------------------
     Total |       58.6       15.2        295

                        Analysis of variance
    Source              SS         df      MS            F     Prob > F
------------------------------------------------------------------------
Between groups      203.62641      2   101.813205      0.44     0.6454
 Within groups      67783.6075    292   232.135642
------------------------------------------------------------------------
    Total           67987.2339    294   231.249095

Bartlett's equal-variances test: chi2(2) =   2.4828    Prob>chi2 = 0.289

. oneway Wieght DAQS_Tertile , tabulate

DAQS_Tertil |          Summary of Wieght
          e |       Mean   Std. dev.       Freq.
------------+------------------------------------
      0.00 |         77         13          85
      1.00 |         78         13         114
      2.00 |         75         13          96
------------+------------------------------------
     Total |         77         13         295

                        Analysis of variance
    Source              SS         df      MS            F     Prob > F
------------------------------------------------------------------------
```

```
Between groups      309.507761      2   154.753881      0.94     0.3904
 Within groups      47885.5973    292   163.991772
------------------------------------------------------------------------
    Total          48195.1051    294   163.928929

Bartlett's equal-variances test: chi2(2) =   0.3118    Prob>chi2 = 0.856

. oneway BMI DAQS_Tertile , tabulate

DAQS_Tertil |          Summary of BMI
          e |        Mean   Std. dev.       Freq.
------------+------------------------------------
       0.00 |       27.64       4.18          85
       1.00 |       28.34       4.48         114
       2.00 |       27.16       4.28          96
------------+------------------------------------
      Total |       27.76       4.35         295

                        Analysis of variance
    Source              SS         df      MS            F     Prob > F
------------------------------------------------------------------------
Between groups      73.4813639      2    36.740682      1.96     0.1431
 Within groups      5480.57473    292   18.7690915
------------------------------------------------------------------------
    Total          5554.05609    294   18.8913472

Bartlett's equal-variances test: chi2(2) =   0.5129    Prob>chi2 = 0.774

. tabulate Sex DAQS_Tertile , chi2 column
```

```
+-------------------+
| Key               |
|-------------------|
|     frequency     |
| column percentage |
+-------------------+

           |          DAQS_Tertile
       Sex |      0.00       1.00       2.00 |     Total
-----------+---------------------------------+----------
         1 |        46         52         40 |       138
           |     54.12      45.61      41.67 |     46.78
-----------+---------------------------------+----------
         2 |        39         62         56 |       157
           |     45.88      54.39      58.33 |     53.22
-----------+---------------------------------+----------
     Total |        85        114         96 |       295
           |    100.00     100.00     100.00 |    100.00

          Pearson chi2(2) =   2.9087   Pr = 0.234

. tabulate Bastary_Satus DAQS_Tertile , chi2 column

+-------------------+
| Key               |
|-------------------|
|     frequency     |
| column percentage |
```

```
+-------------------+

      1: |
  general, |          DAQS_Tertile
    2:icu |     0.00      1.00      2.00 |     Total
-----------+--------------------------------+----------
      0.0 |       41        71        79 |       191
          |    48.24     62.28     82.29 |     64.75
-----------+--------------------------------+----------
   General |       44        43        17 |       104
          |    51.76     37.72     17.71 |     35.25
-----------+--------------------------------+----------
    Total |       85       114        96 |       295
          |   100.00    100.00    100.00 |    100.00

         Pearson chi2(2) =  23.4026   Pr = 0.000

. oneway kcal DAQS_Tertile , tabulate

DAQS_Tertil |          Summary of kcal
         e |       Mean   Std. dev.       Freq.
------------+------------------------------------
      0.00 |    1723.47     696.11         85
      1.00 |    1526.36    1035.29        114
      2.00 |    2183.36     764.69         96
------------+------------------------------------
     Total |    1796.95     904.47        295

                        Analysis of variance
```

```
   Source             SS        df      MS           F     Prob > F
------------------------------------------------------------------------
Between groups      23139917.8      2   11569958.9     15.54     0.0000
 Within groups      217370180    292   744418.426
------------------------------------------------------------------------
   Total          240510098    294   818061.558

Bartlett's equal-variances test: chi2(2) =  17.7281    Prob>chi2 = 0.000

. oneway protein DAQS_Tertile , tabulate

DAQS_Tertil |         Summary of protein
         e |       Mean   Std. dev.       Freq.
------------+-----------------------------------
      0.00 |      95.42      74.68         85
      1.00 |     138.52      98.68        114
      2.00 |     107.68      70.73         96
------------+-----------------------------------
     Total |     116.06      85.41        295

                        Analysis of variance
   Source             SS        df      MS           F     Prob > F
------------------------------------------------------------------------
Between groups      100477.818      2   50238.9091      7.18     0.0009
 Within groups     2044203.63    292   7000.69738
------------------------------------------------------------------------
   Total          2144681.45    294   7294.83487

Bartlett's equal-variances test: chi2(2) =  13.6499    Prob>chi2 = 0.001
```

```
. oneway tfat DAQS_Tertile , tabulate

DAQS_Tertil |          Summary of tfat
          e |        Mean   Std. dev.       Freq.
------------+------------------------------------
       0.00 |       88.30       67.89          85
       1.00 |      131.90      100.08         114
       2.00 |       99.36       77.71          96
------------+------------------------------------
      Total |      108.75       86.46         295

                        Analysis of variance
    Source              SS         df      MS            F     Prob > F
------------------------------------------------------------------------
Between groups      105092.629      2   52546.3146      7.33     0.0008
 Within groups      2092519.91    292   7166.16407
------------------------------------------------------------------------
    Total           2197612.54    294   7474.87258

Bartlett's equal-variances test: chi2(2) =  15.4434    Prob>chi2 = 0.000

. oneway sfat DAQS_Tertile , tabulate

DAQS_Tertil |          Summary of sfat
          e |        Mean   Std. dev.       Freq.
------------+------------------------------------
       0.00 |       22.42       10.56          85
       1.00 |       25.31       10.61         114
```

```
      2.00 |      26.25      10.89         96
------------+------------------------------------
     Total |      24.78      10.76        295

                  Analysis of variance
   Source            SS         df      MS            F     Prob > F
------------------------------------------------------------------------
Between groups      713.197411      2   356.598706      3.12     0.0455
 Within groups      33347.8224    292   114.204871
------------------------------------------------------------------------
   Total          34061.0199    294   115.853809

Bartlett's equal-variances test: chi2(2) =   0.1026    Prob>chi2 = 0.950

. oneway msfat DAQS_Tertile , tabulate

DAQS_Tertil |         Summary of msfat
         e |       Mean   Std. dev.       Freq.
------------+------------------------------------
      0.00 |      20.08       7.40         85
      1.00 |      22.22      10.91        114
      2.00 |      27.57       9.54         96
------------+-----------------------------------
     Total |      23.35      10.01        295

                  Analysis of variance
   Source            SS         df      MS            F     Prob > F
------------------------------------------------------------------------
Between groups      2759.32038      2   1379.66019     15.09     0.0000
```

```
 Within groups      26697.9818    292   91.4314445
------------------------------------------------------------------------
    Total          29457.3022    294   100.194905

Bartlett's equal-variances test: chi2(2) =  13.5775    Prob>chi2 = 0.001

. oneway linoleic DAQS_Tertile , tabulate

DAQS_Tertil |        Summary of linoleic
          e |        Mean   Std. dev.       Freq.
------------+------------------------------------
       0.00 |        9.19       4.77          85
       1.00 |        9.69       8.12         114
       2.00 |       13.38       7.54          96
------------+------------------------------------
      Total |       10.75       7.32         295

                        Analysis of variance
    Source              SS         df      MS            F     Prob > F
------------------------------------------------------------------------
Between groups      998.092276      2   499.046138      9.88     0.0001
 Within groups      14750.2051    292   50.5144011
------------------------------------------------------------------------
    Total          15748.2974    294   53.5656374

Bartlett's equal-variances test: chi2(2) =  25.8463    Prob>chi2 = 0.000

. oneway epa DAQS_Tertile , tabulate
```

```
DAQS_Tertil |          Summary of epa
          e |        Mean   Std. dev.       Freq.
------------+------------------------------------
       0.00 |        0.03        0.04          85
       1.00 |        0.04        0.07         114
       2.00 |        0.04        0.05          96
------------+------------------------------------
      Total |        0.04        0.06         295

                        Analysis of variance
    Source              SS         df      MS            F     Prob > F
------------------------------------------------------------------------
Between groups      .013340347      2   .006670173      2.08     0.1265
 Within groups      .935307507    292   .003203108
------------------------------------------------------------------------
    Total           .948647854    294   .003226693

Bartlett's equal-variances test: chi2(2) =  26.7569    Prob>chi2 = 0.000

. oneway calcium DAQS_Tertile , tabulate

DAQS_Tertil |        Summary of calcium
          e |        Mean   Std. dev.       Freq.
------------+------------------------------------
       0.00 |      841.77      397.58          85
       1.00 |      773.24      579.42         114
       2.00 |     1263.01      629.10          96
------------+------------------------------------
      Total |      952.37      591.25         295
```

```
                    Analysis of variance
   Source             SS         df      MS            F     Prob > F
------------------------------------------------------------------------
Between groups      13961166.6      2   6980583.31     22.95     0.0000
 Within groups      88813866.8    292   304157.078
------------------------------------------------------------------------
   Total           102775033    294   349574.944

Bartlett's equal-variances test: chi2(2) =  18.7189    Prob>chi2 = 0.000

. oneway iron DAQS_Tertile , tabulate

DAQS_Tertil |          Summary of iron
          e |        Mean   Std. dev.       Freq.
------------+-----------------------------------
       0.00 |       18.44       12.74          85
       1.00 |       17.92       15.37         114
       2.00 |       34.95       33.58          96
------------+-----------------------------------
      Total |       23.61       23.75         295

                    Analysis of variance
   Source             SS         df      MS            F     Prob > F
------------------------------------------------------------------------
Between groups      18302.5901      2   9151.29506     18.12     0.0000
 Within groups      147479.483    292   505.066723
------------------------------------------------------------------------
   Total           165782.073    294   563.884603
```

Bartlett's equal-variances test: chi2(2) = 104.3942   Prob>chi2 = 0.000

. oneway mg1 DAQS_Tertile , tabulate

DAQS_Tertile	Summary of mg1		
	Mean	Std. dev.	Freq.
0.00	322.36	131.11	85
1.00	287.75	204.32	114
2.00	477.15	189.73	96
Total	359.36	198.75	295

Analysis of variance

Source	SS	df	MS	F	Prob > F
Between groups	2032932.17	2	1016466.09	30.98	0.0000
Within groups	9580685.91	292	32810.5682		
Total	11613618.1	294	39502.1023		

Bartlett's equal-variances test: chi2(2) = 18.2043   Prob>chi2 = 0.000

. oneway zinc DAQS_Tertile , tabulate

DAQS_Tertile	Summary of zinc		
	Mean	Std. dev.	Freq.

```
      0.00 |       8.81       3.37         85
      1.00 |       8.71       4.77        114
      2.00 |      11.87       4.34         96
------------+------------------------------------
     Total |       9.77       4.49        295

                        Analysis of variance
    Source              SS         df      MS            F     Prob > F
------------------------------------------------------------------------
Between groups      629.716141      2   314.858071     17.32     0.0000
 Within groups      5307.89354    292   18.1777176
------------------------------------------------------------------------
    Total           5937.60968    294   20.1959513

Bartlett's equal-variances test: chi2(2) =  11.0340    Prob>chi2 = 0.004

. oneway mn DAQS_Tertile , tabulate

DAQS_Tertil |           Summary of mn
          e |       Mean   Std. dev.       Freq.
------------+------------------------------------
      0.00 |      18.02      31.38         85
      1.00 |      37.19      47.11        114
      2.00 |      20.31      34.03         96
------------+------------------------------------
     Total |      26.17      39.83        295

                        Analysis of variance
    Source              SS         df      MS            F     Prob > F
```

```
------------------------------------------------------------------------
Between groups      22774.0004      2   11387.0002      7.50     0.0007
 Within groups      443558.339    292   1519.03541
------------------------------------------------------------------------
    Total          466332.339    294   1586.16442

Bartlett's equal-variances test: chi2(2) =  19.2193    Prob>chi2 = 0.000

. oneway fluoride DAQS_Tertile , tabulate

DAQS_Tertil |        Summary of fluoride
          e |       Mean   Std. dev.       Freq.
------------+-----------------------------------
       0.00 |    2237.93    1428.41          85
       1.00 |    1727.45    1584.36         114
       2.00 |    2162.76    1221.49          96
------------+-----------------------------------
      Total |    2016.20    1443.41         295

                        Analysis of variance
    Source              SS         df      MS            F     Prob > F
------------------------------------------------------------------------
Between groups      15746348.3      2   7873174.14      3.85     0.0223
 Within groups       596785091    292   2043784.56
------------------------------------------------------------------------
    Total            612531440    294   2083440.27

Bartlett's equal-variances test: chi2(2) =   6.7744    Prob>chi2 = 0.034
```

```
. oneway vtarae DAQS_Tertile , tabulate

DAQS_Tertil |         Summary of vtarae
        e |        Mean   Std. dev.       Freq.
------------+------------------------------------
      0.00 |      996.28     1824.01         85
      1.00 |     1928.79     2754.73        114
      2.00 |     1458.60     2115.46         96
------------+------------------------------------
     Total |     1507.09     2336.34        295

                      Analysis of variance
    Source              SS         df      MS            F     Prob > F
------------------------------------------------------------------------
Between groups      42677401.7      2   21338700.8      3.99     0.0195
 Within groups      1.5621e+09    292   5349710.42
------------------------------------------------------------------------
    Total          1.6048e+09    294   5458479.06

Bartlett's equal-variances test: chi2(2) =  17.2700    Prob>chi2 = 0.000

. oneway vitemg DAQS_Tertile , tabulate

DAQS_Tertil |         Summary of vitemg
        e |        Mean   Std. dev.       Freq.
------------+------------------------------------
      0.00 |       8.93       2.31          85
      1.00 |      10.11       3.93         114
      2.00 |      13.57       3.81          96
```

```
------------+-----------------------------------
      Total |      10.90       3.98        295

                        Analysis of variance
    Source              SS         df      MS            F     Prob > F
------------------------------------------------------------------------
Between groups      1085.65789      2   542.828946     44.43     0.0000
 Within groups      3567.89143    292   12.2188063
------------------------------------------------------------------------
    Total           4653.54933    294   15.8283991

Bartlett's equal-variances test: chi2(2) =  27.0607    Prob>chi2 = 0.000

. oneway thiamin DAQS_Tertile , tabulate

DAQS_Tertil |        Summary of thiamin
          e |      Mean   Std. dev.       Freq.
------------+-----------------------------------
       0.00 |       1.72       0.46         85
       1.00 |       1.85       0.48        114
       2.00 |       2.09       0.52         96
------------+-----------------------------------
      Total |       1.89       0.51        295

                        Analysis of variance
    Source              SS         df      MS            F     Prob > F
------------------------------------------------------------------------
Between groups      6.27021287      2   3.13510643     13.27     0.0000
 Within groups      68.9748316    292   .236215177
```

```
---------------------------------------------------------------------
    Total          75.2450444    294   .255935525

Bartlett's equal-variances test: chi2(2) =   1.4127    Prob>chi2 = 0.493

. oneway vitb6 DAQS_Tertile , tabulate

DAQS_Tertil |          Summary of vitb6
          e |        Mean   Std. dev.       Freq.
------------+-----------------------------------
       0.00 |       53.46     153.94         85
       1.00 |      157.09      240.47        114
       2.00 |       52.02     168.49         96
------------+-----------------------------------
      Total |       93.04     201.90        295

                      Analysis of variance
    Source             SS         df      MS           F     Prob > F
---------------------------------------------------------------------
Between groups       762293.89      2   381146.945      9.92     0.0001
 Within groups      11222002.4    292   38431.5151
---------------------------------------------------------------------
    Total          11984296.3    294   40762.9126

Bartlett's equal-variances test: chi2(2) =  23.1087    Prob>chi2 = 0.000

. oneway vitc DAQS_Tertile , tabulate

DAQS_Tertil |          Summary of vitc
```

e	Mean	Std. dev.	Freq.
0.00	224.93	362.65	85
1.00	468.38	556.80	114
2.00	295.80	346.31	96
Total	342.07	454.25	295

Analysis of variance

Source	SS	df	MS	F	Prob > F
Between groups	3190811.28	2	1595405.64	8.11	0.0004
Within groups	57473293.7	292	196826.348		
Total	60664105	294	206340.493		

Bartlett's equal-variances test: chi2(2) = 29.4591 Prob>chi2 = 0.000

. oneway vitk DAQS_Tertile , tabulate

DAQS_Tertil e	Summary of vitk Mean	Std. dev.	Freq.
0.00	326.07	387.47	85
1.00	598.36	543.55	114
2.00	608.76	509.90	96
Total	523.29	506.24	295

```
                    Analysis of variance
   Source              SS         df      MS            F     Prob > F
------------------------------------------------------------------------
Between groups      4649930.56      2   2324965.28      9.60     0.0001
 Within groups      70696472.8    292   242111.208
------------------------------------------------------------------------
   Total            75346403.3    294   256280.283

Bartlett's equal-variances test: chi2(2) =  10.8236    Prob>chi2 = 0.004

. oneway sfiber DAQS_Tertile , tabulate

DAQS_Tertil |          Summary of sfiber
          e |        Mean   Std. dev.       Freq.
------------+------------------------------------
       0.00 |        0.55        0.59          85
       1.00 |        0.93        0.90         114
       2.00 |        0.76        0.94          96
------------+------------------------------------
      Total |        0.76        0.85         295

                    Analysis of variance
   Source              SS         df      MS            F     Prob > F
------------------------------------------------------------------------
Between groups      6.98297127      2   3.49148563      4.98     0.0075
 Within groups      204.752812    292   .701208261
------------------------------------------------------------------------
   Total            211.735783    294   .720189739
```

```
Bartlett's equal-variances test: chi2(2) =  19.9698    Prob>chi2 = 0.000

. oneway cfiber DAQS_Tertile , tabulate

DAQS_Tertil |         Summary of cfiber
         e |        Mean   Std. dev.       Freq.
------------+------------------------------------
      0.00 |       19.78       31.79          85
      1.00 |       37.80       44.35         114
      2.00 |       22.87       31.02          96
------------+------------------------------------
     Total |       27.75       37.69         295

                        Analysis of variance
    Source              SS         df       MS            F     Prob > F
------------------------------------------------------------------------
Between groups      19196.9635      2   9598.48175      7.03     0.0010
 Within groups      398547.633    292   1364.88916
------------------------------------------------------------------------
    Total           417744.597    294   1420.89999

Bartlett's equal-variances test: chi2(2) =  17.0877    Prob>chi2 = 0.000

. oneway glucose DAQS_Tertile , tabulate

DAQS_Tertil |        Summary of glucose
         e |        Mean   Std. dev.       Freq.
------------+------------------------------------
      0.00 |       12.14        6.24          85
```

```
       1.00 |       11.89        8.46         114
       2.00 |       18.27        7.89          96
------------+------------------------------------
      Total |       14.04        8.21         295

                        Analysis of variance
    Source              SS         df      MS            F     Prob > F
------------------------------------------------------------------------
Between groups       2551.3479      2   1275.67395     21.58     0.0000
 Within groups      17264.8038    292   59.1260403
------------------------------------------------------------------------
    Total           19816.1517    294   67.4018764

Bartlett's equal-variances test: chi2(2) =   8.6465    Prob>chi2 = 0.013

. oneway fructose DAQS_Tertile , tabulate

DAQS_Tertil |         Summary of fructose
          e |        Mean   Std. dev.       Freq.
------------+------------------------------------
       0.00 |       16.84        6.42          85
       1.00 |       19.76        7.65         114
       2.00 |       23.40        9.68          96
------------+------------------------------------
      Total |       20.10        8.44         295

                        Analysis of variance
    Source              SS         df      MS            F     Prob > F
------------------------------------------------------------------------
```

Between groups 1958.20647 2 979.103236 15.07 0.0000

Within groups 18967.4391 292 64.9569832

Total 20925.6456 294 71.1756651

Bartlett's equal-variances test: chi2(2) = 15.3114 Prob>chi2 = 0.000

. oneway lactose DAQS_Tertile , tabulate

DAQS_Tertile	Summary of lactose		
	Mean	Std. dev.	Freq.
0.00	9.14	6.39	85
1.00	9.27	9.74	114
2.00	12.94	9.43	96
Total	10.43	8.94	295

Analysis of variance

Source	SS	df	MS	F	Prob > F
Between groups	898.847774	2	449.423887	5.81	0.0034
Within groups	22595.7765	292	77.3827964		
Total	23494.6243	294	79.9136881		

Bartlett's equal-variances test: chi2(2) = 17.4098 Prob>chi2 = 0.000

. oneway msfat DAQS_Tertile , tabulate

```
DAQS_Tertil |         Summary of msfat
          e |        Mean   Std. dev.       Freq.
------------+------------------------------------
       0.00 |       20.08       7.40          85
       1.00 |       22.22      10.91         114
       2.00 |       27.57       9.54          96
------------+------------------------------------
      Total |       23.35      10.01         295

                        Analysis of variance
    Source              SS         df      MS            F     Prob > F
------------------------------------------------------------------------
Between groups      2759.32038      2   1379.66019     15.09     0.0000
 Within groups      26697.9818    292   91.4314445
------------------------------------------------------------------------
    Total           29457.3022    294   100.194905

Bartlett's equal-variances test: chi2(2) =  13.5775    Prob>chi2 = 0.001

. oneway oleic DAQS_Tertile , tabulate

DAQS_Tertil |         Summary of oleic
          e |        Mean   Std. dev.       Freq.
------------+------------------------------------
       0.00 |       17.53       6.98          85
       1.00 |       19.60      10.39         114
       2.00 |       24.80       9.15          96
------------+------------------------------------
```

Total	20.70	9.56	295

Analysis of variance

Source	SS	df	MS	F	Prob > F
Between groups	2609.92266	2	1304.96133	15.72	0.0000
Within groups	24244.3815	292	83.0287037		
Total	26854.3041	294	91.3411705		

Bartlett's equal-variances test: chi2(2) = 14.2455 Prob>chi2 = 0.001

. oneway sodium DAQS_Tertile , tabulate

DAQS_Tertile	Summary of sodium Mean	Std. dev.	Freq.
0.00	3602.70	1059.98	85
1.00	3712.04	1006.70	114
2.00	3923.72	944.21	96
Total	3749.42	1007.62	295

Analysis of variance

Source	SS	df	MS	F	Prob > F
Between groups	4905582.84	2	2452791.42	2.44	0.0890
Within groups	293592408	292	1005453.45		

```
   Total          298497991    294   1015299.29

Bartlett's equal-variances test: chi2(2) =   1.1959    Prob>chi2 = 0.550

. oneway phospho DAQS_Tertile , tabulate

DAQS_Tertil |        Summary of phospho
         e |        Mean   Std. dev.       Freq.
------------+------------------------------------
      0.00 |     1334.88     282.81         85
      1.00 |     1438.66     338.23        114
      2.00 |     1669.96     366.45         91
------------+------------------------------------
     Total |     1480.82     357.81        290

                        Analysis of variance
    Source              SS         df      MS            F     Prob > F
------------------------------------------------------------------------
Between groups      5268422.53      2   2634211.27     23.83     0.0000
 Within groups      31731510.3    287   110562.754
------------------------------------------------------------------------
    Total           36999932.9    289   128027.449

Bartlett's equal-variances test: chi2(2) =   5.8190    Prob>chi2 = 0.055

. oneway copper DAQS_Tertile , tabulate

DAQS_Tertil |         Summary of copper
         e |        Mean   Std. dev.       Freq.
```

```
------------+------------------------------------
      0.00 |        2.19        2.10          85
      1.00 |        3.72        3.56         114
      2.00 |        2.71        2.63          96
------------+------------------------------------
     Total |        2.95        2.96         295

                        Analysis of variance
    Source              SS         df      MS            F     Prob > F
------------------------------------------------------------------------
Between groups      121.684575      2   60.8422877      7.22     0.0009
 Within groups      2459.28839    292   8.42222051
------------------------------------------------------------------------
    Total           2580.97297    294   8.77881961

Bartlett's equal-variances test: chi2(2) =  26.7984    Prob>chi2 = 0.000

. oneway selenium DAQS_Tertile , tabulate

DAQS_Tertil |         Summary of selenium
          e |        Mean   Std. dev.       Freq.
------------+------------------------------------
      0.00 |      292.23      600.11          85
      1.00 |      789.66     1170.04         114
      2.00 |      411.19     1042.06          96
------------+------------------------------------
     Total |      523.17     1013.41         295

                        Analysis of variance
```

```
   Source            SS        df      MS          F     Prob > F
---------------------------------------------------------------------
Between groups      13833036.1      2   6916518.05      7.01     0.0011
 Within groups      288106298    292   986665.403
---------------------------------------------------------------------
   Total          301939334    294   1027004.54

Bartlett's equal-variances test: chi2(2) =  38.3715    Prob>chi2 = 0.000

. oneway riboflav DAQS_Tertile , tabulate

DAQS_Tertil |        Summary of riboflav
         e |       Mean   Std. dev.       Freq.
------------+-----------------------------------
      0.00 |       3.74       6.37          85
      1.00 |       8.17       9.90         114
      2.00 |       4.18       6.54          96
------------+-----------------------------------
     Total |       5.60       8.20         295

                     Analysis of variance
   Source            SS        df      MS          F     Prob > F
---------------------------------------------------------------------
Between groups      1241.77662      2   620.888309      9.78     0.0001
 Within groups      18533.7189    292   63.4716399
---------------------------------------------------------------------
   Total          19775.4955    294    67.26359

Bartlett's equal-variances test: chi2(2) =  26.0498    Prob>chi2 = 0.000
```

. oneway vitb12 DAQS_Tertile , tabulate

```
DAQS_Tertil |         Summary of vitb12
          e |       Mean   Std. dev.      Freq.
------------+-----------------------------------
       0.00 |       5.25       7.23         85
       1.00 |      10.36      11.32        114
       2.00 |       5.77       9.07         96
------------+------------------------------------
      Total |       7.40       9.82        295

                    Analysis of variance
    Source              SS         df      MS            F     Prob > F
------------------------------------------------------------------------
Between groups      1647.21145      2   823.605724      9.01     0.0002
 Within groups      26685.2319    292   91.3877804
------------------------------------------------------------------------
    Total           28332.4433    294   96.3688548

Bartlett's equal-variances test: chi2(2) =  18.5562    Prob>chi2 = 0.000
```

. oneway biotin DAQS_Tertile , tabulate

```
DAQS_Tertil |         Summary of biotin
          e |       Mean   Std. dev.      Freq.
------------+-----------------------------------
       0.00 |      23.60      10.19         85
       1.00 |      22.04      14.96        114
```

```
      2.00 |      36.67      16.17         96
------------+------------------------------------
     Total |      27.25      15.60        295

                        Analysis of variance
    Source              SS         df      MS            F     Prob > F
------------------------------------------------------------------------
Between groups      12744.0408      2   6372.02042     31.62     0.0000
 Within groups      58839.8841    292   201.506453
------------------------------------------------------------------------
    Total           71583.925    294   243.482738

Bartlett's equal-variances test: chi2(2) =  19.0997    Prob>chi2 = 0.000

. oneway vitD DAQS_Tertile , tabulate

DAQS_Tertil |         Summary of vit D
          e |       Mean   Std. dev.       Freq.
------------+------------------------------------
      0.00 |       24.9       11.4         85
      1.00 |       31.5       12.6        114
      2.00 |       27.9       11.4         96
------------+------------------------------------
     Total |       28.4       12.1        295

                        Analysis of variance
    Source              SS         df      MS            F     Prob > F
------------------------------------------------------------------------
Between groups      2203.97724      2   1101.98862      7.86     0.0005
```

```
 Within groups      40936.0008    292   140.191784
---------------------------------------------------------------------
    Total          43139.9781    294   146.734619

Bartlett's equal-variances test: chi2(2) =   1.4066    Prob>chi2 = 0.495

. oneway tfiber DAQS_Tertile , tabulate

DAQS_Tertil |         Summary of tfiber
          e |        Mean   Std. dev.       Freq.
------------+-----------------------------------
       0.00 |       32.39       17.72          85
       1.00 |       26.40       20.30         114
       2.00 |       43.17       19.05          96
------------+-----------------------------------
      Total |       33.58       20.39         295

                        Analysis of variance
    Source              SS         df      MS            F     Prob > F
---------------------------------------------------------------------
Between groups      14826.4344      2   7413.21719     20.15     0.0000
 Within groups       107429.09    292   367.907842
---------------------------------------------------------------------
    Total          122255.524    294   415.835116

Bartlett's equal-variances test: chi2(2) =   1.7682    Prob>chi2 = 0.413

. oneway ifiber DAQS_Tertile , tabulate
```

```
DAQS_Tertil |        Summary of ifiber
        e |      Mean   Std. dev.       Freq.
------------+------------------------------------
     0.00 |      2.85      3.00         85
     1.00 |      4.85      4.36        114
     2.00 |      4.00      2.93         96
------------+------------------------------------
    Total |      3.99      3.65        295

                        Analysis of variance
    Source              SS         df      MS            F     Prob > F
------------------------------------------------------------------------
Between groups      194.554866      2    97.277433      7.64     0.0006
 Within groups      3717.10892    292   12.7298251
------------------------------------------------------------------------
    Total          3911.66379    294   13.3049789

Bartlett's equal-variances test: chi2(2) =  21.2479    Prob>chi2 = 0.000

. oneway tsogar DAQS_Tertile , tabulate

DAQS_Tertil |        Summary of tsogar
        e |      Mean   Std. dev.       Freq.
------------+------------------------------------
     0.00 |     84.00      35.28         85
     1.00 |     80.28      51.05        114
     2.00 |    119.74      45.79         96
------------+------------------------------------
    Total |     94.19      48.51        295
```

Analysis of variance

Source	SS	df	MS	F	Prob > F
Between groups	93570.6421	2	46785.321	22.83	0.0000
Within groups	598301.636	292	2048.97821		
Total	691872.279	294	2353.30707		

Bartlett's equal-variances test: chi2(2) = 12.4179 Prob>chi2 = 0.002

. oneway galactos DAQS_Tertile , tabulate

DAQS_Tertile	Summary of galactos Mean	Std. dev.	Freq.
0.00	4.32	6.87	85
1.00	7.41	8.73	114
2.00	3.90	5.60	96
Total	5.38	7.45	295

Analysis of variance

Source	SS	df	MS	F	Prob > F
Between groups	775.667538	2	387.833769	7.28	0.0008
Within groups	15560.8702	292	53.2906514		
Total	16336.5377	294	55.5664549		

```
Bartlett's equal-variances test: chi2(2) =  19.8634    Prob>chi2 = 0.000

. oneway sucrose DAQS_Tertile , tabulate

DAQS_Tertil |        Summary of sucrose
          e |       Mean   Std. dev.       Freq.
------------+------------------------------------
       0.00 |      17.43       8.36          85
       1.00 |      17.07       8.91         114
       2.00 |      21.29       9.21          96
------------+------------------------------------
      Total |      18.55       9.03         295

                        Analysis of variance
    Source              SS         df      MS            F     Prob > F
------------------------------------------------------------------------
Between groups      1077.36564      2   538.682821      6.87     0.0012
 Within groups      22904.0434    292   78.4385047
------------------------------------------------------------------------
    Total            23981.409    294   81.5694184

Bartlett's equal-variances test: chi2(2) =   0.8361    Prob>chi2 = 0.658

. oneway maltose DAQS_Tertile , tabulate

DAQS_Tertil |        Summary of maltose
          e |       Mean   Std. dev.       Freq.
------------+------------------------------------
```

0.00	1.49	0.76	75
1.00	1.56	0.85	79
2.00	1.65	0.78	88
Total	1.57	0.80	242

Analysis of variance

Source	SS	df	MS	F	Prob > F
Between groups	1.03929308	2	.519646542	0.81	0.4448
Within groups	152.801131	239	.639335275		
Total	153.840424	241	.638342007		

Bartlett's equal-variances test: chi2(2) = 1.1444 Prob>chi2 = 0.564

. oneway acarote DAQS_Tertile , tabulate

DAQS_Tertile	Summary of acarote Mean	Std. dev.	Freq.
0.00	917.90	991.30	85
1.00	1427.92	1494.01	114
2.00	1868.11	1137.13	96
Total	1424.21	1301.91	295

Analysis of variance

Source	SS	df	MS	F	Prob > F

```
------------------------------------------------------------------------
Between groups      40707191.8      2   20353595.9     12.99     0.0000
 Within groups       457612078    292   1567164.65
------------------------------------------------------------------------
    Total            498319270    294    1694963.5

Bartlett's equal-variances test: chi2(2) =  17.4244    Prob>chi2 = 0.000

. oneway bcarote DAQS_Tertile , tabulate

DAQS_Tertil |        Summary of bcarote
          e |       Mean   Std. dev.       Freq.
------------+-----------------------------------
       0.00 |    3036.20    1758.83          85
       1.00 |    3196.95    2273.42         114
       2.00 |    7229.02    4391.78          96
------------------------------------------------------------
      Total |    4462.76    3579.26         295

                        Analysis of variance
    Source              SS         df      MS            F     Prob > F
------------------------------------------------------------------------
Between groups      1.0903e+09      2    545125624     59.48     0.0000
 Within groups      2.6762e+09    292   9165131.67
------------------------------------------------------------------------
    Total           3.7665e+09    294   12811121.4

Bartlett's equal-variances test: chi2(2) =  84.8837    Prob>chi2 = 0.000
```

```
.

. ## TABLE 3 univariable

Unknown #command

.

. logistic Bastary_Satus Age


Logistic regression                         Number of obs =    295

                                   LR chi2(1)    =  10.40

                                   Prob > chi2   = 0.0013

Log likelihood = -186.25697                      Pseudo R2     = 0.0272


------------------------------------------------------------------------------

Bastary_Satus | Odds ratio   Std. err.      z    P>|z|     [95% conf. interval]

--------------+---------------------------------------------------------------

          Age |   1.027065   .0087385     3.14   0.002     1.01008    1.044336

        _cons |   .1110422   .0587331    -4.16   0.000     .0393791    .3131197

------------------------------------------------------------------------------

Note: _cons estimates baseline odds.


. logistic Bastary_Satus Wieght


Logistic regression                         Number of obs =    295

                                   LR chi2(1)    =   0.67

                                   Prob > chi2   = 0.4114

Log likelihood = -191.11948                      Pseudo R2     = 0.0018


------------------------------------------------------------------------------

Bastary_Satus | Odds ratio   Std. err.      z    P>|z|     [95% conf. interval]

--------------+---------------------------------------------------------------
```

```
      Wieght |    1.00786   .0096138     0.82   0.412     .9891923     1.02688
       _cons |   .2983104   .2222243    -1.62   0.104     .0692742    1.284592
------------------------------------------------------------------------------
Note: _cons estimates baseline odds.

. logistic Bastary_Satus BMI

Logistic regression                                     Number of obs =    295
                                                        LR chi2(1)    =   2.61
                                                        Prob > chi2   = 0.1063
Log likelihood = -190.15303                             Pseudo R2     = 0.0068

------------------------------------------------------------------------------
Bastary_Satus | Odds ratio   Std. err.      z    P>|z|     [95% conf. interval]
--------------+----------------------------------------------------------------
          BMI |   1.046249    .029372     1.61   0.107     .9902359     1.10543
        _cons |   .1544177   .1227714    -2.35   0.019     .0325043     .733591
------------------------------------------------------------------------------
Note: _cons estimates baseline odds.

. logistic Bastary_Satus Sex

Logistic regression                                     Number of obs =    295
                                                        LR chi2(1)    =   0.42
                                                        Prob > chi2   = 0.5171
Log likelihood = -191.24699                             Pseudo R2     = 0.0011

------------------------------------------------------------------------------
Bastary_Satus | Odds ratio   Std. err.      z    P>|z|     [95% conf. interval]
```

```
--------------+----------------------------------------------------------------
         Sex |   1.171717   .2868939     0.65   0.517     .7251149    1.893384
       _cons |   .4267241   .1694998    -2.14   0.032     .1959025    .9295108
-------------------------------------------------------------------------------
Note: _cons estimates baseline odds.

. logistic Bastary_Satus i.DAQS_Tertile

Logistic regression                                     Number of obs =    295
                                                        LR chi2(2)    =  24.44
                                                        Prob > chi2   = 0.0000
Log likelihood = -179.23572                             Pseudo R2     = 0.0638

-------------------------------------------------------------------------------
Bastary_Satus | Odds ratio   Std. err.      z    P>|z|     [95% conf. interval]
--------------+----------------------------------------------------------------
 DAQS_Tertile |
          1  |   .5643406   .1640066    -1.97   0.049     .3192774    .9975033
          2  |   .2005178   .0690548    -4.67   0.000     .1020973    .3938147
             |
       _cons |   1.073171   .2329486     0.33   0.745     .7012973    1.642236
-------------------------------------------------------------------------------
Note: _cons estimates baseline odds.

. logistic Bastary_Satus kcal

Logistic regression                                     Number of obs =    295
                                                        LR chi2(1)    =   1.44
                                                        Prob > chi2   = 0.2308
```

```
Log likelihood = -190.73899                          Pseudo R2     = 0.0037

------------------------------------------------------------------------------
Bastary_Satus | Odds ratio   Std. err.      z    P>|z|     [95% conf. interval]
--------------+----------------------------------------------------------------
         kcal |   .9998398   .0001333    -1.20   0.230     .9995785    1.000101
        _cons |   .7240724   .1917307    -1.22   0.223     .4309115    1.216678
------------------------------------------------------------------------------
Note: _cons estimates baseline odds.

. logistic Bastary_Satus protein

Logistic regression                             Number of obs =    295
                                       LR chi2(1)    =   0.28
                                       Prob > chi2   = 0.5983
Log likelihood = -191.31804                         Pseudo R2     = 0.0007

------------------------------------------------------------------------------
Bastary_Satus | Odds ratio   Std. err.      z    P>|z|     [95% conf. interval]
--------------+----------------------------------------------------------------
      protein |   1.000745   .0014084     0.53   0.597     .9979886     1.00351
        _cons |   .4991172   .1025635    -3.38   0.001     .3336486    .7466478
------------------------------------------------------------------------------
Note: _cons estimates baseline odds.

. logistic Bastary_Satus tfat

Logistic regression                             Number of obs =    295
                                       LR chi2(1)    =   0.33
```

Prob > chi2 = 0.5654

Log likelihood = -191.29166 Pseudo R2 = 0.0009

```
------------------------------------------------------------------------------
Bastary_Satus | Odds ratio   Std. err.      z    P>|z|     [95% conf. interval]
--------------+----------------------------------------------------------------
         tfat |   1.000801   .0013857     0.58   0.563     .9980885     1.00352
        _cons |   .4987909   .0974781    -3.56   0.000     .3400707    .7315901
------------------------------------------------------------------------------
```

Note: _cons estimates baseline odds.

. logistic Bastary_Satus sfat

Logistic regression Number of obs = 295

LR chi2(1) = 1.73

Prob > chi2 = 0.1888

Log likelihood = -190.59319 Pseudo R2 = 0.0045

```
------------------------------------------------------------------------------
Bastary_Satus | Odds ratio   Std. err.      z    P>|z|     [95% conf. interval]
--------------+----------------------------------------------------------------
         sfat |   1.014834   .0113281     1.32   0.187     .9928726    1.037282
        _cons |   .3767213    .115646    -3.18   0.001      .206404    .6875783
------------------------------------------------------------------------------
```

Note: _cons estimates baseline odds.

. logistic Bastary_Satus msfat

Logistic regression Number of obs = 295

LR chi2(1) = 0.22

Prob > chi2 = 0.6411

Log likelihood = -191.3482 Pseudo R2 = 0.0006

Bastary_Satus	Odds ratio	Std. err.	z	P>|z|	[95% conf. interval]	
msfat	.9942982	.0122358	-0.46	0.642	.9706033	1.018572
_cons	.6219604	.1928859	-1.53	0.126	.3386751	1.1422

Note: _cons estimates baseline odds.

. logistic Bastary_Satus linoleic

Logistic regression Number of obs = 295

LR chi2(1) = 2.95

Prob > chi2 = 0.0857

Log likelihood = -189.98042 Pseudo R2 = 0.0077

Bastary_Satus	Odds ratio	Std. err.	z	P>|z|	[95% conf. interval]	
linoleic	.9708724	.0170425	-1.68	0.092	.9380377	1.004856
_cons	.7426685	.1615521	-1.37	0.171	.4848795	1.137512

Note: _cons estimates baseline odds.

. logistic Bastary_Satus epa

Logistic regression Number of obs = 295

LR chi2(1) = 0.80

Prob > chi2 = 0.3722

Log likelihood = -191.05866 Pseudo R2 = 0.0021

Bastary_Satus	Odds ratio	Std. err.	z	P>|z|	[95% conf.	interval]
epa	.1311594	.3091907	-0.86	0.389	.0012918	13.31659
_cons	.5872748	.08733	-3.58	0.000	.4387977	.7859923

Note: _cons estimates baseline odds.

. logistic Bastary_Satus calcium

Logistic regression Number of obs = 295

LR chi2(1) = 1.34

Prob > chi2 = 0.2478

Log likelihood = -190.78913 Pseudo R2 = 0.0035

Bastary_Satus	Odds ratio	Std. err.	z	P>|z|	[95% conf.	interval]
calcium	.9997594	.0002095	-1.15	0.251	.9993488	1.00017
_cons	.6827066	.1566771	-1.66	0.096	.4354	1.070483

Note: _cons estimates baseline odds.

. logistic Bastary_Satus iron

Logistic regression Number of obs = 295

LR chi2(1) = 2.94

Prob > chi2 = 0.0864

Log likelihood = -189.98645 Pseudo R2 = 0.0077

Bastary_Satus	Odds ratio	Std. err.	z	P>|z|	[95% conf. interval]	
iron	.9904476	.0058608	-1.62	0.105	.979027	1.002001
_cons	.6769082	.1200089	-2.20	0.028	.4782119	.9581623

Note: _cons estimates baseline odds.

. logistic Bastary_Satus mg1

Logistic regression Number of obs = 295

LR chi2(1) = 1.91

Prob > chi2 = 0.1673

Log likelihood = -190.50315 Pseudo R2 = 0.0050

Bastary_Satus	Odds ratio	Std. err.	z	P>|z|	[95% conf. interval]	
mg1	.9991529	.0006142	-1.38	0.168	.9979498	1.000357
_cons	.73537	.1819622	-1.24	0.214	.4527746	1.194345

Note: _cons estimates baseline odds.

. logistic Bastary_Satus zinc

Logistic regression Number of obs = 295

LR chi2(1) = 0.55

Prob > chi2 = 0.4582

Log likelihood = -191.18165 Pseudo R2 = 0.0014

Bastary_Satus	Odds ratio	Std. err.	z	P>|z|	[95% conf. interval]	
zinc	.9801017	.026535	-0.74	0.458	.9294498	1.033514
_cons	.6618471	.1908781	-1.43	0.152	.3760706	1.164786

Note: _cons estimates baseline odds.

. logistic Bastary_Satus mn

Logistic regression Number of obs = 295

LR chi2(1) = 0.54

Prob > chi2 = 0.4617

Log likelihood = -191.18594 Pseudo R2 = 0.0014

Bastary_Satus	Odds ratio	Std. err.	z	P>|z|	[95% conf. interval]	
mn	1.002225	.0030075	0.74	0.459	.9963476	1.008137
_cons	.5131929	.0752174	-4.55	0.000	.3850544	.6839734

Note: _cons estimates baseline odds.

. logistic Bastary_Satus fluoride

Logistic regression Number of obs = 295

LR chi2(1) = 0.00

Prob > chi2 = 0.9512

Log likelihood = -191.45498 Pseudo R2 = 0.0000

Bastary_Satus	Odds ratio	Std. err.	z	P>|z|	[95% conf. interval]	
fluoride	.9999948	.0000846	-0.06	0.951	.999829	1.000161
_cons	.5502097	.1152217	-2.85	0.004	.3649847	.829434

Note: _cons estimates baseline odds.

. logistic Bastary_Satus vtarae

Logistic regression Number of obs = 295

LR chi2(1) = 0.39

Prob > chi2 = 0.5348

Log likelihood = -191.26421 Pseudo R2 = 0.0010

Bastary_Satus	Odds ratio	Std. err.	z	P>|z|	[95% conf. interval]	
vtarae	1.000032	.0000509	0.63	0.532	.9999321	1.000132
_cons	.5186486	.0753052	-4.52	0.000	.3901961	.6893875

```
Note: _cons estimates baseline odds.

. logistic Bastary_Satus vitemg

Logistic regression                    Number of obs =    295
                                LR chi2(1)    =   1.26
                                Prob > chi2   = 0.2612
Log likelihood = -190.82562                  Pseudo R2     = 0.0033

-------------------------------------------------------------------------------
Bastary_Satus | Odds ratio   Std. err.      z    P>|z|     [95% conf. interval]
--------------+----------------------------------------------------------------
       vitemg |   .9654474   .0305907   -1.11   0.267    .9073146    1.027305
        _cons |   .7963355   .2874819   -0.63   0.528    .3924706     1.61579
-------------------------------------------------------------------------------
Note: _cons estimates baseline odds.

. logistic Bastary_Satus thiamin

Logistic regression                    Number of obs =    295
                                LR chi2(1)    =   0.68
                                Prob > chi2   = 0.4106
Log likelihood = -191.11832                  Pseudo R2     = 0.0018

-------------------------------------------------------------------------------
Bastary_Satus | Odds ratio   Std. err.      z    P>|z|     [95% conf. interval]
--------------+----------------------------------------------------------------
      thiamin |   .8190339   .1994458   -0.82   0.412    .5081862    1.320021
        _cons |   .7928418   .3746236   -0.49   0.623    .3140419    2.001638
```

Note: _cons estimates baseline odds.

. logistic Bastary_Satus vitb6

Logistic regression Number of obs = 295

LR chi2(1) = 0.32

Prob > chi2 = 0.5713

Log likelihood = -191.29656 Pseudo R2 = 0.0008

Bastary_Satus	Odds ratio	Std. err.	z	P>|z|	[95% conf. interval]	
vitb6	1.000339	.0005951	0.57	0.569	.9991729	1.001506
_cons	.5272795	.071095	-4.75	0.000	.4048277	.6867702

Note: _cons estimates baseline odds.

. logistic Bastary_Satus vitc

Logistic regression Number of obs = 295

LR chi2(1) = 1.30

Prob > chi2 = 0.2545

Log likelihood = -190.80751 Pseudo R2 = 0.0034

Bastary_Satus	Odds ratio	Std. err.	z	P>|z|	[95% conf. interval]	
vitc	1.0003	.0002614	1.15	0.251	.9997875	1.000812

_cons | .4902892 .0753027 -4.64 0.000 .3628422 .6625016

Note: _cons estimates baseline odds.

. logistic Bastary_Satus vitk

Logistic regression Number of obs = 295

LR chi2(1) = 0.24

Prob > chi2 = 0.6227

Log likelihood = -191.33578 Pseudo R2 = 0.0006

Bastary_Satus	Odds ratio	Std. err.	z	P>|z|	[95% conf. interval]	
vitk	.9998805	.0002439	-0.49	0.624	.9994025	1.000359
_cons	.5793353	.1013124	-3.12	0.002	.4112202	.8161793

Note: _cons estimates baseline odds.

. logistic Bastary_Satus sfiber

Logistic regression Number of obs = 295

LR chi2(1) = 0.01

Prob > chi2 = 0.9298

Log likelihood = -191.45297 Pseudo R2 = 0.0000

Bastary_Satus | Odds ratio Std. err. z P>|z| [95% conf. interval]

--------------+--

sfiber | .9873496 .1429748 -0.09 0.930 .7433808 1.311386

_cons | .5498108 .0903149 -3.64 0.000 .3984651 .7586409

Note: _cons estimates baseline odds.

. logistic Bastary_Satus cfiber

Logistic regression Number of obs = 295

LR chi2(1) = 0.09

Prob > chi2 = 0.7657

Log likelihood = -191.41245 Pseudo R2 = 0.0002

Bastary_Satus | Odds ratio Std. err. z P>|z| [95% conf. interval]

--------------+--

cfiber | 1.000958 .0032046 0.30 0.765 .9946968 1.007258

_cons | .5301299 .0803325 -4.19 0.000 .3939097 .7134572

Note: _cons estimates baseline odds.

. logistic Bastary_Satus glucose

Logistic regression Number of obs = 295

LR chi2(1) = 0.23

Prob > chi2 = 0.6290

Log likelihood = -191.34016 Pseudo R2 = 0.0006

Bastary_Satus | Odds ratio Std. err. z P>|z| [95% conf. interval]

Bastary_Satus	Odds ratio	Std. err.	z	P>|z|	[95% conf. interval]	
glucose	.9928293	.0148081	-0.48	0.629	.9642262	1.022281
_cons	.6020891	.1447886	-2.11	0.035	.3758083	.9646176

Note: _cons estimates baseline odds.

. logistic Bastary_Satus fructose

Logistic regression Number of obs = 295

LR chi2(1) = 0.01

Prob > chi2 = 0.9168

Log likelihood = -191.45139 Pseudo R2 = 0.0000

Bastary_Satus	Odds ratio	Std. err.	z	P>|z|	[95% conf. interval]	
fructose	.9984864	.0144858	-0.10	0.917	.9704947	1.027286
_cons	.5613253	.1771951	-1.83	0.067	.3023524	1.042115

Note: _cons estimates baseline odds.

. logistic Bastary_Satus lactose

Logistic regression Number of obs = 295

LR chi2(1) = 0.15

Prob > chi2 = 0.7016

Log likelihood = -191.38345 Pseudo R2 = 0.0004

```
Bastary_Satus | Odds ratio   Std. err.      z    P>|z|     [95% conf. interval]
--------------+----------------------------------------------------------------
      lactose |   1.005203   .0135691     0.38   0.701     .9789565    1.032152
        _cons |    .515662   .0966264    -3.53   0.000     .3571612    .7445023
-------------------------------------------------------------------------------
Note: _cons estimates baseline odds.

. logistic Bastary_Satus msfat

Logistic regression                             Number of obs =    295
                                                LR chi2(1)    =   0.22
                                                Prob > chi2   = 0.6411
Log likelihood = -191.3482                      Pseudo R2     = 0.0006

-------------------------------------------------------------------------------
Bastary_Satus | Odds ratio   Std. err.      z    P>|z|     [95% conf. interval]
--------------+----------------------------------------------------------------
        msfat |   .9942982   .0122358    -0.46   0.642     .9706033    1.018572
        _cons |   .6219604   .1928859    -1.53   0.126     .3386751      1.1422
-------------------------------------------------------------------------------
Note: _cons estimates baseline odds.

. logistic Bastary_Satus oleic

Logistic regression                             Number of obs =    295
                                                LR chi2(1)    =   0.38
                                                Prob > chi2   = 0.5400
Log likelihood = -191.26906                     Pseudo R2     = 0.0010
```

```
------------------------------------------------------------------------------
Bastary_Satus | Odds ratio   Std. err.      z    P>|z|     [95% conf. interval]
--------------+----------------------------------------------------------------
        oleic |   .9921383   .0128382    -0.61   0.542     .9672923    1.017622
        _cons |   .6405723   .1868313    -1.53   0.127      .361663    1.134572
------------------------------------------------------------------------------
Note: _cons estimates baseline odds.

. logistic Bastary_Satus sodium

Logistic regression                            Number of obs =    295
                                               LR chi2(1)    =   0.27
                                               Prob > chi2   = 0.6024
Log likelihood = -191.32114                    Pseudo R2     = 0.0007

------------------------------------------------------------------------------
Bastary_Satus | Odds ratio   Std. err.      z    P>|z|     [95% conf. interval]
--------------+----------------------------------------------------------------
       sodium |   .9999366    .000122    -0.52   0.603     .9996975    1.000176
        _cons |   .6901935    .325221    -0.79   0.431     .2740832    1.738038
------------------------------------------------------------------------------
Note: _cons estimates baseline odds.

. logistic Bastary_Satus phospho

Logistic regression                            Number of obs =    290
                                               LR chi2(1)    =   0.01
                                               Prob > chi2   = 0.9097
Log likelihood = -188.06072                    Pseudo R2     = 0.0000
```

```
------------------------------------------------------------------------------
Bastary_Satus | Odds ratio   Std. err.      z    P>|z|     [95% conf. interval]
--------------+----------------------------------------------------------------
      phospho |   .9999609    .000345    -0.11   0.910     .9992849    1.000637
        _cons |   .5748927   .3018429    -1.05   0.292      .205433    1.608805
------------------------------------------------------------------------------
Note: _cons estimates baseline odds.

. logistic Bastary_Satus copper

Logistic regression                             Number of obs =    295
                                   LR chi2(1)    =   0.71
                                   Prob > chi2   = 0.4009
Log likelihood = -191.10399                          Pseudo R2     = 0.0018

------------------------------------------------------------------------------
Bastary_Satus | Odds ratio   Std. err.      z    P>|z|     [95% conf. interval]
--------------+----------------------------------------------------------------
       copper |   1.034584   .0415965     0.85   0.398     .9561858     1.11941
        _cons |   .4918746   .0847286    -4.12   0.000     .3509372    .6894128
------------------------------------------------------------------------------
Note: _cons estimates baseline odds.

. logistic Bastary_Satus selenium

Logistic regression                             Number of obs =    295
                                   LR chi2(1)    =   0.08
                                   Prob > chi2   = 0.7839
```

Log likelihood = -191.41924 Pseudo R2 = 0.0002

Bastary_Satus | Odds ratio Std. err. z P>|z| [95% conf. interval]

--------------+--

selenium | 1.000033 .0001191 0.28 0.783 .9997994 1.000266

_cons | .5351616 .073524 -4.55 0.000 .4088286 .700533

Note: _cons estimates baseline odds.

. logistic Bastary_Satus riboflav

Logistic regression Number of obs = 295

LR chi2(1) = 0.41

Prob > chi2 = 0.5245

Log likelihood = -191.25431 Pseudo R2 = 0.0011

Bastary_Satus | Odds ratio Std. err. z P>|z| [95% conf. interval]

--------------+--

riboflav | 1.0094 .0147602 0.64 0.522 .9808812 1.038748

_cons | .5163131 .076532 -4.46 0.000 .3861369 .690375

Note: _cons estimates baseline odds.

. logistic Bastary_Satus vitb12

Logistic regression Number of obs = 295

LR chi2(1) = 0.32

Prob > chi2 = 0.5732

Log likelihood = -191.29818 Pseudo R2 = 0.0008

Bastary_Satus	Odds ratio	Std. err.	z	P>|z|	[95% conf. interval]	
vitb12	1.006936	.0122882	0.57	0.571	.9831378	1.031311
_cons	.5170403	.0790961	-4.31	0.000	.383097	.6978146

Note: _cons estimates baseline odds.

. logistic Bastary_Satus biotin

Logistic regression Number of obs = 295

LR chi2(1) = 1.90

Prob > chi2 = 0.1683

Log likelihood = -190.50773 Pseudo R2 = 0.0050

Bastary_Satus	Odds ratio	Std. err.	z	P>|z|	[95% conf. interval]	
biotin	.9890997	.0079566	-1.36	0.173	.9736272	1.004818
_cons	.730782	.1792538	-1.28	0.201	.451852	1.181896

Note: _cons estimates baseline odds.

. logistic Bastary_Satus vitD

Logistic regression Number of obs = 295

LR chi2(1)   = 49.87

Prob > chi2   = 0.0000

Log likelihood = -166.52274   Pseudo R2   = 0.1302

Bastary_Satus | Odds ratio   Std. err.   z   P>|z| [95% conf. interval]

--------------+--

vitD | .9218476   .0118481   -6.33   0.000   .8989157   .9453645

_cons | 4.8056   1.692269   4.46   0.000   2.409911   9.58284

Note: _cons estimates baseline odds.

. logistic Bastary_Satus tfiber

Logistic regression   Number of obs =   295

LR chi2(1)   =   4.61

Prob > chi2   = 0.0318

Log likelihood = -189.15258   Pseudo R2   = 0.0120

Bastary_Satus | Odds ratio   Std. err.   z   P>|z| [95% conf. interval]

--------------+--

tfiber | .9871131   .0060223   -2.13   0.034   .9753799   .9989874

_cons | .8332352   .1923872   -0.79   0.429   .5299467   1.310096

Note: _cons estimates baseline odds.

. logistic Bastary_Satus ifiber

```
Logistic regression                         Number of obs =    295
                                   LR chi2(1)    =   0.27
                                   Prob > chi2   = 0.6055
Log likelihood = -191.32344                    Pseudo R2     = 0.0007

------------------------------------------------------------------------------
Bastary_Satus | Odds ratio   Std. err.      z    P>|z|     [95% conf. interval]
--------------+---------------------------------------------------------------
      ifiber |   1.017269   .0335806     0.52   0.604     .9535364    1.085262
       _cons |   .5082385   .0920274    -3.74   0.000     .3564014    .7247626
------------------------------------------------------------------------------
Note: _cons estimates baseline odds.

. logistic Bastary_Satus tsogar

Logistic regression                         Number of obs =    295
                                   LR chi2(1)    =   0.64
                                   Prob > chi2   = 0.4247
Log likelihood = -191.13822                    Pseudo R2     = 0.0017

------------------------------------------------------------------------------
Bastary_Satus | Odds ratio   Std. err.      z    P>|z|     [95% conf. interval]
--------------+---------------------------------------------------------------
      tsogar |   .9979929   .0025136    -0.80   0.425     .9930785    1.002932
       _cons |   .6570305   .1732565    -1.59   0.111     .3918562    1.101652
------------------------------------------------------------------------------
Note: _cons estimates baseline odds.

. logistic Bastary_Satus galactos
```

Logistic regression Number of obs = 295

LR chi2(1) = 0.91

Prob > chi2 = 0.3404

Log likelihood = -191.0023 Pseudo R2 = 0.0024

Bastary_Satus	Odds ratio	Std. err.	z	P>|z|	[95% conf. interval]	
galactos	1.015403	.016167	0.96	0.337	.9842052	1.047589
_cons	.5007047	.0755704	-4.58	0.000	.3724873	.6730569

Note: _cons estimates baseline odds.

. logistic Bastary_Satus sucrose

Logistic regression Number of obs = 295

LR chi2(1) = 0.10

Prob > chi2 = 0.7550

Log likelihood = -191.40817 Pseudo R2 = 0.0003

Bastary_Satus	Odds ratio	Std. err.	z	P>|z|	[95% conf. interval]	
sucrose	.9957798	.0135178	-0.31	0.755	.9696348	1.02263
_cons	.5888046	.1639657	-1.90	0.057	.341142	1.016265

Note: _cons estimates baseline odds.

```
. logistic Bastary_Satus maltose

Logistic regression                              Number of obs =    242
                                     LR chi2(1)    =   0.21
                                     Prob > chi2   = 0.6501
Log likelihood = -154.84125                      Pseudo R2     = 0.0007

------------------------------------------------------------------------------
Bastary_Satus | Odds ratio   Std. err.      z    P>|z|     [95% conf. interval]
--------------+----------------------------------------------------------------
      maltose |   1.079677   .1818698     0.46   0.649     .7760873    1.502026
        _cons |   .4540683     .13612    -2.63   0.008     .2523186    .8171339
------------------------------------------------------------------------------
Note: _cons estimates baseline odds.

. logistic Bastary_Satus acarote

Logistic regression                              Number of obs =    295
                                     LR chi2(1)    =   0.02
                                     Prob > chi2   = 0.8956
Log likelihood = -191.44824                      Pseudo R2     = 0.0000

------------------------------------------------------------------------------
Bastary_Satus | Odds ratio   Std. err.      z    P>|z|     [95% conf. interval]
--------------+----------------------------------------------------------------
      acarote |   1.000012   .0000933     0.13   0.895     .9998293    1.000195
        _cons |    .535052   .0967158    -3.46   0.001     .3754336    .7625334
------------------------------------------------------------------------------
Note: _cons estimates baseline odds.
```

```
. logistic Bastary_Satus bcarote

Logistic regression                           Number of obs =    295
                                       LR chi2(1)    =   3.64
                                       Prob > chi2   = 0.0563
Log likelihood = -189.63587                   Pseudo R2     = 0.0095

------------------------------------------------------------------------------
Bastary_Satus | Odds ratio   Std. err.      z    P>|z|     [95% conf. interval]
--------------+----------------------------------------------------------------
      bcarote |   .9999313   .0000373    -1.84   0.065     .9998583    1.000004
        _cons |   .7324643   .1447995    -1.57   0.115     .4971804    1.079093
------------------------------------------------------------------------------
Note: _cons estimates baseline odds.

.
.
. ## TABLE 4 multivariable antioxidan ALL
Unknown #command
.
. logistic Bastary_Satus vitc

Logistic regression                           Number of obs =    295
                                       LR chi2(1)    =   1.30
                                       Prob > chi2   = 0.2545
Log likelihood = -190.80751                   Pseudo R2     = 0.0034

------------------------------------------------------------------------------
```

```
Bastary_Satus | Odds ratio   Std. err.      z    P>|z|     [95% conf. interval]
--------------+----------------------------------------------------------------
        vitc |     1.0003   .0002614     1.15   0.251     .9997875    1.000812
       _cons |   .4902892   .0753027    -4.64   0.000     .3628422    .6625016
-------------------------------------------------------------------------------
Note: _cons estimates baseline odds.

. logistic Bastary_Satus vitc Age

Logistic regression                              Number of obs =    295
                                        LR chi2(2)    =  12.34
                                        Prob > chi2   = 0.0021
Log likelihood = -185.28545                      Pseudo R2     = 0.0322

-------------------------------------------------------------------------------
Bastary_Satus | Odds ratio   Std. err.      z    P>|z|     [95% conf. interval]
--------------+----------------------------------------------------------------
        vitc |   1.000374    .000267     1.40   0.161     .9998512    1.000898
         Age |   1.028213   .0088698     3.23   0.001     1.010975    1.045745
       _cons |   .0910192   .0505073    -4.32   0.000     .0306757    .2700673
-------------------------------------------------------------------------------
Note: _cons estimates baseline odds.

. logistic Bastary_Satus vitc Age sfat linoleic iron biotin tfiber bcarote vitemg selenium zinc vitD

Logistic regression                              Number of obs =    295
                                        LR chi2(12)   =  74.96
                                        Prob > chi2   = 0.0000
Log likelihood = -153.97623                      Pseudo R2     = 0.1958
```

Bastary_Satus	Odds ratio	Std. err.	z	P>|z|	[95% conf. interval]	
vitc	1.000973	.0006032	1.61	0.107	.9997911	1.002156
Age	1.030751	.0100457	3.11	0.002	1.011248	1.050629
sfat	1.019173	.0162977	1.19	0.235	.9877259	1.051622
linoleic	.966846	.0416106	-0.78	0.433	.8886356	1.05194
iron	.9967161	.0098021	-0.33	0.738	.9776883	1.016114
biotin	.9898126	.0204591	-0.50	0.620	.9505149	1.030735
tfiber	.9918128	.0141717	-0.58	0.565	.9644221	1.019981
bcarote	.9999235	.0000787	-0.97	0.331	.9997692	1.000078
vitemg	.9925792	.0633088	-0.12	0.907	.8759388	1.124752
selenium	.9995051	.0002583	-1.92	0.055	.998999	1.000012
zinc	1.095565	.079014	1.27	0.206	.9511479	1.261909
vitD	.9175413	.0127426	-6.20	0.000	.8929031	.9428594
_cons	.829509	.748664	-0.21	0.836	.1414421	4.864783

Note: _cons estimates baseline odds.

.

. logistic Bastary_Satus vitemg

Logistic regression   Number of obs =   295

LR chi2(1)   =   1.26

Prob > chi2   = 0.2612

Log likelihood = -190.82562   Pseudo R2   = 0.0033

Bastary_Satus	Odds ratio	Std. err.	z	P>|z|	[95% conf. interval]	
vitemg	.9654474	.0305907	-1.11	0.267	.9073146	1.027305
_cons	.7963355	.2874819	-0.63	0.528	.3924706	1.61579

Note: _cons estimates baseline odds.

. logistic Bastary_Satus vitemg Age

Logistic regression Number of obs = 295

LR chi2(2) = 12.25

Prob > chi2 = 0.0022

Log likelihood = -185.32975 Pseudo R2 = 0.0320

Bastary_Satus	Odds ratio	Std. err.	z	P>|z|	[95% conf. interval]	
vitemg	.9572383	.0312249	-1.34	0.180	.897954	1.020437
Age	1.027932	.0087833	3.22	0.001	1.01086	1.045292
_cons	.1694042	.1034415	-2.91	0.004	.0511875	.5606406

Note: _cons estimates baseline odds.

. logistic Bastary_Satus vitemg Age sfat linoleic iron biotin tfiber bcarote vitc selenium zinc vitD

Logistic regression Number of obs = 295

LR chi2(12) = 74.96

Prob > chi2 = 0.0000

Log likelihood = -153.97623 Pseudo R2 = 0.1958

Bastary_Satus	Odds ratio	Std. err.	z	P>|z|	[95% conf. interval]	
vitemg	.9925792	.0633088	-0.12	0.907	.8759388	1.124752
Age	1.030751	.0100457	3.11	0.002	1.011248	1.050629
sfat	1.019173	.0162977	1.19	0.235	.9877259	1.051622
linoleic	.966846	.0416106	-0.78	0.433	.8886356	1.05194
iron	.9967161	.0098021	-0.33	0.738	.9776883	1.016114
biotin	.9898126	.0204591	-0.50	0.620	.9505149	1.030735
tfiber	.9918128	.0141717	-0.58	0.565	.9644221	1.019981
bcarote	.9999235	.0000787	-0.97	0.331	.9997692	1.000078
vitc	1.000973	.0006032	1.61	0.107	.9997911	1.002156
selenium	.9995051	.0002583	-1.92	0.055	.998999	1.000012
zinc	1.095565	.079014	1.27	0.206	.9511479	1.261909
vitD	.9175413	.0127426	-6.20	0.000	.8929031	.9428594
_cons	.829509	.748664	-0.21	0.836	.1414421	4.864783

Note: _cons estimates baseline odds.

.

. logistic Bastary_Satus vitD

Logistic regression Number of obs = 295

LR chi2(1) = 49.87

Prob > chi2 = 0.0000

Log likelihood = -166.52274 Pseudo R2 = 0.1302

```
Bastary_Satus | Odds ratio   Std. err.      z    P>|z|     [95% conf. interval]
--------------+----------------------------------------------------------------
         vitD |   .9218476   .0118481    -6.33   0.000     .8989157    .9453645
        _cons |     4.8056   1.692269     4.46   0.000     2.409911     9.58284
-------------------------------------------------------------------------------
Note: _cons estimates baseline odds.

. logistic Bastary_Satus vitD Age

Logistic regression                             Number of obs =    295
                                                LR chi2(2)    =  59.65
                                                Prob > chi2   = 0.0000
Log likelihood = -161.63063                     Pseudo R2     = 0.1558

-------------------------------------------------------------------------------
Bastary_Satus | Odds ratio   Std. err.      z    P>|z|     [95% conf. interval]
--------------+----------------------------------------------------------------
         vitD |   .9206366    .012125    -6.28   0.000     .8971762    .9447105
          Age |    1.02858   .0095338     3.04   0.002     1.010063    1.047437
        _cons |   .9253314   .5874172    -0.12   0.903     .2666485    3.211113
-------------------------------------------------------------------------------
Note: _cons estimates baseline odds.

. logistic Bastary_Satus vitD Age sfat linoleic iron biotin tfiber bcarote vitc selenium zinc vitemg

Logistic regression                             Number of obs =    295
                                                LR chi2(12)   =  74.96
                                                Prob > chi2   = 0.0000
Log likelihood = -153.97623                     Pseudo R2     = 0.1958
```

Bastary_Satus	Odds ratio	Std. err.	z	P>|z|	[95% conf. interval]	
vitD	.9175413	.0127426	-6.20	0.000	.8929031	.9428594
Age	1.030751	.0100457	3.11	0.002	1.011248	1.050629
sfat	1.019173	.0162977	1.19	0.235	.9877259	1.051622
linoleic	.966846	.0416106	-0.78	0.433	.8886356	1.05194
iron	.9967161	.0098021	-0.33	0.738	.9776883	1.016114
biotin	.9898126	.0204591	-0.50	0.620	.9505149	1.030735
tfiber	.9918128	.0141717	-0.58	0.565	.9644221	1.019981
bcarote	.9999235	.0000787	-0.97	0.331	.9997692	1.000078
vitc	1.000973	.0006032	1.61	0.107	.9997911	1.002156
selenium	.9995051	.0002583	-1.92	0.055	.998999	1.000012
zinc	1.095565	.079014	1.27	0.206	.9511479	1.261909
vitemg	.9925792	.0633088	-0.12	0.907	.8759388	1.124752
_cons	.829509	.748664	-0.21	0.836	.1414421	4.864783

Note: _cons estimates baseline odds.

.

. logistic Bastary_Satus zinc

Logistic regression Number of obs = 295

LR chi2(1) = 0.55

Prob > chi2 = 0.4582

Log likelihood = -191.18165 Pseudo R2 = 0.0014

```
Bastary_Satus | Odds ratio   Std. err.      z    P>|z|     [95% conf. interval]
--------------+----------------------------------------------------------------
         zinc |   .9801017    .026535    -0.74   0.458     .9294498    1.033514
        _cons |   .6618471   .1908781    -1.43   0.152     .3760706    1.164786
-------------------------------------------------------------------------------
Note: _cons estimates baseline odds.

. logistic Bastary_Satus zinc Age

Logistic regression                              Number of obs =    295
                                          LR chi2(2)    =  12.19
                                          Prob > chi2   = 0.0023
Log likelihood = -185.3615                       Pseudo R2     = 0.0318

-------------------------------------------------------------------------------
Bastary_Satus | Odds ratio   Std. err.      z    P>|z|     [95% conf. interval]
--------------+----------------------------------------------------------------
         zinc |    .963189   .0270268    -1.34   0.181     .9116478    1.017644
          Age |   1.029328   .0090053     3.30   0.001     1.011829    1.047131
        _cons |   .1400869   .0780833    -3.53   0.000     .0469834    .4176865
-------------------------------------------------------------------------------
Note: _cons estimates baseline odds.

. logistic Bastary_Satus zinc Age sfat linoleic iron biotin tfiber bcarote vitc selenium vitD vitemg

Logistic regression                              Number of obs =    295
                                          LR chi2(12)   =  74.96
                                          Prob > chi2   = 0.0000
Log likelihood = -153.97623                      Pseudo R2     = 0.1958
```

```
------------------------------------------------------------------------------
Bastary_Satus | Odds ratio   Std. err.      z    P>|z|     [95% conf. interval]
--------------+----------------------------------------------------------------
        zinc |   1.095565    .079014     1.27   0.206     .9511479    1.261909
         Age |   1.030751   .0100457     3.11   0.002     1.011248    1.050629
        sfat |   1.019173   .0162977     1.19   0.235     .9877259    1.051622
    linoleic |    .966846   .0416106    -0.78   0.433     .8886356     1.05194
        iron |   .9967161   .0098021    -0.33   0.738     .9776883    1.016114
      biotin |   .9898126   .0204591    -0.50   0.620     .9505149    1.030735
      tfiber |   .9918128   .0141717    -0.58   0.565     .9644221    1.019981
     bcarote |   .9999235   .0000787    -0.97   0.331     .9997692    1.000078
        vitc |   1.000973   .0006032     1.61   0.107     .9997911    1.002156
    selenium |   .9995051   .0002583    -1.92   0.055      .998999    1.000012
        vitD |   .9175413   .0127426    -6.20   0.000     .8929031    .9428594
      vitemg |   .9925792   .0633088    -0.12   0.907     .8759388    1.124752
       _cons |    .829509    .748664    -0.21   0.836     .1414421    4.864783
------------------------------------------------------------------------------
Note: _cons estimates baseline odds.

.
. logistic Bastary_Satus selenium

Logistic regression                                     Number of obs =    295
                                                        LR chi2(1)    =   0.08
                                                        Prob > chi2   = 0.7839
Log likelihood = -191.41924                             Pseudo R2     = 0.0002

------------------------------------------------------------------------------
```

```
Bastary_Satus | Odds ratio   Std. err.      z    P>|z|     [95% conf. interval]
--------------+----------------------------------------------------------------
     selenium |   1.000033   .0001191     0.28   0.783     .9997994    1.000266
        _cons |   .5351616    .073524    -4.55   0.000     .4088286     .700533
-------------------------------------------------------------------------------
Note: _cons estimates baseline odds.

. logistic Bastary_Satus selenium Age

Logistic regression                                     Number of obs =    295
                                                        LR chi2(2)    =  10.64
                                                        Prob > chi2   = 0.0049
Log likelihood = -186.13582                             Pseudo R2     = 0.0278

-------------------------------------------------------------------------------
Bastary_Satus | Odds ratio   Std. err.      z    P>|z|     [95% conf. interval]
--------------+----------------------------------------------------------------
     selenium |    1.00006   .0001207     0.50   0.620     .9998233    1.000296
          Age |   1.027402   .0087888     3.16   0.002      1.01032    1.044773
        _cons |   .1054734   .0570532    -4.16   0.000     .0365348    .3044941
-------------------------------------------------------------------------------
Note: _cons estimates baseline odds.

. logistic Bastary_Satus selenium Age sfat linoleic iron biotin tfiber bcarote vitc zinc vitD vitemg

Logistic regression                                     Number of obs =    295
                                                        LR chi2(12)   =  74.96
                                                        Prob > chi2   = 0.0000
Log likelihood = -153.97623                             Pseudo R2     = 0.1958
```

```
------------------------------------------------------------------------------
Bastary_Satus | Odds ratio   Std. err.      z    P>|z|     [95% conf. interval]
--------------+----------------------------------------------------------------
     selenium |   .9995051   .0002583    -1.92   0.055     .998999    1.000012
          Age |   1.030751   .0100457     3.11   0.002    1.011248    1.050629
         sfat |   1.019173   .0162977     1.19   0.235    .9877259    1.051622
     linoleic |    .966846   .0416106    -0.78   0.433    .8886356     1.05194
         iron |   .9967161   .0098021    -0.33   0.738    .9776883    1.016114
       biotin |   .9898126   .0204591    -0.50   0.620    .9505149    1.030735
       tfiber |   .9918128   .0141717    -0.58   0.565    .9644221    1.019981
      bcarote |   .9999235   .0000787    -0.97   0.331    .9997692    1.000078
         vitc |   1.000973   .0006032     1.61   0.107    .9997911    1.002156
         zinc |   1.095565    .079014     1.27   0.206    .9511479    1.261909
         vitD |   .9175413   .0127426    -6.20   0.000    .8929031    .9428594
       vitemg |   .9925792   .0633088    -0.12   0.907    .8759388    1.124752
        _cons |    .829509    .748664    -0.21   0.836    .1414421    4.864783
------------------------------------------------------------------------------
Note: _cons estimates baseline odds.

.

.

. ## TABLE 4 multivariable antioxidan BY SEX
Unknown #command

.

. sort Sex


.

. by Sex :logistic Bastary_Satus vitc
```

-> Sex = 1

Logistic regression Number of obs = 138

LR chi2(1) = 0.14

Prob > chi2 = 0.7124

Log likelihood = -87.770996 Pseudo R2 = 0.0008

Bastary_Satus	Odds ratio	Std. err.	z	P>|z|	[95% conf.	interval]
vitc	.9998525	.0004037	-0.37	0.715	.9990615	1.000644
_cons	.5266283	.1202918	-2.81	0.005	.3365687	.8240141

Note: _cons estimates baseline odds.

-> Sex = 2

Logistic regression Number of obs = 157

LR chi2(1) = 3.69

Prob > chi2 = 0.0547

Log likelihood = -101.562 Pseudo R2 = 0.0179

Bastary_Satus | Odds ratio Std. err. z P>|z| [95% conf. interval]

```
--------------+----------------------------------------------------------------
        vitc |   1.000698   .0003692     1.89   0.059     .9999742    1.001421
       _cons |   .4625684   .0967823    -3.68   0.000     .3069593    .6970617
-------------------------------------------------------------------------------
Note: _cons estimates baseline odds.

. by Sex :logistic Bastary_Satus vitc Age

---------------------------------------------------------------------------------------------------------------------------------------
-------------------------------------------------
-> Sex = 1

Logistic regression                               Number of obs =    138
                                                  LR chi2(2)    =  12.65
                                                  Prob > chi2   = 0.0018
Log likelihood = -81.516275                       Pseudo R2     = 0.0720

-------------------------------------------------------------------------------
Bastary_Satus | Odds ratio   Std. err.      z    P>|z|     [95% conf. interval]
--------------+----------------------------------------------------------------
         vitc |   .9999705   .0004206    -0.07   0.944     .9991464    1.000795
          Age |   1.043169   .0132696     3.32   0.001     1.017483    1.069504
        _cons |   .0410649   .0339655    -3.86   0.000     .0081176    .2077372
-------------------------------------------------------------------------------
Note: _cons estimates baseline odds.

---------------------------------------------------------------------------------------------------------------------------------------
-------------------------------------------------
-> Sex = 2
```

Logistic regression Number of obs = 157

LR chi2(2) = 4.84

Prob > chi2 = 0.0890

Log likelihood = -100.98872 Pseudo R2 = 0.0234

Bastary_Satus	Odds ratio	Std. err.	z	P>|z|	[95% conf. interval]	
vitc	1.000727	.000373	1.95	0.051	.9999961	1.001458
Age	1.012882	.0122048	1.06	0.288	.989241	1.037088
_cons	.2129244	.1630855	-2.02	0.043	.047453	.9554048

Note: _cons estimates baseline odds.

. by Sex :logistic Bastary_Satus vitc Age sfat linoleic iron biotin tfiber bcarote vitemg selenium zinc vitD

-> Sex = 1

Logistic regression Number of obs = 138

LR chi2(12) = 62.46

Prob > chi2 = 0.0000

Log likelihood = -56.60872 Pseudo R2 = 0.3555

Bastary_Satus | Odds ratio Std. err. z P>|z| [95% conf. interval]

```
       vitc |    .999502   .0013894    -0.36   0.720     .9967826    1.002229
        Age |   1.061879   .0177708     3.59   0.000     1.027614    1.097287
       sfat |   1.023206   .0262259     0.90   0.371      .973074    1.075921
   linoleic |   .9797009   .0701125    -0.29   0.774     .8514852    1.127223
       iron |   1.010408   .0193463     0.54   0.589     .9731922    1.049046
     biotin |   1.034612   .0457473     0.77   0.442     .9487243    1.128275
     tfiber |   .9762101   .0276406    -0.85   0.395     .9235113    1.031916
    bcarote |   .9998389   .0001743    -0.92   0.355     .9994973    1.000181
     vitemg |   .9412669   .0913272    -0.62   0.533     .7782592    1.138417
   selenium |   .9999501   .0004392    -0.11   0.910     .9990897    1.000811
       zinc |   .9752264   .1260068    -0.19   0.846     .7570483    1.256282
       vitD |   .8773432   .0232611    -4.94   0.000     .8329165    .9241395
      _cons |   1.263739   1.768019     0.17   0.867     .0814319    19.61193
------------------------------------------------------------------------------
Note: _cons estimates baseline odds.

--------------------------------------------------------------------------------------------------------------------------------------
-----------------------------------------------

-> Sex = 2

Logistic regression                                 Number of obs =    157
                                        LR chi2(12)   =  30.66
                                        Prob > chi2   = 0.0022
Log likelihood = -88.07887                          Pseudo R2     = 0.1482

------------------------------------------------------------------------------
Bastary_Satus | Odds ratio   Std. err.      z    P>|z|     [95% conf. interval]
--------------+---------------------------------------------------------------
       vitc |    1.00189   .0009051     2.09   0.037     1.000118    1.003666
```

Age | 1.007956 .0133724 0.60 0.550 .9820847 1.03451

sfat | 1.015853 .0235118 0.68 0.497 .9708005 1.062997

linoleic | .9477364 .0597095 -0.85 0.394 .837645 1.072297

iron | .9961583 .0123844 -0.31 0.757 .9721787 1.020729

biotin | .9738013 .0266398 -0.97 0.332 .9229634 1.027439

tfiber | .9972339 .0180941 -0.15 0.879 .9623933 1.033336

bcarote | .9999026 .0000936 -1.04 0.298 .9997192 1.000086

vitemg | 1.107411 .1068952 1.06 0.291 .9165259 1.338052

selenium | .9991442 .0004739 -1.81 0.071 .9982159 1.000073

zinc | 1.151283 .1158374 1.40 0.161 .9452305 1.402254

vitD | .9414856 .0160758 -3.53 0.000 .9104989 .9735268

_cons | .5332292 .6902604 -0.49 0.627 .0421736 6.741968

Note: _cons estimates baseline odds.

.

. by Sex :logistic Bastary_Satus vitemg

-> Sex = 1

Logistic regression Number of obs = 138

LR chi2(1) = 3.46

Prob > chi2 = 0.0629

Log likelihood = -86.109597 Pseudo R2 = 0.0197

Bastary_Satus | Odds ratio Std. err. z P>|z| [95% conf. interval]

--------------+--

vitemg | .919227 .0437846 -1.77 0.077 .8372947 1.009177

_cons | 1.191278 .6091563 0.34 0.732 .4372744 3.245431

Note: _cons estimates baseline odds.

-> Sex = 2

Logistic regression Number of obs = 157

LR chi2(1) = 0.05

Prob > chi2 = 0.8191

Log likelihood = -103.38187 Pseudo R2 = 0.0003

Bastary_Satus | Odds ratio Std. err. z P>|z| [95% conf. interval]

--------------+--

vitemg | 1.010423 .0457464 0.23 0.819 .9246245 1.104182

_cons | .5216966 .27834 -1.22 0.223 .1833487 1.484425

Note: _cons estimates baseline odds.

. by Sex :logistic Bastary_Satus vitemg Age

-> Sex = 1

```
Logistic regression                         Number of obs =    138
                                   LR chi2(2)    =  17.12
                                   Prob > chi2   = 0.0002
Log likelihood = -79.276851                     Pseudo R2     = 0.0975

------------------------------------------------------------------------------
Bastary_Satus | Odds ratio   Std. err.      z    P>|z|     [95% conf. interval]
--------------+----------------------------------------------------------------
      vitemg |   .9015838    .047054    -1.99   0.047     .8139197    .9986899
         Age |   1.045626   .0134745     3.46   0.001     1.019547    1.072372
       _cons |   .1025492   .0921534    -2.53   0.011     .0176206     .596821
------------------------------------------------------------------------------
Note: _cons estimates baseline odds.

------------------------------------------------------------------------------------------------------------------------------------------
--------------------------------------------------
-> Sex = 2

Logistic regression                         Number of obs =    157
                                   LR chi2(2)    =   0.91
                                   Prob > chi2   = 0.6338
Log likelihood = -102.95205                     Pseudo R2     = 0.0044

------------------------------------------------------------------------------
Bastary_Satus | Odds ratio   Std. err.      z    P>|z|     [95% conf. interval]
--------------+----------------------------------------------------------------
      vitemg |   1.006732   .0458299     0.15   0.883     .9207983    1.100687
         Age |   1.010981   .0119789     0.92   0.357     .9877733    1.034734
       _cons |   .2828529   .2422169    -1.47   0.140     .0528016    1.515216
```

Note: _cons estimates baseline odds.

. by Sex :logistic Bastary_Satus vitemg Age sfat linoleic iron biotin tfiber bcarote vitc selenium zinc vitD

-> Sex = 1

Logistic regression Number of obs = 138

LR chi2(12) = 62.46

Prob > chi2 = 0.0000

Log likelihood = -56.60872 Pseudo R2 = 0.3555

Bastary_Satus	Odds ratio	Std. err.	z	P>|z|	[95% conf. interval]	
vitemg	.9412669	.0913272	-0.62	0.533	.7782592	1.138417
Age	1.061879	.0177708	3.59	0.000	1.027614	1.097287
sfat	1.023206	.0262259	0.90	0.371	.973074	1.075921
linoleic	.9797009	.0701125	-0.29	0.774	.8514852	1.127223
iron	1.010408	.0193463	0.54	0.589	.9731922	1.049046
biotin	1.034612	.0457473	0.77	0.442	.9487243	1.128275
tfiber	.9762101	.0276406	-0.85	0.395	.9235113	1.031916
bcarote	.9998389	.0001743	-0.92	0.355	.9994973	1.000181
vitc	.999502	.0013894	-0.36	0.720	.9967826	1.002229
selenium	.9999501	.0004392	-0.11	0.910	.9990897	1.000811
zinc	.9752264	.1260068	-0.19	0.846	.7570483	1.256282
vitD	.8773432	.0232611	-4.94	0.000	.8329165	.9241395

_cons | 1.263739 1.768019 0.17 0.867 .0814319 19.61193

Note: _cons estimates baseline odds.

-> Sex = 2

Logistic regression Number of obs = 157

LR chi2(12) = 30.66

Prob > chi2 = 0.0022

Log likelihood = -88.07887 Pseudo R2 = 0.1482

Bastary_Satus	Odds ratio	Std. err.	z	P>|z|	[95% conf. interval]	
vitemg	1.107411	.1068952	1.06	0.291	.9165259	1.338052
Age	1.007956	.0133724	0.60	0.550	.9820847	1.03451
sfat	1.015853	.0235118	0.68	0.497	.9708005	1.062997
linoleic	.9477364	.0597095	-0.85	0.394	.837645	1.072297
iron	.9961583	.0123844	-0.31	0.757	.9721787	1.020729
biotin	.9738013	.0266398	-0.97	0.332	.9229634	1.027439
tfiber	.9972339	.0180941	-0.15	0.879	.9623933	1.033336
bcarote	.9999026	.0000936	-1.04	0.298	.9997192	1.000086
vitc	1.00189	.0009051	2.09	0.037	1.000118	1.003666
selenium	.9991442	.0004739	-1.81	0.071	.9982159	1.000073
zinc	1.151283	.1158374	1.40	0.161	.9452305	1.402254
vitD	.9414856	.0160758	-3.53	0.000	.9104989	.9735268
_cons	.5332292	.6902604	-0.49	0.627	.0421736	6.741968

```
------------------------------------------------------------------------------

Note: _cons estimates baseline odds.


.

. by Sex :logistic Bastary_Satus vitD


-------------------------------------------------------------------------------------------------------------------------------------------------------------------------------------------

-> Sex = 1


Logistic regression                    Number of obs =     138

                                       LR chi2(1)    =   38.93

                                       Prob > chi2   =  0.0000

Log likelihood = -68.374408            Pseudo R2     =  0.2216


------------------------------------------------------------------------------

Bastary_Satus | Odds ratio   Std. err.      z    P>|z|     [95% conf. interval]

--------------+----------------------------------------------------------------

         vitD |   .8909703   .0194056    -5.30   0.000     .8537364    .9298281

        _cons |   11.37725   6.739355     4.11   0.000     3.563077    36.32868

------------------------------------------------------------------------------

Note: _cons estimates baseline odds.


-------------------------------------------------------------------------------------------------------------------------------------------------------------------------------------------

-> Sex = 2


Logistic regression                    Number of obs =     157

                                       LR chi2(1)    =   15.33
```

Prob > chi2 = 0.0001

Log likelihood = -95.74109 Pseudo R2 = 0.0741

Bastary_Satus	Odds ratio	Std. err.	z	P>|z|	[95% conf. interval]	
vitD	.9440312	.0150155	-3.62	0.000	.9150552	.9739246
_cons	2.690402	1.180827	2.25	0.024	1.138208	6.359347

Note: _cons estimates baseline odds.

. by Sex :logistic Bastary_Satus vitD Age

-> Sex = 1

Logistic regression Number of obs = 138

LR chi2(2) = 54.04

Prob > chi2 = 0.0000

Log likelihood = -60.818457 Pseudo R2 = 0.3076

Bastary_Satus	Odds ratio	Std. err.	z	P>|z|	[95% conf. interval]	
vitD	.8777398	.0217871	-5.25	0.000	.83606	.9214975
Age	1.055981	.0162391	3.54	0.000	1.024628	1.088294
_cons	.6650718	.6329095	-0.43	0.668	.1029984	4.294441

Note: _cons estimates baseline odds.

-> Sex = 2

Logistic regression   Number of obs =   157

LR chi2(2)   = 15.89

Prob > chi2   = 0.0004

Log likelihood = -95.461048   Pseudo R2   = 0.0769

Bastary_Satus	Odds ratio	Std. err.	z	P>|z|	[95% conf. interval]	
vitD	.9444145	.0150571	-3.59	0.000	.9153594	.9743919
Age	1.009329	.0125821	0.74	0.456	.9849671	1.034293
_cons	1.526166	1.335323	0.48	0.629	.2746887	8.479351

Note: _cons estimates baseline odds.

. by Sex :logistic Bastary_Satus vitD Age sfat linoleic iron biotin tfiber bcarote vitc selenium zinc vitemg

----

-> Sex = 1

Logistic regression   Number of obs =   138

LR chi2(12)   = 62.46

Prob > chi2   = 0.0000

Log likelihood = -56.60872 Pseudo R2 = 0.3555

Bastary_Satus	Odds ratio	Std. err.	z	P>|z|	[95% conf. interval]	
vitD	.8773432	.0232611	-4.94	0.000	.8329165	.9241395
Age	1.061879	.0177708	3.59	0.000	1.027614	1.097287
sfat	1.023206	.0262259	0.90	0.371	.973074	1.075921
linoleic	.9797009	.0701125	-0.29	0.774	.8514852	1.127223
iron	1.010408	.0193463	0.54	0.589	.9731922	1.049046
biotin	1.034612	.0457473	0.77	0.442	.9487243	1.128275
tfiber	.9762101	.0276406	-0.85	0.395	.9235113	1.031916
bcarote	.9998389	.0001743	-0.92	0.355	.9994973	1.000181
vitc	.999502	.0013894	-0.36	0.720	.9967826	1.002229
selenium	.9999501	.0004392	-0.11	0.910	.9990897	1.000811
zinc	.9752264	.1260068	-0.19	0.846	.7570483	1.256282
vitemg	.9412669	.0913272	-0.62	0.533	.7782592	1.138417
_cons	1.263739	1.768019	0.17	0.867	.0814319	19.61193

Note: _cons estimates baseline odds.

-> Sex = 2

Logistic regression Number of obs = 157

LR chi2(12) = 30.66

Prob > chi2 = 0.0022

Log likelihood = -88.07887 Pseudo R2 = 0.1482

Bastary_Satus	Odds ratio	Std. err.	z	P>|z|	[95% conf. interval]	
vitD	.9414856	.0160758	-3.53	0.000	.9104989	.9735268
Age	1.007956	.0133724	0.60	0.550	.9820847	1.03451
sfat	1.015853	.0235118	0.68	0.497	.9708005	1.062997
linoleic	.9477364	.0597095	-0.85	0.394	.837645	1.072297
iron	.9961583	.0123844	-0.31	0.757	.9721787	1.020729
biotin	.9738013	.0266398	-0.97	0.332	.9229634	1.027439
tfiber	.9972339	.0180941	-0.15	0.879	.9623933	1.033336
bcarote	.9999026	.0000936	-1.04	0.298	.9997192	1.000086
vitc	1.00189	.0009051	2.09	0.037	1.000118	1.003666
selenium	.9991442	.0004739	-1.81	0.071	.9982159	1.000073
zinc	1.151283	.1158374	1.40	0.161	.9452305	1.402254
vitemg	1.107411	.1068952	1.06	0.291	.9165259	1.338052
_cons	.5332292	.6902604	-0.49	0.627	.0421736	6.741968

Note: _cons estimates baseline odds.

.

. by Sex :logistic Bastary_Satus zinc

-> Sex = 1

Logistic regression Number of obs = 138

LR chi2(1) = 0.10

Prob > chi2 = 0.7478

Log likelihood = -87.78727 Pseudo R2 = 0.0006

Bastary_Satus	Odds ratio	Std. err.	z	P>|z|	[95% conf. interval]	
zinc	.9873908	.0389442	-0.32	0.748	.9139372	1.066748
_cons	.5633537	.2315023	-1.40	0.163	.2517629	1.26058

Note: _cons estimates baseline odds.

-> Sex = 2

Logistic regression Number of obs = 157

LR chi2(1) = 0.61

Prob > chi2 = 0.4342

Log likelihood = -103.10223 Pseudo R2 = 0.0030

Bastary_Satus	Odds ratio	Std. err.	z	P>|z|	[95% conf. interval]	
zinc	.9710872	.0364318	-0.78	0.434	.9022442	1.045183
_cons	.7851929	.3196966	-0.59	0.553	.3535118	1.744009

Note: _cons estimates baseline odds.

. by Sex :logistic Bastary_Satus zinc Age

-> Sex = 1

Logistic regression Number of obs = 138

LR chi2(2) = 14.11

Prob > chi2 = 0.0009

Log likelihood = -80.781486 Pseudo R2 = 0.0803

Bastary_Satus	Odds ratio	Std. err.	z	P>|z|	[95% conf. interval]	
zinc	.9499416	.0403316	-1.21	0.226	.8740927	1.032372
Age	1.047571	.0139783	3.48	0.000	1.020529	1.075329
_cons	.0511518	.0420184	-3.62	0.000	.0102246	.2559033

Note: _cons estimates baseline odds.

-> Sex = 2

Logistic regression Number of obs = 157

LR chi2(2) = 1.68

Prob > chi2 = 0.4308

Log likelihood = -102.56597 Pseudo R2 = 0.0081

```
Bastary_Satus | Odds ratio   Std. err.      z    P>|z|     [95% conf. interval]
--------------+----------------------------------------------------------------
         zinc |   .9667763   .0366937    -0.89   0.373     .8974678    1.041437
          Age |   1.012365   .0121067     1.03   0.304     .9889122    1.036374
        _cons |   .3935679    .310014    -1.18   0.236     .0840479    1.842945
-------------------------------------------------------------------------------
Note: _cons estimates baseline odds.

. by Sex :logistic Bastary_Satus zinc Age sfat linoleic iron biotin tfiber bcarote vitc selenium vitD vitemg

------------------------------------------------------------------------------------------------------------------------------------------------------------------------------------
-> Sex = 1

Logistic regression                                     Number of obs =    138
                                                        LR chi2(12)   =  62.46
                                                        Prob > chi2   = 0.0000
Log likelihood = -56.60872                              Pseudo R2     = 0.3555

-------------------------------------------------------------------------------
Bastary_Satus | Odds ratio   Std. err.      z    P>|z|     [95% conf. interval]
--------------+----------------------------------------------------------------
         zinc |   .9752264   .1260068    -0.19   0.846     .7570483    1.256282
          Age |   1.061879   .0177708     3.59   0.000     1.027614    1.097287
         sfat |   1.023206   .0262259     0.90   0.371      .973074    1.075921
     linoleic |   .9797009   .0701125    -0.29   0.774     .8514852    1.127223
         iron |   1.010408   .0193463     0.54   0.589     .9731922    1.049046
       biotin |   1.034612   .0457473     0.77   0.442     .9487243    1.128275
       tfiber |   .9762101   .0276406    -0.85   0.395     .9235113    1.031916
```

bcarote	.9998389	.0001743	-0.92	0.355	.9994973	1.000181
vitc	.999502	.0013894	-0.36	0.720	.9967826	1.002229
selenium	.9999501	.0004392	-0.11	0.910	.9990897	1.000811
vitD	.8773432	.0232611	-4.94	0.000	.8329165	.9241395
vitemg	.9412669	.0913272	-0.62	0.533	.7782592	1.138417
_cons	1.263739	1.768019	0.17	0.867	.0814319	19.61193

Note: _cons estimates baseline odds.

-> Sex = 2

Logistic regression Number of obs = 157

LR chi2(12) = 30.66

Prob > chi2 = 0.0022

Log likelihood = -88.07887 Pseudo R2 = 0.1482

Bastary_Satus	Odds ratio	Std. err.	z	P>|z|	[95% conf.	interval]
zinc	1.151283	.1158374	1.40	0.161	.9452305	1.402254
Age	1.007956	.0133724	0.60	0.550	.9820847	1.03451
sfat	1.015853	.0235118	0.68	0.497	.9708005	1.062997
linoleic	.9477364	.0597095	-0.85	0.394	.837645	1.072297
iron	.9961583	.0123844	-0.31	0.757	.9721787	1.020729
biotin	.9738013	.0266398	-0.97	0.332	.9229634	1.027439
tfiber	.9972339	.0180941	-0.15	0.879	.9623933	1.033336
bcarote	.9999026	.0000936	-1.04	0.298	.9997192	1.000086

```
        vitc |    1.00189   .0009051     2.09   0.037     1.000118    1.003666
    selenium |   .9991442   .0004739    -1.81   0.071     .9982159    1.000073
        vitD |   .9414856   .0160758    -3.53   0.000     .9104989    .9735268
      vitemg |   1.107411   .1068952     1.06   0.291     .9165259    1.338052
       _cons |   .5332292   .6902604    -0.49   0.627     .0421736    6.741968
------------------------------------------------------------------------------
Note: _cons estimates baseline odds.


.
. by Sex :logistic Bastary_Satus selenium


-------------------------------------------------------------------------------------------------------------------------------------------
--------------------------------------------------

-> Sex = 1


Logistic regression                                     Number of obs =    138
                                                        LR chi2(1)    =   0.03
                                                        Prob > chi2   = 0.8555
Log likelihood = -87.822364                             Pseudo R2     = 0.0002


------------------------------------------------------------------------------
Bastary_Satus | Odds ratio   Std. err.      z    P>|z|     [95% conf. interval]
--------------+----------------------------------------------------------------
     selenium |   1.000028   .0001538     0.18   0.855     .9997268     1.00033
        _cons |   .4910802   .1012309    -3.45   0.001     .3278585    .7355606
------------------------------------------------------------------------------
Note: _cons estimates baseline odds.
```

-> Sex = 2

Logistic regression Number of obs = 157

LR chi2(1) = 0.10

Prob > chi2 = 0.7470

Log likelihood = -103.35598 Pseudo R2 = 0.0005

Bastary_Satus	Odds ratio	Std. err.	z	P>|z|	[95% conf. interval]	
selenium	1.000062	.000192	0.32	0.746	.999686	1.000439
_cons	.5703522	.1057236	-3.03	0.002	.3966065	.8202126

Note: _cons estimates baseline odds.

. by Sex :logistic Bastary_Satus selenium Age

-> Sex = 1

Logistic regression Number of obs = 138

LR chi2(2) = 12.85

Prob > chi2 = 0.0016

Log likelihood = -81.412909 Pseudo R2 = 0.0732

```
Bastary_Satus | Odds ratio   Std. err.      z    P>|z|     [95% conf. interval]
--------------+----------------------------------------------------------------
     selenium |   1.000073   .0001583     0.46   0.643     .9997632    1.000384
          Age |   1.043881   .0133441     3.36   0.001     1.018052    1.070365
        _cons |   .0371619   .0305128    -4.01   0.000     .0074335    .1857804
-------------------------------------------------------------------------------
Note: _cons estimates baseline odds.

-------------------------------------------------------------------------------------------------------------------------------------------
--------------------------------------------------
-> Sex = 2

Logistic regression                                     Number of obs =    157
                                                        LR chi2(2)    =   1.03
                                                        Prob > chi2   = 0.5984
Log likelihood = -102.89451                             Pseudo R2     = 0.0050

-------------------------------------------------------------------------------
Bastary_Satus | Odds ratio   Std. err.      z    P>|z|     [95% conf. interval]
--------------+----------------------------------------------------------------
     selenium |   1.000072   .0001927     0.37   0.710      .999694    1.000449
          Age |   1.011366   .0119762     0.95   0.340     .9881638    1.035114
        _cons |   .2889452   .2138921    -1.68   0.094     .0677193    1.232874
-------------------------------------------------------------------------------
Note: _cons estimates baseline odds.

. by Sex :logistic Bastary_Satus selenium Age sfat linoleic iron biotin tfiber bcarote vitc zinc vitD vitemg
```

-> Sex = 1

Logistic regression Number of obs = 138

LR chi2(12) = 62.46

Prob > chi2 = 0.0000

Log likelihood = -56.60872 Pseudo R2 = 0.3555

Bastary_Satus	Odds ratio	Std. err.	z	P>|z|	[95% conf.	interval]
selenium	.9999501	.0004392	-0.11	0.910	.9990897	1.000811
Age	1.061879	.0177708	3.59	0.000	1.027614	1.097287
sfat	1.023206	.0262259	0.90	0.371	.973074	1.075921
linoleic	.9797009	.0701125	-0.29	0.774	.8514852	1.127223
iron	1.010408	.0193463	0.54	0.589	.9731922	1.049046
biotin	1.034612	.0457473	0.77	0.442	.9487243	1.128275
tfiber	.9762101	.0276406	-0.85	0.395	.9235113	1.031916
bcarote	.9998389	.0001743	-0.92	0.355	.9994973	1.000181
vitc	.999502	.0013894	-0.36	0.720	.9967826	1.002229
zinc	.9752264	.1260068	-0.19	0.846	.7570483	1.256282
vitD	.8773432	.0232611	-4.94	0.000	.8329165	.9241395
vitemg	.9412669	.0913272	-0.62	0.533	.7782592	1.138417
_cons	1.263739	1.768019	0.17	0.867	.0814319	19.61193

Note: _cons estimates baseline odds.

-> Sex = 2

Logistic regression Number of obs = 157

LR chi2(12) = 30.66

Prob > chi2 = 0.0022

Log likelihood = -88.07887 Pseudo R2 = 0.1482

Bastary_Satus	Odds ratio	Std. err.	z	P>|z|	[95% conf.	interval]
selenium	.9991442	.0004739	-1.81	0.071	.9982159	1.000073
Age	1.007956	.0133724	0.60	0.550	.9820847	1.03451
sfat	1.015853	.0235118	0.68	0.497	.9708005	1.062997
linoleic	.9477364	.0597095	-0.85	0.394	.837645	1.072297
iron	.9961583	.0123844	-0.31	0.757	.9721787	1.020729
biotin	.9738013	.0266398	-0.97	0.332	.9229634	1.027439
tfiber	.9972339	.0180941	-0.15	0.879	.9623933	1.033336
bcarote	.9999026	.0000936	-1.04	0.298	.9997192	1.000086
vitc	1.00189	.0009051	2.09	0.037	1.000118	1.003666
zinc	1.151283	.1158374	1.40	0.161	.9452305	1.402254
vitD	.9414856	.0160758	-3.53	0.000	.9104989	.9735268
vitemg	1.107411	.1068952	1.06	0.291	.9165259	1.338052
_cons	.5332292	.6902604	-0.49	0.627	.0421736	6.741968

Note: _cons estimates baseline odds.

.

```
.

.

. ## TABLE 5 multivariable DAQS_Tertile ALL

Unknown #command

.

. logistic Bastary_Satus i.newDAQS_Tertile


Logistic regression                          Number of obs =    295

                                   LR chi2(2)    =  24.44

                                   Prob > chi2   = 0.0000

Log likelihood = -179.23572                      Pseudo R2     = 0.0638


------------------------------------------------------------------------------

  Bastary_Satus | Odds ratio   Std. err.      z    P>|z|     [95% conf. interval]

----------------+----------------------------------------------------------------

newDAQS_Tertile |

          1  |   2.814416   .9284249    3.14   0.002    1.474322    5.372597

          2  |   4.987088   1.717465    4.67   0.000    2.539266    9.794581

             |

        _cons |   .2151899   .0575333    -5.75   0.000    .1274219    .3634124

------------------------------------------------------------------------------

Note: _cons estimates baseline odds.


. logistic Bastary_Satus i.newDAQS_Tertile Age


Logistic regression                          Number of obs =    295

                                   LR chi2(3)    =  35.95

                                   Prob > chi2   = 0.0000

Log likelihood = -173.48411                      Pseudo R2     = 0.0939
```

```
--------------------------------------------------------------------------------
  Bastary_Satus | Odds ratio   Std. err.      z    P>|z|     [95% conf. interval]
----------------+---------------------------------------------------------------
newDAQS_Tertile |
            1  |   3.077187   1.037277     3.33   0.001     1.589372    5.957748
            2  |   5.303347   1.871534     4.73   0.000     2.655606    10.59099
               |
           Age |   1.029895   .0092387     3.28   0.001     1.011946    1.048163
         _cons |   .0353345   .0222384    -5.31   0.000     .0102915    .1213161
--------------------------------------------------------------------------------
Note: _cons estimates baseline odds.

. logistic Bastary_Satus i.newDAQS_Tertile Age sfat linoleic iron biotin tfiber bcarote

Logistic regression                               Number of obs =    295
                                              LR chi2(9)    =  45.60
                                              Prob > chi2   = 0.0000
Log likelihood = -168.65439                       Pseudo R2     = 0.1191

--------------------------------------------------------------------------------
  Bastary_Satus | Odds ratio   Std. err.      z    P>|z|     [95% conf. interval]
----------------+---------------------------------------------------------------
newDAQS_Tertile |
            1  |   4.103332   1.687965     3.43   0.001     1.832241    9.189476
            2  |   8.587072   3.890799     4.75   0.000     3.533182    20.87008
               |
           Age |   1.029788   .0095515     3.16   0.002     1.011236     1.04868
          sfat |   1.033187   .0150133     2.25   0.025     1.004177    1.063035
```

```
    linoleic |   .9863251   .0261266    -0.52   0.603     .9364245    1.038885
        iron |   .9991808   .0087333    -0.09   0.925     .9822096    1.016445
      biotin |   1.010784   .0158615     0.68   0.494      .980169    1.042355
      tfiber |   .9864248   .0132383    -1.02   0.308     .9608165    1.012716
     bcarote |   1.000096    .000071     1.36   0.174     .9999573    1.000236
       _cons |   .0110075   .0099119    -5.01   0.000     .0018845    .0642939
------------------------------------------------------------------------------
Note: _cons estimates baseline odds.


.
. ## TABLE 5 multivariable DAQS_Tertile BY SEX
Unknown #command
.
. by Sex :logistic Bastary_Satus i.newDAQS_Tertile


-----------------------------------------------------------------------------------------------------------------------------------
-------------------------------------------------
-> Sex = 1


Logistic regression                             Number of obs =    138
                                                LR chi2(2)    =  17.92
                                                Prob > chi2   = 0.0001
Log likelihood = -78.878016                     Pseudo R2     = 0.1020


------------------------------------------------------------------------------
  Bastary_Satus | Odds ratio   Std. err.      z    P>|z|     [95% conf. interval]
----------------+-------------------------------------------------------------
newDAQS_Tertile |
              1  |   3.111111   1.756743     2.01   0.044     1.028655    9.409385
```

2 | 8.333333 4.685901 3.77 0.000 2.768096 25.08744

|

_cons | .1428571 .0682988 -4.07 0.000 .0559693 .3646315

Note: _cons estimates baseline odds.

-> Sex = 2

Logistic regression Number of obs = 157

LR chi2(2) = 9.67

Prob > chi2 = 0.0080

Log likelihood = -98.573943 Pseudo R2 = 0.0467

Bastary_Satus | Odds ratio Std. err. z P>|z| [95% conf. interval]

----------------+--

newDAQS_Tertile |

1 | 2.828571 1.171961 2.51 0.012 1.255709 6.371552

2 | 3.483333 1.591286 2.73 0.006 1.422794 8.528017

|

_cons | .2727273 .0888189 -3.99 0.000 .1440516 .5163438

Note: _cons estimates baseline odds.

. by Sex :logistic Bastary_Satus i.newDAQS_Tertile Age

```
--------------------------------------------------------------------------------------------------------------------------------------------
---------------------------------------------------

-> Sex = 1


Logistic regression                           Number of obs =    138

                                        LR chi2(3)    =  31.61

                                        Prob > chi2   = 0.0000

Log likelihood = -72.0348                        Pseudo R2     = 0.1799


--------------------------------------------------------------------------------

  Bastary_Satus | Odds ratio   Std. err.      z    P>|z|     [95% conf. interval]

----------------+---------------------------------------------------------------

newDAQS_Tertile |

          1  |   3.699929   2.173754    2.23   0.026    1.169774    11.70267

          2  |   10.14746   6.051408    3.89   0.000    3.153155    32.65644

             |

          Age |   1.048308   .0143748    3.44   0.001    1.020509    1.076864

        _cons |   .0076583   .0078435   -4.76   0.000    .0010288    .0570056

--------------------------------------------------------------------------------

Note: _cons estimates baseline odds.


--------------------------------------------------------------------------------------------------------------------------------------------
---------------------------------------------------

-> Sex = 2


Logistic regression                           Number of obs =    157

                                        LR chi2(3)    =  10.77

                                        Prob > chi2   = 0.0131

Log likelihood = -98.025375                        Pseudo R2     = 0.0521
```

```
--------------------------------------------------------------------------------
  Bastary_Satus | Odds ratio   Std. err.      z    P>|z|     [95% conf. interval]
----------------+---------------------------------------------------------------
newDAQS_Tertile |
              1  |   2.915128   1.216282     2.56   0.010     1.286796    6.603976
              2  |   3.500996   1.605453     2.73   0.006     1.425134    8.600575
                 |
            Age |   1.012866   .0124596     1.04   0.299     .9887373    1.037583
          _cons |   .1252258   .1034129    -2.52   0.012     .0248177    .6318668
--------------------------------------------------------------------------------
Note: _cons estimates baseline odds.

. by Sex :logistic Bastary_Satus i.newDAQS_Tertile Age sfat linoleic iron biotin tfiber bcarote

-------------------------------------------------------------------------------------------------------------------------------------------------------------------------------------------------------

-> Sex = 1

Logistic regression                              Number of obs =    138
                                           LR chi2(9)    =  44.50
                                           Prob > chi2   = 0.0000
Log likelihood = -65.590946                       Pseudo R2     = 0.2533

--------------------------------------------------------------------------------
  Bastary_Satus | Odds ratio   Std. err.      z    P>|z|     [95% conf. interval]
----------------+---------------------------------------------------------------
newDAQS_Tertile |
              1  |   8.802521   6.954025     2.75   0.006     1.871349    41.40564
```

```
           2  |   34.92133   30.13815    4.12   0.000    6.433959    189.541
              |
         Age |   1.048024   .0158913    3.09   0.002    1.017336    1.079638
        sfat |   1.069806    .026796    2.69   0.007    1.018555    1.123636
    linoleic |   .9454274    .047919   -1.11   0.268    .8560221     1.04417
        iron |   .9896989   .0181738   -0.56   0.573    .9547123    1.025968
      biotin |   1.027235   .0309985    0.89   0.373     .968241    1.089824
      tfiber |   .9919068   .0253516   -0.32   0.751    .9434425    1.042861
     bcarote |   1.000174   .0001578    1.10   0.271    .9998646    1.000483
       _cons |   .0004419    .000733   -4.66   0.000    .0000171    .0114125
------------------------------------------------------------------------------
Note: _cons estimates baseline odds.

-------------------------------------------------------------------------------------------------------------------------------------------
-------------------------------------------------

-> Sex = 2

Logistic regression                                  Number of obs =    157
                                                     LR chi2(9)    =  13.08
                                                     Prob > chi2   = 0.1589
Log likelihood = -96.866585                          Pseudo R2     = 0.0633

------------------------------------------------------------------------------
  Bastary_Satus | Odds ratio   Std. err.      z    P>|z|     [95% conf. interval]
----------------+-------------------------------------------------------------
newDAQS_Tertile |
           1  |    3.30531   1.675862    2.36   0.018    1.223588    8.928721
           2  |   4.574847   2.666985    2.61   0.009    1.459332    14.34164
              |
```

```
         Age |   1.013105   .0127092     1.04   0.299     .9884995    1.038324
        sfat |     1.0137   .0197573     0.70   0.485     .9757069    1.053173
    linoleic |   1.016931   .0374659     0.46   0.649      .946088    1.093079
        iron |   1.000572   .0106369     0.05   0.957       .97994    1.021639
      biotin |   .9950978   .0192928    -0.25   0.800      .957994    1.033639
      tfiber |   .9864652    .016153    -0.83   0.405     .9553085    1.018638
     bcarote |   1.000075   .0000834     0.89   0.371     .9999111    1.000238
       _cons |   .0836386   .0959713    -2.16   0.031     .0088246    .7927212
------------------------------------------------------------------------------
Note: _cons estimates baseline odds.

.

.

. ## P FOR TREND
Unknown #command

. logistic Bastary_Satus newDAQS_Tertile

Logistic regression                                     Number of obs =    295
                                                        LR chi2(1)    =  23.64
                                                        Prob > chi2   = 0.0000
Log likelihood = -179.6352                              Pseudo R2     = 0.0617

------------------------------------------------------------------------------
  Bastary_Satus | Odds ratio   Std. err.      z    P>|z|     [95% conf. interval]
----------------+-------------------------------------------------------------
newDAQS_Tertile |   2.191908   .3673127     4.68   0.000     1.578268    3.044135
          _cons |   .2423361   .0541623    -6.34   0.000     .1563774    .3755454
------------------------------------------------------------------------------
Note: _cons estimates baseline odds.
```

. logistic Bastary_Satus newDAQS_Tertile Age

Logistic regression Number of obs = 295

LR chi2(2) = 34.74

Prob > chi2 = 0.0000

Log likelihood = -174.08536 Pseudo R2 = 0.0907

Bastary_Satus	Odds ratio	Std. err.	z	P>|z|	[95% conf. interval]	
newDAQS_Tertile	2.252017	.385825	4.74	0.000	1.609682	3.150671
Age	1.029249	.0091901	3.23	0.001	1.011394	1.04742
_cons	.0425219	.0255184	-5.26	0.000	.0131155	.1378604

Note: _cons estimates baseline odds.

. logistic Bastary_Satus newDAQS_Tertile Age sfat linoleic iron biotin tfiber bcarote

Logistic regression Number of obs = 295

LR chi2(8) = 44.25

Prob > chi2 = 0.0000

Log likelihood = -169.33345 Pseudo R2 = 0.1156

Bastary_Satus	Odds ratio	Std. err.	z	P>|z|	[95% conf. interval]	
newDAQS_Tertile	2.755306	.593742	4.70	0.000	1.806109	4.20335
Age	1.029737	.0095549	3.16	0.002	1.011178	1.048635

```
         sfat |    1.03156   .0148618     2.16   0.031     1.002839    1.061104
     linoleic |   .9874195   .0260616    -0.48   0.631     .9376384    1.039844
         iron |   .9992686   .0086857    -0.08   0.933      .982389    1.016438
       biotin |   1.010178   .0157088     0.65   0.515     .9798537    1.041441
       tfiber |   .9849849   .0131908    -1.13   0.259     .9594677    1.011181
     bcarote |   1.000083   .0000697     1.19   0.234     .9999462     1.00022
        _cons |   .0157339   .0132277    -4.94   0.000     .0030284    .0817432
------------------------------------------------------------------------------
Note: _cons estimates baseline odds.

.
. by Sex :logistic Bastary_Satus newDAQS_Tertile

-----------------------------------------------------------------------------------------------------------------------------------------------------------------------------------------
-> Sex = 1

Logistic regression                             Number of obs =    138
                                       LR chi2(1)    =  17.89
                                       Prob > chi2   = 0.0000
Log likelihood = -78.894581                     Pseudo R2     = 0.1018

------------------------------------------------------------------------------
  Bastary_Satus | Odds ratio   Std. err.      z    P>|z|     [95% conf. interval]
----------------+-------------------------------------------------------------
newDAQS_Tertile |   2.843399   .7550231     3.94   0.000     1.689716     4.78478
          _cons |   .1501339   .0578323    -4.92   0.000     .0705654    .3194225
------------------------------------------------------------------------------
Note: _cons estimates baseline odds.
```

-> Sex = 2

Logistic regression Number of obs = 157

LR chi2(1) = 8.20

Prob > chi2 = 0.0042

Log likelihood = -99.308213 Pseudo R2 = 0.0396

```
------------------------------------------------------------------------------
  Bastary_Satus | Odds ratio   Std. err.      z    P>|z|     [95% conf. interval]
----------------+-------------------------------------------------------------
newDAQS_Tertile |    1.86922   .4171657     2.80   0.005     1.206959    2.894866
         _cons |    .325899   .0902767    -4.05   0.000     .1893622    .5608838
------------------------------------------------------------------------------
```

Note: _cons estimates baseline odds.

. by Sex :logistic Bastary_Satus newDAQS_Tertile Age

-> Sex = 1

Logistic regression Number of obs = 138

LR chi2(2) = 31.49

Prob > chi2 = 0.0000

Log likelihood = -72.095488 Pseudo R2 = 0.1792

```
------------------------------------------------------------------------------
  Bastary_Satus | Odds ratio   Std. err.      z    P>|z|     [95% conf. interval]
----------------+-------------------------------------------------------------
newDAQS_Tertile |   3.100178   .8763718     4.00   0.000     1.781416    5.395205
            Age |   1.048084   .0143456     3.43   0.001     1.020341    1.076582
          _cons |    .008535   .0082738    -4.91   0.000     .0012766    .0570637
------------------------------------------------------------------------------
Note: _cons estimates baseline odds.

------------------------------------------------------------------------------------------------------------------------------------
-------------------------------------------------
-> Sex = 2

Logistic regression                              Number of obs =    157
                                        LR chi2(2)    =   9.12
                                        Prob > chi2   = 0.0105
Log likelihood = -98.849603                      Pseudo R2     = 0.0441

------------------------------------------------------------------------------
  Bastary_Satus | Odds ratio   Std. err.      z    P>|z|     [95% conf. interval]
----------------+-------------------------------------------------------------
newDAQS_Tertile |   1.873658   .4191516     2.81   0.005     1.208565    2.904762
            Age |   1.011665   .0123411     0.95   0.342     .9877643    1.036145
          _cons |   .1625821   .1283625    -2.30   0.021     .0345962    .7640418
------------------------------------------------------------------------------
Note: _cons estimates baseline odds.

. by Sex :logistic Bastary_Satus newDAQS_Tertile Age sfat linoleic iron biotin tfiber bcarote
```


-> Sex = 1

Logistic regression Number of obs = 138

LR chi2(8) = 43.88

Prob > chi2 = 0.0000

Log likelihood = -65.901136 Pseudo R2 = 0.2498

Bastary_Satus	Odds ratio	Std. err.	z	P>|z|	[95% conf. interval]	
newDAQS_Tertile	5.221775	2.032528	4.25	0.000	2.434982	11.198
Age	1.048766	.015917	3.14	0.002	1.018028	1.080431
sfat	1.065799	.0260027	2.61	0.009	1.016034	1.118001
linoleic	.9505926	.0472992	-1.02	0.309	.8622648	1.047968
iron	.9922434	.0176968	-0.44	0.662	.9581575	1.027542
biotin	1.02672	.0308707	0.88	0.380	.9679624	1.089044
tfiber	.9878704	.0248864	-0.48	0.628	.9402786	1.037871
bcarote	1.000147	.0001529	0.96	0.335	.9998477	1.000447
_cons	.0007272	.0011017	-4.77	0.000	.0000373	.0141644

Note: _cons estimates baseline odds.

-> Sex = 2

Logistic regression Number of obs = 157

```
                                          LR chi2(8)    =  11.68
                                          Prob > chi2   = 0.1659
Log likelihood = -97.566316               Pseudo R2     = 0.0565

---------------------------------------------------------------------------------
  Bastary_Satus | Odds ratio   Std. err.     z    P>|z|     [95% conf. interval]
----------------+----------------------------------------------------------------
newDAQS_Tertile |   2.034182   .5731313    2.52   0.012     1.171018    3.533589
            Age |   1.012134    .012645    0.97   0.334     .9876512    1.037224
           sfat |   1.013033   .0196909    0.67   0.505     .9751653    1.052371
       linoleic |   1.015654   .0371932    0.42   0.671     .9453114    1.091231
           iron |   1.000077   .0105793    0.01   0.994      .979555    1.021028
         biotin |   .9945097   .0193471   -0.28   0.777     .9573039    1.033161
         tfiber |   .9861092   .0161715   -0.85   0.394     .9549175     1.01832
        bcarote |   1.000057   .0000816    0.70   0.481     .9998976    1.000217
          _cons |   .1300314   .1398726   -1.90   0.058     .0157916    1.070709
---------------------------------------------------------------------------------
Note: _cons estimates baseline odds.

.
.
.
.
end of do-file

. log close
      name:  <unnamed>
       log:  D:\Ms.Aghajani-6.14\Untitled.smcl
  log type:  smcl
```

closed on: 13 May 2023, 10:30:10

